# Identification of a Privileged Scaffold for Inhibition of Sterol Transport Proteins through the Synthesis and Ring Distortion of Diverse, Pseudo-Natural Products

**DOI:** 10.1021/acscentsci.4c01657

**Published:** 2025-01-09

**Authors:** Frederik Simonsen Bro, Laura Depta, Nienke J. Dekker, Hogan P. Bryce-Rogers, Maria Lillevang Madsen, Kaia Fiil Præstegaard, Tino Petersson, Thomas Whitmarsh-Everiss, Mariusz Kubus, Luca Laraia

**Affiliations:** Department of Chemistry, Technical University of Denmark, 2800, Kongens Lyngby, Denmark

## Abstract

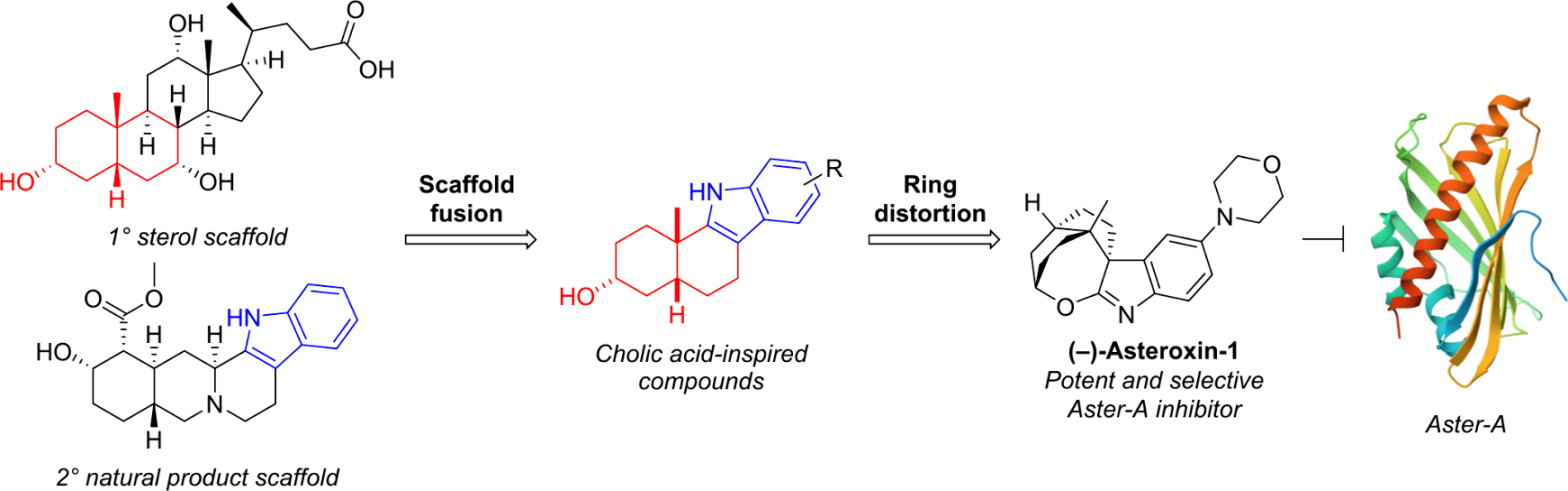

Sterol transport proteins mediate intracellular sterol transport, organelle contact sites, and lipid metabolism. Despite their importance, the similarities in their sterol-binding domains have made the identification of selective modulators difficult. Herein we report a combination of different compound library synthesis strategies to prepare a cholic acid-inspired compound collection for the identification of potent and selective inhibitors of sterol transport proteins. The fusion of a primary sterol scaffold with a range of different fragments found in natural products followed by various ring distortions allowed the synthesis of diverse sterol-inspired compounds. This led to the identification of a complex and three-dimensional spirooxepinoindole as a privileged scaffold for sterol transport proteins. With careful optimization of the scaffold, the selectivity could be directed toward a single transporter, as showcased by the development of a potent and selective Aster-A inhibitor. We suggest that the combination of different design strategies is generally applicable for the identification of potent and selective bioactive compounds with drug-like properties.

## Abstract 2

A privileged scaffold for sterol transporters was identified through fusion of a sterol scaffold and natural product fragments followed by ring distortions, leading to a selective Aster-A inhibitor.

## Introduction

Sterol transport proteins (STPs) are important mediators of nonvesicular transport of sterols, possessing distinct tissue distributions, intracellular localization and functions, and substrate binding. There are three main families of STPs: the oxysterol-binding protein-related proteins (ORPs), the steroidogenic acute regulatory protein-related lipid transfer-related domain (STARD) proteins, and the Asters.^1−3^ STPs are potential targets for therapeutics against various types of cancers, neurodegenerative diseases, and viral infections due to their function in regulating organelle contact sites, lipid metabolism, autophagy, viral entry, and the mechanistic target of rapamycin (mTOR) signaling.^4−10^ Consequently, studies to address the biological functions of STPs are crucial. However, studying STPs with genetic perturbations such as gene knockout (KO) or knockdown (KD) are often met with functional redundancies where one (often unknown) STP can compensate for the loss of another.^11,12^ In contrast, chemical perturbations of STP functions by the use of small molecule inhibitors result in effects within minutes instead of hours or days, meaning that cells do not have time to adapt. Furthermore, STP inhibition with small molecules inhibits one specific function, while complete removal by KO/KD would alter other functions not mediated by the binding site for the inhibitor, potentially resulting in different phenotypes.^13^ This highlights the demand for new potent and selective small molecule inhibitors. Nonetheless, very few STP inhibitors have been reported, often with little or no selectivity annotations, while only covering a fraction of the STPs. These include inhibitors of the ORPs such as the natural product-derived ORPphilins, which target oxysterol binding protein (OSBP) and ORP4L with varying selectivity, while their binding to other STPs remains to be resolved.^14^ Recently, the oxybipins were identified as potent and selective OSBP inhibitors across a broad panel of STPs.^15^ The autogramins have been identified as Aster-A inhibitors^11^ and the astercins as Aster-C inhibitors,^16^ both being selective within the Aster family. No inhibitors of STARDs with evidence of binding have been reported to date. This highlights a significant gap in the field, and identification of new potent and selective molecules would help in understanding the biological functions of STPs and provide potential new therapeutics.

Seeking inspiration from natural products (NPs) is often beneficial when designing compound libraries to identify novel bioactive molecules.^17^ Several strategies to access NP-derived and -inspired libraries with various degrees of NP-character have been reported in the past couple of decades, including (privileged-substructure-based) diversity-oriented synthesis ((p)DOS),^18,19^ biology-oriented synthesis (BIOS),^20,21^ complexity-to-diversity (CtD),^22^ function-oriented synthesis (FOS),^23^ pseudo-natural product (PNP),^24,25^ pharmacophore-directed retrosynthesis (PDR),^26^ and dynamic retrosynthetic analysis (DRA).^27,28^ A common feature in several of these strategies, when used in target-based campaigns, is the use of a natural ligand for the target as a starting point for designing compounds. This general idea has also been used successfully to identify inhibitors of enzymes such as proteases,^29^ neuroamidases,^30^ and nucleosidases.^31^

**Figure 1:**
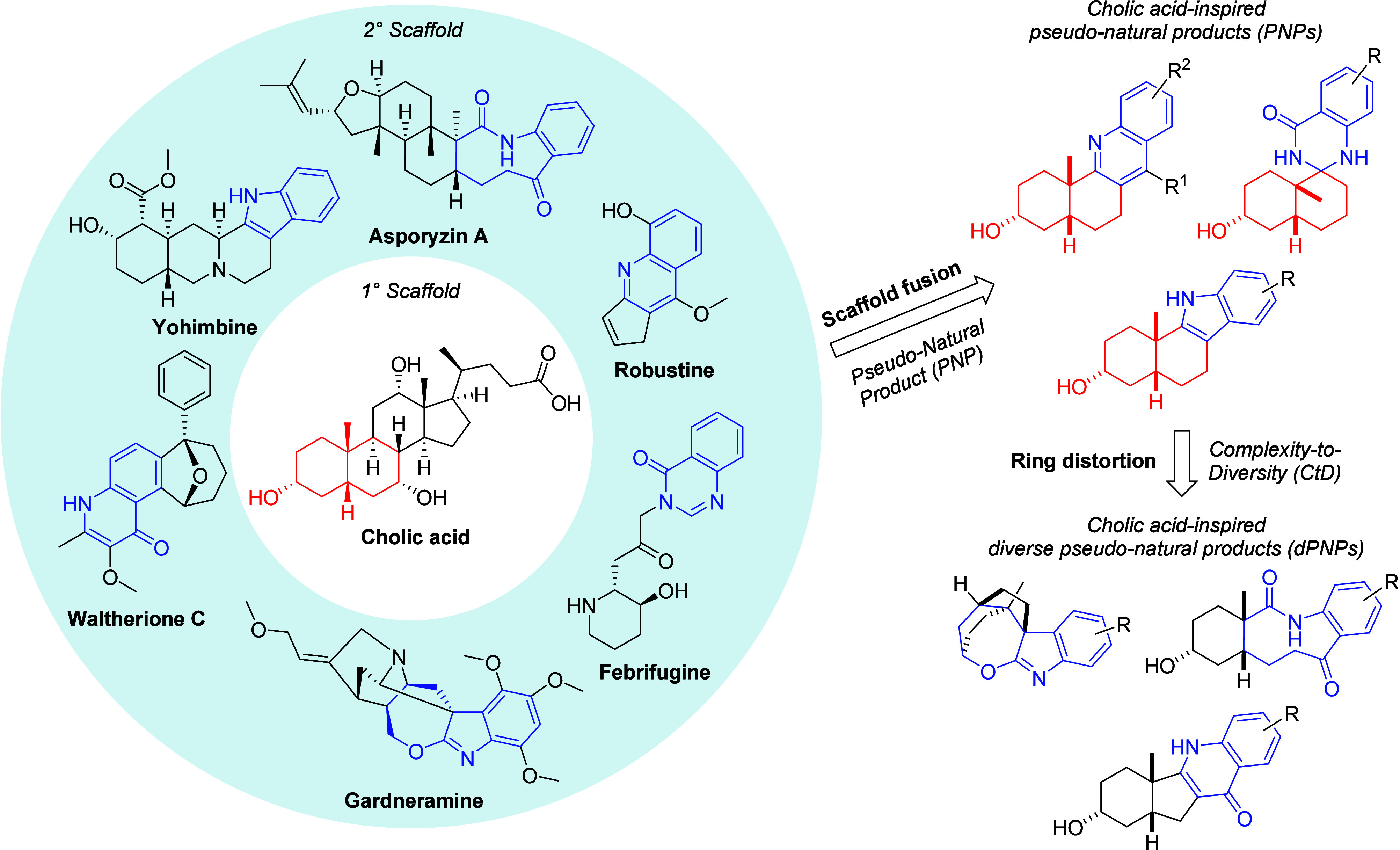
Cholic acid-inspired compounds were designed using a combination of the pseudo-natural product and complexity-to-diversity strategies. The fusion of a primary sterol scaffold as found in cholic acid (red) with secondary natural product scaffolds (blue) gives cholic acid-inspired analogues. Ring distortion of some of the resulting pseudo-natural products affords additional analogues.

The PNP strategy has proven efficient in identification of bioactive molecules including inhibitors of STPs, as seen in our previous work centered on employing a *trans*-decalin primary fragment as a sterol-mimicking moiety.^16,32,33^ We reasoned that the hydroxy *cis*-fused decalin scaffold as found in cholic acid could also be used as primary sterol scaffold to act as “bait” for STPs, while providing a significantly altered three-dimensional structure, which may introduce new activity and selectivity. In that regard, diastereochemical diversity of compound collections has been shown to give diverse biological profiles and activity.^34^ The primary scaffold could then be fused with natural product fragments or privileged scaffolds (the secondary scaffolds), resulting in novel heterocyclic edge- and spiro-fused cholic acid-inspired analogues. It was reasoned that additional analogues could be accessed by ring distortions^35^ of the resulting pseudo-natural products using the CtD approach to give so-called *diverse* pseudo-natural products (dPNPs).^36^ Thus, the compound design would be a combination of the PNP and CtD strategies (Figure 1), which would allow the synthesis of a broad range of scaffolds with structural diversity and complexity and a high degree of three-dimensionality, which is correlated with better physicochemical properties and drug-likeness in the preclinical and clinical stages of drug design.^37,38^ Some guidelines for the synthesis were established in order to optimize the process: The primary scaffold should be available in good yield and selectivity on a gram scale. The secondary scaffold in the initial fusion should be a scaffold found in a natural product or an established privileged scaffold. The novel PNPs should be available in one to three steps from readily accessible starting materials. Furthermore, the additional ring distortions of appropriate PNPs should be limited to one to two additional steps. The goal was to synthesize three to five analogues of each scaffold with both electron-rich and electron-poor groups and different substitution patterns in order to establish early structure–activity relationships (SARs) of potential hits. Lastly, the compounds should be synthesized as racemic mixtures to reduce bias and obtain twice the number of compounds for biological screening.

Herein, we report the synthesis of a novel cholic acid-inspired compound collection. The compounds are derived from the *cis*-decalin AB-ring system as found in cholic acid, and the collection includes both heterocyclic edge- and spiro-fused compounds. In addition, ring-distorted compounds were also accessed to cover new areas of the chemical space. We show that the combination of different strategies for compound design can be advantageous in identifying modulators of STPs. Through the screening of the compounds by fluorescence polarization (FP), differential scanning fluorimetry (DSF), and cholesterol transport assays, the spirooxepinoindoles were identified as a new general privileged scaffold for STPs where the selectivity can be directed through careful optimization showcased by the identification of a new chemotype Aster-A inhibitor with unprecedented potency and selectivity.

## Results and Discussion

**Scheme 1:**
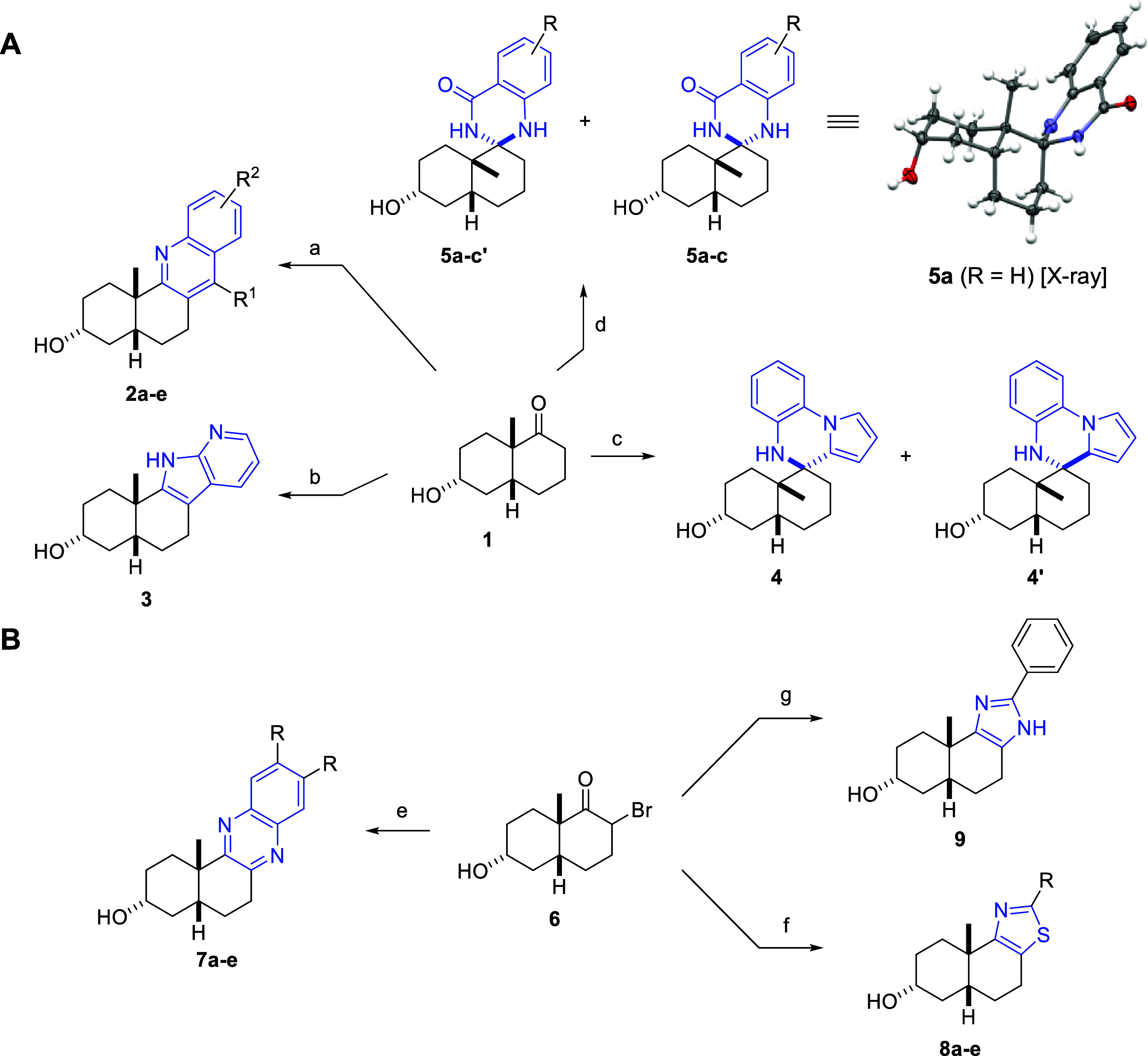
Synthesis of Edge- and Spiro-Fused Analogues. **A)** Analogues from *cis*-Decalone **1**. **B)** Analogues from α-Bromoketone **6** Conditions: **A:a)***o*-aminoaceto- or benzophenones, *p*-TsOH·H_2_O, neat, MWI, 110 °C, 0.5–2 h, 18–81%; **b)** 2-hydrazinopyridine, DEG, MWI, 250 °C, 3 h, 49%; **c)** 1-(2-aminophenyl)pyrrole, TfOH, THF, sealed tube, 100 °C, 21.5 h, 41% (**4**), 8% (**4'**); **d)** anthranilamides, NH_4_Cl, EtOH, reflux, 48 h, 19–32% (**5a-c**), 19-30% (**5a-c'**). **B:e)***o*-phenylenediamines, air, EtOH to neat, reflux to 88 °C, 1–5 d, 30–40%; **f)** thioamides, EtOH, reflux, 1.5–6 h, 15–58%; **g)** benzamidine, K_2_CO_3_, MeCN, reflux, 16 h, 19%

The synthesis of the compound collection began with the *cis*-fused decalone **1**, which was synthesized in six steps with 41% overall yield and high degree of diastereoselectivity from commercially available starting materials (Scheme S1). Initially, a range of edge- and spiro-fused heterocyclic analogues were synthesized directly from the *cis*-decalone **1** in one step (Scheme 1A). A number of quinoline-fused analogues **2a**–**e** were obtained by reacting the appropriate *o*-aminoaceto- or benzophenone in a solvent-free microwave irradiation (MWI) assisted Friedländer quinoline synthesis.^16^ A single example of the azaindole **3** was isolated via the Fischer azaindole synthesis by MWI heating the ketone with the 2-hydrazinopyridine in diethylene glycol.^16^ The spiropyrroloquinoxalines **4** and **4′** were isolated as separable diastereoisomers inspired by reported conditions^39^ by reacting the ketone with 1-(2-aminophenyl)pyrrole using triflic acid as the acid catalyst instead of the standard trifluoroacetic anhydride (TFAA) catalyst which resulted in sluggish conversion. The relative stereochemistry was determined by nuclear Overhauser effect spectroscopy (NOESY). The products proved to be quite sensitive to air and silica gel; thus, only one example was synthesized. The reaction with anthranilamides and NH_4_Cl in refluxing ethanol resulted in the corresponding spirodihydroquinazolinones **5a**–**c** and **5a–c′**(16) as a mixture of diastereoisomers which could be separated upon purification by flash column chromatography. Determination of the relative configuration of the diastereoisomers was guided by observed nuclear Overhauser effects (NOEs), and the structure of **5a** was ultimately confirmed by single-crystal X-ray diffraction.

To access additional scaffolds, the α-bromoketone **6** was synthesized from the ketone using 5,5-dibromobarbituric acid^40^ (Scheme S1) with the β-Br diastereoisomer as the major product (Figures S1 and S2 and accompanying discussion). This new core scaffold allowed for the synthesis of three additional scaffold fusions (Scheme 1B). The quinoxaline-fused analogues **7a**–**e** were synthesized by slowly concentrating a mixture of the α-bromoketone **6** and the appropriate *o*-phenylenediamine in ethanol in a reaction vessel open to air. In addition, refluxing the α-bromoketone with thioamides in ethanol yielded the thiazole-fused analogues **8a**–**e** in a Hantzsch thiazole synthesis.^16^ Lastly, one example of the imidazole-fused analogue **9** was achieved from benzamidine in refluxing acetonitrile using K_2_CO_3_ as a base.

**Scheme 2:**
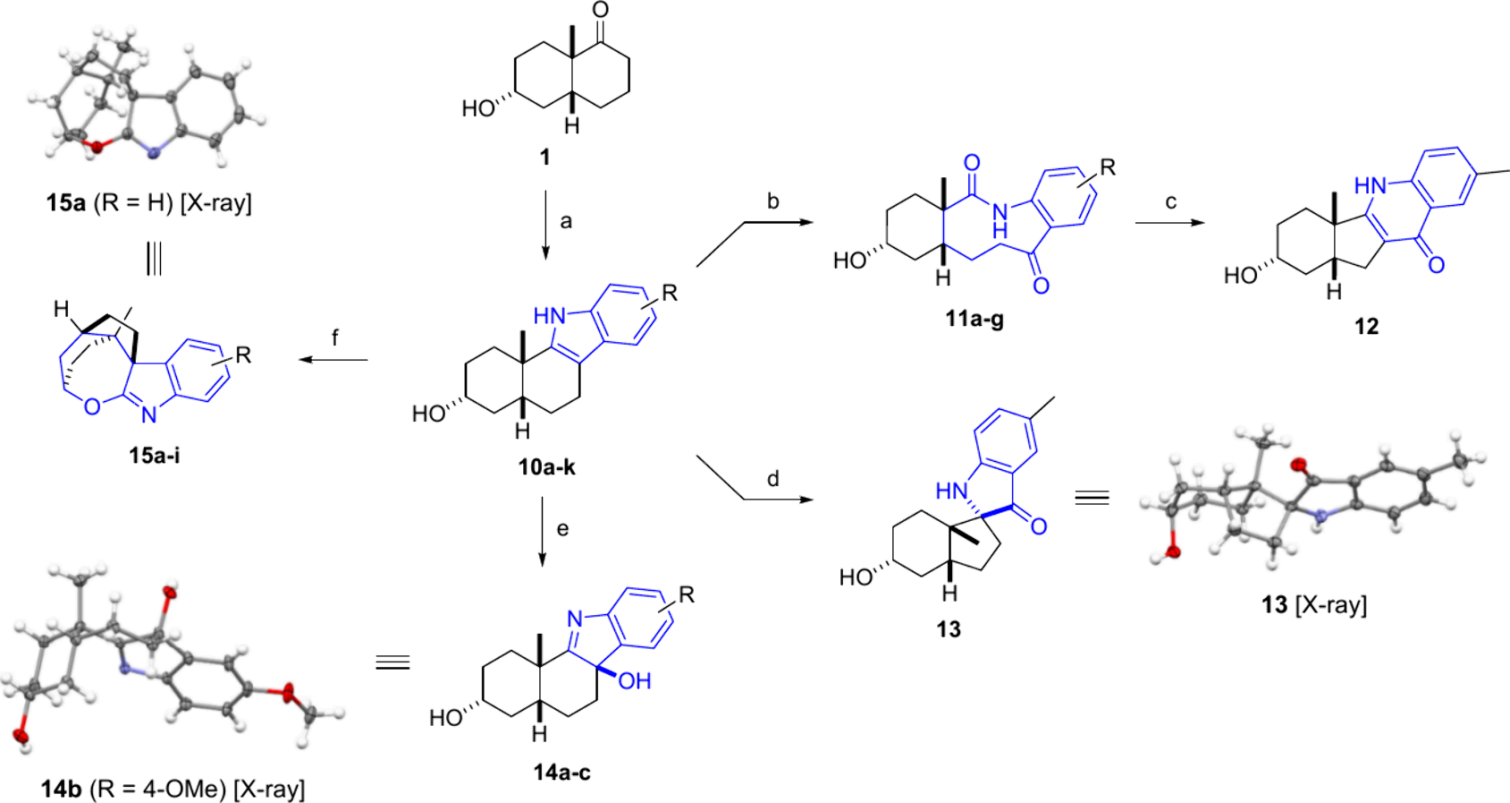
Synthesis of Indole Analogues and Indole-Derived Analogues Conditions: **a)** phenylhydrazines, *p*-TsOH·H_2_O, EtOH, reflux, 6–74 h, 3–98%; **b)** NaIO_4_, 1:1 H_2_O/MeOH, 0 °C to rt (then 50 °C), 22–48 h, 36-91%; **c)** KOH, EtOH, rt, 2 h, 78%; **d)** Oxone, KBr, 1:1:1 H_2_O/AcOH/THF, 0 °C to rt, 71 h, 24%; **e)** Oxone, KBr, 10:1 MeCN/H_2_O, 0 °C to rt, 19 h, 16–29%; **f)** NBS *or* NCS, 1:1:1 H_2_O/AcOH/THF, rt, 1–2 h, 8–58%.

In addition to the aforementioned edge-fused scaffolds, indoles were also targeted, since it was envisioned that they could serve as a suitable scaffold for a range of ring distortion reactions to afford additional analogues. Thus, the indole-fused analogues **10a**–**k** were synthesized directly from the *cis*-decalone **1** and the appropriate phenylhydrazine in one step via the Fischer indole synthesis using tosylic acid in refluxing ethanol (Scheme 2).^16^

We began our diversification of indole-fused scaffolds with a ring expansion by oxidative cleavage of the indoles to the ketolactams **11a**–**g** by employing Witkop oxidation using NaIO_4_ (Scheme 2). The treatment of a ketolactam with base afforded the corresponding ring-contracted quinolone-fused analogue **12** through the Camps cyclization. We subsequently sought to access a range of spirocyclic compounds through ring contraction reactions. Using Oxone in an acidic aqueous solvent system yielded the spiropseudoindoxyl **13** in moderate yields, with traces of ketolactam **11c** isolated. The relative configuration was determined by NOE analysis and confirmed by the X-ray crystal structure. In an attempt to access the spirooxindoles, similar reported conditions using Oxone and catalytic amounts of KBr in a neutral aqueous solvent system^41^ were attempted. The conditions did not give the desired product but instead the 3-hydroxyindolenine-fused analogues **14a**–**c** as a consequence of ring dearomatization through simple oxidation of the starting indole. The relative stereochemistry was determined by NOESY and ultimately confirmed by an X-ray crystal structure. In a final attempt to access spirooxindoles via an oxidative ring contraction of the indoles using *N*-bromosuccinimide (NBS) under acidic conditions, we were surprised to realize that the reaction yielded the spirooxepinoindoles **15a**–**i**. Several analytical observations of the isolated product suggested that the spirooxepinoindole had formed through condensation (transannular ring formation) of the spirooxindole, of which the most convincing was a heteronuclear multiple bond correlation (HMBC) between the C=N carbon and the C(H)-O proton (Table S1). Ultimately the structure was confirmed with single-crystal X-ray diffraction. The reaction performed with NBS was limited to electron-poor indoles and always produced variable amounts of the aromatic bromination side-products. With electron-rich indoles, almost exclusively aromatic bromination occurred. Different conditions were attempted; however, simply exchanging NBS with the less reactive *N*-chlorosuccinimide (NCS) proved to be the best solution for the problem, enabling the formation of the spirooxepinoindoles from electron-rich indoles (Tables S2 and S3).

**Figure 2:**
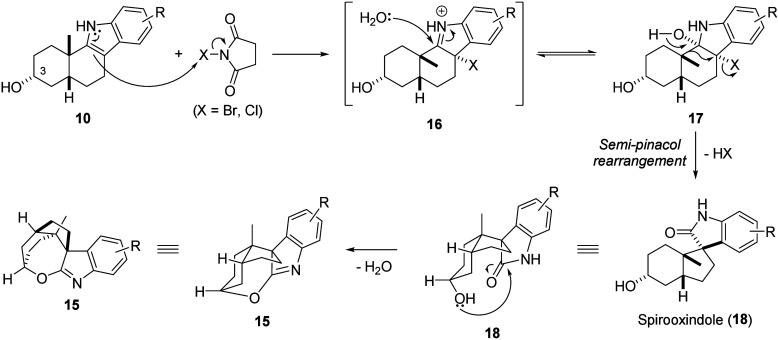
Tentative reaction mechanism for the synthesis of spirooxepinoindole **15**.

To explain the formation of the spirooxepinoindole rather than the expected spirooxindole, a putative reaction mechanism is outlined (Figure 2, see Figure S3 and the accompanying discussion for a more detailed mechanism). It is envisioned that a diastereoselective halogenation of **10** produced 3-haloindolenine **16**, which is then attacked by water to form 3-halo-2-hydroxyindoline **17**, followed by a diastereospecific semipinacol rearrangement to give the spirooxindole **18**. Due to the overall diastereoselectivity of the spirooxindole formation, the resulting amide carbonyl ends up on the *concave* face of the slightly folded *cis*-fused ring system. With the preinstalled hydroxy also on the *concave* face, the two functionalities are in close proximity, enabling a condensation to form the imidate functionality found in the spirooxepinoindole **15**. To exclude a mechanism where substitution at C3 is followed by nucleophilic attack from the lactam, the reaction was performed with oxygen-18 labeled water, which showed no incorporation of oxygen-18 in the final product, supporting the suggested pathway (Figures S4 and S5 and accompanying discussion).

The spirooxepinoindoles were stable in dimethyl sulfoxide (DMSO) for several months and in buffer system relevant for the biological screening for 20 h, showing no sign of degradation (Figures S6 and S7 and accompanying discussion). Treating the spirooxepinoindole with NaBH_4_ at 40 °C or diisobutylaluminum hydride (DIBALH) in refluxing dichloromethane showed no sign of conversion, which confirms the stability of this functionality (Figures S8 and S9 and accompanying discussion).

In total, the collection contained 57 compounds (Figure S10A) which can be grouped into 13 individual scaffold fusions, where 12 could be classified as PNPs and one as a privileged scaffold. The whole library was then screened in biophysical assays against a broad panel of the STPs using FP and DSF assays.^15,42,43^ Surprisingly, none of the edge-fused analogues showed significant activity against any of the STPs, suggesting that the *cis*-decalin ring system may not be tolerated. However, a large number of spirooxepinoindoles showed activity against a range of STPs (Table 1). Interestingly, **15a**–**i** all show activity against Aster-A with **15h** as the only exception. Aster-A mediates the sterol transport from the plasma membrane to the endoplasmic reticulum (ER).^44^ It is reportedly required in the early stages of autophagosome biogenesis, where it may play an important role in phagophore/autophagosome formation, by mediating cholesterol transfer between the ER and the forming phagophore.^11^ In that regard, inhibition of Aster-A using autogramins has resulted in inhibition of autophagosome biogenesis. Autophagy inhibition is a putative therapeutic strategy in cancer, as cancer cells often upregulate autophagy to generate nutrients for their continuous cell growth.^45^ Thus, Aster-A selective inhibitors would be of great value in continuing to probe this hypothesis. Additionally, cancer cells often induce autophagy in response to chemotherapy; thus, Aster-A inhibitors could potentially serve as chemotherapy sensitizers.^46^

**Table 1:**
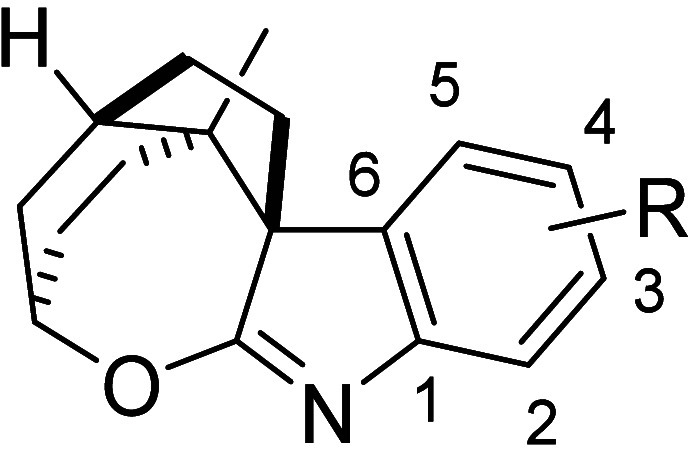
Potency of the Spirooxepinoindoles against a Broad Panel of STPs Determined by FP and DSF^a^

Entry	Compound	R	Aster-A IC_50_ (FP) [μM]^b^	Aster-B IC_50_ (FP) [μM]^b^	Aster-C IC_50_ (FP) [μM]^b^	ORP1 IC_50_ (FP) [μM]^b^	ORP2 IC_50_ (FP) [μM]^b^	STARD1 IC_50_ (FP) [μM]^b^	STARD3 K_d,app_ (DSF) [μM]^c^	STARD4 K_d,app_ (DSF) [μM]^c^	STARD5 K_d,app_ (DSF) [μM]^c^
1	**15a**	H	7.17	>80	>80	nd	nd	nd	nd	nd	>100
2	**15b**	4-Br	4.30	>80	>80	20.88	7.14	>80	nd	nd	nd
3	(−)-**15b**	4-Br	4.14	>80	>80	15.34	5.85	42.56	>100	6.04	>100
4	(+)-**15b**	4-Br	35.03	>80	>80	>80	>80	>80	>100	7.89	>100
5	**15c**	4-Cl	4.59	>80	>80	nd	22.18	nd	nd	nd	nd
6	**15d**	4-F	12.48	>80	>80	nd	nd	nd	nd	nd	nd
7	**15e**	4-Me	6.03	>80	>80	nd	nd	nd	nd	6.18	nd
8	**15f**	4-OMe	5.06	>80	>80	nd	nd^d^	nd	nd	nd	nd
9	**15g**	4-CF_3_	1.60	>80	>80	12.99	8.41	nd	nd	nd	nd
10	**15h**	2-Br,4-CF_3_	>80	>80	>80	>80	9.60	nd	nd	nd	nd
11	**15i**	2-F,4-Cl	3.63	>80	>80	nd	nd	nd	nd	nd	nd
12	**19a** ((±)-Asteroxin-1)	4-morph	0.77	>80	>80	>80	>80	>80	nd	>30	>30
13	(−)-**19a** ((−)-Asteroxin-1)	4-morph	0.46	>80	>80	>50	>50	>80	>100	>30	>30
14	(+)-**19a** ((+)-Asteroxin-1)	4-morph	15.45	>80	>80	>80	>80	>80	>100	>50	>100
15	**19b**	4-N(H)Bn	13.44	>80	>80	21.77	nd	nd	11.33	3.48	19.27
16	**19c**	4-N(H)*n*-Am	5.03	>80	>80	>80	>80	nd	9.54	4.06	20.85
17	**19d**	4-N(Me)*n*-Bu	3.89	>80	>80	6.26	>80	nd	>100	4.53	11.94
18	**19e**	4-pipz-N-Boc	1.25	>80	>80	nd	nd	nd	9.51	2.86	>100
19	**21a**	4-Ph	2.53	>80	>80	nd^d^	nd^d^	nd^d^	nd	nd	nd
20	**21b**	4-PMP	3.37	>80	>80	5.24	>80	nd	27.95	nd	>100
21	**22**	4-pipz	26.53	>80	>80	nd	nd	nd	nd	nd	nd
22	**23**	4-pipz-N-*n*-But	2.32	>80	>80	>80	nd	nd	15.76	4.00	>100
23	**24**	4-pipz-N–CH_2_(4′-Py)	5.33	>80	>80	>50	nd	nd	14.91	6.40	>100
24	U18666A^e^	n/a	2.32	0.93	3.54	>10	7.81	>10	>30	>30	>30
25	Autogramin-2	n/a	1.09	>80	>80	4.26	6.83	>80	>30	>30	>30

a IC_50_ and/or K_d,app_ values were not determined for compounds showing less than 50% inhibition in the FP assay and/or Δ*T*_m_ < ∼1.5 °C in the DSF assay in the single dose (10 μM) experiment. All the compounds showed less than 50% inhibition against OSBP in the single dose (10 μM) experiment (see Supporting data set).

b IC_50_ values are reported as the mean of three experiments run in technical duplicates.

c K_d,app_ values are reported as the mean of three experiments run in technical duplicates.

d Interfered with FP assay (see text and Figure S12).

e Data reproduced from Depta et al.^43^

**Scheme 3:**
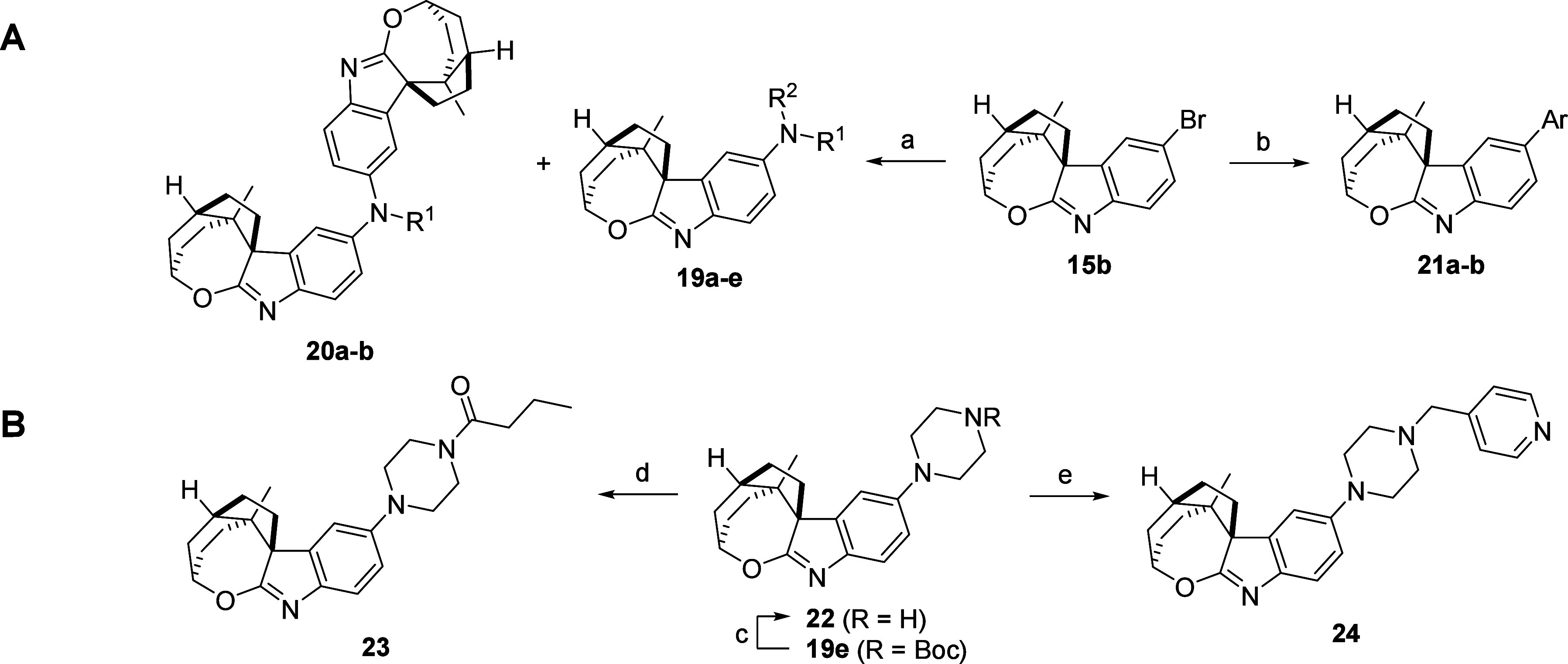
Synthesis for SAR Elucidation of Spirooxepinoindoles. **A)** Analogues from Bromo-spirooxepinoindole **15b**. **B)** Analogues from Boc-Piperazine-spirooxepinoindole **19e** Conditions: **A:a)** amines, Pd_2_(dba)_3_, XPhos, NaO*t*-Bu, PhMe, sealed tube, 80 °C (then 100 °C), 8–43 h, 42–99% (**19a**–**e**), 20–41% (**20a**–**b**); **b)** aryl boronic acids, Pd(PPh_3_)_4_, 2 M Na_2_CO_3_ (aq), PhMe, 80 °C, 18–40 h, 57–75%. **B:c)** TFA, DCM, 0 °C, 1 h then rt, 2 h, 53%; **d)** butyric acid, EDC·HCl, HOBt·H_2_O, DIPEA, DMF, rt, 23 h, 89%; **e)** isonicotinaldehyde, AcOH, DCM, rt, 30 min then NaBH(OAc)_3_, rt, 27 h, 44%.

From the initial nine analogues, several SARs could be determined (Table 1, see Figure S11 for a graphical summary of the SAR study). Substitution at the 4-position was generally tolerated for Aster-A activity, where larger substituents (-CF_3_, **15g**) showed enhanced activity. Interestingly, larger substituents also tended to show increased activity against two other sterol transporters, the highly homologous ORP1 and ORP2. The methyl-substituted analogue **15e** also showed moderate stabilization of STARD4. These results are particularly notable, as no synthetic ligands for ORP1/2 or STARD4 have been reported to date. At the 2-position, only small substituents were tolerated for Aster-A activity (-F, **15i**), while a bromide substituent (**15h**) gave a compound that was inactive against Aster-A and ORP1, but still retained activity against ORP2. These early trends provide indications of how the selectivity for a given STP can be engineered within one compound class. To further elucidate the SAR, a second series of compounds with a variety of substituents of different steric and electronic nature were prepared (Scheme 3).

The bromospirooxepinoindole **15b** was a useful starting point for cross-couplings (Scheme 3A). The aromatic amines **19a**–**e** were accessed by Buchwald-Hartwig cross-coupling with the corresponding amines, where primary amines also produced diarylated amines **20a**–**b** as side-products. The arylated spirooxepinoindoles **21a**–**b** were isolated using Suzuki-Miyaura cross-coupling conditions with the appropriate boronic acid. Furthermore, deprotection of Boc-piperazine-spirooxepinoindole **19e** afforded piperazine **22** (Scheme 3B). This analogue could be used in an amide coupling with butyric acid to give amide **23**, and a reductive amination with isonicotinaldehyde yielded analogue **24**.

**Figure 3:**
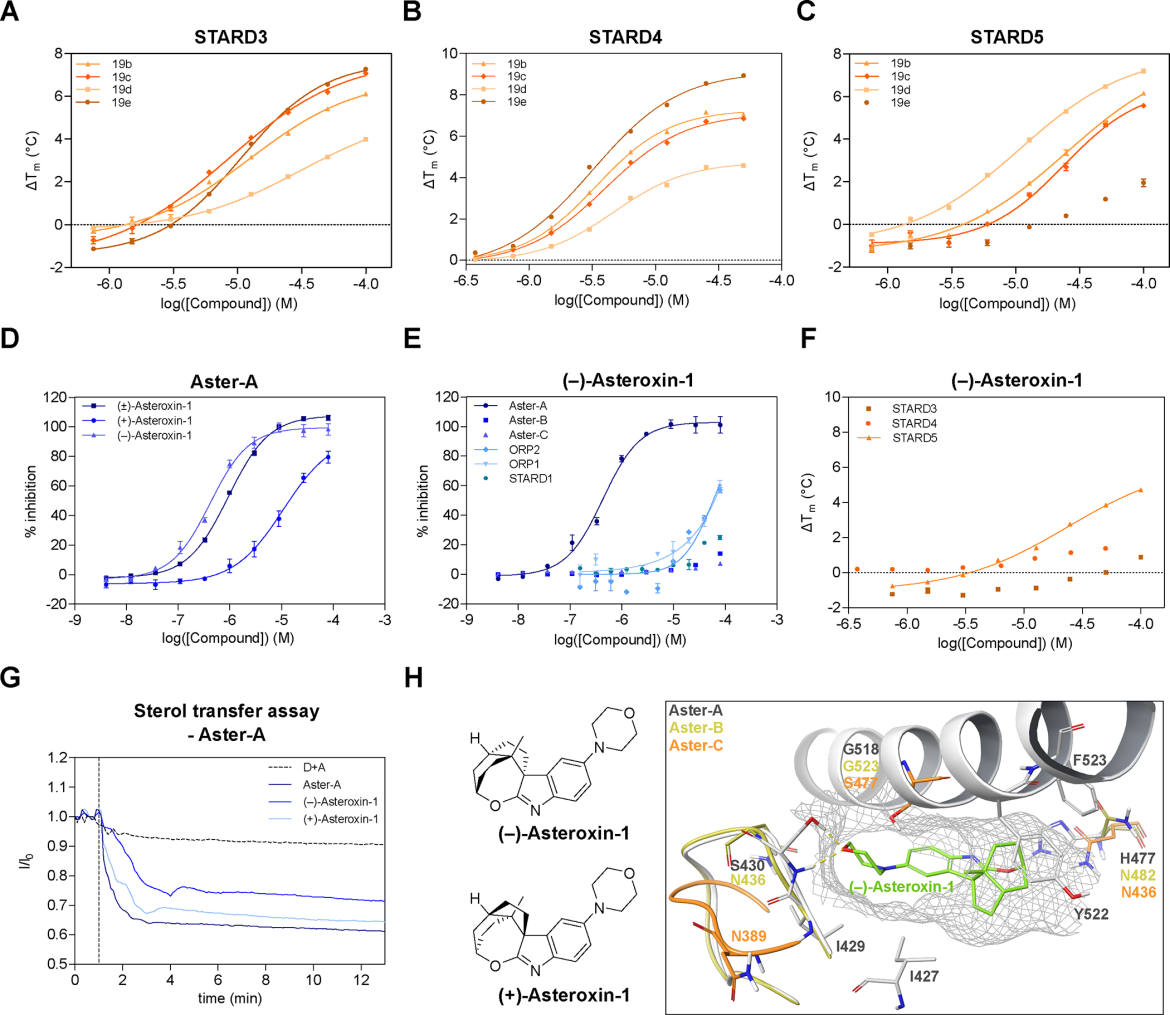
Biophysical characterization of spirooxepinoindoles. **A–C)** Dose-dependent stabilization of STARD3, STARD4 and STARD5 assessed by DSF. **D)** Comparison of the dose-dependent inhibition of Aster-A’s binding to 22-NBD-cholesterol by (±)-asteroxin-1, (+)-asteroxin-1 and (−)-asteroxin-1 assessed by FP measurements. **E)** Dose-dependent inhibition of STPs’ (Aster-A, Aster-B, Aster-C, ORP2, ORP1 and STARD1) binding to NBD-cholesterol probes by (−)-asteroxin-1 assessed by FP. **F)** Dose-dependent stabilization of STARD3, STARD4 and STARD5 by (−)-asteroxin-1 assessed by DSF. For **A–F)**, one representative experiment (*n* = 3) derived from three biologically independent experiments is shown. **G)** Inhibition of sterol transport mediated by Aster-A (125 nM) by enantiomers of asteroxin-1 (10 μM). One representative experiment is shown from two independent experiments (*n* = 2), D = donor liposomes and A = acceptor liposomes. The dotted line represents the addition of proteins and ligands to the liposomes. **H)** Chemical structures of (−)-asteroxin-1 and (+)-asteroxin-1 as well as the predicted binding pose of (−)-asteroxin-1 docked into the homology model of human Aster-A (based on the crystal structure of murine Aster-A (pdb: 6gqf)). The structure is aligned with the crystal structure of human Aster-C and a homology model of human Aster-B (based on pdb: 6gqf) to determine differences in the binding pocket responsible for differential selectivity profiles.

When tested against the panel of STPs, all compounds showed good activity against Aster-A, while varying activity against other STPs became apparent (Table 1 and Figure S11). In particular, amino-substituted analogues **19b**–**e** displayed promising affinity toward STARD3, -4, and -5 (Figure 3A–C). It must be noted that DSF assays were carried out with 5 μM of the respective proteins, and as such, apparent dissociation constants (K_d,app_) were often at the limit of detection of the assay. Therefore, we also report the maximal thermal shift (Δ*T*_m_^max^, see Supporting Data Set) to enable a better comparison between compounds. *N*-Benzyl and -*n*-amyl substituted analogues **19b** and **19c** showed good stabilization of all three STARDs, whereas methylation to produce a tertiary amine **19d** reduced the stabilization (Δ*T*_m_^max^) of STARD3/4, while enhancing that of STARD5 (Figure 3A–C). In contrast to the monoarylated amines, diarylated amines **20a**–**b** did not show any activity against the panel of STPs. Substituted piperazines **19e, 23** and **24** retained good stabilization of STARD3/4 but lost all activity against STARD5, suggesting that this transporter may not tolerate larger substituents. The free secondary amine **22** lost almost all activity, including against Aster-A, suggesting that charged groups are unsurprisingly not tolerated by the lipophilic sterol-binding sites. Finally, arylated products **21a**–**b** lost all activity against the STARDs, while retaining activity against Aster-A and ORP1. It should be noted that no IC_50_ values against STARD1, ORP1, and ORP2 could be measured for compound **21a**, nor for **15f** against ORP2 because they interfered with the FP assay. It was found that compound **21a** dose-dependently quenches the intrinsic fluorescence of STARD1, ORP1, and ORP2, and compound **15f** quenches the ORP2 protein fluorescence in a dose-dependent manner (Figure S12). Most notably, morpholine substituted spirooxepinoindole **19a** was identified as a very potent Aster-A inhibitor with a half maximal inhibitory concentration (IC_50_) of 0.77 μM and a very promising selectivity profile and was thus selected for further study, as well as being renamed (±)-asteroxin-1. This compound shows that with subtle changes in the substitution pattern, the selectivity can be directed and improved.

To determine the active enantiomer of (±)-asteroxin-1, enantioselective syntheses of (+)-asteroxin-1 and (−)-asteroxin-1 were carried out. Initially, enantioenriched Wieland-Miescher ketone (WMK), (+)-WMK and (−)-WMK were synthesized using chiral organocatalysts based on proline following published procedures^16,47,48^ where the rationale for enantioselectivity has been extensively investigated and explained.^49^ This allowed for the synthesis of (+)-**15b**, (−)-**15b**, (+)-asteroxin-1 and (−)-asteroxin-1 via the (+)-*cis*-decalone (+)-**1** and (−)-*cis*-decalone (−)-**1** following the same sequence as in the synthesis of (±)-asteroxin-1 (Scheme S2). Screening of the enantiomers revealed (−)-**15b** and (−)-asteroxin-1 as the active enantiomers (Table 1 and Figure 3D) with (−)-asteroxin-1 showing high potency (IC_50_ = 0.46 μM) and stabilization of Aster-A (Δ*T*_m_^max^ = 7.5 °C). Furthermore, (−)-**15b** maximally stabilizes STARD4 by 3.6 °C, 2-fold higher than (+)-**15b** (at 1.4 °C), contrary to their equipotent apparent K_d_s. This emphasizes the value in having both dose dependent information and changes in stabilization from a single assay. Interestingly, (−)-asteroxin-1 is derived from the “unnatural” stereochemistry of the AB-ring in cholic acid, which further supports the design criteria of synthesizing the initial screening compounds as racemic mixtures. (−)-Asteroxin-1 shows exquisite selectivity for Aster-A over all other STPs tested, with no measurable activity against any other STP other than at high concentrations against ORP1 and ORP2 (IC_50_ > 50 μM, Figure 3E), and weak stabilization of STARD5 (>30 μM, Δ*T*_m_^max^ = 4.7 °C at 100 μM, Figure 3F). The potency and selectivity profile for (−)-asteroxin-1 in the broad panel of STPs is superior to other known Aster-A inhibitors, including the pan-Aster and Niemann-Pick C1 (NPC1) inhibitor U18666A^50,51^ and autogramin-2^11^ (Table 1). In addition to exhibiting slightly lower potency against Aster-A, they also have general issues with selectivity compared to (−)-asteroxin-1. In addition to inhibiting NPC1, U18666A exhibits similar activity toward all three Asters, and autogramin-2 shows activity against ORP1 and ORP2. Recently published Aster-A probes based on the autogramins showed a similar selectivity profile.^42^

(−)-Asteroxin-1 also inhibited the Aster-A mediated transport of 23-BODIPY-cholesterol (BODIPY-Chol) between synthetic liposomes, as assessed by a reduced decrease in Förster resonance energy transfer (FRET) signal with rhodamine 1,2-dihexadecanoyl-*sn*-glycero-3-phosphoethanolamine (Rh-DHPE, Figure 3G).^16,43^ To rationalize the excellent selectivity of (−)-asteroxin-1, we modeled its binding to the different Aster proteins (Figure 3H). (−)-Asteroxin-1 has a great spatial fit in the sterol-binding domain, including a close-proximity and noticeable dipole alignment of the oxygen (∼2.8 Å) and nitrogen (∼4.0 Å) of the spirooxepinoindole core scaffold with H477 and T475, respectively. Furthermore, the morpholine oxygen makes two key interactions with Ser430 at the lid of the pocket. The side chain interactions and spatial fit of the very bulky three-dimensional head resulted in a very precise retention of the pose orientation during docking simulations, supporting the possible binding mode. Notably, neither H477 nor S430 is present in Aster-B and -C. Furthermore, Aster-C contains a serine at position 477, which would create a steric clash with (−)-asteroxin-1, unlike the glycine found in Aster-A. In summary, the combination of the three-dimensional scaffold and the specific substitution on the aryl ring both contribute to the potency and selectivity observed.

**Figure 4:**
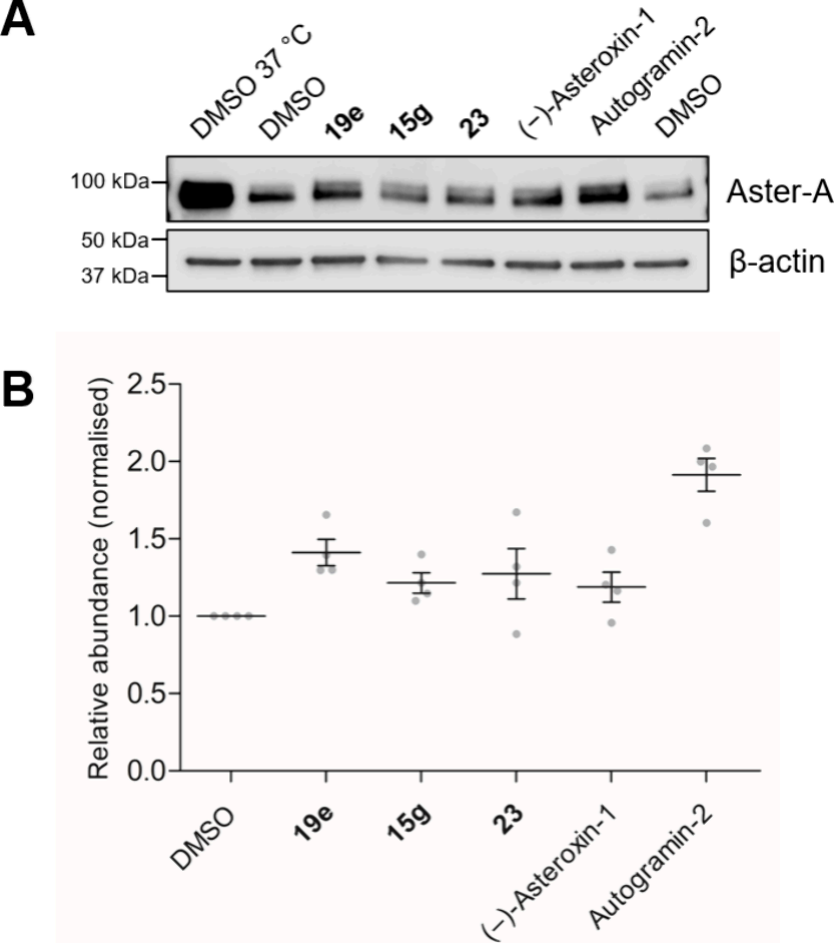
Aster-A target engagement by spirooxepinoindoles in intact Jurkat cells. **A)** Representative Western blot of cells treated with compounds (10 μM) for 1 h and heated to 50 °C, followed by centrifugation and removal of the insoluble fraction (see Figure S14B for uncropped Western blot). **B)** Quantification of four independent biological replicates, each normalized to their respective DMSO control. Data is mean ± sem, with individual replicates shown as gray circles.

As a first exploration of the utility of (−)-asteroxin-1 and analogues in a cellular setting, we conducted an isothermal shift assay in intact Jurkat cells.^15,52^ We initially determined the Aster-A melting temperature in Jurkat cells to be 46.7 °C (Figure S13) and subsequently selected 50 °C as the optimal temperature to measure compound-induced stabilization. We tested compounds **15g**, **19e**, **23**, and (−)-asteroxin-1 at 10 μM to cover a range of activities and to account for possible differences in cell permeability. All new spirooxepinoindoles and the control autogramin-2 showed stabilization of Aster-A toward thermal unfolding, to different degrees (Figure 4). Interestingly **19e**, which showed comparable potency to (−)-asteroxin-1 in the FP assays, showed increased stabilization of Aster-A in intact cells, in line with its increased stabilization in the *in vitro* DSF assay (Δ*T*_m_^max^ = 11.8 °C for **19e** and Δ*T*_m_^max^ = 7.5 °C for (−)-asteroxin-1, see Supporting data set). Further work aimed at correlating the target engagement and cellular activity with permeability, as well as optimizing all parameters, is ongoing and will be reported in due course.

## Conclusion

In conclusion, we have synthesized a cholic acid-inspired library consisting of 69 compounds with 13 distinct scaffolds (Figure S10), which were designed by the combination of the PNP and CtD strategy. Notably, by simply varying oxidative reaction conditions, four different scaffolds could be accessed from one readily accessible indole-fused *cis*-decalin ring system. The synthesis of an unprecedented spirooxepinoindole, through an oxidative ring contraction and intramolecular condensation cascade, led to its identification as a privileged scaffold for STPs with bioactivity against seven out of ten tested STPs. This highlights the benefits of the presented integrative approach including both PNP and CtD elements, as identification of the spirooxepinoindole scaffold would not have been possible through a PNP approach alone. In fact, the spirooxepinoindole displays a limited resemblance to the primary sterol scaffold. The spirooxepinoindole contains a highly 3D structure of high complexity, with the former being an unexpected feature for ligands of sterol-binding proteins. It can thus be classed as an interesting example of “escaping flatland”, where increasing three-dimensionality is predicted to correlate better with drug-like properties in preclinical and clinical development. The identification of (−)-asteroxin-1 as a new potent and selective chemotype Aster-A inhibitor showcased that with careful changes in the substituent on the spirooxepinoindole, the selectivity can be guided to a specific STP. This approach represents an enticing strategy for the further identification of potent and selective inhibitors of other sterol binding proteins in the future.

## Supporting Information Available

General directions, experimental procedures, characterization data, NMR spectra, IR spectra, LC-MS traces, chiral HPLC chromatograms, and X-ray structure reports (PDF)
Supporting data set with single dose screening data and maximal thermal shifts (XLSX)
Crystallographic data for **5a** (CCDC 2347999) (CIF)
Crystallographic data for **13** (CCDC 2345800) (CIF)
Crystallographic data for **14b** (CCDC 2345784) (CIF)
Crystallographic data for **15a** (CCDC 2345798) (CIF)
Transparent Peer Review report available (PDF)

## Supplementary Material

oc4c01657_si_002.xlsx

oc4c01657_si_003.cif

oc4c01657_si_004.cif

oc4c01657_si_005.cif

oc4c01657_si_006.cif

## Abbreviations

Δ*T*_m_^max^: maximal thermal shift
Ac: acetyl
BIOS: biology-oriented synthesis
BODIPY-Chol: 23-BODIPY-cholesterol
Bn: benzyl
Boc: *tert*-butyloxycarbonyl
Bt: benzotriazolyl
CCDC: Cambridge Crystallographic Data Centre
CtD: complexity-to-diversity
dba: dibenzylideneacetone
DCM: dichloromethane
DEG: diethylene glycol
DIBALH: diisobutylaluminum hydride
DIPEA: *N*,*N*-diisopropylethylamine
DMF: dimethylformamide
DMSO: dimethyl sulfoxide
DOS: diversity-oriented synthesis
dPNP: diverse pseudo-natural product
DRA: dynamic retrosynthetic analysis
DSF: differential scanning fluorimetry
DTU: Technical University of Denmark
EDC: 1-ethyl-3-(3-(dimethylamino)propyl)carbodiimide
ER: endoplasmic reticulum
Et: ethyl
FOS: function-oriented synthesis
FP: fluorescence polarization
FRET: Förster resonance energy transfer
HMBC: heteronuclear multiple bond correlation
HPLC: high-performance liquid chromatography
HRMS: high-resolution mass spectrometry
IC_50_: half maximal inhibitory concentration
ICP: inductively coupled plasma
IR: infrared
KD: knockdown
K_d,app_: apparent dissociation constant
KO: knockout
LC: liquid chromatography
Me: methyl
morph: morpholinyl
MS: mass spectrometry
mTOR: mechanistic target of rapamycin
MWI: microwave irradiation
n/a: not applicable
*n*-Am: *n*-amyl
NBD: nitrobenzoxadiazole
NBS: *N*-bromosuccinimide
*n*-Bu: *n*-butyl
*n*-But: *n*-butyryl
NCS: *N*-chlorosuccinimide
nd: not determined
NMR: nuclear magnetic resonance
NOE: nuclear Overhauser effect
NOESY: nuclear Overhauser effect spectroscopy
NP: natural product
NPC1: Niemann-Pick C1
ORP: oxysterol-binding protein-related proteins
OSBP: oxysterol-binding protein
Oxone: KHSO_5_·0.5KHSO_4_·0.5K_2_SO_4_
pDOS: privileged-substructure-based diversity-oriented synthesis
PDR: pharmacophore-directed retrosynthesis
Ph: phenyl
pipz: piperazinyl
PMP: *p*-methoxyphenyl
PNP: pseudo-natural product
*p*-Ts: *p*-tosyl
Py: pyridinyl
Rh-DHPE: rhodamine 1,2-dihexadecanoyl-*sn*-glycero-3-phosphoethanolamine
rt: room temperature
SAR: structure–activity relationship
sem: standard error of the mean
STARD: steroidogenic acute regulatory protein-related lipid transfer-related domain
STP: sterol transport protein
*t*-Bu: *tert*-butyl
Tf: triflyl
TFA: trifluoroacetic acid
TFAA: trifluoroacetic anhydride
THF: tetrahydrofuran
WMK: Wieland-Miescher ketone
XPhos: dicyclohexyl[2′,4′,6′-tris(propan-2-yl)[1,1′-biphenyl]-2-yl]phosphane
