# Supplementary material for: Identification of a Privileged Scaffold for Inhibition of Sterol Transport Proteins through the Synthesis and Ring Distortion of Diverse, Pseudo-Natural Products
Source: ACS Cent Sci. 2025 Jan 9;11(1):136–46. doi: 10.1021/acscentsci.4c01657 (PMC11758220; doi:10.1021/acscentsci.4c01657)
Supplement: Supplementary file 1 — oc4c01657_si_001.pdf [file oc4c01657_si_001.pdf]

Supporting information

Identification of a privileged scaffold for inhibition of sterol transport proteins through the synthesis and ring distortion of diverse, pseudo-natural products

Frederik Simonsen Bro,[1] Laura Depta,[1] Nienke J. Dekker,[1] Hogan P. Bryce-Rogers,[1] Maria Lillevang Madsen,[1] Kaia Fiil Præstegaard,[1] Tino Petersson,[1] Thomas Whitmarsh-Everiss,[1] Mariusz Kubus,[1] and Luca Laraia[1*]

[1]Department of Chemistry, Technical University of Denmark, 2800, Kongens Lyngby, Denmark
*Correspondence to: luclar@kemi.dtu.dk

Contents

Contents 1

Abbreviations 2

Supplementary discussions, figures, schemes, and tables 3

General directions 23

- Biology 23
- Chemistry 27

General procedures and characterisation data 30

- Synthesis of core scaffolds 30
- Synthesis of analogues 35
- Synthesis of enantioenriched compounds 89

NMR spectra 96

IR spectra 238

LC-LRMS traces 243

Chiral HPLC chromatograms 259

X-ray crystallographic data 268

References 276

Abbreviations

1D = one-dimensional, 2D = two-dimensional, Ac = acetyl, ATR = attenuated total reflectance, Bn = benzyl, Boc = *tert*-butyloxycarbonyl, brsm = based on recovered starting material, Bt = benzotriazolyl, Bz = benzoyl, calcd. = calculated, CCDC = Cambridge Crystallographic Data Center, COESY = correlation spectroscopy, conc. = concentration, dba = dibenzylideneacetone, DCM = dichloromethane, dec pt = decomposition point, DEG = diethylene glycol, DIBALH = diisobutylaluminium hydride, DIPEA = *N*,*N*-diisopropylethylamine, DMF = dimethylformamide, DMSO = dimethyl sulfoxide, DOPC = 1,2-dioleoyl-sn-glycero-3-phosphocholine, DPP = diphenyl phosphate, *dr* = diastereoisomeric ratio, DSF = differential scanning fluorimetry, DTT = dithiothreitol, *E. coli* = *Escherichia coli*, EDC = 1-ethyl-3-(3-dimethylaminopropyl)carbodiimide, EDTA = ethylenediaminetetraacetic acid, *ee* = enantiomeric excess, *er* = enantiomeric ratio, ESI = electrospray ionisation, Et = ethyl, FP = fluorescence polarisation, FRET = Förster resonance energy transfer, FT = Fourier-transform, GST = glutathione *S*-transferase, H2BC = heteronuclear two-bond correlation, HEPES = 4-(2-hydroxyethyl)-1-piperazineethanesulfonic acid, HMBC = heteronuclear multiple bond correlation, HMQC = heteronuclear multiple quantum coherence, HPLC = high-performance liquid chromatography, HRMS = high-resolution mass spectrometry, HRP = horseradish peroxidase, HSQC = heteronuclear single quantum coherence, IC_{50} = half maximal inhibitory concentration, ICP = inductively coupled plasma, ITPG = isopropyl β-D-1-thiogalactopyranoside, IR = infrared, LA = acceptor liposome, LB = Luria-Bertani broth, LC = liquid chromatography, LD = donor liposome, LRMS = low-resolution mass spectrometry, L-selectride® = lithium tri-*sec*-butylborohydride, Me = methyl, mol. sieves = molecular sieves, mp = melting point, MS = mass spectrometry, MW = molecular weight, MWI = microwave irradiation, *m*/*z* = mass-to-charge ratio, NBD = nitrobenzoxadiazole, NBS = *N*-bromosuccinimide, NCS = *N*-chlorosuccinimide, NIS = *N*-iodosuccinimide, NMR = nuclear magnetic resonance, NOE = nuclear Overhauser effect, NOESY = nuclear Overhauser effect spectroscopy, ORP = oxysterol-binding protein-related proteins, OSBP = oxysterol-binding protein, Oxone® = $KHSO_5 \cdot 0.5KHSO_4 \cdot 0.5K_2SO_4$, PBS = phosphate-buffered saline, PCR = polymerase chain reaction, PDB = Protein Data Bank, Ph = phenyl, ppm = parts per million, *p*-Ts = *p*-tosyl, QTOF = quadrupole time-of-flight, rel. = relative, Rh-DHPE = *N*-(lissamine rhodamine B sulfonyl)-1,2-dihexadecanoyl-sn-glycero-3-phosphoethanolamine, RP = reverse-phase, rt = room temperature, R_t = retention time, SAR = structure-activity relationship, SDS-PAGE = sodium dodecyl sulfate-polyacrylamide gel electrophoresis, SEC = size-exclusion chromatography, S_N1 = unimolecular nucleophilic substitution, S_N2 = bimolecular nucleophilic substitution, SP = standard precision, SQD = single quadrupole detection, STARD = steroidogenic acute regulatory protein-related lipid transfer-related domain, START = steroidogenic acute regulatory protein-related lipid transfer, TB = terrific broth, TBST = tris-buffered saline with Tween, *t*-Bu = *tert*-butyl, temp. = temperature, Tf = triflyl, TFA = trifluoroacetic acid, THF = tetrahydrofuran, TLC = thin-layer chromatography, TopFluor® cholesterol = 23-(dipyrrometheneboron difluoride)-24-norcholesterol, Tris = tris(hydroxymethyl)aminomethane, UHPLC = ultra-high-performance liquid chromatography, UPLC = ultra-performance liquid chromatography, UV = ultraviolet, VT = variable temperature, XPhos = dicyclohexyl[2′,4′,6′-tris(propan-2-yl)[1,1′-biphenyl]-2-yl]phosphane.

Supplementary discussions, figures, schemes, and tables

Synthesis of core scaffolds

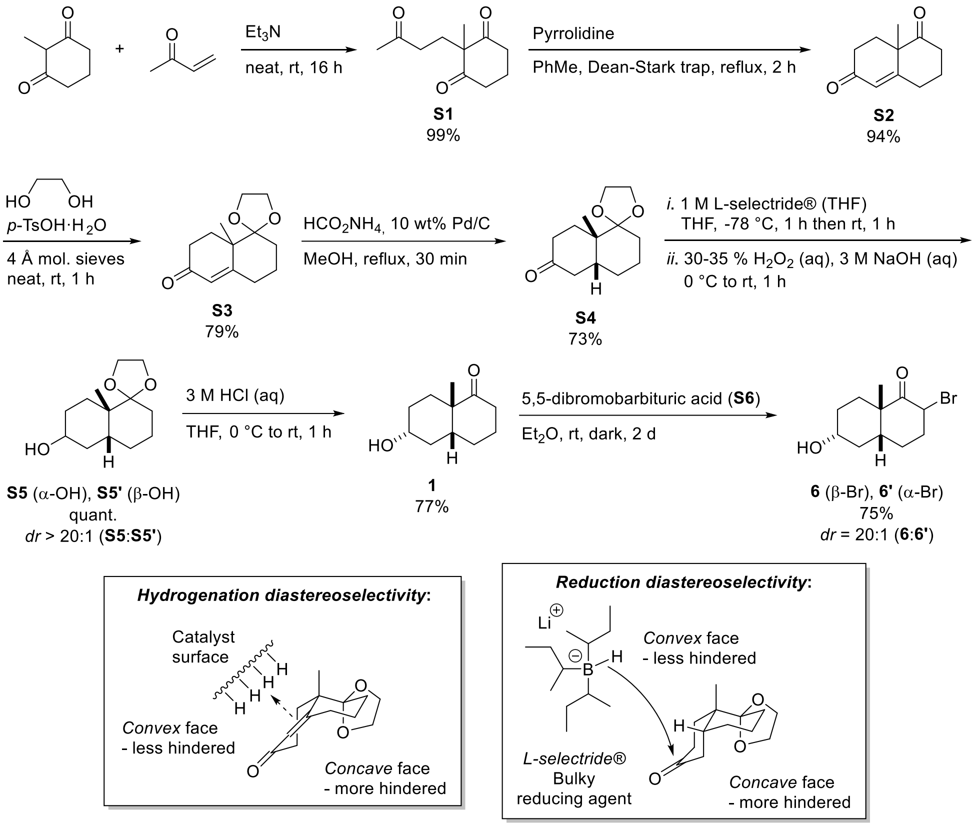

Scheme S1: Optimised synthesis of the *cis*-decalone **1** and α-bromoketone **6**/**6'** core scaffolds. Please see experimental procedures for further details.

Determination of major diastereoisomer of the α-bromoketone

By looking at the dihedral angles of protons H_a, H_b, and H_c in the chair confirmations and optimised structures of the diastereoisomers (Figure S1) it can be concluded that:

6: Angles approx. 55°.
6': Angles approx. 180° and 55°.

Using the Karplus relationship this will result in the estimate of the coupling constants and multiplicities:[1]

6: $^3J_{HaHb} \approx {}^3J_{HaHc} \rightarrow$ t.
6': $^3J_{HaHb} < {}^3J_{HaHc} \rightarrow$ dd.

This pattern is exactly what is observed in the NMR spectrum (Figure S2) which indicates that **6** (β-Br) is the major diastereoisomer. It should be noted that the result would be reversed if you ring-flip the diastereoisomers. However, it is observed that the shown chair conformation match the lowest energy conformation calculated by Chem3D®. In addition, no NOE is observed between the α-proton (H_a) and the methyl in the major diastereoisomer which suggests that **6** is the major diastereoisomer.

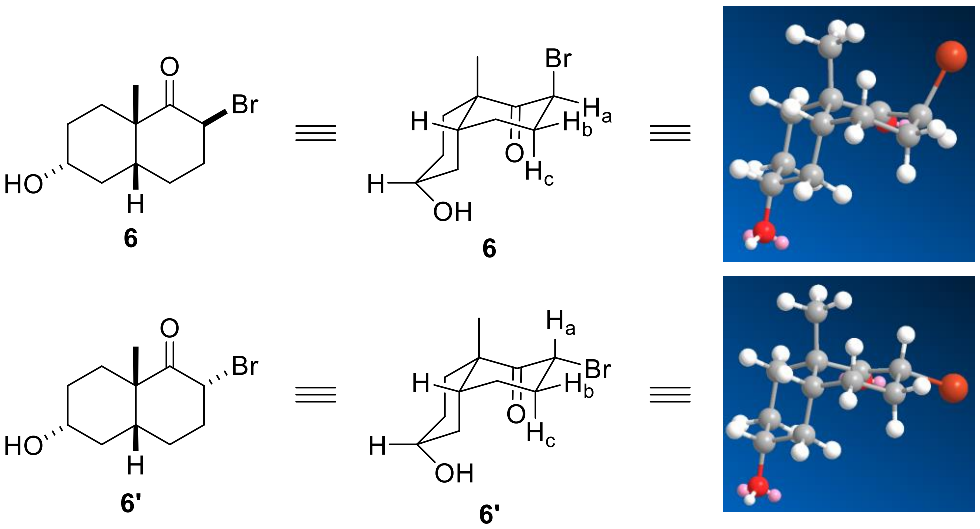

Figure S1: Chair conformations of the diastereoisomers of the α-bromoketone (**6** and **6'**) and their optimised structures (optimised with Chem3D®).

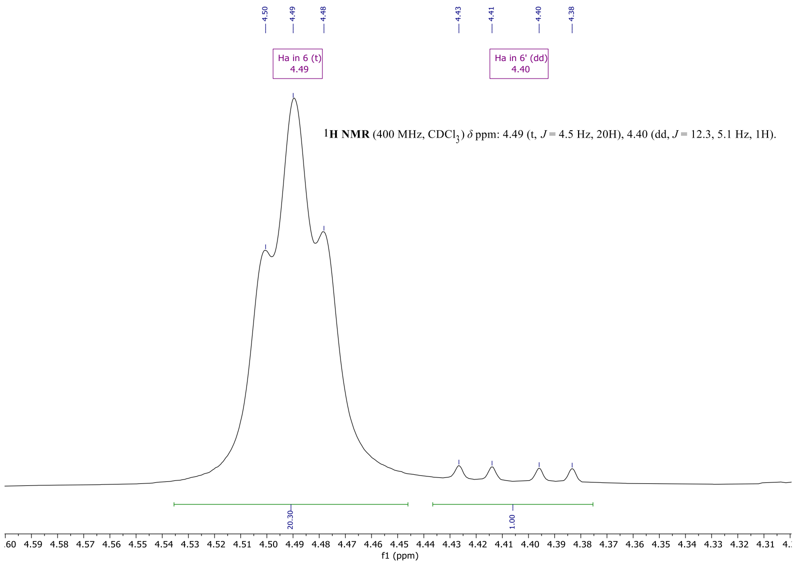

Figure S2: NMR peaks (including chemical shift, integral, multiplicity, and coupling constants) for the α-protons (H_a) in the two diastereoisomers of the α-bromoketone (**6** and **6'**).

Analytical observations for spirooxepinoindoles

Table S1: Different analytical observations that indicated that the forming spirooxindole condenses to the spirooxepinoindole **15**. Please see characterisation data for further details.

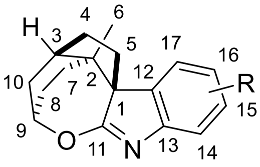

15

Analytical technique	Observations
MS	For all spirooxepinoindole analogues, both LRMS and HRMS data had a base peak with *m/z* = $[M\text{-}H_2O+H]^+$ where M is the exact mass of the expected spirooxindole (please see LC-LRMS and HRMS data for individual analogues and LC-LRMS traces for relevant analogues). This corresponds to the *m/z* = $[M+H]^+$ for the spirooxepinoindole.
IR	For all spirooxepinoindole analogues, a characteristic strong broad alcohol O-H stretch band around 3550 – 3300 cm^{-1} and a strong amide C=O stretch band around 1700 – 1640 cm^{-1} were absent[1] (please see IR data and spectra for individual analogues) which is in accordance with an amide-alcohol condensation.
NMR	For all spirooxepinoindole analogues, an HMBC between C9-H proton and C11 carbon was observed in the 2D ^1H-^{13}C HMBC spectrum. An NOE between the aromatic (C17-H) proton and the methyl (C6-H_3) protons in the 2D ^1H-^1H NOESY spectrum was observed which is in accordance with the relative stereochemistry of the only diastereoisomer of the spirooxindole that can condense. In addition, when performing NMR in dimethyl sulfoxide-d_6 for some of the analogues no O-H or N-H proton were observed. Lastly, a nitrogen chemical shift of ~260 ppm is too high for an amide nitrogen but fits with an imidate nitrogen[2] (please see NMR data and spectra for individual analogues).
X-ray	In addition to the above observations, the amide-alcohol condensation was ultimately confirmed by an X-ray crystal structure (please see X-ray data for **15a**).

Screening and optimisation of conditions for electron-rich indoles

Table S2: Screening and optimisation of the conditions for the synthesis of spirooxepinoindoles **15** from electron-rich indoles **10**.

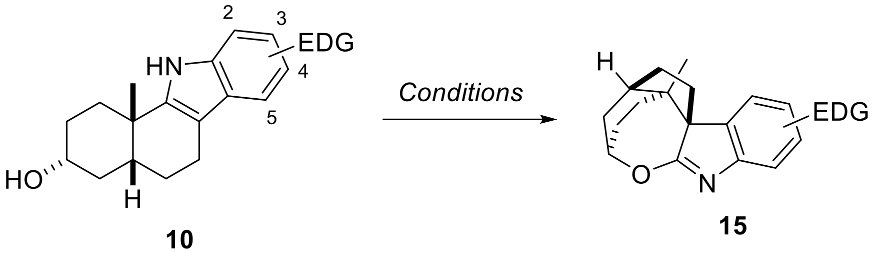

10 **15**

Entry	EDG	Conditions	Result[a]
1	4-OMe	0.1 equiv. KBr, 1.8 equiv. Oxone®, 10:1 MeCN/H_2O, 0 °C to rt, 17 h	Complex mixture including fair conversion into hydroxyindolenine **14b**
2	3,5-Me_2	0.1 equiv. KBr, 1.5 equiv. Oxone®, 10:1 MeCN/H_2O, 0 °C to rt, 21 h	Complex mixture including fair conversion into hydroxyindolenine **14c**
3	4-OMe	0.1 equiv. KBr, 1.2 equiv. Oxone®, 1:1:1 H_2O/AcOH/THF, 0 °C to rt, 6.5 h	Complex mixture
4	4-OMe	1.05 equiv., NBS, 1:1:1 H_2O/AcOH/THF, rt, 1 h	Primarily aromatic bromination of **10f**
5	3,5-Me_2	1.05 equiv., NBS, 1:1:1 H_2O/AcOH/THF, rt, 3.5 h	Primarily aromatic bromination of **10j**
6	4-OMe	1.1 equiv. NIS, 0.1 equiv. DPP, 35:1 THF/H_2O, -41 °C, 7 min then rt, 17 h	Exclusively aromatic iodination of **10f**
7	4-OMe	1. 1.5 equiv., $Pb(OAc)_4$, DCM, 0 °C to rt, 1 h; 2. 1 vol% AcOH, 5:1 MeOH/H_2O, reflux, 18 h	Complex mixture
8	4-Me	1.1 equiv. NCS, 20 mol% TFA, 3:1 MeOH/H_2O, 2 h	Fair conversion into **15e**
9	3,5-Me_2	1.55 equiv. NCS, 20 mol% TFA, 3:1 MeOH/H_2O, 2 h	Complex mixture including fair conversion into **15j**
10	4-Me	1.1 equiv. NCS, 2 mol% TFA, 231:1 MeOH/H_2O, 0 °C to rt, 2 h	Good conversion into **15e**
11	4-OMe	1.05 equiv. NCS, 2 mol% TFA, 231:1 MeOH/H_2O, 0 °C to rt, 20 h	Good conversion into **15f**
12	3,5-Me_2	1.05 equiv. NCS, 2 mol% TFA, 231:1 MeOH/H_2O, 0 °C to rt, 19 h	Poor conversion into **15j**
13	4-Me	1.1 equiv. NCS, 1:1:1 H_2O/AcOH/THF, rt, 1.5 h	Great conversion into **15e**

[a]Based on TLC, LC-LRMS, and NMR.

Table S3: Screening and optimisation of the conditions for the synthesis of spirooxepinoindole **15e** from electron-rich indole **15e** *via* the hydroxyindolenine **14a**.

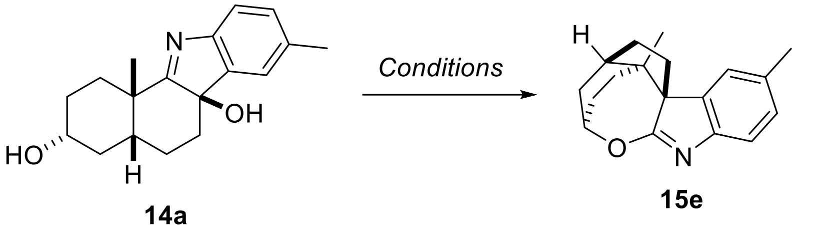

14a **15e**

Entry	Conditions	Result[a]
1	2 M HCl (aq), DCM, rt, 21 h	No/poor conversion into **15e**
2	25 equiv. $BF_3{\cdot}Et_2O$, DCM, sealed tube, 95 °C, 5 h	Complex mixture

[a]Based on TLC, LC-LRMS, and NMR.

Detailed mechanism for spirooxepinoindole formation

A detailed suggested mechanism of the presented mechanism in Figure 2 in the manuscript is presented in Figure S3. Initially, a diastereoselective halogenation of the indole occurs from the *concave* face to form the 3-haloindolenine **16**. A possible reason for the diastereoselectivity is that the nearby methyl doing a good job of blocking the otherwise preferred *convex* approach. This is followed by a diastereoselective reversible attack by water from the *concave* face to give the preferred *cis*-fused 6,5-membered ring to give the 3-halo-2-hydroxyindoline **17**. This then undergoes a diastereospecific semi-pinacol rearrangement to form the spirooxindole **18**. In this diastereoisomer of the spirooxindole, the resulting amide carbonyl ends up on *concave* face of the slightly folded *cis*-fused ring system. With the pre-installed hydroxy also on the concave face, the two functionalities are in close proximity, enabling a condensation to form the imidate functionality. The condensation can only occur with this diastereoisomer of the spirooxindole. In the other diastereoisomer, the carbonyl- and hydroxy-group are on opposite faces.

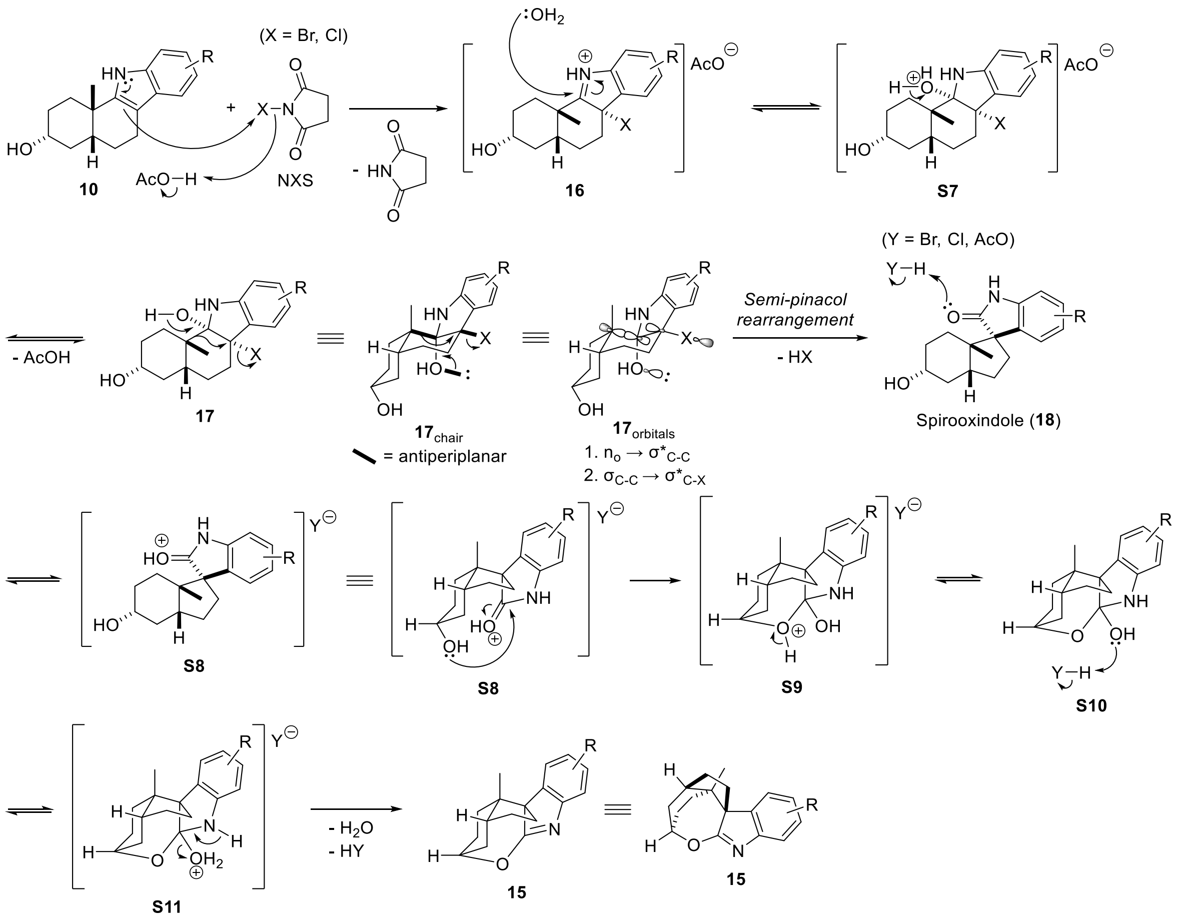

Figure S3: Detailed tentative reaction mechanism for the synthesis of spirooxepinoindoles **15**.

The diastereoisomer of the spirooxindole that can condense can only be formed from the specific diastereoisomer of the 3-halo-2-hydroxyindoline **17** since only in this diastereoisomer the bonds involved in the rearrangement are antiperiplanar. This is necessary for the rearrangement to occur, thus making the semi-pinacol rearrangement diastereospecific. The necessary overlap between the orbitals in play occurs when the bonds are antiperiplanar (**17**$_{chair}$). The lone pair on the hydroxy (n_o) donates into the antibonding orbital of the carbon-carbon bond (σ^*_{C-C}), and the bonding orbital of the carbon-carbon bond (σ_{C-C}) then donates into the antibonding orbital of the carbon-halogen bond (σ^*_{C-X}) (**17**$_{orbitals}$). The diastereoisomer of the 3-halo-2-hydroxyindoline that forms is determined by which diastereoisomer of the 3-haloindolenine that forms assuming the formation of the *cis*-fused 6,5-membered ring is highly preferred. Thus, the overall diastereoselective step is the halogenation since it determines which diastereoisomer of the spirooxindole is formed.

It is predicted that the amide carbonyl act as the electrophile and the alcohol as the nucleophile (**S8**). However, due to the general decreased electrophilicity of the amide carbonyl compared with other

carbonyls, another pathway with the amide acting as the nucleophile in a S_N2- or S_N1-like fashion could be imagined. However, the S_N2 is not possible at the alcohol since the required “back-attack” into the $σ^*_{C-O}$ orbital is impossible due to the stereochemistry. It would require an initial inversion of the stereogenic centre by another nucleophile (eg. Br^- or Cl^-) followed by the intramolecular S_N2. The S_N1-type mechanism would expel water under the acidic conditions to form the carbocation which is then attacked by the amide carbonyl. Both the S_N2- or S_N1-type pathways would require a labile alcohol. However, the alcohol proves stable under other acidic conditions such as the Fischer indole synthesis and Friedländer quinoline synthesis and epimerisation or dehydration of the alcohol is not an issue. Thus, these pathways seem unlikely and the pathway with the amide carbonyl acting as the electrophile is the most probable mechanism. Performing the reaction with oxygen-18 labelled water showed no incorporation of oxygen-18 in the final product which support the suggested pathway (please see mechanistic study with $H_2{}^{18}O$).

Mechanistic study with $H_2{}^{18}O$ of spirooxepinoindole formation

Procedure

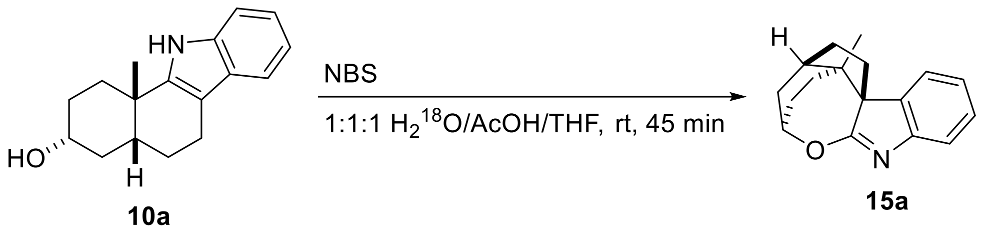

10a **15a**

A solution of compound **10a** (21.0 mg, 82.2 µmol, 1.00 equiv.) and *N*-bromosuccinimide (15.5 mg, 87.1 µmol, 1.05 equiv.) in 1:1:1 water-^{18}O/glacial acetic acid/tetrahydrofuran (1.4 mL) was stirred at room temperature for 45 minutes. The reaction mixture was analysed by LC-LRMS.

LC-LRMS data

Reaction after 30 minutes with $H_2{}^{18}O$

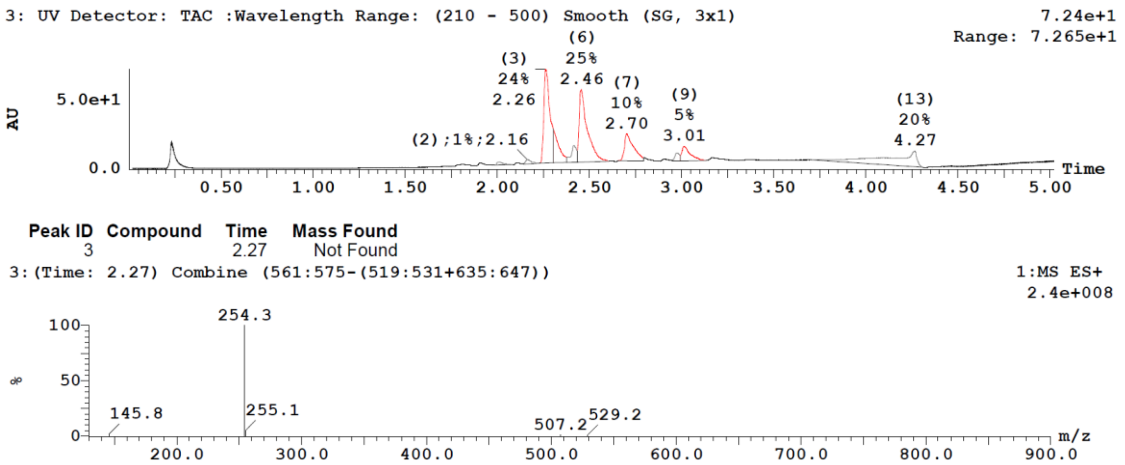

Figure S4: LC-LRMS trace of the reaction of compound **10a** and NBS in 1:1:1 water-^{18}O/glacial acetic acid/tetrahydrofuran after 30 minutes. Top: UV chromatogram, bottom: MS spectrum.

LC-LRMS (ESI+) observed *m/z*: 254.3 found for $[M+H]^+$ for **15a**[^{16}O], 254.2 calcd. for $C_{17}H_{20}N^{16}O^+$. Not observed: $[M+H]^+$ for **15a**[^{18}O], 256.2 calcd. for $C_{17}H_{20}N^{18}O^+$.

Reaction after 45 minutes with $H_2{}^{16}O$

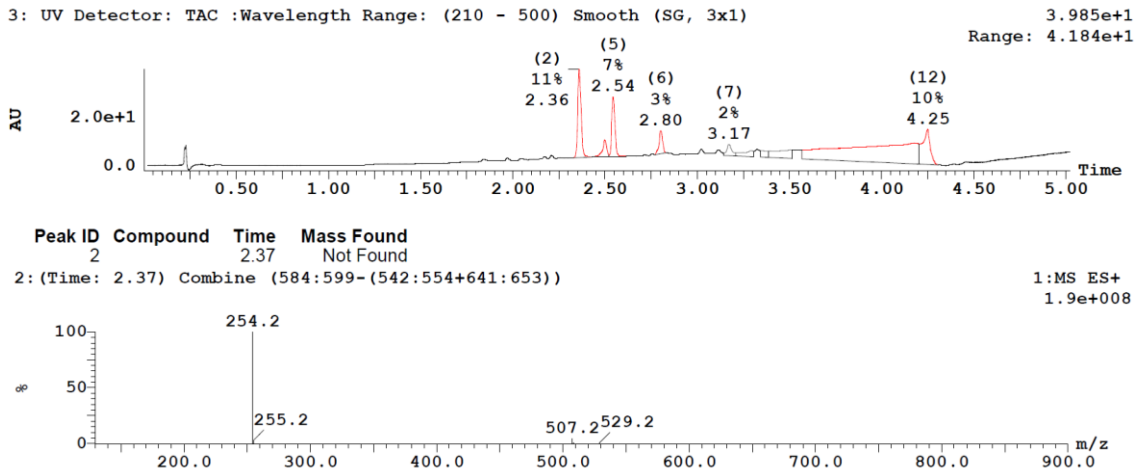

Figure S5: LC-LRMS trace of the reaction of compound **10a** and NBS in 1:1:1 water-^{16}O/glacial acetic acid/tetrahydrofuran after 45 minutes. Top: UV chromatogram, bottom: MS spectrum.

Conclusion

Performing the reaction with oxygen-18 labelled water showed no incorporation of oxygen-18 in the forming spirooxepinoindole product (Figure S4) and similar LC-LRMS data as the reaction with normal water (Figure S5) which support the suggested pathway with the amide carbonyl acting as the electrophile and the alcohol as the nucleophile. Incorporation of oxygen-18 in the product would be expected if the amide carbonyl acted as the nucleophile.

Stability test of spirooxepinoindole

Experiment

The stability of the spirooxepinoindoles in dimethyl sulfoxide and in the assay buffer was tested to be sure the imidate functionality did not hydrolyse under these conditions. A sample in the FP assay buffer was prepared from the dimethyl sulfoxide stock solution of compound **15b**. The dimethyl sulfoxide stock solution was prepared 2021.12.10, and the sample in the assay buffer was prepared 2022.09.28. The samples were monitored by LC-LRMS over time to look for degradation. Both acidic and basic LC-LRMS runs were used since acidic conditions could catalyse the re-condensation of potential hydrolysed spirooxepinoindoles.

Sample: Compound **15b** in FP assay buffer: 20 mM HEPES, 300 mM NaCl, 0.01 %v/v Tween-20, 0.5 %v/v glycerol, 2 mM DTT. Concentration: 1.7 mM.

LC-LRMS data

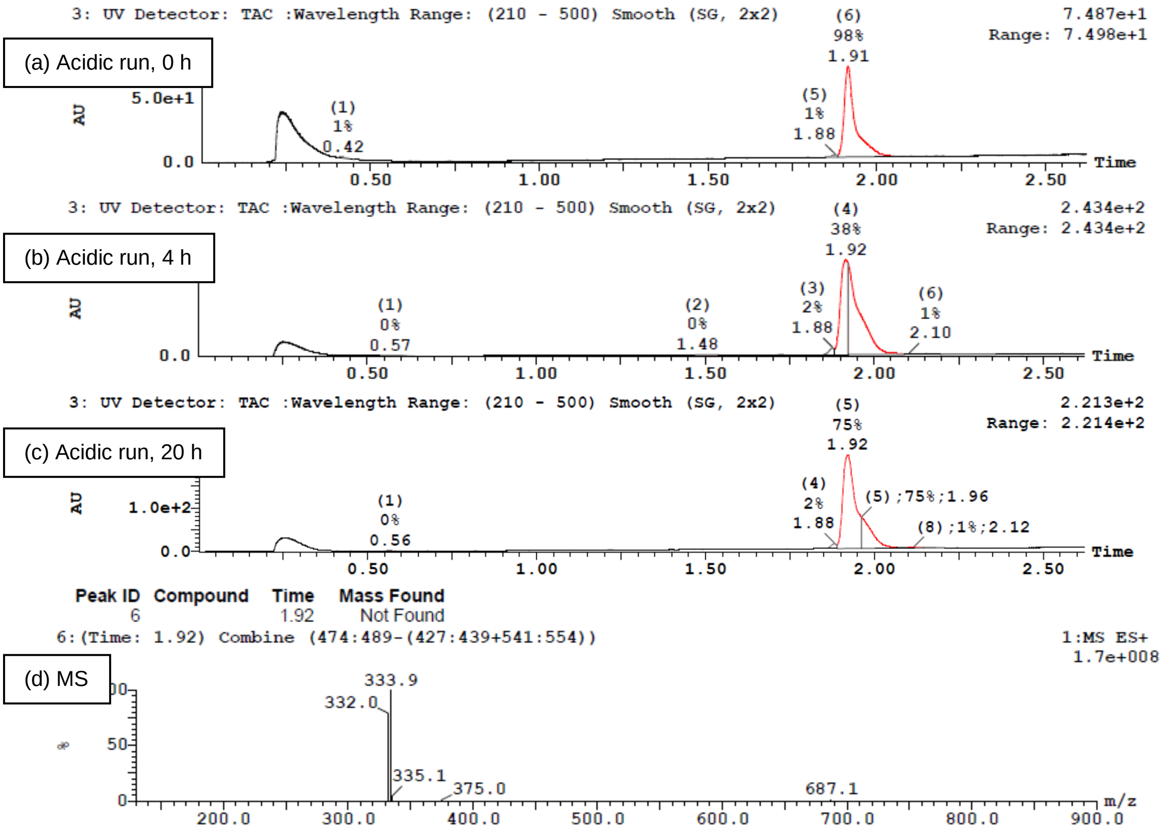

Figure S6: LC-LRMS trace of the stability test of spirooxepinoindole **15b** in FP assay buffer measured using the acidic run. (a) UV chromatogram after zero hours, (b) UV chromatogram after four hours, (c) UV chromatogram after 20 hours, (d) MS spectrum.

LC-LRMS (ESI+) *m/z*: 332.0 found for $[M+H]^+$ for **15b**, 332.1 calcd. For $C_{17}H_{19}BrNO^+$.

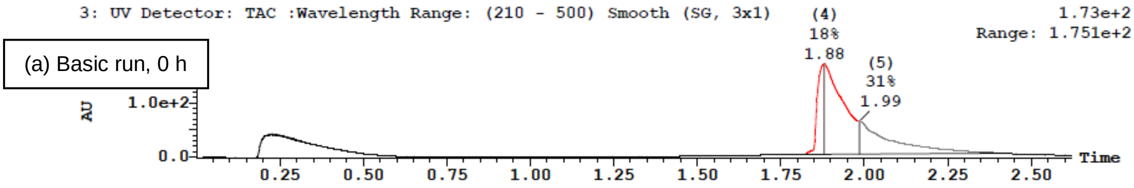

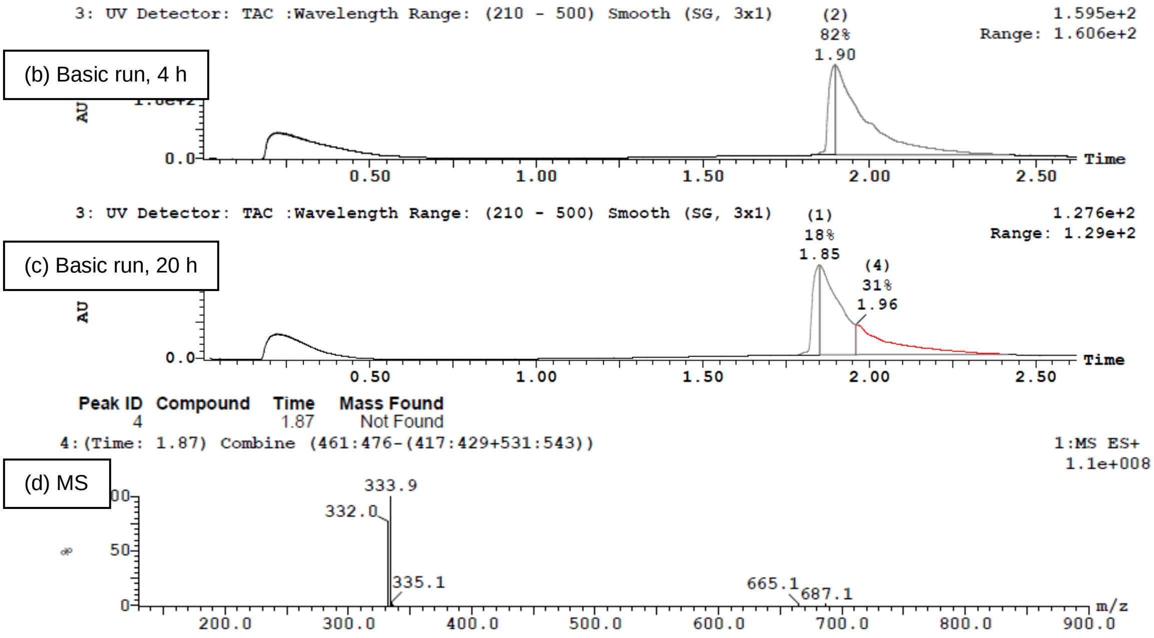

Figure S7: LC-LRMS trace of the stability test of spirooxepinoindole **15b** in FP assay buffer measured using the basic run. (a) UV chromatogram after zero hours, (b) UV chromatogram after four hours, (c) UV chromatogram after 20 hours, (d) MS spectrum.

LC-LRMS (ESI+) *m/z*: 332.0 found for $[M+H]^+$ for **15b**, 332.1 calcd. For $C_{17}H_{19}BrNO^+$.

Conclusion

The spirooxepinoindole **15b** was stable in dimethyl sulfoxide for several months and in the FP buffer for 20 hours showing no sign of degradation (Figure S6 and Figure S7).

Reaction of spirooxepinoindole with $NaBH_4$ and DIBALH

The spirooxepinoindole **15b** was reacted with $NaBH_4$ and DIBALH to try to reduce the C=N bond in the imidate functionality for SAR purposes.

Reaction with $NaBH_4$

Procedure

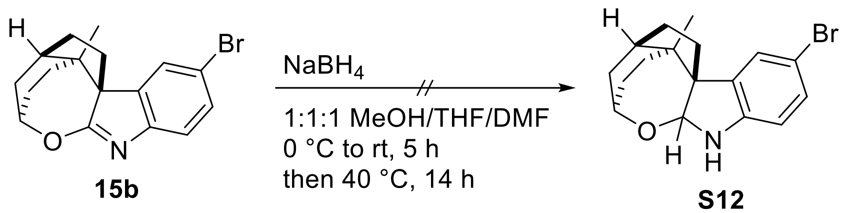

15b **S12**

A solution of compound **15b** (5.0 mg, 15 µmol, 1.0 equiv.) in 1:1:1 methanol/tetrahydrofuran/dimethylformamide (0.15 mL) at 0 °C was added $NaBH_4$ (1.8 mg, 48 µmol, 3.0 equiv.). The reaction mixture was stirred at room temperature for five hours. Then additional $NaBH_4$ (1.8 mg, 48 µmol, 3.0 equiv.) was added. The reaction mixture was stirred at 40 °C for 14 hours. The reaction was monitored by LC-LRMS.

LC-LRMS data

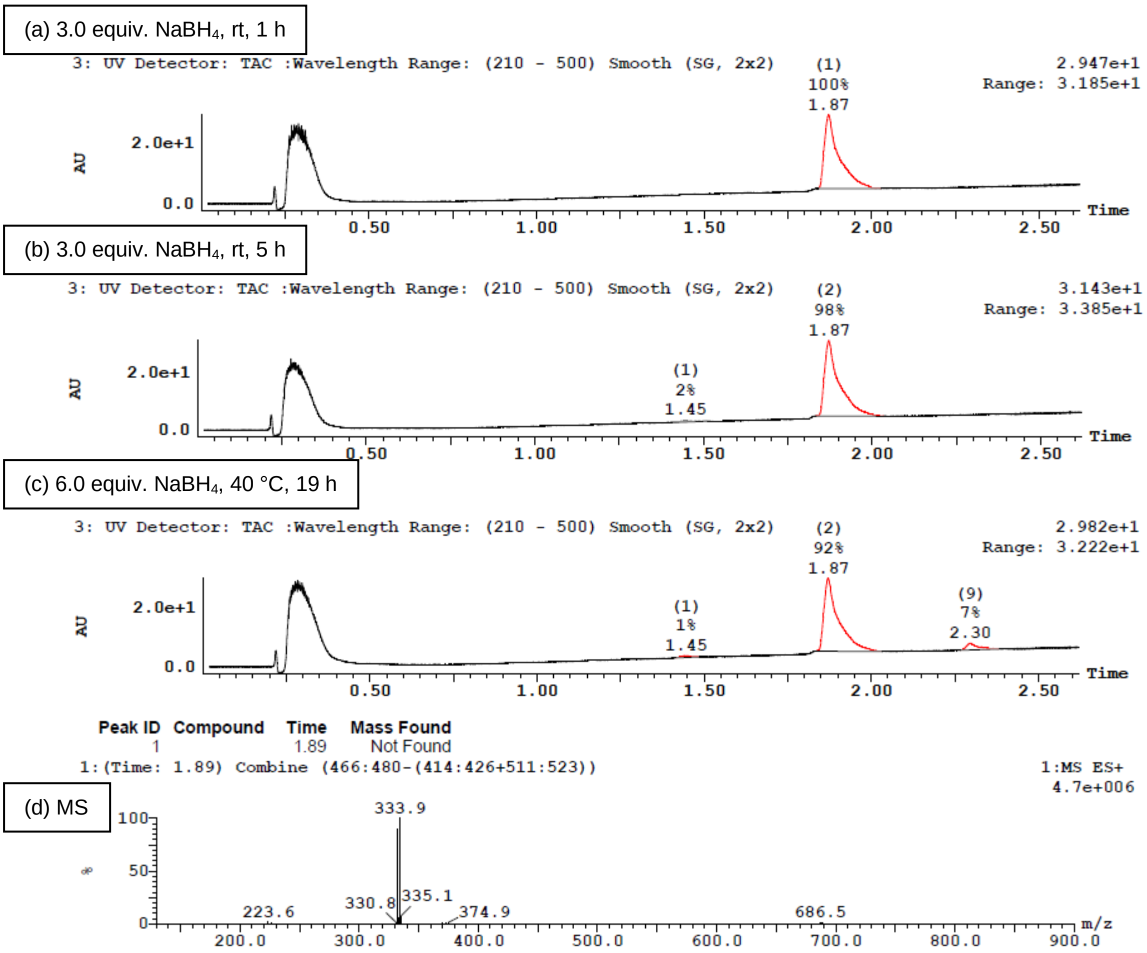

Figure S8: LC-LRMS trace of the reaction of compound **15b** and $NaBH_4$. (a) UV chromatogram after one hour at room temperature, (b) UV chromatogram after five hours at room temperature, (c) UV chromatogram after 19 hours in total and 14 hours at 40 °C, (d) MS spectrum.

LC-LRMS (ESI+) *m/z*: 332.0 found for $[M+H]^+$ for **15b**, 332.1 calcd. For $C_{17}H_{19}BrNO^+$.

Reaction with DIBALH

Procedure

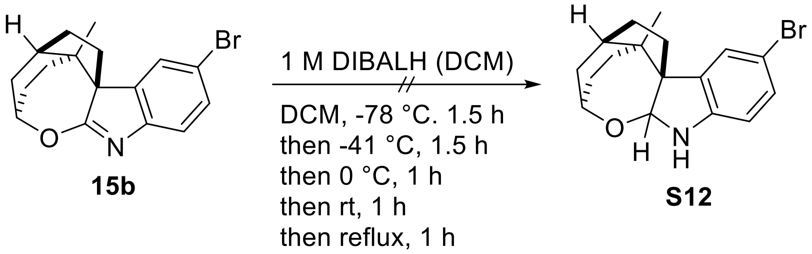

A solution of compound **15b** (5.0 mg, 15 μmol, 1.0 equiv.) in dry dichloromethane at -78 °C was added 1 M DIBALH in dichloromethane (30 μL, 30 μmol, 2.0 equiv.). The reaction mixture was stirred at -78 °C for 1.5 hour, then at -41 °C for 1.5 hour, then at 0 °C for one hour, then at room temperature for one hour, and then at reflux for one hour. The reaction was monitored by LC-LRMS.

LC-LRMS data

(a) 1 h, -78 °C

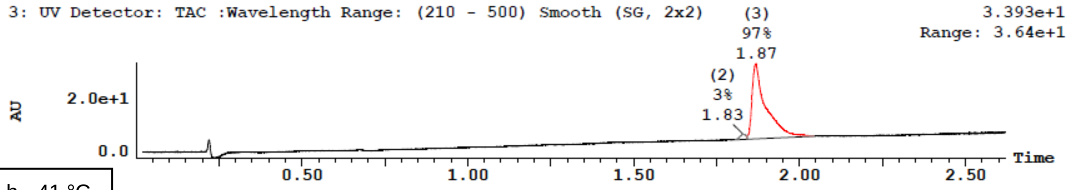

(b) 3 h, -41 °C

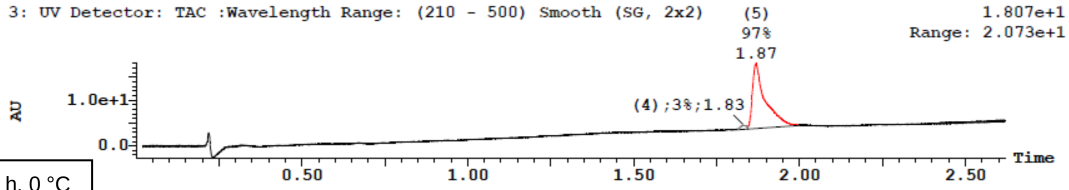

(c) 4 h, 0 °C

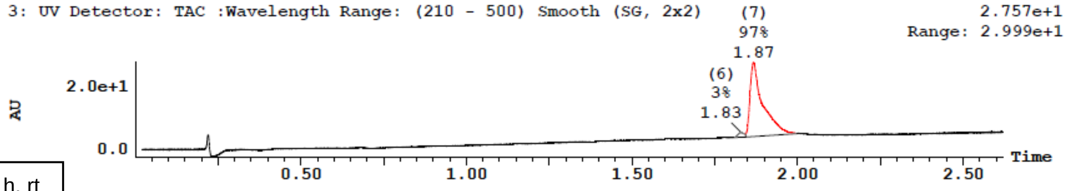

(d) 5 h, rt

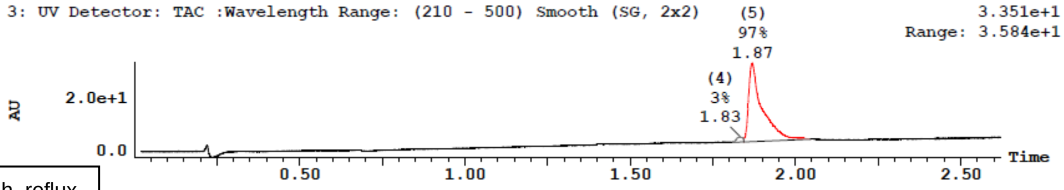

(e) 6 h, reflux

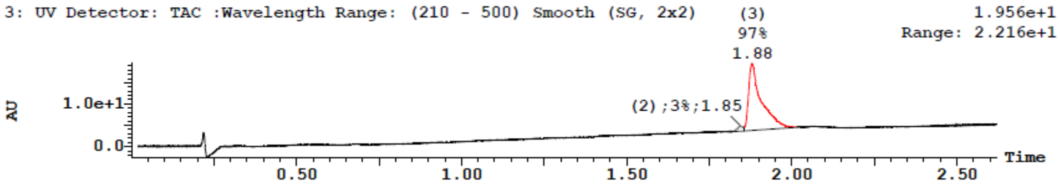

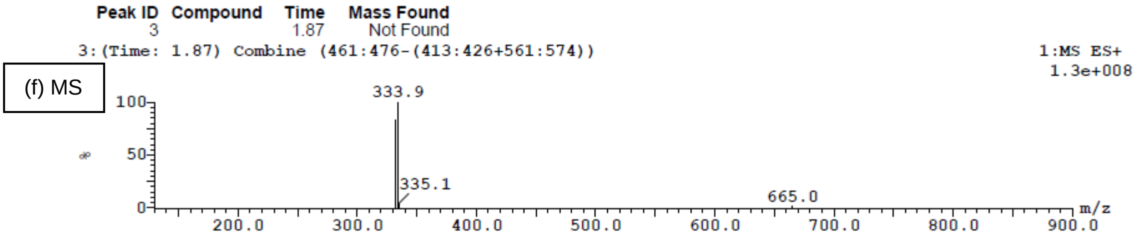

Figure S9: LC-LRMS trace of the reaction of compound **15b** and DIBALH. (a) UV chromatogram after one hour at -78 °C, (b) UV chromatogram after three hours in total and 1.5 hours at -41 °C, (c) UV chromatogram after four hours in total and one hour at 0 °C, (d) UV chromatogram after five hours in total and one hour at room temperature, (e) UV chromatogram after six hours in total and one hour at reflux, (f) MS spectrum.

LC-LRMS (ESI+) *m/z*: 332.0 found for $[M+H]^+$ for **15b**, 332.1 calcd. For $C_{17}H_{19}BrNO^+$.

Conclusion

Treating the spirooxepinoindole **15b** with $NaBH_4$ at 40 °C or DIBAL in refluxing dichloromethane showed no sign of conversion of the spirooxepinoindole (Figure S8 and Figure S9) which confirms the stability of this functionality.

Final compound collection

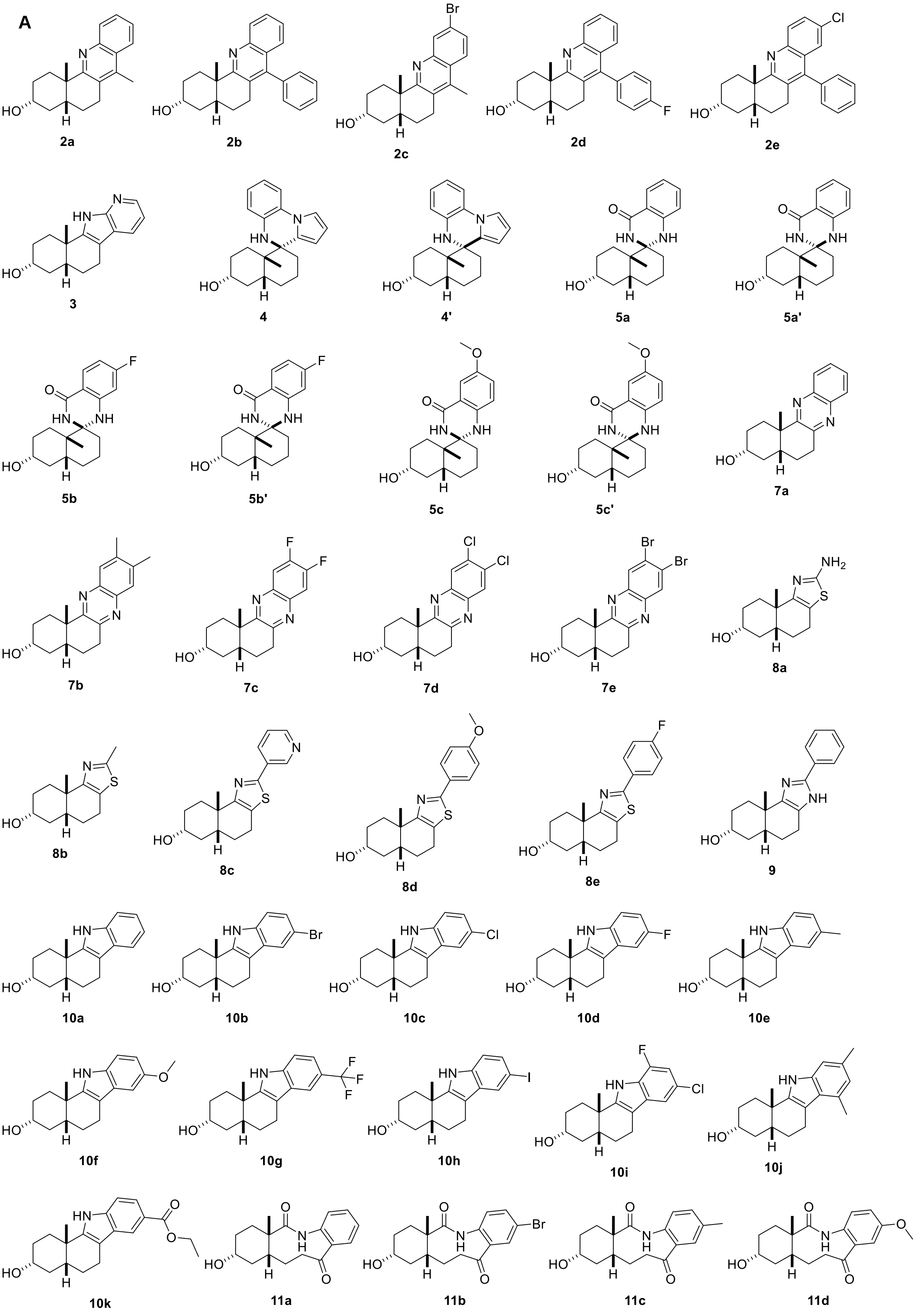

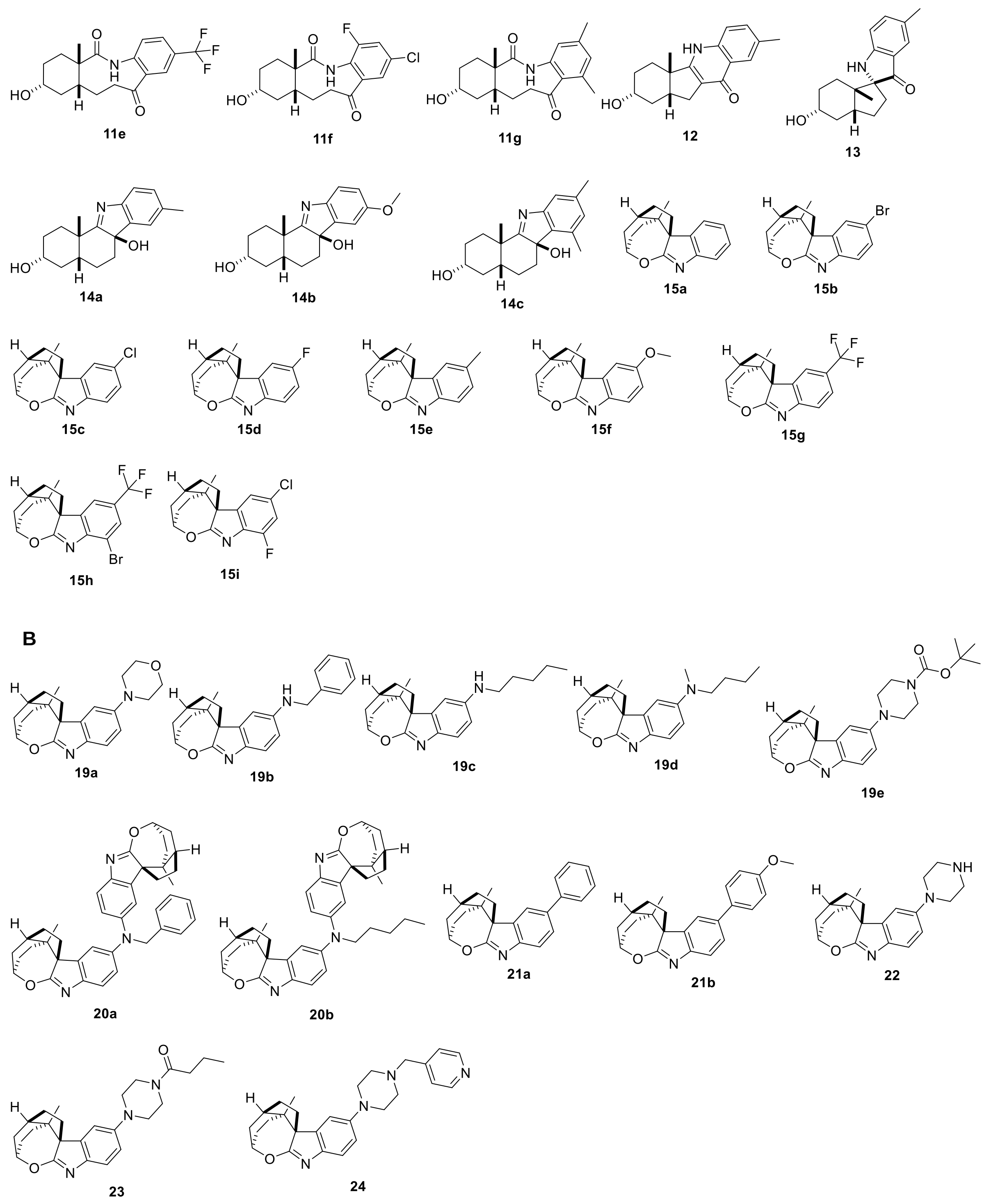

Figure S10: Final compound collection. **A)** Initial compound collection. **B)** SAR compound collection.

Graphical summary of SAR study

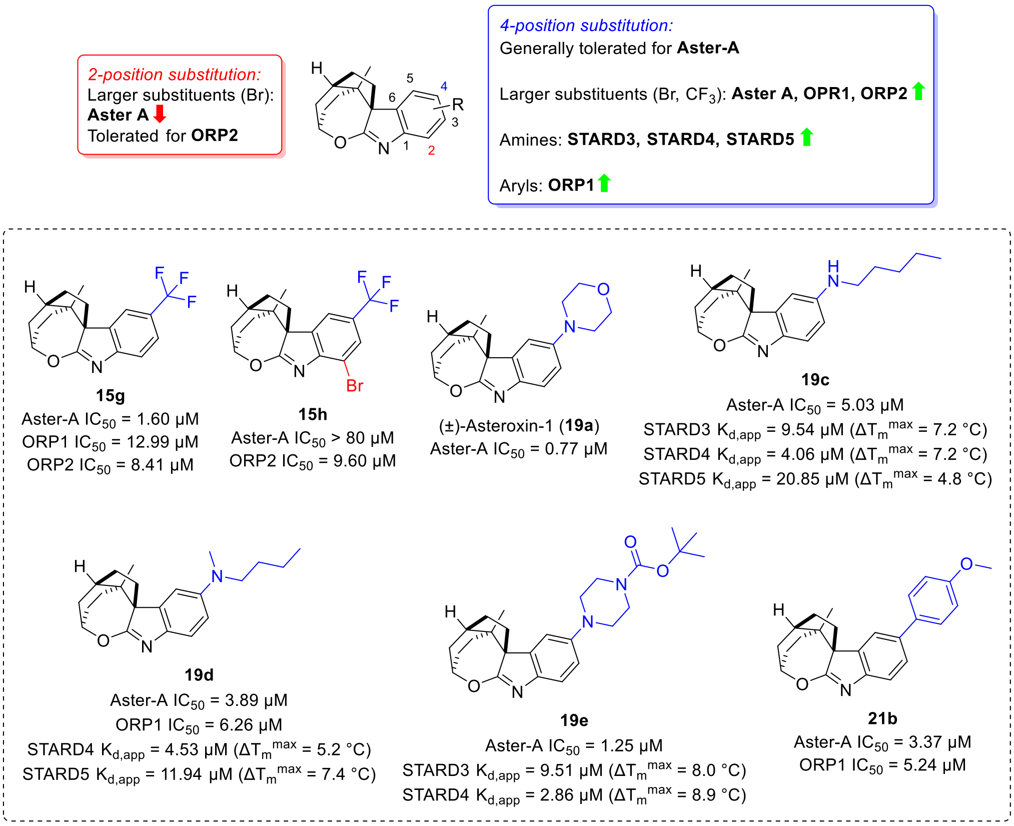

Figure S11: Graphical summary of the SAR study showing how specific substituent patterns influence potency selectivity across the STP panel using some of the most active compounds as examples.

Effect of spirooxepinoindoles on intrinsic protein fluorescence

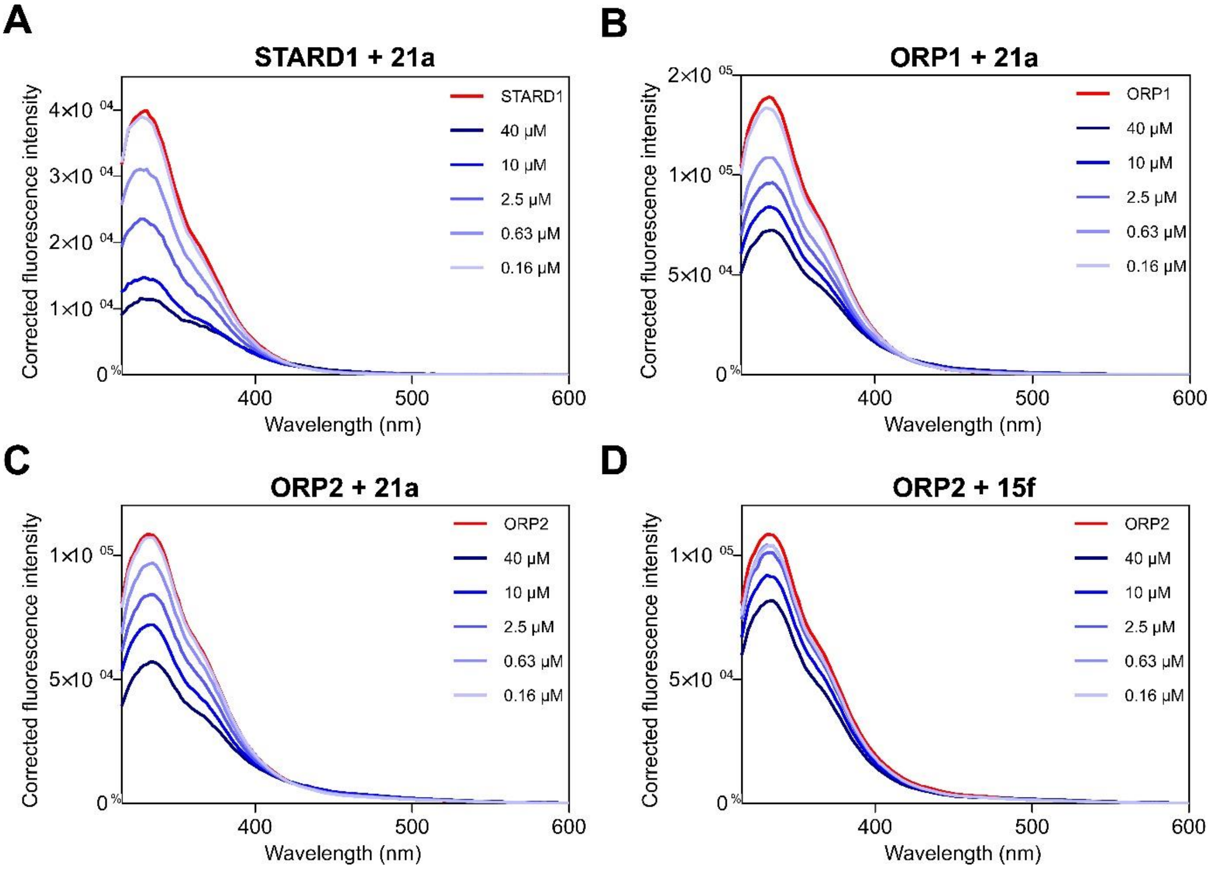

Figure S12: Spirooxepinoindoles **21a** and **15f** affect intrinsic protein fluorescence in dose-dependent fashion: Respective proteins (1 µM) were incubated with the compounds at different doses. Proteins were excited at 280 ± 10 nm and fluorescence spectra were measured from 315 ± 20 nm to 600 ± 20 nm with a step size of 2 nm. **A)** STARD1 and **21a**. **B)** ORP1 and **21a**. **C)** ORP2 and **21a**. **D)** ORP2 and **15f**.

Synthesis of asteroxin-1 enantiomers

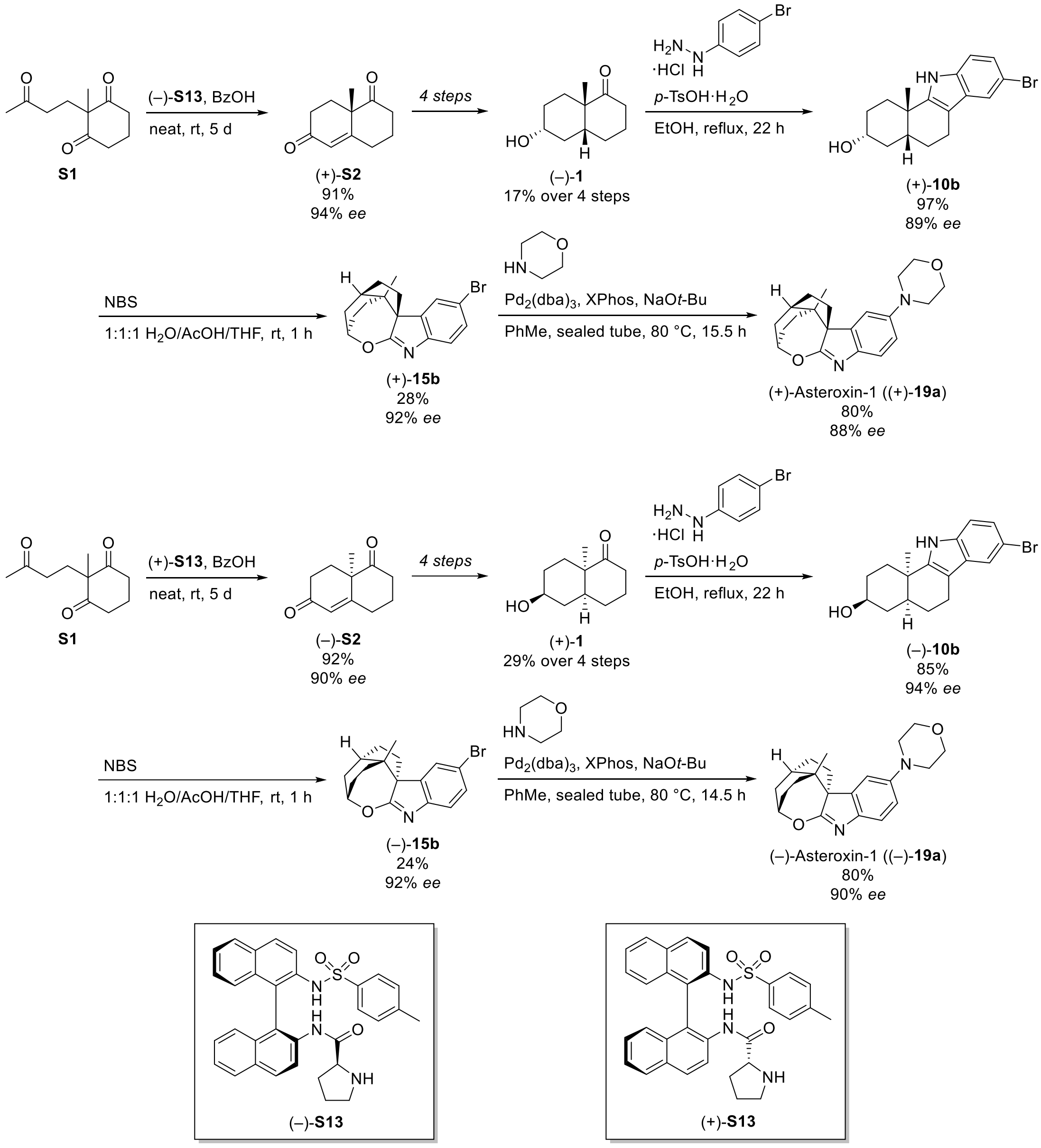

Scheme S2: Enantioselective synthesis of (+)-asteroxin-1 ((+)-**19a**) and (–)-asteroxin-1 ((–)-**19a**). Please see experimental procedures for further details.

Determination of Aster-A melting temperature in Jurkat cells

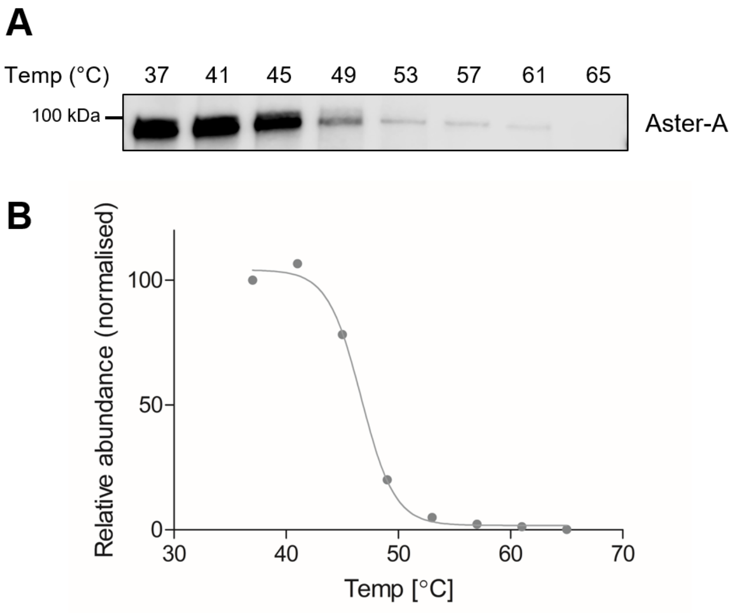

Figure S13: In-cell thermal stability assay to determine the melting temperature of Aster-A in Jurkat cells. **A)** Western blot of soluble Aster-A after heating intact cells to different temperatures and removing the insoluble fraction by centrifugation (please see Figure S14A for uncropped western blot). **B)** Quantification of A) to determine the melting curve for Aster-A based on relative abundance (n=1).

Uncropped western blots

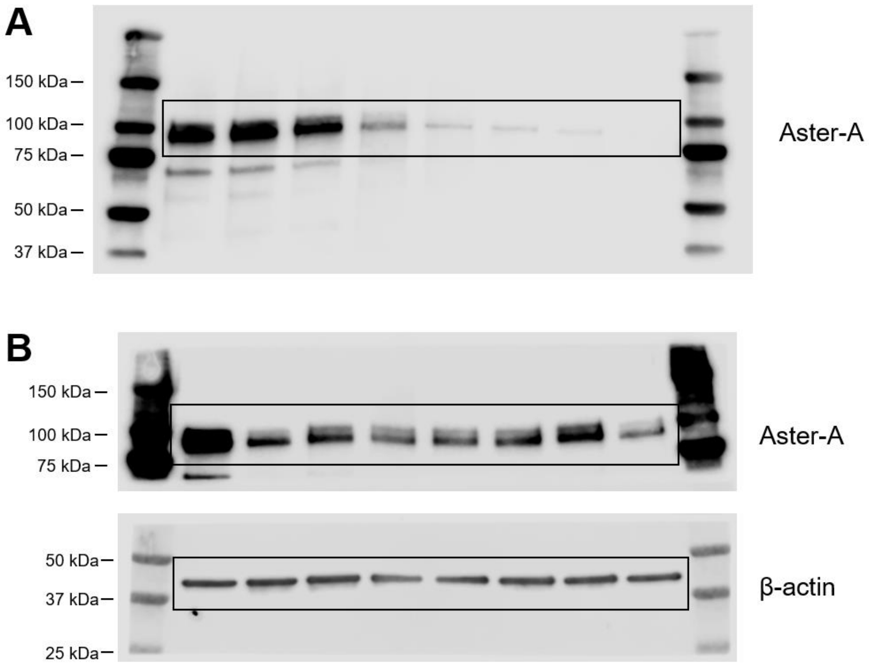

Figure S14: Uncropped western blots. **A)** Western blot from in-cell thermal stability assay to determine the melting temperature of Aster-A in Jurkat cells (from Figure S13A). **B)** Western blot from in-cell isothermal shift assay to measure compound-induced stability of Aster-A in Jurkat cells (from Figure 4A).

General directions

Biology

Protein expression constructs

Human ASTER domains of Aster-A(359-547), -B(364-552) and -C(318-504) were subcloned into a pGEX-6p-2rbs vector, thus introducing the cloning artifact 'GPLGS'.[3] The pGEX-6P-1-GST-OSBP(377-807), pET24b(+)-ORP1(534-950) and pET24b(+)-ORP2(49-480) plasmids were purchased from Genscript. The pET22b_His6_STARD1(66-284) and pET22b_His6_STARD3(216-444) plasmids were a gift from James H. Hurley (University of California).[4] The pHIS_2His6_Thrombin_STARD4(2-205;C75S) plasmid was a gift from Young Jun Im (Chonnam National University).[5] STARD5A was a gift from Nicola Burgess-Brown (Addgene plasmid #42392; http://n2t.net/addgene:42392; RRID:Addgene_42392).

Protein expression and purification

The ASTER domains of human Aster-A(359-547), -B(364-552) and -C(318-504) in pGEX-6p-2rps vectors including an N-terminal PreScission-cleavable glutathione *S*-transferase (GST)-tag were expressed in *Escherichia coli* (*E. coli*) OverExpress C41 in Terrific Broth (TB) medium for 16 h at 18 °C after the induction with 0.1 mM isopropyl β-D-1-thiogalactopyranoside (IPTG). Cells were harvested at 3500 × g for 15 minutes and lysed by sonication in buffer containing 50 mM HEPES pH 7.5, 300 mM NaCl, 10 %v/v glycerol, 5 mM dithiothreitol (DTT), 0.1 %v/v Triton X-100 and protease inhibitor mix HP plus (Serva). The lysate was purified by affinity chromatography on a GSTrap FF column (Cytiva) using an ÄKTA Start (Cytiva) in buffer containing 50 mM HEPES pH 7.5, 300 mM NaCl, 10 %v/v glycerol, 5 mM DTT and 0.01 %v/v Triton X-100. The GST-tag was cleaved overnight on the column at 4 °C. The Aster sterol binding domains were further purified by size-exclusion chromatography (SEC) on a HiLoad 16/600 Superdex 75 pg (Cytiva) in buffer containing 20 mM HEPES pH 7.5, 300 mM NaCl, 10 %v/v glycerol and 2 mM DTT.

The START domains of human STARD1(66-284), STARD3(216-444), STARD4(2-205; C75S) and STARD5(6-213) harboring an N-terminal His_6-Tag were expressed in *E. coli* BL21(DE3) in Luria-Bertani Broth (LB) medium for approximately 16 h at 18 °C after induction with 0.15 mM IPTG. Cells were harvested at 3500 × g for 15 minutes and lysed by sonication in buffer containing 50 mM HEPES pH 7.5, 150 mM NaCl, 5 %v/v glycerol, 5 mM DTT, 0.1 %v/v Triton X-100 and ethylenediaminetetraacetic acid (EDTA)-free protease inhibitor cocktail (Sigma-Aldrich). The cleared lysate was purified by affinity chromatography on a Ni-NTA Superflow Cartridge (Qiagen) using an ÄKTA Start (Cytiva) in buffer containing 50 mM HEPES pH 7.5, 150 mM NaCl, 5 %v/v glycerol, 5 mM DTT. START domains were eluted by using elution buffer containing 50 mM HEPES pH 7.5, 150 mM NaCl, 5 %v/v glycerol, 5 mM DTT and 500 mM imidazole. Proteins were further purified by SEC on a HiLoad 16/600 Superdex 75 pg (Cytiva) in buffer containing 20 mM HEPES pH 7.5, 150 mM NaCl, 5 %v/v glycerol and 2 mM DTT.

The ORP domain of human OSBP(377-807) in the pGEX-6p-1 vector with an N-terminal PreScission-cleavable GST-tag was expressed in *E. coli* OverExpress C41 in LB medium for 16 h at 18 °C after induction with 0.1 mM IPTG. Cells were harvested at 3500 × g for 15 minutes and lysed by sonication in buffer containing 20 mM HEPES pH 7.5, 300 mM NaCl, 10 %v/v glycerol, 5 mM DTT, 0.1 %v/v Triton X-100 and EDTA-free protease inhibitor cocktail (Sigma-Aldrich). The cleared lysate was purified by affinity chromatography on a GSTrap HF column (Cytiva) using an ÄKTA Start (Cytiva) in buffer containing 20 mM HEPES pH 7.5, 300 mM NaCl, 10 %v/v glycerol, 5 mM DTT. OSBP(377-807) was eluted by using elution buffer containing 20 mM HEPES pH 7.5, 300 mM NaCl, 10 %v/v glycerol, 5 mM DTT and 10 mM reduced glutathione. Proteins were further purified by SEC on a HiLoad 16/600 Superdex 75 pg (Cytiva) using an ÄKTA Explorer (Cytiva) in buffer containing 20 mM HEPES pH 7.5, 150 mM NaCl, 10 %v/v glycerol and 2 mM DTT.

The ORP domains of human ORP1(534-950) in pET24b(+) and ORP2(49-480) in the pET24b(+) vector including an N-terminal His_6-Tag were expressed in *E. coli* BL21(DE3) in TB medium for approximately 16 h at 18 °C after induction with 0.1 mM IPTG. Cells were collected at 3500 × g for 15 minutes and lysed by sonification in buffer containing 10 mM Tris-HCl pH 8, 300 mM NaCl, 5 % glycerol, 2 mM DTT, 0.1 % Triton X-100 and EDTA-free protease inhibitor cocktail (Sigma-Aldrich). The cleared lysate was purified by affinity chromatography on a Ni-NTA Superflow Cartridge (Qiagen) using an ÄKTA Start (Cytiva) in buffer containing 10 mM Tris-HCl, pH 8, 300 mM NaCl, 5 % glycerol, 2 mM DTT. ORP domains were eluted using elution buffer containing 10 mM Tris-HCl, pH 8, 300 mM NaCl, 5 % glycerol, 500 mM imidazole, 2 mM DTT. The proteins were further purified by SEC on a HiLoad 16/600

Superdex 75 pg (Cytiva) using an ÄKTA Explorer (Cytiva) in buffer containing 10 mM Tris-HCl, pH 8, 150 mM NaCl, 5 % glycerol and 2 mM DTT.

Fluorescence intensity and polarisation

Fluorescence intensity and polarisation (FP) experiments were performed in a buffer containing 20 mM HEPES pH 7.5, 300 mM NaCl, 0.01 %v/v Tween-20, 0.5% glycerol and 2 mM DTT in a final volume of 30 µl in black, flat-bottom, non-binding 384-well plates (Corning). All compounds were dispensed using the LabCyte Echo 550 Liquid handler. For K_d measurements fluorophore was incubated with desired concentrations of protein. For competition experiments, 20 nM 22-NBD-cholesterol or 80 nM 25-NBD-cholesterol was mixed with protein and incubated with desired concentrations of screening compounds (Table S4) The fluorescence polarisation signal was measured using a Spark Cyto multimode microplate reader (Tecan) with filters set at 485 ± 20 nm for excitation and at 535 ± 20 nm for emission. Measured mP values were normalised setting 0% inhibition as the FP signal from the protein + fluorophore control well and 100% as the FP signal from the fluorophore only control well. Curves were fitted to the normalised data via non-linear regression to allow the determination of IC_{50} values.

To determine the effect of compounds on protein fluorescence, the protein concentration was kept constant at 1 µM. Compounds were dispensed in 2-fold dilution across 10 points, starting with 80 µM. The protein solution was then added to the plate and incubated for 20 minutes after centrifugation. The fluorescence intensity spectrum was measured using a Spark Cyto multimode microplate reader (Tecan) with monochromator set at 280 ± 10 nm for excitation of the protein. The emission was measured from 315 ± 20 nm to 600 ± 20 nm with a step size of 2 nm. The resulting fluorescence spectra were normalised against the appropriate compound only controls. All data was analysed using GraphPad Prism 5.

Table S4: Conditions for FP experiments with the relevant STPs.

Protein	Protein conc. for selectivity measurements	Protein conc. for dose response measurements	Incubation temp.	Incubation time (Protein/NBD cholesterol)	Incubation time (Protein-NBD-cholesterol/compound)
**Aster-A**	1 µM	0.5 µM	4 °C	20 min	20 min
**Aster-B**	1 µM	0.5 µM	4 °C	20 min	20 min
**Aster-C**	1 µM	0.5 µM	4 °C	20 min	20 min
**STARD1**	1 µM	1 µM	4 °C	30 min	20 min
**OSBP**	1 µM	0.25 µM	4 °C	60 min	20 min
**ORP1**	1 µM	0.2 µM	4 °C	60 min	20 min
**ORP2**	1 µM	0.7 µM	4 °C	60 min	20 min

Differential scanning fluorimetry

Differential scanning fluorimetry (DSF) experiments were performed in a buffer composed of 20 mM HEPES pH 7.5, 300 mM NaCl, and 2 mM DTT in Milli-Q water. Stock solutions of STARD3 and STARD5 were made at a concentration of 5 µM, and STARD4 at 2.5 µM in the HEPES Buffer. A LabCyte Echo 550 Liquid Handler was used to transfer the required amount of DMSO dissolved ligand into the 384-well plate (LightCycler® 480 Multiwell Plate 384, white). Final concentrations of ligands in a single concentration high-throughput screening are 12.5 µM. For dose response measurements, a 2-fold dilution over 8 points was made starting at concentrations of either 100 µM (STARD3 and 5) or 50 µM (STARD4). This was lowered for compounds clearly showing solubility issues and became compound specific. After compound addition, 10 µL of protein solutions was manually pipetted to each well using an electronic 12-channel pipette. The plate was then briefly centrifuged before subsequently adding 20 nL of 5000x SYPRO orange (Sigma-Aldrich), with the Echo liquid handler, for a final concentration of 10x SYPRO orange. The fluorescence intensity was measured in a Roche LightCycler 480 II with an initial incubation at room temperature for 10 minutes before ramping the temperature from 30 °C, by steps of 0.2 °C, up to 90 °C with incubation for 5 seconds at each step. Melting temperatures were calculated with the Roche TSA analysis program.

Sterol transfer assay

Preparation of vesicles

1,2-dioleoyl-sn-glycero-3-phosphocholine (DOPC, Avanti Polar Lipids, 850375C) was prepared in chloroform (10 mg/mL); 23-(dipyrrometheneboron difluoride)-24-norcholesterol (TopFluor® Cholesterol, Avanti Polar Lipids, 810255) and *N*-(lissamine rhodamine B sulfonyl)-1,2-dihexadecanoyl-

sn-glycero-3-phosphoethanolamine (triethylammonium salt) (Rh-DHPE, Invitrogen, L1392) were prepared in methanol (100 µM). The acceptor liposomes (LA) consist of DOPC only while the donor liposomes (LD) consist of a mixture of DOPC:TF-Chol:Rh-DHPE (99:0.5:0.5). The solvent was evaporated under a stream of nitrogen, followed by drying under vacuum overnight. The lipid films were hydrated to a final concentration of 60 µM using buffer containing 20 mM HEPES pH 7.5, 300 mM NaCl and 2 mM DTT. To fully dissolve the lipid films the solutions were vortexed and sonicated for 5 minutes in a 40 °C water bath, followed by five freeze and thaw cycles in liquid nitrogen. Extrusion through a polycarbonate membrane (21 times, 0.1 µM pore size, Avanti Polar Lipids) at 40 °C yielded homogenous unilamellar vesicles, which were kept on ice and used on the same day.

Microplate-based cholesterol transfer assay

Aster-A with a final concentration of 125 nM was pre-incubated with either dimethyl sulfoxide or compound from a 10 mM stock solution in dimethyl sulfoxide with a final concentration of 10 µM at room temperature for 30 minutes in buffer (20 mM HEPES pH 7.5, 300 mM NaCl, 2 mM DTT). The measurements were carried out in non-binding clear-bottom 96-well plates (Greiner Bio-One, 655906). Equal amounts of LA and LD were added to the wells (16 µM). After 2.5 min of Förster resonance energy transfer (FRET) signal measurement Aster-A pre-incubated with dimethyl sulfoxide or compound was added and rapidly mixed using a pipette. Control wells contained the LA/LD mixture only. FRET signal was measured using a Tecan Spark Cyto plate reader, with readings from the bottom of the wells every 10 seconds for 15 minutes. The excitation monochromator was set at 488 ± 20 nm and the emission monochromator at 590 ± 20 nm.

Cell culture

Jurkat cells were cultured in RPMI 1640 GlutaMAX supplemented with 10% fetal bovine serum and 1% penicillin-streptomycin at 37 °C, 5% CO_2.

In-cell thermal stability assay

To a 15 mL falcon tube were added 3×10^5 Jurkat cells in media and centrifuged at 300 × g for three minutes. The supernatant was aspirated, and the cells were resuspended in 4 mL media containing 0.1% DMSO. The cells were gently vortexed to avoid pelleting at 30 minutes intervals. After 1 h incubation at 37 °C and 5% CO_2, the cells were washed twice with PBS and collected in 0.9 mL PBS and divided into eight aliquots, each 100 µL in PCR tubes. The aliquots were individually heated at different temperatures (Doppio 2 × 48 well Thermal cycler, VWR). After the heat treatment, 10 µL of PBS containing 4.4 %v/v NP-40 was added to each sample and cells were lysed by freeze-thaw four times. The cell lysates were transferred to 1.5 mL Eppendorf tubes and centrifuged (Micro Star 30, VWR) at 25000 × g, 4 °C for 25 minutes. The cleared supernatants were collected and further analysed by western blotting.

In-cell isothermal shift assay

To eight 15 mL falcon tubes were added 3×10^5 Jurkat cells in 1 mL of media per tube and incubated with indicated test compounds for one hour at 37 °C and 5% CO_2. The cells were gently vortexed to avoid pelleting at 30 minutes intervals. After one hour incubation at 37 °C and 5% CO_2, the cells were centrifuged at 300 × g for three minutes, washed once with PBS and centrifuged again at 300 × g for three minutes. The cells were then resuspended in 50 µL of PBS in PCR tubes. The samples were then heated at the given temperature (Eppendorf Mastercycler ep Gradient S). After the heat treatment, 5 µL of PBS containing 4.4 %v/v NP-40 were added to each sample and cells lysed by freeze-thaw four times. Finally, the cell lysates were completely transferred to 1.5 mL Eppendorf tubes and centrifuged (Micro Star 30, VMR) at 25000 × g, 4 °C for 25 minutes. The cleared supernatants were collected and further analysed by western blotting.

Sodium dodecyl sulfate-polyacrylamide gel electrophoresis and western blotting

Sodium dodecyl sulfate-polyacrylamide gel electrophoresis (SDS-PAGE) experiments were carried out using 4-15% precast polyacrylamide gels (Bio-Rad) and run at a constant voltage of 100 V for ten minutes followed by 120 V for one hour. Semidry transfer onto a 0.2 µm nitrocellulose membrane was performed using Bio-Rad Trans-Blot Turbo Transfer System at 1.3 A for seven minutes. For chemiluminescent detection, membranes were blocked in 5% milk in TBST (tris-buffered saline with Tween; 137 mM NaCl, 19 mM Tris-base, 2.7 mM KCl and 0.1 %v/v Tween-20, blocking buffer) for one houre at room temperature. The membrane was incubated with the primary antibody in blocking buffer

overnight at 4 °C. After washing with TBST (3 × 15 minutes), the membrane was incubated with the secondary antibody in blocking buffer for one hour at room temperature. The antibodies used for immunoblotting can be found in Table S5. Signals were visualised using the SuperSignal West Pico Chemiluminescent Substrate (Thermo Fisher, catalogue no. 34579) or the SuperSignal West Femto Maximum Sensitivity Substrate (Thermo Fisher, catalogue no. 34094) on a Li-COR Odyssey Fc. Western blot quantification was performed using LI-COR Image Studio Lite. Visualisation of graphs was performed using GraphPad Prism 10.

Table S5: Antibodies used for immunoblotting.

**Antibody**	**Host**	**Clonality**	**Vendor**	**Cat. No.**	**Dilution**
GRAMD1A (Aster-A)	Rabbit	Polyclonal	Novus Biologicals	NBP2-32148	1:1000
Β-actin (AC-15)	Mouse	Monoclonal	Invitrogen	AM4302	1:5000
**Antibody**	**Host**	**Conjugation**	**Vendor**	**Cat. No.**	**Dilution**
Anti-rabbit	Goat	HRP	Invitrogen	31460	1:10000
Anti-mouse	Horse	HRP	VWR	VECTPI-2000	1:10000

Molecular modelling

Software: Maestro version 13.3.121, MMshare Version 5.9.121, Release 2022-3, Platform Windows-x64.

Crystal structure PDB source files were obtained from the Protein Data Bank (PDB ID: 6gqf). Generation of the homology model of human Aster-A based on the crystal structure of murin Aster-A (pdb: 6gqf) was performed using the Maestro default workflow. Protein preparation was carried out using the default workflow with a few minor adjustments. Namely, in the preprocess workflow, create disulphide bonds, fill in missing loops (using Prime) were selected, and setting the variation of het states (using Epik) to pH range 7.5 ± 0.5. For the H-bond assignments workflow; H-bonds were assigned using PROPKA at pH 7.5. In the final Minimise and Delete waters workflow; a restrained minimisation was performed with a convergence of 0.3 Å to heavy atoms. If there existed waters in the binding site, the preparation was optimised by running a validation test on the binding of the cognate ligands. Ligand preparation was carried out on all possible stereoisomeric forms of the ligand, which is desalted, and ionised at pH 7.5 ± 0.5 using Epik. Force field used is OPLS4.

For initial screenings of crystal structures with ligands, receptor grid generation was carried out centroid on the co-crystalised ligand. High throughput ligand docking was carried out using Glide. Utilising standard precision (SP), with flexible ligands. The settings allowed for sampling of nitrogen inversions and ring conformations. Epik state penalties were applied to docking scores. Three poses for each ligand were generated, allowing for more precise accounts of ligand-protein viability. Post-docking minimisation and strain correction were also applied to the scoring of each ligand. Pose views were sampled in correlation to the Docking scores. For Induced Fit workflows, the binding domain is centroid on the resident ligand. Ligands are free to sample variations in ring conformation. In glide docking the protein preparation constrained refinement is selected for with maximally 20 poses to be generated. Prime refinement is within 5 Å of ligand poses and the Glide redocking is at standard precision. Pose analysis was performed on all examples provided from the simulation. Key considerations were on the retention of any significant position, residue interactions and orientations that were observed as median averages.

Chemistry

All reactions were run in oven-dried glassware (round-bottom flask unless otherwise specified) equipped with a magnetic stir bar under ambient light and under a nitrogen atmosphere (nitrogen flow) achieved by a Schlenk line unless otherwise specified. Argon atmosphere was achieved using a balloon with argon gas unless otherwise specified. Ambient air atmosphere was achieved using an open reaction vessel. Reactions were monitored by thin-layer chromatography (TLC) and/or reversed-phase ultra-performance liquid chromatography-low resolution mass spectrometry (RP-UPLC-LRMS). Reaction heating was performed with an oil-bath unless otherwise specified. Reflux conditions was achieved by setting the oil bath temperature approximately 10 °C above the boiling point of the solvent. Specific reaction temperatures refer to the temperature of the oil bath. Reaction cooling was achieved with cooling baths (0 °C: water/ice, -41 °C: acetonitrile/dry ice, -78 °C: acetone/dry ice). Commercially available reagents were purified according to standard procedures or were used as received from Sigma Aldrich, Alfa Aesar, Acros Organics, Combi-Blocks, Fisher Scientific, Strem, TCI, and Merck unless otherwise specified. All solvents used were of high-performance liquid chromatography (HPLC) quality and dry solvents (acetonitrile, dichloromethane, diethyl ether, dimethylformamide, tetrahydrofuran, toluene, and dimethyl sulfoxide) were obtained from a PureSolv system (Innovative Technology, Tronyx). Compounds and intermediates were stored in the freezer (-20 °C). Determination of diastereoisomeric ratio (*dr*) and amount of known side-products in isolated compounds was based on quantitative ^{1}H nuclear magnetic resonance (NMR) spectroscopy. Determination of enantiomeric ratio (*er*)/enantiomeric excess (*ee*) was based on quantitative HPLC.

Analytical TLC was conducted on Merck aluminium sheets covered with silica (C60). The plates were either visualised under ultraviolet (UV)-light or stained by dipping in a developing agent followed by heating. $KMnO_4$ [3 g in water (300 mL) along with K_2CO_3 (20 g) and 5% aqueous NaOH (5 mL)], cerium molybdate [$Ce(NH_4)_2(NO_3)_6$ (0.5g), $(NH_4)_6Mo_7O_{24}\cdot 4H_2O$ (24.0 g), and H_2SO_4 (24.0 g)], phosphomolybdic acid [phosphomolybdic acid (10 g) in ethanol (100 mL], and *p*-anisaldehyde [*p*-anisaldehyde (15 mL) in ethanol (250 mL) along with conc. H_2SO_4 (2.5 mL)] were used as developing agents. Filtration through celite was performed using Celite® 512 or 545. Flash column chromatography was performed using 1) Merck Geduran® Si 60 (40-63 µm) silica gel in the indicated solvent system and applying pressure with air using a hand-powered blower unless otherwise specified *or* 2) BÜCHI® Pure C-805 Flash Chromatography system equipped with a UV (200-400 nm) diode array detector using FlashPure EcoFlex or Select Silica cartridges of appropriate size and the indicated solvents and flow rate. Crude reaction mixtures were wet-loaded or dry-loaded on Celite® 512 or 545. After flash column chromatography, the appropriate fractions were pooled, the solvent removed under reduced pressure, and dried under high vacuum for at least overnight.

Searches for reported characterisation data using Reaxys® and SciFinder® were performed on all compounds. All biologically tested compounds were obtained in high purity (≥95%) unless otherwise stated following the purity criteria of J. Med. Chem.[6] All new compounds (no published characterisation data) were characterised by NMR spectroscopy, infrared (IR) spectroscopy, LRMS, high resolution mass spectrometry (HRMS), and melting point (mp)/decomposition point (dec pt) where appropriate (by-products were not fully characterised). Structural assignments were made when possible for new compounds using 2D COESY, HSQC, HMBC, H2BC, and NOESY spectra where appropriate. The recording of all NMR spectra was performed at 298 K unless otherwise specified. For the recording of 1D ^{1}H NMR, 1D ^{13}C NMR, 1D ^{15}N NMR, and 2D NMR spectra, a Bruker Avance III 400 spectrometer with a Bruker Ascend 400 magnet and a Prodigy CryoProbe (operating at 400 MHz for proton, 101 MHz for carbon-13, and 41 MHz nitrogen-15) was used. For the recording of 1D ^{19}F NMR spectra, a Bruker Avance III 400 spectrometer with a Bruker Ultrashield 400 PLUS magnet and a SmartProbe (operating at 377 MHz for fluorine-19) was used. For the recording of some 1D ^{1}H NMR, 1D ^{13}C NMR, 1D ^{15}N NMR, 1D ^{19}F NMR and 2D NMR spectra, and variable temperature (VT) NMR up to 348 K, a Bruker Avance III 800 spectrometer with a Bruker Ascend 800 magnet and a 5 mm TCI CryoProbe (operating at 800 MHz for proton, 201 MHz for carbon-13, 81 MHz for nitrogen-15, and 753 MHz for fluorine-19) was used. For the recording of some 1D ^{1}H NMR, 1D ^{13}C NMR, and 1D ^{15}N NMR spectra and VT-NMR above 348 K and up to 423 K, a Bruker Avance III 600 spectrometer with a Bruker Ascend 600 magnet and a 5 mm BBFO Probe (operating at 600 MHz for proton, 151 MHz for carbon-13, and 61 MHz for nitrogen-15) was used. The 1D ^{13}C NMR and 1D ^{19}F NMR spectra were ^{1}H decoupled unless otherwise specified. The chemical shifts (δ) are reported in parts per million (ppm) and the coupling constants (J) in Hz. The chemical shifts (δ) are referenced (δ = 0.0 ppm) to tetramethylsilane ($Si(CH_3)_3$) for proton and carbon-13, liquid ammonia (NH_3 (l)) for nitrogen-15, and trichlorofluoromethane ($CFCl_3$) for fluorine-19. Spectra were referenced using the residual solvent peaks of the respective solvent; DMSO-d_6 (δ = 2.50 ppm for ^{1}H NMR and δ = 39.52 ppm for ^{13}C NMR), $CDCl_3$ (δ = 7.26 ppm for ^{1}H NMR and δ = 77.16

ppm for ^{13}C NMR), CD_3OD (δ = 3.31 ppm for ^1H NMR and δ = 49.00 ppm for ^{13}C NMR. The 1D ^{15}N NMR chemical shifts were determined by correlations in the 2D ^1H-^{15}N HSQC and HMBC spectra. The following abbreviations were used to report peak multiplicities: s = singlet, d = doublet, t = triplet, q = quartet, quint = quintet, m = multiplet, br = broad, dd = doublet of doublets, dt = doublet of triblets, dq = doublet of quartets, td = triplet of doublets, tt = triplet of triplets, tq = triplet of quartets, qd = quartet of doublets, qt = quartet of triplets, ddd = doublet of doublets of doublets, dtd = doublet of triplets of doublets, tdd = triplet of doublets of doublets, dddd = doublet of doublets of doublets of doublets. The NMR spectra were processed, analysed, and represented by MestReNova® 14.1.1.

IR analysis was performed on a Bruker Alpha FT-IR spectrometer or a PerkinElmer Spectrum 100 FT-IR spectrometer. The wavenumbers ($\tilde{\upsilon}_{\max}$) are reported in cm^{-1}. The following abbreviations were used to report band appearances and intensities: s = strong, m = medium, w = weak, br = broad.

Analytical RP-UPLC-LRMS (electrospray ionisation (ESI)) analysis was performed on a S2 Waters AQUITY RP-UPLC system equipped with a diode array detector using an Thermo Accucore C18 column (pore size = 80 Å, particle size = 2.6 µm, id = 2.1 mm, l = 50 mm) operating at 50 °C and a flow rate of 1.0 mL/min. Acidic run: Eluents A1 (0.1% HCO_2H in H_2O) and B1 (0.1% HCO_2H in MeCN). Basic run: Eluents A2 (15 mM NH_4OAc in H_2O) and B2 (15 mM NH_4OAc in 9:1 MeCN/H_2O). Eluents were used in a linear gradient. Gradient (short run): 5% B to 100% B in 2.4 min and then held for 0.1 min at 100% B (total run time: 2.6 min). Gradient (long run): 5% B to 100% B in 3.0 min then held for 0.1 min at 100% B (total run time: 5.0 min). The liquid chromatography (LC) system was coupled to a single quadrupole detection (SQD) mass spectrometer. The LC-LRMS data was processed, analysed, and represented by MassLynx® V4.1.

Analytical RP-ultra-high performance liquid chromatography (UHPLC)-HRMS (ESI) analysis was performed on an Agilent Infinity 1290 RP-UHPLC system equipped with a diode array detector using an Agilent Poroshell 120 Phenyl-Hexyl column (pore size = 120 Å, particle size = 1.9 µm, id = 2.1 mm, l = 150 mm) operating at 60 °C and a flow rate of 0.35 mL/min. Eluents A (20 mM HCO_2H in H_2O) and B (20 mM HCO_2H in MeCN) were used in a linear gradient. Gradient: 10% B to 100% B in 10 min, held for 2 min at 100% B, returned to 10% B in 0.1 min, and then held for 2 min (total run time: 14.2 min). An injection volume of 1 µL was used. Mass sprectrometry (MS) detection was performed in both positive and negative detection on an Agilent 6545 QTOF MS equipped with Agilent Dual Jet Stream electrospray ion source with a drying gas temperature of 250 °C, gas flow of 8 L/min, sheath gas temperature of 300 °C and flow of 12 L/min. Capillary voltage was set to 4000 V and nozzle voltage to 500 V. Mass spectra were recorded at 10, 20 and 40 eV as centroid data for m/z = 85–1700 in MS mode and m/z = 30–1700 in MS/MS mode, with an acquisition rate of 10 spectra/s. Lock mass solution in 70:30 methanol:water was infused in the second sprayer using an extra LC pump at a flow of 15 µL/min using a 1:100 splitter. The solution contained 1 µM tributylamine and 10 µM hexakis(2,2,3,3-tetrafluoropropoxy)phosphazene as lock masses. The $[M + H]^+$ ions (m/z 186.2216 and 922.0098 respectively) of both compounds was used. The HRMS data was processed and analysed by MassHunter Qualitative Analysis® B.07.00.

Preparative RP-HPLC was performed on a Waters Alliance reversed-phase HPLC system consisting of a Waters 2545 Binary Gradient Module equipped with an xBridge BEH C18 OBD Prep Column (pore size = 130 Å, particle size = 5 µm, id = 30 mm, l = 150 mm) operating at 20 °C and a flow rate of 20 mL/min, a Waters Photodiode Array Detector (detecting at 210-600 nm), a Waters UV Fraction Manager, and a Waters 2767 Sample Manager. Eluents A1 (0.1% HCO_2H in H_2O) and B1 (0.1% HCO_2H in MeCN) were used.

Analytical chiral HPLC analysis was performed on a Waters 2695 Alliance Separations module with a Waters 2996 PhotoDiode Array detector, equipped with a ChiralPak AD-H (particle size = 5 µm, id = 4.6 mm, l = 250 mm), Lux 3u cellulose-4 (particle size = 3 µm, id = 4.6 mm, l = 250 mm), or Lux 3u amylose-2 (particle size = 3 µm, id = 4.6 mm, l = 250 mm) column. The chiral HPLC data was processed, analysed, and represented by MassLynx® V4.1.

Melting/decomposition points were obtained in an open capillary using a Stuart SMP30 melting point apparatus. Optical rotation was carried out using a PerkinElmer Polarimeter 241 using the D-line from sodium-vapor lamp, and the specific rotation ($[\alpha]_D$) are reported in deg·mL·g^{-1}·dm^{-1}. Lyophilisation was performed in a SvanVac CoolSafe freeze dryer at approximately -92 ˚C and 0.4 mbar. Microwave irradiation was carried out in a Biotage Initiator+ Microwave Synthesizer.

Single crystals for single-crystal X-ray diffraction were grown as specified in the characterisation of the individual compounds. All crystals were grown from racemic materials. The single crystals were immersed in polybutene oil and mounted on a nylon loop, which was attached to a SuperNova Dual Source CCD-diffractometer. Data were collected using Cu Kα (λ = 1.5406 Å) radiation at 120(1) K. The structures were solved as specified in the individual reports. The X-ray crystal structures were represented with Mercury® 2021.3.0 drawing atoms as thermal ellipsoid plots at 50% probability level

(hydrogen atoms drawn as fixed-sized spheres (r = 0.20 Å)) and bonds as sticks (r = 0.07 Å). Colour codes: H = white, C = grey, N = light blue, O = red, Cl = green.

Inductively coupled plasma (ICP)-MS analysis was performed on an Thermo Scientific iCAP TQ ICP-MS equipped with a Teledyne CETAC ASX-560 autosampler. Sample preparation was performed as follows. The samples (weight between 3.4 and 5.0 mg) were weighted into 18 mL quartz vials using an analytical Sartorius GENIUS ME balance. Afterwards, concentrated aqueous (67-69%) nitric acid (PlasmaPure, SCP Science; 0.5 mL) and ultrapure water (resistivity 18.2 mΩ·cm at 21.5 °C, Millipore Element apparatus; 0.1 mL) were added. The samples were digested in a microwave reaction system Multiwave 7000 (Anton Paar), using the following digestion program: ramp to T = 250 ºC for 20 min, hold at T = 250 ºC for 10 min, cool down for 30 min, starting pressure of 40 bar and maximum pressure of around 120 bar. After digestion, the samples were transferred to 15 mL disposable polypropylene tubes (Sarstedt AG & Co. KG, Germany), diluted with a solution containing 2% aqueous HNO_3 and 1% aqueous HCl (34-37% HCl, PlasmaPure, SCP Science) to a final sample volume of approximately 10 mL, exactly weighted and the density determined. Prior to ICP-MS analysis, the digested samples were further diluted two-fold with a mix of 2% aqueous HNO_3 and 1% aqueous HCl. For quality assurance, blank sample (N = 2) and the reference material ClinChek – Control Level II (Recipe Chemicals) with a certified Pd concentration of 8.37 ± 2.12 mg/kg (N = 1) were included in all analyses. For digestion of the reference material, 0.1 mL of the sample was used. The total mass concentration of Pd in the digested samples was determined by the ICP-MS (please see above). The analysis was performed in single quadrupole mode with Helium as collision gas. Determination of the Pd mass concentrations was performed based on external calibration by measuring Pd standards in the concentration range of 0.025 to 10 µg/L with internal standardisation (1 µg/L indium (In)). Calibration and internal standards were prepared from standard solutions that contained 1000 mg/L of Pd or In (PlasmaCAL, SCP Science). All standards were matrix-matched with the diluted samples (i.e., prepared in 2% aqueous HNO_3 and 1% aqueous HCl). The instrumental configuration and operating parameters for ICP-MS analysis are summarised in Table S6.

Table S6: Instrumental configuration and operating parameters for ICP-MS analysis.

Parameter	Value/type
Nebuliser type	MicroFlow PFA-ST
Spray chamber	Quartz baffled cyclonic, cooled at 3 °C
Torch, id injector	Quartz, 2.5 mm
Interface	Nickel cones with 3.5 mm insert
RF Power	1550 W
Sampling depth	8.0 mm
Plasma gas flow rate	14 L/min
Nebuliser gas flow rate	1.05 L/min
Auxiliary gas flow rate	0.80 L/min
Cell gas	None
Monitored isotope	^{108}Pd
Isotope of internal standard	^{115}In
Dwell time	0.1 s, 10 sweeps

The average mass concentration of Pd determined in the blank samples was subtracted from the Pd mass concentrations determined in the corresponding samples. The average recovery based on the certified reference material was found to be satisfactory in the range of 80 and 120%. The repeatability was satisfactory with a relative standard deviation less than 2%. The limit of detection and quantification was respectively estimated (blank standard deviation multiplied by 3 and 10) at 0.03 mg/kg and 0.1 mg/kg.

General procedures and characterisation data

Synthesis of core scaffolds

2-Methyl-2-(3-oxobutyl)cyclohexane-1,3-dione (S1)

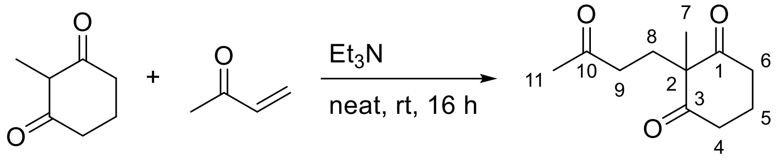

S1
99%

A mixture of 2-methyl-1,3-cyclohexanedione (20.0 g, 159 mmol, 1.0 equiv.), methyl vinyl ketone (14.2 mL, 175 mmol, 1.1 equiv.), and triethylamine (2.2 mL, 15.9 mmol, 0.1 equiv.) was stirred neat at room temperature for 16 hours. The reaction mixture was added ethyl acetate (200 mL) and activated carbon (6.0 g). The mixture was stirred at 45 °C for 30 min, cooled to room temperature, filtered by suction through a plug of silica gel eluting with ethyl acetate (4 × 150 mL), and the solvent removed under reduced pressure. The residue was dried under high vacuum to afford compound **S1** (30.7 g, 99% yield) as a yellow oil.

TLC: R_f = 0.43 (1:1 *n*-hexane/ethyl acetate), $KMnO_4$ stain.
IR (ATR) $\tilde{\upsilon}_{max}$ cm^{-1}: 2963 (w), 2944 (w), 1714 (m), 1689 (s), 1457 (w), 1423 (w), 1364 (w), 1168 (w), 1026 (w).
^{1}H NMR (400 MHz, $CDCl_3$) δ ppm: 2.76 – 2.58 (m, 4H, C4-H_2 + C6-H_2), 2.34 (t, *J* = 7.4 Hz, 2H, C9-H_2), 2.10 (s, 3H, C11-H_3), 2.08 – 1.97 (m, 3H, C5-H + C8-H_2), 1.95 – 1.84 (m, 1H, C5-H), 1.23 (s, 3H, C7-H_3).
^{13}C NMR (101 MHz, $CDCl_3$) δ ppm: 210.2 (C1 + C3), 207.7 (C10), 64.5 (C2), 38.5 (C9), 37.9 (C4 + C6), 30.1 (C11), 29.7 (C8), 20.2 (C7), 17.7 (C5).
NMR data are in accordance with literature values.[7]
LRMS (ESI+) *m/z*: 196.96 found for $[M+H]^+$, 197.12 calcd. for $C_{11}H_{17}O_3^+$.

8a-Methyl-3,4,8,8a-tetrahydronaphthalene-1,6(2*H*,7*H*)-dione (S2)

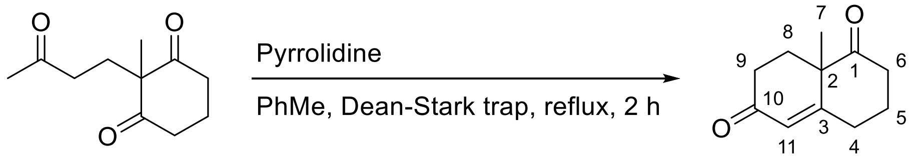

S1 **S2**
94%

To a solution of compound **S1** (14.1 g, 72.0 mmol, 1.0 equiv.) in dry toluene (40 mL) at room temperature was added pyrrolidine (0.60 mL, 7.2 mmol, 0.1 equiv.). The reaction mixture was stirred at reflux with a Dean-Stark trap for two hours. The reaction mixture was cooled to room temperature, concentrated under reduced pressure, and added ethyl acetate (100 mL) and activated carbon (3.0 g). The mixture was stirred at room temperature for 13 hours, added *n*-hexane (100 mL), filtered by suction through a plug of silica gel eluting with 1:1 *n*-hexane/ethyl acetate (7 × 100 mL), and the solvent removed under reduced pressure. The residue was dried under high vacuum to afford compound **S2** (12.0 g, 94% yield) as a reddish-brown oil that solidified to a reddish-brown glassy solid upon storage in the freezer (-20 °C).

TLC: R_f = 0.37 (1:1 *n*-hexane/ethyl acetate), UV.
mp = 47.0 – 50.0 °C. Melting point is in accordance with literature values (mp = 49 – 50 °C).[8]
IR (ATR) $\tilde{\upsilon}_{max}$ cm^{-1}: 3026 (w), 2953 (m), 2872 (w), 1709 (s), 1664 (s), 1618 (m), 1460 (w), 1447 (w), 1421 (w), 1374 (w), 1350 (w), 1325 (w), 1270 (w), 1237 (m), 1181 (w), 1155 (w), 1014 (w), 939 (w), 829 (w).
^{1}H NMR (400 MHz, $CDCl_3$) δ ppm: 5.85 (d, *J* = 1.9 Hz, 1H, C11-H), 2.77 – 2.65 (m, 2H, C4-H + C6-H), 2.54 – 2.41 (m, 4H, C4-H + C6-H + C9-H_2), 2.19 – 2.07 (m, 3H, C5-H + C8-H_2), 1.70 (qt, *J* = 13.3, 4.4 Hz, 1H, C5-H), 1.44 (s, 3H, C7-H_3).

^{13}C NMR (101 MHz, $CDCl_3$) δ ppm: 211.2 (C1), 198.4 (C10), 165.9 (C3), 126.0 (C11), 50.7 (C2), 37.8 (C6), 33.7 (C9), 31.9 (C4), 29.8 (C8), 23.4 (C7), 23.0 (C5).
NMR data are in accordance with literature values.[7]
LC-LRMS (ESI+) *m/z*: 178.99 found for $[M+H]^+$, 179.11 calcd. for $C_{11}H_{15}O_2^+$, R_t = 0.89 min.

8a-Methyl-3,4,8,8a-tetrahydro-2*H*-spiro[naphthalene-1,2'-[1,3]dioxolan]-6(7*H*)-one (S3)

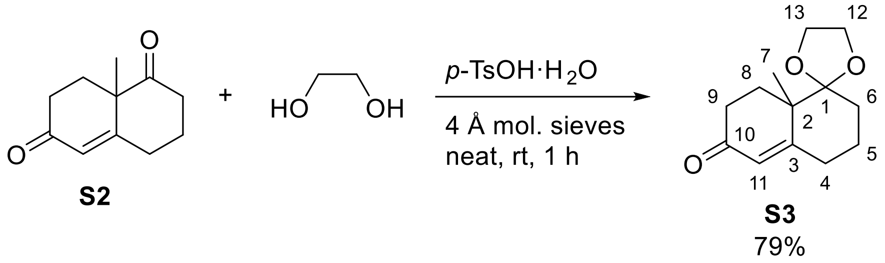

A mixture of compound **S2** (12.0 g, 67.5 mmol, 1.0 equiv.), activated 4 Å molecular sieves (12.0 g, 100 wt% of **S2**), *p*-toluenesulfonic acid monohydrate (12.8 g, 67.6 mmol, 1.0 equiv.), and dry ethylene glycol (85.0 mL, 1.52 mol, 22.5 equiv.) was stirred neat at room temperature for one hour. The reaction mixture was poured carefully into a mixture of 2:1 ice-water/saturated aqueous $NaHCO_3$ (200 mL) and extracted with ethyl acetate (3 × 200 mL). The combined organic phases were washed with water (130 mL) and brine (130 mL). The combined organic phases were dried over anhydrous Na_2SO_4 and the solvent removed under reduced pressure to yield a crude reddish-brown oil. The crude product was purified by flash column chromatography on silica gel (4:1 to 3:2 *n*-heptane/ethyl acetate) to afford compound **S3** (11.8 g, 79% yield) as a clear colourless oil that solidified to a white solid upon storage in the freezer (-20 °C).

TLC: R_f = 0.30 (3:2 *n*-heptane/ethyl acetate), UV.
mp = 62.0 – 65.0 °C. Melting point is in accordance with literature values (mp = 62 – 64 °C).[9]
IR (ATR) $\tilde{\upsilon}_{\max}$ cm^{-1}: 3029 (w), 2955 (m), 2935 (m), 2880 (w), 1664 (s), 1617 (m), 1453 (w), 1421 (w), 1374 (w), 1326 (w), 1281 (w), 1229 (w), 1170 (m), 1155 (w), 1118 (w), 1071 (m), 1054 (w), 1016 (w), 950 (w), 854 (w).
^1H NMR (400 MHz, $CDCl_3$) δ ppm: 5.81 (d, *J* = 2.0 Hz, 1H, C11-H), 4.02 – 3.89 (m, 4H, C12-H_2 + C13-H_2), 2.48 – 2.23 (m, 5H, C4-H_2 + C8-H + C9-H_2), 1.89 (td, *J* = 14.6, 14.0, 4.5 Hz, 1H, C6-H), 1.82 – 1.61 (m, 4H, C5-H_2 + C6-H + C8-H), 1.35 (s, 3H, C7-H_3).
^{13}C NMR (101 MHz, $CDCl_3$) δ ppm: 199.4 (C10), 167.8 (C3), 125.8 (C11), 112.5 (C1), 65.5 (C12/C13), 65.2 (C12/C13), 45.2 (C2), 34.1 (C9), 31.6 (C4), 30.2 (C6), 27.0 (C8), 21.9 (C5), 20.6 (C7).
NMR data are in accordance with literature values.[10]
LC-LRMS (ESI+) *m/z*: 222.99 found for $[M+H]^+$, 223.13 calcd. for $C_{13}H_{19}O_3^+$, R_t = 1.18 min.

(4a*R,8a*S**)-8a-Methylhexahydro-2*H*-spiro[naphthalene-1,2'-[1,3]dioxolan]-6(5*H*)-one (S4)**

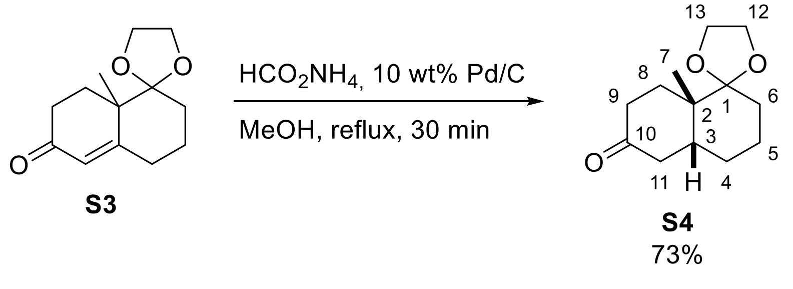

To a solution of compound **S3** (8.40 g, 37.8 mmol, 1.00 equiv.) and HCO_2NH_4 (7.15 g, 113 mmol, 3.00 equiv.) in methanol (300 mL) at room temperature was added 10 wt% Pd/C (463 mg, 0.379 mmol Pd, 0.01 equiv. Pd = 1 mol%). The reaction mixture was stirred at reflux for 30 min. The reaction mixture was cooled to room temperature and filtered through a pad of celite® washing with dichloromethane (500 mL). The organic phase was washed with water (2 × 100 mL) and brine (100 mL), dried over anhydrous $MgSO_4$, and the solvent removed under reduced pressure to yield a crude pale-yellow oil. The crude product was purified by flash chromatography on silica gel (9:1 to 3:1 *n*-heptane/ethyl acetate) to afford compound **S4** (6.16 g, 73% yield) as a clear colourless oil that solidified to a white solid upon storage in the freezer (-20 °C).

Note: Optimisation of the hydrogenation was required to obtain good *dr* of the *cis*- (**S4**) and *trans*-fused (**S4'**) diastereoisomers since they are inseparable in the used eluent system. With the optimised conditions, only trace amounts (under the limit of quantification) of the *trans*-fused diastereoisomer[11] were detected in the crude and the purified product by NMR. The crude product is used in the subsequent step without purification at this stage. The Leuckart–Wallach amine product was observed as a side-product in various amounts when the internal pressure of the reaction system is too high (closed system with high nitrogen flow).

TLC: R_f = 0.28 (3:1 *n*-heptane/ethyl acetate), $KMnO_4$ stain.
mp = 49.0 – 52.0 °C. Melting point is in accordance with literature values (mp = 52 – 54 °C).[12]
IR (ATR) $\tilde{\upsilon}_{\max}$ cm^{-1}: 2968 (m), 2934 (s), 2897 (m), 2878 (s), 2860 (m), 1703 (s), 1471 (w), 1455 (w), 1420 (w), 1377 (w), 1345 (w), 1327 (w), 1221 (w), 1188 (m), 1157 (m), 1116 (m), 1089 (s), 1035 (m), 996 (w), 946 (w), 911 (w), 872 (m).
^{1}H NMR (400 MHz, $CDCl_3$) δ ppm: 4.01 – 3.88 (m, 4H, C12-H_2 + C13-H_2), 2.62 (dd, *J* = 14.6, 5.6 Hz, 1H, C11-H), 2.48 – 2.37 (m, 1H, C9-H), 2.36 – 2.28 (m, 1H, C9-H), 2.20 – 2.02 (m, 3H, C3-H + C8-H + C11-H), 1.76 (dddd, *J* = 13.8, 7.0, 3.1, 1.6 Hz, 1H, C8-H), 1.69 – 1.45 (m, 5H, C4-H + C5-H_2 + C6-H_2), 1.30 – 1.18 (m, 4H, C4-H + C7-H_3).
^{13}C NMR (101 MHz, $CDCl_3$) δ ppm: 212.6 (C10), 112.6 (C1), 65.2 (C12/C13), 65.1 (C12/C13), 44.3 (C11), 42.8 (C3), 41.4 (C2), 38.0 (C9), 29.8 (C6), 29.1 (C8), 28.4 (C4), 22.4 (C5), 17.8 (C7).
NMR data are in accordance with literature values.[12]
LRMS (ESI+) *m/z*: 225.11 found for $[M+H]^+$, 225.15 calcd. for $C_{13}H_{21}O_3^+$.

(4a*R,6*R**,8a*S**)-8a-Methyloctahydro-2*H*-spiro[naphthalene-1,2'-[1,3]dioxolan]-6-ol (S5) and (4a*R**,6*S**,8a*S**)-8a-methyloctahydro-2*H*-spiro[naphthalene-1,2'-[1,3]dioxolan]-6-ol (S5')**

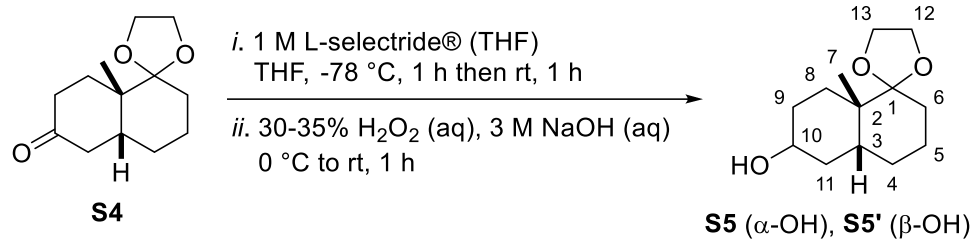

To a solution of compound **S4** (5.89 g, 26.3 mmol, 1.0 equiv.) in dry tetrahydrofuran (205 mL) at -78 °C was added 1 M L-selectride® in tetrahydrofuran (52.5 mL, 52.6 mmol, 2.0 equiv.). The reaction mixture was stirred at -78 °C for one hour and then at room temperature for one hour. The reaction mixture was cooled to 0 °C, quenched with water (3.5 mL), 3 M aqueous NaOH (17.3 mL), and 30-35% aqueous H_2O_2 (17.3 mL), and stirred for one hour at room temperature. The reaction mixture was added water (150 mL) and extracted with ethyl acetate (3 × 175 mL). The combined organic phases were washed with water (100 mL) and brine (100 mL), dried over anhydrous Na_2SO_4, and the solvent removed under reduced pressure. The residue was dried under high vacuum to afford a diastereoisomeric mixture of compound **S5** and **S5'** (5.94 g, quant. yield) with a *dr* > 20:1 (**S5**:**S5'**) as a clear colourless oil. The mixture of diastereoisomers was used in the subsequent step without any further purification.

Note: The *dr* was determined by the 1D ^{1}H NMR spectrum ratio of the **S5** and **S5'** methyl (C7-H_3) protons integrals at 1.02 and 0.96 ppm, respectively. The diastereoisomers **S5** and **S5'** can be separated in this step, but a better separation is achieved for the diastereoisomers **1** and **1'** after the ketal deprotection. A preparative scale (1.85 g, 8.27 mmol) reaction was purified by flash column chromatography on silica gel (19:1 to 5:1 dichloromethane/ethyl acetate) to afford compound **S5** (1.14 g, 61% yield) as a clear colourless oil. Only characterisation data for the purified compound **S5** is presented below. The 1D ^{13}C NMR data and spectrum are not presented since peak broadening was observed, and it could not be resolved by VT NMR since decomposition of the product was observed in $CDCl_3$ at elevated temperatures. The 1D ^{1}H NMR spectrum for non-purified diastereoisomeric mixture of compound **S5** and **S5'** is also presented (please see NMR spectra).

Characterisation data for **S5**:
TLC: R_f = 0.26 (5:1 dichloromethane/ethyl acetate), $KMnO_4$ stain.

IR (ATR) $\tilde{\upsilon}_{max}$ cm^{-1}: 3387 (br, m), 2927 (s), 2865 (s), 1462 (w), 1447 (m), 1380 (w), 1363 (w), 1344 (w), 1334 (w), 1309 (w), 1293 (w), 1267 (w), 1192 (m), 1170 (m), 1113 (s), 1083 (s), 1046 (s), 1032 (s), 994 (s), 949 (m), 921 (m), 907 (m), 882 (w), 840 (w).
^{1}H NMR (400 MHz, $CDCl_3$) δ ppm: 3.98 – 3.83 (m, 4H, C12-H_2 + C13-H_2), 3.80 – 3.64 (m, 1H, C10-H), 2.06 – 1.96 (m, 1H, C8-H), 1.94 – 1.81 (m, 1H, C11-H), 1.78 – 1.38 (m, 10H, C3-H + C4-H_2 + C5-H_2 + C6-H_2 + C9-H_2 + C11-H), 1.11 – 0.98 (m, 4H, C7-H_3 + C8-H).
NMR data are in accordance with literature values.[13]
LRMS (ESI+) *m/z*: 227.03 found for $[M+H]^+$, 227.16 calcd. for $C_{13}H_{23}O_3^+$.

(4a*R,6*R**,8a*S**)-6-Hydroxy-8a-methyloctahydronaphthalen-1(2*H*)-one (1) and (4a*R**,6*S**,8a*S**)-6-hydroxy-8a-methyloctahydronaphthalen-1(2*H*)-one (1')**

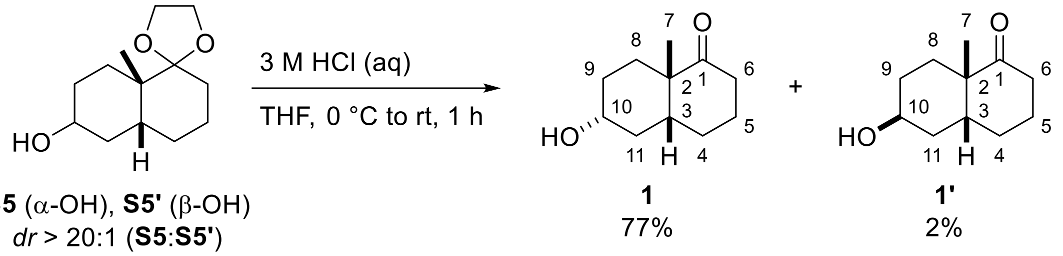

S5 (α-OH), **S5'** (β-OH)
dr > 20:1 (**S5**:**S5'**)

1
77%

1'
2%

To a solution of a diastereoisomeric mixture of compound **S5** and **S5'** (5.94 g, 26.3 mmol, 1.0 equiv.) with a *dr* > 20:1 (**S5**:**S5'**) in tetrahydrofuran (200 mL) at 0 °C was added 3 M aqueous HCl (57.8 mL). The reaction mixture was stirred at room temperature for one hour. The reaction was neutralised with saturated aqueous $NaHCO_3$ and extracted with dichloromethane (3 × 175 mL). The combined organic phases were washed with water (50 mL) and brine (50 mL), dried over anhydrous $MgSO_4$, and the solvent removed under reduced pressure to yield a crude mixture of diastereoisomers as a clear colourless oil. The crude product was purified by flash column chromatography on silica gel (1:0 to 3:1 dichloromethane/ethyl acetate) to afford compound **1** (3.69 g, 77% yield) as a clear colourless oil that solidified to a white solid upon storage in the freezer (-20 °C) and compound **1'** (99.9 mg, 2% yield) as a clear colourless oil that solidified to a white solid upon storage in the freezer (-20 °C) in separate fractions. Combined yield: 3.78 g, 79%.

Characterisation data for **1**:
TLC: R_f = 0.21 (4:1 dichloromethane/ethyl acetate), $KMnO_4$ stain.
mp = 71.0 – 74.0 °C. Melting point is in accordance with literature values (mp = 73.0 – 74.5 °C).[14]
IR (ATR) $\tilde{\upsilon}_{max}$ cm^{-1}: 3386 (br, m), 2935 (s), 2869 (m), 1698 (s), 1463 (m), 1446 (m), 1364 (m), 1312 (w), 1226 (w), 1144 (m), 1104 (m), 1083 (m), 1050 (s), 1013 (m), 989 (s), 923 (m), 884 (w), 824 (m), 770 (w), 701 (m).
^{1}H NMR (400 MHz, $CDCl_3$) δ ppm: 3.60 (tt, *J* = 10.8, 4.2 Hz, 1H, C10-H), 2.62 – 2.45 (m, 1H, C6-H), 2.33 – 2.12 (m, 3H, C4-H + C6-H + C8-H), 2.01 – 1.83 (m, 3H, C3-H + C5-H_2), 1.79 (dq, *J* = 16.4, 3.8 Hz, 1H, C9-H), 1.69 (dtd, *J* = 12.6, 3.8, 2.6 Hz, 1H, C11-H), 1.48 (d, *J* = 13.5 Hz, 1H, C4-H), 1.44 – 1.17 (m, 5H, C7-H_3 + C9-H + C11-H), 0.94 (td, *J* = 13.7, 4.1 Hz, 1H, C8-H).
^{13}C NMR (101 MHz, $CDCl_3$) δ ppm: 215.1 (C1), 71.0 (C10), 48.6 (C2), 43.7 (C3), 38.5 (C11), 38.0 (C6), 33.1 (C8), 32.9 (C9), 26.7 (C7), 26.4 (C4), 22.2 (C5).
NMR data are in accordance with literature values.[15]
LRMS (ESI+) *m/z*: 183.07 found for $[M+H]^+$, 183.14 calcd. for $C_{11}H_{19}O_2^+$.

Characterisation data for **1'**:
TLC: R_f = 0.29 (4:1 dichloromethane/ethyl acetate), $KMnO_4$ stain.
mp = 39.0 – 42.0 °C.
IR (ATR) $\tilde{\upsilon}_{max}$ cm^{-1}: 3400 (br, m), 2929 (s), 2866 (m), 1693 (s), 1467 (w), 1447 (w), 1432 (w), 1379 (w), 1311 (w), 1260 (w), 1230 (w), 1144 (w), 1050 (w), 1030 (m), 993 (w), 960 (w), 820 (w).
^{1}H NMR (400 MHz, $CDCl_3$) δ ppm: 4.11 – 4.02 (m, C10-H), 2.48 (ddd, *J* = 15.8, 9.8, 6.4 Hz, 1H, C6-H), 2.35 – 2.24 (m, 1H, C6-H), 2.20 – 2.11 (m, 1H, C3-H), 2.10 – 1.86 (m, 3H, C4-H + C5-H + C8-H), 1.85 – 1.69 (m, 2H, C5-H + C9-H), 1.63 – 1.48 (m, 4H, C4-H + C9-H + C11-H_2), 1.33 (ddd, *J* = 14.1, 10.3, 4.2 Hz, 1H, C8-H), 1.24 (s, 3H, C7-H_3).
^{13}C NMR (101 MHz, $CDCl_3$) δ ppm: 216.0 (C1), 66.8 (C10), 49.0 (C2), 40.2 (C3), 38.0 (C6), 35.8 (C11), 30.3 (C9), 28.8 (C8), 26.6 (C4), 24.8 (C7), 23.5 (C5).
NMR data are in accordance with literature values.[16]
LRMS (ESI+) *m/z*: 183.07 found for $[M+H]^+$, 183.14 calcd. for $C_{11}H_{19}O_2^+$.

5,5-Dibromobarbituric acid (S6)

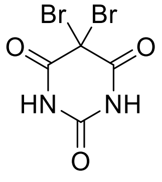

S6

Following procedure of Whitmarsh-Everiss *et al.*[11] using barbituric acid (10.0 g, 78.1 mmol) afforded compound **S6** (16.1 g, 72% yield) as a white solid.

^{1}H NMR (400 MHz, DMSO-d_6) δ ppm: 11.8 (s).
^{13}C NMR (101 MHz, DMSO-d_6) δ ppm: 163.5, 149.0, 48.8.
NMR data are in accordance with literature values.[11]

(2*S,4a*R**,6*R**,8a*S**)-2-Bromo-6-hydroxy-8a-methyloctahydronaphthalen-1(2*H*)-one (6) and (2*R**,4a*R**,6*R**,8a*S**)-2-bromo-6-hydroxy-8a-methyloctahydronaphthalen-1(2*H*)-one (6')**

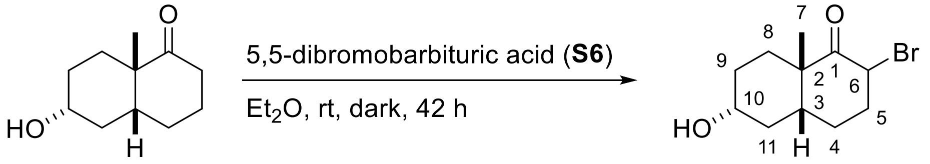

To a solution of compound **1** (1.02 g, 5.62 mmol, 1.0 equiv.) in dry diethyl ether (28 mL) at room temperature was added 5,5-dibromobarbituric acid (**S6**; 0.964 g, 3.37 mmol, 0.6 equiv.). The reaction mixture was covered in aluminium foil and stirred in the dark at room temperature for 42 hours. The reaction mixture was added water (25 mL) and the phases were separated. The organic phase was washed with water (25 mL), dried over anhydrous $MgSO_4$, and the solvent removed under reduced pressure to yield a crude light-brown oil. The crude product was purified by flash column chromatography on silica gel (3:2 *n*-heptane/ethyl acetate) to afford a diastereoisomeric mixture of compound **6** and **6'** (1.10 g, 75% yield) with a *dr* = 20:1 (**6**:**6'**) as a clear colourless oil that solidified to a white solid upon storage in the freezer (-20 °C) under argon and covered in aluminium foil.

Note: The *dr* was determined by the 1D ^{1}H NMR spectrum ratio of the **6** and **6'** α-protons (C6-H) integrals at 4.49 and 4.40 ppm, respectively. Only NMR data for the major diastereoisomer is presented below.

TLC: R_f = 0.17 (3:2 *n*-heptane/ethyl acetate), $KMnO_4$ stain.
mp = 47.0 – 52.0 °C.
IR (ATR) $\tilde{\upsilon}_{\max}$ cm^{-1}: 3381 (br, m), 2934 (s), 2868 (m), 1702 (s), 1442 (m), 1371 (w), 1342 (w), 1298 (w), 1242 (m), 1210 (w), 1100 (w), 1043 (m), 990 (w), 920 (m).
^{1}H NMR (400 MHz, $CDCl_3$) δ ppm: 4.49 (t, *J* = 4.9 Hz, 1H, C6-H), 3.73 (tt, *J* = 9.0, 4.0 Hz, 1H, C10-H), 2.67 – 2.54 (m, 1H, C5-H), 2.48 – 2.30 (m, 2H, C4-H + C8-H), 2.23 – 2.11 (m, 1H, C5-H), 1.95 – 1.85 (m, 1H, C3-H), 1.84 – 1.67 (m, 3H, C4-H + C9-H + C11-H), 1.51 (s, 3H, C7-H_3), 1.34 – 1.17 (m, 2H, C9-H + C11-H), 1.05 – 0.91 (m, 1H, C8-H).
^{13}C NMR (101 MHz, $CDCl_3$) δ ppm: 207.5 (C1), 69.6 (C10), 49.4 (C2), 48.6 (C6), 42.0 (C3), 37.5 (C11), 32.6 (C8), 32.3 (C5), 32.0 (C9), 28.6 (C7), 23.8 (C4).
LRMS (ESI+) *m/z*: 260.91 found for $[M+H]^+$, 261.05 calcd. for $C_{11}H_{18}BrO_2^+$.
HRMS (ESI+) *m/z*: 283.0302 found for $[M+Na]^+$, 283.0304 calcd. for $C_{11}H_{17}BrNaO_2^+$ (Δ = -0.78 ppm).

Note: The relative stereochemistry and identification of the diastereoisomers were determined by structural analysis using the coupling constants of the α-protons (Figure S1 and Figure S2 and accompanying discussion). Residual ethyl acetate observed in the 1D ^{1}H NMR spectrum.

Synthesis of analogues

Synthesis of quinoline analogues

General procedure A

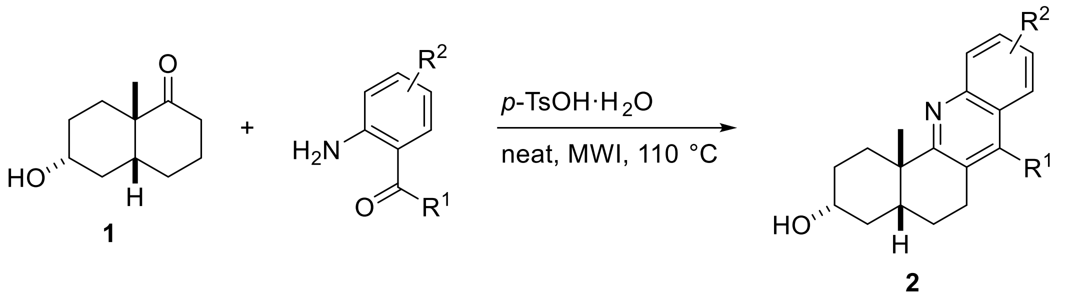

A mixture of compound **1** (1.0 equiv.), the appropriate *o*-aminoaceto-/benzophenone (1.3 equiv.), and *p*-toluenesulfonic acid monohydrate (1.3 equiv.) in a 0.2-0.5 mL Biotage microwave vial at room temperature under an ambient air atmosphere was sealed and then heated neat at 110 °C *via* microwave irradiation for the indicated time. The reaction mixture was dissolved in dichloromethane and washed with saturated aqueous Na_2CO_3 and water (2×). The organic phase was dried over anhydrous $MgSO_4$ and the solvent removed under reduced pressure to yield the crude products. The crude products were purified by flash column chromatography on silica gel to afford the quinoline analogues (**2**).

(3*R,4a*R**,12b*S**)-7,12b-Dimethyl-1,2,3,4,4a,5,6,12b-octahydrobenzo[*c*]acridin-3-ol (2a)**

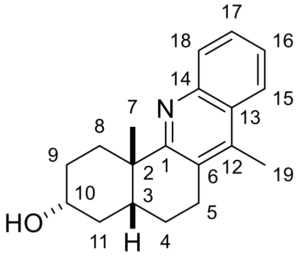

2a

Following general procedure A using compound **1** (17.4 mg, 95.5 µmol) and 2′-aminoacetophenone (16.9 mg, 125 µmol), heating for two hours, and eluting with 13:7 *n*-heptane/ethyl acetate afforded compound **2a** (10.4 mg, 39% yield) as an off-white solid.

TLC: R_f = 0.31 (13:7 *n*-heptane/ethyl acetate), UV.
mp = 71.0 – 74.0 °C.
IR (ATR) $\tilde{\upsilon}_{\max}$ cm^{-1}: 3273 (br, m), 3067 (w), 2929 (s), 2869 (m), 1620 (w), 1577 (m), 1561 (w), 1495 (m), 1436 (s), 1399 (w), 1366 (m), 1351 (m), 1320 (m), 1299 (w), 1161 (m), 1141 (w), 1058 (m), 1035 (s), 1019 (s), 980 (w), 950 (m), 754 (s), 730 (s), 705 (s).
^{1}H NMR (400 MHz, $CDCl_3$) δ ppm: 7.97 (dd, *J* = 8.4, 3.5 Hz, 2H, C15-H + C18-H), 7.58 (t, *J* = 7.6 Hz, 1H, C17-H), 7.45 (t, *J* = 7.6 Hz, 1H, C16-H), 3.74 (d, *J* = 12.0 Hz, 1H, C10-H), 3.22 (dt, *J* = 13.6, 3.6 Hz, 1H, C8-H), 3.03 – 2.83 (m, 2H, C5-H_2), 2.54 (s, 3H, C19-H_3), 2.40 – 2.26 (m, 1H, C4-H), 1.96 – 1.71 (m, 4H, C3-H + C4-H + C9-H + C11-H), 1.41 – 1.15 (m, 5H, C7-H_3 + C8-H + C11-H), 1.08 – 0.93 (m, 1H, C9-H).
^{13}C NMR (101 MHz, $CDCl_3$) δ ppm: 162.2 (C1), 146.6 (C14), 141.1 (C12), 129.9 (C18), 127.7 (C17), 127.0 (C6), 126.7 (C13), 125.4 (C16), 123.2 (C15), 71.3 (C10), 40.8 (C2), 39.7 (C3), 37.6 (C11), 35.7 (C8), 33.0 (C9), 31.3 (C7), 23.6 (C5), 23.4 (C4), 13.8 (C19).
LC-LRMS (ESI+) *m/z*: 282.35 found for $[M+H]^+$, 282.19 calcd. for $C_{19}H_{24}NO^+$, R_t = 1.94 min, purity (UV area): >99%.
HRMS (ESI+) *m/z*: 282.1858 found for $[M+H]^+$, 282.1853 calcd. for $C_{19}H_{24}NO^+$ (Δ = 1.86 ppm).

(3*R,4a*R**,12b*S**)-12b-Methyl-7-phenyl-1,2,3,4,4a,5,6,12b-octahydrobenzo[*c*]acridin-3-ol (2b)**

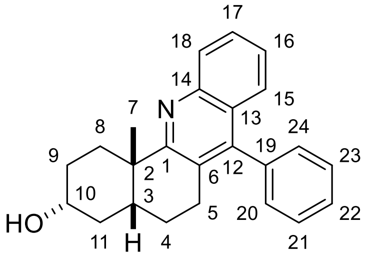

2b

Following general procedure A using compound **1** (76.4 mg, 0.419 mmol) and 2-aminobenzophenone (108 mg, 0.547 mmol), heating for two hours, and eluting with 2:1 *n*-heptane/ethyl acetate afforded compound **2b** (40.4 mg, 28% yield) as a dirt-brown solid.

TLC: R_f = 0.34 (2:1 *n*-heptane/ethyl acetate), UV.
mp = 140 – 142 °C.
IR (ATR) $\tilde{\upsilon}_{\max}$ cm^{-1}: 3313 (br, m), 2929 (s), 2867 (m), 2167 (w), 1569 (w), 1485 (m), 1435 (m), 1396 (w), 1365 (w), 1100 (m), 1067 (m), 1039 (m), 760 (s), 705 (m).
^{1}H NMR (400 MHz, DMSO-d_6) δ ppm: 7.96 (d, *J* = 8.7 Hz, 1H, C18-H), 7.67 – 7.46 (m, 4H, C17-H + C21-H + C22-H + C23-H), 7.39 (ddd, *J* = 8.3, 6.8, 1.3 Hz, 1H, C16-H), 7.31 – 7.24 (m, 2H, C20-H + C24-H), 7.17 (d, *J* = 8.4 Hz, 1H, C15-H), 4.36 (d, *J* = 4.7 Hz, 1H, O-H), 3.54 (td, *J* = 10.9, 5.9 Hz, 1H, C10-H), 3.08 (dt, *J* = 13.2, 3.5 Hz, 1H, C8-H), 2.70 – 2.56 (m, 1H, C5-H), 2.53 – 2.46 (m, 1H, C5-H), 2.21 – 2.07 (m, 1H, C4-H), 1.83 (dq, *J* = 12.5, 3.3 Hz, 1H, C3-H), 1.72 – 1.61 (m, 2H, C9-H + C11-H), 1.58 – 1.48 (m, 1H, C4-H), 1.33 (td, *J* = 13.3, 3.3 Hz, 1H, C8-H), 1.23 (s, 3H, C7-H_3), 1.20 – 1.06 (m, 1H, C11-H), 0.97 – 0.81 (m, 1H, C9-H).
^{13}C NMR (101 MHz, DMSO-d_6) δ ppm: 162.8 (C1), 146.2 (C12), 146.0 (C14), 136.8 (C19), 128.9 (C21/C23), 128.8 (C21/C23), 128.74 (C20/C24), 128.73 (C20/C24), 128.66 (C18), 128.2 (C22), 127.8 (C17), 126.4 (C6), 125.8 (C13), 125.7 (C16), 125.0 (C15), 69.1 (C10), 40.7 (C2), 38.9 (C3), 37.5 (C11), 35.2 (C8), 32.7 (C9), 31.3 (C7), 23.9 (C5), 22.7 (C4).
LC-LRMS (ESI+) *m/z*: 344.25 found for $[M+H]^+$, 344.20 calcd. for $C_{24}H_{26}NO^+$, R_t = 2.07 min, purity (UV area): ≥99%.
HRMS (ESI+) *m/z*: 344.2020 found for $[M+H]^+$, 344.2009 calcd. for $C_{19}H_{24}NO^+$ (Δ = 3.26 ppm).

Note: The peak for the C5-H proton at 2.53 – 2.46 ppm in the 1D ^{1}H NMR spectrum and the peak for the C3 carbon at 38.9 ppm in the 1D ^{13}C NMR spectrum overlap with the dimethyl sulfoxide-d_6 solvent peaks, thus increasing the integral, but the chemical shifts and integral were confirmed by correlations in the 2D ^{1}H-^{1}H COESY and ^{1}H-^{13}C HSQC and HMBC spectra.

(3*R,4a*R**,12b*S**)-10-Bromo-7,12b-dimethyl-1,2,3,4,4a,5,6,12b-octahydrobenzo[*c*]acridin-3-ol (2c)**

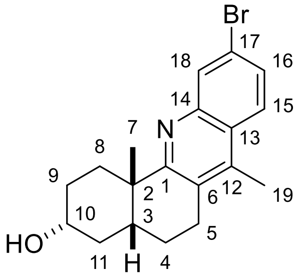

2c

Following general procedure A using compound **1** (75.0 mg, 0.411 mmol) and 2'-amino-4'-bromoacetophenone (115 mg, 0.537 mmol), heating for 30 minutes, and eluting with 2:1 *n*-heptane/ethyl acetate afforded compound **2c** (58.1 mg, 39% yield) as an off-white solid.

TLC: R_f = 0.22 (2:1 *n*-heptane/ethyl acetate), UV.
mp = 155.0 – 158.0 °C.

IR (ATR) $\tilde{\upsilon}_{\max}$ cm^{-1}: 3252 (br, s), 3072 (w), 2961 (m), 2941 (s), 2915 (s), 2857 (s), 1596 (s), 1573 (m), 1555 (w), 1482 (m), 1435 (m), 1417 (w), 1369 (m), 1307 (w), 1256 (w), 1190 (w), 1160 (w), 1125 (w), 1076 (w), 1046 (m), 1034 (m), 1022 (m), 949 (w), 926 (w), 897 (m), 875 (w), 810 (s), 773 (s), 751 (w), 706 (w), 666 (w), 582 (w).
^{1}H NMR (400 MHz, $CDCl_3$) δ ppm: 8.18 (s, 1H, C18-H), 7.82 (d, *J* = 9.0 Hz, 1H, C15-H), 7.52 (d, *J* = 8.7 Hz, 1H, C16-H), 3.76 (t, *J* = 11.3 Hz, 1H, C10-H), 3.16 (d, *J* = 13.7 Hz, 1H, C8-H), 2.97 – 2.82 (m, 2H, C5-H_2), 2.52 (s, 3H, C19-H_3), 2.37 – 2.25 (m, 1H, C4-H), 1.94 – 1.72 (m, 4H, C3-H + C4-H + C9-H + C11-H), 1.40 – 1.12 (m, 5H, C7-H_3 + C8-H + C11-H), 1.03 – 0.90 (m, 1H, C9-H).
^{13}C NMR (101 MHz, $CDCl_3$) δ ppm: 163.5 (C1), 147.4 (C14), 141.4 (C12), 132.0 (C18), 128.8 (C16), 127.6 (C6), 125.4 (C13), 124.8 (C15), 121.8 (C17), 71.2 (C10), 40.9 (C2), 39.5 (C3), 37.5 (C11), 35.7 (C8), 33.0 (C9), 31.2 (C7), 23.6 (C5), 23.3 (C4), 13.9 (C19).
LC-LRMS (ESI+) *m/z*: 359.96 found for $[M+H]^+$, 360.10 calcd. for $C_{19}H_{23}BrNO^+$, R_t = 2.06 min, purity (UV area): ≥95%.
HRMS (ESI+) *m/z*: 360.0961 found for $[M+H]^+$, 360.0958 calcd. for $C_{19}H_{23}BrNO^+$ (Δ = 0.90 ppm).

(3*R,4a*R**,12b*S**)-7-(4-Fluorophenyl)-12b-methyl-1,2,3,4,4a,5,6,12b-octahydrobenzo[*c*]acridin-3-ol (2d)**

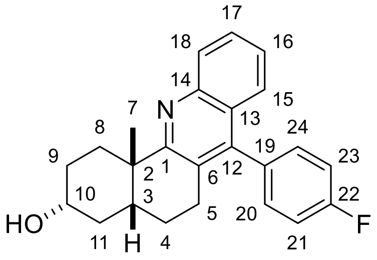

2d

Following general procedure A using compound **1** (79.1 mg, 0.434 mmol) and 2-amino-4'-fluorobenzophenone (121 mg, 0.564 mmol), heating for two hours, and eluting with 5:1 to 2:1 *n*-heptane/ethyl acetate afforded compound **2d** (28.9 mg, 18% yield) as a dirt-brown solid.

TLC: R_f = 0.27 (2:1 *n*-heptane/ethyl acetate), UV.
mp = 67 – 68 °C.
IR (ATR) $\tilde{\upsilon}_{\max}$ cm^{-1}: 3336 (br, m), 3061 (w), 2923 (s), 2856 (m), 2367 (w), 2335 (w), 1489 (m), 1436 (m), 1397 (w), 1354 (w), 1092 (m), 1066 (m), 1039 (m), 761 (s).
^{1}H NMR (400 MHz, DMSO-d_6) δ ppm: 8.01 – 7.92 (m, 1H, C18-H), 7.70 – 7.61 (m, 1H, C17-H), 7.43 – 7.32 (m, 5H, C16-H + C20-H + C21-H + C23-H + C24-H), 7.20 – 7.15 (m, 1H, C15-H), 4.42 – 4.36 (m, 1H, O-H), 3.61 – 3.47 (m, 1H, C10-H), 3.07 (dt, *J* = 13.1, 3.5 Hz, 1H, C8-H), 2.69 – 2.58 (m, 1H, C5-H), 2.54 – 2.44 (m, 1H, C5-H), 2.19 – 2.07 (m, 1H, C4-H), 1.86 – 1.78 (m, 1H, C3-H), 1.71 – 1.63 (m, 2H, C9-H + C11-H), 1.58 – 1.50 (m, 1H, C4-H), 1.32 (td, *J* = 13.4, 3.4 Hz, 1H, C8-H), 1.22 (s, 3H, C7-H_3), 1.13 (q, *J* = 12.5 Hz, 1H, C11-H), 0.95 – 0.84 (m, 1H, C9-H).
^{13}C NMR (101 MHz, DMSO-d_6) δ ppm: 162.9 (C1), 161.7 (d, *J* = 244.1 Hz, C22), 146.0 (C14), 145.2 (C12), 133.0 (d, *J* = 3.3 Hz, C19), 131.0 (d, *J* = 8.2 Hz, C20 + C24), 128.7 (C18), 128.2 (C17), 126.7 (C6), 125.9 (C13), 125.8 (C16), 124.9 (C15), 115.8 (d, *J* = 21.0 Hz, C21 + C22), 69.1 (C10), 40.7 (C2), 38.9 (C3), 37.5 (C11), 35.2 (C8), 32.6 (C9), 31.3 (C7), 23.9 (C5), 22.7 (C4).
^{19}F NMR (377 MHz, DMSO-d_6) δ ppm: -114.4.
LC-LRMS (ESI+) *m/z*: 362.11 found for $[M+H]^+$, 362.19 calcd. for $C_{24}H_{25}FNO^+$, R_t = 1.48 min, purity (UV area): ≥95%.
HRMS (ESI+) *m/z*: 362.1924 found for $[M+H]^+$, 362.1915 calcd. for $C_{24}H_{25}FNO^+$ (Δ = 2.55 ppm).

Note: The peak for the C5-H proton at 2.54 – 2.44 ppm in the 1D ^{1}H NMR spectrum and the peak for the C3 carbon at 38.9 ppm in the 1D ^{13}C NMR spectrum overlap with the dimethyl sulfoxide-d_6 solvent peaks, thus increasing the integral, but the chemical shifts and integral were confirmed by correlations in the 2D ^{1}H-^{1}H COESY and ^{1}H-^{13}C HSQC and HMBC spectra.

(3*R,4a*R**,12b*S**)-9-Chloro-12b-methyl-7-phenyl-1,2,3,4,4a,5,6,12b-octahydrobenzo[*c*]acridin-3-ol (2e)**

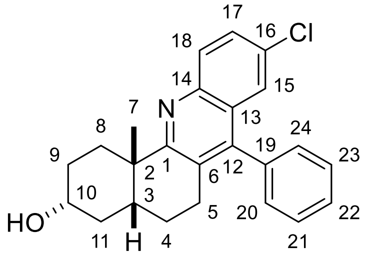

2e

Following general procedure A using compound **1** (75.0 mg, 0.411 mmol) and 2-amino-5-chlorobenzophenone (124 mg, 0.537 mmol), heating for 30 minutes, and eluting with 3:1 *n*-heptane/ethyl acetate afforded compound **2e** (126 mg, 81% yield) as yellow solid.

TLC: R_f = 0.16 (3:1 *n*-heptane/ethyl acetate), UV.
mp = 150.5 – 153.5 °C.
IR (ATR) $\tilde{\upsilon}_{\max}$ cm^{-1}: 3337 (br, m), 3080 (w), 3057 (w), 3024 (w), 2924 (s), 2857 (s), 1602 (w), 1567 (m), 1478 (s), 1440 (m), 1365 (m), 1353 (m), 1335 (w), 1317 (w), 1266 (w), 1166 (w), 1126 (w), 1100 (w), 1066 (m), 1038 (m), 1009 (w), 973 (w), 962 (w), 943 (w), 876 (w), 824 (m), 769 (w), 702 (s), 621 (w), 584 (w).
^{1}H NMR (400 MHz, DMSO-*d*$_6$) δ ppm: 8.00 (d, *J* = 9.0 Hz, 1H, C18-H), 7.67 – 7.50 (m, 4H, C17-H + C20-H + C22-H + C24-H), 7.33 – 7.27 (m, 2H, C21-H + C23-H), 7.09 (d, *J* = 2.4 Hz, 1H, C15-H), 4.38 (d, *J* = 4.8 Hz, 1H, O-H), 3.59 – 3.48 (m, 1H, C10-H), 3.04 (dt, *J* = 13.2, 3.5 Hz, 1H, C8-H), 2.69 – 2.58 (m, 1H, C5-H), 2.55 – 2.45 (m, 1H, C5-H), 2.19 – 2.07 (m, 1H, C4-H), 1.83 (dd, *J* = 12.7, 3.4 Hz, 1H, C3-H), 1.71 – 1.62 (m, 2H, C9-H + C11-H), 1.57 – 1.48 (m, 1H, C4-H), 1.33 (td, *J* = 13.4, 3.2 Hz, 1H, C8-H), 1.23 (s, 3H, C7-H_3), 1.11 (q, J = 12.9 Hz, 1H, C11-H), 0.94 – 0.83 (m, 1H, C9-H).
^{13}C NMR (101 MHz, DMSO-*d*$_6$) δ ppm: 163.7 (C1), 145.5 (C12), 144.4 (C14), 136.0 (C19), 131.0 (C18), 130.1 (C16), 129.1 (C20/C24), 129.0 (C20/C24), 128.74 (C17), 128.70 (C21 + C23), 128.2 (C22), 127.8 (C6), 126.6 (C13), 123.5 (C15), 69.0 (C10), 40.8 (C2), 38.8 (C3), 37.5 (C11), 35.1 (C8), 32.6 (C9), 31.2 (C7), 24.0 (C5), 22.6 (C4).
LC-LRMS (ESI+) *m/z*: 378.05 found for $[M+H]^+$, 378.16 calcd. for $C_{24}H_{25}ClNO^+$, R_t = 3.19 min, purity (UV area): >99%.
HRMS (ESI+) *m/z*: 378.1621 found for $[M+H]^+$, 378.1619 calcd. for $C_{24}H_{25}ClNO^+$ (Δ = 0.59 ppm).

Note: The peak for the C5-H proton at 2.55 – 2.45 ppm in the 1D ^{1}H NMR spectrum overlaps with the dimethyl sulfoxide-*d*$_6$ solvent peak, thus increasing the integral, but the chemical shift and integral were confirmed by correlations in the 2D ^{1}H-^{1}H COESY and ^{1}H-^{13}C HSQC and HMBC spectra.

Synthesis of azaindole analogue

(3*R,4a*R**,11b*S**)-11b-Methyl-2,3,4,4a,5,6,11,11b-octahydro-1*H*-benzo[*g*]pyrido[2,3-*b*]indol-3-ol (3)**

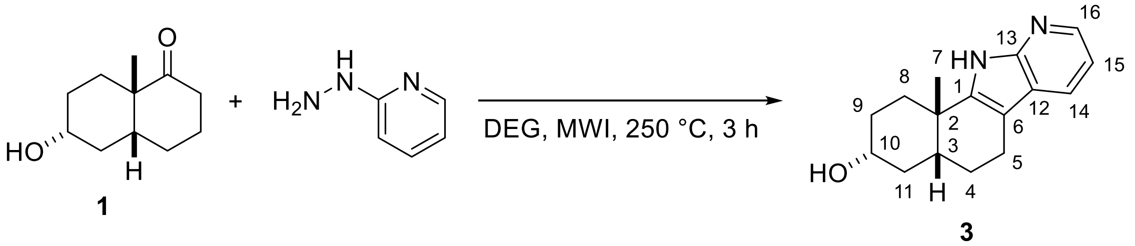

A suspension of compound **1** (245 mg, 1.35 mmol, 1.0 equiv.) and 2-hydrazinopyridine (221 mg, 2.03 mmol, 1.5 equiv.) in diethylene glycol (1.5 mL) in a 0.5-2.0 mL Biotage microwave vial at room temperature under an ambient air atmosphere was sealed and then heated at 250 °C *via* microwave irradiation for three hours. The reaction mixture was poured into saturated aqueous $NaHCO_3$ (25 mL) and extracted with ethyl acetate (3 × 50 mL). The combined organic phases were dried over anhydrous

Na_2SO_4 and the solvent removed under reduced pressure to yield a crude brown oil. The crude product was purified twice by flash column chromatography on silica gel (1. 24:1 dichloromethane/methanol and 2. 93:7 dichloromethane/isopropanol) to afford compound **3** (170 mg, 49% yield) as a cream-white solid.

TLC: R_f = 0.18 (93:7 dichloromethane/isopropanol), UV.
mp = 201.0 – 204.0 °C.
IR (ATR) $\tilde{\upsilon}_{\max}$ cm^{-1}: 3151 (br, s), 3095 (m), 3056 (m), 2926 (s), 2856 (s), 1610 (w), 1586 (w), 1522 (w), 1491 (w), 1444 (m), 1413 (m), 1386 (w), 1366 (m), 1341 (w), 1323 (w), 1304 (w), 1284 (m), 1218 (w), 1187 (w), 1176 (w), 1159 (w), 1121 (m), 1098 (m), 1072 (m), 1033 (m), 948 (w), 790 (w), 767 (m), 735 (m), 621 (w), 547 (w).
^{1}H NMR (400 MHz, $CDCl_3$) δ ppm: 10.59 (br s, 1H, N-H), 8.21 (dd, *J* = 4.9, 1.5 Hz, 1H, C16-H), 7.76 (dd, *J* = 7.8, 1.5 Hz, 1H, C14-H), 7.03 (dd, *J* = 7.7, 4.9 Hz, 1H, C15-H), 3.69 (tt, *J* = 10.8, 4.1 Hz, 1H, C10-H), 2.74 – 2.67 (m, 2H, C5-H_2), 2.35 (dt, *J* = 14.3, 3.6 Hz, 1H, C8-H), 2.26 – 2.15 (m, 1H, C4-H), 1.88 – 1.69 (m, 4H, C3-H + C4-H + C9-H + C11-H), 1.58 (td, *J* = 13.9, 3.3 Hz, 1H, C8-H), 1.42 (q, *J* = 12.0 Hz, 1H, C11-H), 1.35 (s, 3H, C7-H_3), 1.02 (dtd, *J* = 13.7, 11.1, 3.3 Hz, 1H, C9-H).
^{13}C NMR (101 MHz, $CDCl_3$) δ ppm: 149.1 (C13), 141.1 (C1), 140.3 (C16), 126.2 (C14), 121.1 (C12), 115.2 (C15), 106.8 (C6), 70.6 (C10), 40.4 (C3), 36.6 (C11), 35.6 (C8), 34.9 (C2), 32.7 (C9), 30.6 (C7), 24.4 (C4), 17.2 (C5).
LC-LRMS (ESI+) *m/z*: 257.16 found for $[M+H]^+$, 257.16 calcd. for $C_{16}H_{21}N_2O^+$, R_t = 0.87 min, purity (UV area): >99%.
HRMS (ESI+) *m/z*: 257.1647 found for $[M+H]^+$, 257.1648 calcd. for $C_{16}H_{21}N_2O^+$ (Δ = -0.53 ppm).

Synthesis of spiropyrroloquinoxaline analogues

(1*S,4a*R**,6*R**,8a*S**)-8a-Methyl-3,4,4a,5,6,7,8,8a-octahydro-2*H*,5'*H*-spiro[naphthalene-1,4'-pyrrolo[1,2-*a*]quinoxalin]-6-ol (4) and (1*R**,4a*R**,6*R**,8a*S**)-8a-methyl-3,4,4a,5,6,7,8,8a-octahydro-2*H*,5'*H*-spiro[naphthalene-1,4'-pyrrolo[1,2-*a*]quinoxalin]-6-ol (4')**

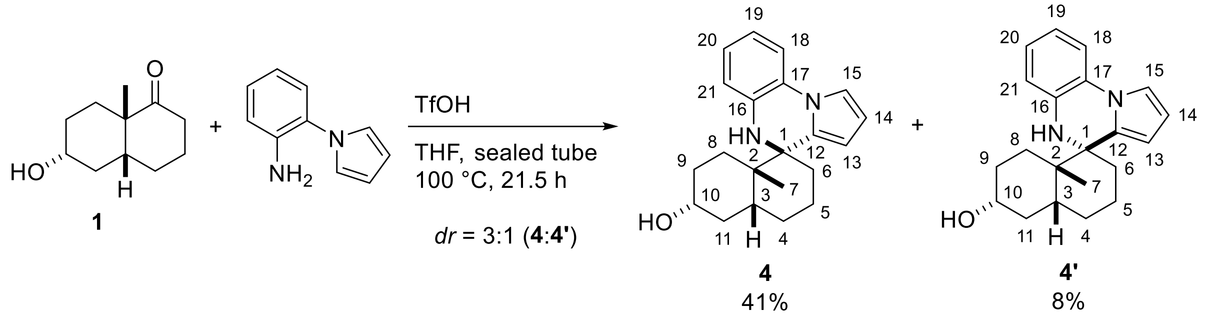

A pressure tube containing compound **1** (150 mg, 0.823 mmol, 1.00 equiv.) and 1-(2-aminophenyl)pyrrole (138 mg, 0.869 mmol, 1.05 equiv.) was evacuated and backfilled with argon three times. The tube was added dry tetrahydrofuran (3.7 mL) and triflic acid (14.6 µL, 165 µmol, 0.20 equiv.), flushed with argon for 15 minutes at room temperature, and sealed. The reaction mixture was stirred at 100 °C in a sealed tube for 21.5 hours. The reaction mixture was cooled to 0 °C, quenched with saturated aqueous $NaHCO_3$ (5 mL), and extracted with ethyl acetate (3 × 25 mL). The combined organic phases were dried over anhydrous Na_2SO_4 and the solvent removed under reduced pressure to yield a crude mixture of diastereoisomers with a *dr* = 3:1 (**4**:**4'**) as a pale-yellow solid. The crude product was purified by flash column chromatography on silica gel (3:1 to 2:1 *n*-heptane/ethyl acetate) to yield the slightly impure diastereoisomers in separate fractions. One diastereoisomer was re-purified by flash column chromatography on neutralised (with triethylamine) silica gel (75:23:2 *n*-heptane/ethyl acetate/triethylamine) applying pressure with nitrogen to afford compound **4** (109 mg, 41% yield) as a pale-yellow solid, and the other diastereoisomer was re-purified twice by flash column chromatography on neutralised (with triethylamine) silica gel (1. 75:23:2 to 67:31:2 *n*-heptane/ethyl acetate/triethylamine and 2. 75:23:2 *n*-heptane/ethyl acetate/triethylamine) applying pressure with nitrogen to afford **4'** (22.4 mg, 8% yield) as a yellow solid. Combined yield: 131 mg, 49%.

Note: The *dr* was determined by the crude 1D ^{1}H NMR spectrum ratio of the **4** and **4'** methyl (C7-H_3) protons integrals at 0.65 and 0.56 ppm, respectively. Both diastereoisomers were found to be unstable under aerobic acidic conditions. Thus, it is important to ensure argon atmosphere during the reaction, neutralising slightly acidic silica gel and applying pressure with nitrogen, and not using chloroform-*d* as the NMR solvent.

Characterisation data for **4**:
TLC: R_f = 0.13 (75:23:2 *n*-heptane/ethyl acetate/triethylamine), UV.
dec pt = 195.0 – 198.0 °C.
IR (ATR) $\tilde{\upsilon}_{\max}$ cm^{-1}: 3551 (m), 3390 (s), 3130 (w), 3106 (w), 3068 (w), 2991 (w), 2976 (w), 2957 (m), 2924 (s), 2850 (s), 1731 (w), 1710 (w), 1609 (m), 1512 (s), 1489 (s), 1465 (s), 1427 (s), 1406 (m), 1379 (w), 1363 (w), 1347 (m), 1333 (s), 1315 (s), 1296 (m), 1286 (m), 1260 (m), 1243 (w), 1217 (w), 1189 (w), 1171 (m), 1158 (m), 1141 (w), 1111 (w), 1063 (w), 1044 (w), 1012 (w), 990 (m), 947 (m), 926 (w), 887 (w), 754 (s), 741 (m), 698 (m).
^{1}H NMR (800 MHz, DMSO-d_6, 348 K) δ ppm: 7.33 – 7.29 (m, 2H, C15-H + C18-H), 6.97 (dd, *J* = 7.9, 1.4 Hz, 1H, C21-H), 6.83 (td, *J* = 7.6, 1.4 Hz, 1H, C20-H), 6.52 (td, *J* = 7.5, 1.4 Hz, 1H, C19-H), 6.21 (t, *J* = 3.2 Hz, 1H, C14-H), 5.93 (dd, *J* = 3.5, 1.5 Hz, 1H, C13-H), 5.84 (s, 1H, N-H), 3.98 (d, *J* = 2.3 Hz, 1H, O-H), 3.78 – 3.74 (m, 1H, C10-H), 2.39 (qd, *J* = 13.6, 4.8 Hz, 1H, C4-H), 2.33 (td, *J* = 13.6, 4.3 Hz, 1H, C6-H), 2.00 (td, *J* = 13.3, 4.7 Hz, 1H, C8-H), 1.89 – 1.85 (m, 1H, C3-H), 1.77 (qt, *J* = 13.8, 4.0 Hz, 1H, C5-H), 1.71 (dt, *J* = 14.6, 4.6 Hz, 1H, C11-H), 1.68 – 1.64 (m, 1H, C6-H), 1.58 – 1.42 (m, 4H, C5-H + C9-H_2 + C11-H), 1.41 – 1.37 (m, 1H, C4-H), 0.96 – 0.92 (m, 1H, C8-H), 0.65 (s, 3H, C7-H_3).
^{13}C NMR (201 MHz, DMSO-d_6, 348 K) δ ppm: 137.3 (C16), 129.0 (C12), 124.2 (C20), 123.0 (C17), 115.2 (C19), 113.2 (C18), 113.0 (C15), 112.9 (C21), 108.9 (C14), 106.4 (C13), 63.5 (C10), 59.0 (C1), 44.3 (C2), 35.1 (C6), 34.5 (C3), 33.7 (C11), 28.8 (C4/C9), 28.7 (C4/C9), 23.3 (C8), 19.6 (C5), 18.6 (C7).
LC-LRMS (ESI+) *m/z*: 323.16 found for $[M+H]^+$, 323.21 calcd. for $C_{21}H_{27}N_2O^+$, R_t = 1.92 min, purity (UV area): ≥95%.
HRMS (ESI+) *m/z*: 323.2119 found for $[M+H]^+$, 323.2118 calcd. for $C_{21}H_{27}N_2O^+$ (Δ = 0.38 ppm).

Note: Peak broadening in the 1D ^{13}C NMR spectrum observed which is resolved by VT NMR. The relative stereochemistry was determined by the observed NOEs between the amine (N-H) proton (δ_H = 6.04 ppm at 400 MHz, rt) and both the methyl (C7-H_3) and C3-H protons in the 2D ^{1}H-^{1}H NOESY spectrum (please see NMR spectra).

Characterisation data for **4'**:
TLC: R_f = 0.12 (67:31:2 *n*-heptane/ethyl acetate/triethylamine), UV.
dec pt = 155.0 – 158.0 °C.
IR (ATR) $\tilde{\upsilon}_{\max}$ cm^{-1}: 3403 (m), 3285 (br, m), 3144 (w), 3107 (w), 3064 (w), 3018 (w), 2919 (s), 2873 (m), 1695 (w), 1671 (m), 1518 (s), 1484 (m), 1470 (m), 1419 (m), 1381 (w), 1365 (w), 1342 (m), 1331 (m), 1313 (m), 1294 (m), 1242 (w), 1198 (w), 1185 (w), 1172 (w), 1137 (w), 1100 (w), 1037 (m), 987 (w), 961 (w), 932 (w), 777 (w), 742 (s), 708 (m), 689 (w).
^{1}H NMR (800 MHz, DMSO-d_6, 348 K) δ ppm: 7.36 – 7.31 (m, 2H, C15-H + C18-H), 6.84 – 6.81 (m, 2H, C20-H + C21-H), 6.57 – 6.51 (m, 1H, C19-H), 6.24 – 6.19 (m, 2H, C13-H + C14-H), 5.45 (s, 1H, N-H), 4.03 (d, *J* = 2.8 Hz, 1H, O-H), 3.78 – 3.74 (m, 1H, C10-H), 2.26 (qd, *J* = 12.6, 4.4 Hz, 1H, C4-H), 1.96 – 1.87 (m, 2H, C6-H + C8-H), 1.81 (td, *J* = 12.9, 4.4 Hz, 1H, C6-H), 1.77 – 1.67 (m, 3H, C3-H + C5-H + C11-H), 1.66 – 1.57 (m, 3H, C5-H + C9-H_2), 1.52 – 1.46 (m, 2H, C4-H + C11-H), 1.20 (dt, *J* = 13.2, 4.6 Hz, 1H, C8-H), 0.56 (s, 3H, C7-H_3).
^{13}C NMR (201 MHz, DMSO-d_6, 348 K) δ ppm: 135.9 (C16), 130.8 (C12), 124.0 (C20), 122.9 (C17), 115.5 (C19), 113.2 (C18), 113.1 (C21), 112.7 (C15), 108.9 (C14), 106.2 (C13), 64.1 (C10), 59.3 (C1), 43.5 (C2), 37.9 (C3), 35.8 (C6), 34.1 (C11), 29.3 (C9), 28.4 (C4), 21.7 (C5), 21.5 (C8), 19.8 (C7).
LC-LRMS (ESI+) *m/z*: 323.12 found for $[M+H]^+$, 323.21 calcd. for $C_{21}H_{27}N_2O^+$, R_t = 1.94 min, purity (UV area): 91%.
HRMS (ESI+) *m/z*: 323.2127 found for $[M+H]^+$, 323.2118 calcd. for $C_{21}H_{27}N_2O^+$ (Δ = 2.86 ppm).

Note: LC-LRMS indicates a purity of 91% (please see LC-LRMS trace) and unknown impurities are observed in the 1D ^{1}H and ^{13}C NMR spectra which might be due to the instability of the product. Peak broadening in the 1D ^{13}C NMR spectrum observed which is resolved by VT NMR. The relative stereochemistry was determined by an observed NOE between the pyrrole (C13-H) proton and the methyl (C7-H_3) protons in the 2D ^{1}H-^{1}H NOESY spectrum (please see NMR spectra).

Synthesis of spirodihydroquinazolinone analogues

General procedure B

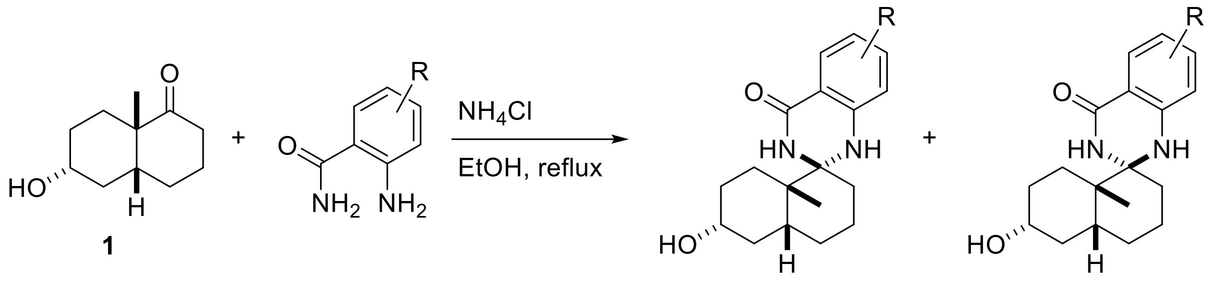

1 5 5'

To a solution of compound **1** (1.0 equiv.) in absolute ethanol (0.14 M **1**) at room temperature was added the appropriate anthranilamide (1.4 equiv.) and NH_4Cl (1.4 equiv.). The reaction mixture was stirred at reflux for 48 hours. If necessary, additional anthranilamide was added during the reaction. The reaction mixture was cooled to room temperature and concentrated under reduced pressure. At this stage, an NMR aliquot was taken for determination of the diastereoisomeric ratio. The residue was added ethyl acetate and water. The phases were separated, and the aqueous phase was extracted with ethyl acetate (2×). The combined organic phases were dried over anhydrous Na_2SO_4 and the solvent removed under reduced pressure to yield the crude products as mixtures of diastereoisomers. The crude products were purified by flash column chromatography on silica gel to afford the two diastereoisomers of the spirodihydroquinazolinone analogues (**5** and **5'**) in separate fractions.

(1*S,4a*R**,6*R**,8a*S**)-6-Hydroxy-8a-methyl-3,4,4a,5,6,7,8,8a-octahydro-1'*H*,2*H*-spiro[naphthalene-1,2'-quinazolin]-4'(3'*H*)-one (5a)**

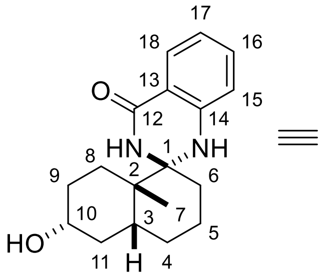

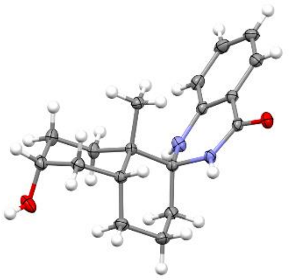

5a **5a** [X-ray]

(1*R,4a*R**,6*R**,8a*S**)-6-Hydroxy-8a-methyl-3,4,4a,5,6,7,8,8a-octahydro-1'*H*,2*H*-spiro[naphthalene-1,2'-quinazolin]-4'(3'*H*)-one (5a')**

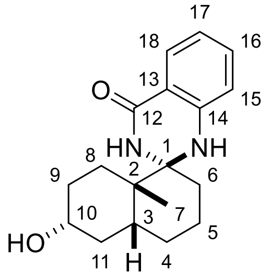

5a'

Following general procedure B using compound **1** (150 mg, 0.823 mmol) and anthranilamide (157 mg, 1.16 mmol) yielded a reaction mixture with a *dr* = 1.2:1 (**5a**:**5a'**) and purifying twice eluting with 1. 83:16:0:1 to 99:0:0:1 to 97:0:2:1 ethyl acetate/dichloromethane/methanol/triethylamine and 2. 99:0:1 to 97:2:1 ethyl acetate/methanol/triethylamine afforded compound **5a** (48.2 mg, 19% yield) as a white solid and **5a'** (52.9 mg, 21% yield) as a white solid in separate fractions. Combined yield (including mixed fractions): 110 mg, 44%.

Note: The *dr* was determined by the crude 1D ^{1}H NMR spectrum ratio of the **5a** and **5a'** methyl (C7-H_3) protons integrals at 0.97 and 0.95 ppm, respectively. A clean mixture of diastereoisomers (9.1 mg, 4%) with a *dr* = 1:1 (**5a**:**5a'**) was isolated in separate mixed fractions.

Characterisation data for **5a**:
TLC: R_f = 0.20 (83:16:1 ethyl acetate/dichloromethane/triethylamine), UV.
mp = 210.0 – 213.0 °C.
IR (ATR) $\tilde{\upsilon}_{\max}$ cm^{-1}: 3448 (br, m), 3397 (s), 3346 (br, m), 3063 (w), 3032 (w), 2989 (w), 2966 (w), 2921 (s), 2862 (m), 1646 (s), 1626 (s), 1607 (s), 1577 (m), 1519 (s), 1487 (m), 1466 (w), 1446 (w), 1419 (m), 1379 (w), 1360 (w), 1335 (w), 1288 (w), 1265 (w), 1225 (w), 1172 (w), 1148 (w), 1110 (w), 1054 (w), 1041 (w), 995 (w), 979 (w), 957 (w), 936 (w), 898 (w), 747 (m), 697 (w), 577 (w).
^{1}H NMR (800 MHz, DMSO-d_6, 298 K) δ ppm: 7.57 (s, 1H, C12(O)-N-H), 7.52 (d, *J* = 7.7 Hz, 1H, C18-H), 7.16 (t, *J* = 7.7 Hz, 1H, C16-H), 6.77 (d, *J* = 8.1 Hz, 1H, C15-H), 6.53 (t, *J* = 7.3 Hz, 1H, C17-H), 6.06 (s, 1H, C14-N-H), 4.32 – 4.24 (m, 1H, O-H), 3.85 – 3.75 (m, 1H, C10-H), 2.26 – 2.09 (m, 1H, C4-H), 1.88 – 1.78 (m, 3H, C3-H + C6-H + C9-H), 1.73 – 1.57 (m, 4H, C6-H + C8-H + C9-H + C11-H), 1.52 (q, *J* = 13.3 Hz, 1H, C5-H), 1.46 – 1.33 (m, 2H, C5-H + C11-H), 1.30 – 1.18 (m, 2H, C4-H + C8-H), 0.97 (s, 3H, C7-H_3).
^{13}C NMR (151 MHz, DMSO-d_6, 353 K) δ ppm: 162.2 (C12), 147.0 (C14), 132.5 (C16), 126.4 (C18), 115.3 (C17), 113.8 (C15), 112.6 (C13), 74.0 (C1), 63.8 (C10), 42.1 (C2), 35.5 (C3), 34.2 (C6), 33.6 (C11), 28.8 (C9), 28.4 (C4), 20.7 (C8), 19.4 (C5), 18.3 (C7).
^{15}N NMR (41 MHz, DMSO-d_6) δ ppm: 118.7 (C12(O)-NH), 80.7 (C14-NH).
LC-LRMS (ESI+) *m/z*: 301.10 found for $[M+H]^+$, 301.19 calcd. for $C_{18}H_{25}N_2O_2^+$, R_t = 1.21 min, purity (UV area): ≥97%.
HRMS (ESI+) *m/z*: 323.1731 found for $[M+Na]^+$, 323.1730 calcd. for $C_{18}H_{24}N_2NaO_2^+$ (Δ = 0.25 ppm).
X-ray (single-crystal): Crystal was grown by vapour diffusion (solvent = dichloromethane/methanol; antisolvent = *n*-pentane) until equilibrium then followed by mixed solvent slow evaporation.

Note: Peak broadening in the 1D ^{13}C NMR spectrum observed which is resolved by VT NMR. The nitrogen protons were assigned by the 2D ^{1}H-^{15}N HSQC and HMBC spectra. The relative stereochemistry was determined by an observed NOE between the amide (C12(O)-N-H) proton and the methyl (C7-H_3) protons in the 2D ^{1}H-^{1}H NOESY spectrum (please see NMR spectra) and ultimately confirmed by the X-ray crystal structure.

Characterisation data for **5a'**:
TLC: R_f = 0.16 (98:1:1 ethyl acetate/methanol/triethylamine), UV.
mp = 196.5 – 199.5 °C.
IR (ATR) $\tilde{\upsilon}_{\max}$ cm^{-1}: 3461 (w), 3425 (m), 3346 (m), 3272 (br, m), 3208 (m), 3058 (w), 2993 (w), 2968 (w), 2928 (m), 2859 (m), 1632 (s), 1611 (s), 1510 (m), 1486 (m), 1426 (w), 1386 (w), 1355 (w), 1332 (w), 1270 (w), 1153 (w), 1034 (w), 1001 (w), 981 (w), 936 (w), 751 (w).
^{1}H NMR (800 MHz, DMSO-d_6, 333 K) δ ppm: 7.51 (dd, *J* = 7.7, 0.9 Hz, 1H, C18-H), 7.17 – 7.08 (m, 2H, C16-H + C12(O)-N-H), 6.84 (d, *J* = 8.2 Hz, 1H, C15-H), 6.50 (t, *J* = 7.4 Hz, 1H, C17-H), 6.31 (s, 1H, C14-N-H), 4.12 (d, *J* = 2.7 Hz, 1H, O-H), 3.83 – 3.80 (m, 1H, C10-H), 2.25 (qd, *J* = 13.5, 4.4 Hz, 1H, C4-H), 1.84 (td, *J* = 13.3, 4.4 Hz, 2H, C6-H_2), 1.78 – 1.70 (m, 3H, C3-H + C8-H + C9-H), 1.67 (tt, *J* = 13.3, 4.4 Hz, 1H, C11-H), 1.63 – 1.52 (m, 2H, C5-H + C9-H), 1.46 – 1.41 (m, 2H, C5-H + C11-H), 1.33 – 1.29 (m, 1H; C4-H), 1.22 – 1.17 (m, 1H, C8-H), 0.95 (s, 3H, C7-H_3).
^{13}C NMR (201 MHz, DMSO-d_6, 333 K) δ ppm: 162.5 (C12), 147.2 (C14), 132.9 (C16), 126.5 (C18), 115.0 (C17), 113.2 (C15), 112.6 (C13), 74.0 (C1), 63.7 (C10), 43.7 (C2), 35.4 (C3), 34.8 (C6), 33.5 (C11), 28.8 (C9), 28.5 (C4), 19.9 (C5), 19.8 (C8), 18.1 (C7).
^{15}N NMR (41 MHz, DMSO-d_6) δ ppm: 120.7 (C12(O)-NH), 76.1 (C14-NH).
LC-LRMS (ESI+) *m/z*: 301.06 found for $[M+H]^+$, 301.19 calcd. for $C_{18}H_{25}N_2O_2^+$, R_t = 1.29 min, purity (UV area): 94%.
HRMS (ESI+) *m/z*: 323.1728 found for $[M+Na]^+$, 323.1730 calcd. for $C_{18}H_{24}N_2NaO_2^+$ (Δ = -0.68 ppm).

Note: LC-LRMS indicates a purity of 94% (please see LC-LRMS trace) and unknown impurities are observed in the 1D ^{13}C NMR spectrum. Peak broadening in the 1D ^{13}C NMR spectrum observed which is resolved by VT NMR. The nitrogen protons were assigned by the 2D ^{1}H-^{15}N HSQC and HMBC spectra. The relative stereochemistry was determined by an observed NOE between the amine (C14-N-H) proton (δ_H = 6.45 ppm at 400 MHz, rt) and the methyl (C7-H_3) protons in the 2D ^{1}H-^{1}H NOESY spectrum (please see NMR spectra).

(1*S,4a*R**,6*R**,8a*S**)-7'-Fluoro-6-hydroxy-8a-methyl-3,4,4a,5,6,7,8,8a-octahydro-1'*H*,2*H*-spiro[naphthalene-1,2'-quinazolin]-4'(3'*H*)-one (5b)**

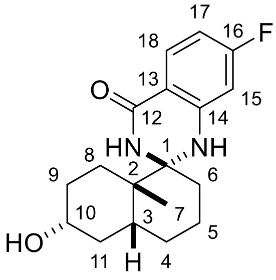

5b

(1*R,4a*R**,6*R**,8a*S**)-7'-Fluoro-6-hydroxy-8a-methyl-3,4,4a,5,6,7,8,8a-octahydro-1'*H*,2*H*-spiro[naphthalene-1,2'-quinazolin]-4'(3'*H*)-one (5b')**

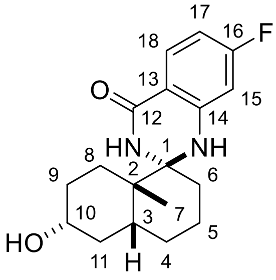

5b'

Following general procedure B using compound **1** (150 mg, 0.823 mmol) and 4-fluoroanthranilamide (178 mg, 1.16 mmol), adding additional 4-fluoroanthranilamide (0.3 equiv.) after 23.5 hours yielded a reaction mixture with a *dr* = 1.6:1 (**5b**:**5b'**), and purifying twice eluting with 1. 83:16:0:1 to 99:0:0:1 to 97:0:2:1 ethyl acetate/dichloromethane/methanol/triethylamine and 2. 99:0:1 to 97:2:1 ethyl acetate/methanol/triethylamine afforded compound **5b** (78.0 mg, 30% yield) as a white solid and **5b'** (49.1 mg, 19% yield) as a cream-white solid in separate fractions. Combined yield (including mixed fractions): 161 mg, 62%.

Note: The *dr* was determined by the crude 1D ^{1}H NMR spectrum ratio of the **5b** and **5b'** methyl (C7-H_3) protons integrals at 1.01 and 0.93 ppm, respectively. A clean mixture of diastereoisomers (34.0 mg, 13%) with a *dr* = 1:2.9 (**5b**:**5b'**) was isolated in separate mixed fractions.

Characterisation data for **5b**:
TLC: R_f = 0.16 (83:16:1 ethyl acetate/dichloromethane/triethylamine), UV.
mp = 215.5 – 218.5 °C.
IR (ATR) $\tilde{\upsilon}_{\max}$ cm^{-1}: 3400 (s), 3296 (br, m), 3103 (w), 3067 (w), 3032 (w), 2985 (w), 2926 (m), 2906 (m), 2872 (m), 2856 (m), 1659 (s), 1622 (s), 1536 (m), 1494 (m), 1441 (m), 1409 (m), 1365 (m), 1333 (w), 1295 (m), 1281 (m), 1241 (m), 1208 (w), 1177 (m), 1163 (m), 1145 (m), 1111 (m), 1093 (w), 1064 (m), 994 (m), 973 (m), 931 (w), 892 (w), 814 (m), 756 (w), 684 (w), 568 (w), 528 (m).
^{1}H NMR (600 MHz, DMSO-*d*$_6$, 373 K) δ ppm: 7.60 (dd, *J* = 8.6, 6.8 Hz, 1H, C18-H), 7.02 (s, 1H, C12(O)-N-H), 6.55 (dd, *J* = 11.5, 2.5 Hz, 1H, C15-H), 6.30 (td, *J* = 8.7, 2.5 Hz, 1H, C17-H), 6.13 (s, 1H, C14-N-H), 3.89 (s, 1H, O-H), 3.86 – 3.81 (m, 1H, C10-H), 2.21 (qd, *J* = 12.7, 4.6 Hz, 1H, C4-H), 1.96 – 1.83 (m, 2H, C3-H + C9-H), 1.81 – 1.62 (m, 5H, C6-H_2 + C8-H + C9-H + C11-H), 1.61 – 1.43 (m, 3H, C5-H_2 + C11-H), 1.40 – 1.33 (m, 1H, C4-H), 1.25 (dt, *J* = 13.1, 3.5 Hz, 1H, C8-H), 1.01 (s, 3H, C7-H_3).
^{13}C NMR (151 MHz, DMSO-*d*$_6$, 373 K) δ ppm: 165.3 (d, *J* = 246.2 Hz, C16), 161.2 (C12), 148.7 (d, *J* = 13.2 Hz, C14), 129.1 (d, *J* = 11.5 Hz, C18), 109.4 (d, *J* = 1.6 Hz, C13), 102.5 (d, *J* = 23.0 Hz, C17), 99.2 (d, *J* = 25.5 Hz, C15), 74.3 (C1), 63.8 (C10), 42.2 (C2), 35.5 (C3), 34.3 (C6), 33.5 (C11), 28.7 (C9), 28.2 (C4), 20.8 (C8), 19.2 (C5), 18.2 (C7).
^{15}N NMR (41 MHz, DMSO-*d*$_6$) δ ppm: 118.1 (C12(O)-NH), 84.2 (C14-NH).
^{19}F NMR (377 MHz, DMSO-*d*$_6$) δ ppm: -107.5.
LC-LRMS (ESI+) *m/z*: 319.15 found for $[M+H]^+$, 319.18 calcd. for $C_{18}H_{24}FN_2O_2^+$, R_t = 1.20 min, purity (UV area): >98%.
HRMS (ESI+) *m/z*: 341.1636 found for $[M+Na]^+$, 341.1636 calcd. for $C_{18}H_{23}FN_2NaO_2^+$ (Δ = 0.00 ppm).

Note: Peak broadening in the 1D ^{13}C NMR spectrum observed which is resolved by VT NMR. The nitrogen protons were assigned by the 2D ^{1}H-^{15}N HSQC and HMBC spectra. The relative stereochemistry was determined by an observed NOE between the amide (C12(O)-N-H) proton (δ_H = 7.66 ppm at 400 MHz, rt) and the methyl (C7-H_3) protons in the 2D ^{1}H-^{1}H NOESY spectrum (please see NMR spectra).

Characterisation data for **5b'**:
TLC: R_f = 0.17 (97:2:1 ethyl acetate/methanol/triethylamine), UV.
mp = 205.0 – 208.0 °C.
IR (ATR) $\tilde{\upsilon}_{\max}$ cm^{-1}: 3419 (m), 3323 (br, m), 3188 (m), 3078 (w), 3044 (w), 2928 (m), 2866 (m), 1722 (w), 1642 (s), 1613 (s), 1527 (m), 1490 (m), 1429 (m), 1371 (m), 1334 (w), 1292 (m), 1168 (m), 1146 (m), 1089 (w), 1053 (w), 1009 (m), 934 (w), 842 (w), 763 (w).
^{1}H NMR (800 MHz, DMSO-*d*$_6$, 333 K) δ ppm: 7.54 (dd, *J* = 8.6, 6.8 Hz, 1H, C18-H), 7.23 (s, 1H, C12(O)-N-H), 6.67 – 6.60 (m, 2H, C15-H + C14-N-H), 6.27 (td, *J* = 8.7, 2.5 Hz, 1H, C17-H), 4.13 (d, *J* = 2.7 Hz, 1H, O-H), 3.84 – 3.80 (m, 1H, C10-H), 2.30 – 2.22 (m, 1H, C4-H), 1.89 – 1.80 (m, 2H, C6-H_2), 1.76 – 1.70 (m, 3H, C3-H + C8-H + C11-H), 1.66 (tt, *J* = 13.3, 4.4 Hz, 1H, C9-H), 1.63 – 1.58 (m, 1H, C9-H), 1.54 (qt, *J* = 13.6, 4.0 Hz, 1H, C5-H), 1.49 – 1.41 (m, 2H, C5-H + C11-H), 1.34 – 1.29 (m, 1H, C4-H), 1.18 (dt, *J* = 13.2, 4.2 Hz, 1H, C8-H), 0.93 (s, 3H, C7-H_3).
^{13}C NMR (201 MHz, DMSO-*d*$_6$, 333 K) δ ppm: 165.6 (d, *J* = 245.8 Hz, C16), 161.7 (C12), 149.0 (d, *J* = 13.2 Hz, C14), 129.3 (d, *J* = 11.6 Hz, C18), 109.4 (d, *J* = 1.5 Hz, C13), 102.3 (d, *J* = 22.9 Hz, C17), 98.6 (d, *J* = 25.7 Hz, C15), 74.4 (C1), 63.6 (C10), 44.0 (C2), 35.4 (C3), 34.9 (C6), 33.4 (C11), 28.7 (C4), 28.5 (C9), 19.9 (C5), 19.8 (C8), 17.9 (C7).
^{15}N NMR (41 MHz, DMSO-*d*$_6$) δ ppm: 120.1 (C12(O)-NH), 79.8 (C14-NH).
^{19}F NMR (377 MHz, DMSO-*d*$_6$) δ ppm: -107.4.
LC-LRMS (ESI+) *m/z*: 319.11 found for $[M+H]^+$, 319.18 calcd. for $C_{18}H_{24}FN_2O_2^+$, R_t = 1.26 min, purity (UV area): 92%.
HRMS (ESI+) *m/z*: 341.1636 found for $[M+Na]^+$, 341.1636 calcd. for $C_{18}H_{23}FN_2NaO_2^+$ (Δ = 0.00 ppm).

Note: LC-LRMS indicates a purity of 92% (please see LC-LRMS trace) and unknown impurities are observed in the 1D ^{13}C NMR spectrum. Peak broadening in the 1D ^{13}C NMR spectrum observed which is resolved by VT NMR. The nitrogen protons were assigned by the 2D ^{1}H-^{15}N HSQC and HMBC spectra. The relative stereochemistry was determined by an observed NOE between the amine (C14-N-H) proton (δ_H = 6.76 ppm at 400 MHz, rt) and the methyl (C7-H_3) protons in the 2D ^{1}H-^{1}H NOESY spectrum (please see NMR spectra).

(1*S,4a*R**,6*R**,8a*S**)-6-Hydroxy-6'-methoxy-8a-methyl-3,4,4a,5,6,7,8,8a-octahydro-1'*H*,2*H*-spiro[naphthalene-1,2'-quinazolin]-4'(3'*H*)-one (5c)**

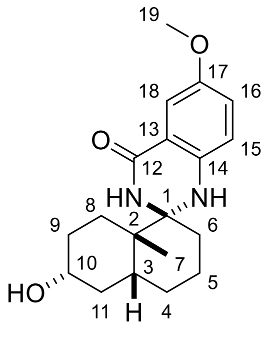

5c

(1*R,4a*R**,6*R**,8a*S**)-6-Hydroxy-6'-methoxy-8a-methyl-3,4,4a,5,6,7,8,8a-octahydro-1'*H*,2*H*-spiro[naphthalene-1,2'-quinazolin]-4'(3'*H*)-one (5c')**

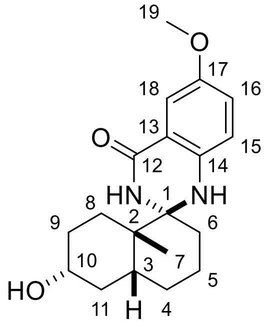

5c'

Following general procedure B using compound **1** (150 mg, 0.823 mmol) and 5-methoxyanthranilamide (192.0 mg, 1.16 mmol), adding additional 5-methoxyanthranilamide (0.3 equiv.) after 23.5 hours yielded a reaction mixture with a *dr* = 1.3:1 (**5c**:**5c'**), and purifying thrice eluting with 1. 99:0:1 to 97:2:1 ethyl acetate/methanol/triethylamine, 2. 79:20:1 to 99:0:1 ethyl acetate/dichloromethane/triethylamine, and 3. 99:0:1 to 97:2:1 ethyl acetate/methanol/triethylamine afforded compound **5c** (86.1 mg, 32% yield) as a pale-yellow solid and **5c'** (80.3 mg, 30% yield) as a yellow solid in separate fractions. Combined yield (including mixed fractions): 181 mg, 67%.

Note: The *dr* was determined by the crude 1D ^{1}H NMR spectrum ratio of the **5c** and **5c'** methyl (C7-H_3) protons integrals at 1.04 and 0.96 ppm, respectively. A clean mixture of diastereoisomers (14.3 mg, 5%) with a *dr* = 1:1 (**5c**:**5c'**) was isolated in separate mixed fractions.

Characterisation data for **5c**:
TLC: R_f = 0.17 (99:1 ethyl acetate/triethylamine), UV.
dec pt = 170.0 – 173.0 °C.
IR (ATR) $\tilde{\upsilon}_{max}$ cm^{-1}: 3513 (m), 3307 (br, m), 3075 (w), 3034 (w), 2916 (m), 2963 (m), 2837 (m), 1650 (s), 1615 (s), 1590 (m), 1498 (s), 1468 (m), 1433 (m), 1408 (m), 1371 (m), 1337 (m), 1262 (m), 1214 (m), 1188 (m), 1171 (w), 1140 (m), 1119, (w), 1084 (w), 1041 (w), 1027 (w), 999 (m), 892 (w), 874 (w), 819 (w), 781 (w), 704 (w), 661 (w), 576 (w), 551 (w), 516 (w).
^{1}H NMR (600 MHz, DMSO-*d*$_6$, 373 K) δ ppm: 7.15 (d, *J* = 3.0 Hz, 1H, C18-H), 6.96 (s, 1H, C12(O)-N-H), 6.86 (dd, *J* = 8.8, 3.1 Hz, 1H, C16-H), 6.76 (d, *J* = 8.8 Hz, 1H, C15-H), 5.39 (s, 1H, C14-N-H), 3.89 – 3.81 (m, 2H, C10-H + O-H), 3.69 (s, 3H, C19-H_3), 2.25 – 2.15 (m, 1H, C4-H), 1.95 – 1.87 (m, 2H, C3-H + C6-H), 1.80 – 1.63 (m, 5H, C6-H + C8-H + C9-H_2 + C11-H), 1.56 – 1.43 (m, 3H, C5-H_2 + C11-H), 1.39 – 1.33 (m, 1H, C4-H), 1.28 – 1.23 (m, 1H, C8-H), 1.04 (s, 3H, C7-H_3).
^{13}C NMR (151 MHz, DMSO-*d*$_6$, 373 K) δ ppm: 161.9 (C12), 150.4 (C17), 141.3 (C14), 120.9 (C16-H), 115.4 (C15-H), 113.2 (C13), 110.0 (C18), 73.9 (C1), 63.8 (C10), 55.2 (C19), 41.6 (C2), 35.6 (C3), 33.61 (C6/C11), 33.56 (C6/C11), 28.7 (C9), 28.3 (C4), 20.7 (C8), 19.4 (C5), 18.2 (C7).
^{15}N NMR (41 MHz, DMSO-*d*$_6$) δ ppm: 119.5 (C12(O)-NH), 76.1 (C14-NH).
LC-LRMS (ESI+) *m/z*: 331.09 found for $[M+H]^+$, 331.20 calcd. for $C_{19}H_{27}N_2O_3^+$, R_t = 1.06 min, purity (UV area): ≥95%.
HRMS (ESI+) *m/z*: 331.2018 found for $[M+H]^+$, 331.2016 calcd. for $C_{19}H_{27}N_2O_3^+$ (Δ = 0.67 ppm).

Note: Peak broadening in the 1D ^{13}C NMR spectrum observed which is resolved by VT NMR. The nitrogen protons were assigned by the 2D ^{1}H-^{15}N HSQC and HMBC spectra. The relative stereochemistry was determined by an observed NOE between the amide (C12(O)-N-H) proton (δ_H = 7.62 ppm at 400 MHz, rt) and the methyl (C7-H_3) protons in the 2D ^{1}H-^{1}H NOESY spectrum (please see NMR spectra).

Characterisation data for **5c'**:
TLC: R_f = 0.16 (97:2:1 ethyl acetate/methanol/triethylamine), UV.
dec pt = 113.0 – 116.0 °C.
IR (ATR) $\tilde{\upsilon}_{max}$ cm^{-1}: 3339 (br, m), 3072 (w), 3043 (w), 2924 (m), 2866 (m), 1724 (w), 1643 (s), 1619 (s), 1590 (m), 1498 (s), 1437 (m), 1369 (m), 1263 (m), 1246 (m), 1219 (m), 1179 (w), 1146 (w), 1087 (w), 1034 (m), 999 (m), 820 (w), 781 (w), 706 (w), 618 (w), 572 (w).
^{1}H NMR (800 MHz, DMSO-*d*$_6$, 333 K) δ ppm: 7.12 (s, 1H, C12(O)-N-H), 7.08 (d, *J* = 3.1 Hz, 1H, C18-H), 6.85 (dd, *J* = 8.7, 3.0 Hz, 1H, C16-H), 6.81 (d, *J* = 8.8 Hz, 1H, C15-H), 6.01 (s, 1H, C14-N-H), 4.12

(d, J = 2.0 Hz, 1H, O-H), 3.84 – 3.81 (m, 1H, C10-H), 3.67 (s, 3H, C19-H_3), 2.25 (qd, J = 13.5, 4.4 Hz, 1H, C4-H), 1.86 – 1.72 (m, 5H, C3-H + C6-H_2 + C8-H + C11-H), 1.68 (tt, J = 13.4, 4.3 Hz, 1H, C9-H), 1.63 – 1.59 (m, 1H, C9-H), 1.53 (qt, J = 13.5, 4.1 Hz, 1H, C5-H), 1.45 – 1.40 (m, 2H, C5-H + C11-H), 1.33 – 1.29 (m, 1H, C4-H), 1.21 (dt, J = 12.9, 3.8 Hz, 1H, C8-H), 0.96 (s, 3H, C7-H_3).
^{13}C NMR (201 MHz, DMSO-d_6, 333 K) δ ppm: 162.6 (C12), 150.0 (C17), 141.7 (C14), 121.3 (C16), 114.7 (C15), 112.9 (C13), 109.6 (C18), 74.0 (C1), 63.7 (C10), 55.2 (C19), 43.4 (C2), 35.4 (C3), 34.3 (C6), 33.5 (C11), 28.8 (C9), 28.5 (C4), 20.0 (C5), 19.9 (C8), 18.2 (C7).
^{15}N NMR (41 MHz, DMSO-d_6) δ ppm: 121.3 (C12(O)-NH), 70.8 (C14-NH).
LC-LRMS (ESI+) *m/z*: 331.12 found for $[M+H]^+$, 331.20 calcd. for $C_{19}H_{27}N_2O_3^+$, R_t = 1.09 min, purity (UV area): 92%.
HRMS (ESI+) *m/z*: 331.2021 found for $[M+H]^+$, 331.2016 calcd. for $C_{19}H_{27}N_2O_3^+$ (Δ = 1.58 ppm).

Note: LC-LRMS indicates a purity of 92% (please see LC-LRMS trace) and unknown impurities are observed in the 1D ^{1}H and ^{13}C NMR spectra. Peak broadening in the 1D ^{13}C NMR spectrum observed which is resolved by VT NMR. The nitrogen protons were assigned by the 2D ^{1}H-^{15}N HSQC and HMBC spectra. The relative stereochemistry was determined by an observed NOE between the amine (C14-N-H) proton (δ_H = 6.14 ppm at 400 MHz, rt) and the methyl (C7-H_3) protons in the 2D ^{1}H-^{1}H NOESY spectrum (please see NMR spectra).

Synthesis of quinoxaline analogues

General procedure C

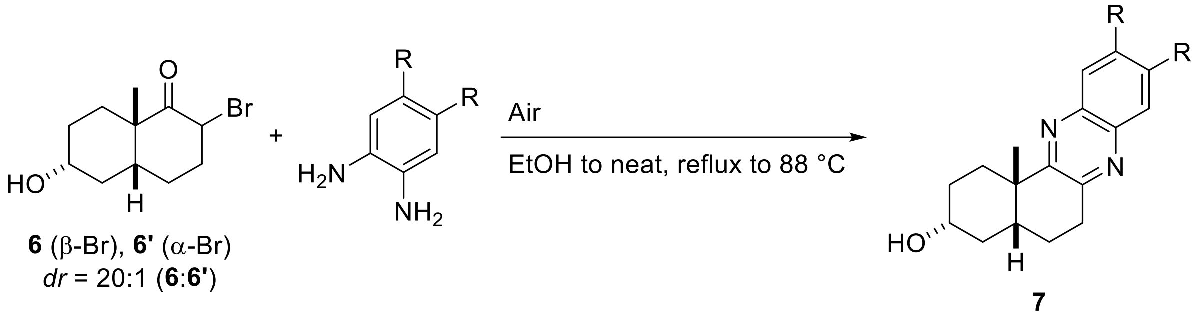

6 (β-Br), **6'** (α-Br)
dr = 20:1 (**6**:**6'**)

7

A solution of a diastereoisomeric mixture of compound **6** and **6’** (1.00 equiv.) with a *dr* = 20:1 (**6**:**6’**) and the appropriate *o*-phenylenediamine (1.05 equiv.) in absolute ethanol (0.15 M **6**/**6’**) was stirred at reflux, slowly concentrated, and heated neat at 88 °C under an ambient air atmosphere during 24 hours. The residue was redissolved in absolute ethanol (0.15 M **6**/**6’**) and the 24-hour refluxing-concentrating-heating-redissolving cycle was repeated the indicated times. The reaction mixture was cooled to room temperature and the solvent removed under reduced pressure to yield the crude products. The crude products were purified by flash column chromatography on silica gel to afford the quinoxaline analogues (**7**).

(3*R,4a*R**,12b*S**)-12b-Methyl-1,2,3,4,4a,5,6,12b-octahydrobenzo[*a*]phenazin-3-ol (7a)**

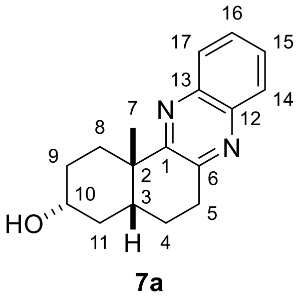

7a

Following general procedure C using compound **6**/**6’** (79.2 mg, 0.303 mmol) and *o*-phenylenediamine (34.4 mg, 0.318 mmol), stirring for 48 hours (two cycles), and eluting with 19:1 dichloromethane/methanol afforded compound **7a** (28.3 mg, 35% yield) as a brown sticky solid.

TLC: R_f = 0.15 (19:1 dichloromethane/methanol), UV.

IR (ATR) $\tilde{\upsilon}_{max}$ cm^{-1}: 3338 (br, m), 2926 (s), 2867 (s), 2224 (w), 1701 (w), 1667 (w), 1627 (w), 1561 (w), 1504 (w), 1483 (m), 1452 (m), 1433 (m), 1393 (w), 1346 (m), 1309 (w), 1293 (m), 1253 (w), 1222 (w), 1199 (m), 1128 (m), 1097 (m), 1082 (m), 1065 (m), 1040 (m), 1020 (w), 1008 (w), 953 (w), 921 (w), 904 (w), 869 (w), 831 (w), 760 (m), 728 (m), 646 (w), 610 (w).
^{1}H NMR (400 MHz, $CDCl_3$) δ ppm: 8.04 – 7.96 (m, 2H, C14-H + C17-H), 7.71 – 7.63 (m, 2H, C15-H + C16-H), 3.79 (tt, *J* = 10.9, 4.3 Hz, 1H, C10-H), 3.33 – 3.17 (m, 2H, C5-H_2), 3.10 (dt, *J* = 13.6, 3.6 Hz, 1H, C8-H), 2.50 – 2.39 (m, 1H, C4-H), 2.01 – 1.79 (m, 4H, C3-H + C4-H + C9-H + C11-H), 1.43 (td, *J* = 13.5, 3.5 Hz, 1H, C8-H), 1.32 – 1.17 (m, 4H, C7-H_3 + C11-H), 1.04 (tdd, *J* = 13.3, 10.9, 3.6 Hz, 1H, C9-H).
^{13}C NMR (101 MHz, $CDCl_3$) δ ppm: 158.5 (C1), 152.8 (C6), 141.9 (C13), 140.6 (C12), 129.3 (C14), 129.0 (C16), 128.9 (C15), 128.0 (C17), 70.9 (C10), 41.2 (C2), 39.7 (C3), 37.7 (C11), 34.8 (C8), 32.7 (C9), 31.0 (C7), 28.7 (C5), 23.2 (C4).
LC-LRMS (ESI+) *m/z*: 269.45 found for $[M+H]^+$, 269.16 calcd. for $C_{17}H_{21}N_2O^+$, R_t = 1.35 min, purity (UV area): >98%.
HRMS (ESI+) *m/z*: 269.1650 found for $[M+H]^+$, 269.1648 calcd. for $C_{17}H_{21}N_2O^+$ (Δ = 0.46 ppm).

(3*R,4a*R**,12b*S**)-9,10,12b-Trimethyl-1,2,3,4,4a,5,6,12b-octahydrobenzo[*a*]phenazin-3-ol (7b)**

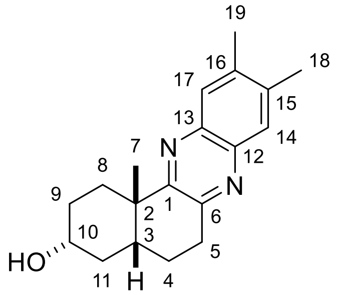

7b

Following general procedure C using compound **6**/**6'** (101 mg, 0.387 mmol) and 4,5-dimethyl-1,2-phenylenediamine (55.3 mg, 0.406 mmol), stirring for 24 hours (one cycle), and eluting with 94:5:1 dichloromethane/methanol/triethylamine afforded compound **7b** (34.0 mg, 30% yield) as a reddish-brown solid.

TLC: R_f = 0.28 (94:5:1 dichloromethane/methanol/triethylamine), UV.
mp = 55.0 – 60.0 °C.
IR (ATR) $\tilde{\upsilon}_{max}$ cm^{-1}: 3300 (br, m), 3078 (w), 3044 (w), 2925 (s), 2864 (s), 1628 (w), 1557 (w), 1520 (w), 1473 (m), 1454 (m), 1432 (m), 1348 (m), 1304 (w), 1257 (w), 1223 (w), 1202 (w), 1172 (w), 1122 (w), 1100 (m), 1070 (m), 1042 (m), 1023 (w), 996 (w), 968 (w), 952 (w), 923 (w), 904 (w), 869 (m), 825 (w), 719 (w), 647 (w), 607 (w).
^{1}H NMR (400 MHz, DMSO-d_6) δ ppm: 7.77 (s, 1H, C17-H), 7.70 (s, 1H, C14-H), 4.36 (d, *J* = 5.0 Hz, 1H, O-H), 3.59 – 3.46 (m, 1H, C10-H), 3.17 – 3.01 (m, 2H, C5-H_2), 2.86 (dt, *J* = 13.3, 3.5 Hz, 1H, C8-H), 2.43 (s, 6H, C18-H_3 + C19-H_3), 2.39 – 2.26 (m, 1H, C4-H), 1.88 (dq, *J* = 12.9, 3.6 Hz, 1H, C3-H), 1.77 – 1.60 (m, 3H, C4-H + C9-H + C11-H), 1.40 – 1.30 (m, 1H, C8-H), 1.19 (s, 3H, C7-H_3), 1.04 (td, *J* = 12.9, 10.7 Hz, 1H, C11-H), 0.83 – 0.71 (m, 1H, C9-H).
^{13}C NMR (101 MHz, DMSO-d_6) δ ppm: 157.1 (C1), 151.9 (C6), 139.7 (C13), 139.4 (C12), 139.2 (C15), 138.9 (C16), 127.4 (C17), 126.8 (C14), 68.8 (C10), 40.6 (C2), 38.9 (C3), 37.5 (C11), 34.4 (C8), 32.5 (C9), 30.8 (C7), 28.1 (C5), 22.6 (C4), 19.8 (C18), 19.6 (C19).
LC-LRMS (ESI+) *m/z*: 297.40 found for $[M+H]^+$, 297.20 calcd. for $C_{19}H_{25}N_2O^+$, R_t = 1.57 min, purity (UV area): 99%.
HRMS (ESI+) *m/z*: 297.1966 found for $[M+H]^+$, 297.1961 calcd. for $C_{19}H_{25}N_2O^+$ (Δ = 1.56 ppm).

Note: The peak for the C3 carbon at 38.9 ppm in the 1D ^{13}C NMR spectrum overlaps with the dimethyl sulfoxide-d_6 solvent peak but the chemical shift was confirmed by correlations in the 2D ^{1}H-^{13}C HSQC and HMBC spectra.

(3*R,4a*R**,12b*S**)-9,10-Difluoro-12b-methyl-1,2,3,4,4a,5,6,12b-octahydrobenzo[*a*]phenazin-3-ol (7c)**

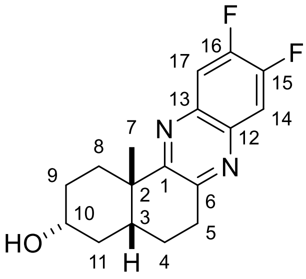

7c

Following general procedure C using compound **6**/**6'** (100 mg, 0.383 mmol) and 1,2-diamino-4,5-difluorobenzene (58.3 mg, 0.405 mmol), stirring for three days (three cycles), and purifying twice eluting with 1. 97:3 dichloromethane/methanol and 2. 197:2:1 to 196:3:1 dichloromethane/methanol/acetic acid afforded compound **7c** (46.3 mg, 40% yield) as a pale-brown solid.

TLC: R_f = 0.24 (196:3:1 dichloromethane/methanol/acetic acid), UV.
mp = 158.0 – 161.0 °C.
IR (ATR) $\tilde{\upsilon}_{\max}$ cm^{-1}: 3356 (br, m), 3041 (w), 2933 (m), 2891 (m), 2874 (m), 1704 (w), 1633 (w), 1573 (w), 1495 (s), 1471 (m), 1453 (m), 1436 (m), 1416 (w), 1380 (w), 1364 (m), 1347 (w), 1347 (w), 1305 (m), 1276 (w), 1255 (w), 1233 (s), 1217 (m), 1203 (m), 1170 (w), 1156 (w), 1147 (w), 1124 (w), 1096 (w), 1070 (m), 1040 (m), 1011 (m), 970 (w), 952 (w), 885 (m), 869 (m), 857 (m), 828 (w), 784 (w), 769 (w), 750 (w), 627 (w), 586 (w).
^{1}H NMR (400 MHz, $CDCl_3$) δ ppm: 7.78 – 7.66 (m, 2H, C14-H + C17-H), 3.79 (tt, *J* = 10.8, 4.3 Hz, 1H, C10-H), 3.28 – 3.11 (m, 2H, C5-H_2), 3.03 (dt, *J* = 13.7, 3.6 Hz, 1H, C8-H), 2.48 – 2.37 (m, 1H, C4-H), 2.01 – 1.79 (m, 4H, C3-H + C4-H + C9-H + C11-H), 1.43 (td, *J* = 13.6, 3.5 Hz, 1H, C8-H), 1.29 – 1.15 (m, 4H, C7-H_3 + C11-H), 1.04 – 0.93 (m, 1H, C9-H).
^{13}C NMR (101 MHz, $CDCl_3$) δ ppm: 158.8 (d, *J* = 3.3 Hz, C1), 153.4 (d, *J* = 2.9 Hz, C6), 152.1 (dd, *J* = 254.0, 15.4 Hz, C16), 151.8 (dd, *J* = 254.0, 15.6 Hz, C15), 138.9 (d, *J* = 11.1 Hz, C13), 138.1 (d, *J* = 10.9 Hz, C12), 114.6 (dd, *J* = 16.9, 1.9 Hz, C17), 113.8 (dd, *J* = 17.0, 1.9 Hz, C14), 70.8 (C10), 41.2 (C2), 39.6 (C3), 37.6 (C11), 34.7 (C8), 32.7 (C9), 31.0 (C7), 28.6 (C5), 23.1 (C4).
^{19}F NMR (377 MHz, $CDCl_3$) δ ppm: -131.6 (d, *J* = 21.1 Hz, C16-F), -132.3 (d, *J* = 20.9 Hz, C15-F).
LC-LRMS (ESI+) *m/z*: 305.02 found for $[M+H]^+$, 305.15 calcd. for $C_{17}H_{19}F_2N_2O^+$, R_t = 1.63 min, purity (UV area): 95%.
HRMS (ESI+) *m/z*: 305.1460 found for $[M+H]^+$, 305.1460 calcd. for $C_{17}H_{19}F_2N_2O^+$ (Δ = 0.00 ppm).

Note: The fluorines were assigned by the 2D ^{19}F-^{13}C HMQC spectrum (please see NMR spectra).

(3*R,4a*R**,12b*S**)-9,10-Dichloro-12b-methyl-1,2,3,4,4a,5,6,12b-octahydrobenzo[*a*]phenazin-3-ol (7d)**

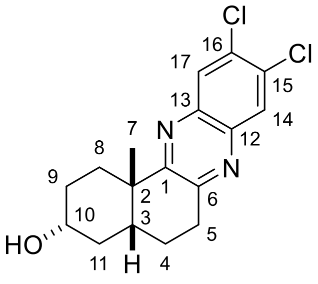

7d

Following general procedure C using compound **6**/**6'** (82.1 mg, 0.314 mmol) and 1,2-diamino-4,5-dichlorobenzene (58.4 mg, 0.330 mmol), stirring for five days (five cycles), and purifying twice eluting with 1. 90:9:1 dichloromethane/ethyl acetate/triethylamine and 2. 1:0 to 3:2 *n*-heptane/ethyl acetate afforded compound **7d** (24.7 mg, 30% yield) as a light-pink solid.

TLC: R_f = 0.43 (3:2 *n*-heptane/ethyl acetate), UV.

mp = 177.1 °C.
IR (ATR) $\tilde{\upsilon}_{\max}$ cm^{-1}: 3249 (br, s), 3088 (m), 2969 (m), 2949 (m), 2922 (s), 2892 (m), 2855 (m), 1738 (w), 1700 (w), 1595 (w), 1557 (w), 1446 (s), 1416 (m), 1391 (w), 1366 (m), 1343 (w), 1322 (w), 1300 (m), 1272 (w), 1251 (w), 1168 (s), 1123 (w), 1107 (m), 1081 (w), 1073 (m), 1053 (w), 1041 (m), 1010 (w), 979 (w), 949 (w), 888 (m), 838 (w), 715 (w), 671 (w), 557 (w).
^{1}H NMR (400 MHz, $CDCl_3$) δ ppm: 8.15 (s, 1H, C17-H), 8.10 (s, 1H, C14-H), 3.79 (tt, *J* = 10.8, 4.3 Hz, 1H, C10-H), 3.30 – 3.14 (m, 2H, C5-H_2), 3.02 (dt, *J* = 13.7, 3.6 Hz, 1H, C8-H), 2.48 – 2.37 (m, 1H, C4-H), 2.02 – 1.80 (m, 4H, C3-H + C4-H + C9-H + C11-H), 1.43 (td, *J* = 13.6, 3.6 Hz, 1H, C8-H), 1.29 – 1.14 (m, 4H, C7-H_3 + C11-H), 1.05 – 0.93 (m, 1H, C9-H).
^{13}C NMR (101 MHz, $CDCl_3$) δ ppm: 160.0 (C1), 154.4 (C6), 140.6 (C13), 139.5 (C12), 133.7 (C16), 133.4 (C15), 129.7 (C17), 128.8 (C14), 70.7 (C10), 41.5 (C2), 39.5 (C3), 37.7 (C11), 34.6 (C8), 32.7 (C9), 31.0 (C7), 28.7 (C5), 23.0 (C4).
LC-LRMS (ESI+) *m/z*: 337.05 found for $[M+H]^+$, 337.09 calcd. for $C_{17}H_{19}Cl_2N_2O^+$, R_t = 1.88 min, purity (UV area): 97%.
HRMS (ESI+) *m/z*: 337.0880 found for $[M+H]^+$, 337.0869 calcd. for $C_{17}H_{19}Cl_2N_2O^+$ (Δ = 3.33 ppm).

(3*R,4a*R**,12b*S**)-9,10-Dibromo-12b-methyl-1,2,3,4,4^a,5,6,12b-octahydrobenzo[*a*]phenazin-3-ol (7e)**

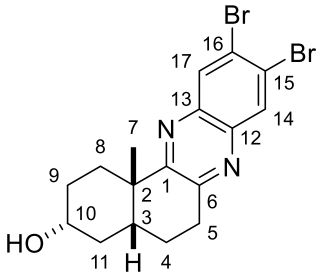

7e

Following general procedure C using compound **6**/**6'** (100 mg, 0.383 mmol) and 1,2-diamino-4,5-dibromobenzene (107.5 mg, 0.404 mmol), stirring for three days (three cycles), and eluting with 172:27:1 dichloromethane/ethyl acetate/acetic acid afforded compound **7e** (53.6 mg, 33% yield) as a reddish-brown solid.

TLC: R_f = 0.20 (172:27:1 dichloromethane/ethyl acetate/acetic acid), UV.
dec pt = 165.5 – 168.5 °C.
IR (ATR) $\tilde{\upsilon}_{\max}$ cm^{-1}: 3297 (br, m), 3077 (w), 3050 (w), 2931 (s), 2894 (m), 2874 (s), 1703 (w), 1585 (w), 1464 (w), 1440 (s), 1407 (m), 1387 (m), 1364 (m), 1341 (m), 1298 (m), 1273 (m), 1251 (m), 1163 (m), 1128 (w), 1100 (m), 1085 (m), 1068 (m), 1040 (m), 1011 (m), 970 (w), 955 (w), 941 (m), 926 (w), 892 (m), 881 (m), 837 (m), 732 (w), 653 (w).
^{1}H NMR (400 MHz, $CDCl_3$) δ ppm: 8.34 (s, 1H, C17-H), 8.28 (s, 1H, C14-H), 3.79 (tt, *J* = 10.8, 4.3 Hz, 1H, C10-H), 3.28 – 3.12 (m, 2H, C5-H_2), 3.02 (dt, *J* = 13.7, 3.6 Hz, 1H, C8-H), 2.48 – 2.37 (m, 1H, C4-H), 2.02 – 1.80 (m, 4H, C3-H + C4-H + C9-H + C11-H), 1.43 (td, *J* = 13.6, 3.6 Hz, 1H, C8-H), 1.29 – 1.14 (m, 4H, C7-H_3 + C11-H), 1.05 – 0.93 (m, 1H, C9-H).
^{13}C NMR (101 MHz, $CDCl_3$) δ ppm: 160.1 (C1), 154.6 (C6), 141.1 (C13), 140.2 (C12), 133.1 (C17), 132.3 (C14), 125.6 (C16), 125.2 (C15), 70.7 (C10), 41.5 (C2), 39.5 (C3), 37.7 (C11), 34.6 (C8), 32.7 (C9), 30.9 (C7), 28.8 (C5), 23.0 (C4).
LC-LRMS (ESI+) *m/z*: 424.83 found for $[M+H]^+$, 424.99 calcd. for $C_{17}H_{19}Br_2N_2O^+$, R_t = 2.01 min, purity (UV area): ≥95%.
HRMS (ESI+) *m/z*: 424.9860 found for $[M+H]^+$, 424.9859 calcd. for $C_{17}H_{19}Br_2N_2O^+$ (Δ = 0.29 ppm).

Synthesis of thiazole analogues

General procedure D

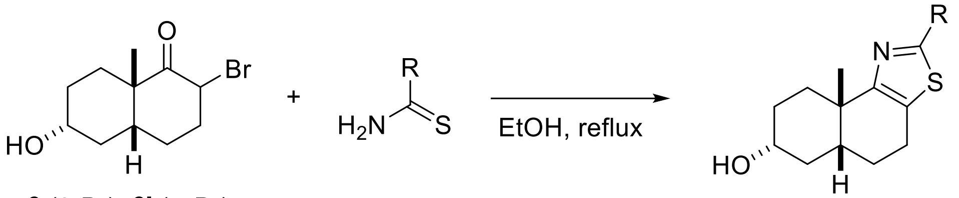

6 (β-Br), **6'** (α-Br)
dr = 20:1 (**6**:**6'**)

8

To a solution of a diastereoisomeric mixture of compound **6** and **6’** (1.0 equiv.) with a *dr* = 20:1 (**6**:**6’**) in absolute ethanol (80 mM **6**/**6’**) at room temperature was added the appropriate thioamide (1.5 equiv.). The reaction mixture was stirred at reflux for the indicated time. If necessary, additional thioamide was added during the reaction. The reaction mixture was cooled to room temperature and the solvent removed under reduced pressure to yield the crude products. The crude products were purified by flash column chromatography on silica gel or preparative HPLC to afford the thiazole-fused analogues (**8**).

(5a*R,7*R**,9a*S**)-2-Amino-9a-methyl-4,5,5a,6,7,8,9,9a-octahydronaphtho[1,2-*d*]thiazol-7-ol (8a)**

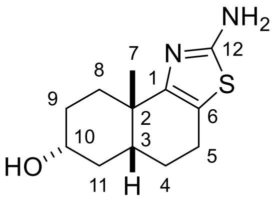

8a

Following general procedure D using compound **6**/**6’** (100 mg, 0.383 mmol) and thiourea (43.7 mg, 0.574 mmol) and refluxing for two hours yielded the initial crude product. The initial crude product was dissolved in dichloromethane, washed with water (2×), and the phases separated. The combined aqueous phases were neutralised with saturated aqueous $NaHCO_3$ and extracted with dichloromethane (5×). The combined organic phases were dried over anhydrous Na_2SO_4 and the solvent removed under reduced pressure to yield the final crude product. The final crude product was purified on silica gel eluting with 99:1 ethyl acetate/triethylamine to afford compound **8a** (53.1 mg, 58% yield) as a white solid.

TLC: R_f = 0.23 (99:1 ethyl acetate/triethylamine), UV.
dec pt = 137.0 °C.
IR (ATR) $\tilde{\upsilon}_{\max}$ cm^{-1}: 3450 (br, m), 3291 (m), 3175 (m), 3126 (m), 2936 (m), 2922 (s), 2879 (m), 2845 (s), 1621 (s), 1572 (w), 1522 (s), 1445 (m), 1365 (w), 1355 (w), 1336 (m), 1304 (m), 1290 (m), 1271 (w), 1256 (w), 1237 (w), 1164 (w), 1134 (w), 1091 (w), 1072 (m), 1035 (m), 1021 (w), 963 (w), 947 (w), 823 (w), 742 (w),
^{1}H NMR (400 MHz, CD_3OD) δ ppm: 3.58 (tt, *J* = 10.9, 4.2 Hz, 1H, C10-H), 2.63 (ddd, *J* = 16.5, 11.6, 6.4 Hz, 1H, C5-H), 2.51 (ddd, *J* = 16.6, 6.8, 1.5 Hz, 1H, C5-H), 2.39 (dt, *J* = 13.8, 3.5 Hz, 1H, C8-H), 2.23 – 2.12 (m, 1H, C4-H), 1.74 – 1.65 (m, 3H, C3-H + C9-H + C11-H), 1.64 – 1.56 (m, 1H, C4-H), 1.41 – 1.22 (m, 2H, C8-H + C11-H), 1.17 (s, 3H, C7-H_3), 1.06 – 0.94 (m, 1H, C9-H).
^{13}C NMR (101 MHz, CD_3OD) δ ppm: 169.3 (C12), 150.6 (C1), 116.9 (C6), 71.3 (C10), 41.2 (C3), 38.1 (C2), 37.2 (C11), 36.6 (C8), 33.4 (C9), 30.1 (C7), 25.5 (C4), 20.5 (C5).
LC-LRMS (ESI+) *m/z*: 239.00 found for $[M+H]^+$, 239.12 calcd. for $C_{12}H_{19}N_2OS^+$, R_t = 0.96 min, purity (UV area): ≥95%.
HRMS (ESI+) *m/z*: 239.1216 found for $[M+H]^+$, 239.1213 calcd. for $C_{12}H_{19}N_2OS^+$ (Δ = 1.35 ppm).

(5a*R,7*R**,9a*S**)-2,9a-Dimethyl-4,5,5a,6,7,8,9,9a-octahydronaphtho[1,2-*d*]thiazol-7-ol (8b)**

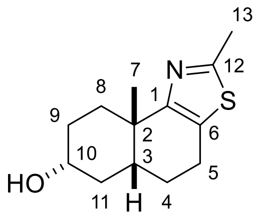

8b

Following general procedure D using compound **6**/**6'** (100 mg, 0.383 mmol) and thioacetamide (43.2 mg, 0.574 mmol), refluxing for three hours, and purifying on silica gel eluting with 1:1 dichloromethane/ethyl acetate afforded compound **8b** (17.9 mg, 20% yield) as a white solid.

TLC: R_f = 0.20 (1:1 dichloromethane/ethyl acetate), UV.
mp = 140.0 – 143.0 °C.
IR (ATR) $\tilde{\upsilon}_{max}$ cm^{-1}: 3263 (br, m), 2965 (w), 2943 (m), 2921 (s), 2850 (s), 1545 (m), 1484 (w), 1439 (m), 1362 (m), 1332 (w), 1317 (w), 1295 (w), 1231 (w), 1219 (w), 1201 (m), 1171 (w), 1159 (w), 1124 (w), 1092 (w), 1078 (m), 1056 (w), 1039 (m), 1022 (w), 1012 (w), 1000 (w), 962 (w), 943 (w), 823 (w), 733 (w), 710 (w), 672 (w), 617 (w), 603 (w).
^{1}H NMR (400 MHz, CD_3OD) δ ppm: 3.61 (tt, *J* = 11.0, 4.2 Hz, 1H, C10-H), 2.85 – 2.70 (m, 2H, C5-H_2), 2.62 (s, 3H, C13-H_3), 2.57 (dt, *J* = 13.9, 3.5 Hz, 1H, C8-H), 2.26 – 2.15 (m, 1H, C4-H), 1.81 – 1.63 (m, 4H, C3-H + C4-H + C9-H + C11-H), 1.38 (td, *J* = 13.8, 3.3 Hz, 1H, C8-H), 1.32 – 1.22 (m, 1H, C11-H), 1.20 (s, 3H, C7-H_3), 0.96 – 0.85 (m, 1H, C9-H).
^{13}C NMR (101 MHz, CD_3OD) δ ppm: 165.5 (C12), 155.8 (C1), 129.2 (C6), 71.1 (C10), 41.0 (C3), 38.5 (C2), 37.2 (C11), 36.6 (C8), 33.3 (C9), 30.5 (C7), 25.3 (C4), 20.7 (C5), 18.6 (C13).
LC-LRMS (ESI+) *m/z*: 237.96 found for $[M+H]^+$, 238.13 calcd. for $C_{13}H_{20}NOS^+$, R_t = 1.17 min, purity (UV area): 100%.
HRMS (ESI+) *m/z*: 238.1261 found for $[M+H]^+$, 238.1260 calcd. for $C_{13}H_{20}NOS^+$ (Δ = 0.52 ppm).

(5a*R,7*R**,9a*S**)-9a-Methyl-2-(pyridin-3-yl)-4,5,5a,6,7,8,9,9a-octahydronaphtho[1,2-*d*]thiazol-7-ol (8c)**

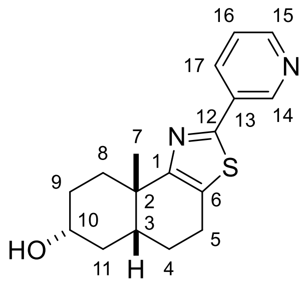

8c

Following general procedure D using compound **6**/**6'** (85.5 mg, 0.327 mmol) and thionicotinamide (67.9 mg, 0.491 mmol), refluxing for four hours, and purifying twice 1. on silica gel eluting with 1:1 to 3:2 acetonitrile/dichloromethane and 2. on neutralised (with triethylamine) silica gel eluting with 99:0:1 to 83:16:1 dichloromethane/acetonitrile/triethylamine yielded compound **8c** containing a triethylamine salt. To remove the salt, the residue was added tetrahydrofuran and filtered washing with tetrahydrofuran. The filtrate was concentrated under reduced pressure and dried on high vacuum to afford compound **8c** (39.2 mg, 45% yield) as a white solid.

TLC: R_f = 0.49 (83:16:1 dichloromethane/acetonitrile/triethylamine (TLC plate neutralised with triethylamine)), UV.
mp = 157.0 °C.
IR (ATR) $\tilde{\upsilon}_{max}$ cm^{-1}: 3324 (br, m), 3063 (w), 3043 (w), 2927 (s), 2907 (s), 2882 (m), 2843 (m), 1724 (w), 1659 (w), 1573 (m), 1529 (m), 1490 (m), 1453 (w), 1440 (m), 1420 (m), 1357 (m), 1341 (m), 1324 (m), 1285 (m), 1261 (m), 1252 (m), 1241 (w), 1220 (w), 1190 (w), 1155 (w), 1125 (w), 1188 (w), 1074 (m), 1036 (m), 1024 (m), 1009 (w), 989 (m), 957 (w), 946 (m), 803 (m), 701 (m), 594 (w).

^{1}H NMR (400 MHz, CD_3OD) δ ppm: 9.04 (dd, *J* = 2.3, 0.9 Hz, 1H, C14-H), 8.56 (dd, *J* = 4.9, 1.6 Hz, 1H, C15-H), 8.29 (ddd, *J* = 8.0, 2.2, 1.6 Hz, 1H, C17-H), 7.52 (ddd, *J* = 8.1, 4.9, 0.9 Hz, 1H, C16-H), 3.64 (tt, *J* = 11.0, 4.2 Hz, 1H, C10-H), 2.99 – 2.84 (m, 2H, C5-H_2), 2.78 (dt, *J* = 13.7, 3.5 Hz, 1H, C8-H), 2.35 – 2.22 (m, 1H, C4-H), 1.85 (dq, *J* = 12.7, 3.6 Hz, 1H, C3-H), 1.80 – 1.70 (m, 3H, C4-H + C9-H + C11-H), 1.45 (td, *J* = 13.7, 3.3 Hz, 1H, C8-H), 1.28 (s, 4H, C7-H_3 + C11-H), 1.03 – 0.89 (m, 1H, C9-H).
^{13}C NMR (101 MHz, CD_3OD) δ ppm: 162.3 (C12), 158.7 (C1), 150.7 (C15), 147.5 (C14), 135.3 (C17), 132.0 (C13), 131.5 (C6), 125.6 (C16), 71.2 (C10), 40.9 (C3), 38.8 (C2), 37.3 (C11), 36.7 (C8), 33.3 (C9), 30.6 (C7), 25.3 (C4), 21.0 (C5).
LC-LRMS (ESI+) *m/z*: 301.02 found for $[M+H]^+$, 301.14 calcd. for $C_{17}H_{21}N_2OS^+$, R_t = 1.63 min, purity (UV area): 95%.
HRMS (ESI+) *m/z*: 301.1372 found for $[M+H]^+$, 301.1369 calcd. for $C_{17}H_{21}N_2OS^+$ (Δ = 1.07 ppm).

(5a*R,7*R**,9a*S**)-2-(4-Methoxyphenyl)-9a-methyl-4,5,5a,6,7,8,9,9a-octahydronaphtho[1,2-*d*]thiazol-7-ol (8d)**

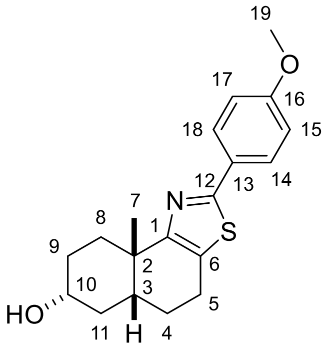

8d

Following general procedure D using compound **6**/**6'** (70.0 mg, 0.268 mmol) and 4-methoxythiobenzamide (67.2 mg, 0.402 mmol), refluxing for 1.5 hours, purifying by preparative HPLC, and drying by lyophilisation afforded compound **8d** (17.0 mg, 19% yield) as a white solid.

TLC: R_f = 0.63 (97:2:1 dichloromethane/acetonitrile/triethylamine), UV.
mp = 140.0 – 143.0 °C.
IR (ATR) $\tilde{\upsilon}_{max}$ cm^{-1}: 3245 (m), 3078 (w), 3045 (w), 2925 (s), 2886 (m), 2859 (s), 1606 (m), 1576 (w), 1537 (w), 1518 (m), 1462 (m), 1434 (w), 1411 (w), 1373 (w), 1298 (w), 1250 (s), 1167 (m), 1125 (w), 1109 (w), 1091 (w), 1073 (m), 1037 (m), 1025 (w), 987 (w), 953 (w), 821 (m), 792 (w), 720 (w).
^{1}H NMR (400 MHz, CD_3OD) δ ppm: 7.83 – 7.76 (m, 2H, C14-H + C18-H), 7.01 – 6.94 (m, 2H, C15-H + C17-H), 3.84 (s, 3H, C19-H_3), 3.62 (tt, *J* = 11.0, 3.9 Hz, 1H, C10-H), 2.94 – 2.79 (m, 2H, C5-H_2), 2.74 (dt, *J* = 13.8, 3.6 Hz, 1H, C8-H), 2.31 – 2.21 (m, 1H, C4-H), 1.85 – 1.66 (m, 4H, C3-H + C4-H + C9-H + C11-H), 1.40 (td, *J* = 14.1, 3.6 Hz, 1H, C8-H), 1.34 – 1.23 (m, 4H, C7-H_3 + C11-H), 0.98 (tdd, *J* = 13.8, 11.1, 3.4 Hz, 1H, C9-H).
^{13}C NMR (101 MHz, CD_3OD) δ ppm: 166.7 (C12), 162.5 (C16), 157.4 (C1), 128.9 (C6), 128.8 (C14 + C18), 128.2 (C13), 115.2 (C15 + C17), 71.3 (C10), 55.9 (C19), 41.1 (C3), 38.7 (C2), 37.3 (C11), 36.7 (C8), 33.4 (C9), 30.5 (C7), 25.4 (C4), 20.9 (C5).
LC-LRMS (ESI+) *m/z*: 330.43 found for $[M+H]^+$, 330.15 calcd. for $C_{19}H_{24}NO_2S^+$, R_t = 1.82 min, purity (UV area): 100%.
HRMS (ESI+) *m/z*: 330.1525 found for $[M+H]^+$, 330.1522 calcd. for $C_{19}H_{24}NO_2S^+$ (Δ = 0.98 ppm).

Note: Residual methanol observed in the 1D ^{1}H NMR spectrum.

(5a*R,7*R**,9a*S**)-2-(4-Fluorophenyl)-9a-methyl-4,5,5a,6,7,8,9,9a-octahydronaphtho[1,2-*d*]thiazol-7-ol (8e)**

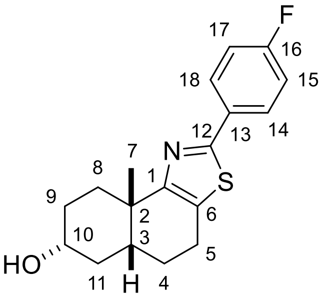

8e

Following general procedure D using compound **6**/**6'** (75.0 mg, 0.287 mmol) and 4-fluorothiobenzamide (66.9 mg, 0.431 mmol), adding additional 4-fluorothiobenzamide (0.5 equiv.) after two hours, refluxing for six hours, and purifying thrice on silica gel eluting with 1. 9:1 to 7:3 *n*-heptane/ethyl acetate, 2. 7:3 to 1:1 *n*-heptane/ethyl acetate, and 3. 1:0 to 7:3 *n*-heptane/ethyl acetate afforded compound **8e** (13.3 mg, 15% yield) as a yellow solid.

TLC: R_f = 0.16 (1:1 *n*-heptane/ethyl acetate), UV.
mp = 64.0 °C.
IR (ATR) $\tilde{\upsilon}_{\max}$ cm^{-1}: 3330 (br, m), 3077 (w), 2927 (s), 2853 (m), 1722 (w), 1602 (m), 1535 (w), 1512 (s), 1458 (m), 1437 (m), 1407 (w), 1364 (w), 1339 (w), 1296 (w), 1259 (w), 1228 (m), 1155 (m), 1125 (w), 1095 (w), 1072 (m), 1035 (m), 990 (m), 955 (w), 942 (w), 836 (m), 809 (w), 728 (w), 689 (w), 604 (w), 587 (w).
^{1}H NMR (400 MHz, $CDCl_3$) δ ppm: 7.97 – 7.86 (m, 2H, C14-H + C18-H), 7.13 – 7.06 (m, 2H, C15-H + C17-H), 3.71 (tt, *J* = 10.7, 4.1 Hz, 1H, C10-H), 2.91 – 2.76 (m, 3H, C5-H_2 + C8-H), 2.27 – 2.17 (m, 1H, C4-H), 1.85 – 1.66 (m, 4H, C3-H + C4-H + C9-H + C11-H), 1.47 – 1.22 (m, 5H, C7-H_3 + C8-H + C11-H), 1.09 – 0.98 (m, 1H, C9-H).
^{13}C NMR (101 MHz, $CDCl_3$) δ ppm: 164.2 (C12), 163.9 (d, *J* = 249.9 Hz, C16), 156.2 (C1), 130.0 (C13), 128.63 (d, *J* = 8.8 Hz, C14 + C18), 128.58 (C6), 116.0 (d, *J* = 22.0 Hz, C15 + C17), 70.7 (C10), 39.7 (C3), 37.7 (C2), 36.5 (C11), 35.6 (C8), 32.8 (C9), 30.2 (C7), 24.5 (C4), 20.4 (C5).
^{19}F NMR (377 MHz, $CDCl_3$) δ ppm: -111.0.
LC-LRMS (ESI+) *m/z*: 318.38 found for $[M+H]^+$, 318.13 calcd. for $C_{18}H_{21}FNOS^+$, R_t = 1.91 min, purity (UV area): 98%.
HRMS (ESI+) *m/z*: 318.1325 found for $[M+H]^+$, 318.1322 calcd. for $C_{18}H_{21}FNOS^+$ (Δ = 0.83 ppm).

Note: LC-LRMS indicates a purity of 98% (please see LC-LRMS trace) but unknown impurities are observed in the 1D ^{13}C and ^{19}F NMR spectra.

Synthesis of imidazole analogue

(5a*R,7*R**,9a*S**)-9a-Methyl-2-phenyl-4,5,5a,6,7,8,9,9a-octahydro-3*H*-naphtho[1,2-*d*]imidazol-7-ol (9)**

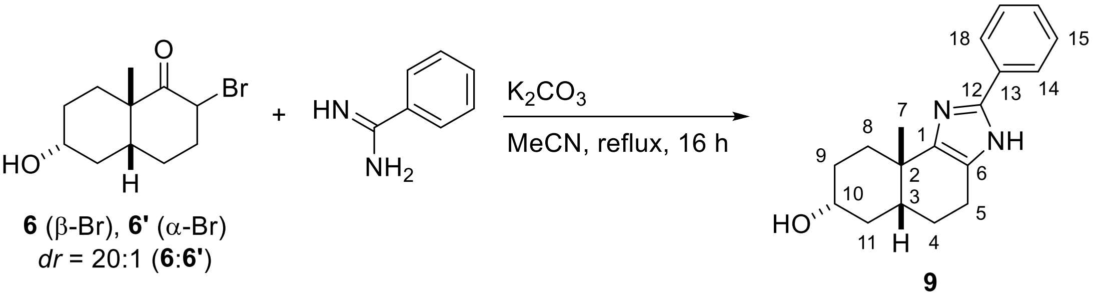

6 (β-Br), **6'** (α-Br)
dr = 20:1 (**6**:**6'**)

9
19%

A solution of a diastereoisomeric mixture of compound **6** and **6'** (75.0 mg, 0.287 mmol, 1.00 equiv.) with a *dr* = 20:1 (**6**:**6'**), benzamidine (36.5 mg, 0.304 mmol, 1.05 equiv.), and K_2CO_3 (1.00 equiv.) in dry

acetonitrile (10 mL) was stirred at reflux for 16 hours. The reaction mixture was cooled to room temperature, neutralised with 2 M aqueous HCl, and extracted with ethyl acetate (3 × 25 mL). The combined organic phases were washed with brine (10 mL). The combined aqueous phases were adjusted to pH 8-9 with saturated aqueous $NaHCO_3$, and back-extracted with 9:1 chloroform/isopropanol (3 × 25 mL). The combined organic phases were dried over anhydrous Na_2SO_4, and the solvent removed under reduced pressure to yield a crude light-brown sticky solid. The crude product was purified by flash column chromatography on BÜCHI® system using FlashPure EcoFlex Silica (12 g; 49:1 to 0:1 dichloromethane/methanol; flow rate = 20 mL/min) to afford compound **9** (15.5 mg, 19% yield) as a white solid.

TLC: R_f = 0.21 (23:2 dichloromethane/methanol), UV.
dec pt = 145.0 – 149.5 °C.
IR (ATR) $\tilde{\upsilon}_{\max}$ cm^{-1}: 3217 (br, m), 3166 (m), 3115 (m), 3102 (m), 3069 (m), 3042 (m), 2926 (s), 2854 (s), 1662 (w), 1595 (m), 1529 (w), 1483 (w), 1459 (m), 1399 (m), 1365 (m), 1297 (w), 1253 (w), 1229 (w), 1163 (w), 1124 (w), 1102 (w), 1073 (m), 1033 (m), 971 (w), 947 (w), 920 (w), 906 (w), 771 (m), 692 (m).
^{1}H NMR (400 MHz, CD_3OD) δ ppm: 7.86 – 7.80 (m, 2H, C14-H + C18-H), 7.45 – 7.38 (m, 2H, C15-H + C17-H), 7.36 – 7.30 (m, 1H, C16-H), 3.61 (tt, *J* = 10.8, 4.0 Hz, 1H, C10-H), 2.73 – 2.55 (m, 2H, C5-H_2), 2.48 (dt, *J* = 14.1, 3.6 Hz, 1H, C8-H), 2.26 – 2.15 (m, 1H, C4-H), 1.81 – 1.65 (m, 4H, C3-H + C4-H + C9-H + C11-H), 1.45 (td, *J* = 13.9, 3.3 Hz, 1H, C8-H), 1.34 (q, *J* = 12.1 Hz, 1H, C11-H), 1.26 (s, 3H, C7-H_3), 1.12 – 1.00 (m, 1H, C9-H).
^{13}C NMR (101 MHz, CD_3OD) δ ppm: 146.7 (C12), 136.7 (C1), 131.8 (C13), 131.4 (C6), 129.8 (C15 + C17), 129.3 (C16), 126.3 (C14 + C18), 71.2 (C10), 41.9 (C3), 37.1 (C11), 35.83 (C2), 35.77 (C8), 33.2 (C9), 30.8 (C7), 25.4 (C4), 19.9 (C5).
LC-LRMS (ESI+) *m/z*: 283.04 found for $[M+H]^+$, 283.18 calcd. for $C_{18}H_{23}N_2O^+$, R_t = 0.80 min, purity (UV area): ≥99%.
HRMS (ESI+) *m/z*: 283.1805 found for $[M+H]^+$, 283.1805 calcd. for $C_{18}H_{23}N_2O^+$ (Δ = 0.00 ppm).

Note: The peaks for the C1 and C6 carbons at 136.7 and 131.4 ppm are of low intensity in the 1D ^{13}C NMR spectrum in methanol-*d*$_4$, but the chemical shifts were confirmed by correlations in the 2D ^{1}H-^{13}C HMBC spectrum.

Synthesis of indole analogues

General procedure E

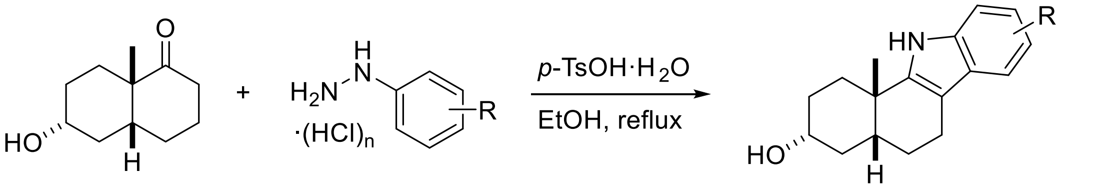

1 **10**

To a solution of compound **1** (1.0 equiv.) and *p*-toluenesulfonic acid monohydrate (2.0 equiv.) in absolute ethanol (0.12 M **1**) at room temperature was added the appropriate phenylhydrazine or phenylhydrazine hydrochloride salt (1.4 equiv.). The reaction mixture was stirred at reflux for the indicated time. The reaction mixture was cooled to room temperature, neutralised with saturated aqueous $NaHCO_3$, and extracted with dichloromethane (3×). The combined organic phases were washed with 5% aqueous HCl, water, and brine, dried over anhydrous Na_2SO_4, and the solvent removed under reduced pressure to yield the crude products. The crude products were purified by flash column chromatography to afford the indole analogues (**10**).

(3*R,4a*R**,11b*S**)-11b-Methyl-2,3,4,4a,5,6,11,11b-octahydro-1*H*-benzo[*a*]carbazol-3-ol (10a)**

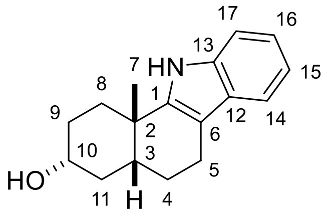

10a

Following general procedure E using compound **1** (248 mg, 1.36 mmol) and phenylhydrazine (190 µL, 1.93 mmol), refluxing for 21 hours, and purifying on silica gel eluting with 10:1 dichloromethane/ethyl acetate afforded compound **10a** (265 mg, 76% yield) as an off-white solid.

TLC: R_f = 0.25 (10:1 dichloromethane/ethyl acetate), UV.
mp = 178.0 – 181.0 °C.
IR (ATR) $\tilde{\nu}_{max}$ cm^{-1}: 3513 (s), 3276 (br, s), 3192 (m), 3107 (m), 3074 (w), 3026 (w), 2975 (m), 2927 (w), 2889 (m), 2857 (m), 2847 (m), 1578 (w), 1559 (w), 1494 (w), 1453 (m), 1356 (m), 1325 (m), 1283 (m), 1255 (m), 1235 (m), 1124 (m), 1096 (m), 1069 (m), 1034 (m), 1009 (m), 946 (w), 743 (m), 730 (m).
^{1}H NMR (400 MHz, $CDCl_3$) δ ppm: 7.72 (br s, 1H, N-H), 7.48 – 7.43 (m, 1H, C14-H), 7.33 – 7.28 (m, 1H, C17-H), 7.16 – 7.05 (m, 2H, C15-H + C16-H), 3.67 (tt, *J* = 10.8, 4.1 Hz, 1H, C10-H), 2.79 – 2.66 (m, 2H, C5-H_2), 2.26 – 2.09 (m, 2H, C4-H + C8-H), 1.85 – 1.68 (m, 4H, C3-H + C4-H + C9-H + C11-H), 1.57 (td, *J* = 14.0, 3.3 Hz, 1H, C8-H), 1.49 – 1.38 (m, 1H, C11-H), 1.30 (s, 3H, C7-H_3), 1.11 – 0.99 (m, 1H, C9-H).
^{13}C NMR (101 MHz, $CDCl_3$) δ ppm: 138.6 (C1), 135.9 (C13), 128.2 (C12), 121.2 (C16), 119.3 (C15), 118.2 (C14), 110.7 (C17), 108.6 (C6), 70.8 (C10), 40.7 (C3), 36.7 (C11), 35.8 (C8), 34.9 (C2), 32.7 (C9), 30.9 (C7), 24.5 (C4), 17.4 (C5).
LC-LRMS (ESI+) *m/z*: 256.06 found for $[M+H]^+$, 256.17 calcd. for $C_{17}H_{22}NO^+$, R_t = 1.68 min, purity (UV area): ≥95%.
HRMS (ESI+) *m/z*: 256.1702 found for $[M+H]^+$, 256.1696 calcd. for $C_{17}H_{22}NO^+$ (Δ = 2.43 ppm).

(3*R,4a*R**,11b*S**)-8-Bromo-11b-methyl-2,3,4,4a,5,6,11,11b-octahydro-1*H*-benzo[*a*]carbazol-3-ol (10b)**

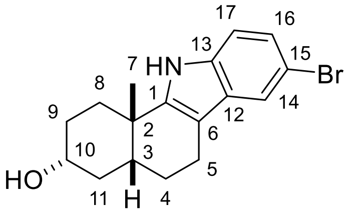

10b

Following general procedure E using compound **1** (1.50 g, 8.23 mmol) and 4-bromophenylhydrazine hydrochloride (2.58 g, 11.6 mmol), refluxing for 22 hours, and purifying on silica gel eluting with 11:1 dichloromethane/ethyl acetate afforded compound **10b** (2.36 g, 86% yield) as an off-white solid.

TLC: R_f = 0.26 (11:1 dichloromethane/ethyl acetate), UV.
mp = 90.0 – 93.0 °C.
IR (ATR) $\tilde{\nu}_{max}$ cm^{-1}: 3550 (w), 3423 (m), 3296 (br, s), 3093 (w), 3056 (w), 2970 (w), 2928 (s), 2855 (s), 1711 (m), 1573 (w), 1473 (m), 1434 (s), 1372 (m), 1302 (m), 1254 (m), 1235 (m), 1176 (w), 1128 (w), 1104 (w), 1070 (m), 1032 (m), 1018 (m), 959 (w), 946 (w), 888 (w), 859 (w), 792 (m), 635 (w).
^{1}H NMR (400 MHz, $CDCl_3$) δ ppm: 7.75 (br s, 1H, N-H), 7.56 (s, 1H, C14-H), 7.23 – 7.13 (m, 2H, C16-H + C17-H), 3.68 (tt, *J* = 10.8, 4.0 Hz, 1H, C10-H), 2.74 – 2.62 (m, 2H, C5-H_2), 2.24 – 2.07 (m, 2H, C4-H + C8-H), 1.86 – 1.68 (m, 4H, C3-H + C4-H + C9-H + C11-H), 1.58 (td, *J* = 14.1, 3.3 Hz, 1H, C8-H), 1.39 (q, *J* = 11.6 Hz, 1H, C11-H), 1.29 (s, 3H, C7-H_3), 1.08 – 0.96 (m, 1H, C9-H).
^{13}C NMR (101 MHz, $CDCl_3$) δ ppm: 140.2 (C1), 134.5 (C13), 130.0 (C12), 123.9 (C16), 120.8 (C14), 112.6 (C15), 112.0 (C17), 108.4 (C6), 70.6 (C10), 40.5 (C3), 36.6 (C11), 35.7 (C8), 34.9 (C2), 32.6 (C9), 30.8 (C7), 24.4 (C4), 17.3 (C5).

LC-LRMS (ESI+) *m/z*: 333.97 found for $[M+H]^+$, 334.08 calcd. for $C_{17}H_{21}BrNO^+$, R_t = 1.91 min, purity (UV area): 98%.
HRMS (ESI+) *m/z*: 334.0810 found for $[M+H]^+$, 334.0801 calcd. for $C_{17}H_{21}BrNO^+$ (Δ = 2.76 ppm).

(3*R,4a*R**,11b*S**)-8-Chloro-11b-methyl-2,3,4,4a,5,6,11,11b-octahydro-1*H*-benzo[*a*]carbazol-3-ol (10c)**

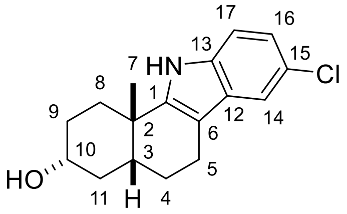

10c

Following general procedure E using compound **1** (150 mg, 0.823 mmol) and 4-chlorophenylhydrazine hydrochloride (207 mg, 1.16 mmol), refluxing for 20 hours, and purifying on BÜCHI® system using FlashPure EcoFlex Silica (12 g) eluting with 87:13 to 0:1 dichloromethane/ethyl acetate (flow rate = 30 mL/min) afforded compound **10c** (233 mg, 98% yield) as an off-white solid.

TLC: R_f = 0.23 (11:1 dichloromethane/ethyl acetate), UV.
mp = 83.0 – 84.6 °C.
IR (ATR) $\tilde{\upsilon}_{max}$ cm^{-1}: 3548 (w), 3423 (m), 3280 (br, m), 3102 (w), 3060 (w), 2929 (s), 2856 (s), 1712 (w), 1575 (w), 1473 (m), 1444 (m), 1373 (w), 1304 (m), 1254 (m), 1236 (m), 1176 (w), 1107 (w), 1071 (w), 1032 (m), 1018 (w), 964 (w), 946 (w), 893 (w), 859 (w), 794 (w), 591 (w).
^{1}H NMR (400 MHz, $CDCl_3$) δ ppm: 7.74 (br s, 1H, N-H), 7.40 (d, *J* = 2.0 Hz, 1H, C14-H), 7.20 (d, *J* = 8.6 Hz, 1H, C17-H), 7.07 (dd, *J* = 8.5, 2.0 Hz, 1H, C16-H), 3.73 – 3.62 (m, 1H, C10-H), 2.71 – 2.64 (m, 2H, C5-H_2), 2.24 – 2.07 (m, 2H, C4-H + C8-H), 1.85 – 1.67 (m, 4H, C3-H + C4-H + C9-H + C11-H), 1.57 (td, *J* = 14.0, 3.3 Hz, 1H, C8-H), 1.39 (q, *J* = 11.6 Hz, 1H, C11-H), 1.29 (s, 3H, C7-H_3), 1.08 – 0.97 (m, 1H, C9-H).
^{13}C NMR (101 MHz, $CDCl_3$) δ ppm: 140.3 (C1), 134.3 (C13), 129.3 (C12), 125.0 (C15), 121.3 (C16), 117.8 (C14), 111.5 (C17), 108.5 (C6), 70.7 (C10), 40.5 (C3), 36.6 (C11), 35.7 (C8), 34.9 (C2), 32.6 (C9), 30.8 (C7), 24.4 (C4), 17.3 (C5).
LC-LRMS (ESI+) *m/z*: 289.93 found for $[M+H]^+$, 290.13 calcd. for $C_{17}H_{21}ClNO^+$, R_t = 1.79 min, purity (UV area): 97%.
HRMS (ESI+) *m/z*: 290.1297 found for $[M+H]^+$, 290.1306 calcd. for $C_{17}H_{21}ClNO^+$ (Δ = -3.02 ppm).

(3*R,4a*R**,11b*S**)-8-Fluoro-11b-methyl-2,3,4,4a,5,6,11,11b-octahydro-1*H*-benzo[*a*]carbazol-3-ol (10d)**

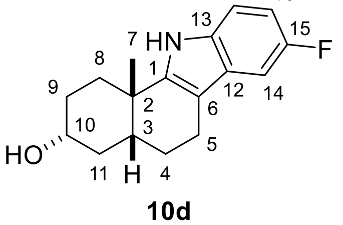

10d

Following general procedure E using compound **1** (150 mg, 0.823 mmol) and 4-fluorophenylhydrazine hydrochloride (188 mg, 1.16 mmol), refluxing for 20 hours, and purifying on BÜCHI® system using FlashPure EcoFlex Silica (12 g) eluting with 1:0 to 0:1 dichloromethane/ethyl acetate (flow rate = 30 mL/min) afforded compound **10d** (218 mg, 97% yield) as an off-white solid.

TLC: R_f = 0.22 (12:1 dichloromethane/ethyl acetate), UV.
mp = 75.6 – 77.7 °C.
IR (ATR) $\tilde{\upsilon}_{max}$ cm^{-1}: 3600 (w), 3548 (w), 3463 (w), 3423 (m), 3300 (br, m), 3071 (w), 3059 (w), 2928 (s), 2856 (m), 1720 (w), 1627 (w), 1582 (w), 1483 (m), 1453 (s), 1366 (w), 1313 (w), 1283 (m), 1254 (m), 1231 (m), 1177 (m), 1162 (m), 1128 (w), 1093 (w), 1070 (m), 1032 (m), 1018 (m), 979 (w), 946 (w), 910 (w), 847 (w), 836 (w), 793 (m), 634 (w).

^{1}H NMR (400 MHz, $CDCl_3$) δ ppm: 7.69 (br s, 1H, N-H), 7.19 (dd, *J* = 8.7, 4.3 Hz, 1H, C17-H), 7.08 (dd, *J* = 9.6, 2.5 Hz, 1H, C14-H), 6.85 (ddd, *J* = 9.4, 8.7, 2.5 Hz, 1H, C16-H), 3.74 – 3.62 (m, 1H, C10-H), 2.71 – 2.63 (m, 2H, C5-H_2), 2.26 – 2.07 (m, 2H, C4-H + C8-H), 1.87 – 1.66 (m, 4H, C3-H + C4-H + C9-H + C11-H), 1.57 (tq, *J* = 14.2, 3.4 Hz, 1H, C8-H), 1.41 (q, *J* = 11.7 Hz, 1H, C11-H), 1.30 (s, 3H, C7-H_3), 1.11 – 0.97 (m, 1H, C9-H).
^{13}C NMR (101 MHz, $CDCl_3$) δ ppm: 157.9 (d, *J* = 234.0 Hz, C15), 140.7 (C1), 132.4 (C13), 128.6 (d, *J* = 9.6 Hz, C12), 111.0 (d, *J* = 9.6 Hz, C17), 109.1 (d, *J* = 26.0 Hz, C16), 108.9 (d, *J* = 4.4 Hz, C6), 103.3 (d, *J* = 23.3 Hz, C14), 70.7 (C10), 40.6 (C3), 36.7 (C11), 35.7 (C8), 35.0 (C2), 32.7 (C9), 30.8 (C7), 24.4 (C4), 17.4 (C5).
^{19}F NMR (377 MHz, $CDCl_3$) δ ppm: -125.3.
LC-LRMS (ESI+) *m/z*: 273.96 found for $[M+H]^+$, 274.16 calcd. for $C_{17}H_{21}FNO^+$, R_t = 1.65 min, purity (UV area): 96%.
HRMS (ESI+) *m/z*: 274.1599 found for $[M+H]^+$, 274.1602 calcd. for $C_{17}H_{21}FNO^+$ (Δ = -1.00 ppm).

(3*R,4a*R**,11b*S**)-8,11b-Dimethyl-2,3,4,4a,5,6,11,11b-octahydro-1*H*-benzo[*a*]carbazol-3-ol (10e)**

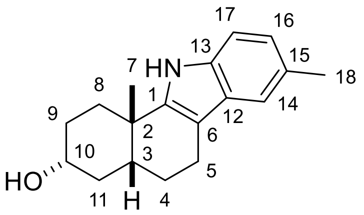

10e

Following general procedure E using compound **1** (250.0 mg, 1.37 mmol) and 4-methylphenylhydrazine hydrochloride (305 mg, 1.92 mmol), refluxing for 18.5 hours, and purifying on silica gel eluting with 14:1 dichloromethane/ethyl acetate afforded compound **10e** (305 mg, 82% yield) as an off-white solid.

TLC: R_f = 0.20 (14:1 dichloromethane/ethyl acetate), UV.
mp = 212.5 – 215.5 °C.
IR (ATR) $\tilde{\upsilon}_{\max}$ cm^{-1}: 3509 (m), 3284 (br, m), 3017 (w), 2977 (w), 2930 (m), 2887 (m), 2860 (m), 1720 (w), 1586 (w), 1469 (m), 1454 (s), 1361 (m), 1347 (w), 1308 (s), 1255 (m), 1237 (m), 1193 (w), 1184 (w), 1152 (w), 1121 (w), 1093 (m), 1070 (m), 1035 (s), 1020 (m), 1010 (m), 966 (w), 949 (m), 899 (w), 867 (m), 797 (s), 728 (m), 702 (w), 543 (m).
^{1}H NMR (400 MHz, $CDCl_3$) δ ppm: 7.61 (br s, 1H, N-H), 7.24 (s, 1H, C14-H), 7.19 (d, *J* = 8.1 Hz, 1H, C17-H), 6.95 (dd, *J* = 8.1, 1.7 Hz, 1H, C16-H), 3.72 – 3.60 (m, 1H, C10-H), 2.75 – 2.65 (m, 2H, C5-H_2), 2.44 (s, 3H, C18-H_3), 2.26 – 2.07 (m, 2H, C4-H + C8-H), 1.85 – 1.65 (m, 4H, C3-H + C4-H + C9-H + C11-H), 1.56 (td, *J* = 14.0, 3.3 Hz, 1H, C8-H), 1.42 (q, J = 11.8 Hz, 1H, C11-H), 1.29 (s, 3H, C7-H_3), 1.11 – 0.97 (m, 1H, C9-H).
^{13}C NMR (101 MHz, $CDCl_3$) δ ppm: 138.8 (C1), 134.2 (C13), 128.6 (C15), 128.4 (C12), 122.7 (C16), 117.9 (C14), 110.3 (C17), 108.1 (C6), 70.8 (C10), 40.7 (C3), 36.7 (C11), 35.8 (C8), 34.9 (C2), 32.7 (C9), 30.8 (C7), 24.6 (C4), 21.6 (C18), 17.4 (C5).
LC-LRMS (ESI+) *m/z*: 270.03 found for $[M+H]^+$, 270.19 calcd. for $C_{18}H_{24}NO^+$, R_t = 1.97 min, purity (UV area): 99%.
HRMS (ESI+) *m/z*: 270.1853 found for $[M+H]^+$, 270.1853 calcd. for $C_{18}H_{24}NO^+$ (Δ = 0.00 ppm).

(3*R,4a*R**,11b*S**)-8-Methoxy-11b-methyl-2,3,4,4a,5,6,11,11b-octahydro-1*H*-benzo[*a*]carbazol-3-ol (10f)**

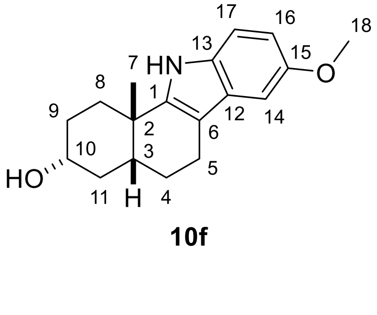

10f

Following general procedure E using compound **1** (225 mg, 1.23 mmol) and 4-methoxyphenylhydrazine hydrochloride (302 mg, 1.73 mmol), refluxing for 21 hours, and purifying on silica gel eluting with 7:1 dichloromethane/ethyl acetate afforded compound **10f** (255 mg, 72% yield) as a light-grey solid.

TLC: R_f = 0.25 (7:1 dichloromethane/ethyl acetate), UV.
mp = 191.5 – 194.5 °C.
IR (ATR) $\tilde{\upsilon}_{\max}$ cm^{-1}: 3516 (s), 3279 (br, s), 3066 (w), 3052 (w), 3011 (w), 2970 (m), 2931 (s), 2895 (m), 2862 (m), 2846 (m), 1723 (w), 1629 (w), 1585 (m), 1485 (m), 1466 (s), 1455 (s), 1432 (s), 1448 (w), 1362 (m), 1306 (m), 1255 (w), 1233 (m), 1204 (s), 1167 (s), 1122 (w), 1094 (s), 1071 (m), 1051 (w), 1026 (s), 948 (w), 869 (m), 838 (m), 788 (m).
^{1}H NMR (400 MHz, $CDCl_3$) δ ppm: 7.60 (br s, 1H, N-H), 7.19 (d, *J* = 8.7 Hz, 1H, C17-H), 6.92 (d, *J* = 2.4 Hz, 1H, C14-H), 6.78 (dd, *J* = 8.7, 2.5 Hz, 1H, C16-H), 3.86 (s, 3H, C18-H_3), 3.67 (tt, *J* = 10.6, 4.0 Hz, 1H, C10-H), 2.74 – 2.64 (m, 2H, C5-H_2), 2.26 – 2.08 (m, 2H, C4-H + C8-H), 1.85 – 1.68 (m, 4H, C3-H + C4-H + C9-H + C11-H), 1.60 – 1.50 (m, 1H, C8-H), 1.48 – 1.36 (m, 1H, C11-H), 1.29 (s, 3H, C7-H_3), 1.11 – 1.00 (m, 1H, C9-H).
^{13}C NMR (101 MHz, $CDCl_3$) δ ppm: 154.1 (C15), 139.7 (C1), 131.0 (C13), 128.5 (C12), 111.3 (C17), 110.9 (C16), 108.4 (C6), 100.6 (C14), 70.8 (C10), 56.2 (C18), 40.7 (C3), 36.7 (C11), 35.8 (C8), 35.0 (C2), 32.7 (C9), 30.9 (C7), 24.5 (C4), 17.5 (C5).
LC-LRMS (ESI+) *m/z*: 286.01 found for $[M+H]^+$, 286.18 calcd. for $C_{18}H_{24}NO_2^+$, R_t = 1.76 min, purity (UV area): ≥95%.
HRMS (ESI+) *m/z*: 286.1812 found for $[M+H]^+$, 286.1802 calcd. for $C_{18}H_{24}NO_2^+$ (Δ = 3.58 ppm).

11b-Methyl-8-(trifluoromethyl)-2,3,4,4a,5,6,11,11b-octahydro-1*H*-benzo[*a*]carbazol-3-ol (10g)

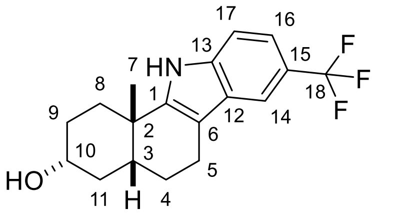

10g

Following general procedure E using compound **1** (205 mg, 1.12 mmol) and 4-(trifluoromethyl)phenylhydrazine hydrochloride (428 mg, 2.25 mmol), refluxing for 46 hours, and purifying on silica gel eluting with 20:1 dichloromethane/ethyl acetate afforded compound **10g** (224 mg, 62% yield) as a cream-white solid.

TLC: R_f = 0.17 (20:1 dichloromethane/ethyl acetate), UV.
mp = 156.5 – 159.5 °C.
IR (ATR) $\tilde{\upsilon}_{\max}$ cm^{-1}: 3448 (m), 3338 (br, m), 3270 (br, m), 3076 (w), 3055 (w), 2992 (w), 2934 (m), 2857 (w), 1474 (w), 1460 (w), 1325 (s), 1266 (m), 1160 (m), 1114 (s), 1067 (w), 1055 (m), 1042 (m), 1031 (m), 969 (w), 896 (w), 804 (w), 638 (w).
^{1}H NMR (400 MHz, $CDCl_3$) δ ppm: 7.93 (br s, 1H, N-H), 7.73 (s, 1H, C14-H), 7.39 – 7.32 (m, 2H, C16-H + C17-H), 3.69 (tt, *J* = 10.8, 4.1 Hz, 1H, C10-H), 2.81 – 2.67 (m, 2H, C5-H_2), 2.26 – 2.10 (m, 2H, C4-H + C8-H), 1.88 – 1.70 (m, 4H, C3-H + C4-H + C9-H + C11-H), 1.60 (td, *J* = 14.1, 3.3 Hz, 1H, C8-H), 1.38 (q, *J* = 11.9 Hz, 1H, C11-H), 1.31 (s, 3H, C7-H_3), 1.08 – 0.95 (m, 1H, C9-H).
^{13}C NMR (101 MHz, $CDCl_3$) δ ppm: 140.6 (C1), 137.3 (C13), 127.6 (C12), 125.6 (q, *J* = 271.3 Hz, C18), 121.7 (q, *J* = 31.7 Hz, C15), 118.1 (q, *J* = 3.6 Hz, C16), 115.8 (q, *J* = 4.2 Hz, C14), 110.7 (C17), 109.6 (C6), 70.6 (C10), 40.5 (C3), 36.6 (C11), 35.7 (C8), 35.0 (C2), 32.6 (C9), 30.7 (C7), 24.4 (C4), 17.3 (C5).
^{19}F NMR (377 MHz, $CDCl_3$) δ ppm: -60.2.
LC-LRMS (ESI+) *m/z*: 324.00 found for $[M+H]^+$, 324.16 calcd. for X^+, R_t = 1.85 min, purity (UV area): >99%.
HRMS (ESI+) *m/z*: 324.1571 found for $[M+H]^+$, 324.1570 calcd. for $C_{18}H_{21}F_3NO^+$ (Δ = 0.38 ppm).

(3*R,4a*R**,11b*S**)-8-Iodo-11b-methyl-2,3,4,4a,5,6,11,11b-octahydro-1*H*-benzo[*a*]carbazol-3-ol (10h)**

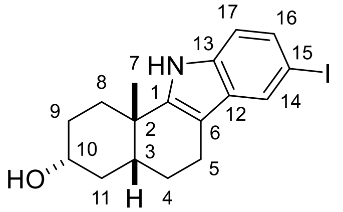

10h

Following general procedure E using compound **1** (125 mg, 0.687 mmol) and 4-iodophenylhydrazine hydrochloride (260 mg, 0.961 mmol), refluxing for 23 hours, and purifying twice on silica gel eluting with 1. 6:1 to 4:1 dichloromethane/ethyl acetate and 2. 4:1 to 3:2 *n*-pentane/ethyl acetate afforded compound **10h** (8.4 mg, 3% yield) as a brown solid.

Note: The deiodinated side-product (**10a**; 29.6 mg, 17% yield) was isolated in separate fractions.

TLC: R_f = 0.24 (3:2 *n*-pentane/ethyl acetate), UV.
dec pt = 98.0 – 101.0 °C.
IR (ATR) $\tilde{\upsilon}_{\max}$ cm^{-1}: 3534 (w), 3414 (m), 3283 (br, m), 3049 (w), 2926 (s), 2852 (s), 1695 (w), 1572 (w), 1471 (m), 1453 (m), 1433 (s), 1363 (w), 1300 (m), 1236 (w), 1214 (w), 1176 (w), 1102 (w), 1069 (w), 1031 (m), 1017 (m), 956 (w), 885 (w), 793 (m), 750 (m), 731 (m).
^{1}H NMR (400 MHz, $CDCl_3$) δ ppm: 7.82 – 7.67 (m, 2H, C14-H + N-H), 7.37 (dd, *J* = 8.4, 1.7 Hz, 1H, C16-H), 7.08 (d, *J* = 8.4 Hz, 1H, C17-H), 3.67 (tt, *J* = 10.8, 4.1 Hz, 1H, C10-H), 2.72 – 2.62 (m, 2H, C5-H_2), 2.24 – 2.07 (m, 2H, C4-H + C8-H), 1.87 – 1.66 (m, 4H, C3-H + C4-H + C9-H + C11-H), 1.65 – 1.52 (m, 1H, C8-H), 1.42 – 1.34 (m, 1H, C11-H), 1.29 (s, 3H, C7-H_3), 1.09 – 0.94 (m, 1H, C9-H).
^{13}C NMR (101 MHz, $CDCl_3$) δ ppm: 139.7 (C1), 135.0 (C13), 130.8 (C12), 129.5 (C16), 127.1 (C14), 112.6 (C17), 108.2 (C6), 82.7 (C15), 70.6 (C10), 40.5 (C3), 36.6 (C11), 35.7 (C8), 34.9 (C2), 32.6 (C9), 30.8 (C7), 24.4 (C4), 17.3 (C5).
LC-LRMS (ESI+) *m/z*: 382.02 found for [M+H]$^+$, 382.07 calcd. for $C_{17}H_{21}INO^+$, R_t = 2.20 min, purity (UV area): ≥95%.
HRMS (ESI+) *m/z*: 382.0674 found for [M+H]$^+$, 382.0663 calcd. for $C_{17}H_{21}INO^+$ (Δ = 2.94 ppm).

Note: The peak for the C8-H proton at 1.65 – 1.52 ppm in the 1D ^{1}H NMR spectrum overlaps with the H_2O solvent peak, thus increasing the integral, but the chemical shift and integral were confirmed by correlations in the 2D ^{1}H-^{1}H COESY and ^{1}H-^{13}C HSQC and HMBC spectra.

(3*R,4a*R**,11b*S**)-8-Chloro-10-fluoro-11b-methyl-2,3,4,4a,5,6,11,11b-octahydro-1*H*-benzo[*a*]carbazol-3-ol (10i)**

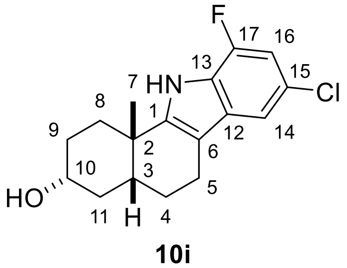

10i

Following general procedure E using compound **1** (225 mg, 1.24 mmol) and 4-chloro-2-fluorophenylhydrazine hydrochloride (341 mg, 1.73 mmol), refluxing for 18.5 hours, and purifying on silica gel eluting with 2:1 *n*-heptane/ethyl acetate afforded compound **10i** (281 mg, 74% yield) as a beige solid.

TLC: R_f = 0.18 (2:1 *n*-heptane/ethyl acetate), UV.
mp = 107.0 – 110.0 °C.
IR (ATR) $\tilde{\upsilon}_{\max}$ cm^{-1}: 3576 (w), 3439 (m), 3277 (br, m), 3060 (w), 2926 (s), 2855 (s), 1635 (w), 1574 (m), 1476 (m), 1454 (m), 1417 (w), 1368 (m), 1316 (w), 1296 (m), 1256 (w), 1206 (w), 1068 (m), 1032 (m), 1009 (m), 924 (m), 892 (m), 822 (w), 586 (w).

¹H NMR (400 MHz, $CDCl_3$) δ ppm: 7.88 (br s, 1H, N-H), 7.19 (d, *J* = 1.6 Hz, 1H, C14-H), 6.86 (dd, *J* = 10.7, 1.7 Hz, 1H, C16-H), 3.69 (tt, *J* = 10.7, 4.1 Hz, 1H, C10-H), 2.71 – 2.61 (m, 2H, C5-H_2), 2.23 – 2.11 (m, 2H, C4-H + C8-H), 1.88 – 1.69 (m, 4H, C3-H + C4-H + C9-H + C11-H), 1.64 – 1.54 (m, 1H, C8-H), 1.40 – 1.32 (m, 1H, C11-H), 1.30 (s, 3H, C7-H_3), 1.09 – 0.97 (m, 1H, C9-H).
¹³C NMR (101 MHz, $CDCl_3$) δ ppm: 148.7 (d, *J* = 245.9 Hz, C17), 141.0 (C1), 132.0 (d, *J* = 6.2 Hz, C12), 124.4 (d, *J* = 8.7 Hz, C15), 122.4 (d, *J* = 12.7 Hz, C13), 113.8 (d, *J* = 3.4 Hz, C14), 109.4 (d, *J* = 2.3 Hz, C6), 107.5 (d, *J* = 20.3 Hz, C16), 70.5 (C10), 40.4 (C3), 36.6 (C11), 35.6 (C8), 35.1 (C2), 32.6 (C9), 30.7 (C7), 24.3 (C4), 17.4 (C5).
¹⁹F NMR (377 MHz, $CDCl_3$) δ ppm: -133.5.
LC-LRMS (ESI+) *m/z*: 307.95 found for $[M+H]^+$, 308.12 calcd. for $C_{17}H_{20}ClFNO^+$, R_t = 1.93 min, purity (UV area): >99%.
HRMS (ESI+) *m/z*: 308.1215 found for $[M+H]^+$, 308.1212 calcd. for $C_{17}H_{20}ClFNO^+$ (Δ = 1.05 ppm).

(3*R,4a*R**,11b*S**)-7,9,11b-Trimethyl-2,3,4,4a,5,6,11,11b-octahydro-1*H*-benzo[*a*]carbazol-3-ol (10j)**

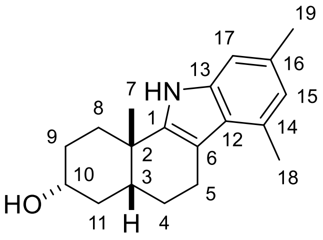

10j

Following general procedure E using compound **1** (151 mg, 0.830 mmol) and 3,5-dimethylphenylhydrazine hydrochloride (199 mg, 1.15 mmol), refluxing for six hours, and purifying on silica gel eluting with 11:1 dichloromethane/ethyl acetate afforded compound **10j** (135 mg, 57% yield) as an off-white solid.

TLC: R_f = 0.27 (11:1 dichloromethane/ethyl acetate), UV.
mp = 219.5 – 222.5 °C.
IR (ATR) $\tilde{\upsilon}_{max}$ cm^{-1}: 3417 (s), 3358 (br, s), 3286 (br, s), 3020 (w), 2968 (m), 2943 (s), 2928 (s), 2911 (s), 2887 (s), 2857 (s), 1722 (w), 1620 (w), 1557 (w), 1453 (s), 1407 (w), 1364 (m), 1333 (m), 1307 (m), 1292 (w), 1262 (w), 1219 (w), 1160 (w), 1133 (w), 1107 (w), 1068 (m), 1037 (m), 1028 (w), 1012 (m), 970 (w), 945 (w), 851 (m), 830 (m).
¹H NMR (400 MHz, $CDCl_3$) δ ppm: 7.55 (br s, 1H, N-H), 6.92 (s, 1H, C17-H), 6.64 (s, 1H, C15-H), 3.66 (tt, *J* = 10.9, 4.1 Hz, 1H, C10-H), 3.09 – 2.84 (m, 2H, C5-H_2), 2.61 (s, 3H, C18-H_3), 2.38 (s, 3H, C19-H_3), 2.23 – 2.05 (m, 2H, C4-H + C8-H), 1.83 – 1.63 (m, 4H, C3-H + C4-H + C9-H + C11-H), 1.59 – 1.37 (m, 2H, C8-H + C11-H), 1.28 (s, 3H, C7-H_3), 1.10 – 0.97 (m, 1H, C9-H).
¹³C NMR (101 MHz, $CDCl_3$) δ ppm: 137.1 (C1), 136.4 (C13), 131.0 (C16), 130.2 (C14), 124.9 (C12), 122.4 (C15), 108.8 (C6), 108.5 (C17), 70.8 (C10), 40.1 (C3), 36.6 (C11), 35.9 (C8), 34.7 (C2), 32.7 (C9), 30.9 (C7), 24.9 (C4), 21.6 (C19), 20.2 (C5), 19.8 (C18).
LC-LRMS (ESI+) *m/z*: 284.08 found for $[M+H]^+$, 284.20 calcd. for $C_{19}H_{26}NO^+$, R_t = 1.93 min, purity (UV area): 100%.
HRMS (ESI+) *m/z*: 284.2018 found for $[M+H]^+$, 284.2009 calcd. for $C_{19}H_{26}NO^+$ (Δ = 3.25 ppm).

Note: The peak for the C8-H and C11-H protons at 1.59 – 1.37 ppm in the 1D ¹H NMR spectrum overlaps with the H_2O solvent peak, thus increasing the integral, but the chemical shift and integral were confirmed by correlations in the 2D ¹H-¹H COESY and ¹H-¹³C HSQC and HMBC spectra.

Ethyl (3*R,4a*R**,11b*S**)-3-hydroxy-11b-methyl-2,3,4,4a,5,6,11,11b-octahydro-1*H*-benzo[*a*]carbazole-8-carboxylate (10k)**

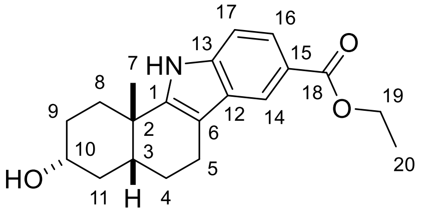

10k

Following general procedure E using compound **1** (0.852 g, 4.67 mmol) and methyl 4-hydrazinylbenzoate hydrochloride (1.34 g, 6.60 mmol), refluxing for 74 hours, and purifying on BÜCHI® system using FlashPure Select Silica (25 g) eluting with 1:0 to 0:1 *n*-heptane/acetone (flow rate = 24 mL/min) afforded compound **10k** (1.18 g, 77% yield) as a yellow solid.

Note: Transesterification from the methyl ester to the ethyl ester occurred under the reaction conditions. The purified product was isolated alongside 5% of inseparable non-transesterificated methyl ester.

TLC: R_f = 0.23 (13:7 *n*-heptane/acetone), UV.
mp = 197.5 – 199.5 °C.
IR (ATR) $\tilde{\upsilon}_{max}$ cm^{-1}: 3419 (w), 3361 (br, m), 3085 (w), 3064 (w), 3053 (w), 2974 (w), 2929 (s), 2856 (m), 1681 (s), 1617 (w), 1458 (w), 1444 (w), 1366 (w), 1307 (m), 1274 (m), 1294 (m), 1239 (s), 1174 (w), 1111 (m), 1092 (w), 1073 (m), 1033 (w), 1019 (w), 769 (w), 749 (w).
^{1}H NMR (400 MHz, $CDCl_3$) δ ppm: 8.21 (d, *J* = 1.6 Hz, 1H, C14-H), 7.94 (br s, 1H, N-H), 7.85 (dd, *J* = 8.5, 1.7 Hz, 1H, C16-H), 7.29 (d, *J* = 8.5 Hz, 1H, C17-H), 4.39 (q, *J* = 7.1 Hz, 2H, C19-H_2), 3.68 (tt, *J* = 10.8, 4.1 Hz, 1H, C10-H), 2.82 – 2.68 (m, 2H, C5-H_2), 2.25 – 2.10 (m, 2H, C4-H + C8-H), 1.87 – 1.69 (m, 4H, C3-H + C4-H + C9-H + C11-H), 1.59 (td, *J* = 14.0, 3.3 Hz, 1H, C8-H), 1.45 – 1.38 (m, 4H, C11-H + C20-H_3), 1.30 (s, 3H, C7- H_3), 1.09 – 0.97 (m, 1H, C9-H).
^{13}C NMR (101 MHz, $CDCl_3$) δ ppm: 168.0 (C18), 140.0 (C1), 138.6 (C13), 127.9 (C12), 122.9 (C16), 121.7 (C15), 121.0 (C14), 110.2 (C17), 110.0 (C6), 70.6 (C10), 60.6 (C19), 40.5 (C3), 36.6 (C11), 35.7 (C8), 34.9 (C2), 32.6 (C9), 30.7 (C7), 24.4 (C4), 17.4 (C5), 14.6 (C20).
LC-LRMS (ESI+) *m/z*: 328.12 found for $[M+H]^+$, 328.19 calcd. for $C_{20}H_{26}NO_3^+$, R_t = 1.66 min, purity (UV area): 94%.
HRMS (ESI+) *m/z*: 328.1907 found for $[M+H]^+$, 328.1907 calcd. for $C_{20}H_{26}NO_3^+$ (Δ = 0.00 ppm).

Note: LC-LRMS indicates a purity of 94% and 5% methyl ester (please see LC-LRMS trace). The exact amount of the methyl ester was determined by the 1D ^{1}H NMR spectrum methyl ester methyl (C(O)-C-H_3) protons integral at 3.93 ppm.

Synthesis of ketolactam analogues

General procedure F

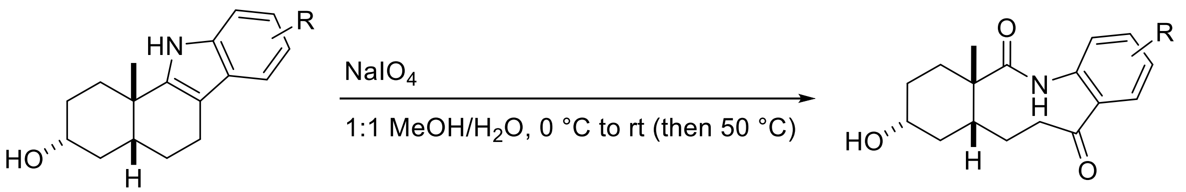

10 **11**

To a solution of the appropriate indole analogue (**10**; 1.0 equiv.) in methanol (41-59 mM **10**) at 0 °C was added a solution of $NaIO_4$ (4.0 equiv.) in deionised water (100 vol% of methanol; final **10** concentration = 21-29 mM) dropwise. The reaction mixture was stirred at room temperature for the indicated time. If necessary, additional $NaIO_4$ was added during the reaction. If necessary, the reaction was stirred at 50 °C. The reaction mixture was poured into water and extracted with dichloromethane (6×). The combined organic phases were dried over anhydrous $MgSO_4$ and the solvent removed under reduced pressure to yield the crude products. The crude products were purified by flash column chromatography on silica gel to afford the ketolactam analogues (**11**).

(6a*S,9*R**,10a*R**)-9-Hydroxy-6a-methyl-6a,7,8,9,10,10a,11,12-octahydro-5*H*-dibenzo[*b*,*g*]azonine-6,13-dione (11a)**

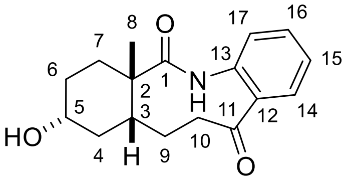

11a

Following general procedure F using compound **10a** (75.0 mg, 0.294 mmol), adding additional $NaIO_4$ (2.0 equiv.), stirring at room temperature for 28 hours, and eluting with 19:1 dichloromethane/methanol afforded compound **11a** (70.7 mg, 84% yield) as a white solid.

TLC: R_f = 0.25 (19:1 dichloromethane/methanol), UV.
mp = 215.0 – 218.0 °C.
IR (ATR) $\tilde{\upsilon}_{max}$ cm^{-1}: 3469 (w), 3305 (br, m), 3078 (w), 3034 (w), 2967 (w), 2929 (m), 2876 (w), 1671 (s), 1599 (m), 1502 (m), 1478 (m), 1433 (m), 1326 (w), 1273 (w), 1251 (w), 1242 (w), 1209 (w), 1046 (w), 1024 (w), 751 (w), 589 (w).
^{1}H NMR (800 MHz, DMSO-d_6, 333 K) δ ppm: 9.51 (s, 1H, N-H), 7.52 (td, *J* = 7.5, 1.7 Hz, 1H, C16-H), 7.33 (dd, *J* = 7.6, 1.7 Hz, 1H, C14-H), 7.30 (t, *J* = 7.4 Hz, 1H, C15-H), 7.18 (d, *J* = 7.8 Hz, 1H, C17-H), 4.08 (d, *J* = 2.8 Hz, 1H, O-H), 3.78 – 3.75 (m, 1H, C5-H), 2.48 – 2.41 (m, 2H, C10-H_2), 2.23 (td, *J* = 13.5, 4.2 Hz, 1H, C7-H), 2.08 – 2.03 (m, 1H, C9-H), 2.01 – 1.96 (m, 1H, C9-H), 1.85 – 1.79 (m, 2H, C3-H + C4-H), 1.64 – 1.59 (m, 1H, C6-H), 1.59 – 1.54 (m, 2H, C4-H + C6-H), 1.36 (s, 3H, C8-H_3), 0.98 (dt, *J* = 14.2, 4.3 Hz, 1H, C7-H).
^{13}C NMR (201 MHz, DMSO-d_6, 333 K) δ ppm: 204.1 (C11), 176.4 (C1), 139.3 (C12), 137.1 (C13), 131.0 (C16), 126.8 (C14), 126.7 (C17), 125.6 (C15), 64.1 (C5), 44.3 (C3), 43.0 (C2), 42.8 (C10), 35.2 (C4), 30.3 (C9), 28.3 (C6), 23.6 (C7), 20.9 (C8).
LC-LRMS (ESI+) *m/z*: 288.01 found for $[M+H]^+$, 288.16 calcd. for $C_{17}H_{22}NO_3^+$, R_t = 0.89 min, purity (UV area): 96%.
HRMS (ESI+) *m/z*: 310.1411 found for $[M+Na]^+$, 310.1414 calcd. for $C_{17}H_{21}NNaO_3^+$ (Δ = -0.74 ppm).

Note: Peak broadening in the 1D ^{13}C NMR spectrum observed which is resolved by VT NMR (please see NMR spectra).

(6a*S,9*R**,10a*R**)-2-Bromo-9-hydroxy-6a-methyl-6a,7,8,9,10,10a,11,12-octahydro-5*H*-dibenzo[*b*,*g*]azonine-6,13-dione (11b)**

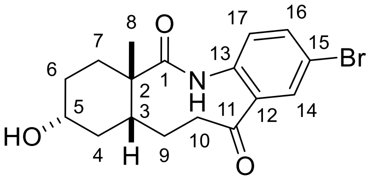

11b

Following general procedure F using compound **10b** (75.0 mg, 0.224 mmol), adding additional $NaIO_4$ (4.0 equiv.), stirring at room temperature for 30 hours, and eluting with 24:1 dichloromethane/methanol afforded compound **11b** (29.5 mg, 36% yield) as a grey solid.

TLC: R_f = 0.23 (24:1 dichloromethane/methanol), UV.
mp = 228.5 – 231.5 °C (dec).
IR (ATR) $\tilde{\upsilon}_{max}$ cm^{-1}: 3501 (s), 3475 (br, s), 3346 (s), 3059 (w), 2984 (w), 2961 (w), 2930 (m), 2869 (m), 1679 (s), 1650 (s), 1591 (m), 1473 (s), 1446 (m), 1433 (m), 1393 (m), 1363 (w), 1319 (m), 1276 (w), 1244 (m), 1208 (m), 1188 (w), 1130 (m), 1120 (m), 1091 (w), 1071 (w), 1058 (w), 1036 (m), 1011 (w), 987 (w), 918 (w), 895 (w), 862 (w), 805 (m), 568 (w), 524 (m).
^{1}H NMR (800 MHz, DMSO-d_6, 333 K) δ ppm: 9.61 (s, 1H, N-H), 7.69 (dd, *J* = 8.3, 2.4 Hz, 1H, C16-H), 7.42 (d, *J* = 2.4 Hz, 1H, C14-H), 7.14 (d, *J* = 8.3 Hz, 1H, C17-H), 4.10 (d, *J* = 2.7 Hz, 1H, O-H), 3.79 – 3.75 (m, 1H, C5-H), 2.48 – 2.40 (m, 2H, C10-H_2), 2.22 (td, *J* = 13.4, 4.6 Hz, 1H, C7-H), 2.06 – 2.01 (m, 1H, C9-H), 1.99 – 1.94 (m, 1H, C9-H), 1.85 – 1.82 (m, 1H, C4-H), 1.81 – 1.78 (m, 1H, C3-H), 1.60 – 1.53 (m, 3H, C4-H + C6-H_2), 1.35 (s, 3H, C8-H_3), 0.96 (dt, *J* = 14.2, 4.1 Hz, 1H, C7-H).

^{13}C NMR (201 MHz, DMSO-d_6, 333 K) δ ppm: 202.6 (C11), 176.7 (C1), 140.8 (C12), 136.5 (C13), 133.7 (C16), 129.4 (C14), 128.7 (C17), 118.1 (C15), 64.0 (C5), 44.5 (C3), 43.2 (C2), 42.9 (C10), 35.1 (C4), 30.6 (C9), 28.1 (C6), 23.0 (C7), 20.7 (C8).
LC-LRMS (ESI+) *m/z*: 365.93 found for $[M+H]^+$, 366.07 calcd. for $C_{17}H_{21}BrNO_3^+$, R_t = 1.07 min, purity (UV area): ≥95%.
HRMS (ESI+) *m/z*: 388.0516 found for $[M+Na]^+$, 388.0519 calcd. for $C_{17}H_{20}BrNNaO_3^+$ (Δ = -0.72 ppm).

Note: Peak broadening in the 1D ^{13}C NMR spectrum observed which is resolved by VT NMR (please see NMR spectra).

(6a*S,9*R**,10a*R**)-9-Hydroxy-2,6a-dimethyl-6a,7,8,9,10,10a,11,12-octahydro-5*H*-dibenzo[*b*,*g*]azonine-6,13-dione (11c)**

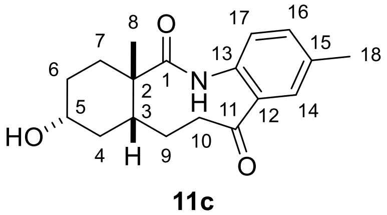

11c

Following general procedure F using compound **10e** (75.0 mg, 0.278 mmol), adding additional $NaIO_4$ (2.0 equiv.), stirring at room temperature for 23 hours, and eluting with 24:1 dichloromethane/methanol afforded compound **11c** (52.0 mg, 62% yield) as an off-white solid.

TLC: R_f = 0.15 (24:1 dichloromethane/methanol), UV.
mp = 224.0 – 227.0 °C (dec).
IR (ATR) $\tilde{\upsilon}_{\max}$ cm^{-1}: 3498 (s), 3460 (s), 3347 (s), 3053 (w), 3037 (w), 2984 (w), 2959 (w), 2931 (m), 2870 (m), 1675 (s), 1649 (s), 1606 (w), 1580 (w), 1483 (s), 1469 (s), 1445 (m), 1436 (m), 1411 (m), 1362 (w), 1332 (w), 1319 (m), 1274 (m), 1238 (m), 1207 (m), 1186 (w), 1154 (m), 1131 (w), 1091 (m), 1059 (w), 1046 (m), 1012 (m), 994 (w), 943 (w), 919 (w), 891 (w), 873 (w), 814 (w), 804 (w), 599 (w), 574 (w), 523 (w).
^{1}H NMR (800 MHz, DMSO-d_6, 348 K) δ ppm: 9.32 (s, 1H, N-H), 7.31 (dd, *J* = 8.0, 2.1 Hz, 1H, C16-H), 7.14 (d, *J* = 2.1 Hz, 1H, C14-H), 7.06 (d, *J* = 7.9 Hz, 1H, C17-H), 4.00 (d, *J* = 2.9 Hz, 1H, O-H), 3.78 – 3.75 (m, 1H, C5-H), 2.48 – 2.43 (m, 2H, C10-H_2), 2.33 (s, 3H, C18-H_3), 2.23 (td, *J* = 13.3, 4.3 Hz, 1H, C7-H), 2.08 – 2.03 (m, 1H, C9-H), 2.02 – 1.97 (m, 1H, C9-H), 1.84 – 1.79 (m, 2H, C3-H + C4-H), 1.65 – 1.61 (m, 1H, C6-H), 1.60 – 1.54 (m, 2H, C4-H + C6-H), 1.35 (s, 3H, C8-H_3), 0.99 (dt, *J* = 14.2, 4.4 Hz, 1H, C7-H).
^{13}C NMR (201 MHz, DMSO-d_6, 348 K) δ ppm: 204.1 (C11), 176.3 (C1), 139.0 (C12), 134.9 (C15), 134.5 (C13), 131.2 (C16), 127.1 (C14), 126.6 (C17), 64.2 (C5), 44.1 (C3), 42.9 (C2), 42.5 (C10), 35.1 (C4), 30.1 (C9), 28.4 (C6), 23.8 (C7), 21.0 (C8), 20.0 (C18).
LC-LRMS (ESI+) *m/z*: 302.17 found for $[M+H]^+$, 302.18 calcd. for $C_{18}H_{24}NO_3^+$, R_t = 1.03 min, purity (UV area): ≥95%.
HRMS (ESI+) *m/z*: 324.1578 found for $[M+Na]^+$, 324.1570 calcd. for $C_{18}H_{23}NNaO_3^+$ (Δ = 2.41 ppm).

Note: Peak broadening in the 1D ^{13}C NMR spectrum observed which is resolved by VT NMR (please see NMR spectra).

(6a*S,9*R**,10a*R**)-9-Hydroxy-2-methoxy-6a-methyl-6a,7,8,9,10,10a,11,12-octahydro-5*H*-dibenzo[*b*,*g*]azonine-6,13-dione (11d)**

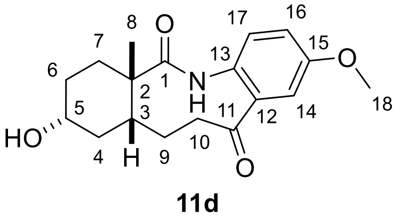

11d

Following general procedure F using compound **10f** (75.0 mg, 0.263 mmol), adding additional $NaIO_4$ (2.0 equiv.), stirring at room temperature for 25 hours, and eluting with 24:1 dichloromethane/methanol afforded compound **11d** (75.5 mg, 91% yield) as a grey solid.

TLC: R_f = 0.21 (24:1 dichloromethane/methanol), UV.
mp = 232.5 – 235.5 °C.
IR (ATR) $\tilde{\nu}_{\max}$ cm^{-1}: 3336 (s), 3280 (br, s), 3083 (w), 3071 (w), 2973 (w), 2930 (m), 2872 (m), 2840 (w), 1671 (s), 1601 (w), 1584 (w), 1494 (s), 1430 (m), 1410 (m), 1363 (w), 1324 (w), 1274 (m), 1251 (m), 1220 (m), 1161 (w), 1133 (w), 1119 (w), 1100 (w), 1060 (w), 1034 (s), 991 (w), 947 (w), 918 (w), 878 (w), 850 (w), 841 (w), 730 (w), 580 (m).
^{1}H NMR (800 MHz, DMSO-*d*$_6$, 333 K) δ ppm: 9.29 (s, 1H, N-H), 7.10 (d, *J* = 8.5 Hz, 1H, C17-H), 7.07 (dd, *J* = 8.6, 2.9 Hz, 1H, C16-H), 6.86 (d, *J* = 2.9 Hz, 1H, C14-H), 4.08 (d, *J* = 2.9 Hz, 1H, O-H), 3.78 (s, 3H, C18-H$_3$), 3.76 – 3-73 (m, 1H, C5-H), 2.49 – 2.44 (m, 2H, C10-H$_2$), 2.21 (ddd, *J* = 14.1, 12.4, 4.2 Hz, 1H, C7-H), 2.07 – 1.96 (m, 2H, C9-H$_2$), 1.84 – 1.78 (m, 2H, C3-H + C4-H), 1.64 – 1.60 (m, 1H, C6-H), 1.58 – 1.53 (m, 2H, C4-H + C6-H), 1.34 (s, 3H, C8-H$_3$), 0.98 (dt, *J* = 14.3, 4.4 Hz, 1H, C7-H).
^{13}C NMR (201 MHz, DMSO-*d*$_6$, 333 K) δ ppm: 204.1 (C11), 176.6 (C1), 157.1 (C15), 140.3 (C12), 130.0 (C13), 128.3 (C17), 116.6 (C16), 111.7 (C14), 64.3 (C5), 55.3 (C18), 44.1 (C3), 42.9 (C2), 42.5 (C10), 35.2 (C4), 30.1 (C9), 28.5 (C6), 23.9 (C7), 21.2 (C8).
LC-LRMS (ESI+) *m/z*: 318.30 found for [M+H]$^+$, 318.17 calcd. for $C_{18}H_{24}NO_4^+$, R_t = 0.90 min, purity (UV area): 97%.
HRMS (ESI+) *m/z*: 318.1700 found for [M+H]$^+$, 318.1700 calcd. for $C_{18}H_{24}NO_4^+$ (Δ = 0.00 ppm).

Note: Peak broadening in the 1D ^{13}C NMR spectrum observed which is resolved by VT NMR (please see NMR spectra).

(6a*S,9*R**,10a*R**)-9-Hydroxy-6a-methyl-2-(trifluoromethyl)-6a,7,8,9,10,10a,11,12-octahydro-5*H*-dibenzo[*b*,*g*]azonine-6,13-dione (11e)**

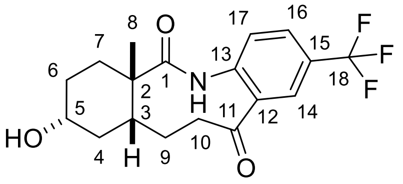

11e

Following general procedure F using compound **10g** (50.0 mg, 155 µmol), adding additional $NaIO_4$ (8.0 equiv.), stirring at room temperature for 44 hours and then at 50 °C for four hours, and eluting with 19:1 dichloromethane/methanol afforded compound **11e** (31.7 mg, 58% yield) as a white solid.

TLC: R_f = 0.20 (19:1 dichloromethane/methanol), UV.
mp = 210.0 – 213.0 °C (dec).
IR (ATR) $\tilde{\nu}_{\max}$ cm^{-1}: 3472 (s), 3326 (s), 3071 (w), 3011 (w), 2985 (w), 2934 (m), 2877 (m), 1685 (s), 1655 (s), 1613 (m), 1487 (s), 1472 (s), 1436 (w), 1415 (w), 1334 (s), 1319 (s), 1280 (w), 1268 (m), 1242 (s), 1215 (m), 1190 (w), 1160 (s), 1115 (s), 1069 (s), 1043 (m), 1034 (m), 1012 (w), 989 (w), 918 (w), 871 (w), 829 (m), 720 (w), 617 (w), 592 (w), 571 (w), 512 (w).
^{1}H NMR (800 MHz, DMSO-*d*$_6$, 348 K) δ ppm: 9.82 (s, 1H, N-H), 7.87 (dd, *J* = 8.2, 2.3 Hz, 1H, C16-H), 7.58 (d, *J* = 2.3 Hz, 1H, C14-H), 7.41 (d, *J* = 8.2 Hz, 1H, C17-H), 4.03 (d, *J* = 2.8 Hz, 1H, O-H), 3.82 – 3.78 (m, 1H, C5-H), 2.54 – 2.51 (m, 1H, C10-H), 2.45 (ddd, J = 13.5, 8.4, 1.9 Hz, 1H, C10-H), 2.28 (td, J = 13.3, 4.9 Hz, 1H, C7-H), 2.13 – 2.07 (m, 1H, C9-H), 2.03 – 1.98 (m, 1H, C9-H), 1.87 (ddd, *J* = 14.1, 5.6, 3.1 Hz, 1H, C4-H), 1.83 – 1.79 (m, 1H, C3-H), 1.63 – 1.55 (m, 3H, C4-H + C6-H$_2$), 1.40 (s, 3H, C8-H$_3$), 0.98 (dt, *J* = 14.6, 3.7 Hz, 1H, C7-H).
^{13}C NMR (201 MHz, DMSO-*d*$_6$, 348 K) δ ppm: 202.3 (C11), 176.7 (C1), 140.8 (C12), 139.1 (C13), 127.6 (q, *J* = 3.0 Hz, C16), 126.9 (C17), 125.8 (q, *J* = 32.5 Hz, C15), 123.60 (q, *J* = 272.1 Hz, C18), 123.55 (q, *J* = 3.8 Hz, C14), 63.8 (C5), 44.5 (C3), 43.3 (C2), 42.9 (C10), 34.9 (C4), 30.5 (C9), 27.9 (C6), 22.7 (C7), 20.3 (C8).
^{19}F NMR (377 MHz, DMSO-*d*$_6$) δ ppm: -60.8.
LC-LRMS (ESI+) *m/z*: 356.11 found for [M+H]$^+$, 356.15 calcd. for $C_{18}H_{21}F_3NO_3^+$, R_t = 1.25 min, purity (UV area): ≥95%.
HRMS (ESI+) *m/z*: 378.1295 found for [M+Na]$^+$, 378.1287 calcd. for $C_{18}H_{20}F_3NNaO_3^+$ (Δ = 2.06 ppm).

Note: Peak broadening in the 1D ^{13}C NMR spectrum observed which is resolved by VT NMR (please see NMR spectra).

(6a*S,9*R**,10a*R**)-2-Chloro-4-fluoro-9-hydroxy-6a-methyl-6a,7,8,9,10,10a,11,12-octahydro-5*H*-dibenzo[*b*,*g*]azonine-6,13-dione (11f)**

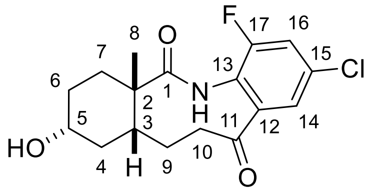

11f

Following general procedure F using compound **10i** (75.0 mg, 0.244 mmol), adding additional $NaIO_4$ (8.0 equiv.), stirring at room temperature for 44.5 hours, and eluting with 24:1 dichloromethane/methanol afforded compound **11f** (38.8 mg, 47% yield) as an off-white solid.

TLC: R_f = 0.13 (24:1 dichloromethane/methanol), UV.
mp = 213.0 – 216.0 °C (dec).
IR (ATR) $\tilde{\upsilon}_{\max}$ cm^{-1}: 3517 (m), 3477 (s), 3345 (s), 3084 (w), 2984 (w), 2971 (w), 2935 (m), 2893 (w), 2871 (w), 1686 (s), 1656 (s), 1607 (w), 1685 (m), 1479 (s), 1466 (s), 1435 (m), 1416 (s), 1335 (w), 1321 (m), 1279 (s), 1241 (m), 1207 (m), 1129 (w), 1107 (w), 1084 (m), 1057 (m), 1033 (w), 992 (w), 958 (m), 915 (m), 885 (w), 855 (m), 559 (w), 505 (w).
^{1}H NMR (800 MHz, DMSO-d_6, 348 K) δ ppm: 9.38 (s, 1H, N-H), 7.64 (dd, *J* = 9.2, 2.3 Hz, 1H, C16-H), 7.19 (dd, *J* = 2.4, 1.0 Hz, 1H, C14-H), 4.04 (s, 1H, O-H), 3.79 – 3.75 (m, 1H, C5-H), 2.54 – 2.43 (m, 2H, C10-H_2), 2.22 (t, *J* = 11.8 Hz, 1H, C7-H), 2.07 – 1.99 (m, 2H, C9-H_2), 1.90 – 1.81 (m, 2H, C3-H + C4-H), 1.65 – 1.61 (m, 1H, C6-H), 1.61 – 1.55 (m, 2H, C4-H + C6-H), 1.37 (s, 3H, C8-H_3), 1.05 – 0.98 (m, 1H, C7-H).
^{13}C NMR (201 MHz, DMSO-d_6, 348 K) δ ppm: 201.4 (C11), 176.6 (C1), 157.1 (d, *J* = 250.5 Hz, C17), 142.3 (C15), 130.8 (d, *J* = 7.5 Hz, C12), 124.1 (d, *J* = 14.9 Hz, C13), 122.5 (d, *J* = 3.0 Hz, C14), 118.2 (d, *J* = 24.3 Hz, C16), 64.0 (C5), 43.8 (C3), 43.2 (C2), 42.2 (C10), 34.9 (C4), 30.1 (C9), 28.2 (C6), 23.5 (C7), 20.8 (C8).
^{19}F NMR (377 MHz, DMSO-d_6) δ ppm: -121.6.
LC-LRMS (ESI+) *m/z*: 340.02 found for $[M+H]^+$, 340.11 calcd. for $C_{17}H_{20}ClFNO_3^+$, R_t = 1.18 min, purity (UV area): >95%.
HRMS (ESI+) *m/z*: 362.0934 found for $[M+Na]^+$, 362.0930 calcd. for $C_{17}H_{19}ClFNNaO_3^+$ (Δ = 1.19 ppm).

Note: The peak for the C10-H_2 protons at 2.54 – 2.43 ppm in the 1D ^{1}H NMR spectrum overlaps with the dimethyl sulfoxide-d_6 solvent peak, thus increasing the integral, but the chemical shift and integral were confirmed by correlations in the 2D ^{1}H-^{1}H COESY and ^{1}H-^{13}C HSQC and HMBC spectra. Peak broadening in the 1D ^{13}C NMR spectrum observed which is resolved by VT NMR (please see NMR spectra). The missing characteristic $^3J_{C-F}$ coupling for the C15 carbon is likely due to peak broadening.

(6a*S,9*R**,10a*R**)-9-Hydroxy-1,3,6a-trimethyl-6a,7,8,9,10,10a,11,12-octahydro-5*H*-dibenzo[*b*,*g*]azonine-6,13-dione (11g)**

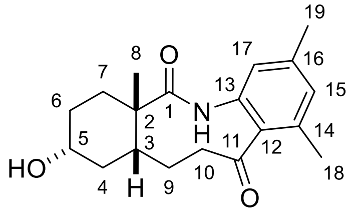

11g

Following general procedure F using compound **10j** (45.0 mg, 159 µmol), adding additional $NaIO_4$ (2.0 equiv.), stirring at room temperature for 22 hours, and eluting with 24:1 dichloromethane/methanol afforded compound **11g** (42.3 mg, 84% yield) as a white solid.

TLC: R_f = 0.19 (24:1 dichloromethane/methanol), UV.
mp = 237.0 – 240.0 °C.
IR (ATR) $\tilde{\upsilon}_{\max}$ cm^{-1}: 3470 (s), 3330 (s), 3082 (w), 3043 (w), 3013 (w), 2966 (w), 2920 (m), 2966 (m), 1733 (w), 1679 (s), 1650 (s), 1611 (m), 1569 (w), 1505 (s), 1463 (m), 1444 (m), 1429 (m), 1385 (w),

1364 (w), 1333 (m), 1324 (m), 1311 (w), 1246 (m), 1222 (m), 1190 (m). 1157 (m), 1117 (m), 1094 (w), 1046 (w), 1032 (m), 1008 (w), 982 (w), 944 (w), 919 (w), 894 (w), 882 (w), 857 (w), 617 (w), 541 (w).
^{1}H NMR (800 MHz, DMSO-*d*$_6$, 333 K) δ ppm: 9.22 (s, 1H, N-H), 6.94 (s, 1H, C15-H), 6.82 (s, 1H, C17-H), 4.08 (s, 1H, O-H), 3.76 – 3.70 (m, 1H, C5-H), 2.45 – 2.34 (m, 2H, C10-H$_2$), 2.30 (s, 3H, C19-H$_3$), 2.20 (s, 3H, C18-H$_3$), 2.17 – 2.09 (m, 2H, C7-H + C9-H), 1.94 (t, J = 13.2 Hz, 1H, C9-H), 1.85 – 1.75 (m, 2H, C3-H + C4-H), 1.67 – 1.62 (m, 1H, C6-H), 1.59 – 1.50 (m, 2H, C4-H + C6-H), 1.31 (s, 3H, C8-H$_3$), 1.01 (dt, J = 14.6, 4.7 Hz, 1H, C7-H).
^{13}C NMR (151 MHz, DMSO-*d*$_6$, 368 K) δ ppm: 205.1 (C11), 175.7 (C1), 138.8 (C14), 136.6 (C16), 135.6 (C12), 134.6 (C13), 128.3 (C15), 125.3 (C17), 64.6 (C5), 43.4 (C3), 42.6 (C2), 42.3 (C10), 35.1 (C4), 29.5 (C9), 28.7 (C6), 24.9 (C7), 21.4 (C8), 19.9 (C19), 18.2 (C18).
LC-LRMS (ESI+) *m/z*: 316.11 found for [M+H]$^+$, 316.19 calcd. for $C_{19}H_{26}NO_3^+$, R_t = 1.03 min, purity (UV area): 97%.
HRMS (ESI+) *m/z*: 338.1728 found for [M+Na]$^+$, 338.1727 calcd. for $C_{19}H_{25}NNaO_3^+$ (Δ = 0.41 ppm).

Note: Peak broadening in the 1D ^{13}C NMR spectrum observed which is resolved by VT NMR (please see NMR spectra).

Synthesis of quinolone analogue

(2*R,4a*S**,11a*R**)-2-Hydroxy-4a,8-dimethyl-1,2,3,4,4a,5,11,11a-octahydro-10*H*-indeno[1,2-*b*] quinolin-10-one (12)**

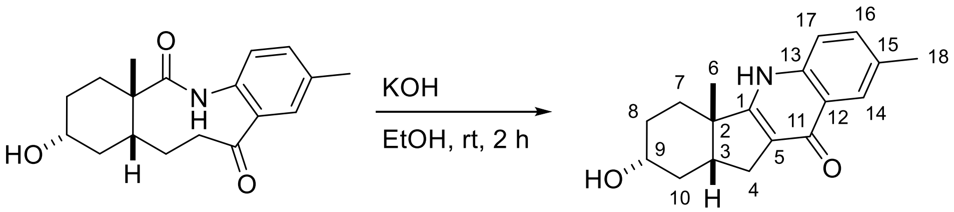

11c

12
78%

To a solution of compound **11c** (45.0 mg, 149 µmol, 1.0 equiv.) in absolute ethanol (2.4 mL) at room temperature was added KOH (10.8 mg, 193 µmol, 1.3 equiv.). The reaction mixture was stirred at room temperature for two hours. The reaction mixture was neutralised with saturated aqueous NH_4Cl and extracted with 9:1 chloroform/isopropanol (3 × 10 mL). The combined organic phases were washed with brine (10 mL), dried over anhydrous $MgSO_4$, and the solvent removed under reduced pressure to yield a crude light-orange solid. The crude product was purified by flash column chromatography on BÜCHI® system using FlashPure EcoFlex Silica (12 g; 49:1 to 0:1 dichloromethane/methanol; flow rate = 20 mL/min) to afford compound **12** (32.8 mg, 78% yield) as a white solid.

TLC: R_f = 0.22 (23:2 dichloromethane/methanol), UV.
dec pt = 285.0 – 290.0 °C.
IR (ATR) $\tilde{\upsilon}_{\max}$ cm^{-1}: 3243 (br, s), 3148 (m), 3119 (m), 3062 (m), 3054 (m), 2925 (s), 2854 (s), 1635 (m), 1607 (m), 1578 (s), 1557 (s), 1498 (s), 1479 (s), 1359 (m), 1253 (m), 1154 (w), 1118 (w), 1106 (w), 1086 (w), 1061 (m), 1034 (m), 818 (m), 788 (w), 733 (w), 695 (w), 572 (w), 548 (w).
^{1}H NMR (400 MHz, DMSO-*d*$_6$) δ ppm: 11.40 (br s, 1H, N-H), 7.88 (s, 1H, C14-H), 7.55 (d, *J* = 8.4 Hz, 1H, C17-H), 7.42 (dd, *J* = 8.5, 2.1 Hz, 1H, C16-H), 4.43 (d, *J* = 4.5 Hz, 1H, O-H), 3.50 – 3.40 (m, 1H, C9-H), 2.77 (dd, *J* = 14.8, 6.2 Hz, 1H, C4-H), 2.43 – 2.29 (m, 5H, C4-H + C7-H + C18-H$_3$), 2.11 – 2.03 (m, 1H, C3-H), 1.85 – 1.77 (m, 1H, C10-H), 1.73 – 1.65 (m, 1H, C8-H), 1.48 (ddd, *J* = 14.4, 12.3, 3.7 Hz, 1H, C7-H), 1.17 (s, 3H, C6-H$_3$), 1.03 – 0.91 (m, 2H, C8-H + C10-H).
^{13}C NMR (101 MHz, DMSO-*d*$_6$) δ ppm: 174.8 (C11), 158.6 (C1), 138.6 (C13), 132.0 (C16), 131.7 (C15), 125.4 (C12), 124.0 (C14), 118.3 (C17), 117.0 (C5), 66.9 (C9), 46.7 (C2), 43.2 (C3), 38.6 (C10), 32.3 (C4), 31.9 (C8), 30.1 (C7), 26.0 (C6), 20.8 (C18).
^{1}H NMR (400 MHz, CD_3OD) δ ppm: 8.09 – 8.05 (m, 1H, C14-H), 7.61 (d, *J* = 8.5 Hz, 1H, C17-H), 7.51 (dd, *J* = 8.6, 2.1 Hz, 1H, C16-H), 3.63 (tt, *J* = 10.2, 3.8 Hz, 1H, C9-H), 3.00 (dd, *J* = 15.1, 6.2 Hz, 1H, C4-H), 2.57 (dd, *J* = 15.1, 1.6 Hz, 1H, C4-H), 2.51 – 2.44 (m, 4H, C7-H + C18-H$_3$), 2.25 (dtd, *J* = 12.3, 6.1, 1.6 Hz, 1H, C3-H), 2.03 – 1.95 (m, 1H, C10-H), 1.91 – 1.82 (m, 1H, C8-H), 1.66 (ddd, *J* = 14.8, 12.7, 3.8 Hz, 1H, C7-H), 1.27 (s, 3H, C6-H$_3$), 1.17 – 1.06 (m, 2H, C8-H + C10-H).

^{13}C NMR (101 MHz, CD_3OD) δ ppm: 178.0 (C11), 162.3 (C1), 140.0 (C13), 135.1 (C15), 134.0 (C16), 126.3 (C12), 125.1 (C14), 119.5 (C5), 119.3 (C17), 69.3 (C9), 48.5 (C2), 45.3 (C3), 39.6 (C10), 33.2 (C4), 32.8 (C8), 31.5 (C7), 26.7 (C6), 21.2 (C18).
LC-LRMS (ESI+) *m/z*: 284.04 found for $[M+H]^+$, 284.16 calcd. for $C_{18}H_{22}NO_2^+$, R_t = 1.08 min, purity (UV area): 100%.
HRMS (ESI+) *m/z*: 284.1646 found for $[M+H]^+$, 284.1645 calcd. for $C_{18}H_{22}NO_2^+$ (Δ = 0.43 ppm).

Note: The peak for the C2 carbon at 48.5 ppm in the 1D ^{13}C NMR spectrum overlaps with the methanol-*d*$_4$ solvent peak but the chemical shift was confirmed by correlations in the 2D ^{1}H-^{13}C HMBC spectrum.

Synthesis of spiropseudoindoxyl analogue

(1*R,3a*R**,5*R**,7a*S**)-5-Hydroxy-5',7a-dimethyl-2,3,3a,4,5,6,7,7a-octahydrospiro[indene-1,2'-indolin]-3'-one (13)**

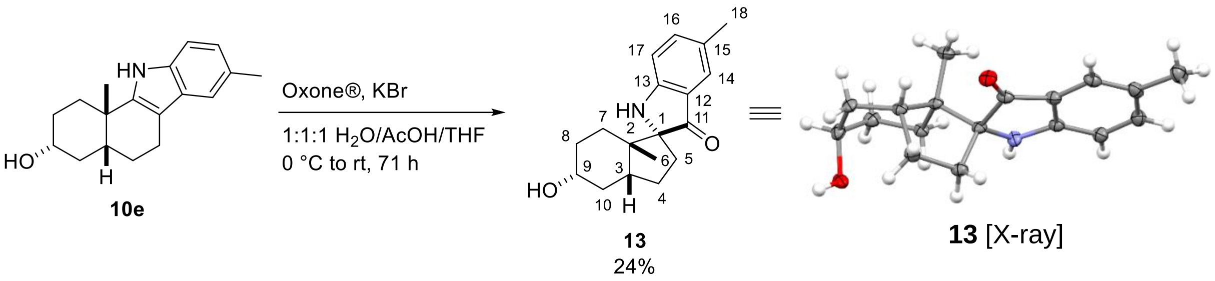

To a solution of compound **10e** (75.0 mg, 0.278 mmol, 1.00 equiv.) and KBr (1.7 mg, 14 µmol, 0.05 equiv.) in 1:1:1 deionised water/glacial acetic acid/tetrahydrofuran (2.8 mL) at 0 °C was added Oxone® (MW = 307.38) (103 mg, 335 mmol, 1.20 equiv.) portionwise over 15 minutes. The reaction mixture was stirred at room temperature for seven hours. Then additional KBr (1.7 mg, 14 µmol, 0.05 equiv.) and Oxone® (25.7 mg, 86.6 µmol, 0.30 equiv.) were added. Then the reaction mixture was stirred at room temperature for 64 hours. The reaction mixture was quenched with saturated aqueous $NaHCO_3$ (5 mL) and saturated aqueous Na_2SO_3 (5 mL) and extracted with ethyl acetate (4 × 10 mL). The combined organic phases were washed with brine (5 mL), dried over anhydrous Na_2SO_4, and the solvent removed under reduced pressure to yield a crude brown solid. The crude product was purified by flash column chromatography on silica gel (4:1 to 1:2 *n*-heptane/ethyl acetate) to afford compound **13** (19.3 mg, 24% yield) as an orange solid.

Note: The ketolactam side-product (**11c**; 8.9 mg, 11% yield) was isolated in separate fractions.

TLC: R_f = 0.17 (3:2 *n*-heptane/ethyl acetate), UV.
mp = 228.3 – 230.2 °C (dec).
IR (ATR) $\tilde{\upsilon}_{\max}$ cm^{-1}: 3446 (br, m), 3299 (s), 2920 (m), 2873 (m), 1651 (s), 1624 (s), 1585 (w), 1503 (m), 1464 (m), 1449 (m), 1437 (m), 1417 (w), 1365 (m), 1331 (w), 1288 (m), 1272 (m), 1238 (m), 1213 (m), 1152 (m), 1130 (m), 1105 (w), 1084 (m), 1060 (w), 1047 (w), 1009 (m), 983 (w), 942 (w), 916 (w), 818 (w), 806 (w), 705 (w), 594 (w), 571 (w), 554 (w).
^{1}H NMR (400 MHz, DMSO-*d*$_6$) δ ppm: 7.20 (dd, *J* = 8.4, 1.9 Hz, 1H, C16-H), 7.10 (d, *J* = 1.9 Hz, 1H, C14-H), 6.86 (s, 1H, N-H), 6.74 (d, *J* = 8.3 Hz, 1H, C17-H), 4.35 (br s, 1H, O-H), 3.89 – 3.83 (m, 1H, C9-H), 2.49 – 2.39 (m, 2H, C3-H + C4-H), 2.18 (s, 3H, C18-H_3), 1.94 – 1.78 (m, 2H, C5-H + C7-H), 1.76 – 1.52 (m, 5H, C4-H + C5-H + C8-H_2 + C10-H), 1.46 (ddd, *J* = 14.5, 5.3, 3.5 Hz, 1H, C10-H), 1.08 (dt, *J* = 13.5, 3.6 Hz, 1H, C7-H), 0.63 (s, 3H, C6-H_3).
^{13}C NMR (101 MHz, DMSO-*d*$_6$) δ ppm: 206.1 (C11), 159.9 (C13), 137.9 (C16), 125.2 (C15), 122.9 (C14), 119.3 (C12), 111.4 (C17), 79.2 (C1), 64.0 (C9), 44.9 (C2), 38.5 (C3), 30.1 (C10), 29.9 (C5), 27.7 (C8), 25.8 (C4), 23.9 (C7), 20.0 (C18), 17.0 (C6).
^{1}H NMR (400 MHz, CD_3OD) δ ppm: 7.24 (dd, *J* = 8.4, 1.9 Hz, 1H, C16-H), 7.18 (d, *J* = 1.9 Hz, 1H, C14-H), 6.76 (d, *J* = 8.4 Hz, 1H, C17-H), 4.01 (quint, *J* = 3.0 Hz, 1H, C9-H), 2.62 – 2.41 (m, 2H, C3-H + C4-H), 2.23 (s, 3H, C18-H_3), 2.08 – 1.86 (m, 3H, C4-H + C5-H + C7-H), 1.83 – 1.68 (m, 4H, C5-H + C8-H_2 + C10-H), 1.63 (ddd, *J* = 14.9, 5.7, 3.6 Hz, 1H, C10-H), 1.25 – 1.18 (m, 1H, C7-H), 0.75 (s, 3H, C6-H_3).
^{13}C NMR (101 MHz, CD_3OD) δ ppm: 210.0 (C11), 162.2 (C13), 140.0 (C16), 127.7 (C15), 124.0 (C14), 120.8 (C12), 112.7 (C17), 81.3 (C1), 67.0 (C9), 46.7 (C2), 40.3 (C3), 31.42 (C5), 31.40 (C10), 28.7 (C8), 27.4 (C4), 25.2 (C7), 20.5 (18), 17.7 (C6).
LC-LRMS (ESI+) *m/z*: 286.04 found for $[M+H]^+$, 286.18 calcd. for $C_{18}H_{24}NO_2^+$, R_t = 1.47 min, purity (UV area): 95%.

HRMS (ESI+) *m/z*: 286.1801 found for $[M+H]^+$, 286.1802 calcd. for $C_{18}H_{24}NO_2^+$ (Δ = -0.27 ppm).
X-ray (single-crystal): Crystal was grown by vapour diffusion (solvent = dichloromethane/methanol; antisolvent = *n*-pentane) until equilibrium then followed by mixed solvent slow evaporation.

Note: LC-LRMS indicates a purity of 95% (please see LC-LRMS trace) but unknown impurities are observed in the 1D ^1H NMR spectrum. The peak for the C3-H and C4-H protons at 2.49 – 2.39 ppm in the 1D ^1H NMR spectrum overlaps with the dimethyl sulfoxide-d_6 solvent peak, thus increasing the integral, but the chemical shift and integral were confirmed by correlations in the 2D ^1H-^1H COESY and ^1H-^{13}C HSQC and HMBC spectra. The relative stereochemistry was determined by the observed NOEs between the amine (N-H) proton and the C7-H_2 protons in the 2D ^1H-^1H NOESY spectrum (please see NMR spectra) and ultimately confirmed by the X-ray crystal structure.

Synthesis of hydroxyindolenine analogues

General procedure G

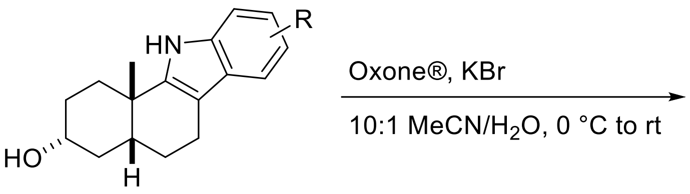

10 **14**

To a solution of the appropriate indole analogue (**10**; 1.00 equiv.) and KBr (0.05 equiv.) in 10:1 acetonitrile/deionised water (0.10 M **10**) at 0 °C was added Oxone® (MW = 307.38) (1.20 equiv.) portionwise over 15 minutes. The reaction mixture was stirred at room temperature for 19 hours. Additional KBr (0.05 equiv.) and Oxone® (0.30 equiv.) were added after 15 hours during the reaction. The reaction mixture was quenched with saturated aqueous $NaHCO_3$ and saturated aqueous Na_2SO_3 and extracted with ethyl acetate (4×). The combined organic phases were washed with brine, dried over anhydrous Na_2SO_4, and the solvent removed under reduced pressure to yield the crude products. The crude products were purified by flash column chromatography on silica gel to afford the hydroxyindolenine analogues (**14**).

(3*R,4a*R**,6a*R**,11b*S**)-8,11b-Dimethyl-1,2,3,4,4a,5,6,11b-octahydro-6a*H*-benzo[*a*]carbazole-3,6a-diol (14a)**

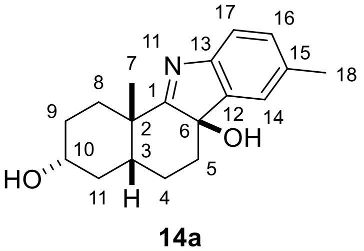

14a

Following general procedure G using compound **10e** (75.0 mg, 0.278 mmol) and eluting with 3:2 to 1:2 *n*-heptane/ethyl acetate afforded compound **14a** (13.1 mg, 16% yield) as a pale-orange solid.

Note: The ketolactam side-product (**11c**; 12.1 mg, 14% yield) and the spirooxepinoindole side-product (**15e**; 6.5 mg, 9% yield) were isolated in separate fractions.

TLC: R_f = 0.19 (3:2 ethyl acetate/*n*-heptane), UV.
mp = 212.0 – 215.0 °C.
IR (ATR) $\tilde{\upsilon}_{\max}$ cm^{-1}: 3290 (br, s), 3210 (br, m), 3053 (w), 3005 (w), 2970 (m), 2948 (m), 2922 (s), 2873 (s), 1739 (w), 1696 (w), 1667 (w), 1633 (w), 1599 (w), 1574 (m), 1490 (w), 1474 (m), 1438 (m), 1367 (m), 1325 (w), 1288 (w), 1274 (w), 1242 (w), 1208 (w), 1197 (w), 1159 (w), 1151 (w), 1135 (w), 1116 (w), 1097 (w), 1084 (w), 1068 (w), 1035 (m), 1026 (m), 970 (w), 942 (w), 916 (w), 816 (m), 786 (w), 721 (w), 702 (w), 609 (w), 595 (w), 581 (w).
^{1}H NMR (400 MHz, DMSO-d_6) δ ppm: 7.32 (d, J = 7.7 Hz, 1H, C17-H), 7.14 (d, J = 1.7 Hz, 1H, C14-H), 7.09 (dd, J = 7.8, 1.8 Hz, 1H, C16-H), 5.58 (s, 1H, C6-O-H), 4.37 (d, J = 4.7 Hz, 1H, C10-O-H), 3.42 – 3.32 (m, 1H, C10-H), 2.45 – 2.30 (m, 5H, C18-H_3 + C4-H + C8-H), 2.18 (dt, J = 13.9, 3.2 Hz, 1H, C5-

H), 1.87 – 1.79 (m, 1H, C3-H), 1.74 – 1.55 (m, 2H, C9-H_2), 1.44 – 1.21 (m, 6H, C4-H + C5-H + C7-H_3 + C11-H), 1.15 (td, *J* = 13.5, 4.2 Hz, 1H, C8-H), 0.88 (td, *J* = 12.8, 10.8 Hz, 1H, C11-H).
^{13}C NMR (101 MHz, DMSO-*d*$_6$) δ ppm: 186.6 (C1), 150.3 (C13), 143.4 (C12), 134.4 (C15), 128.8 (C16), 122.6 (C14), 119.5 (C17), 82.1 (C6), 69.1 (C10), 44.6 (C3), 40.5 (C2), 37.4 (C11), 35.7 (C8), 33.6 (C5), 32.3 (C9), 25.4 (C7), 23.5 (C4), 20.9 (C18).
^{1}H NMR (400 MHz, CD_3OD) δ ppm: 7.34 (d, *J* = 7.8 Hz, 1H, C17-H), 7.20 – 7.17 (m, 1H, C14-H), 7.16 – 7.12 (m, 1H, C16-H), 3.61 – 3.52 (m, 1H, C10-H), 2.63 – 2.47 (m, 2H, C4-H + C8-H), 2.37 (s, 3H, C18-H_3), 2.32 – 2.26 (m, 1H, C5-H), 2.00 – 1.92 (m, 1H, C3-H), 1.83 – 1.72 (m, 2H, C9-H_2), 1.61 – 1.46 (m, 5H, C5-H + C7-H_3 + C11-H), 1.42 – 1.26 (m, 2H, C4-H + C8-H), 1.06 (td, *J* = 13.0, 11.1 Hz, 1H, C11-H).
^{13}C NMR (101 MHz, CD_3OD) δ ppm: 189.1 (C1), 151.0 (C13), 143.9 (C12), 137.2 (C15), 130.5 (C16), 123.8 (C14), 120.4 (C17), 83.9 (C6), 71.5 (C10), 46.7 (C3), 42.4 (C2), 38.4 (C11), 36.9 (C8), 34.5 (C5), 33.3 (C9), 26.1 (C7), 24.9 (C4), 21.4 (C18).
LC-LRMS (ESI+) *m/z*: 286.04 found for $[M+H]^+$, 286.18 calcd. for $C_{18}H_{24}NO_2^+$, R_t = 1.08 min, purity (UV area): ≥95%.
HRMS (ESI+) *m/z*: 286.1810 found for $[M+H]^+$, 286.1802 calcd. for $C_{18}H_{24}NO_2^+$ (Δ = 2.88 ppm).

Note: The peak for the C10-H proton at 3.42 – 3.32 ppm in the 1D ^{1}H NMR spectrum in dimethyl sulfoxide-*d*$_6$ overlaps with the H_2O solvent peak, thus increasing the integral, but the chemical shift and integral were confirmed by correlations in the 2D ^{1}H-^{1}H COESY and ^{1}H-^{13}C HSQC and HMBC spectra. The relative stereochemistry was determined by an observed NOE between the hydroxy (C6-O-H) proton and the methyl (C7-H_3) protons in the 2D ^{1}H-^{1}H NOESY spectrum (please see NMR spectra).

(3*R,4a*R**,6a*R**,11b*S**)-8-Methoxy-11b-methyl-1,2,3,4,4a,5,6,11b-octahydro-6a*H*-benzo[*a*]carbazole-3,6a-diol (14b)**

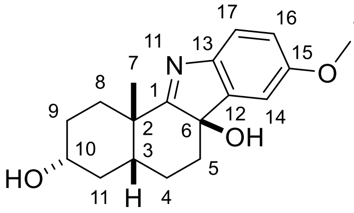

14b

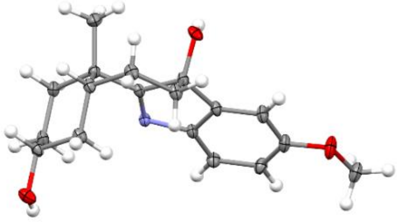

14b [X-ray]

Following general procedure G using compound **10f** (75.0 mg, 0.263 mmol) and eluting with 3:1 *n*-hexane/ethyl acetate afforded compound **14b** (16.3 mg, 21% yield) as a light-yellow solid.

TLC: R_f = 0.21 (3:1 *n*-hexane/ethyl acetate), UV.
mp = 200.5 – 203.5 °C.
IR (ATR) $\tilde{\upsilon}_{\max}$ cm^{-1}: 3293 (br, m), 3062 (w), 3005 (w), 2924 (m), 2896 (w), 2872 (m), 2835 (w), 1600 (w), 1573 (w),1476 (m), 1458 (m), 1432 (m), 1367 (m), 1321 (w), 1285 (m), 1274 (m), 1240 (m), 1219 (m), 1194 (m), 1173 (m), 1139 (m), 1117 (m), 1109 (m), 1098 (m), 1084 (w), 1070 (w), 1036 (s), 1024 (s), 971 (m), 940 (m), 924 (m), 917 (m), 877 (w), 843 (w), 819 (m), 803 (m), 788 (m), 778 (m), 672 (w), 608 (w).
^{1}H NMR (800 MHz, DMSO-*d*$_6$) δ ppm: 7.35 (d, *J* = 8.3 Hz, 1H, C17-H), 6.92 (d, *J* = 2.6 Hz, 1H, C14-H), 6.83 (dd, *J* = 8.3, 2.6 Hz, 1H, C16-H), 5.63 (s, 1H, C6-O-H), 4.36 (d, *J* = 4.9 Hz, 1H, C10-O-H), 3.75 (s, 3H, C18-H_3), 3.40 – 3.35 (m, 1H, C10-H), 2.40 (tt, *J* = 13.7, 3.9 Hz, 1H, C4-H), 2.34 (dt, *J* = 13.8, 3.6 Hz, 1H, C8-H), 2.17 (dt, *J* = 14.1, 3.1 Hz, 1H, C5-H), 1.85 – 1.80 (m, 1H, C3-H), 1.72 – 1.65 (m, 1H, C9-H), 1.61 – 1.56 (m, 1H, C9-H), 1.40 (s, 3H, C7-H_3), 1.38 – 1.31 (m, 2H, C5-H + C11-H), 1.27 – 1.22 (m, 1H, C4-H), 1.14 (td, *J* = 13.7, 4.1 Hz, 1H, C8-H), 0.89 (q, *J* = 12.2 Hz, 1H, C11-H).
^{13}C NMR (201 MHz, DMSO-*d*$_6$) δ ppm: 185.5 (C1), 157.6 (C15), 146.0 (C13), 144.8 (C12), 120.2 (C17), 112.9 (C16), 108.6 (C14), 82.4 (C6), 69.1 (C10), 55.5 (C18), 44.6 (C3), 40.5 (C2), 37.4 (C11), 35.7 (C8), 33.5 (C5), 32.3 (C9), 25.4 (C7), 23.5 (C4).
^{15}N NMR (81 MHz, DMSO-*d*$_6$) δ ppm: 309.9.
^{1}H NMR (400 MHz, CD_3OD) δ ppm: 7.37 (d, *J* = 8.4 Hz, 1H, C17-H), 6.95 (d, *J* = 2.5 Hz, 1H, C14-H), 6.87 (dd, *J* = 8.4, 2.6 Hz, 1H, C16-H), 3.81 (s, 3H, C18-H_3), 3.61 – 3.52 (m, 1H, C10-H), 2.62 – 2.45 (m, 2H, C4-H + C8-H), 2.30 – 2.23 (m, 1H, C5-H), 1.95 (d, *J* = 13.0 Hz, 1H, C3-H), 1.83 – 1.72 (m, 2H, C9-H_2), 1.62 – 1.45 (m, 5H, C5-H + C7-H_3 + C11-H), 1.43 – 1.25 (m, 2H, C4-H + C8-H), 1.07 (q, *J* = 12.3 Hz, 1H, C11-H).

^{13}C NMR (101 MHz, CD_3OD) δ ppm: 187.9 (C1), 160.3 (C15), 146.6 (C13), 145.3 (C12), 121.2 (C17), 114.6 (C16), 109.7 (C14), 84.1 (C6), 71.5 (C10), 56.2 (C18), 46.7 (C3), 42.3 (C2), 38.3 (C11), 36.9 (C8), 34.5 (C5), 33.3 (C9), 26.2 (C7), 24.9 (C4).
LC-LRMS (ESI+) *m/z*: 302.06 found for $[M+H]^+$, 302.18 calcd. for $C_{18}H_{24}NO_3^+$, R_t = 0.94 min, purity (UV area): ≥97%.
HRMS (ESI+) *m/z*: 302.1750 found for $[M+H]^+$, 302.1751 calcd. for $C_{18}H_{24}NO_3^+$ (Δ = -0.25 ppm).
X-ray (single-crystal): Crystal was grown by vapour diffusion (solvent = acetone; antisolvent = chloroform) until equilibrium then followed by mixed solvent slow evaporation.

Note: The relative stereochemistry was determined by an observed NOE between the hydroxy (C6-O-H) proton and the methyl (C7-H_3) protons in the 2D ^{1}H-^{1}H NOESY spectrum (please see NMR spectra) and ultimately confirmed by the X-ray crystal structure.

(3*R,4a*R**,6a*R**,11b*S**)-7,9,11b-Trimethyl-1,2,3,4,4a,5,6,11b-octahydro-6a*H*-benzo[*a*]carbazole-3,6a-diol (14c)**

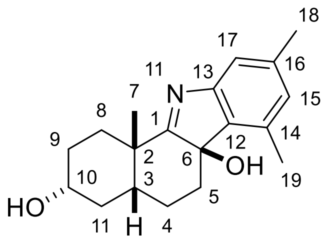

14c

Following general procedure G using compound **10j** (75.0 mg, 0.265 mmol) and eluting with 3:2 ethyl acetate/*n*-heptane afforded compound **14c** (23.2 mg, 29% yield) as an off-white solid.

TLC: R_f = 0.20 (3:2 ethyl acetate/*n*-heptane), UV.
mp = 220.0 – 225.0 °C.
IR (ATR) $\tilde{\upsilon}_{\max}$ cm^{-1}: 3291 (br, m), 3026 (w), 2949 (m), 2927 (m), 2862 (m), 1623 (w), 1600 (w), 1577 (m), 1477 (w), 1437 (m), 1383 (m), 1368 (m), 1236 (m), 1181 (w), 1144 (m), 1115 (w), 1096 (m), 1071 (w), 1029 (s), 961 (w), 937 (m), 916 (w), 881 (w), 850 (s), 829 (w), 720 (m), 699 (m), 650 (w).
^{1}H NMR (800 MHz, DMSO-*d*$_6$) δ ppm: 7.06 (s, 1H, C17-H), 6.73 (s, 1H, C15-H), 5.49 (d, *J* = 2.0 Hz, 1H, C6-O-H), 4.36 (d, *J* = 4.7 Hz, 1H, C10-O-H), 3.41 – 3.35 (m, 1H, C10-H), 2.42 – 2.33 (m, 3H, C4-H + C5-H + C8-H), 2.31 (s, 3H, C19-H_3), 2.28 (s, 3H, C18-H_3), 1.84 – 1.79 (m, 1H, C3-H), 1.71 – 1.65 (m, 1H, C9-H), 1.61 – 1.56 (m, 1H, C9-H), 1.43 – 1.35 (m, 4H, C7-H_3 + C5-H), 1.34 – 1.30 (m, 1H, C11-H), 1.26 – 1.22 (m, 1H, C4-H), 1.13 (td, *J* = 13.7, 4.1 Hz, 1H, C8-H), 0.88 (td, *J* = 12.9, 10.9 Hz, 1H, C11-H).
^{13}C NMR (201 MHz, DMSO-*d*$_6$) δ ppm: 187.5 (C1), 152.9 (C13), 137.6 (C16), 137.3 (C12), 133.0 (C14), 127.6 (C15), 118.2 (C17), 82.8 (C6), 69.1 (C10), 44.3 (C3), 40.5 (C2), 37.4 (C11), 35.8 (C8), 32.2 (C9), 32.1 (C5), 25.4 (C7), 23.4 (C4), 21.0 (C18), 16.6 (C19).
^{15}N NMR (81 MHz, DMSO-*d*$_6$) δ ppm: 311.1.
^{1}H NMR (400 MHz, CD_3OD) δ ppm: 7.11 – 7.08 (m, 1H, C17-H), 6.81 – 6.78 (m, 1H, C15-H), 3.62 – 3.52 (m, 1H, C10-H), 2.61 – 2.47 (m, 3H, C4-H + C5-H + C8-H), 2.39 (s, 3H, C19-H_3), 2.32 (s, 3H, C18-H_3), 1.94 (dq, *J* = 13.5, 3.5 Hz, 1H, C3-H), 1.83 – 1.72 (m, 2H, C9-H_2), 1.67 – 1.58 (m, 1H, C5-H), 1.55 – 1.46 (m, 4H, C7-H_3 + C11-H), 1.41 – 1.26 (m, 2H, C4-H + C8-H), 1.07 (td, *J* = 13.0, 11.1 Hz, 1H, C11-H).
^{13}C NMR (101 MHz, CD_3OD) δ ppm: 190.0 (C1), 153.5 (C13), 140.0 (C16), 137.8 (C12), 135.1 (C14), 129.7 (C15), 119.1 (C17), 84.8 (C6), 71.6 (C10), 46.3 (C3), 42.4 (C2), 38.4 (C11), 37.0 (C8), 33.3 (C9), 33.2 (C5), 26.2 (C7), 24.8 (C4), 21.4 (C18), 17.1 (C19).
LC-LRMS (ESI+) *m/z*: 300.06 found for $[M+H]^+$, 300.20 calcd. for $C_{19}H_{26}NO_2^+$, R_t = 1.26 min, purity (UV area): ≥95%.
HRMS (ESI+) *m/z*: 300.1957 found for $[M+H]^+$, 300.1958 calcd. for $C_{19}H_{26}NO_2^+$ (Δ = -0.25 ppm).

Note: The relative stereochemistry was determined by an observed NOE between the hydroxy (C6-O-H) proton and the methyl (C7-H_3) protons in the 2D ^{1}H-^{1}H NOESY spectrum (please see NMR spectra).

Synthesis of spirooxepinoindole analogues

General procedure H

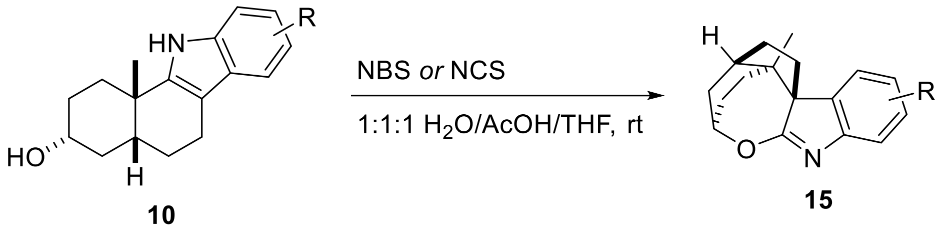

10 **15**

A solution of the appropriate indole analogue (**10**; 1.00 equiv.) and *N*-bromosuccinimide (1.05 equiv.) (with electron-poor indoles) *or* *N*-chlorosuccinimide (1.05 equiv.) (with electron-rich indoles) in 1:1:1 deionised water/glacial acetic acid/tetrahydrofuran (46-59 mM **10**) was stirred at room temperature for the indicated time. The reaction mixture was poured into saturated aqueous Na_2CO_3 and extracted with dichloromethane (3×). The organic phases were washed with saturated aqueous $NaHCO_3$, water, and brine, dried over anhydrous $MgSO_4$, and the solvent removed under reduced pressure to yield the crude products. The crude products were purified by flash column chromatography to afford the spirooxepinoindole analogues (**15**).

(3*R,3a*S**,6*R**,12b*R**)-3a-Methyl-2,3,3a,4,5,6-hexahydro-1*H*-3,6-methanocyclopenta[3,4]oxepino[2,3-*b*]indole (15a)**

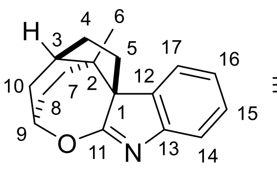

15a

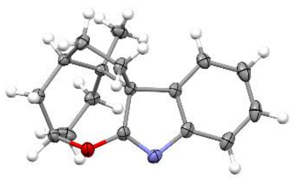

15a [X-ray]

Following general procedure H using compound **10a** (74.9 mg, 0.293 mmol) and *N*-bromosuccinimide (54.8 mg, 0.308 mmol), stirring for one hour, and purifying on silica gel eluting with 1:1 to 2:1 diethyl ether/*n*-pentane afforded compound **15a** (35.7 mg, 48% yield (59% brsm)) as a white solid.

Note: The unreacted starting material (**10a**; 14.0 mg) was isolated in separate fractions.

TLC: R_f = 0.25 (2:1 diethyl ether/*n*-pentane), UV.
mp = 112.0 – 115.0 °C.
IR (ATR) $\tilde{\upsilon}_{\max}$ cm^{-1}: 3048 (w), 2950 (m), 2920 (m), 2877 (m), 2856 (m), 1700 (w), 1611 (w), 1570 (s), 1454 (m), 1391 (m), 1351 (w), 1340 (w), 1312 (m), 1245 (m), 1219 (m), 1208 (m), 1121 (w), 1110 (w), 1092 (w), 1051 (w), 1039 (w), 997 (m), 966 (w), 946 (m), 770 (w), 744 (m).
^{1}H NMR (400 MHz, CD_3OD) δ ppm: 7.36 (d, *J* = 7.7 Hz, 1H, C17-H), 7.28 – 7.20 (m, 2H, C14-H + C15-H), 7.12 (td, *J* = 7.2, 2.0 Hz, 1H, C16-H), 4.67 (quint, *J* = 2.9 Hz, 1H, C9-H), 2.59 – 2.48 (m, 1H, C4-H), 2.34 – 2.19 (m, 3H, C3-H + C5-H + C10-H), 2.14 – 1.94 (m, 3H, C5-H + C8-H + C10-H), 1.82 – 1.72 (m, 1H, C8-H), 1.70 – 1.61 (m, 1H, C4-H), 1.58 – 1.47 (m, 1H, C7-H), 1.32 (s, 3H, C6-H_3), 1.29 – 1.20 (m, 1H, C7-H).
^{13}C NMR (101 MHz, CD_3OD) δ ppm: 187.8 (C11), 153.6 (C13), 140.2 (C12), 128.9 (C15), 125.4 (C17), 125.3 (C17), 119.2 (C14), 76.7 (C9), 66.9 (C1), 45.5 (C2), 43.8 (C3), 36.9 (C10), 36.7 (C5), 33.1 (C4), 28.1 (C6), 27.3 (C7), 25.9 (C8).
^{1}H NMR (800 MHz, DMSO-d_6) δ ppm: 7.34 (d, *J* = 7.4 Hz, 1H, C17-H), 7.23 – 7.19 (m, 2H, C14-H + C15-H), 7.07 (ddd, *J* = 7.3, 5.9, 2.8 Hz, 1H, , C16-H), 4.60 (quint, *J* = 2.9 Hz, 1H, C9-H), 2.45 – 2.39 (m, 1H, C4-H), 2.23 – 2.11 (m, 3H, C3-H + C5-H + C10-H), 1.99 – 1.92 (m, 1H, C5-H), 1.88 – 1.82 (m, 2H, C8-H + C10-H), 1.72 – 1.65 (m, 1H, C8-H), 1.55 (ddd, *J* = 13.2, 9.8, 3.9 Hz, 1H, C4-H), 1.42 (ddd, *J* = 14.9, 12.0, 5.1 Hz, 1H, C7-H), 1.23 (s, 3H, C6-H_3), 1.07 (ddd, *J* = 14.9, 10.5, 4.1 Hz, 1H, C7-H).
^{13}C NMR (101 MHz, DMSO-d_6) δ ppm: 184.7 (C11), 153.6 (C13), 139.3 (C12), 127.4 (C15), 124.1 (C17), 123.6 (C16), 118.3 (C14), 73.8 (C9), 64.9 (C1), 43.8 (C2), 41.8 (C3), 35.5 (C10), 34.9 (C5), 31.8 (C4), 27.2 (C6), 26.0 (C7), 24.4 (C8).
^{15}N NMR (81 MHz, DMSO-d_6) δ ppm: 264.6.
LC-LRMS (ESI+) *m/z*: 254.28 found for $[M+H]^+$, 254.15 calcd. For $C_{17}H_{20}NO^+$, R_t = 2.74 min, purity (UV area): ≥95%.

HRMS (ESI+) *m/z*: 254.1550 found for $[M+H]^+$, 254.1539 calcd. For $C_{17}H_{20}NO^+$ (Δ = 4.18 ppm).
X-ray (single-crystal): Crystal was grown by vapour diffusion (solvent = dichloromethane; antisolvent = *n*-pentane) until equilibrium then followed by mixed solvent slow evaporation.

(3*R,3a*S**,6*R**,12b*R**)-11-Bromo-3a-methyl-2,3,3a,4,5,6-hexahydro-1*H*-3,6-methanocyclopenta[3,4]oxepino[2,3-*b*]indole (15b)**

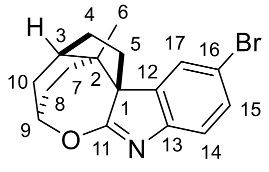

15b

Following general procedure H using compound **10b** (2.34 g, 6.99 mmol) and *N*-bromosuccinimide (1.31 g, 7.38 mmol), stirring for one hour, and purifying on silica gel eluting with 20:1 to 5:1 toluene/ethyl acetate afforded compound **15b** (0.695 g, 30% yield) as a white solid.

TLC: R_f = 0.23 (4:1 toluene/ethyl acetate), UV.
mp = 156.0 – 159.0 °C.
IR (ATR) $\tilde{\upsilon}_{\max}$ cm^{-1}: 2961 (m), 2915 (m), 2859 (m), 1715 (w), 1608 (w), 1568 (s), 1459 (m), 1445 (m), 1415 (w), 1386 (m), 1354 (w), 1336 (w), 1319 (w), 1306 (w), 1277 (w), 1244 (s), 1212 (m), 1120 (w), 1093 (w), 1053 (w) 1001 (m), 967 (w), 952 (m), 888 (w), 877 (w), 794 (m), 735 (w), 686 (w), 637 (w), 528 (w).
^{1}H NMR (400 MHz, CD_3OD) δ ppm: 7.52 (d, *J* = 2.0 Hz, 1H, C17-H), 7.41 (dd, *J* = 8.2, 2.0 Hz, 1H, C15-H), 7.14 (d, *J* = 8.3 Hz, 1H, C14-H), 4.69 (quint, *J* = 2.8 Hz, 1H, C9-H), 2.59 – 2.47 (m, 1H, C4-H), 2.33 – 2.20 (m, 3H, C3-H + C5-H + C10-H), 2.16 – 2.07 (m, 1H, C5-H), 2.06 – 1.93 (m, 2H, C8-H + C10-H), 1.82 – 1.73 (m, 1H, C8-H), 1.69 – 1.61 (m, 1H, C4-H), 1.59 – 1.51 (m, 1H, C7-H), 1.32 – 1.21 (m, 4H, C6-H_3 + C7-H).
^{13}C NMR (101 MHz, CD_3OD) δ ppm: 188.2 (C11), 152.8 (C13), 142.6 (C12), 131.9 (C15), 128.6 (C17), 120.7 (C14), 118.4 (C16), 77.0 (C9), 67.3 (C1), 45.8 (C2), 43.7 (C3), 36.72 (C10), 36.71 (C5), 33.0 (C4), 27.9 (C6), 27.3 (C7), 25.9 (C8).
^{1}H NMR (400 MHz, $CDCl_3$) δ ppm: 7.40 – 7.35 (m, 2H, C15-H + C17-H), 7.23 (d, *J* = 8.7 Hz, 1H, C14-H), 4.71 – 4.65 (m, 1H, C9-H), 2.51 – 2.40 (m, 1H, C4-H), 2.28 – 2.07 (m, 5H, C3-H + C5-H_2 + C8-H + C10-H), 1.99 (dq, *J* = 15.1, 3.0 Hz, 1H, C10-H), 1.71 – 1.61 (m, 2H, C4-H + C8-H), 1.50 – 1.41 (m, 1H, C7-H), 1.38 – 1.32 (m, 1H, C7-H), 1.30 (s, 3H, C6-H_3).
^{13}C NMR (101 MHz, $CDCl_3$) δ ppm: 185.6 (C11), 152.7 (C13), 141.3 (C12), 130.9 (C15), 127.1 (C17), 120.6 (C14), 117.2 (C16), 74.9 (C9), 66.0 (C1), 44.7 (C2), 42.6 (C3), 36.2 (C10), 35.6 (C5), 32.3 (C4), 27.7 (C6), 26.4 (C7), 25.0 (C8).
^{1}H NMR (800 MHz, DMSO-d_6) δ ppm: 7.52 (d, *J* = 2.0 Hz, 1H, C17-H), 7.40 (dd, *J* = 8.1, 2.0 Hz, 1H, C15-H), 7.16 (d, *J* = 8.1 Hz, 1H, C14-H), 4.62 (quint, *J* = 3.0 Hz, 1H, C9-H), 2.45 – 2.39 (m, 1H, C4-H), 2.23 – 2.18 (m, 2H, C3-H + C5-H), 2.15 (ddd, *J* = 14.1, 10.9, 2.8 Hz, 1H, C10-H), 1.97 (ddd, *J* = 14.1, 9.3, 7.4 Hz, 1H, C5-H), 1.88 – 1.80 (m, 2H, C8-H + C10-H), 1.71 – 1.66 (m, 1H, C8-H), 1.54 (ddd, *J* = 13.1, 9.7, 3.7 Hz, 1H, C4-H), 1.44 (ddd, *J* = 14.9, 12.0, 4.9 Hz, 1H, C7-H), 1.23 (s, 3H, C6-H_3), 1.08 (ddd, *J* = 15.0, 10.6, 4.3 Hz, 1H, C7-H).
^{13}C NMR (201 MHz, DMSO-d_6) δ ppm: 185.2 (C11), 152.8 (C13), 141.8 (C12), 130.3 (C15), 127.0 (C17), 120.1 (C14), 116.0 (C16), 74.2 (C9), 65.5 (C1), 44.1 (C2), 41.7 (C3), 35.3 (C10), 34.7 (C5), 31.7 (C4), 27.1 (C6), 26.0 (C7), 24.4 (C8).
^{15}N NMR (81 MHz, DMSO-d_6) δ ppm: 262.2.
LC-LRMS (ESI+) *m/z*: 331.93 found for $[M+H]^+$, 332.06 calcd. For $C_{17}H_{19}BrNO^+$, R_t = 2.84 min, purity (UV area): 100%.
HRMS (ESI+) *m/z*: 332.0643 found for $[M+H]^+$, 332.0645 calcd. For $C_{17}H_{19}BrNO^+$ (Δ = -0.53 ppm).

(3*R,3a*S**,6*R**,12b*R**)-11-Chloro-3a-methyl-2,3,3a,4,5,6-hexahydro-1*H*-3,6-methanocyclopenta[3,4]oxepino[2,3-*b*]indole (15c)**

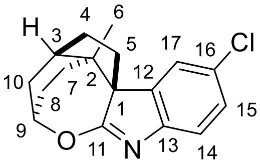

15c

Following general procedure H using compound **10c** (100 mg, 0.345 mmol) and *N*-bromosuccinimide (64.7 mg, 0.364 mmol), stirring for one hour, and purifying on BÜCHI® system using FlashPure Select Silica (12 g) eluting with 1:0 to 0:1 dichloromethane/ethyl acetate (flow rate = 24 mL/min) afforded compound **15c** (41.0 mg, 41% yield) as an eggshell-white solid.

TLC: R_f = 0.24 (19:1 dichloromethane/ethyl acetate), UV.
mp = 156.0 – 160.0 °C.
IR (ATR) $\tilde{\upsilon}_{\max}$ cm^{-1}: 2960 (m), 2942 (m), 2926 (m), 2875 (m), 2861 (m), 1739 (w), 1610 (w), 1568 (s), 1448 (m), 1385 (m), 1357 (w), 1338 (w), 1319 (w), 1308 (w), 1279 (w), 1244 (m), 1202 (m), 1118 (w), 1093 (w), 1073 (w), 1048 (w), 999 (m), 954 (m), 887 (w), 819 (m), 795 (w), 711 (w), 670 (w), 539 (w).
^{1}H NMR (400 MHz, $CDCl_3$) δ ppm: 7.28 – 7.20 (m, 3H, C14-H + C15-H + C17-H), 4.69 – 4.64 (m, 1H, C9-H), 2.51 – 2.41 (m, 1H, C4-H), 2.27 – 2.07 (m, 5H, C3-H + C5-H_2 + C8-H + C10-H), 1.99 (dq, *J* = 15.1, 3.0 Hz, 1H, C10-H), 1.71 – 1.61 (m, 2H, C4-H + C8-H), 1.45 (ddd, *J* = 14.9, 11.8, 5.4 Hz, 1H, C7-H), 1.38 – 1.28 (m, 4H, C6-H_3 + C7-H).
^{13}C NMR (101 MHz, $CDCl_3$) δ ppm: 185.6 (C11), 152.5 (C13), 141.0 (C12), 129.3 (C16), 127.9 (C15), 124.2 (C17), 120.1 (C14), 74.7 (C9), 66.0 (C1), 44.7 (C2), 42.7 (C3), 36.2 (C10), 35.6 (C5), 32.3 (C4), 27.7 (C6), 26.5 (C7), 25.0 (C8).
LC-LRMS (ESI+) *m/z*: 287.97 found for $[M+H]^+$, 288.12 calcd. for $C_{17}H_{19}ClNO^+$, R_t = 1.82 min, purity (UV area): >99%.
HRMS (ESI+) *m/z*: 288.1149 found for $[M+H]^+$, 288.1150 calcd. for $C_{17}H_{19}ClNO^+$ (Δ = -0.27 ppm).

Note: The peak for the C14-H, C15-H, and C17-H protons at 7.28 – 7.20 ppm in the 1D ^{1}H NMR spectrum overlaps with the chloroform-*d* solvent peak, thus increasing the integral, but the chemical shift and integral were confirmed by correlations in the 2D ^{1}H-^{1}H COESY and ^{1}H-^{13}C HSQC and HMBC spectra.

(3*R,3a*S**,6*R**,12b*R**)-11-Fluoro-3a-methyl-2,3,3a,4,5,6-hexahydro-1*H*-3,6-methanocyclopenta[3,4]oxepino[2,3-*b*]indole (15d)**

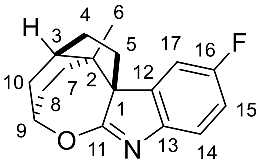

15d

Following general procedure H using compound **10d** (100 mg, 0.366 mmol) and *N*-bromosuccinimide (64.7 mg, 0.387 mmol), stirring for one hour, and purifying on BÜCHI® system using FlashPure Select Silica (12 g) eluting with 1:0 to 0:1 *n*-heptane/ethyl acetate (flow rate = 24 mL/min) afforded compound **15d** (45.5 mg, 46% yield) as an eggshell-white solid.

TLC: R_f = 0.29 (1:1 *n*-heptane/ethyl acetate), UV.
mp = 167.5 – 169.5 °C.
IR (ATR) $\tilde{\upsilon}_{\max}$ cm^{-1}: 3024 (w), 2952 (m), 2923 (m), 2889 (w), 2860 (w), 1617 (w), 1572 (s), 1459 (s), 1394 (m), 1360 (w), 1342 (w), 1328 (w), 1311 (w), 1283 (w), 1254 (m), 1205 (m), 1163 (w), 1153 (w), 1119 (w). 1103 (m), 1050 (w), 1040 (w), 998 (m), 961 (m), 896 (w), 873 (w), 833 (w), 818 (m), 781 (m), 645 (w), 567 (w).
^{1}H NMR (400 MHz, $CDCl_3$) δ ppm: 7.27 (dd, *J* = 8.4, 4.9 Hz, 1H, C14-H), 7.00 – 6.91 (m, 2H, C15-H + C17-H), 4.68 – 4.63 (m, 1H, C9-H), 2.51 – 2.41 (m, 1H, C4-H), 2.27 – 2.07 (m, 5H, C3-H + C5-H_2 + C8-H + C10-H), 2.00 (dq, *J* = 14.9, 2.9 Hz, 1H, C10-H), 1.73 – 1.58 (m, 2H, C4-H + C8-H), 1.45 (ddd, *J* = 14.8, 11.7, 5.5 Hz, 1H, C7-H), 1.38 – 1.27 (m, 4H, C6-H_3 + C7-H).

^{13}C NMR (101 MHz, $CDCl_3$) δ ppm: 185.0 (d, *J* = 2.8 Hz, C11), 160.1 (d, *J* = 240.7 Hz, C16), 149.7 (d, *J* = 2.2 Hz, C13), 140.9 (d, *J* = 8.3 Hz, C12), 119.7 (d, *J* = 8.6 Hz, C14), 114.2 (d, *J* = 23.1 Hz, C15), 111.7 (d, *J* = 24.9 Hz, C17), 74.5 (C9), 66.1 (d, *J* = 2.5 Hz, C1), 44.7 (C2), 42.7 (C3), 36.3 (C10), 35.6 (C5), 32.3 (C4), 27.5 (C6), 26.5 (C7), 25.0 (C8).
^{19}F NMR (377 MHz, $CDCl_3$) δ ppm: -118.8.
LC-LRMS (ESI+) *m/z*: 272.26 found for $[M+H]^+$, 272.14 calcd. for $C_{17}H_{19}FNO^+$, R_t = 1.62 min, purity (UV area): 98%.
HRMS (ESI+) *m/z*: 272.1444 found for $[M+H]^+$, 272.1445 calcd. for $C_{17}H_{19}FNO^+$ (Δ = -0.28 ppm).

Note: The peak for the C14-H proton at 7.27 ppm in the 1D ^{1}H NMR spectrum overlaps with the chloroform-*d* solvent peak, thus increasing the integral, but the chemical shift and integral were confirmed by correlations in the 2D ^{1}H-^{1}H COESY and ^{1}H-^{13}C HSQC and HMBC spectra.

(3*R,3a*S**,6*R**,12b*R**)-3a,11-Dimethyl-2,3,3a,4,5,6-hexahydro-1*H*-3,6-methanocyclopenta[3,4]oxepino[2,3-*b*]indole (15e)**

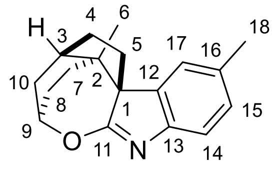

15e

Following general procedure H using compound **10e** (47.5 mg, 176 μmol) and *N*-chlorosuccinimide (25.0 mg, 187 μmol), stirring for two hours, and purifying on BÜCHI® system using FlashPure Select Silica (4 g) eluting with 1:0 to 0:1 dichloromethane/ethyl acetate (flow rate = 12 mL/min) afforded compound **15e** (27.1 mg, 57% yield) as a cream-white solid.

TLC: R_f = 0.26 (10:1 dichloromethane/ethyl acetate), UV.
mp = 144.0 – 146.6 °C.
IR (ATR) $\tilde{\upsilon}_{max}$ cm^{-1}: 2964 (m), 2914 (m), 2894 (m), 2860 (m), 1615 (w), 1573 (s), 1461 (m), 1388 (m), 1354 (w), 1336 (w), 1309 (m), 1246 (m), 1200 (m), 1154 (w), 1118 (w), 1093 (m), 1054 (w), 1028 (w), 999 (m), 955 (m), 892 (w), 879 (w), 818 (m), 802 (w), 777 (w), 642 (w), 585 (w).
^{1}H NMR (400 MHz, CD_3OD) δ ppm: 7.20 – 7.16 (m, 1H, C17-H), 7.12 – 7.03 (m, 2H, C14-H + C15-H), 4.66 – 4.62 (m, 1H, C9-H), 2.58 – 2.46 (m, 1H, C4-H), 2.35 (s, 3H, C18-H_3), 2.31 – 2.17 (m, 3H, C3-H + C5-H + C10-H), 2.11 – 1.93 (m, 3H, C5-H + C8-H + C10-H), 1.81 – 1.70 (m, 1H, C8-H), 1.64 (dddd, *J* = 13.6, 9.5, 4.3, 1.4 Hz, 1H, C4-H), 1.51 (ddd, *J* = 14.9, 12.0, 5.3 Hz, 1H, C7-H), 1.31 (s, 3H, C6-H_3), 1.24 (ddd, *J* = 14.9, 10.9, 4.3 Hz, 1H, C7-H).
^{13}C NMR (101 MHz, CD_3OD) δ ppm: 187.1 (C11), 151.1 (C13), 140.2 (C12), 135.1 (C16), 129.3 (C15), 126.2 (C17), 118.8 (C14), 76.5 (C9), 66.8 (C1), 45.5 (C2), 43.8 (C3), 37.0 (C10), 36.8 (C5), 33.1 (C4), 28.1 (C6), 27.4 (C7), 25.9 (C8), 21.6 (C18).
LC-LRMS (ESI+) *m/z*: 268.34 found for $[M+H]^+$, 268.17 calcd. for $C_{18}H_{22}NO^+$, R_t = 1.55 min, purity (UV area): 97%.
HRMS (ESI+) *m/z*: 268.1696 found for $[M+H]^+$, 268.1696 calcd. for $C_{18}H_{22}NO^+$ (Δ = 0.00 ppm).

(3*R,3a*S**,6*R**,12b*R**)-11-Methoxy-3a-methyl-2,3,3a,4,5,6-hexahydro-1*H*-3,6-methanocyclopenta[3,4]oxepino[2,3-*b*]indole (15f)**

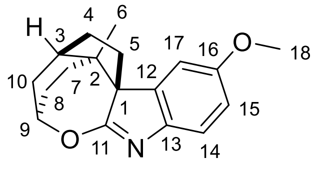

15f

Following general procedure H using compound **10f** (67.4 mg, 0.236 mmol) and *N*-chlorosuccinimide (33.8 mg, 0.253 mmol), stirring for two hours, and purifying on BÜCHI® system using FlashPure Select Silica (12 g) eluting with 1:0 to 0:1 *n*-heptane/ethyl acetate (flow rate = 24 mL/min) afforded compound **15f** (39.1 mg, 58% yield) as a brown solid.

TLC: R_f = 0.24 (1:1 *n*-heptane/ethyl acetate), UV.

mp = 114.5 – 117.0 °C (dec).
IR (ATR) $\tilde{\upsilon}_{\max}$ cm^{-1}: 2941 (m), 2834 (w), 1734 (w), 1707 (w), 1620 (w), 1576 (s), 1461 (s), 1387 (m), 1341 (m), 1311 (w), 1245 (m), 1210 (m), 1158 (m), 1115 (m), 1094 (w), 1040 (m), 998 (m), 954 (m), 872 (w), 814 (m), 767 (w), 649 (w), 598 (w), 584 (w).
^{1}H NMR (400 MHz, $CDCl_3$) δ ppm: 7.26 (d, *J* = 8.4 Hz, 1H, C14-H), 6.85 (d, *J* = 2.5 Hz, 1H, C17-H), 6.78 (dd, *J* = 8.4, 2.6 Hz, 1H, C15-H), 4.65 – 4.61 (m, 1H, C9-H), 3.80 (s, 3H, C18-H_3), 2.50 – 2.40 (m, 1H, C4-H), 2.26 – 2.05 (m, 5H, C3-H + C5-H_2 + C8-H + C10-H), 1.99 (dq, *J* = 14.9, 2.8 Hz, 1H, C10-H), 1.69 – 1.60 (m, 2H, C4-H + C8-H), 1.47 – 1.31 (m, 2H, C7-H_2), 1.29 (s, 3H, C6-H_3).
^{13}C NMR (101 MHz, $CDCl_3$) δ ppm: 183.8 (C11), 156.8 (C16), 147.3 (C13), 140.7 (C12), 119.3 (C14), 111.7 (C17), 111.6 (C15), 74.3 (C9), 65.8 (C1), 55.9 (C18), 44.6 (C2), 42.7 (C3), 36.4 (C10), 35.8 (C5), 32.4 (C4), 27.5 (C6), 26.5 (C7), 25.0 (C8).
LC-LRMS (ESI+) *m/z*: 284.31 found for $[M+H]^+$, 284.16 calcd. for $C_{18}H_{22}NO_2^+$, R_t = 1.42 min, purity (UV area): ≥95%.
HRMS (ESI+) *m/z*: 284.1645 found for $[M+H]^+$, 284.1645 calcd. for $C_{18}H_{22}NO_2^+$ (Δ = 0.00 ppm).

Note: The peak for the C14-H proton at 7.26 ppm in the 1D ^{1}H NMR spectrum overlaps with the chloroform-*d* solvent peak, thus increasing the integral, but the chemical shift and integral were confirmed by correlations in the 2D ^{1}H-^{1}H COESY and ^{1}H-^{13}C HSQC and HMBC spectra.

(3*R,3a*S**,6*R**,12b*R**)-3a-Methyl-11-(trifluoromethyl)-2,3,3a,4,5,6-hexahydro-1*H*-3,6-methanocyclopenta[3,4]oxepino[2,3-*b*]indole (15g)**

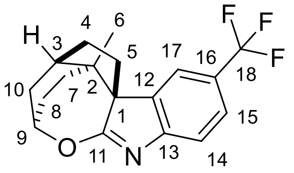

15g

(3*R,3a*S**,6*R**,12b*R**)-9-Bromo-3a-methyl-11-(trifluoromethyl)-2,3,3a,4,5,6-hexahydro-1*H*-3,6-methanocyclopenta[3,4]oxepino[2,3-*b*]indole (15h)**

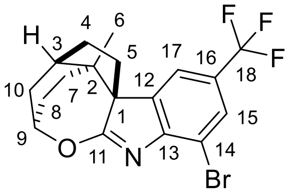

15h

Following general procedure H using compound **10g** (75.0 mg, 0.232 mmol) and *N*-bromosuccinimide (43.6 mg, 0.245 mmol), stirring for one hour, and purifying twice on silica gel eluting with 1. 9:2 to 2:3 *n*-heptane/ethyl acetate and 2. 2:1 *n*-heptane/ethyl acetate afforded compound **15g** (14.9 mg, 20% yield) as a white solid and compound **15h** (7.3 mg, 8% yield) as a pale-yellow solid in separate fractions.

Note: The unreacted starting material (**10g**; 7.1 mg) was isolated in separate fractions.

Characterisation data for **15g**:
TLC: R_f = 0.20 (2:1 *n*-heptane/ethyl acetate), UV.
mp = 114.0 – 117.0 °C.
IR (ATR) $\tilde{\upsilon}_{\max}$ cm^{-1}: 2951 (m), 2923 (m), 2884 (w), 1626 (w), 1565 (s), 1462 (w), 1434 (m), 1401 (w), 1381 (w), 1346 (m), 1316 (s), 1243 (s), 1201 (m), 1158 (m), 1146 (m), 1115 (s), 1104 (s), 1058 (m), 995 (m), 953 (m), 896 (w), 840 (m), 799 (w), 766 (w), 680 (w), 633 (w).
^{1}H NMR (400 MHz, CD_3OD) δ ppm: 7.64 (d, *J* = 1.8 Hz, 1H, C17-H), 7.61 – 7.57 (m, 1H, C15-H), 7.38 (d, *J* = 8.1 Hz, 1H, C14-H), 4.74 (quint, *J* = 2.9 Hz, 1H, C9-H), 2.63 – 2.51 (m, 1H, C4-H), 2.37 – 2.24 (m, 3H, C3-H + C5-H + C10-H), 2.20 – 2.11 (m, 1H, C5-H), 2.09 – 1.97 (m, 2H, C8-H + C10-H), 1.84 – 1.73 (m, 1H, C8-H), 1.70 – 1.63 (m, 1H, C4-H), 1.57 (ddd, *J* = 15.0, 12.0, 4.8 Hz, 1H, C7-H), 1.35 (s, 3H, C6-H_3), 1.25 (ddd, *J* = 15.1, 11.0, 4.8 Hz, 1H, C7-H).
^{13}C NMR (101 MHz, CD_3OD) δ ppm: 190.2 (C11), 157.3 (C13), 141.2 (C12), 127.2 (q, *J* = 32.1 Hz, C16), 126.6 (q, *J* = 4.0 Hz, C15), 126.1 (q, *J* = 270.9 Hz, C18), 122.2 (q, *J* = 4.0 Hz, C17), 119.4 (C14),

77.4 (C9), 67.3 (C1), 45.8 (C2), 43.7 (C3), 36.7 (C10), 36.6 (C5), 33.0 (C4), 28.0 (C6), 27.3 (C7), 26.0 (C8).
^{19}F NMR (377 MHz, CD_3OD) δ ppm: -62.9.
LC-LRMS (ESI+) *m/z*: 322.35 found for $[M+H]^+$, 322.14 calcd. for $C_{18}H_{19}F_3NO^+$, R_t = 1.93 min, purity (UV area): 99%.
HRMS (ESI+) *m/z*: 322.1413 found for $[M+H]^+$, 322.1413 calcd. for $C_{18}H_{19}F_3NO^+$ (Δ = 0.00 ppm).

Note: LC-LRMS indicates a purity of 99% (please see LC-LRMS trace) but unknown impurities are observed in the 1D ^{13}C NMR spectrum.

Characterisation data for **15h**:
TLC: R_f = 0.26 (2:1 *n*-heptane/ethyl acetate), UV.
mp = 157.0 – 160.0 °C.
IR (ATR) $\tilde{\upsilon}_{max}$ cm^{-1}: 2958 (m), 2933 (m), 2864 (w), 1726 (w), 1621 (w), 1552 (s), 1475 (w), 1457 (w), 1410 (m), 1350 (w), 1333 (m), 1303 (s), 1288 (m), 1253 (m), 1230 (m), 1195 (w), 1145 m), 1118 (s), 1098 (m), 1080 (m), 1049 (m), 1030 (m), 997 (m), 960 (m), 887 (m), 860 (w), 830 (w), 812 (m), 766 (w), 675 (w), 661 (w).
^{1}H NMR (400 MHz, CD_3OD) δ ppm: 7.77 – 7.74 (m, 1H, C15-H), 7.64 – 7.61 (m, 1H, C17-H), 4.79 (quint, *J* = 3.0 Hz, 1H, C9-H), 2.62 – 2.52 (m, 1H, C4-H), 2.38 – 2.27 (m, 3H, C3-H + C5-H + C10-H), 2.24 – 2.14 (m, 1H, C5-H), 2.12 – 1.96 (m, 2H, C8-H + C10-H), 1.86 – 1.76 (m, 1H, C8-H), 1.72 – 1.55 (m, 2H, C4-H + C7-H), 1.35 (s, 3H, C6-H_3), 1.28 (ddd, *J* = 15.2, 10.6, 4.8 Hz, 1H, C7-H).
^{13}C NMR (101 MHz, CD_3OD) δ ppm: 191.1 (C11), 156.0 (C13), 142.8 (C12), 129.7 (q, *J* = 4.1 Hz, C15), 128.4 (q, *J* = 32.7 Hz, C16), 125.1 (q, *J* = 271.8 Hz, C18), 121.3 (q, J = 3.6 Hz, C17), 112.7 (C14), 77.9 (C9), 68.8 (C1), 46.1 (C2), 43.7 (C3), 36.8 (C5), 36.5 (C10), 33.0 (C4), 28.0 (C6), 27.3 (C7), 26.0 (C8).
^{19}F NMR (377 MHz, CD_3OD) δ ppm: -63.1.
LC-LRMS (ESI+) *m/z*: 400.04 found for $[M+H]^+$, 400.05 calcd. for $C_{18}H_{18}BrF_3NO^+$, R_t = 2.13 min, purity (UV area): 97%.
HRMS (ESI+) *m/z*: 400.0529 found for $[M+H]^+$, 400.0518 calcd. for $C_{18}H_{18}BrF_3NO^+$ (Δ = 2.66 ppm).

Note: LC-LRMS indicates a purity of 97% (please see LC-LRMS trace) but unknown impurities are observed in the 1D ^{1}H and ^{13}C NMR spectra. The regiochemistry (bromination site) was determined by the characteristic $^3J_{C\text{-}F}$ coupling for the C15 and C17 carbons and the observed correlations between the C15-H proton and C15 carbon and the C17-H proton and C17 carbon in the 2D ^{1}H-^{13}C HSQC spectrum (please see NMR spectra).

(3*R,3a*S**,6*R**,12b*R**)-11-Chloro-9-fluoro-3a-methyl-2,3,3a,4,5,6-hexahydro-1*H*-3,6-methanocyclopenta[3,4]oxepino[2,3-*b*]indole (15i)**

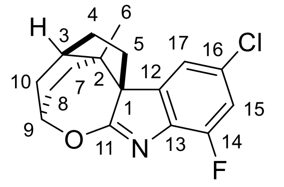

15i

Following general procedure H using compound **10i** (75.0 mg, 0.244 mmol) and *N*-bromosuccinimide (45.6 mg, 0.256 mmol), stirring for one hour, and purifying on silica gel eluting with 5:1 to 1:1 *n*-heptane/ethyl acetate afforded compound **15i** (30.5 mg, 41% yield) as a white solid.

TLC: R_f = 0.18 (3:1 *n*-heptane/ethyl acetate), UV.
mp = 140.0 – 143.0 °C.
IR (ATR) $\tilde{\upsilon}_{max}$ cm^{-1}: 2957 (m), 2925 (m), 2912 (m), 2847 (w), 1729 (w), 1625 (m), 1568 (s), 1446 (m), 1426 (m), 1385 (m), 1355 (w), 1313 (w), 1282 (m), 1256 (m), 1219 (m), 1203 (m), 1130 (w), 1097 (m), 1078 (w), 1052 (w), 1036 (w), 1001 (m), 960 (m), 919 (m), 906 (w), 877 (w), 846 (w), 817 (m), 793 (w), 759 (w), 718 (w), 663 (w), 584 (w).
^{1}H NMR (400 MHz, CD_3OD) δ ppm: 7.25 (d, *J* = 1.8 Hz, 1H, C17-H), 7.15 (dd, *J* = 9.7, 1.8 Hz, 1H, C15-H), 4.72 (quint, *J* = 3.0 Hz, 1H, C9-H), 2.60 – 2.48 (m, 1H, C4-H), 2.35 – 2.22 (m, 3H, C3-H + C5-H + C10-H), 2.20 – 2.09 (m, 1H, C5-H), 2.08 – 1.96 (m, 2H, C8-H + C10-H), 1.84 – 1.74 (m, 1H, C8-H), 1.66 (ddd, *J* = 13.3, 9.3, 4.0 Hz, 1H, C4-H), 1.57 (ddd, *J* = 15.0, 12.0, 5.0 Hz, 1H, C7-H), 1.33 – 1.23 (m, 4H, C6-H_6 + C7-H).

^{13}C NMR (101 MHz, CD_3OD) δ ppm: 188.4 (C11), 153.3 (d, *J* = 253.5 Hz, C14), 144.7 (d, *J* = 4.4 Hz, C12), 139.2 (d, *J* = 11.3 Hz, C13), 131.0 (d, *J* = 8.4 Hz, C16), 121.9 (d, *J* = 3.6 Hz, C17), 116.7 (d, *J* = 22.1 Hz, C15), 77.3 (C9), 68.0 (d, *J* = 1.6 Hz, C1), 46.0 (C2), 43.7 (C3), 36.9 (C5), 36.6 (C10), 33.0 (C4), 27.9 (C6), 27.3 (C7), 25.9 (C8).
^{19}F NMR (377 MHz, CD_3OD) δ ppm: -129.0.
^{1}H NMR (400 MHz, DMSO-d_6) δ ppm: 7.35 – 7.28 (m, 2H, C15-H + C17-H), 4.66 (quint, *J* = 3.0 Hz, 1H, C9-H), 2.47 – 2.37 (m, 1H, C4-H), 2.27 – 2.12 (m, 3H, C3-H + C5-H + C10-H), 2.06 – 1.95 (m, 1H, C5-H), 1.92 – 1.81 (m, 2H, C8-H + C10-H), 1.75 – 1.65 (m, 1H, C8-H), 1.55 (ddd, *J* = 12.8, 9.3, 3.8 Hz, 1H, C4-H), 1.45 (ddd, *J* = 14.9, 11.9, 4.9 Hz, 1H, C7-H), 1.23 (s, 3H, C6-H_3), 1.11 (ddd, *J* = 15.0, 10.3, 4.3 Hz, 1H, C7-H).
^{13}C NMR (101 MHz, DMSO-d_6) δ ppm: 185.5 (C11), 151.4 (d, *J* = 252.4 Hz, C14), 143.9 (d, *J* = 4.8 Hz, C12), 139.0 (d, *J* = 10.7 Hz, C13), 128.1 (d, *J* = 8.7 Hz, C16), 120.7 (d, *J* = 3.5 Hz, C17), 115.4 (d, *J* = 22.3 Hz, C15), 74.6 (C9), 66.2 (d, *J* = 1.5 Hz, C1), 44.3 (C2), 41.7 (C3), 35.2 (C10), 35.0 (C5), 31.7 (C4), 27.0 (C6), 25.9 (C7), 24.4 (C8).
^{19}F NMR (377 MHz, DMSO-d_6) δ ppm: -126.8.
LC-LRMS (ESI+) *m/z*: 306.02 found for $[M+H]^+$, 306.11 calcd. for $C_{17}H_{18}ClFNO^+$, R_t = 1.95 min, purity (UV area): 95%.
HRMS (ESI+) *m/z*: 306.1065 found for $[M+H]^+$, 306.1056 calcd. for $C_{17}H_{18}ClFNO^+$ (Δ = 3.02 ppm).

Unsuccessful analogues

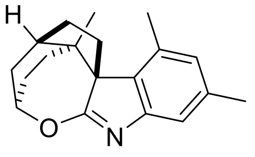

15j

Note: Primarily mono-chlorinated product (aromatic chlorination of **15j**) under the *N*-chlorosuccinimide conditions. No purification attempted at this point.

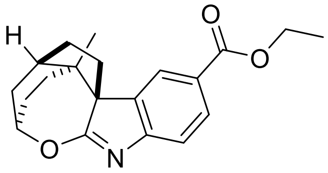

15k

Note: Slow and poor conversion into **15k** under the *N*-bromosuccinimide conditions, and primarily mono- and di-brominated products (aromatic bromination of **15k**) upon addition of *N*-chlorosuccinimide. No purification attempted at this point.

Synthesis of SAR spirooxepinoindole analogues

General procedure for palladium-scavenging

A mixture of the initial crude product and Biotage® MP-TMT (capacity = 0.65 mmol/g) (4.0 equiv. relative to starting Pd) in dichloromethane (40 mL/g Biotage® MP-TMT) was stirred at room temperature between 16-24 hours. The mixture was filtered washing with dichloromethane and 1:1 dichloromethane/methanol. The filtrate was concentrated under reduced pressure to yield the final crude products.

General procedure I

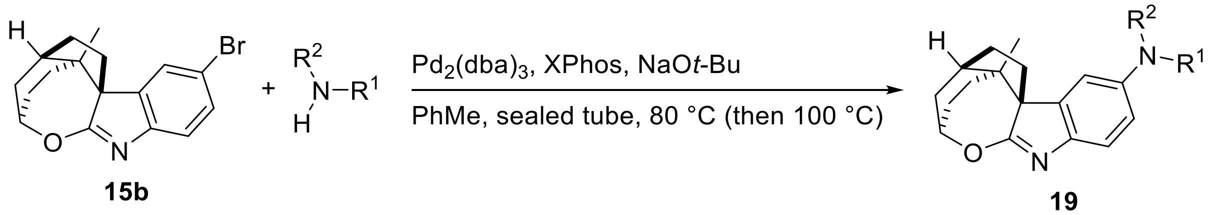

15b **19**

A screw-capped tube containing compound **15b** (1.00 equiv.), $Pd_2(dba)_3$ (0.02 equiv. = 2 mol%), XPhos (0.08 equiv. = 8 mol%), sodium *tert*-butoxide (1.40 equiv.), and the appropriate amine (if a solid at room temperature; 1.20 equiv.) was evacuated and backfilled with argon three times. The tube was added the appropriate amine (if a liquid at room temperature; 1.20 equiv.) and dry toluene (0.13 M **15b**), flushed with argon for 15 minutes at room temperature, and sealed. The reaction mixture was stirred at 80 °C in a sealed tube for the indicated time. If necessary, additional amine was added during the reaction. If necessary, the reaction was stirred at 100 °C. The reaction mixture was cooled to room temperature, diluted with ethyl acetate, and filtered through a pad of celite® washing with ethyl acetate. The filtrate was concentrated under reduced pressure to yield the initial crude products. The initial crude products were scavenged for palladium following the general procedure for palladium-scavenging to yield the final crude products. The final crude products were purified by flash column chromatography on BÜCHI® system to afford the spirooxepinoindole analogues (**19**).

(3*R,3a*S**,6*R**,12b*R**)-3a-Methyl-11-morpholino-2,3,3a,4,5,6-hexahydro-1*H*-3,6-methanocyclopenta[3,4]oxepino[2,3-*b*]indole, (±)-asteroxin-1 (19a)**

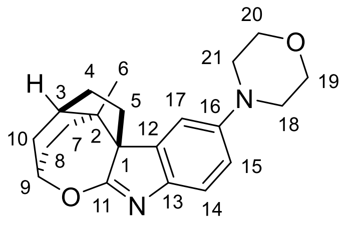

19a

Following general procedure I using compound **15b** (50.0 mg, 150 µmol) and morpholine (16 µL, 183 µmol), stirring at 80 °C for 14 hours, scavenging for 18 hours, and purifying using FlashPure EcoFlex Silica (4 g) eluting with 1:0 to 0:1 *n*-heptane/ethyl acetate (flow rate = 15 mL/min) afforded (±)-asteroxin-1 (**19a**; 41.5 mg, 82% yield) as a pale-yellow solid.

TLC: R_f = 0.21 (3:1 ethyl acetate/*n*-heptane), UV.
mp = 137.5 – 140.0 °C.
IR (ATR) $\tilde{\upsilon}_{\max}$ cm^{-1}: 2955 (m), 1926 (m), 2855 (m), 2806 (m), 1619 (w), 1571 (s), 1470 (s), 1454 (m), 1393 (m), 1377 (m), 1336 (w), 1303 (w), 1289 (w), 1239 (m), 1223 (m), 1194 (m), 1159 (w), 1110 (s), 1094 (m), 1066 (w), 1036 (w), 997 (m), 970 (w), 927 (m), 886 (m), 854 (m), 807 (m), 743 (w), 657 (w).
^{1}H NMR (400 MHz, $CDCl_3$) δ ppm: 7.26 (d, *J* = 8.4 Hz, 1H, C14-H), 6.89 (d, *J* = 2.4 Hz, 1H, C17-H), 6.81 (dd, *J* = 8.4, 2.5 Hz, 1H, C15-H), 4.65 – 4.61 (m, 1H, C9-H), 3.92 – 3.83 (m, 4H, C19-H_2 + C20-H_2), 3.16 – 3.06 (m, 4H, C18-H_2 + C21-H_2), 2.50 – 2.39 (m, 1H, C4-H), 2.26 – 2.05 (m, 5H, C3-H + C5-H_2 + C8-H + C10-H), 1.99 (dq, *J* = 14.9, 2.8 Hz, 1H, C10-H), 1.70 – 1.59 (m, 2H, C4-H + C8-H), 1.47 – 1.31 (m, 2H, C7-H_2), 1.29 (s, 3H, C6-H_3).
^{13}C NMR (101 MHz, $CDCl_3$) δ ppm: 183.9 (C11), 148.6 (C16), 147.4 (C13), 140.3 (C12), 119.3 (C14), 115.5 (C15), 113.7 (C17), 74.3 (C9), 67.2 (C19 + C20), 65.8 (C1), 51.0 (C18 + C21), 44.6 (C2), 42.7 (C3), 36.4 (C10), 35.8 (C5), 32.4 (C4), 27.6 (C6), 26.5 (C7), 25.0 (C8).
LC-LRMS (ESI+) *m/z*: 339.09 found for $[M+H]^+$, 339.21 calcd. for $C_{21}H_{27}N_2O_2^+$, R_t = 1.19 min, purity (UV area): 98%.
HRMS (ESI+) *m/z*: 339.2067 found for $[M+H]^+$, 339.2067 calcd. for $C_{21}H_{27}N_2O_2^+$ (Δ = 0.00 ppm).
ICP-MS (Pd-108): 60 mg/kg (ppm).

Note: The peak for the C14-H proton at 7.26 ppm in the 1D ^{1}H NMR spectrum overlaps with the chloroform-*d* solvent peak, thus increasing the integral, but the chemical shift and integral were confirmed by correlations in the 2D ^{1}H-^{1}H COESY and ^{1}H-^{13}C HSQC and HMBC spectra.

(3*R,3a*S**,6*R**,12b*R**)-*N*-Benzyl-3a-methyl-2,3,3a,4,5,6-hexahydro-1*H*-3,6-methanocyclopenta[3,4]oxepino[2,3-*b*]indol-11-amine (19b)**

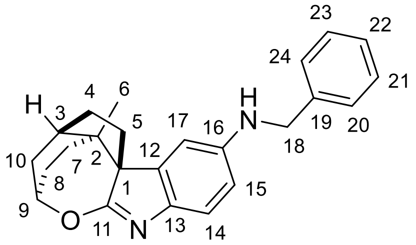

19b

(3*R,3a*S**,6*R**,12b*R**)-*N*-Benzyl-3a-methyl-*N*-((3*R**,3a*S**,6*R**,12b*R**)-3a-methyl-2,3,3a,4,5,6-hexahydro-1*H*-3,6-methanocyclopenta[3,4]oxepino[2,3-*b*]indol-11-yl)-2,3,3a,4,5,6-hexahydro-1*H*-3,6-methanocyclopenta[3,4]oxepino[2,3-*b*]indol-11-amine (20a)**

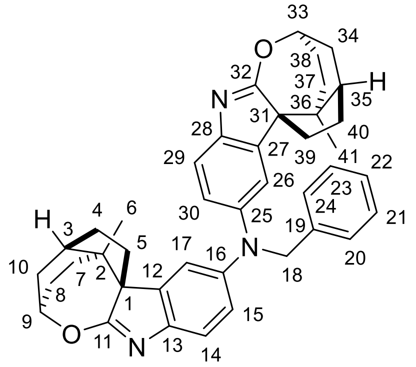

20a

Following general procedure I using compound **15b** (50.0 mg, 150 µmol) and benzylamine (20 µL, 183 µmol), stirring at 80 °C for 16 hours, scavenging for 23 hours, and purifying using FlashPure Select Silica (4 g) eluting with 1:0 to 0:1 *n*-heptane/ethyl acetate (flow rate = 12 mL/min) afforded compound **19b** (35.5 mg, 66% yield) as a cream-white solid.

Note: The diarylated side-product (**20a**; 9.4 mg, 20%) was isolated as a light-brown solid in separate fractions.

Characterisation data for **19b**:
TLC: R_f = 0.25 (3:2 ethyl acetate/*n*-heptane), UV.
mp = 168.4 – 169.9 °C.
IR (ATR) $\tilde{\upsilon}_{max}$ cm^{-1}: 3289 (s), 3121 (w), 3082 (w), 3047 (w), 3025 (w), 2941 (s), 2910 (m), 2875 (m), 2857 (m), 1735 (w), 1623 (m), 1574 (s), 1532 (m), 1494 (w), 1469 (s), 1390 (m), 1348 (m), 1315 (w), 1246 (s), 1212 (m), 1199 (m), 1173 (w), 1116 (m), 1089 (w), 1066 (w), 1051 (w), 1026 (w), 1000 (m), 956 (m), 906 (w), 888 (w), 812 (m), 785 (w), 730 (m), 696 (m), 642 (w), 599 (w), 568 (w).
^{1}H NMR (400 MHz, $CDCl_3$) δ ppm: 7.41 – 7.32 (m, 4H, C20-H + C21-H + C23-H + C24-H), 7.30 – 7.25 (m, 1H, C22-H), 7.18 (d, *J* = 8.2 Hz, 1H, C14-H), 6.60 – 6.52 (m, 2H, C15-H + C17-H), 4.63 – 4.57 (m, 1H, C9-H), 4.31 (s, 2H, C18-H_2), 2.47 – 2.36 (m, 1H, C4-H), 2.23 – 1.94 (m, 6H, C3-H + C5-H_2 + C8-H + C10-H_2), 1.68 – 1.57 (m, 2H, C4-H + C8-H), 1.44 – 1.30 (m, 2H, C7-H_2), 1.20 (s, 3H, C6-H_3).
^{13}C NMR (101 MHz, $CDCl_3$) δ ppm: 182.5 (C11), 145.3 (C16), 144.9 (C13), 140.5 (C12), 139.6 (C19), 128.8 (C21 + C23), 127.8 (C20 + C24), 127.4 (C22), 119.6 (C14), 111.7 (C15), 110.0 (C17), 74.2 (C9), 65.6 (C1), 49.3 (C18), 44.6 (C2), 42.8 (C3), 36.5 (C10), 36.0 (C5), 32.4 (C4), 27.3 (C6), 26.5 (C7), 25.0 (C8).
LC-LRMS (ESI+) *m/z*: 359.15 found for $[M+H]^+$, 359.21 calcd. for $C_{24}H_{27}N_2O^+$, R_t = 1.44 min, purity (UV area): 98%.
HRMS (ESI+) *m/z*: 359.2118 found for $[M+H]^+$, 359.2118 calcd. for $C_{24}H_{27}N_2O^+$ (Δ = 0.00 ppm).
ICP-MS (Pd-108): 65 mg/kg (ppm).

Note: The peak for the C22-H proton at 7.30 – 7.25 ppm in the 1D ^{1}H NMR spectrum overlaps with the chloroform-*d* solvent peak, thus increasing the integral, but the chemical shift and integral were confirmed by correlations in the 2D ^{1}H-^{1}H COESY and ^{1}H-^{13}C HSQC and HMBC spectra.

Characterisation data for **20a**:
TLC: R_f = 0.05 (3:2 ethyl acetate/*n*-heptane), UV.
dec pt = 72.0 – 76.0 °C.
IR (ATR) $\tilde{\upsilon}_{\max}$ cm^{-1}: 3060 (w), 3024 (w), 3024 (s), 2872 (m), 2854 (m), 1710 (w), 1613 (m), 1574 (s), 1467 (s), 1389 (m), 1353 (m), 1336 (w), 1313 (m), 1242 (s), 1214 (m), 1197 (m), 1116 (m), 1093 (w), 1049 (w), 1028 (w), 998 (m), 956 (m), 906 (w), 845 (w), 814 (w), 726 (m), 697 (w), 642 (w).
^{1}H NMR (400 MHz, $CDCl_3$) δ ppm: 7.37 – 7.33 (m, 2H, C20-H + C24-H), 7.31 – 7.26 (m, 2H, C21-H + C23-H), 7.24 – 7.18 (m, 3H, C14-H + C22-H+ C29-H), 6.95 – 6.88 (m, 4H, C15-H + C17-H + C26-H + C30-H), 5.04 – 4.85 (m, 2H, C18-H_2), 4.65 – 4.60 (m, 2H, C9-H + C33-H), 2.43 – 2.29 (m, 2H, C4-H + C40-H), 2.21 – 2.02 (m, 10H, C3-H + C5-H_2 + C8-H + C10-H + C34-H + C35-H + C38-H + C39-H_2), 2.00 – 1.92 (m, 2H, C10-H + C34-H), 1.69 – 1.56 (m, 4H, C4-H + C8-H + C38-H + C40-H), 1.44 – 1.29 (m, 4H, C7-H_2 + C37-H_2), 1.10 – 1.04 (m, 6H, C6-H_3 + C41-H_3).
^{13}C NMR (101 MHz, $CDCl_3$) δ ppm: 184.12 (C11/C32), 184.07 (C11/C32), 147.9 (C13/C28), 147.7 (C13/C28), 145.4 (C16/C25), 145.2 (C16/C25), 140.3 (C12/C27), 140.2 (C12/C27), 139.5 (C19), 128.7 (C21 + C23), 127.0 (C22), 126.9 (C20 + C24), 120.2 (C15/C30), 120.0 (C15/C30), 119.6 (C14/C29), 119.5 (C14/C29), 117.6 (C17/C26), 117.4 (C17/C26), 74.4 (C9 + C33), 65.73 (C1/C31), 65.67 (C1/C31), 57.7 (C18), 44.7 (C2 + C36), 42.64 (C3/C35), 42.62 (C3/C35), 36.4 (C10/C34), 36.3 (C10/C34), 35.7 (C5/C39), 35.6 (C5/C39), 32.4 (C4/C40), 32.3 (C4/C40), 27.6 (C6/C41), 27.5 (C6/C41), 26.53 (C7/C37), 26.49 (C7/C37), 25.0 (C8 + C38).
LC-LRMS (ESI+) *m/z*: 610.40 found for $[M+H]^+$, 610.34 calcd. for $C_{41}H_{44}N_3O_2^+$, R_t = 2.02 min, purity (UV area): ≥95%.
HRMS (ESI+) *m/z*: 610.3427 found for $[M+H]^+$, 610.3428 calcd. for $C_{41}H_{44}N_3O_2^+$ (Δ = -0.13 ppm).

Note: The peak for the C21-H and C23-H protons at 7.31 – 7.26 ppm in the 1D ^{1}H NMR spectrum overlaps with the chloroform-*d* solvent peak, thus increasing the integral, but the chemical shift and integral were confirmed by correlations in the 2D ^{1}H-^{1}H COESY and ^{1}H-^{13}C HSQC and HMBC spectra. The peaks for the C13 and C28 carbons at 147.9 and 147.7 ppm are of low intensity in the 1D ^{13}C NMR spectrum, but the chemical shifts were confirmed by correlations in the 2D ^{1}H-^{13}C HMBC spectrum. The splitting of the peaks for the C18-H_2 protons at 5.04 – 4.85 ppm in the 1D ^{1}H NMR spectrum and the C19, C20, C24, C18 carbons at 139.5, 126.9, and 57.7 ppm in the 1D ^{13}C NMR spectrum is probably due to rotamers.

(3*R,3a*S**,6*R**,12b*R**)-3a-Methyl-*N*-pentyl-2,3,3a,4,5,6-hexahydro-1*H*-3,6-methanocyclopenta[3,4]oxepino[2,3-*b*]indol-11-amine (19c)**

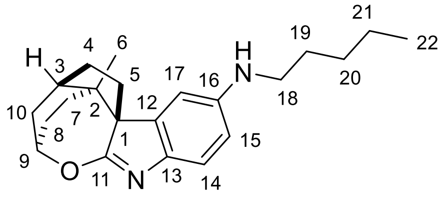

19c

(3*R,3a*S**,6*R**,12b*R**)-3a-Methyl-*N*-((3*R**,3a*S**,6*R**,12b*R**)-3a-methyl-2,3,3a,4,5,6-hexahydro-1*H*-3,6-methanocyclopenta[3,4]oxepino[2,3-*b*]indol-11-yl)-*N*-pentyl-2,3,3a,4,5,6-hexahydro-1*H*-3,6-methanocyclopenta[3,4]oxepino[2,3-*b*]indol-11-amine (20b)**

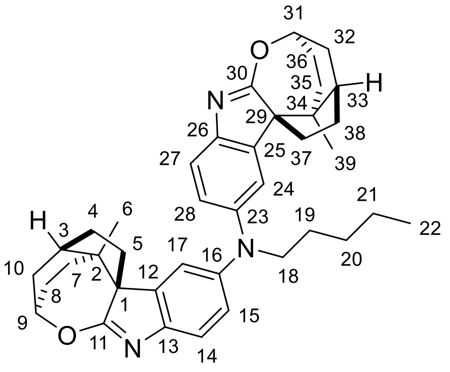

20b

Following general procedure I using compound **15b** (50.0 mg, 150 μmol) and *n*-amylamine (21 μL, 181 μmol), stirring at 80 °C for 16 hours, scavenging for 21 hours, and purifying using FlashPure Select Silica (4 g) eluting with 1:0 to 0:1 dichloromethane/ethyl acetate (flow rate = 12 mL/min) afforded compound **19c** (21.5 mg, 42% yield) as a light-green solid.

Note: The diarylated side-product (**20b**; 18.2 mg, 41%) was isolated as a green solid in separate fractions.

Characterisation data for **19c**:
TLC: R_f = 0.21 (4:1 dichloromethane/ethyl acetate), UV.
mp = 133.2 – 139.4 °C.
IR (ATR) $\tilde{\upsilon}_{\max}$ cm^{-1}: 3286 (m), 3047 (w), 2917 (s), 2853 (s), 1626 (m), 1578 (s), 1531 (m), 1463 (m), 1388 (m), 1351 (m), 1313 (w), 1245 (m), 1198 (m), 1138 (w), 1113 (m), 1092 (w), 1051 (w), 1001 (m), 957 (m), 885 (w), 801 (m), 644 (w).
^{1}H NMR (400 MHz, $CDCl_3$) δ ppm: 7.17 (d, *J* = 8.2 Hz, 1H, C14-H), 6.57 (d, *J* = 2.3 Hz, 1H, C17-H), 6.50 (dd, *J* = 8.2, 2.4 Hz, 1H, C15-H), 4.63 – 4.58 (m, 1H, C9-H), 3.09 (t, *J* = 7.1 Hz, 2H, C18-H_2), 2.50 – 2.38 (m, 1H, C4-H), 2.25 – 1.94 (m, 6H, C3-H + C5-H_2 + C8-H + C10-H_2), 1.70 – 1.58 (m, 4H, C4-H + C8-H + C19-H_2), 1.45 – 1.33 (m, 6H, C7-H_2 + C20-H_2 + C21-H_2), 1.28 (s, 3H, C6-H_3), 0.92 (t, *J* = 7.1 Hz, 3H, C22-H_3).
^{13}C NMR (101 MHz, $CDCl_3$) δ ppm: 182.3 (C11), 145.6 (C16), 144.6 (C13), 140.5 (C12), 119.6 (C14), 111.5 (C15), 109.9 (C17), 74.1 (C9), 65.6 (C1), 44.9 (C18), 44.6 (C2), 42.8 (C3), 36.5 (C10), 36.0 (C5), 32.4 (C4), 29.54 (C19), 29.49 (C20), 27.5 (C6), 26.5 (C7), 25.0 (C8), 22.7 (C21), 14.2 (C22).
LC-LRMS (ESI+) *m/z*: 339.13 found for $[M+H]^+$, 339.24 calcd. for $C_{22}H_{31}N_2O^+$, R_t = 1.37 min, purity (UV area): ≥99%.
HRMS (ESI+) *m/z*: 339.2430 found for $[M+H]^+$, 339.2431 calcd. for $C_{22}H_{31}N_2O^+$ (Δ = -0.23 ppm).
ICP-MS (Pd-108): 64 mg/kg (ppm).

Characterisation data for **20b**:
TLC: R_f = 0.16 (3:2 dichloromethane/ethyl acetate), UV.
dec pt = 112.0 – 115.0 °C.
IR (ATR) $\tilde{\upsilon}_{\max}$ cm^{-1}: 2949 (s), 2927 (s), 2870 (m), 2856 (m), 1710 (m). 1614 (m), 1574 (s), 1467 (s), 1389 (m), 1354 (w), 1336 (w), 1311 (w), 1241 (m), 1198 (m), 1114 (m), 1093 (w), 1050 (w), 998 (m), 956 (m), 921 (w), 910 (w), 813 (w), 725 (m), 642 (w).
^{1}H NMR (400 MHz, $CDCl_3$) δ ppm: 7.28 – 7.23 (m, 2H, C14-H + C27-H), 6.91 – 6.78 (m, 4H, C15-H + C17-H + C24-H + C28-H), 4.64 (quint, J = 3.1 Hz, 2H, C9-H + C31-H), 3.74 – 3.53 (m, 2H, C18-H_2), 2.45 – 2.32 (m, 2H, C4-H + C38-H), 2.23 – 2.03 (m, 10H, C3-H + C5-H_2 + C8-H + C10-H + C32-H + C33-H + C36-H + C37-H_2), 2.02 – 1.93 (m, 2H, C10-H + C32-H), 1.72 – 1.57 (m, 6H, C4-H + C8-H + C19-H_2 + C36-H + C38-H), 1.47 – 1.23 (m, 8H, C7-H_2 + C20-H_2 + C21-H_2 + C35-H_2), 1.19 – 1.12 (m, 6H, C6-H_3 + C39-H_3), 0.88 (t, *J* = 6.8 Hz, 3H, C22-H_3).
^{13}C NMR (101 MHz, $CDCl_3$) δ ppm: 184.0 (C11/C30), 183.9 (C11/C30), 147.3 (C13/C26), 147.2 (C13/C26), 145.4 (C16/C23), 145.3 (C16/C23), 140.24 (C12/C25), 140.18 (C12/C25), 120.1 (C15/C28), 119.9 (C15/C28), 119.6 (C14/C27), 119.5 (C14/C27), 117.7 (C17/C24), 117.5 (C17/C24), 74.5 (C9 + C31), 65.8 (C1/C29), 65.7 (C1/C29), 53.3 (C18), 44.7 (C2 + C34), 42.7 (C3/C33), 42.6 (C3/C33), 36.4

(C10/C32), 36.3 (C10/C32), 35.8 (C5/C37), 35.7 (C5/C37), 32.4 (C4 + C38), 29.5 (C20), 27.7 (C6/C39), 27.6 (C6/C39), 27.3 (C19), 26.54 (C7/C35), 26.52 (C7/C35), 25.0 (C8 + C36), 22.7 (C21), 14.2 (C22).
LC-LRMS (ESI+) *m/z*: 590.34 found for $[M+H]^+$, 590.37 calcd. for $C_{39}H_{48}N_3O_2^+$, R_t = 2.19 min, purity (UV area): ≥95%.
HRMS (ESI+) *m/z*: 590.3738 found for $[M+H]^+$, 590.3741 calcd. for $C_{39}H_{48}N_3O_2^+$ (Δ = -0.47 ppm).

Note: The peak for the C14-H and C27-H protons at 7.28 – 7.23 ppm in the 1D ^{1}H NMR spectrum overlaps with the chloroform-*d* solvent peak, thus increasing the integral, but the chemical shift and integral were confirmed by correlations in the 2D ^{1}H-^{1}H COESY and ^{1}H-^{13}C HSQC and HMBC spectra. The peaks for the C13 and C26 carbons at 147.3 and 147.2 ppm are of low intensity in the 1D ^{13}C NMR spectrum, but the chemical shifts were confirmed by correlations in the 2D ^{1}H-^{13}C HMBC spectrum. The splitting of the peaks for the C18-H_2 protons at 3.74 – 3.53 ppm in the 1D ^{1}H NMR spectrum and the C18 and C19 carbons at 53.3 and 27.3 ppm in the 1D ^{13}C NMR spectrum is probably due to rotamers.

(3*R,3a*S**,6*R**,12b*R**)-*N*-Butyl-*N*,3a-dimethyl-2,3,3a,4,5,6-hexahydro-1*H*-3,6-methanocyclopenta[3,4]oxepino[2,3-*b*]indol-11-amine (19d)**

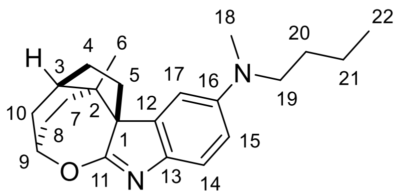

19d

Following general procedure I using compound **15b** (50.0 mg, 150 µmol) and *N*-methyl-*n*-butylamine (22 µL, 186 µmol), adding additional *N*-methyl-*n*-butylamine (2.4 equiv.), stirring at 80 °C for 30 hours and then at 100 °C for 13 hours, scavenging for 21 hours, and purifying using FlashPure Select Silica (4 g) eluting with 1:0 to 0:1 *n*-heptane/ethyl acetate (flow rate = 12 mL/min) afforded compound **19d** (37.6 mg, 74% yield) as an amber oil.

TLC: R_f = 0.25 (1:1 *n*-heptane/ethyl acetate), UV.
IR (ATR) $\tilde{\upsilon}_{max}$ cm^{-1}: 2950 (s), 2929 (s), 2870 (m), 1617 (m), 1576 (s), 1484 (m), 1460 (m), 1384 (m), 1353 (m), 1310 (w), 1285 (m), 1243 (m), 1211 (m), 1197 (m), 1112 (m), 1091 (w), 1050 (w), 999 (m), 952 (m), 929 (m), 846 (w), 800 (m), 646 (w).
^{1}H NMR (400 MHz, $CDCl_3$) δ ppm: 7.21 (d, *J* = 8.4 Hz, 1H, C14-H), 6.67 (d, *J* = 2.5 Hz, 1H, C17-H), 6.60 (dd, *J* = 8.5, 2.6 Hz, 1H, C15-H), 4.63 – 4.58 (m, 1H, C9-H), 3.35 – 3.19 (m, 2H, C19-H_2), 2.91 (s, 3H, C18-H_3), 2.51 – 2.40 (m, 1H, C4-H), 2.26 – 1.95 (m, 6H, C3-H + C5-H_2 + C8-H + C10-H_2), 1.71 – 1.51 (m, 4H, C4-H + C8-H + C20-H_2), 1.43 – 1.31 (m, 4H, C7-H_2 + C21-H_2), 1.29 (s, 3H, C6-H_3), 0.94 (t, *J* = 7.3 Hz, 3H, C22-H_3).
^{13}C NMR (101 MHz, $CDCl_3$) δ ppm: 182.4 (C11), 147.1 (C16), 143.8 (C13), 140.3 (C12), 119.4 (C14), 111.7 (C15), 109.7 (C17), 74.1 (C9), 65.7 (C1), 53.7 (C19), 44.6 (C2), 42.8 (C3), 39.2 (C18), 36.5 (C10), 36.0 (C5), 32.4 (C4), 29.0 (C20), 27.4 (C6), 26.5 (C7), 25.0 (C8), 20.5 (C21), 14.2 (C22).
LC-LRMS (ESI+) *m/z*: 339.44 found for $[M+H]^+$, 339.24 calcd. for $C_{22}H_{31}N_2O^+$, R_t = 1.19 min, purity (UV area): 95%.
HRMS (ESI+) *m/z*: 339.2427 found for $[M+H]^+$, 339.2431 calcd. for $C_{22}H_{31}N_2O^+$ (Δ = -1.11 ppm).
ICP-MS (Pd-108): 94 mg/kg (ppm).

***tert*-Butyl 4-((3*R**,3a*S**,6*R**,12b*R**)-3a-methyl-2,3,3a,4,5,6-hexahydro-1*H*-3,6-methanocyclopenta[3,4]oxepino[2,3-*b*]indol-11-yl)piperazine-1-carboxylate (19e)**

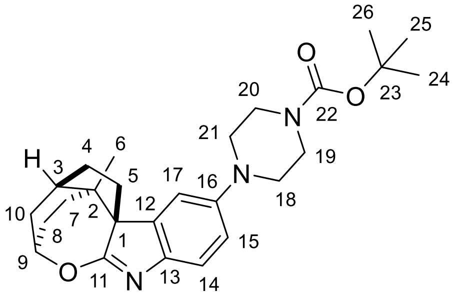

19e

Following general procedure I using compound **15b** (175 mg, 0.527 mmol) and *N*-Boc-piperazine (118 mg, 0.634 mmol), stirring at 80 °C for eight hours, scavenging for 18 hours, and purifying using FlashPure Select Silica (12 g) eluting with 1:0 to 0:1 dichloromethane/ethyl acetate (flow rate = 24 mL/min) afforded compound **19e** (228 mg, 99% yield) as a light-yellow solid.

TLC: R_f = 0.20 (2:1 dichloromethane/ethyl acetate), UV.
mp = 68.0 – 69.8 °C.
IR (ATR) $\tilde{\upsilon}_{\max}$ cm^{-1}: 2930 (m), 2857 (m), 2816 (w), 1736 (w), 1690 (s), 1615 (w), 1577 (s), 1471 (m), 1458 (m), 1419 (m), 1389 (m), 1364 (m), 1337 (w), 1309 (w), 1284 (m), 1241 (s), 1163 (s), 1119 (m), 1093 (m), 1037 (m), 998 (m), 960 (w), 926 (m), 881 (w), 863 (w), 816 (w), 769 (w), 743 (w), 650 (w).
^{1}H NMR (400 MHz, $CDCl_3$) δ ppm: 7.27 – 7.23 (m, 1H, C14-H), 6.90 (d, *J* = 2.4 Hz, 1H, C17-H), 6.82 (dd, *J* = 8.3, 2.4 Hz, 1H, C15-H), 4.66 – 4.61 (m, 1H, C9-H), 3.59 (dd, *J* = 6.5, 4.0 Hz, 4H, C19-H_2 + C20-H_2), 3.12 – 3.00 (m, 4H, C18-H_2 + C21-H_2), 2.51 – 2.37 (m, 1H2, C4-H), 2.27 – 2.05 (m, 5H, C3-H + C5-H_2 + C8-H + C10-H), 1.99 (dq, *J* = 14.9, 2.8 Hz, 1H, C10-H), 1.69 – 1.60 (m, 2H, C4-H + C8-H), 1.48 (s, 9H, C24-H_3 + C25-H_3 + C26-H_3), 1.45 – 1.32 (m, 2H, C7-H_2), 1.29 (s, 3H, C6-H_3).
^{13}C NMR (101 MHz, $CDCl_3$) δ ppm: 184.0 (C11), 154.9 (C22), 148.7 (C16), 147.7 (C13), 140.3 (C12), 119.3 (C14), 116.5 (C15), 114.8 (C17), 80.0 (C23), 74.3 (C9), 65.8 (C1), 51.1 (C18 + C21), 44.6 (C2), 43.7 (C19 + C20), 42.7 (C3), 36.4 (C10), 35.8 (C5), 32.4 (C4), 28.6 (C24 + C25 + C26), 27.6 (C6), 26.5 (C7), 25.0 (C8).
LC-LRMS (ESI+) *m/z*: 438.57 found for $[M+H]^+$, 438.28 calcd. for $C_{26}H_{36}N_3O_3^+$, R_t = 1.78 min, purity (UV area): 98%.
HRMS (ESI+) *m/z*: 438.2751 found for $[M+H]^+$, 438.2751 calcd. for $C_{26}H_{36}N_3O_3^+$ (Δ = 0.00 ppm).
ICP-MS (Pd-108): 292 mg/kg (ppm).

Note: The peak for the C14-H proton at 7.27 – 7.23 ppm in the 1D ^{1}H NMR spectrum overlaps with the chloroform-*d* solvent peak, thus increasing the integral, but the chemical shift and integral were confirmed by correlations in the 2D ^{1}H-^{1}H COESY and ^{1}H-^{13}C HSQC and HMBC spectra. The peak for the C19+C20 carbons at 43.7 ppm is of low intensity in the 1D ^{13}C NMR spectrum, but the chemical shift was confirmed by correlations in the 2D ^{1}H-^{13}C HSQC and HMBC spectra.

Unsuccessful analogues

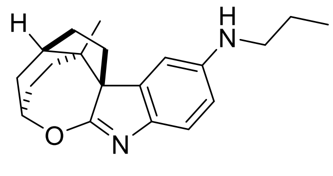

19f

Note: Primarily diarylated side-product (double coupling of **15b** to *n*-propylamine). No purification attempted at this point.

General procedure J

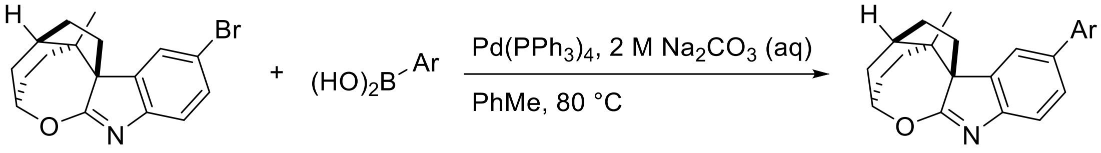

15b **21**

A mixture of compound **15b** (1.00 equiv.), the appropriate aryl boronic acid (1.50 equiv.), and $Pd(PPh_3)_4$ (0.05 equiv. = 5 mol%) was evacuated and backfilled with argon three times. The mixture was added toluene (55 mM **15b**) and 2 M aqueous Na_2CO_3 (3.00 equiv.) and bubbled with argon for 15 minutes at room temperature. The reaction mixture was stirred at 80 °C under an argon atmosphere for the indicated time. If necessary, additional aryl boronic acid was added during the reaction. The reaction mixture was cooled to room temperature, diluted with ethyl acetate, and filtered through a pad of celite® washing with ethyl acetate. The filtrate was concentrated under reduced pressure to yield the initial crude products. The initial crude products were scavenged for palladium following the general procedure for palladium-scavenging to yield the final crude products. The final crude products were purified by flash column chromatography on BÜCHI® system to afford the spirooxepinoindole analogues (**21**).

(3*R,3a*S**,6*R**,12b*R**)-3a-Methyl-11-phenyl-2,3,3a,4,5,6-hexahydro-1*H*-3,6-methanocyclopenta[3,4]oxepino[2,3-*b*]indole (21a)**

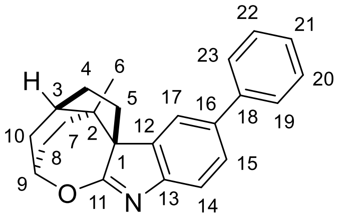

21a

Following general procedure J using compound **15b** (50.0 mg, 150 μmol) and phenylboronic acid (27.5 mg, 0.226 mmol), stirring for 18 hours, scavenging for 20 hours, and purifying using FlashPure EcoFlex Silica (12 g) eluting with 1:0 to 0:1 dichloromethane/ethyl acetate (flow rate = 20 mL/min) afforded compound **21a** (37.1 mg, 75% yield) as a white solid.

TLC: R_f = 0.24 (12:1 dichloromethane/ethyl acetate), UV.
mp = 148.0 – 150.5 °C.
IR (ATR) $\tilde{\upsilon}_{\max}$ cm^{-1}: 3057 (w), 3030 (w), 2944 (m), 2873 (m), 2856 (m), 1734 (w), 1614 (w), 1567 (s), 1461 (s), 1418 (w), 1389 (m), 1354 (w), 1313 (m), 1283 (w), 1241 (s), 1198 (m), 1119 (w), 1093 (w), 1043 (w), 997 (m), 952 (m), 831 (m), 771 (m), 755 (m), 697 (m).
^{1}H NMR (400 MHz, $CDCl_3$) δ ppm: 7.59 – 7.54 (m, 2H, C19-H + C23-H), 7.50 – 7.40 (m, 5H, C14-H + C15-H + C17-H + C20-H + C22-H), 7.35 – 7.30 (m, 1H, C21-H), 4.71 – 4.65 (m, 1H, C9-H), 2.54 – 2.43 (m, 1H, C4-H), 2.35 – 2.09 (m, 5H, C3-H + C5-H_2 + C8-H + C10-H), 2.02 (dq, *J* = 15.0, 2.9 Hz, 1H, C10-H), 1.73 – 1.62 (m, 2H, C4-H + C8-H), 1.52 – 1.39 (m, 2H, C7-H_2), 1.37 (s, 3H, C6-H_3).
^{13}C NMR (101 MHz, $CDCl_3$) δ ppm: 185.7 (C11), 153.4 (C13), 141.8 (C18), 139.9 (C12), 137.2 (C16), 128.9 (C20 + C22), 127.3 (C19 + C23), 127.00 (C21), 126.96 (C15), 123.0 (C17), 119.4 (C14), 74.6 (C9), 65.7 (C1), 44.6 (C2), 42.7 (C3), 36.3 (C10), 35.7 (C5), 32.4 (C4), 27.9 (C6), 26.5 (C7), 25.1 (C8).
LC-LRMS (ESI+) *m/z*: 330.05 found for $[M+H]^+$, 330.19 calcd. for $C_{23}H_{24}NO^+$, R_t = 1.96 min, purity (UV area): ≥95%.
HRMS (ESI+) *m/z*: 330.1852 found for $[M+H]^+$, 330.1852 calcd. for $C_{23}H_{24}NO^+$ (Δ = 0.00 ppm).
ICP-MS (Pd-108): 178 mg/kg (ppm).

(3*R,3a*S**,6*R**,12b*R**)-11-(4-Methoxyphenyl)-3a-methyl-11-phenyl-2,3,3a,4,5,6-hexahydro-1*H*-3,6-methanocyclopenta[3,4]oxepino[2,3-*b*]indole (21b)**

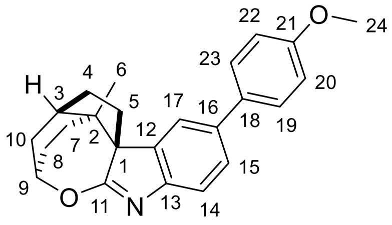

21b

Following general procedure J using compound **15b** (50.0 mg, 150 μmol) and 4-methoxyphenylboronic acid (34.4 mg, 0.226 mmol), adding additional 4-methoxyphenylboronic acid (1.5 equiv.), stirring for 40 hours, scavenging for 21 hours, and purifying using FlashPure Select Silica (4 g) eluting with 1:0 to 0:1 dichloromethane/ethyl acetate (flow rate = 12 mL/min) afforded compound **21b** (31.0 mg, 57% yield) as a cream-white solid.

TLC: R_f = 0.26 (10:1 dichloromethane/ethyl acetate), UV.
mp = 181.5 – 183.5 °C.
IR (ATR) $\tilde{\upsilon}_{max}$ cm^{-1}: 3048 (w), 2996 (w), 2950 (m), 2905 (m), 2878 (m), 2854 (w), 2838 (w), 1605 (w), 1563 (s), 1514 (m), 1462 (s), 1439 (m), 1397 (m), 1309 (m), 1273 (m), 1244 (s), 1223 (m), 1202 (m), 1177 (m), 1120 (w), 1094 (w), 1036 (m), 1020 (m), 997 (m), 973 (w), 953 (m), 919 (w), 846 (w), 824 (s), 793 (m), 581 (w), 534 (w), 516 (w).
^{1}H NMR (400 MHz, $CDCl_3$) δ ppm: 7.52 – 7.47 (m, 2H, C19-H + C23-H), 7.45 – 7.36 (m, 3H, C14-H + C15-H + C17-H), 7.00 – 6.94 (m, 2H, C20-H + C22-H), 4.71 – 4.64 (m, 1H, C9-H), 3.85 (s, 3H, C24-H_3), 2.54 – 2.42 (m, 1H, C4-H), 2.35 – 2.08 (m, 5H, C3-H + C5-H_2 + C8-H + C10-H), 2.01 (dq, *J* = 14.9, 2.8 Hz, 1H, C10-H), 1.72 – 1.62 (m, 2H, C4-H + C8-H), 1.52 – 1.38 (m, 2H, C7-H_2), 1.36 (s, 3H, C6-H_3).
^{13}C NMR (101 MHz, $CDCl_3$) δ ppm: 185.4 (C11), 159.0 (C21), 152.9 (C13), 139.9 (C12), 136.9 (C16), 134.5 (C18), 128.3 (C19 + C23), 126.5 (C15), 122.6 (C17), 119.4 (C14), 114.3 (C20 + C22), 74.6 (C9), 65.7 (C1), 55.5 (C24), 44.7 (C2), 42.7 (C3), 36.4 (C10), 35.7 (C5), 32.4 (C4), 27.8 (C6), 26.5 (C7), 25.1 (C8).
LC-LRMS (ESI+) *m/z*: 360.11 found for $[M+H]^+$, 360.20 calcd. for $C_{24}H_{26}NO_2^+$, R_t = 1.88 min, purity (UV area): 100%.
HRMS (ESI+) *m/z*: 360.1959 found for $[M+H]^+$, 360.1958 calcd. for $C_{24}H_{26}NO_2^+$ (Δ = 0.34 ppm).
ICP-MS (Pd-108): 65 mg/kg (ppm).

Unsuccessful analogues

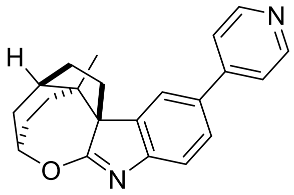

21c

Note: Poor conversion into **21c**. No purification attempted at this point.

(3*R,3a*S**,6*R**,12b*R**)-3a-Methyl-11-(piperazin-1-yl)-2,3,3a,4,5,6-hexahydro-1*H*-3,6-methanocyclopenta[3,4]oxepino[2,3-*b*]indole (22)**

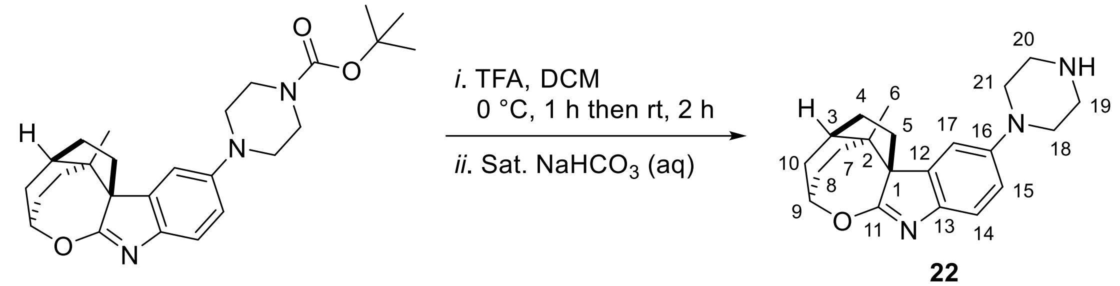

19e **22** 53%

To a solution of compound **19e** (197 mg, 0.450 mmol, 1.0 equiv.) in dry dichloromethane (5.0 mL) at 0 °C was added trifluoroacetic acid (346 µL, 4.52 mmol, 10.0 equiv.) portionwise over one hour. The reaction mixture was warmed to room temperature and additional trifluoroacetic acid (519 µL, 6.78 mmol, 15.0 equiv.) was added portionwise over one hour. Then the reaction mixture was stirred at room temperature for one hour. The reaction mixture was quenched with saturated aqueous $NaHCO_3$ (5 mL) and the phases were separated. The aqueous phase was adjusted to pH 8-9 with saturated aqueous $NaHCO_3$ and extracted with dichloromethane (3 × 25 mL). The combined organic phases were dried over anhydrous $MgSO_4$ and the solvent removed under reduced pressure to yield a crude dark-green sticky solid. The crude product was purified by flash column chromatography on BÜCHI® system using FlashPure Select Silica (12 g; 0:1 to 1:0 methanol/48:1:1 dichloromethane/methanol/triethylamine; flow rate = 24 mL/min) to yield the slightly impure product as a brown oil. The impure product was dissolved in dichloromethane (25 mL), washed with 0.01 M aqueous NaOH (3 × 10 mL) (to remove trifluoroacetic acid-triethylamine salt) and brine (10 mL), dried over anhydrous $MgSO_4$, and the solvent removed under reduced pressure to yield an off-white solid. The residue was purified twice by flash column chromatography on BÜCHI® system using FlashPure Select Silica (1. 4 g; 0:1 to 1:0 methanol/96:3:1 dichloromethane/methanol/triethylamine; flow rate = 12 mL/min and 2. 12 g; 0:1 to 1:0 methanol/48:1:1 dichloromethane/methanol/triethylamine; flow rate = 24 mL/min) to afford compound **22** (80.4 mg, 53% yield) as a white solid.

TLC: R_f = 0.24 (48:1:1 dichloromethane/methanol/triethylamine), UV.
mp = 75.0 – 78.0 °C.
IR (ATR) $\tilde{\upsilon}_{\max}$ cm^{-1}: 3277 (w), 2941 (s), 2848 (m), 2814 (m), 2751 (w), 1615 (m), 1574 (s), 1470 (m), 1554 (m), 1388 (m), 1355 (w), 1334 (w), 1308 (w), 1241 (m), 1197 (m), 1144 (m), 1114 (m), 1093 (w), 1050 (w), 1038 (w), 1021 (w), 997 (m), 958 (m), 928 (m), 871 (m), 813 (m), 729 (w), 654 (w).
^{1}H NMR (400 MHz, CD_3OD) δ ppm: 7.12 (d, *J* = 8.4 Hz, 1H, C14-H), 7.02 (d, *J* = 2.4 Hz, 1H, C17-H), 6.89 (dd, *J* = 8.4, 2.4 Hz, 1H, C15-H), 4.65 – 4.60 (m, 1H, C9-H), 3.13 – 3.06 (m, 4H, C18-H_2 + C21-H_2), 3.02 – 2.96 (m, 4H, C19-H_2 + C20-H_2), 2.58 – 2.47 (m, 1H, C4-H), 2.31 – 2.16 (m, 3H, C3-H + C5-H + C10-H), 2.13 – 1.93 (m, 3H, C5-H + C8-H + C10-H), 1.80 – 1.70 (m, 1H, C8-H), 1.68 – 1.60 (m, 1H, C4-H), 1.56 – 1.47 (m, 1H, C7-H), 1.32 (s, 3H, C6-H_3), 1.25 (ddd, *J* = 14.8, 11.0, 4.4 Hz, 1H, C7-H).
^{13}C NMR (101 MHz, CD_3OD) δ ppm: 186.3 (C11), 150.8 (C16), 147.0 (C13), 141.0 (C12), 119.3 (C14), 117.5 (C15), 115.8 (C17), 76.4 (C9), 67.0 (C1), 52.4 (C18 + C21), 46.6 (C19 + C20), 45.6 (C2), 43.8 (C3), 37.0 (C10), 36.9 (C5), 33.1 (C4), 27.9 (C6), 27.4 (C7), 25.9 (C8).
LC-LRMS (ESI+) *m/z*: 338.17 found for $[M+H]^+$, 338.22 calcd. for for $C_{21}H_{28}N_3O^+$, R_t = 0.74 min, purity (UV area): 100%.
HRMS (ESI+) *m/z*: 338.2227 found for $[M+H]^+$, 338.2227 calcd. for $C_{21}H_{28}N_3O^+$ (Δ = 0.00 ppm).

Note: LC-LRMS indicates a purity of 100% (please see LC-LRMS trace) but unknown impurities are observed in the 1D ^{13}C NMR spectrum. Residual dichloromethane observed in the 1D ^{1}H NMR spectrum.

1-(4-((3R*,3aS*,6R*,12bR*)-3a-Methyl-2,3,3a,4,5,6-hexahydro-1H-3,6-methanocyclopenta[3,4]oxepino[2,3-*b*]indol-11-yl)piperazin-1-yl)butan-1-one (23)

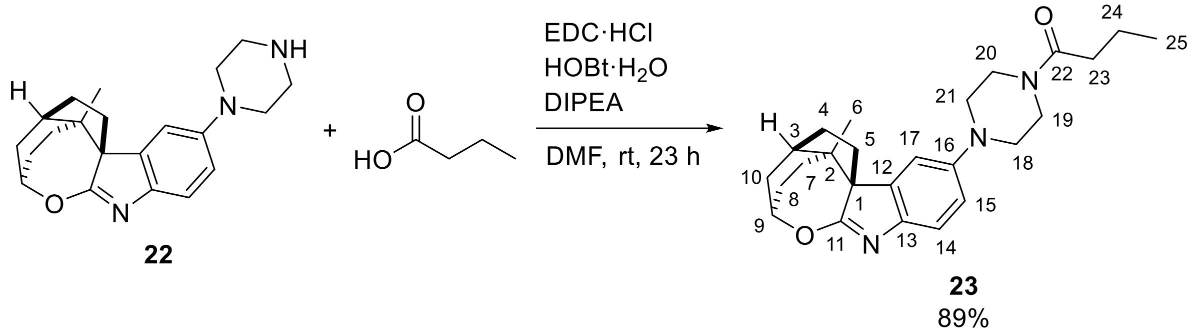

A solution of butyric acid (9.0 µL, 97 µmol, 1.3 equiv.), *N*-ethyl-*N'*-(3-dimethylaminopropyl)carbodiimide hydrochloride (21.5 mg, 112 µmol, 1.5 equiv.), 1-hydroxybenzotriazole monohydrate (17.2 mg, 112 µmol, 1.5 equiv.), and *N*,*N*-diisopropylethylamine (38.8 µL, 223 µmol, 3.0 equiv.) in dimethylformamide (2.0 mL) was stirred at room temperature for five minutes and then added compound **22** (25.0 mg, 74.0 µmol, 1.0 equiv.). The reaction mixture was stirred at room temperature for 23 hours. The reaction mixture was diluted with ethyl acetate (15 mL) and washed with saturated aqueous $NaHCO_3$ (5 mL) and brine (3 × 5 mL). The combined aqueous phases were back-extracted with ethyl acetate (15 mL). The combined organic phases were dried over anhydrous Na_2SO_4 and the solvent removed under reduced pressure (co-evaporation with *n*-heptane) to yield a crude light-green sticky solid. The crude product was purified by flash column chromatography on BÜCHI® system using FlashPure Select Silica (4 g; 1:0 to 0:1 dichloromethane/ethyl acetate; flow rate = 12 mL/min) to afford compound **23** (27.0 mg, 89% yield) as a white solid.

TLC: R_f = 0.23 (4:1 dichloromethane/ethyl acetate), UV.
mp = 49.5 – 51.2 °C.
IR (ATR) $\tilde{\upsilon}_{\max}$ cm^{-1}: 3481 (w), 2954 (m), 2872 (m), 2813 (m), 1638 (s), 1575 (s), 1464 (m), 1434 (m), 1387 (m), 1355 (w), 1336 (m), 1308 (w), 1232 (m), 1153 (m), 1117 (w), 1093 (w), 1028 (m), 998 (m), 978 (w), 950 (m), 924 (w), 876 (w).
^{1}H NMR (400 MHz, $CDCl_3$) δ ppm: 7.26 (d, *J* = 8.3 Hz, 1H, C14-H), 6.90 (d, *J* = 2.4 Hz, 1H, C17-H), 6.82 (dd, *J* = 8.4, 2.4 Hz, 1H, C15-H), 4.66 – 4.59 (m, 1H, C9-H), 3.79 (t, *J* = 5.2 Hz, 2H, C19-H_2), 3.63 (td, *J* = 4.5, 1.6 Hz, 2H, C20-H_2), 3.15 – 3.02 (m, 4H, C18-H_2 + C21-H_2), 2.51 – 2.40 (m, 1H, C4-H), 2.35 (t, *J* = 7.5 Hz, 2H, C23-H_2), 2.27 – 2.05 (m, 5H, C3-H + C5-H_2 + C8-H + C10-H), 1.99 (dq, *J* = 14.9, 2.9 Hz, 1H, C10-H), 1.74 – 1.59 (m, 4H, C4-H + C8-H + C24-H_2), 1.47 – 1.33 (m, 2H, C7-H_2), 1.29 (s, 3H, C6-H_3), 0.99 (t, *J* = 7.4 Hz, 3H, C25-H_3).
^{13}C NMR (101 MHz, $CDCl_3$) δ ppm: 184.1 (C11), 171.7 (C22), 148.4 (C16), 147.9 (C13), 140.4 (C12), 119.4 (C14), 116.6 (C15), 114.8 (C17), 74.4 (C9), 65.8 (C1), 51.5 (C21), 51.2 (C18), 45.9 (C20), 44.6 (C2), 42.7 (C3), 41.8 (C19), 36.4 (C10), 35.8 (C5), 35.4 (C23), 32.4 (C4), 27.6 (C6), 26.5 (C7), 25.0 (C8), 18.9 (C24), 14.2 (C25).
^{1}H NMR (400 MHz, DMSO-d_6) δ ppm: 7.07 (d, *J* = 8.3 Hz, 1H, C14-H), 7.00 (d, *J* = 2.4 Hz, 1H, C17-H), 6.78 (dd, *J* = 8.4, 2.4 Hz, 1H, C15-H), 4.55 (quint, *J* = 3.4 Hz, 1H, C9-H), 3.64 – 3.53 (m, 4H, C19-H_2 + C20-H_2), 3.10 – 2.96 (m, 4H, C18-H_2 + C21-H_2), 2.45 – 2.36 (m, 1H, C4-H), 2.32 (t, *J* = 7.4 Hz, 2H, C23-H_2), 2.22 – 2.05 (m, 3H, C3-H + C5-H + C10-H), 1.98 – 1.88 (m, 1H, C5-H), 1.87 – 1.78 (m, 2H, C8-H + C10-H), 1.73 – 1.59 (m, 1H, C8-H), 1.58 – 1.47 (m, 3H, C4-H + C24-H_2), 1.46 – 1.34 (m, 1H, C7-H), 1.22 (s, 3H, C6-H_3), 1.07 (ddd, *J* = 14.7, 10.5, 4.0 Hz, 1H, C7-H), 0.90 (t, *J* = 7.4 Hz, 3H, C25-H_3).
^{13}C NMR (101 MHz, DMSO-d_6) δ ppm: 182.8 (C11), 170.5 (C22), 148.1 (C16), 146.6 (C13), 140.1 (C12), 118.2 (C14), 115.3 (C15), 114.2 (C17), 73.5 (C9), 65.0 (C1), 50.2 (C21), 49.9 (C18), 44.9 (C20), 43.9 (C2), 41.8 (C3), 41.0 (C19), 35.6 (C10), 35.0 (C5), 34.2 (C23), 31.9 (C4), 27.1 (C6), 26.1 (C7), 24.3 (C8), 18.2 (C24), 13.8 (C25).
^{15}N NMR (61 MHz, DMSO-d_6) δ ppm: 114.6 (C22(O)-N), 63.7 (C16-N).
LC-LRMS (ESI+) *m/z*: 408.58 found for $[M+H]^+$, 408.26 calcd. for $C_{25}H_{34}N_3O_2^+$, R_t = 1.35 min, purity (UV area): ≥95%.
HRMS (ESI+) *m/z*: 408.2646 found for $[M+H]^+$, 408.2646 calcd. for $C_{25}H_{34}N_3O_2^+$ (Δ = 0.00 ppm).

Note: The peak for the C14-H proton at 7.26 ppm in the 1D ^{1}H NMR spectrum overlaps with the chloroform-*d* solvent peak, thus increasing the integral, but the chemical shift and integral were

confirmed by correlations in the 2D ^{1}H-^{1}H COESY and ^{1}H-^{13}C HSQC and HMBC spectra. The splitting of the C19-H_2 and C20-H_2 in the 1D ^{1}H NMR spectrum in chloroform-*d* and individual peaks for C18, C19, C20, and C21 in the 1D ^{13}C NMR spectrum are probably due to the limited rotation of the C22-N bond due to its double bond character and limited interconversion (ring flip) between piperazine ring chair conformations resulting in different chemical environment which is known for acylpiperazines.[17]

(3*R,3a*S**,6*R**,12b*R**)-3a-Methyl-11-(4-(pyridin-4-ylmethyl)piperazin-1-yl)-2,3,3a,4,5,6-hexahydro-1H-3,6-methanocyclopenta[3,4]oxepino[2,3-b]índole (24)**

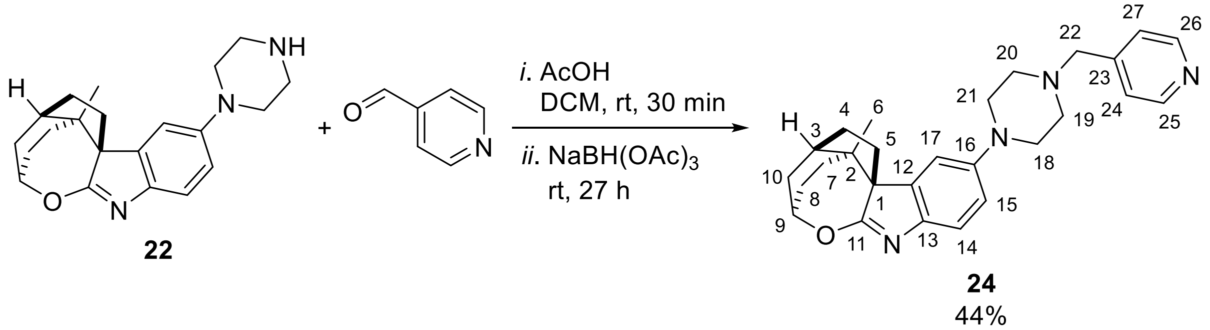

A solution of compound **22** (25.0 mg, 74.0 µmol, 1.0 equiv.), isonicotinaldehyde (7.7 µL, 82 µmol**,** 1.1 equiv.), and glacial acetic acid (3.7 µL, 65 µmol, 0.9 equiv.) in dry dichloromethane (1.8 mL) was stirred at room temperature for 30 minutes and then added $NaBH(OAc)_3$ (22.1 mg, 104 µmol, 1.4 equiv.). The reaction mixture was stirred at room temperature for four hours. Then additional isonicotinaldehyde (6.3 µL, 67 µmol**,** 0.9 equiv.) was added. Then the reaction mixture was stirred at room temperature for four hours. Then additional isonicotinaldehyde (7.0 µL, 74 µmol**,** 1.0 equiv.) $NaBH(OAc)_3$ (25.1 mg, 118 µmol, 1.6 equiv.) were added. Then the reaction mixture was stirred at room temperature for 15 hours. Then additional isonicotinaldehyde (7.0 µL, 74 µmol**,** 1.0 equiv.) $NaBH(OAc)_3$ (15.7 mg, 74.0 µmol, 1.0 equiv.) were added. Then the reaction mixture was stirred at room temperature for four hours. The reaction mixture was quenched with saturated aqueous $NaHCO_3$ (5 mL), added dichloromethane (15 mL), and the phases separated. The aqueous phase was adjusted to pH 9-10 with saturated aqueous Na_2CO_3 and extracted with dichloromethane (2 × 15 mL). The combined organic phases were washed with brine (10 mL), dried over anhydrous $MgSO_4$, and the solvent removed under reduced pressure to yield a crude green oil. The crude product was purified by flash column chromatography on BÜCHI® system using FlashPure Select Silica (4 g; 1:0 to 0:1 dichloromethane/methanol; flow rate = 12 mL/min) to afford compound **24** (13.9 mg, 44% yield) as an orange sticky solid.

TLC: R_f = 0.24 (24:1 dichloromethane/methanol), UV.
IR (ATR) $\tilde{\upsilon}_{\max}$ cm^{-1}: 3026 (w), 2941 (m), 2877 (m), 2854 (m), 2815 (m), 2773 (w), 1576 (s), 1471 (m), 1453 (m), 1414 (m), 1389 (m), 1355 (w), 1335 (w), 1321 (w), 1301 (w), 1242 (m), 1197 (m), 1142 (m), 1119 (w), 1094 (w), 1051 (w), 1011 (w), 995 (m), 960 (w), 928 (m), 883 (w), 852 (w), 815 (m), 792 (m), 726 (m), 643 (w).
^{1}H NMR (400 MHz, $CDCl_3$) δ ppm: 8.60 – 8.52 (m, 2H, C25-H + C26-H), 7.34 – 7.28 (m, 2H, C24-H + C27-H), 7.24 (d, *J* = 8.3 Hz, 1H, C14-H), 6.90 (d, *J* = 2.4 Hz, 1H, C17-H), 6.83 (dd, *J* = 8.4, 2.4 Hz, 1H, C15-H), 4.65 – 4.57 (m, 1H, C9-H), 3.58 (s, 2H, C22-H_2), 3.21 – 3.09 (m, 4H, C18-H_2 + C21-H_2), 2.68 – 2.58 (m, 4H, C19-H_2 + C20-H_2), 2.49 – 2.38 (m, 1H, C4-H), 2.26 – 2.04 (m, 5H, C3-H + C5-H_2 + C8-H + C10-H), 1.99 (dq, *J* = 14.8, 2.8 Hz, 1H, C10-H), 1.69 – 1.58 (m, 2H, C4-H + C8-H), 1.48 – 1.33 (m, 2H, C7-H_2), 1.28 (s, 3H, C6-H_3).
^{13}C NMR (101 MHz, $CDCl_3$) δ ppm: 183.8 (C11), 150.0 (C25 + C26), 148.7 (C16), 147.6 (C23), 147.3 (C13), 140.2 (C12), 124.0 (C24 + C27), 119.3 (C14), 115.9 (C15), 114.2 (C17), 74.3 (C9), 65.8 (C1), 61.9 (C22), 53.5 (C19 + C20), 50.8 (C18 + C21), 44.6 (C2), 42.7 (C3), 36.4 (C10), 35.8 (C5), 32.4 (C4), 27.6 (C6), 26.5 (C7), 25.0 (C8).
LC-LRMS (ESI+) *m/z*: 429.18 found for $[M+H]^+$, 429.26 calcd. for $C_{27}H_{33}N_4O^+$, R_t = 0.84 min, purity (UV area): ≥99%.
HRMS (ESI+) *m/z*: 429.2649 found for $[M+H]^+$, 429.2649 calcd. for $C_{27}H_{33}N_4O^+$ (Δ = 0.00 ppm).

Note: The peak for the C14-H proton at 7.24 ppm in the 1D ^{1}H NMR spectrum overlaps with the chloroform-*d* solvent peak, thus increasing the integral, but the chemical shift and integral were confirmed by correlations in the 2D ^{1}H-^{1}H COESY and ^{1}H-^{13}C HSQC and HMBC spectra.

Synthesis of enantioenriched compounds

Synthesis of organocatalysts

(*S*)-*N*-((*S*)-2'-((4-Methylphenyl)sulfonamido)-[1,1'-binaphthalen]-2-yl)pyrrolidine-2-carboxamide ((–)-S13)

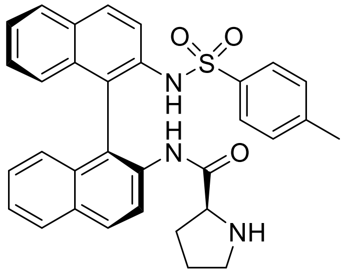

(–)-**S13**

Following procedure of Viózquez *et al.*[18] using (*S*)-(−)-1,1′-binaphthyl-2,2′-diamine (1.00 g, 3.52 mmol) and Boc-L-Proline (1.14 g, 5.30 mmol) stirring for 18 hours (step 1), refluxing for 15.5 hours (step 2), and stirring for 1.5 hours (step 3) afforded compound (–)-**S13** (0.906 g, 50% yield over three steps) as a white solid with an *er* = 99.6:0.4 (>99% *ee*).

^{1}H NMR (400 MHz, $CDCl_3$) δ ppm: 9.25 (br s, 1H), 8.78 (d, *J* = 9.0 Hz, 1H), 8.15 (d, *J* = 9.1 Hz, 1H), 8.06 (d, *J* = 9.0 Hz, 1H), 7.99 (d, *J* = 9.1 Hz, 1H), 7.94 (d, *J* = 8.2 Hz, 1H), 7.87 (d, *J* = 8.2 Hz, 1H), 7.46 – 7.34 (m, 4H), 7.23 – 7.14 (m, 2H), 7.11 (d, *J* = 8.0 Hz, 2H), 6.93 (d, *J* = 8.5 Hz, 1H), 6.85 (d, *J* = 8.4 Hz, 1H), 6.33 (br s, 1H), 3.34 (dd, *J* = 9.5, 4.1 Hz, 1H), 2.36 (s, 3H), 2.26 (q, *J* = 8.1 Hz, 1H), 1.85 – 1.73 (m, 1H), 1.66 – 1.54 (m, 1H), 1.37 – 1.14 (m, 2H), 0.78 – 0.64 (m, 1H).
^{13}C NMR (101 MHz, $CDCl_3$) δ ppm: 173.5, 144.0, 136.6, 135.7, 133.8, 132.6, 132.3, 131.3, 130.9, 130.6, 130.3, 129.6, 128.7, 128.3, 127.7, 127.6, 125.8, 125.3, 125.3, 124.3, 120.9, 119.5, 119.5, 117.0, 60.5, 46.2, 30.7, 25.3, 21.7.
NMR data are in accordance with literature values.[18]
Optical rotation: $[\alpha]_D^{24}$ = -90.8 (*c* = 0.53, $CHCl_3$).
Specific rotation in accordance with literature value ($[\alpha]_D^{25}$ = -95 (*c* = 1.0, $CHCl_3$)).[18]

(*R*)-*N*-((*R*)-2'-((4-Methylphenyl)sulfonamido)-[1,1'-binaphthalen]-2-yl)pyrrolidine-2-carboxamide ((+)-S13)

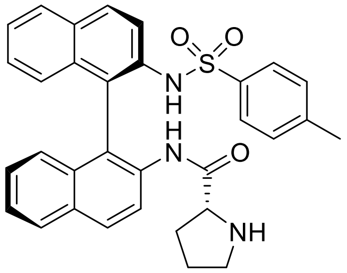

(+)-**S13**

Following procedure of Viózquez *et al.*[18] using (*R*)-(+)-1,1′-binaphthyl-2,2′-diamine (1.56 g, 5.50 mmol) and Boc-D-Proline (1.88 g, 8.74 mmol) stirring for 15 hours (step 1), refluxing for 13 hours (step 2), and stirring for two hours (step 3) afforded compound (+)-**S13** (1.60 g, 56% yield over three steps) as a white solid with an *er* = 100.0:0.0 (>99% *ee*).

^{1}H NMR (400 MHz, $CDCl_3$) δ ppm: 9.27 (br s, 1H), 8.80 (d, *J* = 9.0 Hz, 1H), 8.17 (d, *J* = 9.1 Hz, 1H), 8.06 (d, *J* = 9.0 Hz, 1H), 7.99 (d, *J* = 9.1 Hz, 1H), 7.94 (d, *J* = 8.1 Hz, 1H), 7.87 (d, *J* = 8.2 Hz, 1H), 7.46 – 7.34 (m, 4H), 7.18 (dddd, *J* = 9.8, 8.2, 6.8, 1.3 Hz, 2H), 7.11 (d, *J* = 8.1 Hz, 2H), 6.93 (d, *J* = 8.6 Hz, 1H), 6.85 (d, *J* = 8.4 Hz, 1H), 6.33 (br s, 1H), 3.32 (dd, *J* = 9.5, 4.1 Hz, 1H), 2.36 (s, 3H), 2.24 (ddd, *J* = 9.6, 8.1, 6.4 Hz, 1H), 1.84 – 1.72 (m, 1H), 1.64 – 1.55 (m, 1H), 1.31 – 1.13 (m, 2H), 0.74 – 0.62 (m, 1H).

^{13}C NMR (101 MHz, $CDCl_3$) δ ppm: 173.4, 144.0, 136.6, 135.7, 133.8, 132.6, 132.2, 131.3, 130.9, 130.6, 130.2, 129.6, 128.7, 128.3, 127.7, 127.6, 125.7, 125.3, 125.2, 124.3, 120.8, 119.5, 119.4, 117.1, 60.5, 46.2, 30.7, 25.3, 21.7.
NMR data are in accordance with literature values.[18]
Optical rotation: $[\alpha]_D^{24}$ = +86.7 (*c* = 0.53, $CHCl_3$).
Specific rotation in accordance with literature value ($[\alpha]_D^{25}$ = +93 (*c* = 1.0, $CHCl_3$), >99% *ee*).[18]

(*S*)-8a-Methyl-3,4,8,8a-tetrahydronaphthalene-1,6(2*H*,7*H*)-dione ((+)-S2)

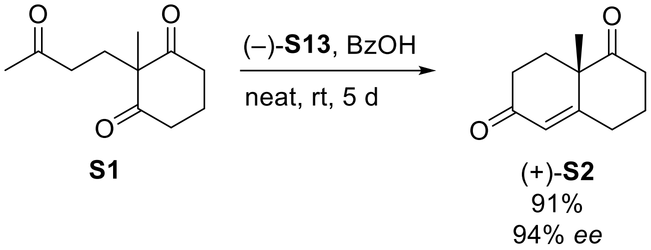

A mixture of compound **S1** (15.4 g, 78.5 mmol, 1.00 equiv.), compound (−)-**S13** (420 mg, 0.785 mmol, 0.01 equiv. = 1 mol%), and benzoic acid (240 mg, 1.97 mmol, 0.025 equiv. = 2.5 mol%) was stirred neat at room temperature for 5 days. The reaction mixture was added ethyl acetate (100 mL) and activated carbon (3.0 g). The mixture was stirred at room temperature for 18 hours, added *n*-hexane (100 mL), filtered by suction through a plug of silica gel eluting with 1:1 *n*-hexane/ethyl acetate, and the solvent removed under reduced pressure to yield a crude dark-amber oil. The crude product was purified by flash column chromatography on silica gel (9:1 to 3:2 toluene/ethyl acetate) to afford compound (+)-**S2** (12.78 g, 91% yield) as a light-red oil that solidified to an off-white solid upon storage in the freezer (-20 °C) with an *er* = 97.0:3.0 (94% *ee*).

TLC: R_f = 0.29 (3:1 toluene/ethyl acetate), UV.
^{1}H NMR (400 MHz, $CDCl_3$) δ ppm: 5.85 (d, *J* = 1.9 Hz, 1H), 2.77 – 2.66 (m, 2H), 2.54 – 2.42 (m, 4H), 2.19 – 2.08 (m, 3H), 1.71 (qt, *J* = 13.3, 4.4 Hz, 1H), 1.45 (s, 3H).
^{13}C NMR (101 MHz, $CDCl_3$) δ ppm: 211.2, 198.5, 165.9, 126.0, 50.7, 37.8, 33.8, 31.9, 29.8, 23.4, 23.1.
NMR data are in accordance with literature values.[7]
Optical rotation: $[\alpha]_D^{22}$ = +102.7 (*c* = 1.05, $CHCl_3$).
Specific rotation in accordance with literature value ($[\alpha]_D^{22}$ = +107.5 (*c* = 2.0, $CHCl_3$), 92% *ee*).[19]

(*R*)-8a-Methyl-3,4,8,8a-tetrahydronaphthalene-1,6(2*H*,7*H*)-dione ((–)-S2)

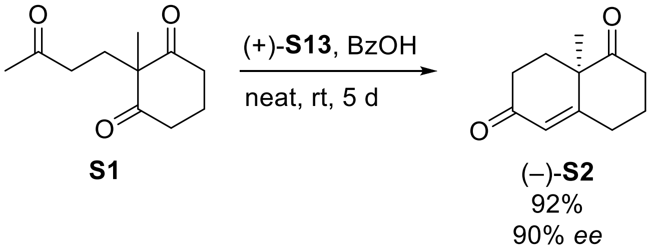

A mixture of compound **S1** (19.2 g, 97.7 mmol, 1.00 equiv.), compound (+)-**S13** (0.525 g, 0.979 mmol, 0.01 equiv. = 1 mol%), and benzoic acid (301 mg, 2.47 mmol, 0.025 equiv. = 2.5 mol%) was stirred neat at room temperature for 5 days. The reaction mixture was added ethyl acetate (128 mL) and activated carbon (3.8 g). The mixture was stirred at room temperature for 24 hours, added *n*-hexane (128 mL), filtered by suction through a plug of silica gel eluting with 1:1 *n*-hexane/ethyl acetate, and the solvent removed under reduced pressure to yield a crude dark-amber oil. The crude product was purified by flash column chromatography on silica gel (9:1 to 3:2 toluene/ethyl acetate) to afford compound (–)-**S2** (16.0 g, 92% yield) as a purple oil that solidified to a brown solid upon storage in the freezer (-20 °C) with an *er* = 95.2:4.8 (90% *ee*).

^{1}H NMR (400 MHz, $CDCl_3$) δ ppm: 5.86 (d, *J* = 1.9 Hz, 1H), 2.78 – 2.66 (m, 2H), 2.55 – 2.42 (m, 4H), 2.19 – 2.08 (m, 3H), 1.71 (qt, *J* = 13.3, 4.4 Hz, 1H), 1.45 (s, 3H).
^{13}C NMR (101 MHz, $CDCl_3$) δ ppm: 211.2, 198.5, 165.9, 126.0, 50.7, 37.8, 33.8, 31.9, 29.8, 23.4, 23.1.
NMR data are in accordance with literature values.[7]
Optical rotation: $[\alpha]_D^{22}$ = -97.9 (*c* = 1.00, $CHCl_3$).

Specific rotation in accordance with literature value ([α]$_D^{25}$ = -104 (*c* = 1.0, $CHCl_3$), 82% *ee*).[20]

(*S*)-8a-Methyl-3,4,8,8a-tetrahydro-2*H*-spiro[naphthalene-1,2'-[1,3]dioxolan]-6(7*H*)-one ((+)-S3)

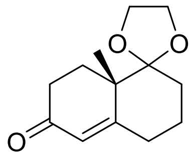

(+)-**S3**

Following procedure of compound (±)-**S3** using compound (+)-**S2** (12.6 g, 70.7 mmol) stirring for three hours afforded compound (+)-**S3** (12.7 g, 81% yield) as a white solid with an *er* = 95.2:4.8 (90% *ee*).

^{1}H NMR (400 MHz, $CDCl_3$) δ ppm: 5.81 (d, *J* = 2.0 Hz, 1H), 4.01 – 3.90 (m, 4H), 2.48 – 2.22 (m, 5H), 1.94 – 1.84 (m, 1H), 1.82 – 1.61 (m, 4H), 1.35 (s, 3H).
^{13}C NMR (101 MHz, $CDCl_3$) δ ppm: 199.4, 167.8, 125.8, 112.5, 65.5, 65.2, 45.2, 34.1, 31.6, 30.2, 27.0, 21.9, 20.6.
NMR data are in accordance with racemic compound and literature values.[10]
Optical rotation: [α]$_D^{22}$ = +110.5 (*c* = 0.55, $CHCl_3$).
Specific rotation in accordance with literature value ([α]$_D^{20}$ = +124.2 (*c* = 0.153, $CHCl_3$)).[21]

(*R*)-8a-Methyl-3,4,8,8a-tetrahydro-2*H*-spiro[naphthalene-1,2'-[1,3]dioxolan]-6(7*H*)-one ((–)-S3)

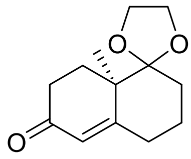

(–)-**S3**

Following procedure of compound (±)-**S3** using compound (–)-**S2** (13.0 g, 72.9 mmol) afforded compound (–)-**S3** (11.3 g, 70% yield) as an off-white solid with an *er* = 94.7:5.3 (89% *ee*).

^{1}H NMR (400 MHz, $CDCl_3$) δ ppm: 5.81 (d, *J* = 2.0 Hz, 1H), 4.01 – 3.91 (m, 4H), 2.49 – 2.23 (m, 5H), 1.94 – 1.84 (m, 1H), 1.83 – 1.64 (m, 4H), 1.35 (s, 3H).
^{13}C NMR (101 MHz, $CDCl_3$) δ ppm: 199.4, 167.8, 125.8, 112.5, 65.5, 65.2, 45.2, 34.1, 31.6, 30.2, 27.0, 21.9, 20.6.
NMR data are in accordance with racemic compound and literature values.[10]
Optical rotation: [α]$_D^{22}$ = -101.7 (*c* = 0.54, $CHCl_3$).
Specific rotation in accordance with literature value ([α]$_D^{20}$ = -102.6 (*c* = 1.06, $CHCl_3$)).[22]

(4a*R*,8a*S*)-8a-Methylhexahydro-2*H*-spiro[naphthalene-1,2'-[1,3]dioxolan]-6(5*H*)-one ((+)-S4)

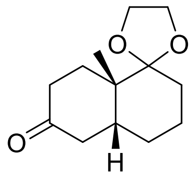

(+)-**S4**

Following procedure of compound (±)-**S4** using compound (+)-**S3** (12.7 g, 57.2 mmol) afforded compound (+)-**S4** (4.30 g, 34% yield) as a white solid.

Note: A significant amount of the Leuckart–Wallach amine side-product was formed, thus lowering the yield.

^{1}H NMR (400 MHz, $CDCl_3$) δ ppm: 4.01 – 3.93 (m, 4H), 2.62 (dd, *J* = 14.6, 5.5 Hz, 1H), 2.48 – 2.37 (m, 1H), 2.36 – 2.28 (m, 1H), 2.21 – 2.04 (m, 3H), 1.76 (dddd, *J* = 13.8, 7.0, 3.1, 1.6 Hz, 1H), 1.69 – 1.44 (m, 5H), 1.31 – 1.17 (m, 4H).
^{13}C NMR (101 MHz, $CDCl_3$) δ ppm: 212.5, 112.6, 65.2, 65.1, 44.3, 42.8, 41.4, 38.0, 29.8, 29.2, 28.4, 22.4, 17.9.

NMR data are in accordance with racemic compound and literature values.[12]
Optical rotation: $[\alpha]_D^{22}$ = +37.7 (c = 0.51, $CHCl_3$).
Specific rotation in accordance with literature value ($[\alpha]_D^{20}$ = +14.0 (c = 0.2, $CHCl_3$)).[13]

(4a*S*,8a*R*)-8a-Methylhexahydro-2*H*-spiro[naphthalene-1,2'-[1,3]dioxolan]-6(5*H*)-one ((–)-S4)

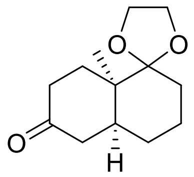

(–)-**S4**

Following procedure of compound (±)-**S4** using compound (–)-**S3** (11.3 g, 50.9 mmol) stirring for 1.5 hours afforded compound (–)-**S4** (6.12 g, 54% yield) as a white solid.

Note: A fair amount of the Leuckart–Wallach amine side-product was formed, thus lowering the yield.

^{1}H NMR (400 MHz, $CDCl_3$) δ ppm: 3.99 – 3.94 (m, 4H), 2.63 (dd, J = 14.6, 5.6 Hz, 1H), 2.48 – 2.38 (m, 1H), 2.36 – 2.28 (m, 1H), 2.21 – 2.05 (m, 3H), 1.76 (dddd, J = 13.8, 7.0, 3.0, 1.7 Hz, 1H), 1.69 – 1.45 (m, 5H), 1.32 – 1.19 (m, 4H).
^{13}C NMR (101 MHz, $CDCl_3$) δ ppm: 212.6, 112.6, 65.2, 65.1, 44.3, 42.8, 41.4, 38.0, 29.8, 29.2, 28.4, 22.4, 17.8.
NMR data are in accordance with racemic compound and literature values.[12]
Optical rotation: $[\alpha]_D^{22}$ = -35.6 (c = 0.58, $CHCl_3$).
Specific rotation in accordance with literature value ($[\alpha]_D^{20}$ = +14.0 (c = 0.2, $CHCl_3$) for the enantiomer).[13]

(4a*R*,6*R*,8a*S*)-6-Hydroxy-8a-methyloctahydronaphthalen-1(2*H*)-one ((–)-1)

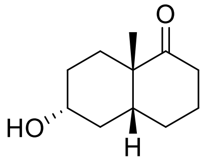

(–)-**1**

Following procedures of compound (±)-**S5** and (±)-**1** using compound (+)-**S4** (4.10 g, 18.3 mmol) afforded compound (–)-**1** (2.01 g, 60% yield over two steps) as a white solid.

^{1}H NMR (400 MHz, $CDCl_3$) δ ppm: 3.60 (tt, J = 11.2, 4.3 Hz, 1H), 2.61 – 2.50 (m, 1H), 2.33 – 2.16 (m, 3H), 2.01 – 1.85 (m, 3H), 1.84 – 1.76 (m, 1H), 1.69 (dtd, J = 12.6, 3.8, 2.5 Hz, 1H), 1.53 – 1.46 (m, 1H), 1.43 – 1.19 (m, 5H), 0.94 (td, J = 13.7, 4.0 Hz, 1H).
^{13}C NMR (101 MHz, $CDCl_3$) δ ppm: 215.1, 71.0, 48.6, 43.7, 38.5, 38.0, 33.1, 32.9, 26.7, 26.4, 22.2.
NMR data are in accordance with racemic compound and literature values.[15]
Optical rotation: $[\alpha]_D^{22}$ = -55.8 (c = 0.50, $CHCl_3$).
Specific rotation in accordance with literature value ($[\alpha]_D^{25}$ = +47.5 (c = 2.0, $CHCl_3$), ≥99% *ee* for the enantiomer).[15]

(4a*S*,6*S*,8a*R*)-6-Hydroxy-8a-methyloctahydronaphthalen-1(2*H*)-one ((+)-1)

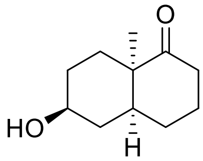

(+)-**1**

Following procedures of compound (±)-**S5** and (±)-**1** using compound (–)-**S4** (5.89 g, 26.3 mmol) afforded compound (+)-**1** (3.69 g, 77% yield over two steps) as a white solid.

^{1}H NMR (400 MHz, $CDCl_3$) δ ppm: 3.67 – 3.55 (m, 1H), 2.62 – 2.48 (m, 1H), 2.33 – 2.16 (m, 3H), 2.01 – 1.85 (m, 3H), 1.84 – 1.77 (m, 1H), 1.70 (dtd, *J* = 12.7, 3.8, 2.5 Hz, 1H), 1.53 – 1.46 (m, 1H), 1.44 – 1.18 (m, 5H), 0.94 (td, *J* = 13.7, 4.0 Hz, 1H).
^{13}C NMR (101 MHz, $CDCl_3$) δ ppm: 215.1, 71.1, 48.7, 43.7, 38.5, 38.0, 33.1, 32.9, 26.7, 26.4, 22.2.
NMR data are in accordance with racemic compound and literature values.[15]
Optical rotation: $[\alpha]_D^{22}$ = +54.5 (*c* = 0.53, $CHCl_3$).
Specific rotation in accordance with literature value ($[\alpha]_D^{25}$ = +47.5 (*c* = 2.0, $CHCl_3$), ≥99% *ee*).[15]

(3*R*,4a*R*,11b*S*)-8-Bromo-11b-methyl-2,3,4,4a,5,6,11,11b-octahydro-1*H*-benzo[*a*]carbazol-3-ol ((+)-10b)

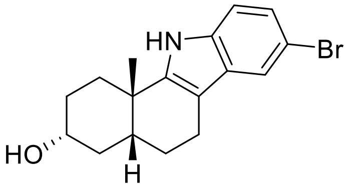

(+)-**10b**

Following procedure of compound (±)-**10b** using compound (–)-**1** (1.67 g, 9.16 mmol) and purifying using FlashPure Select Silica (25 g) eluting with 1:0 to 0:1 dichloromethane/ethyl acetate (flow rate = 25 mL/min) afforded compound (+)-**10b** (2.98 g, 97% yield) as a light-pink solid with an *er* = 94.6:5.4 (89% *ee*).

^{1}H NMR (400 MHz, $CDCl_3$) δ ppm: 7.74 (br s, 1H), 7.56 (d, *J* = 1.7 Hz, 1H), 7.23 – 7.14 (m, 2H), 3.73 – 3.61 (m, 1H), 2.73 – 2.62 (m, 2H), 2.24 – 2.08 (m, 2H), 1.86 – 1.68 (m, 4H), 1.58 (td, *J* = 14.1, 3.3 Hz, 1H), 1.39 (q, *J* = 11.8 Hz, 1H), 1.29 (s, 3H), 1.08 – 0.96 (m, 1H).
^{13}C NMR (101 MHz, $CDCl_3$) δ ppm: 140.1, 134.5, 130.0, 123.9, 120.8, 112.6, 112.0, 108.4, 70.6, 40.5, 36.6, 35.7, 34.9, 32.6, 30.8, 24.4, 17.3.
NMR data are in accordance with racemic compound.
HRMS (ESI+) *m/z*: 334.0791 found for $[M+H]^+$, 334.0801 calcd. for $C_{17}H_{21}BrNO^+$ (Δ = -2.92 ppm).
Optical rotation: $[\alpha]_D^{21}$ = +29.7 (*c* = 0.51, $CHCl_3$).

(3*S*,4a*S*,11b*R*)-8-Bromo-11b-methyl-2,3,4,4a,5,6,11,11b-octahydro-1*H*-benzo[*a*]carbazol-3-ol ((–)-10b)

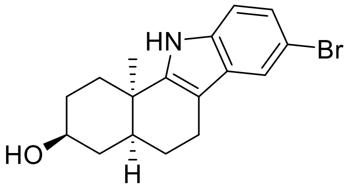

(–)-**10b**

Following procedure of compound (±)-**10b** using compound (+)-**1** (2.13 g, 11.7 mmol) and purifying using FlashPure Select Silica (25 g) eluting with 1:0 to 0:1 dichloromethane/ethyl acetate (flow rate = 25 mL/min) afforded compound (–)-**10b** (3.33 g, 85% yield) as a pale-yellow solid with an *er* = 97.0:3.0 (94% *ee*).

^{1}H NMR (400 MHz, $CDCl_3$) δ ppm: 7.74 (br s, 1H), 7.56 (d, *J* = 1.7 Hz, 1H), 7.22 – 7.14 (m, 2H), 3.74 – 3.62 (m, 1H), 2.73 – 2.61 (m, 2H), 2.23 – 2.08 (m, 2H), 1.87 – 1.67 (m, 4H), 1.62 – 1.53 (m, 1H), 1.39 (q, *J* = 12.1, 11.7 Hz, 1H), 1.29 (s, 3H), 1.09 – 0.96 (m, 1H).
^{13}C NMR (101 MHz, $CDCl_3$) δ ppm: 140.1, 134.5, 130.0, 123.9, 120.9, 112.6, 112.0, 108.4, 70.7, 40.5, 36.7, 35.7, 34.9, 32.7, 30.8, 24.4, 17.3.
NMR data are in accordance with racemic compound.
HRMS (ESI+) *m/z*: 334.0790 found for $[M+H]^+$, 334.0801 calcd. for $C_{17}H_{21}BrNO^+$ (Δ = -3.22 ppm)
Optical rotation: $[\alpha]_D^{21}$ = -34.6 (*c* = 0.51, $CHCl_3$).

(3*R*,3a*S*,6*R*,12b*R*)-11-Bromo-3a-methyl-2,3,3a,4,5,6-hexahydro-1*H*-3,6-methanocyclopenta[3,4]oxepino[2,3-*b*]indole ((+)-15b)

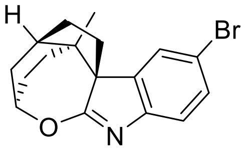

(+)-**15b**

Following procedure of compound (±)-**15b** using compound (+)-**10b** (2.45 g, 7.34 mmol) and purifying using FlashPure Select Silica (25 g) eluting with 1:0 to 0:1 *n*-heptane/ethyl acetate (flow rate = 25 mL/min) afforded compound (+)-**15b** (0.692 g, 28% yield) as a yellow solid with an *er* = 96.0:4.0 (92% *ee*).

TLC: R_f = 0.18 (2:1 *n*-heptane/ethyl acetate), UV.
^{1}H NMR (400 MHz, CD_3OD) δ ppm: 7.52 (d, *J* = 1.9 Hz, 1H), 7.41 (dd, *J* = 8.2, 2.0 Hz, 1H), 7.14 (d, *J* = 8.3 Hz, 1H), 4.69 (quint, *J* = 2.8 Hz, 1H), 2.59 – 2.48 (m, 1H), 2.34 – 2.20 (m, 3H), 2.17 – 2.07 (m, 1H), 2.07 – 1.94 (m, 2H), 1.83 – 1.72 (m, 1H), 1.70 – 1.61 (m, 1H), 1.60 – 1.50 (m, 1H), 1.35 – 1.21 (m, 4H).
^{13}C NMR (101 MHz, CD_3OD) δ ppm: 188.2, 152.8, 142.6, 132.0, 128.6, 120.7, 118.4, 77.0, 67.3, 45.8, 43.7, 36.72, 36.71, 33.0, 27.9, 27.3, 25.9.
^{1}H NMR (400 MHz, $CDCl_3$) δ ppm: 7.40 – 7.34 (m, 2H), 7.22 (d, *J* = 8.7 Hz, 1H), 4.69 – 4.65 (m, 1H), 2.51 – 2.41 (m, 1H), 2.28 – 2.08 (m, 5H), 1.99 (dq, *J* = 15.1, 3.0 Hz, 1H), 1.71 – 1.61 (m, 2H), 1.50 – 1.40 (m, 1H), 1.38 – 1.32 (m, 1H), 1.30 (s, 3H).
^{13}C NMR (101 MHz, $CDCl_3$) δ ppm: 185.6, 153.0, 141.5, 130.8, 127.0, 120.7, 117.1, 74.7, 66.0, 44.7, 42.7, 36.2, 35.6, 32.3, 27.7, 26.5, 25.0.
NMR data are in accordance with racemic compound.
LC-LRMS (ESI+) *m/z*: 332.01 found for $[M+H]^+$, 332.06 calcd. for $C_{17}H_{19}BrNO^+$, R_t = 2.72 min, purity (UV area): 98%.
HRMS (ESI+) *m/z*: 332.0644 found for $[M+H]^+$, 332.0645 calcd. for $C_{17}H_{19}BrNO^+$ (Δ = -0.23 ppm).
Optical rotation: $[\alpha]_D^{21}$ = +74.1 (*c* = 0.52, $CHCl_3$).

(3*S*,3a*R*,6*S*,12b*S*)-11-Bromo-3a-methyl-2,3,3a,4,5,6-hexahydro-1*H*-3,6-methanocyclopenta[3,4]oxepino[2,3-*b*]indole ((–)-15b)

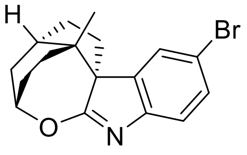

(–)-**15b**

Following procedure of compound (±)-**15b** using compound (–)-**10b** (2.89 g, 8.66 mmol) afforded compound (–)-**15b** (0.702 g, 24% yield) as a cream-white solid with an *er* = 96.2:3.8 (92% *ee*).

^{1}H NMR (400 MHz, CD_3OD) δ ppm: 7.52 (d, *J* = 2.0 Hz, 1H), 7.41 (dd, *J* = 8.2, 2.0 Hz, 1H), 7.14 (d, *J* = 8.3 Hz, 1H), 4.69 (quint, *J* = 2.8 Hz, 1H), 2.59 – 2.47 (m, 1H), 2.34 – 2.20 (m, 3H), 2.16 – 2.07 (m, 1H), 2.07 – 1.94 (m, 2H), 1.83 – 1.73 (m, 1H), 1.69 – 1.61 (m, 1H), 1.60 – 1.50 (m, 1H), 1.34 – 1.21 (m, 4H).
^{13}C NMR (101 MHz, CD_3OD) δ ppm: 188.2, 152.8, 142.6, 132.0, 128.6, 120.7, 118.4, 77.0, 67.3, 45.8, 43.7, 36.72, 36.71, 33.0, 27.9, 27.3, 25.9.
NMR data are in accordance with racemic compound.
LC-LRMS (ESI+) *m/z*: 332.01 found for $[M+H]^+$, 332.06 calcd. for $C_{17}H_{19}BrNO^+$, R_t = 2.72 min, purity (UV area): 99%.
HRMS (ESI+) *m/z*: 332.0644 found for $[M+H]^+$, 332.0645 calcd. for $C_{17}H_{19}BrNO^+$ (Δ = -0.23 ppm).
Optical rotation: $[\alpha]_D^{21}$ = -75.5 (*c* = 0.50, $CHCl_3$).

(3*R*,3a*S*,6*R*,12b*R*)-3a-Methyl-11-morpholino-2,3,3a,4,5,6-hexahydro-1*H*-3,6-methanocyclopenta[3,4]oxepino[2,3-*b*]indole, (+)-asteroxin-1 ((+)-19a)

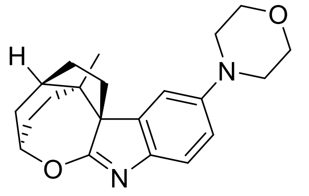

(+)-**19a**

Following procedure of compound (±)-**19a** using compound (+)-**15b** (0.549 g, 1.65 mmol) stirring for 15.5 hours, scavenging for 19 hours, and purifying using FlashPure Select Silica (25 g) eluting with 1:0 to 0:1 *n*-heptane/ethyl acetate (flow rate = 25 mL/min) afforded (+)-asteroxin-1 ((+)-**19a**; 449 mg, 80% yield) as a pale-yellow solid with an *er* = 94.1:5.9 (88% *ee*).

^{1}H NMR (400 MHz, $CDCl_3$) δ ppm: 7.26 (d, *J* = 8.3 Hz, 2H), 6.89 (d, *J* = 2.4 Hz, 1H), 6.81 (dd, *J* = 8.4, 2.4 Hz, 1H), 4.67 – 4.60 (m, 1H), 3.93 – 3.83 (m, 4H), 3.17 – 3.06 (m, 4H), 2.51 – 2.39 (m, 1H), 2.27 – 2.04 (m, 5H), 1.99 (dq, *J* = 14.8, 2.7 Hz, 1H), 1.71 – 1.60 (m, 2H), 1.47 – 1.31 (m, 2H), 1.29 (s, 3H).
^{13}C NMR (101 MHz, $CDCl_3$) δ ppm: 183.9, 148.7, 147.4, 140.3, 119.3, 115.5, 113.7, 74.3, 67.2, 65.8, 51.0, 44.6, 42.7, 36.4, 35.8, 32.4, 27.6, 26.5, 25.0.
NMR data are in accordance with racemic compound.
LC-LRMS (ESI+) *m/z*: 339.17 found for $[M+H]^+$, 339.21 calcd. for $C_{21}H_{27}N_2O_2^+$, R_t = 1.20 min, purity (UV area): 98%.
HRMS (ESI+) *m/z*: 339.2067 found for $[M+H]^+$, 339.2067 calcd. for $C_{21}H_{27}N_2O_2^+$ (Δ = 0.00 ppm).
Optical rotation: $[\alpha]_D^{21}$ = +90.2 (*c* = 0.50, $CHCl_3$).
ICP-MS (Pd-108): 58 mg/kg (ppm).

(3*S*,3a*R*,6*S*,12b*S*)-3a-Methyl-11-morpholino-2,3,3a,4,5,6-hexahydro-1*H*-3,6-methanocyclopenta[3,4]oxepino[2,3-*b*]indole, (–)-asteroxin-1 ((–)-19a)

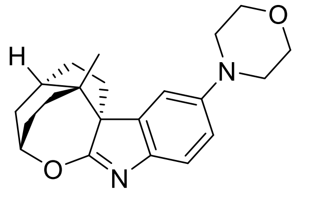

(–)-**19a**

Following procedure of compound (±)-**19a** using compound (–)-**15b** (475 mg, 1.43 mmol) stirring for 14.5 hours, scavenging for 23 hours, and purifying using FlashPure Select Silica (25 g) eluting with 1:0 to 0:1 *n*-heptane/ethyl acetate (flow rate = 25 mL/min) afforded (–)-asteroxin-1 ((–)-**19a**; 387 mg, 80% yield) as a pale-yellow solid with an *er* = 95.1:4.9 (90% *ee*).

^{1}H NMR (400 MHz, $CDCl_3$) δ ppm: 7.26 (d, *J* = 8.4 Hz, 2H), 6.89 (d, *J* = 2.4 Hz, 1H), 6.82 (dd, *J* = 8.3, 2.5 Hz, 1H), 4.67 – 4.60 (m, 1H), 3.94 – 3.82 (m, 4H), 3.18 – 3.04 (m, 4H), 2.52 – 2.38 (m, 1H), 2.29 – 2.03 (m, 5H), 1.99 (dq, *J* = 14.9, 2.7 Hz, 1H), 1.72 – 1.58 (m, 2H), 1.49 – 1.31 (m, 2H), 1.29 (s, 3H).
^{13}C NMR (101 MHz, $CDCl_3$) δ ppm: 183.9, 148.6, 147.4, 140.3, 119.3, 115.5, 113.7, 74.3, 67.2, 65.8, 51.0, 44.6, 42.7, 36.4, 35.8, 32.4, 27.6, 26.5, 25.0.
NMR data are in accordance with racemic compound.
LC-LRMS (ESI+) *m/z*: 339.17 found for $[M+H]^+$, 339.21 calcd. for $C_{21}H_{27}N_2O_2^+$, R_t = 1.19 min, purity (UV area): 97%.
HRMS (ESI+) *m/z*: 339.2067 found for $[M+H]^+$, 339.2067 calcd. for $C_{21}H_{27}N_2O_2^+$ (Δ = 0.00 ppm).
Optical rotation: $[\alpha]_D^{21}$ = -93.6 (*c* = 0.50, $CHCl_3$).
ICP-MS (Pd-108): 41 mg/kg (ppm).

NMR spectra

2-Methyl-2-(3-oxobutyl)cyclohexane-1,3-dione (S1)

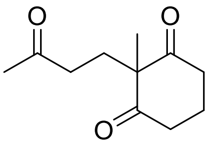

S1

^{1}H NMR (400 MHz, $CDCl_3$)

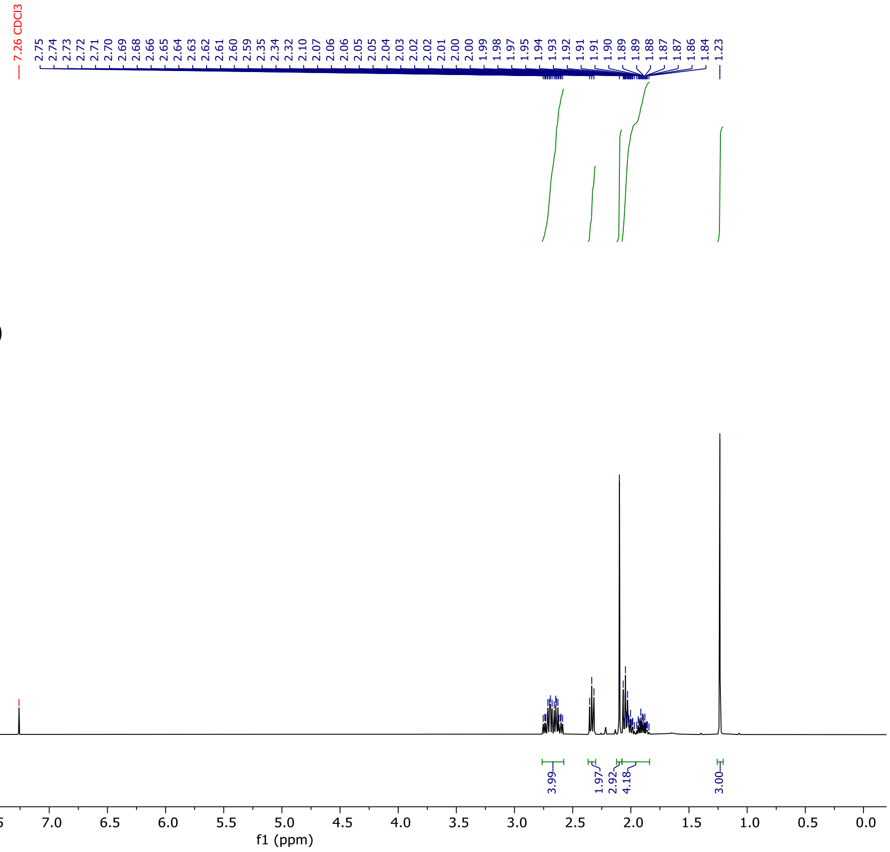

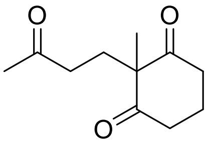

S1

^{13}C NMR (101 MHz, $CDCl_3$)

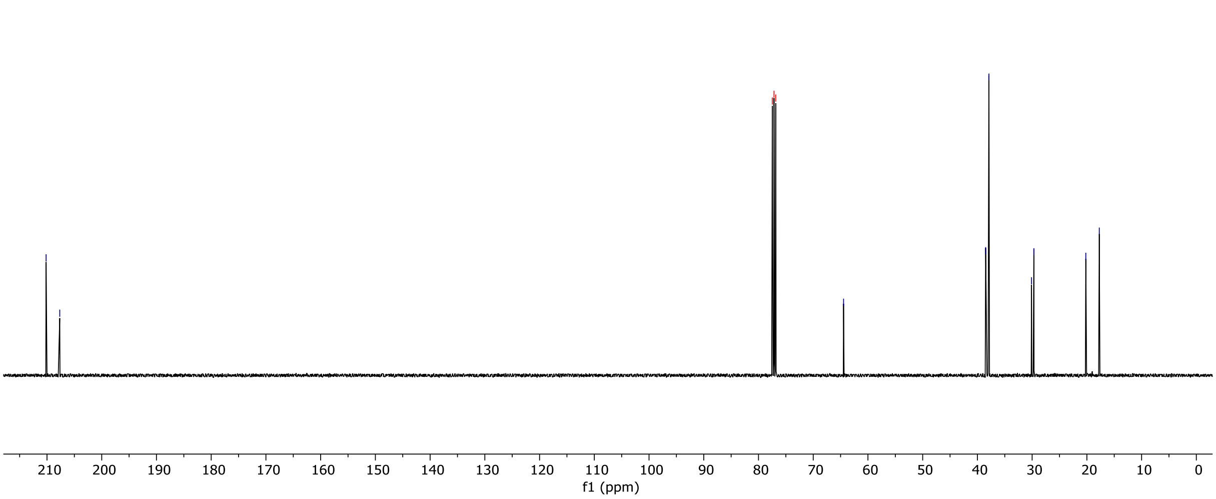

8a-Methyl-3,4,8,8a-tetrahydronaphthalene-1,6(2*H*,7*H*)-dione (S2)

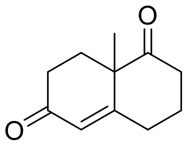

S2

^{1}H NMR (400 MHz, $CDCl_3$)

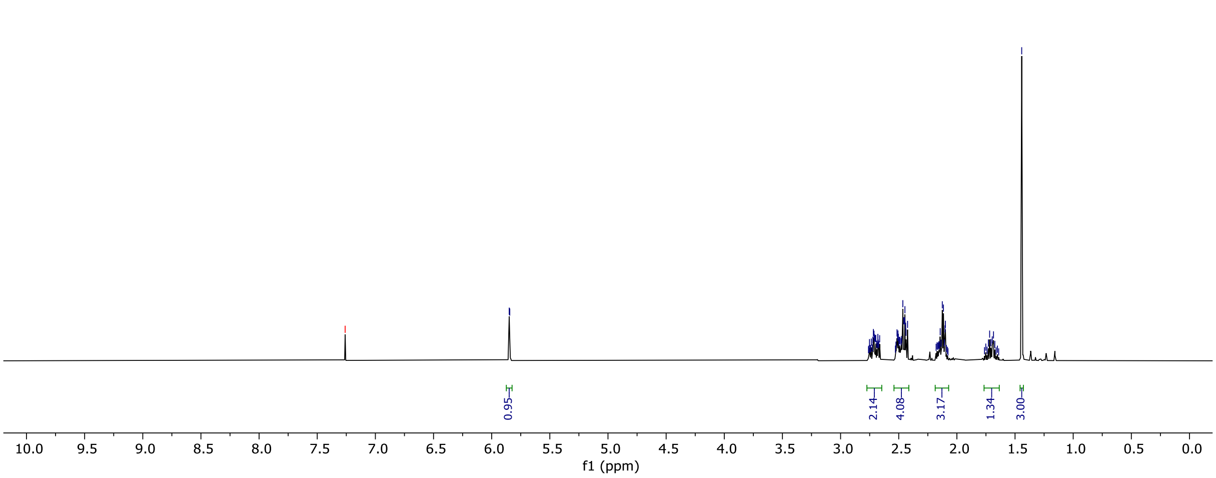

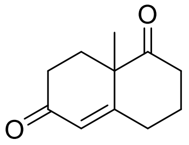

S2

^{13}C NMR (101 MHz, $CDCl_3$)

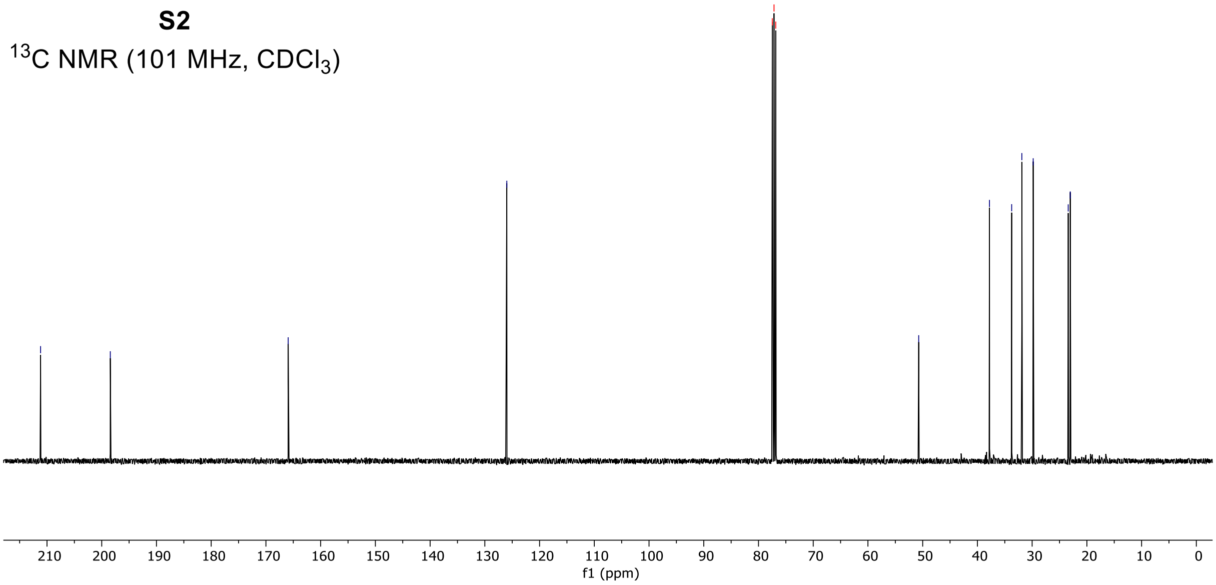

8a-Methyl-3,4,8,8a-tetrahydro-2*H*-spiro[naphthalene-1,2'-[1,3]dioxolan]-6(7*H*)-one (S3)

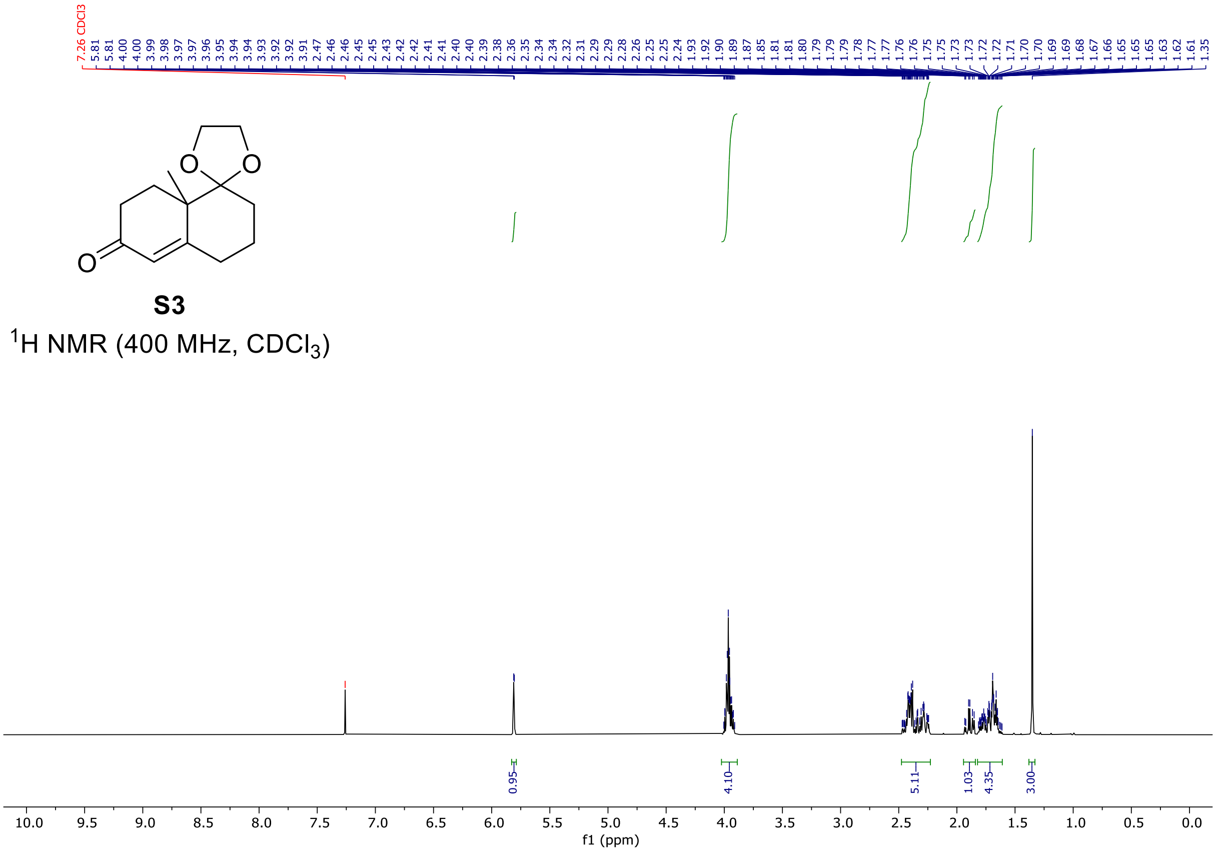

S3

^{1}H NMR (400 MHz, $CDCl_3$)

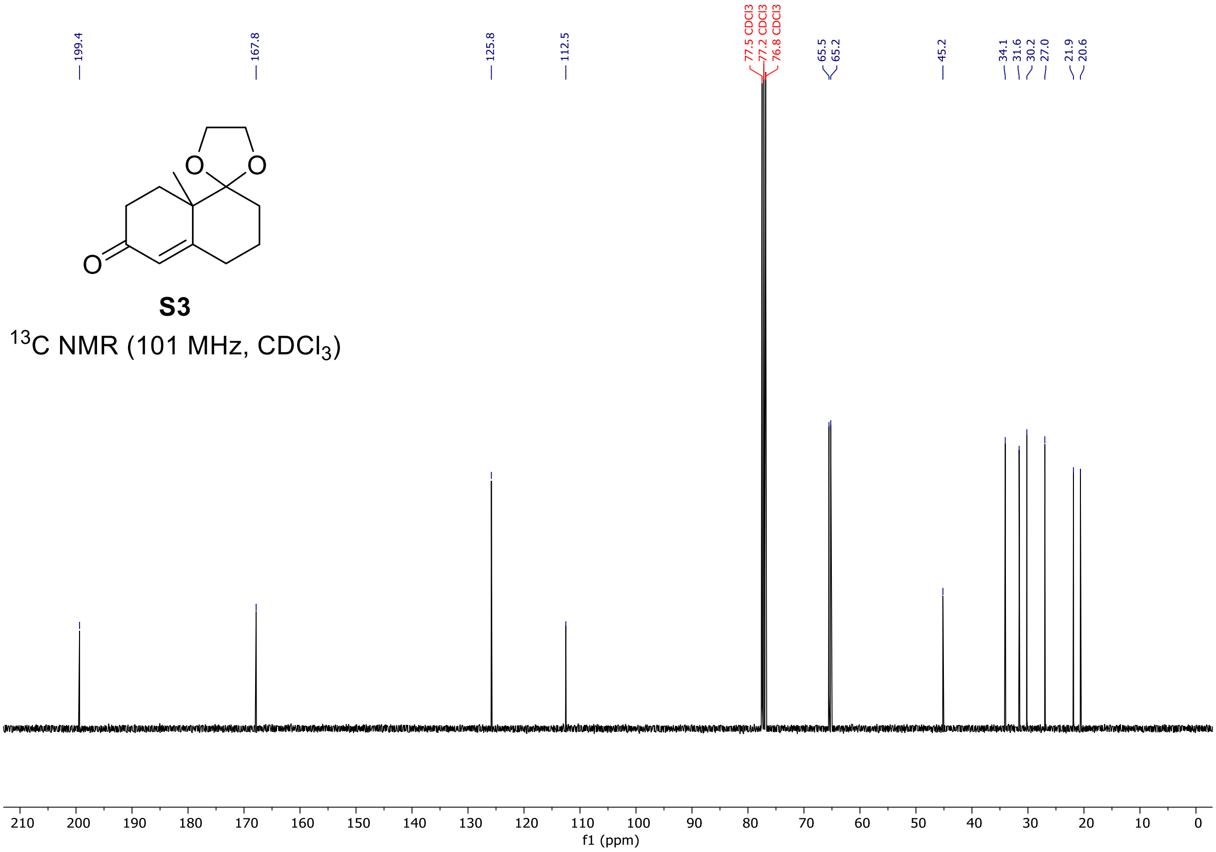

S3

^{13}C NMR (101 MHz, $CDCl_3$)

(4a*R,8a*S**)-8a-Methylhexahydro-2*H*-spiro[naphthalene-1,2'-[1,3]dioxolan]-6(5*H*)-one (S4)**

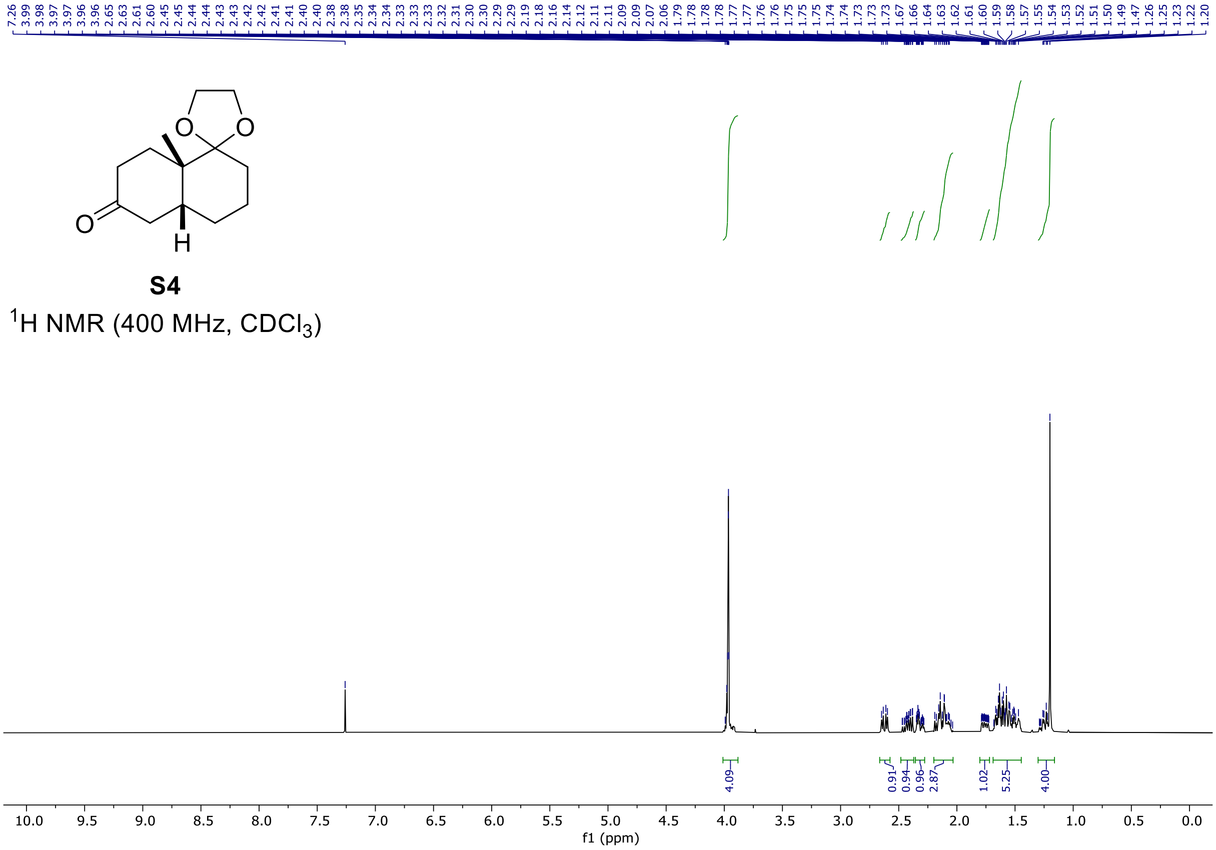

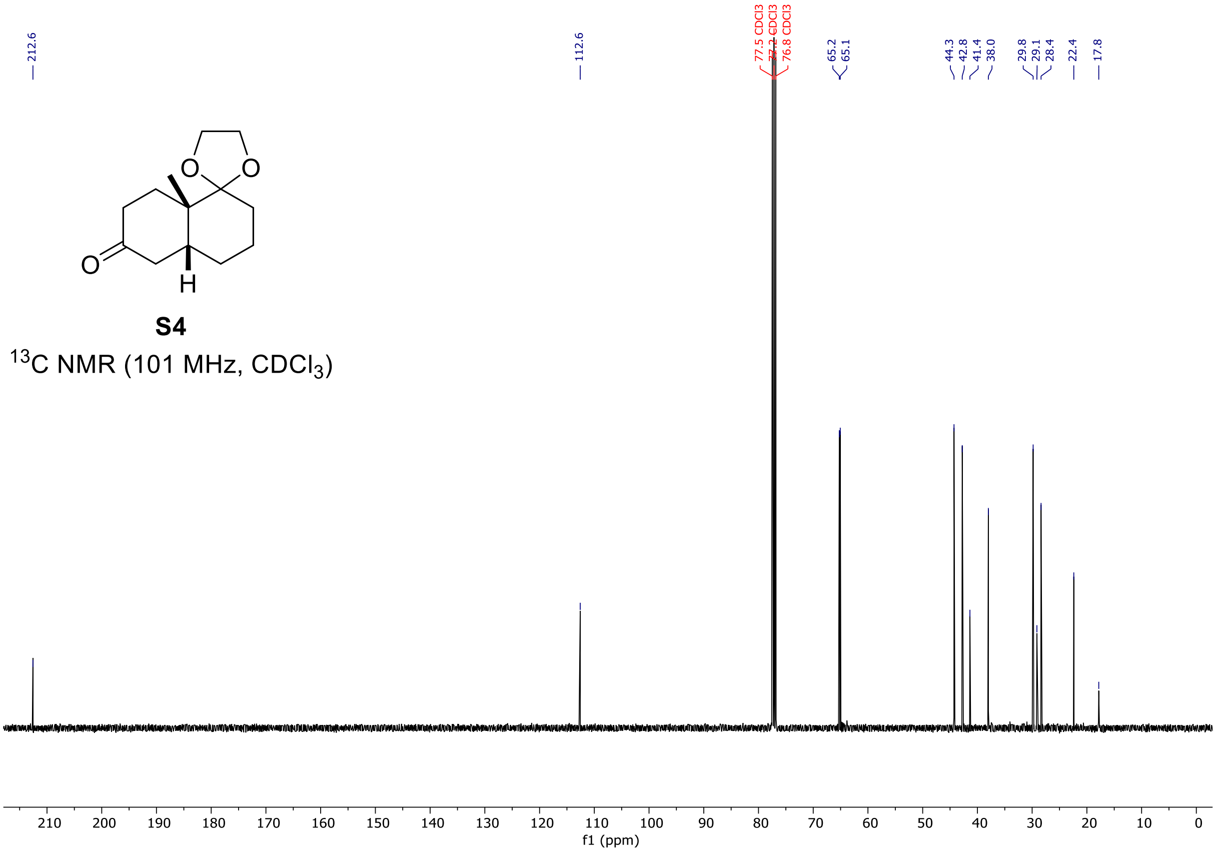

(4a*R**,6*R**,8a*S**)-8a-Methyloctahydro-2*H*-spiro[naphthalene-1,2'-[1,3]dioxolan]-6-ol (S5)

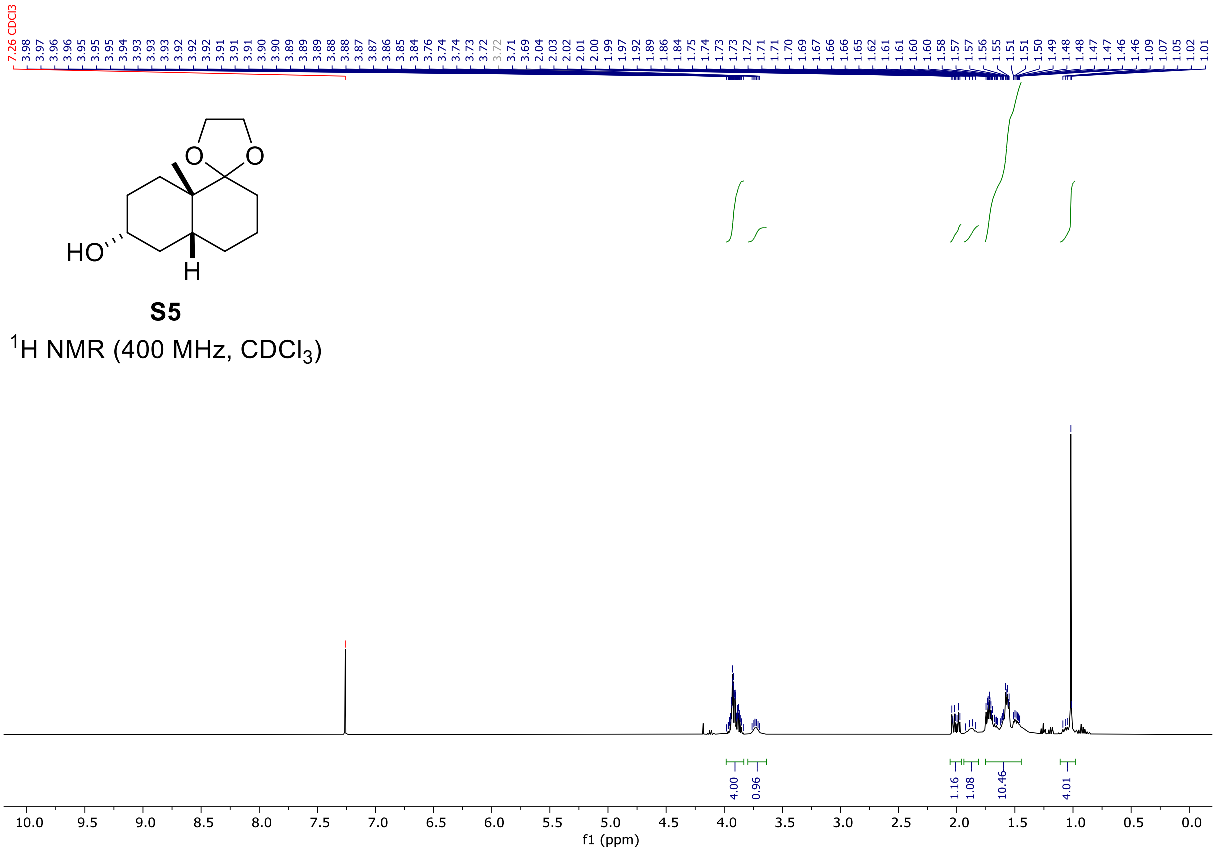

S5

^{1}H NMR (400 MHz, $CDCl_3$)

(4a*R**,6*R**,8a*S**)-8a-Methyloctahydro-2*H*-spiro[naphthalene-1,2'-[1,3]dioxolan]-6-ol (S5) and (4a*R**,6*S**,8a*S**)-8a-methyloctahydro-2*H*-spiro[naphthalene-1,2'-[1,3]dioxolan]-6-ol (S5')

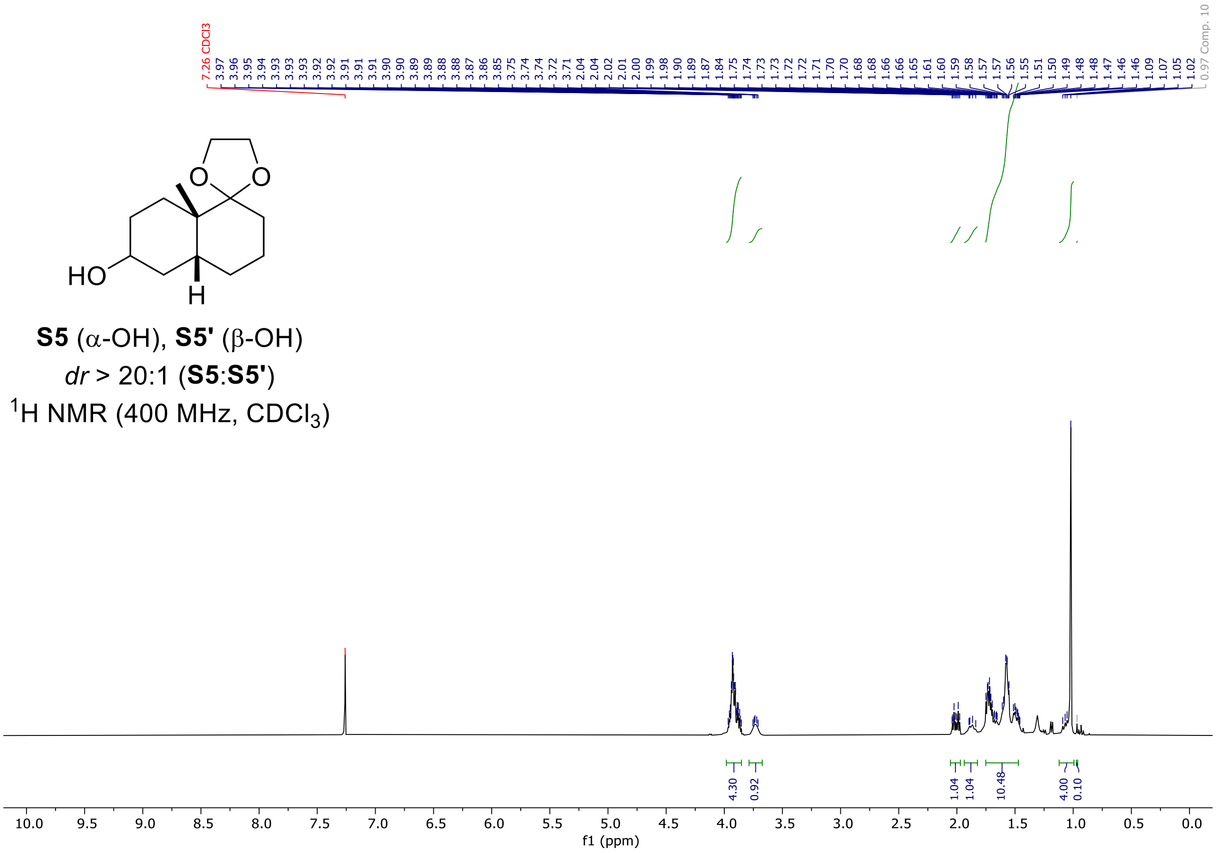

S5 (α-OH), **S5'** (β-OH)

dr > 20:1 (**S5**:**S5'**)

^{1}H NMR (400 MHz, $CDCl_3$)

(4a*R,6*R**,8a*S**)-6-Hydroxy-8a-methyloctahydronaphthalen-1(2*H*)-one (1)**

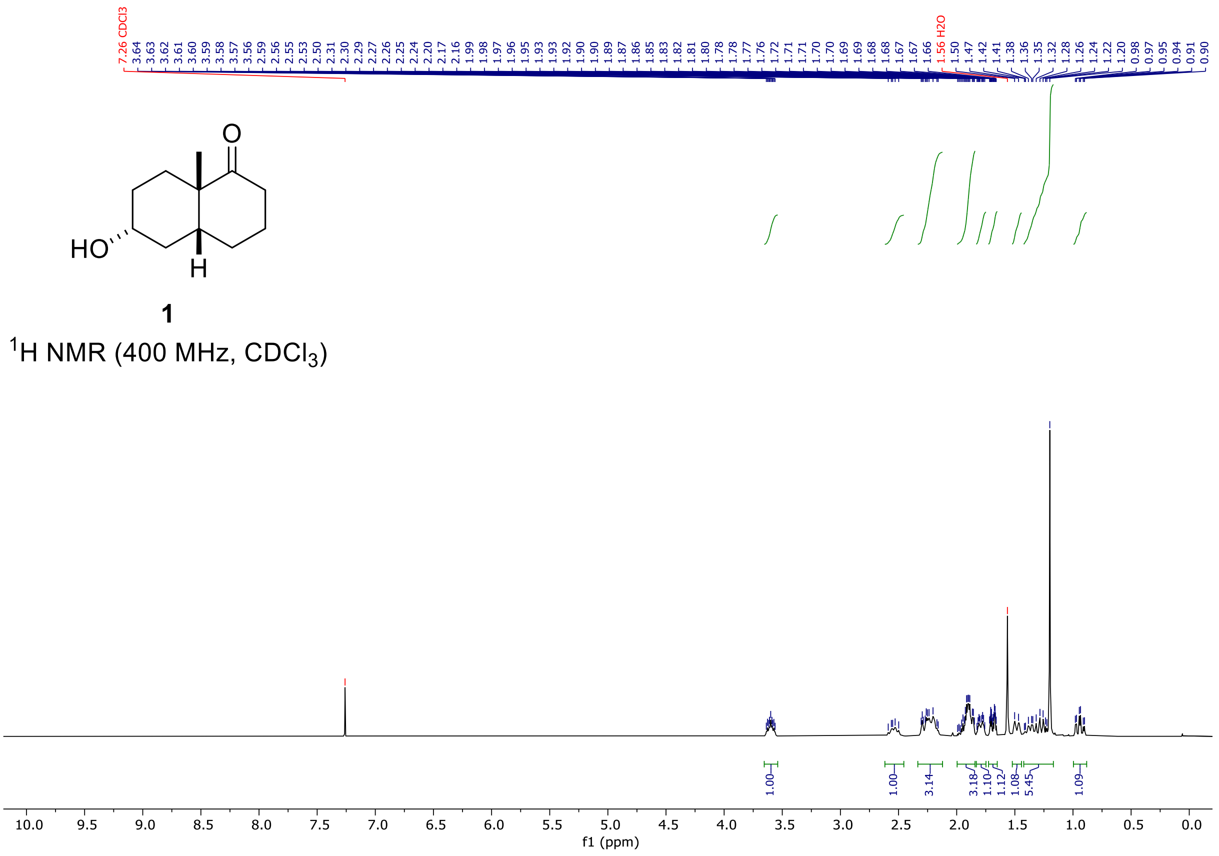

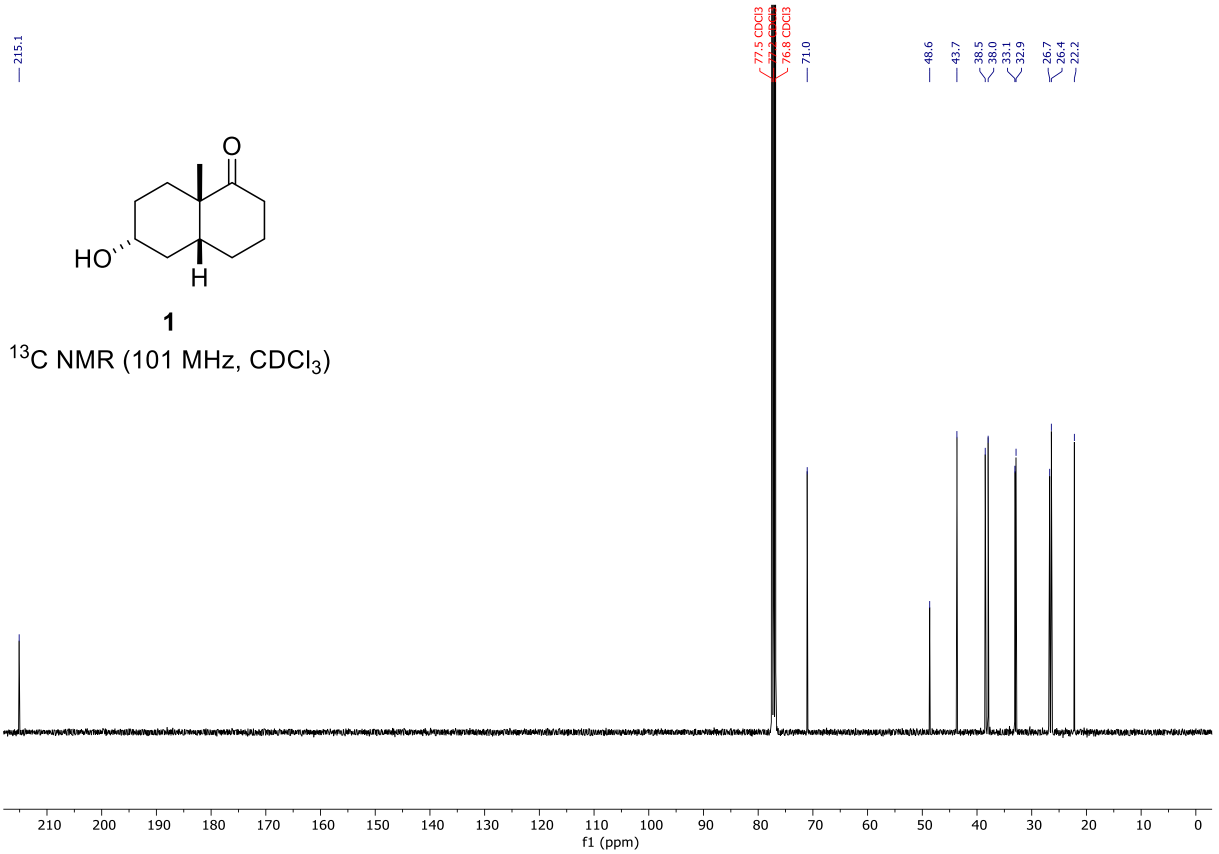

(4a*R,6*S**,8a*S**)-6-hydroxy-8a-methyloctahydronaphthalen-1(2*H*)-one (1’)**

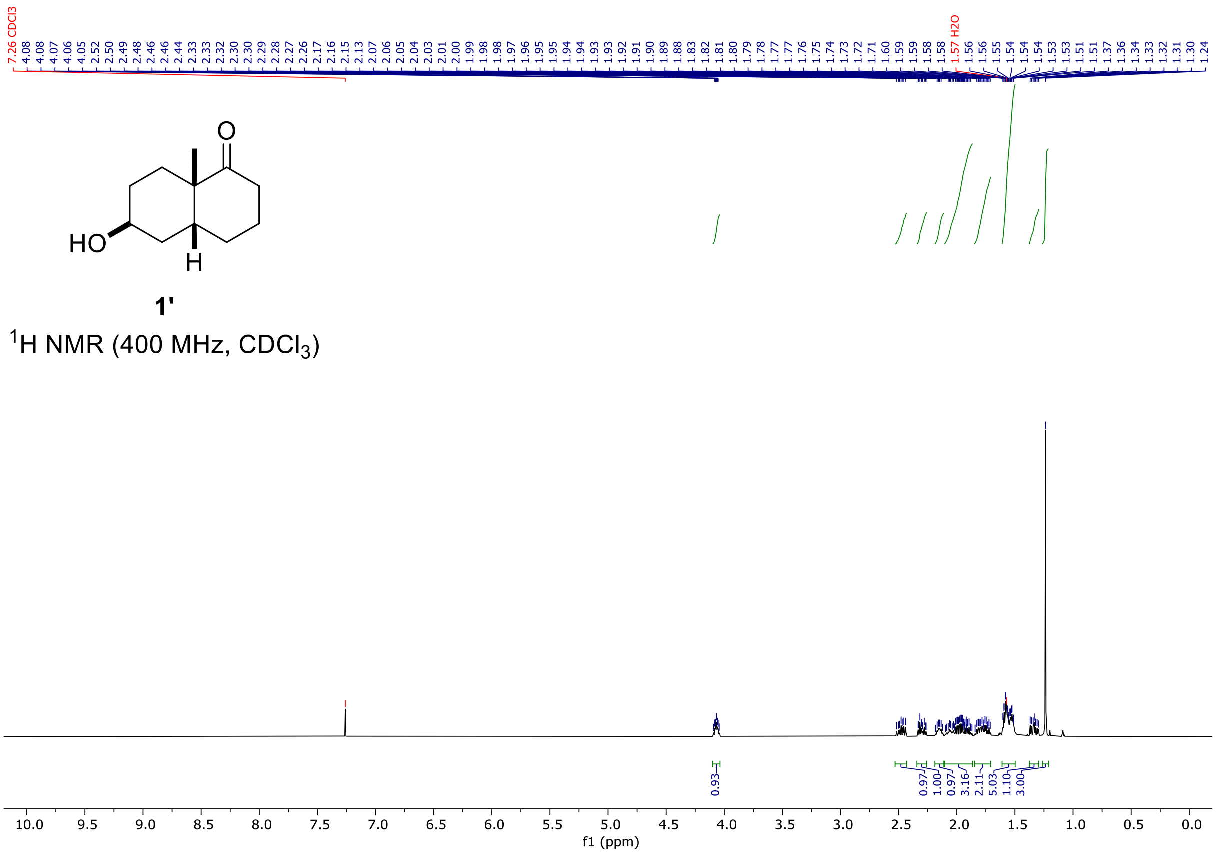

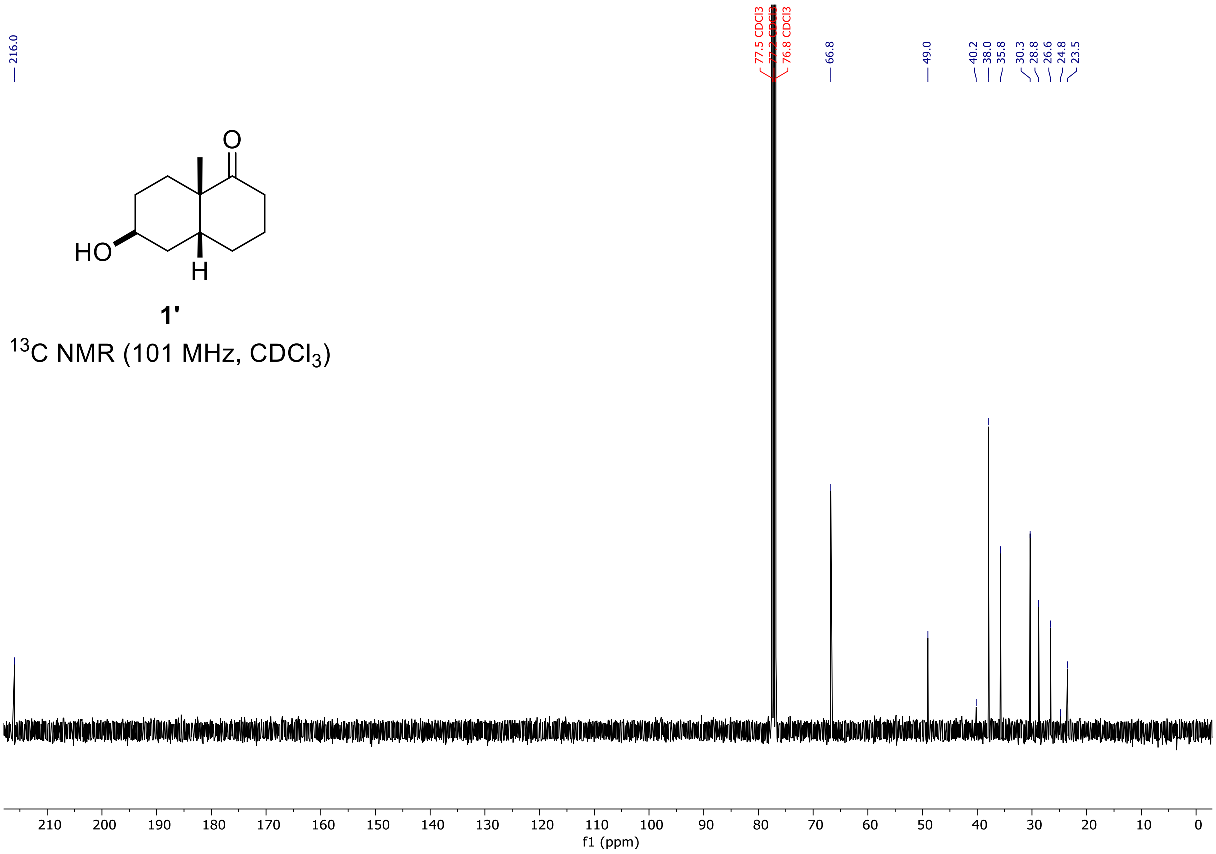

(2*S,4a*R**,6*R**,8a*S**)-2-Bromo-6-hydroxy-8a-methyloctahydronaphthalen-1(2*H*)-one (6) and (2*R**,4a*R**,6*R**,8a*S**)-2-bromo-6-hydroxy-8a-methyloctahydronaphthalen-1(2*H*)-one (6')**

6 (β-Br), **6'** (α-Br)
dr = 20:1 (**6**:**6'**)
^{1}H NMR (400 MHz, $CDCl_3$)

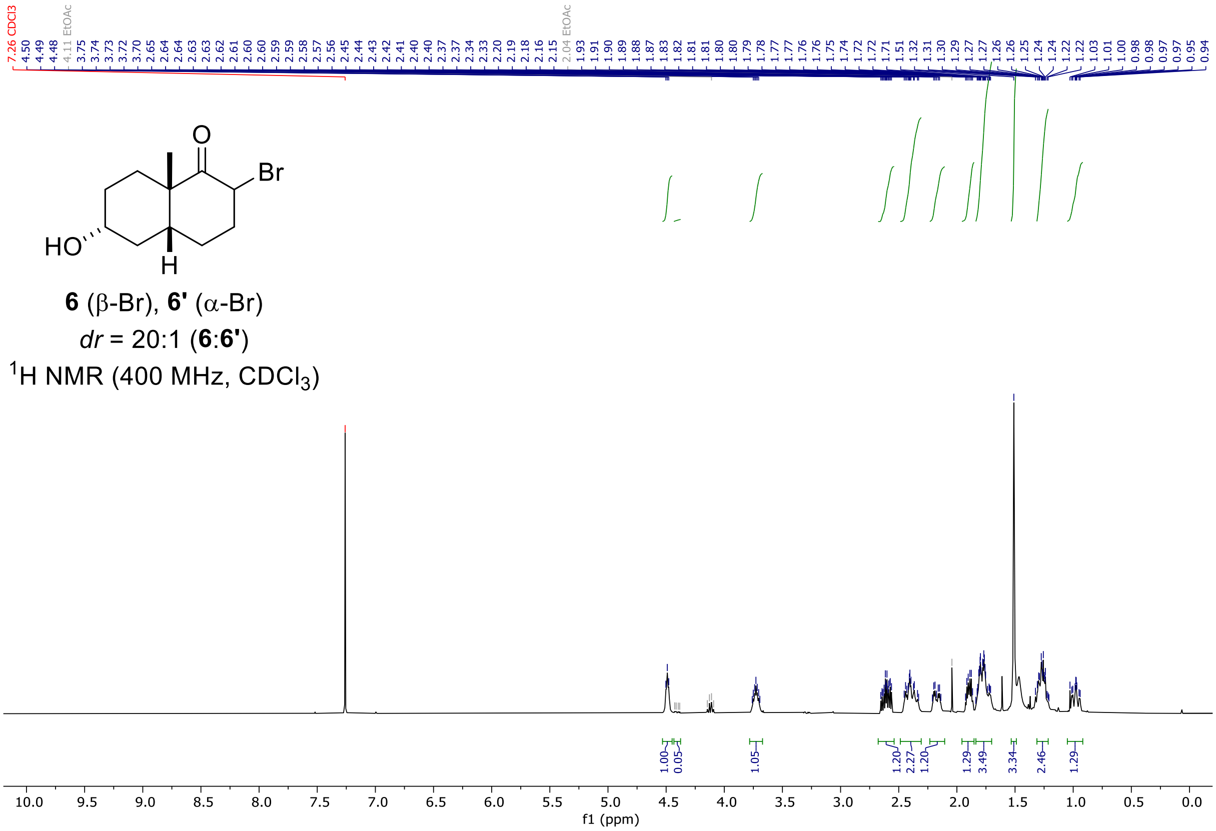

6 (β-Br), **6'** (α-Br)
dr = 20:1 (**6**:**6'**)
^{13}C NMR (101 MHz, $CDCl_3$)

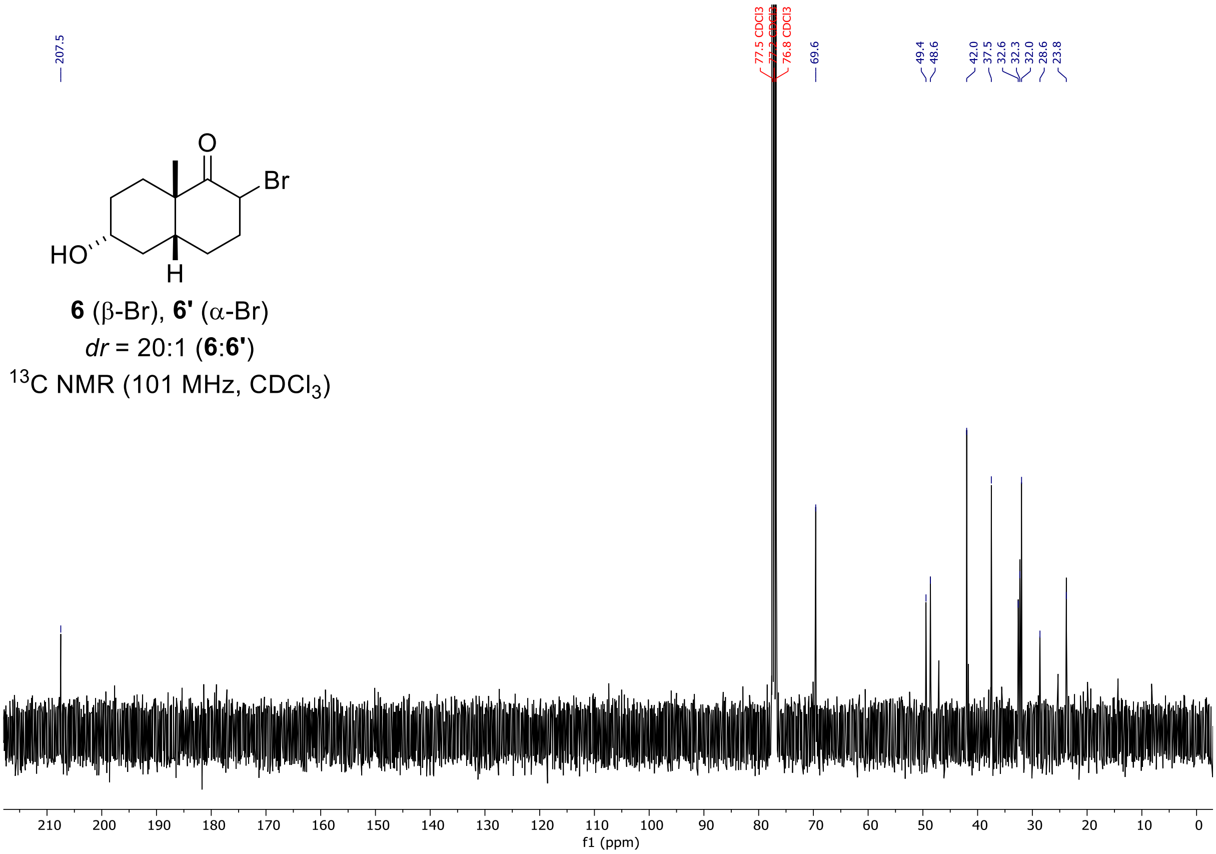

(3*R,4a*R**,12b*S**)-7,12b-Dimethyl-1,2,3,4,4a,5,6,12b-octahydrobenzo[*c*]acridin-3-ol (2a)**

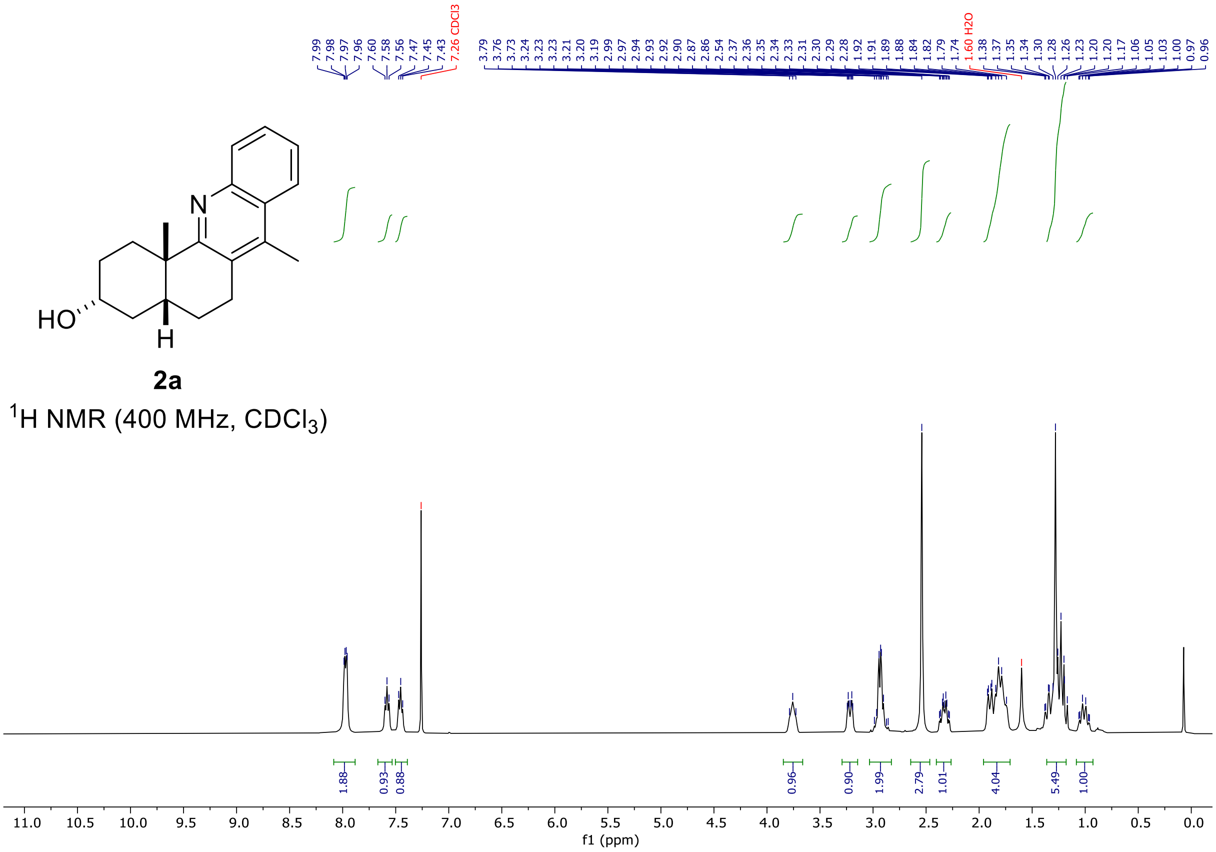

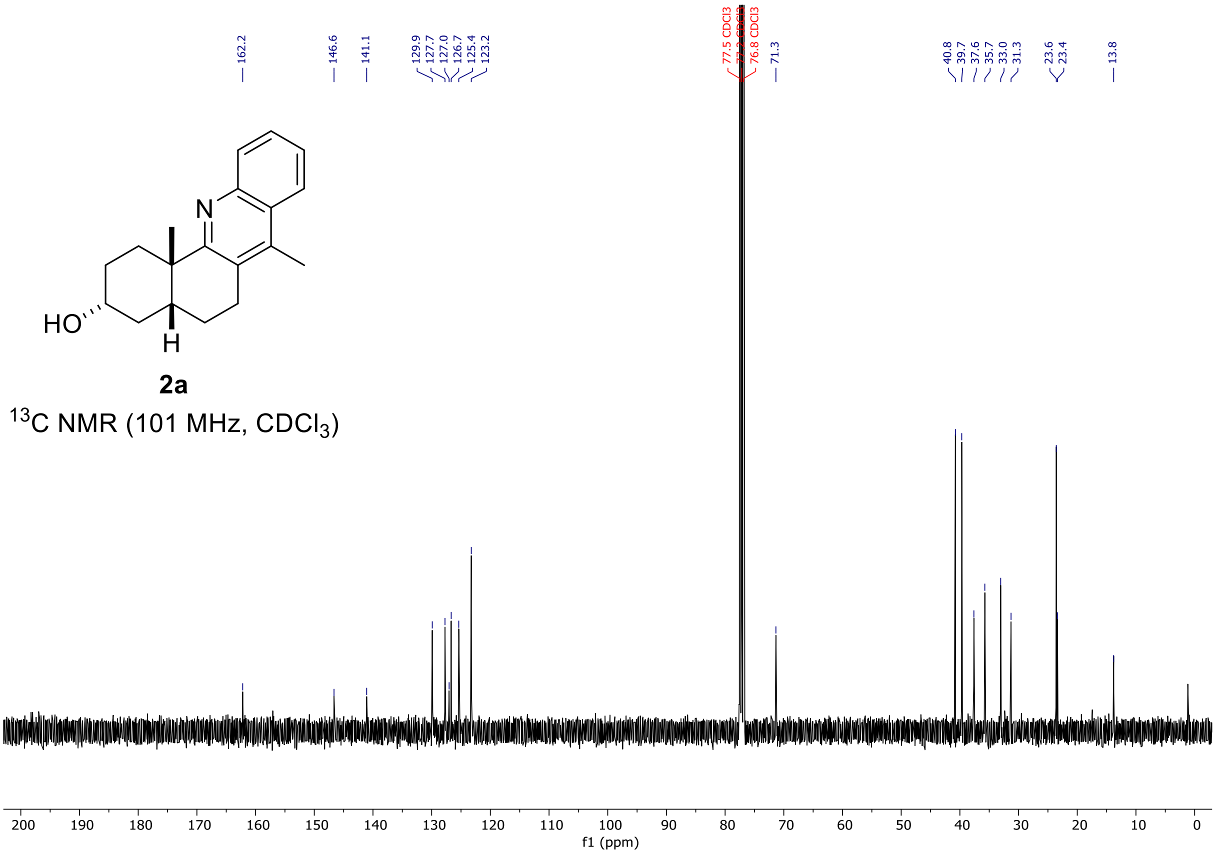

(3*R,4a*R**,12b*S**)-12b-Methyl-7-phenyl-1,2,3,4,4a,5,6,12b-octahydrobenzo[*c*]acridin-3-ol (2b)**

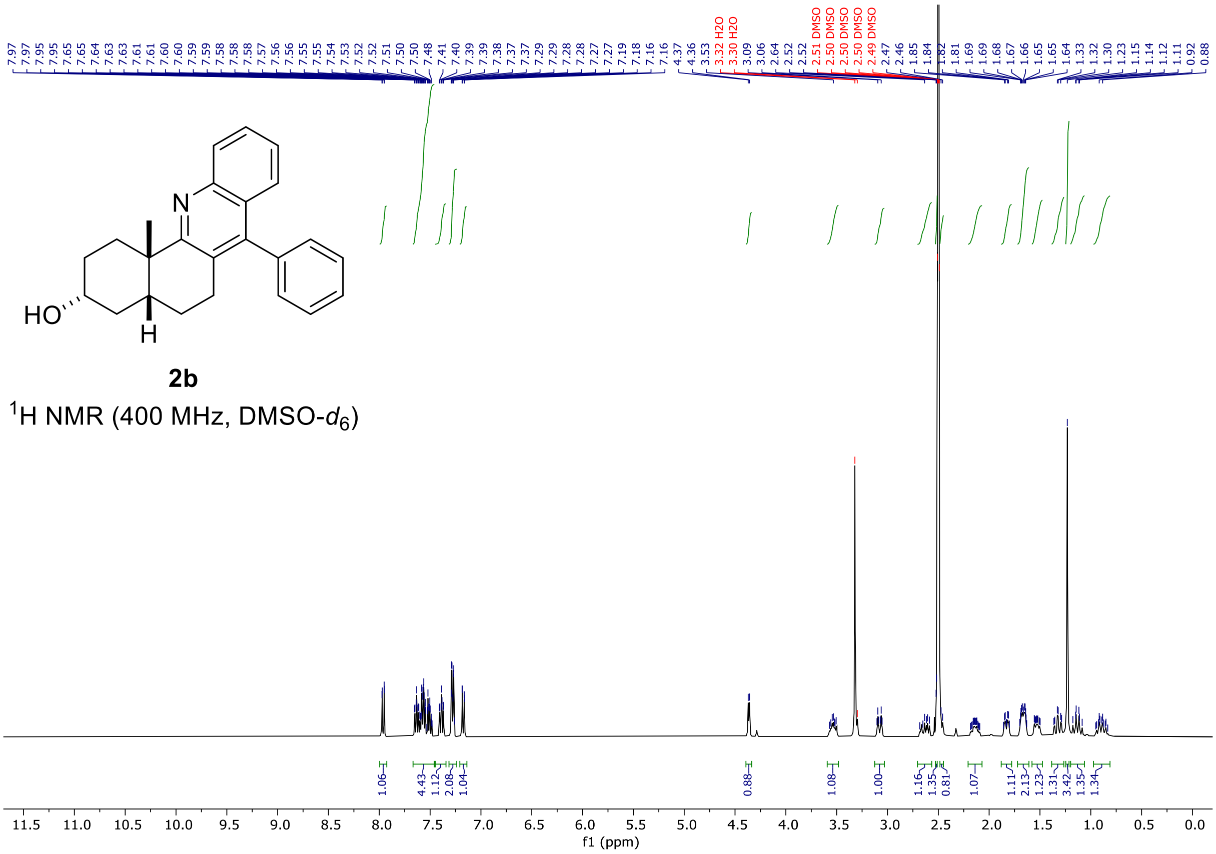

2b

^{1}H NMR (400 MHz, DMSO-d_6)

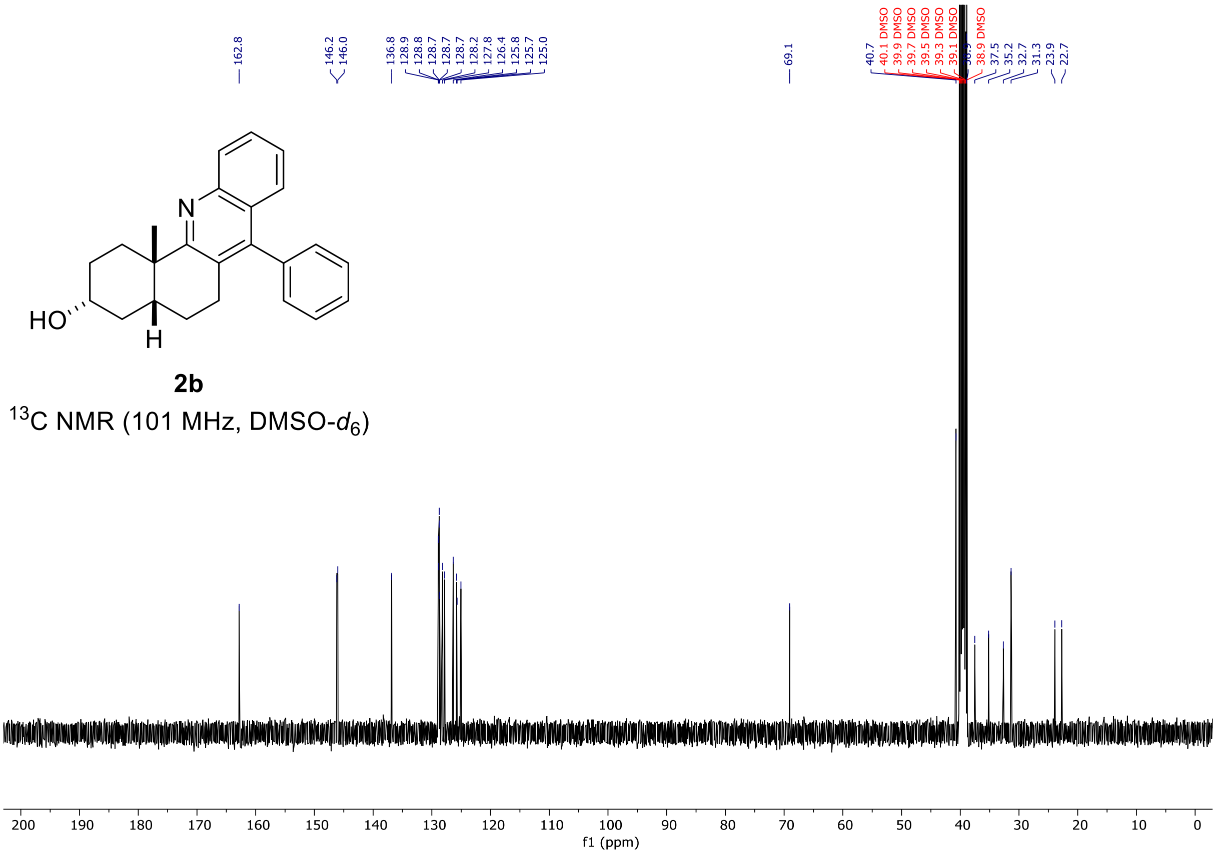

2b

^{13}C NMR (101 MHz, DMSO-d_6)

(3*R,4a*R**,12b*S**)-10-Bromo-7,12b-dimethyl-1,2,3,4,4a,5,6,12b-octahydrobenzo[*c*]acridin-3-ol (2c)**

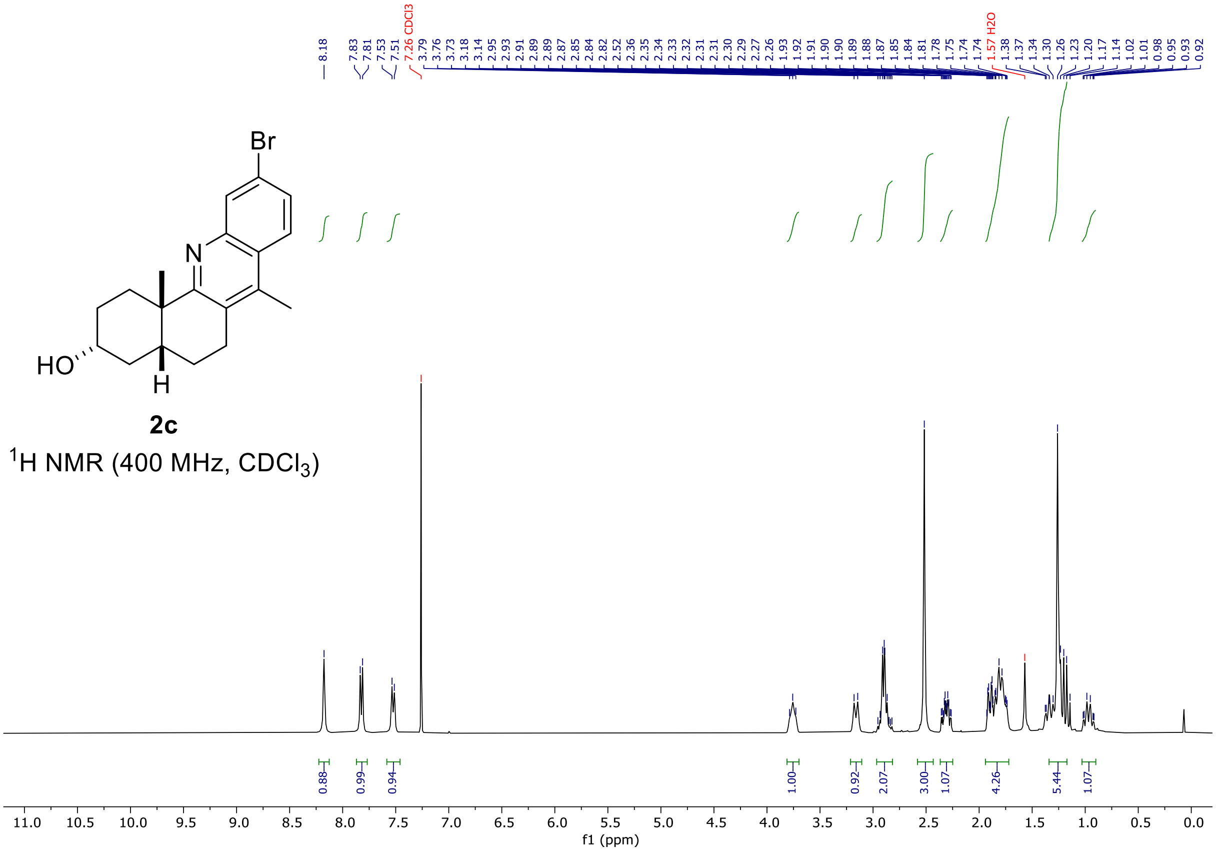

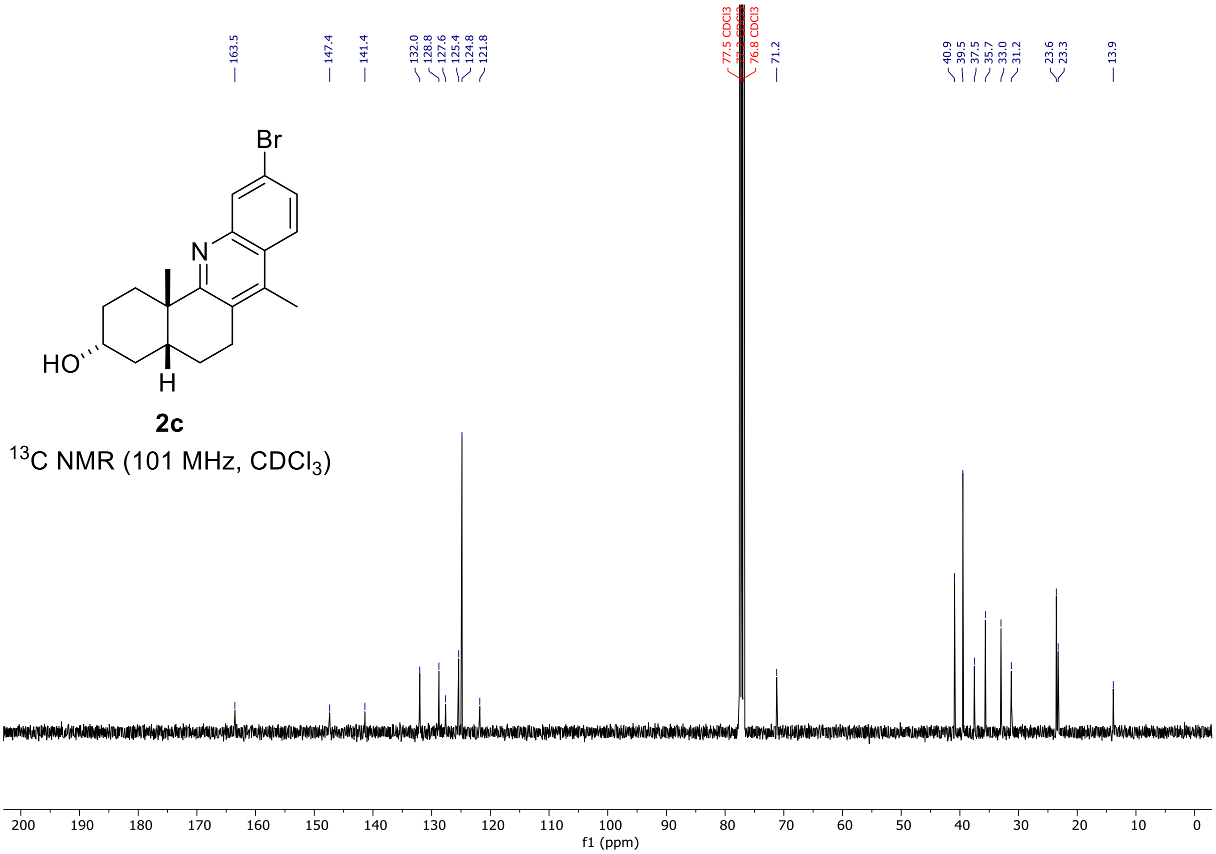

(3*R,4a*R**,12b*S**)-7-(4-Fluorophenyl)-12b-methyl-1,2,3,4,4a,5,6,12b-octahydrobenzo[*c*]acridin-3-ol (2d)**

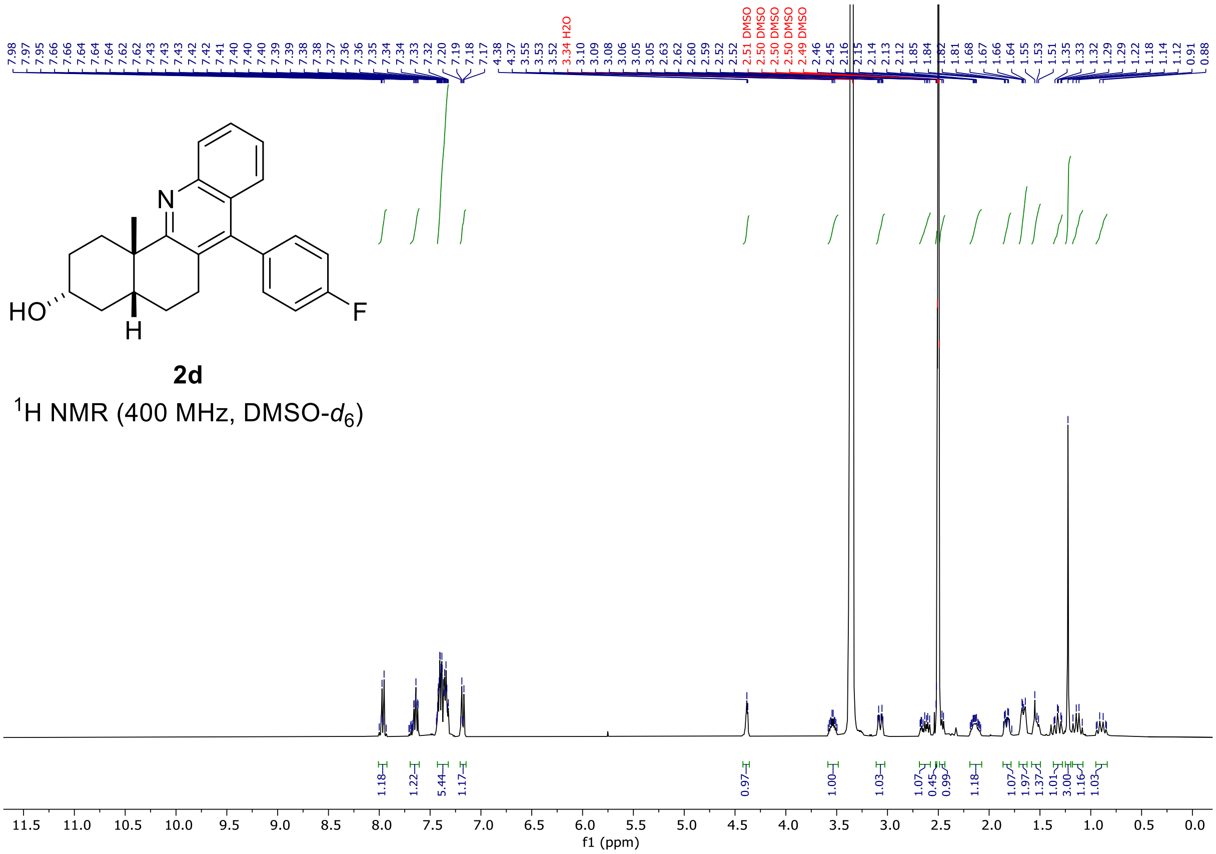

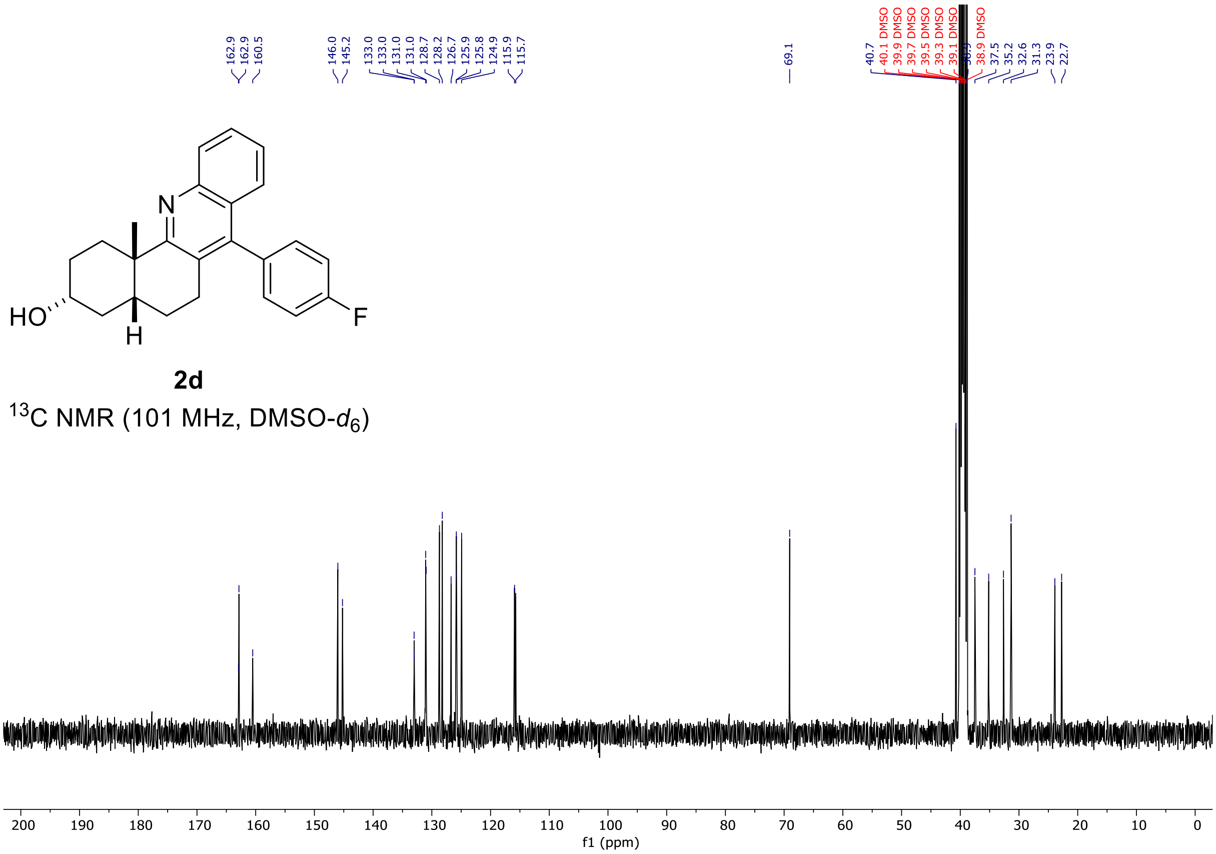

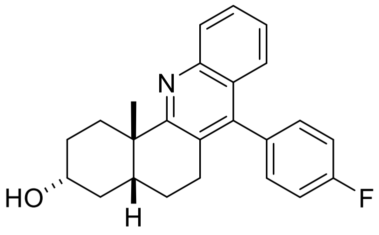

2d

^{19}F NMR (377 MHz, DMSO-d_6)

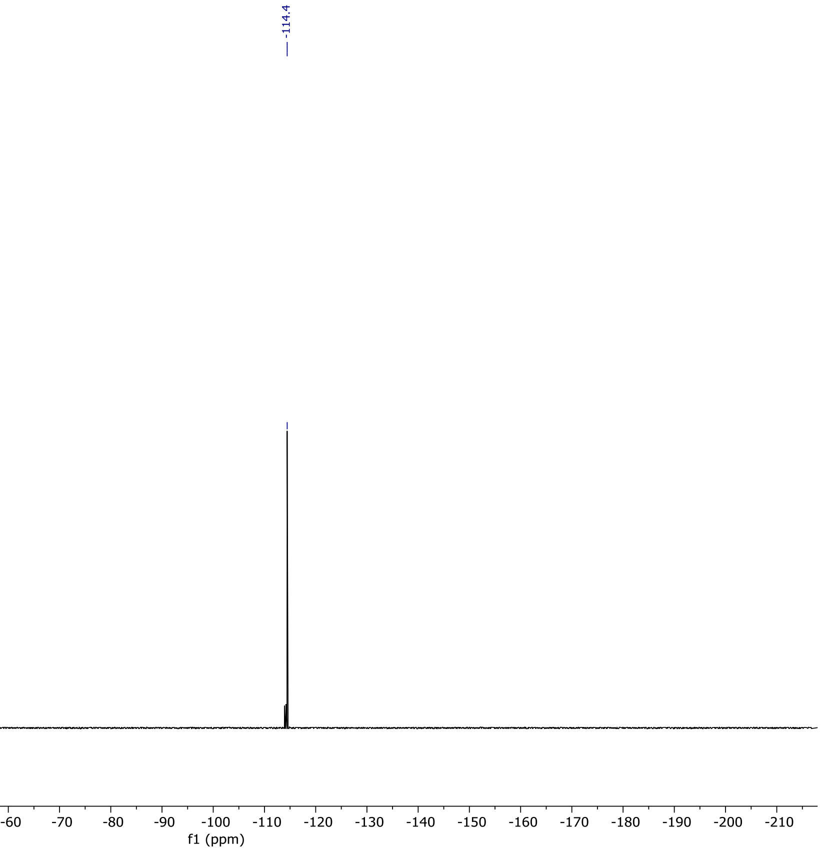

(3*R,4a*R**,12b*S**)-9-Chloro-12b-methyl-7-phenyl-1,2,3,4,4a,5,6,12b-octahydrobenzo[*c*]acridin-3-ol (2e)**

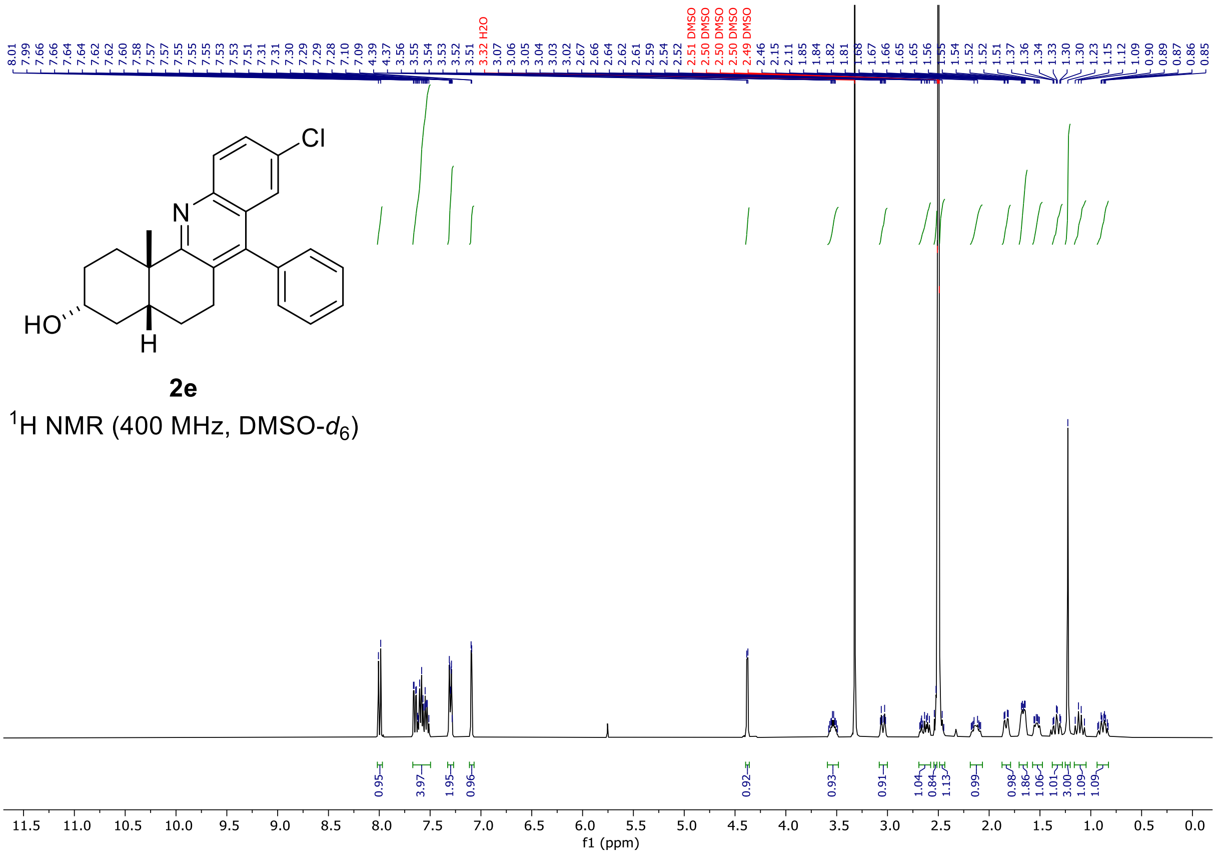

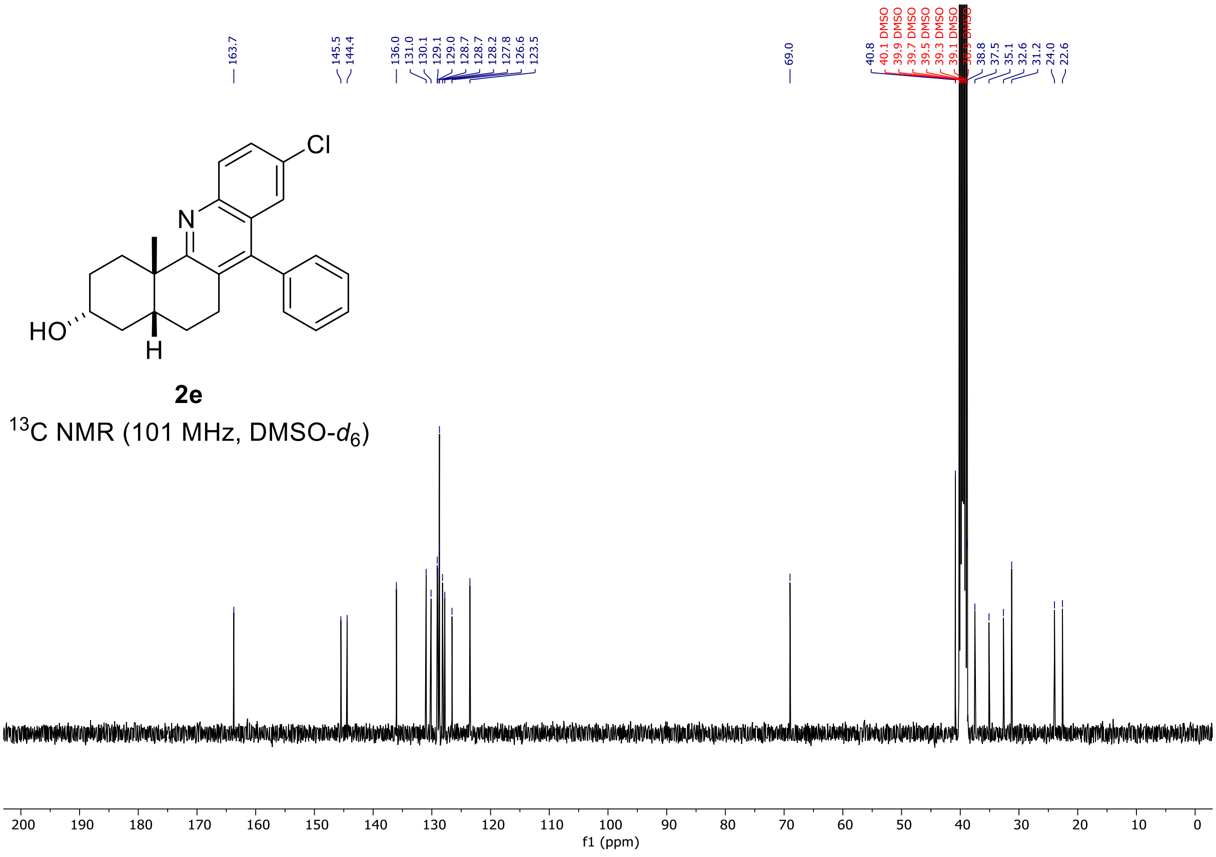

(3*R,4a*R**,11b*S**)-11b-Methyl-2,3,4,4a,5,6,11,11b-octahydro-1*H*-benzo[*g*]pyrido[2,3-*b*]indol-3-ol (3)**

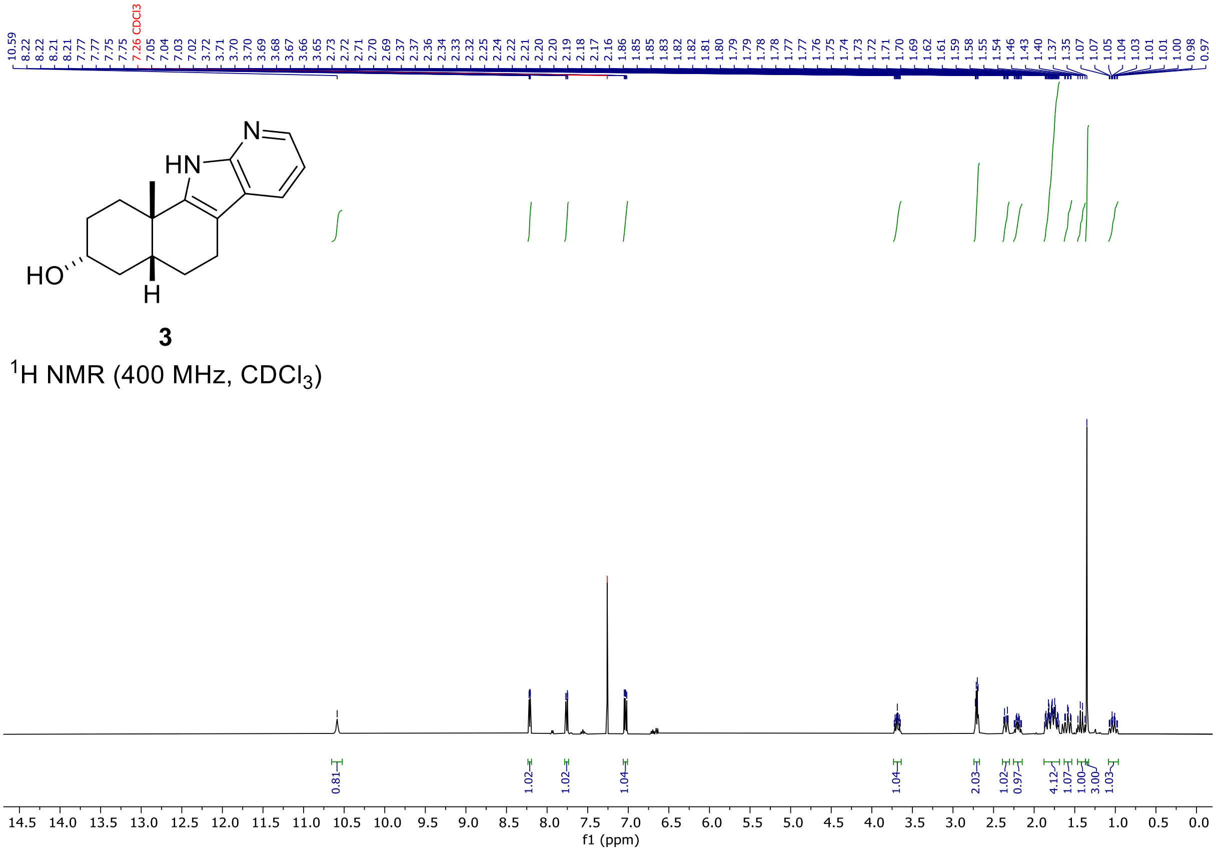

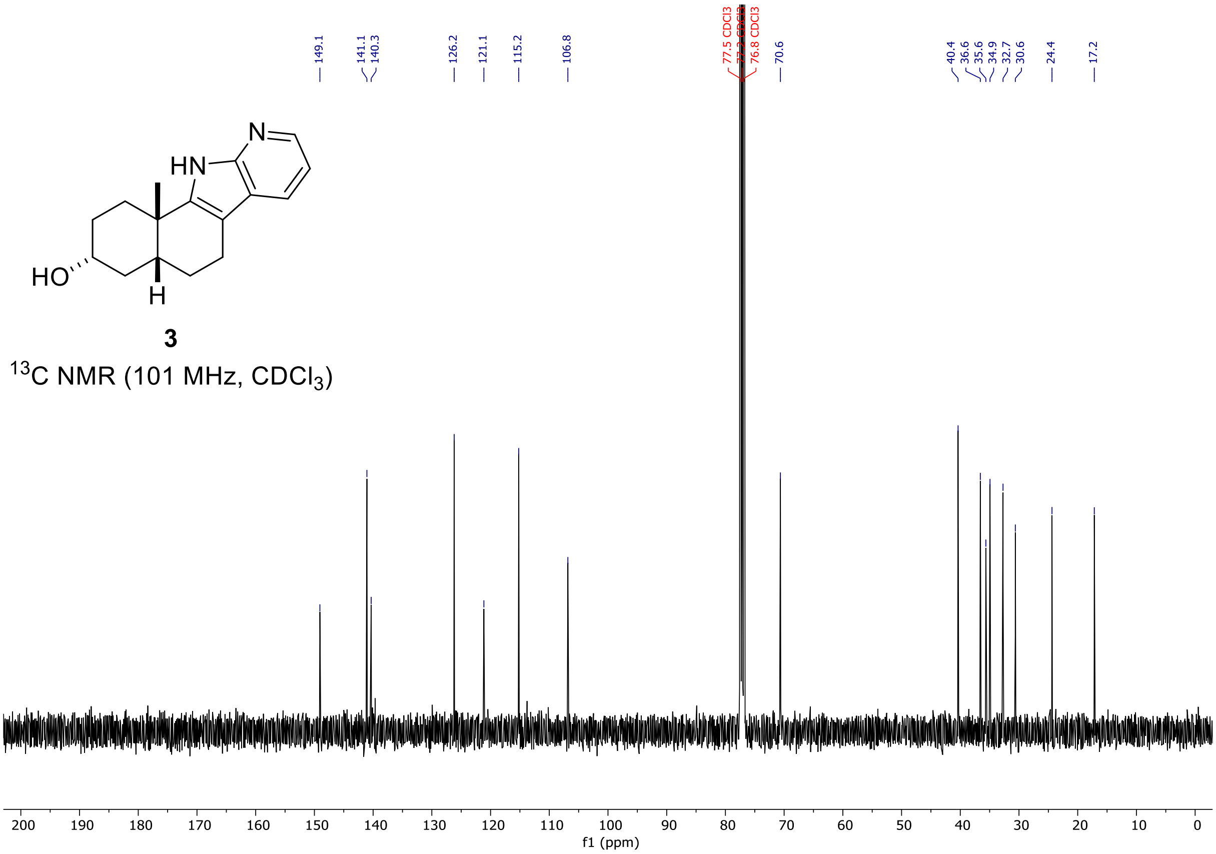

(1*S,4a*R**,6*R**,8a*S**)-8a-Methyl-3,4,4a,5,6,7,8,8a-octahydro-2*H*,5'*H*-spiro[naphthalene-1,4'-pyrrolo[1,2-*a*]quinoxalin]-6-ol (4)**

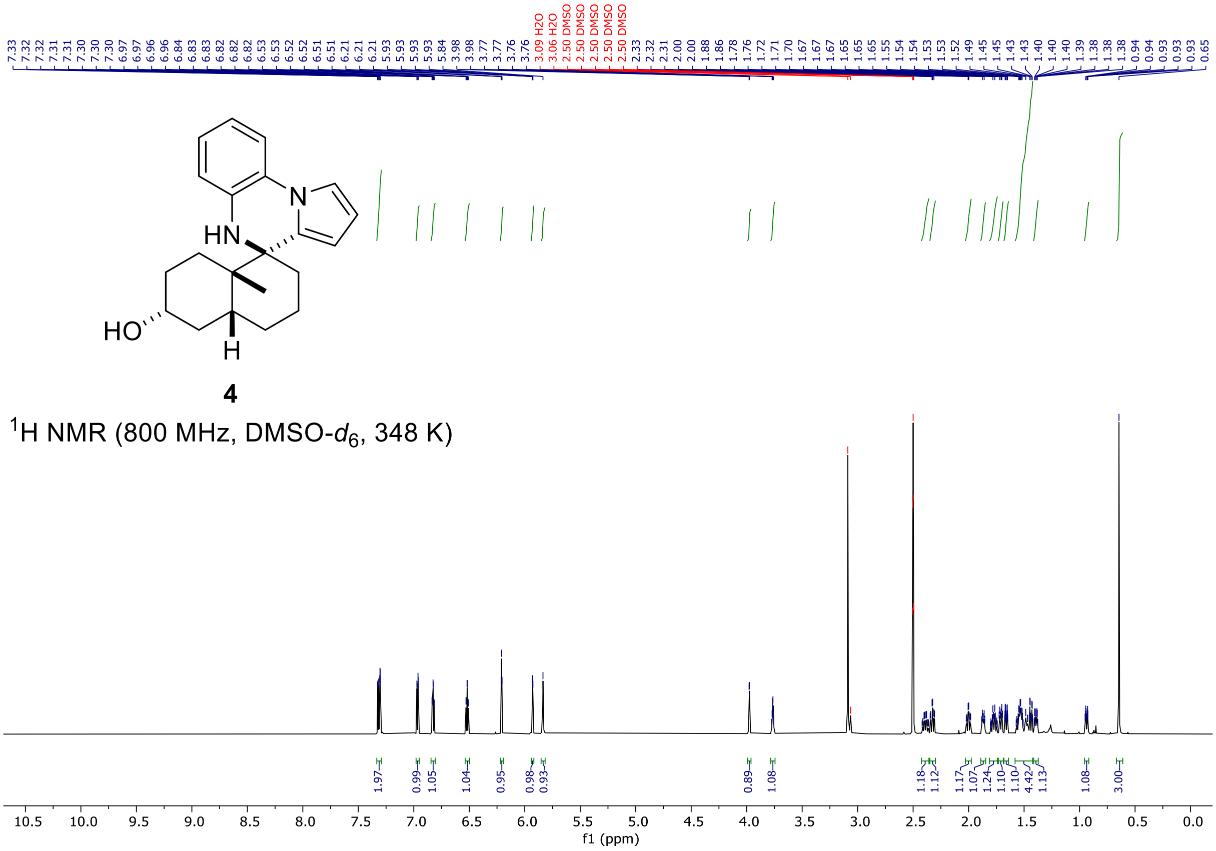

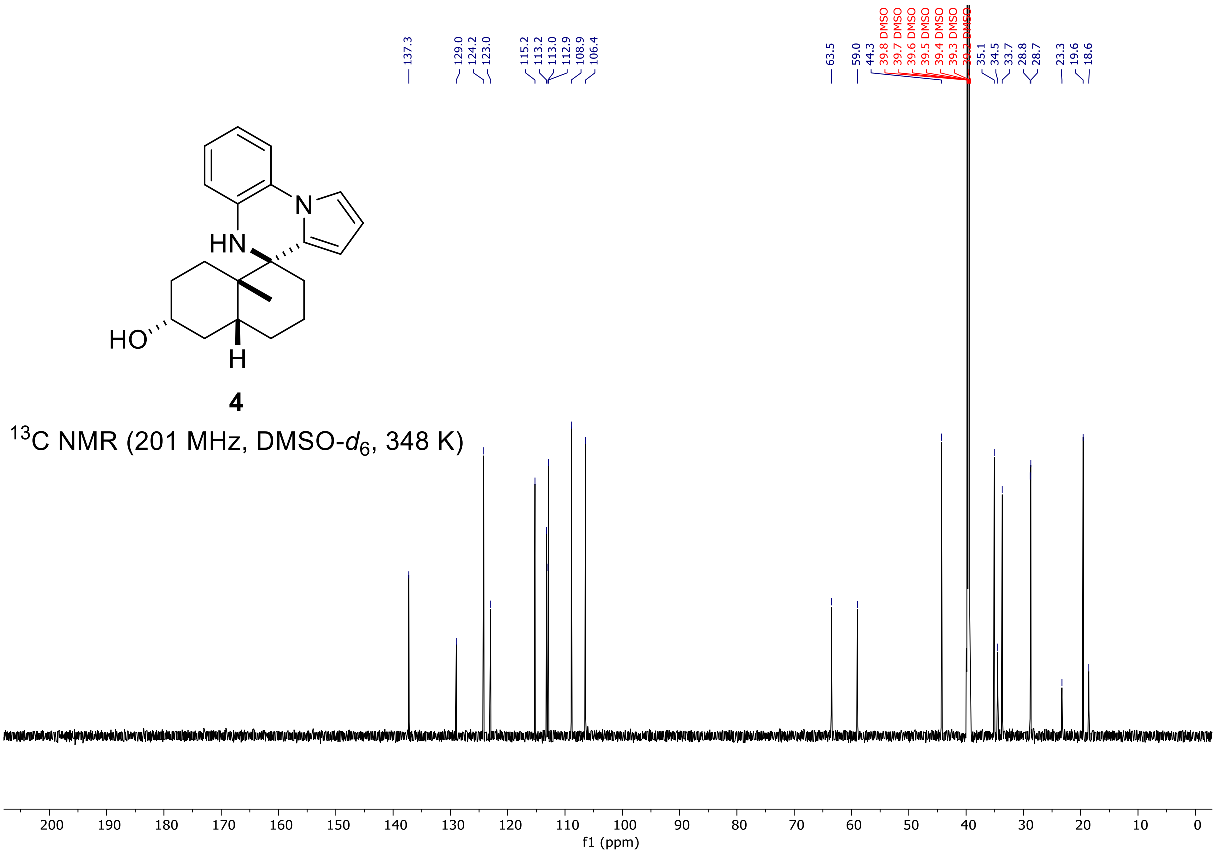

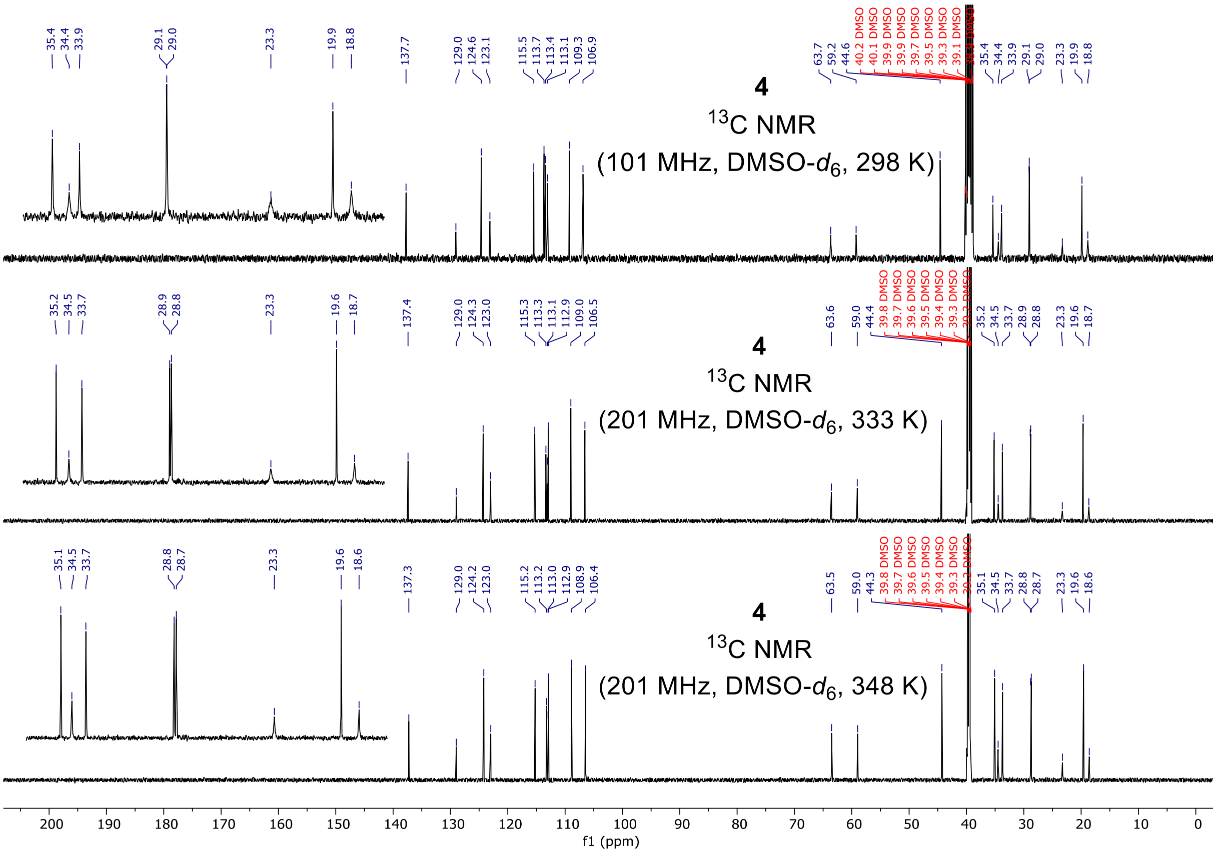
4
^{13}C NMR
(101 MHz, DMSO-d_6, 298 K)
4
^{13}C NMR
(201 MHz, DMSO-d_6, 333 K)
4
^{13}C NMR
(201 MHz, DMSO-d_6, 348 K)
f1 (ppm)

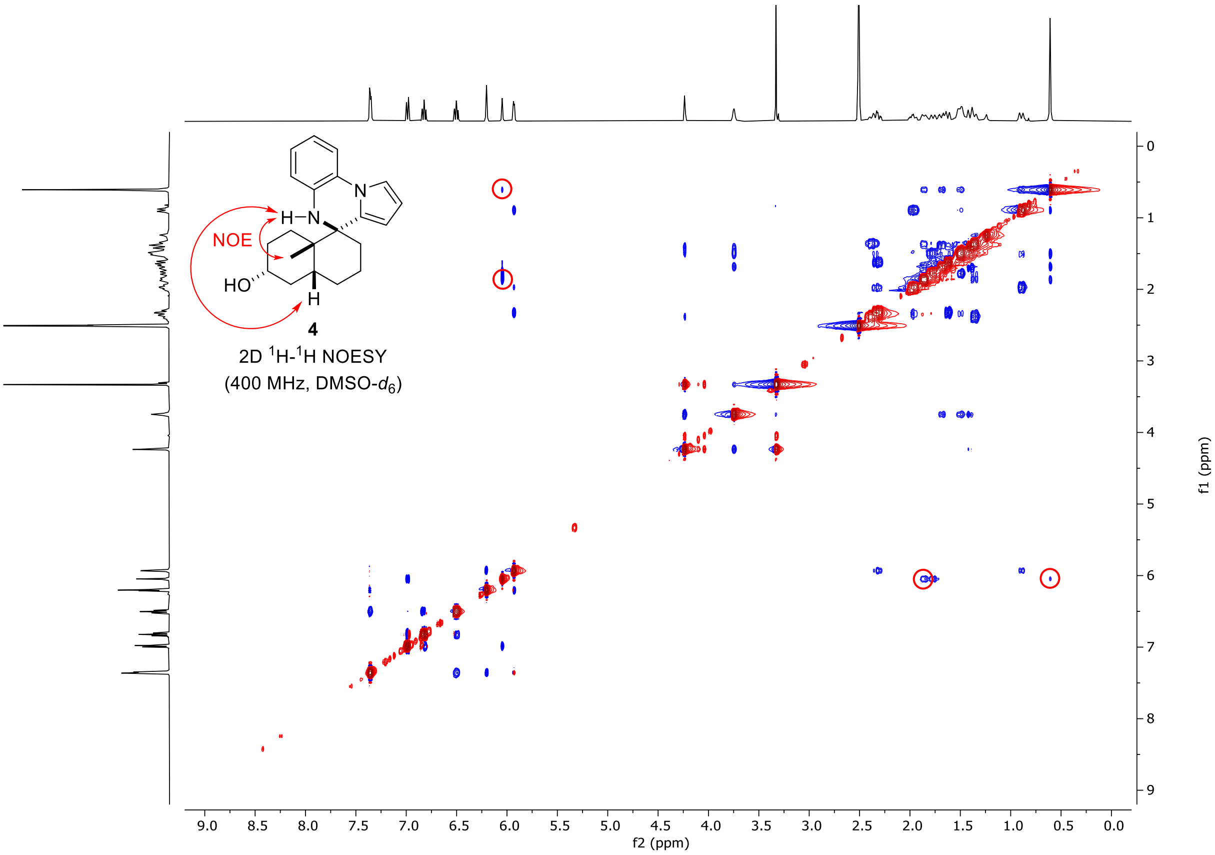
NOE
H–N
N
HO
H
4
2D ^{1}H-^{1}H NOESY
(400 MHz, DMSO-d_6)
f2 (ppm)
f1 (ppm)

(1*R,4a*R**,6*R**,8a*S**)-8a-Methyl-3,4,4a,5,6,7,8,8a-octahydro-2*H*,5'*H*-spiro[naphthalene-1,4'-pyrrolo[1,2-*a*]quinoxalin]-6-ol (4')**

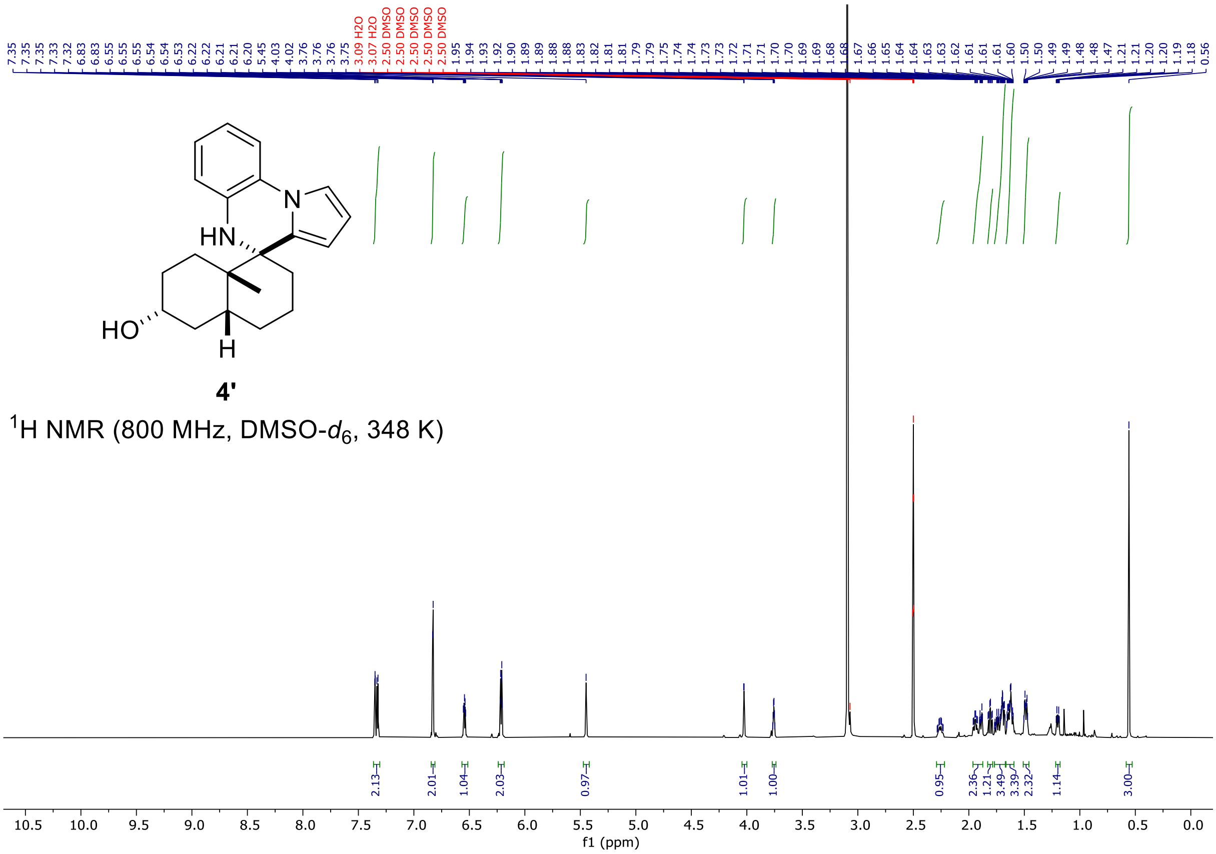

4'

^{1}H NMR (800 MHz, DMSO-d_6, 348 K)

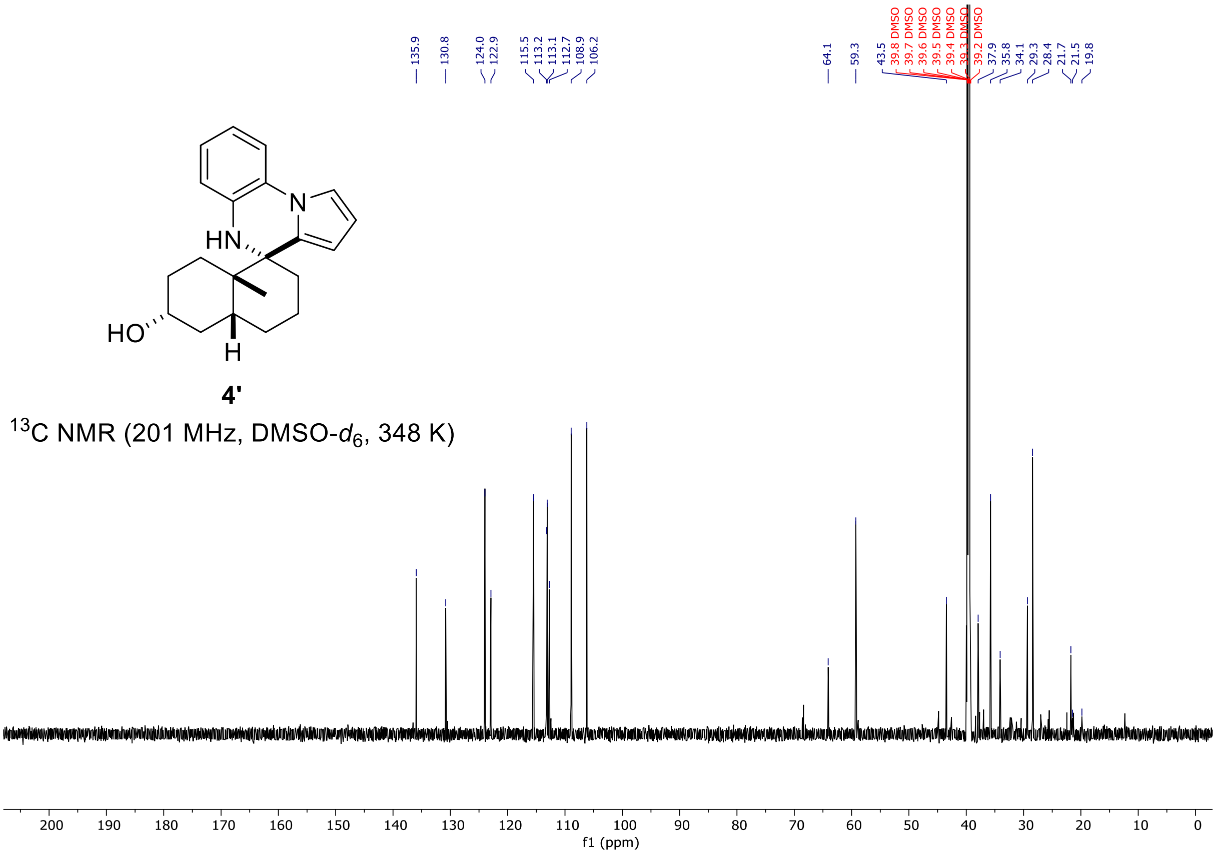

4'

^{13}C NMR (201 MHz, DMSO-d_6, 348 K)

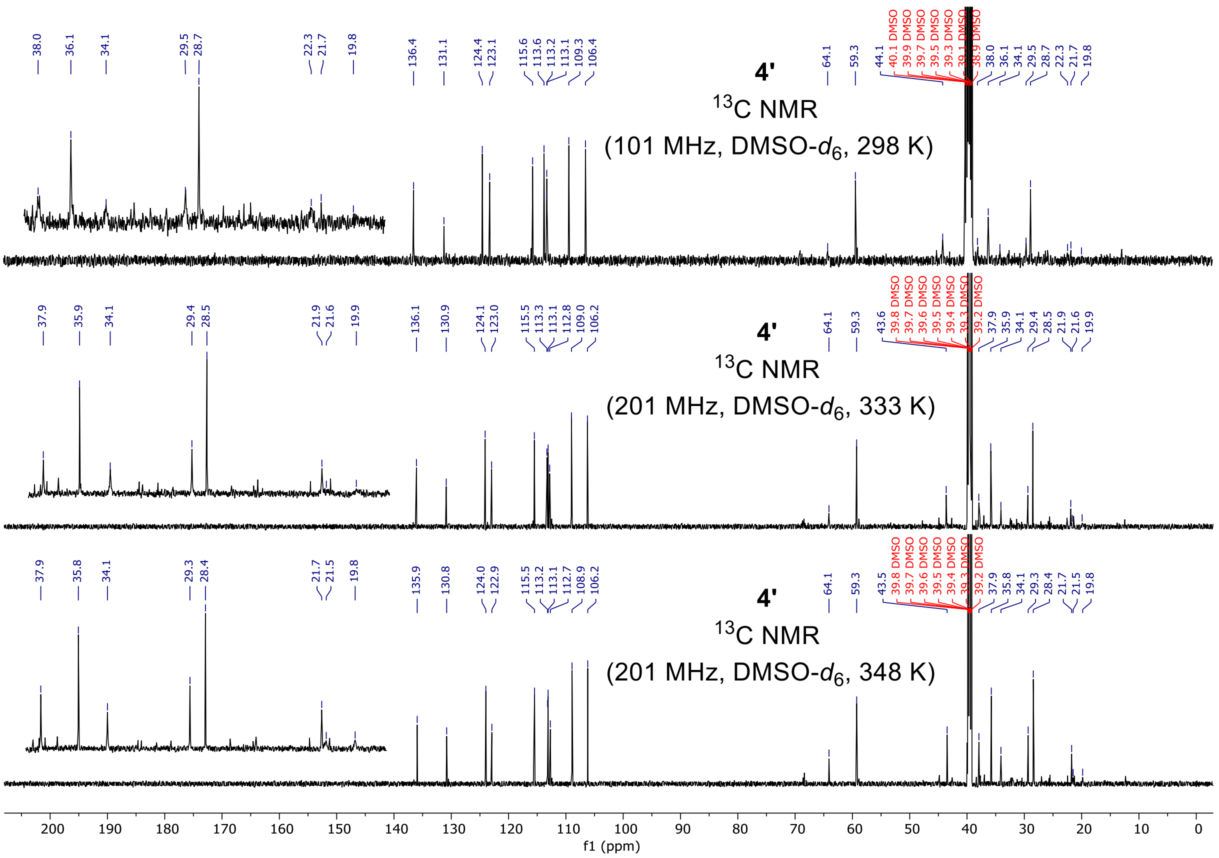
4'
13C NMR
(101 MHz, DMSO-d6, 298 K)
4'
13C NMR
(201 MHz, DMSO-d6, 333 K)
4'
13C NMR
(201 MHz, DMSO-d6, 348 K)
f1 (ppm)

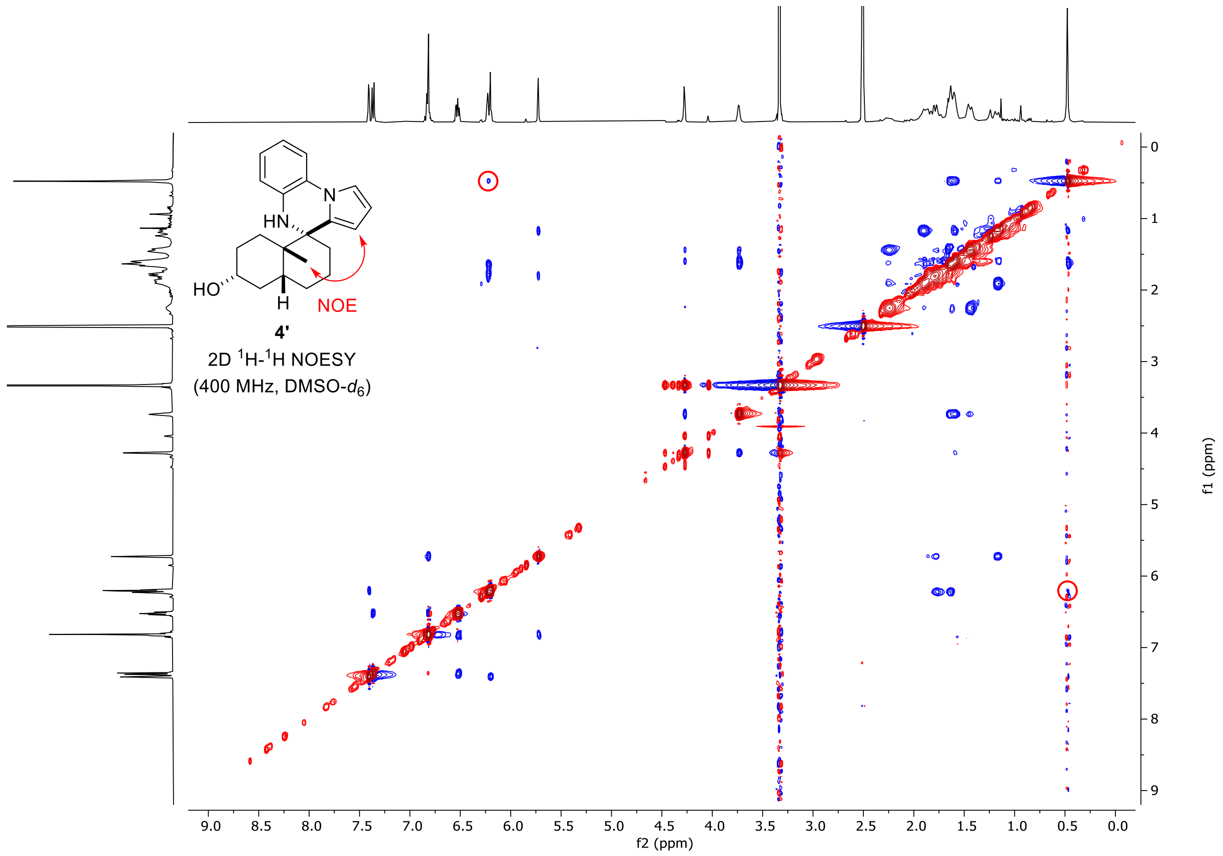
HN
N
HO
H
NOE
4'
2D 1H-1H NOESY
(400 MHz, DMSO-d6)
f2 (ppm)
f1 (ppm)

(1*S,4a*R**,6*R**,8a*S**)-6-Hydroxy-8a-methyl-3,4,4a,5,6,7,8,8a-octahydro-1'*H*,2*H*-spiro[naphthalene-1,2'-quinazolin]-4'(3'*H*)-one (5a)**

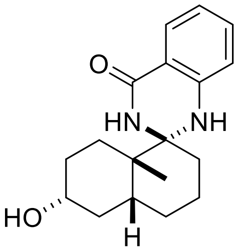

5a

^{1}H NMR (800 MHz, DMSO-d_6, 298 K)

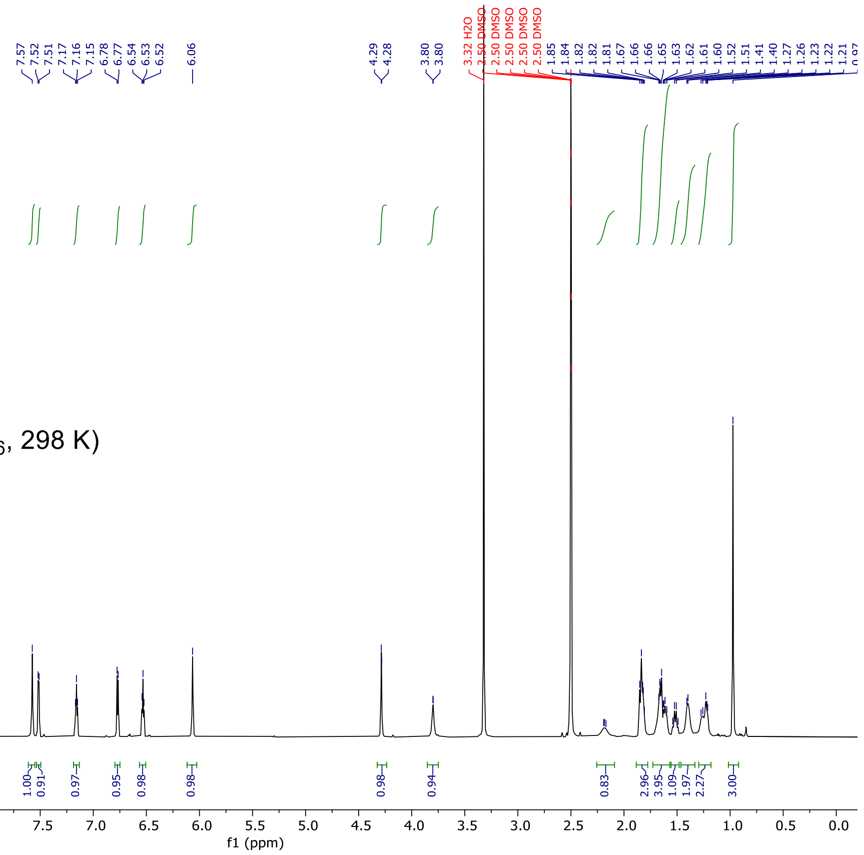

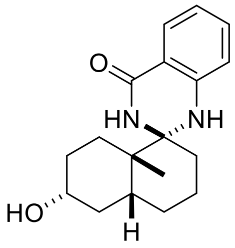

5a

^{13}C NMR (151 MHz, DMSO-d_6, 353 K)

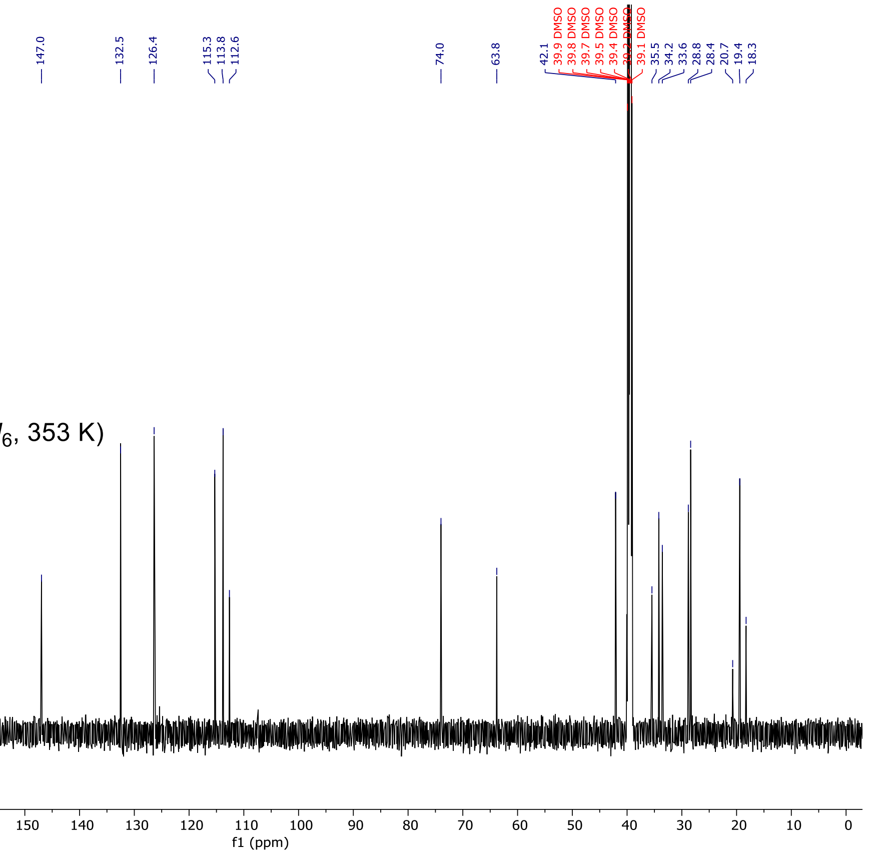

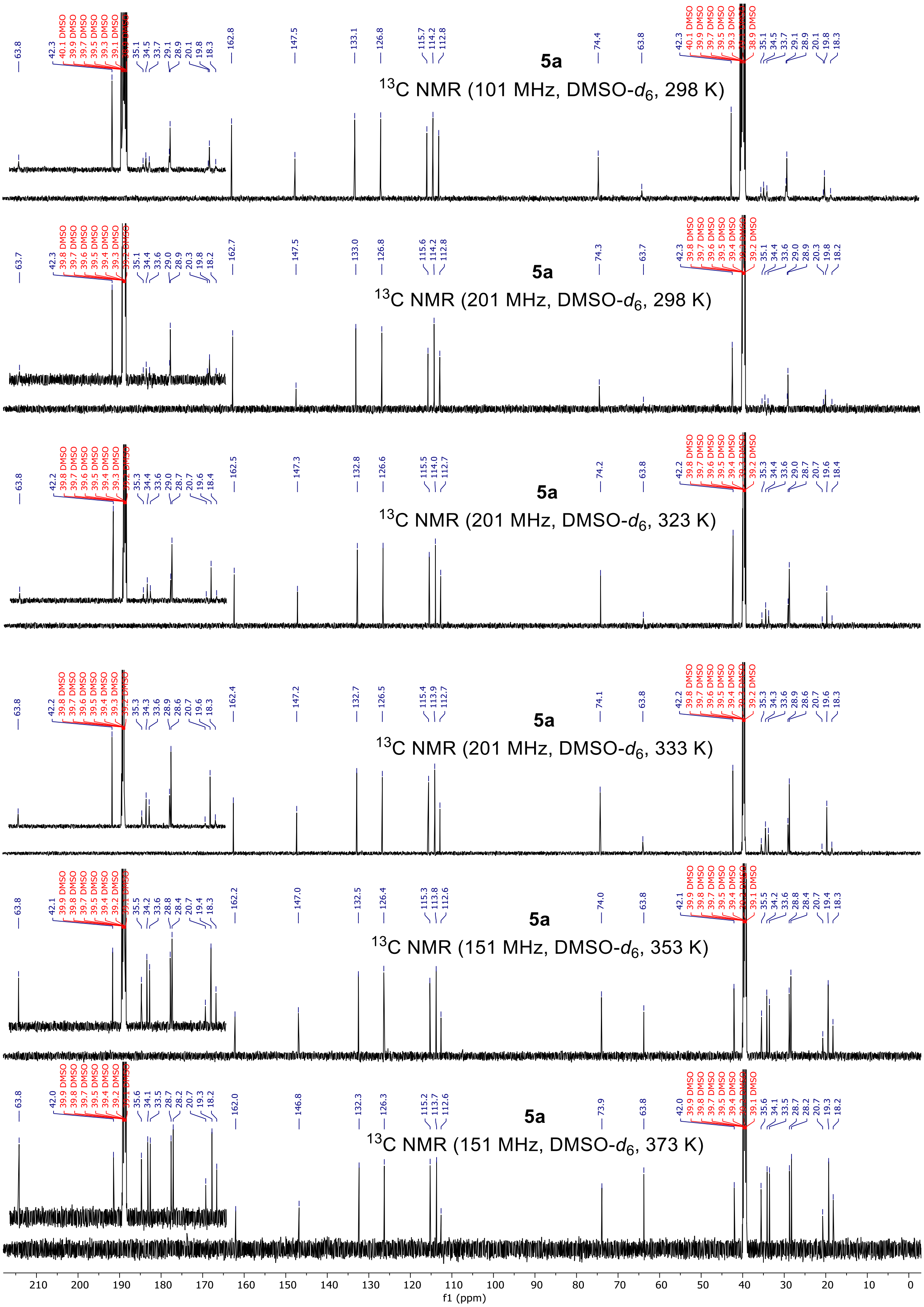
5a
13C NMR (101 MHz, DMSO-d6, 298 K)
5a
13C NMR (201 MHz, DMSO-d6, 298 K)
5a
13C NMR (201 MHz, DMSO-d6, 323 K)
5a
13C NMR (201 MHz, DMSO-d6, 333 K)
5a
13C NMR (151 MHz, DMSO-d6, 353 K)
5a
13C NMR (151 MHz, DMSO-d6, 373 K)
f1 (ppm)

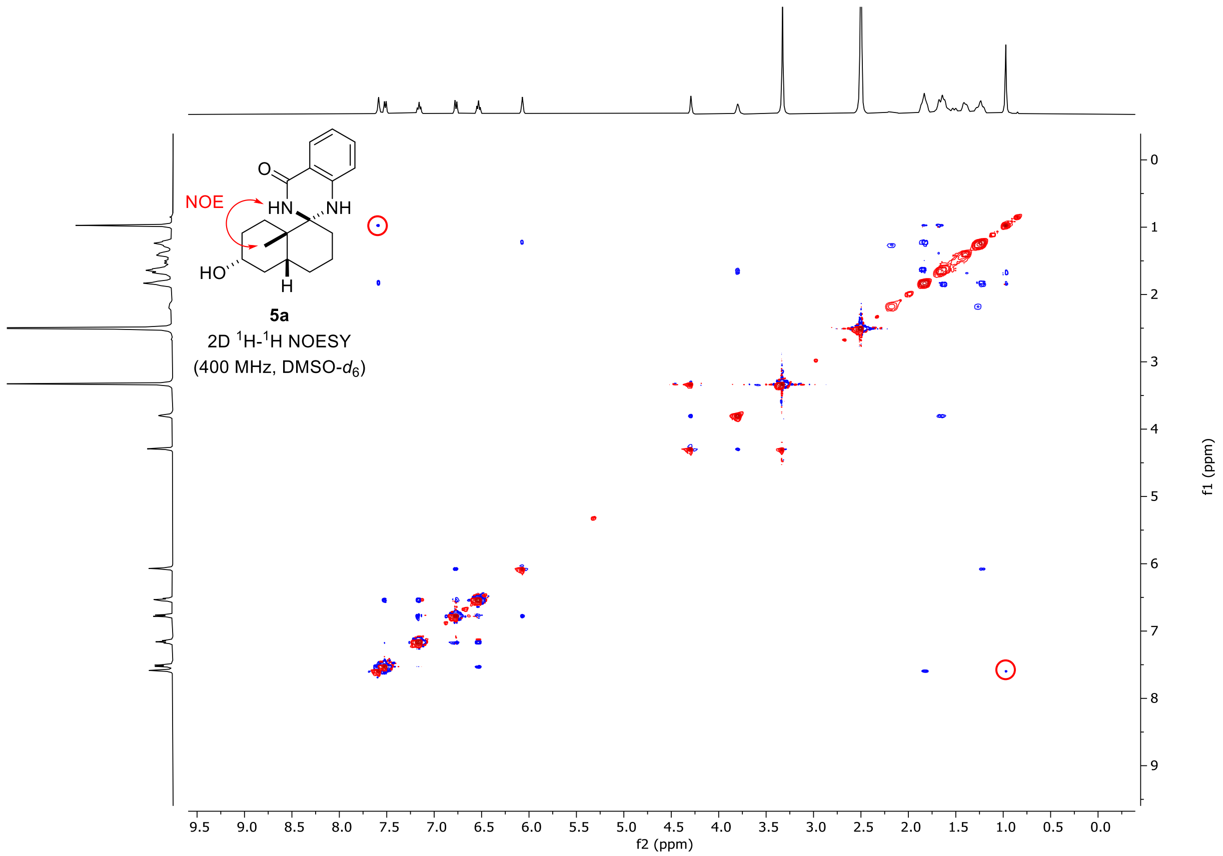
NOE
O
HN
NH
HO
H
5a
2D 1H-1H NOESY
(400 MHz, DMSO-d6)
f1 (ppm)
f2 (ppm)

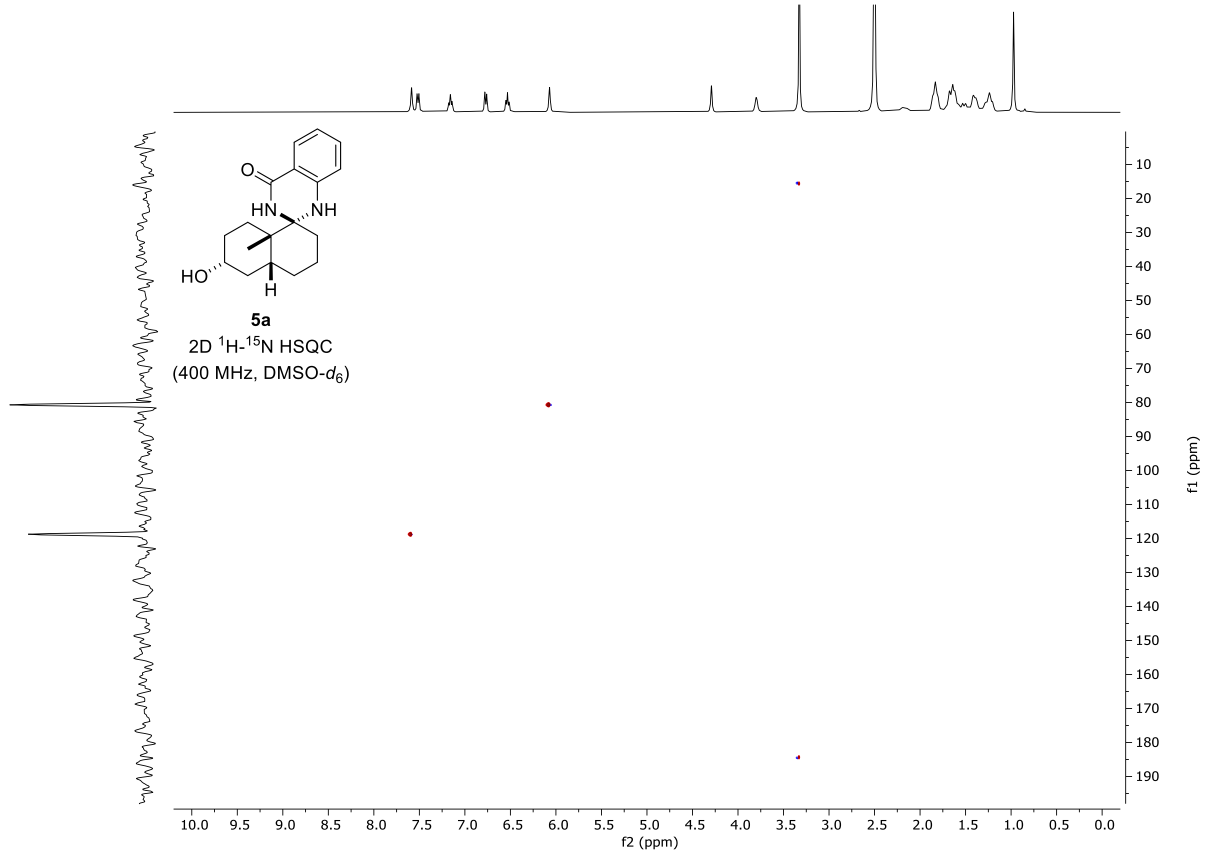
O
HN
NH
HO
H
5a
2D 1H-15N HSQC
(400 MHz, DMSO-d6)
f1 (ppm)
f2 (ppm)

(1*R,4a*R**,6*R**,8a*S**)-6-Hydroxy-8a-methyl-3,4,4a,5,6,7,8,8a-octahydro-1'*H*,2*H*-spiro[naphthalene-1,2'-quinazolin]-4'(3'*H*)-one (5a')**

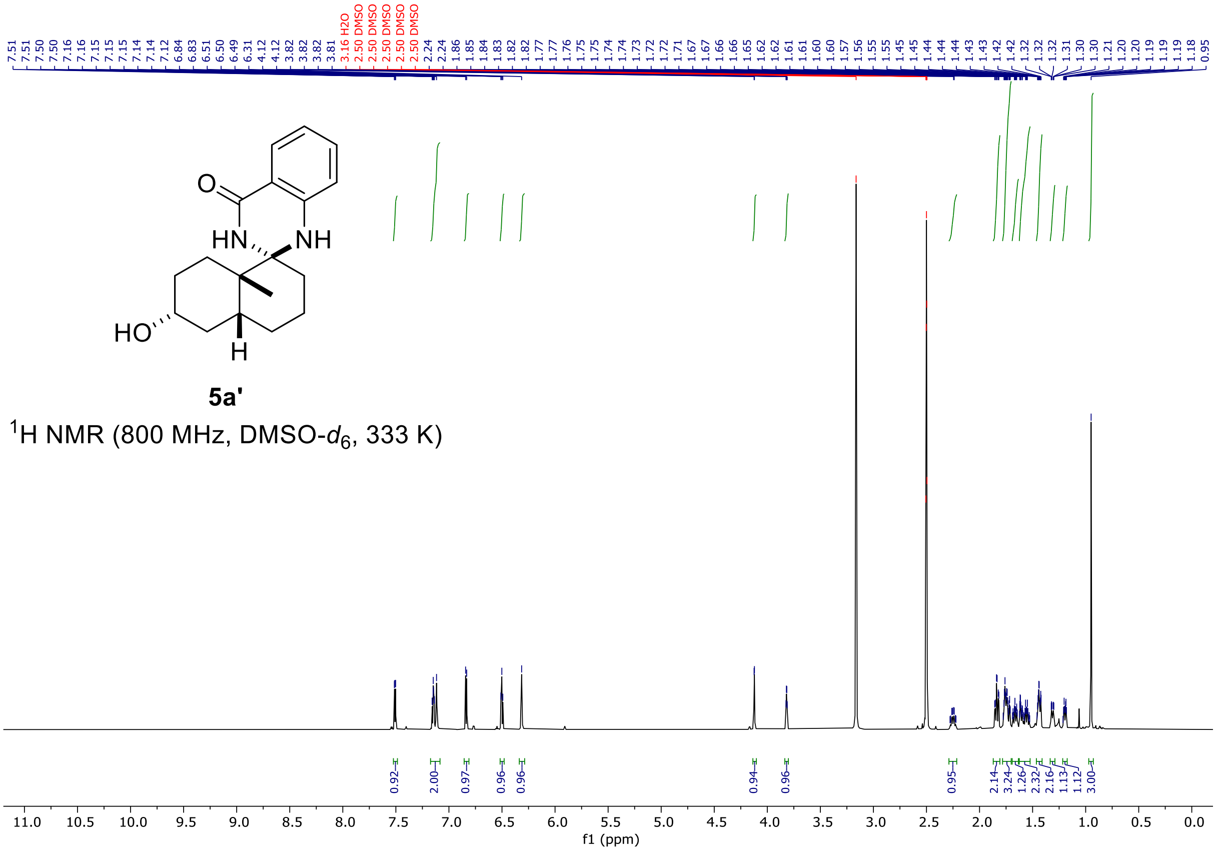

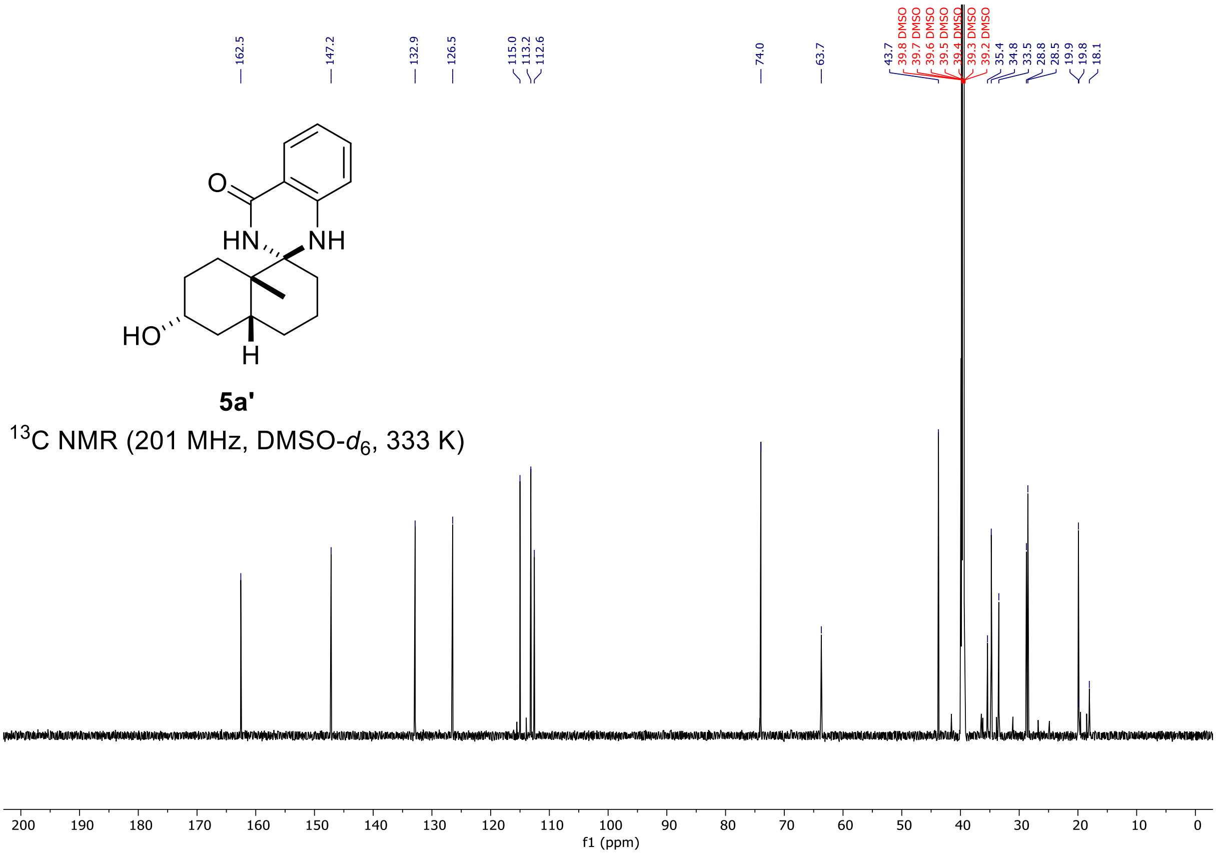

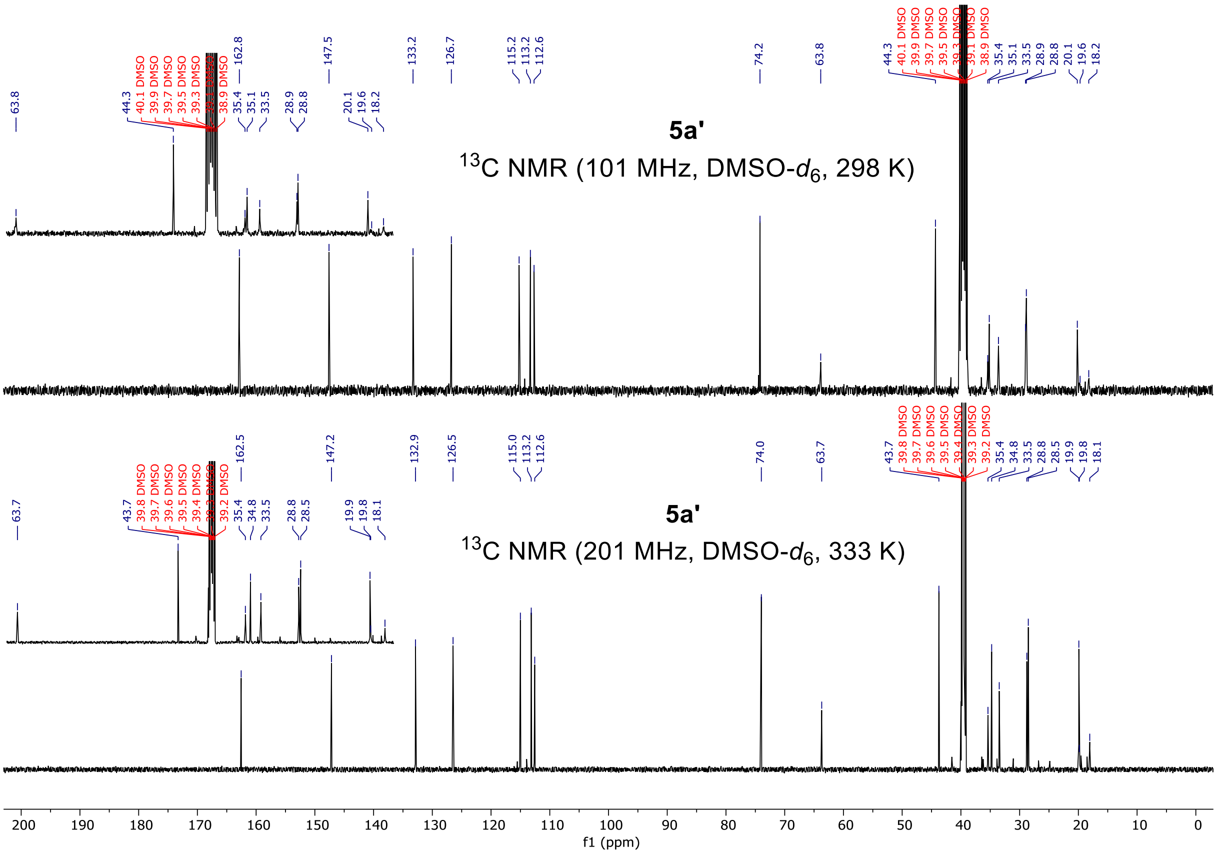
5a'
13C NMR (101 MHz, DMSO-d6, 298 K)
5a'
13C NMR (201 MHz, DMSO-d6, 333 K)
f1 (ppm)

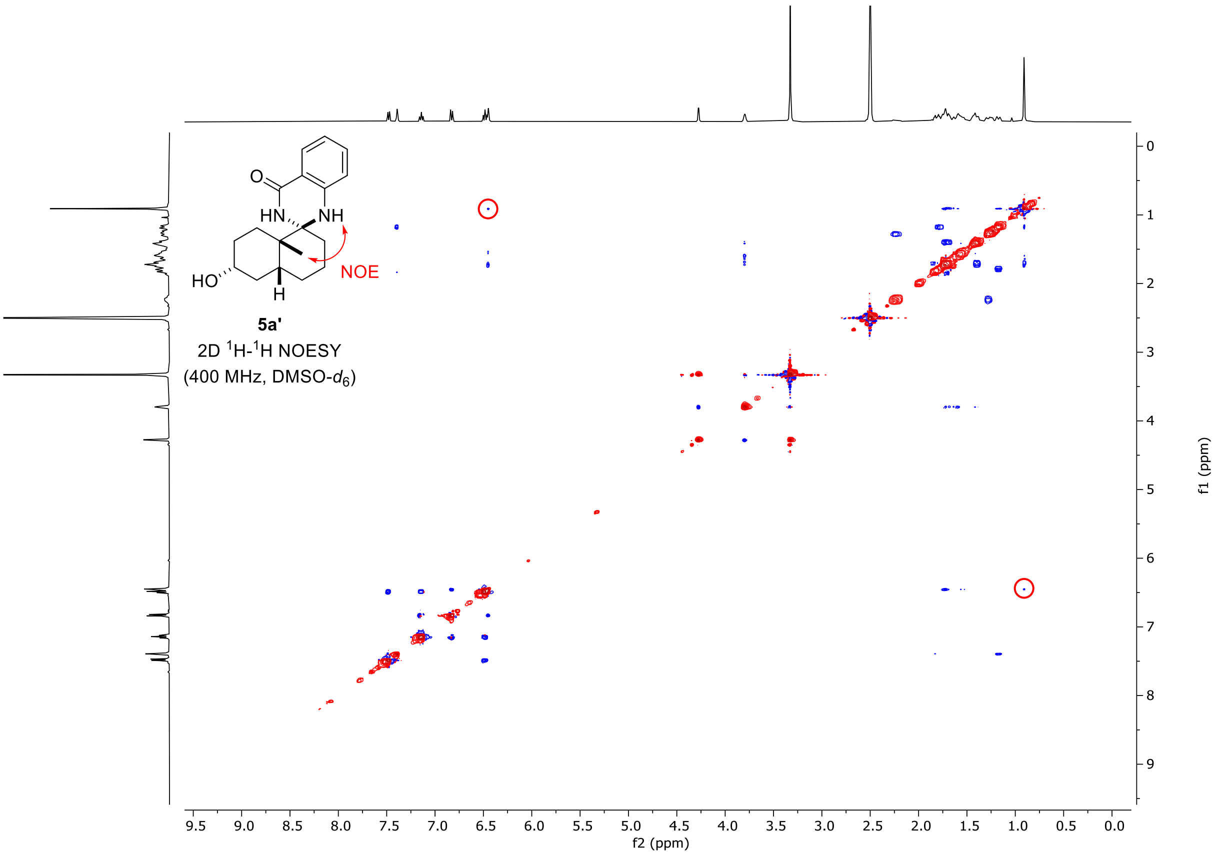
NOE
5a'
2D 1H-1H NOESY
(400 MHz, DMSO-d6)
f2 (ppm)
f1 (ppm)

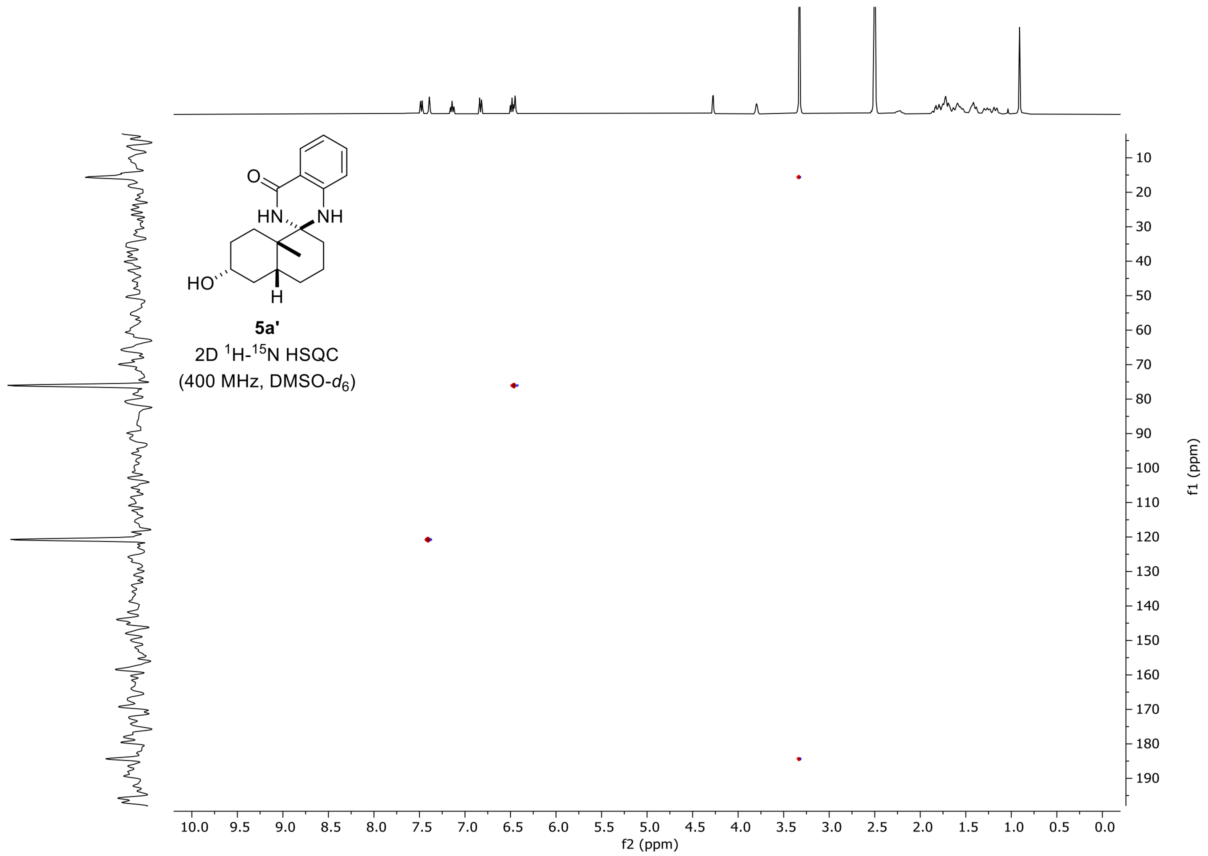
5a'
2D 1H-15N HSQC
(400 MHz, DMSO-d6)
f1 (ppm)
f2 (ppm)

(1*S,4a*R**,6*R**,8a*S**)-7'-Fluoro-6-hydroxy-8a-methyl-3,4,4a,5,6,7,8,8a-octahydro-1'*H*,2*H*-spiro[naphthalene-1,2'-quinazolin]-4'(3'*H*)-one (5b)**

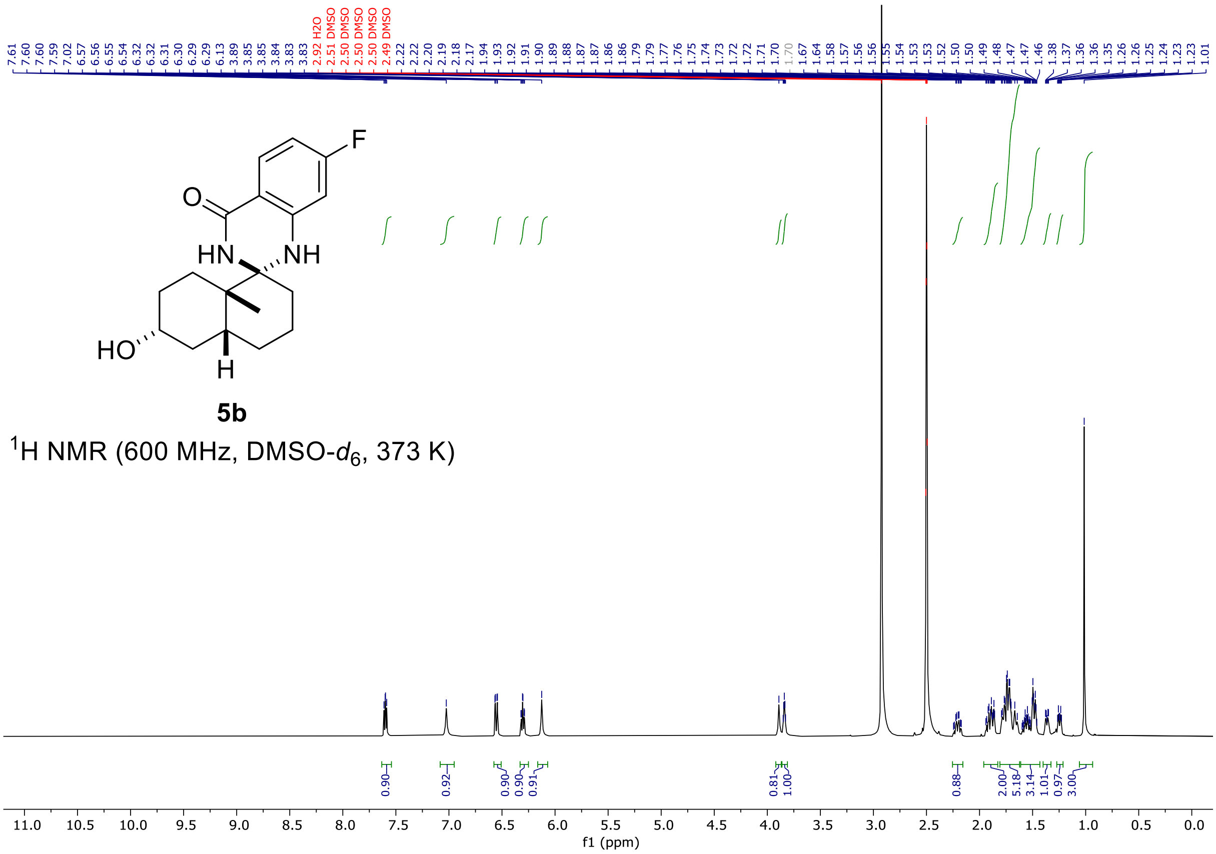

5b

^{1}H NMR (600 MHz, DMSO-d_6, 373 K)

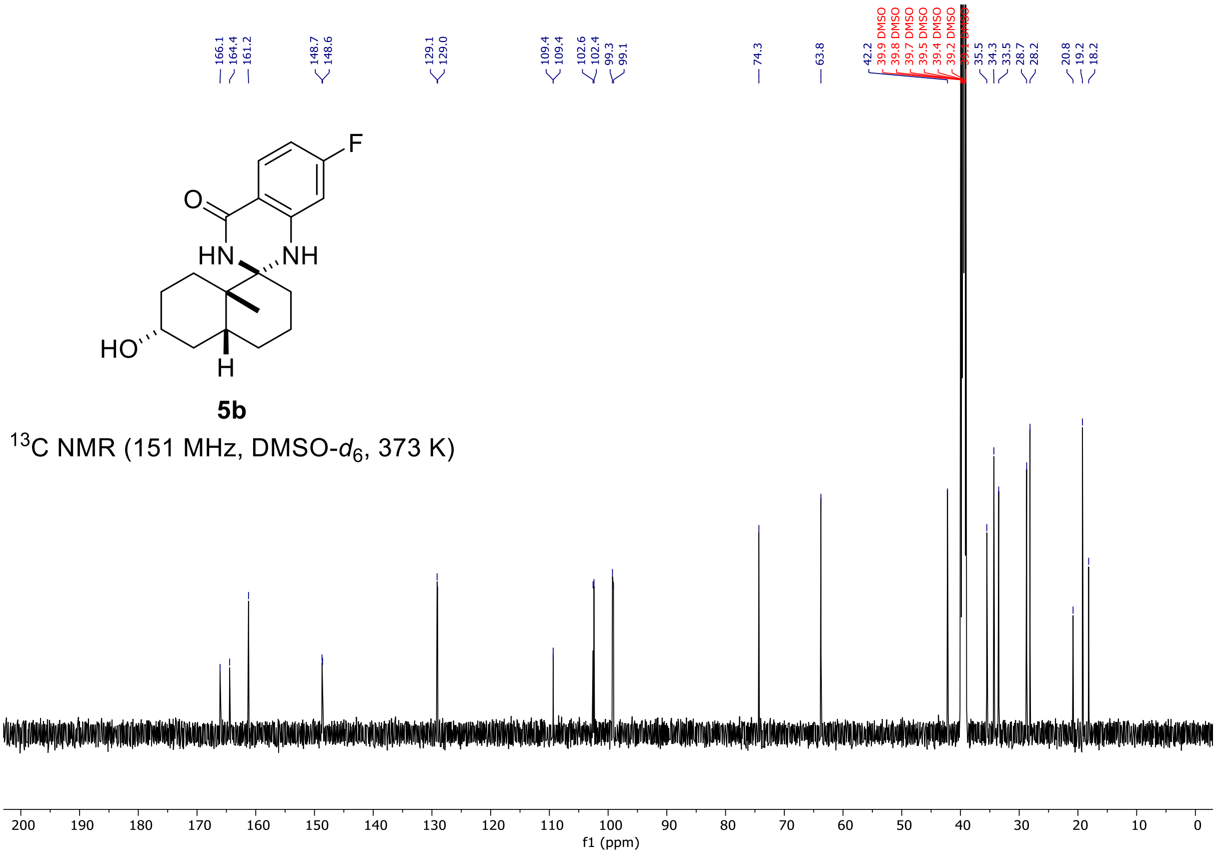

5b

^{13}C NMR (151 MHz, DMSO-d_6, 373 K)

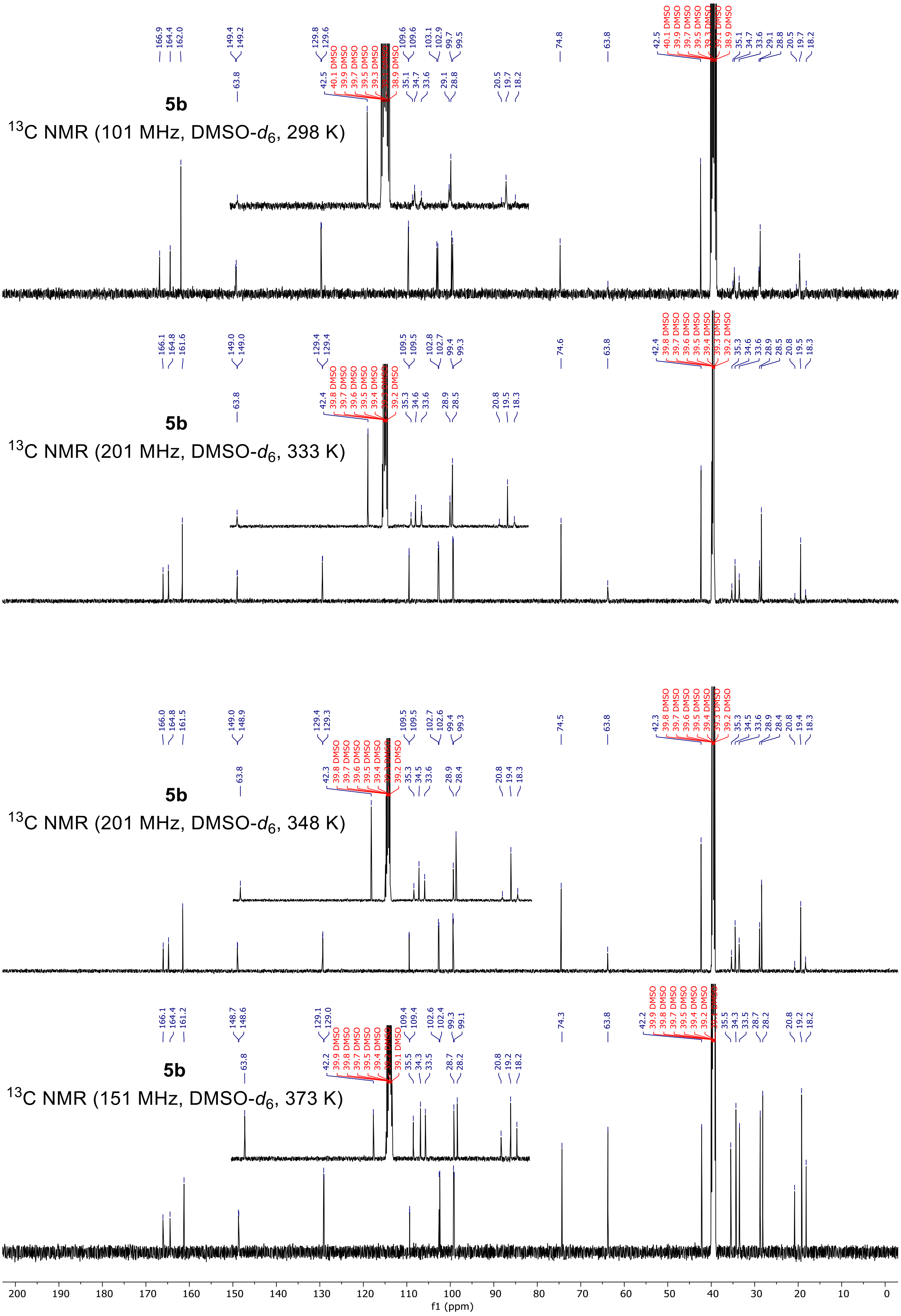
5b
13C NMR (101 MHz, DMSO-d6, 298 K)
5b
13C NMR (201 MHz, DMSO-d6, 333 K)
5b
13C NMR (201 MHz, DMSO-d6, 348 K)
5b
13C NMR (151 MHz, DMSO-d6, 373 K)
200 190 180 170 160 150 140 130 120 110 100 90 80 70 60 50 40 30 20 10 0
f1 (ppm)

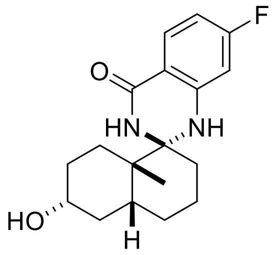

5b

^{19}F NMR (377 MHz, DMSO-d_6)

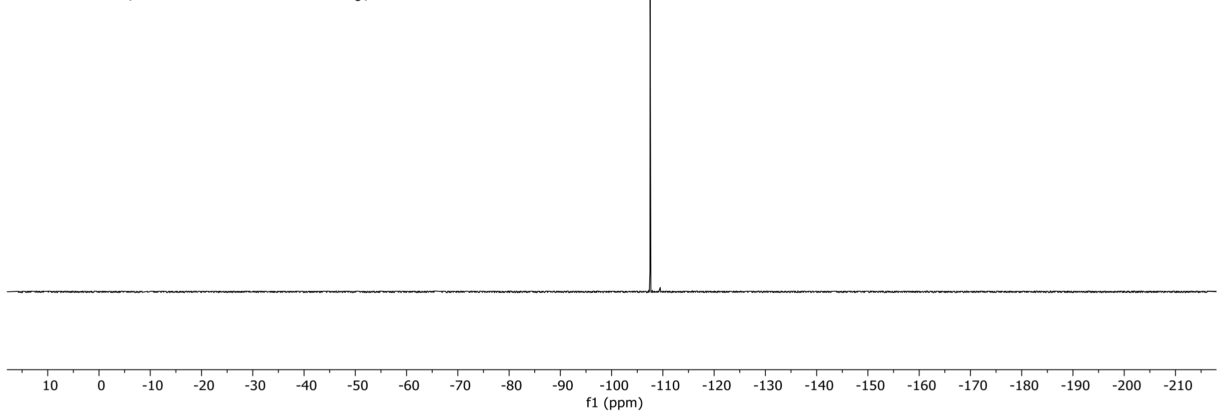

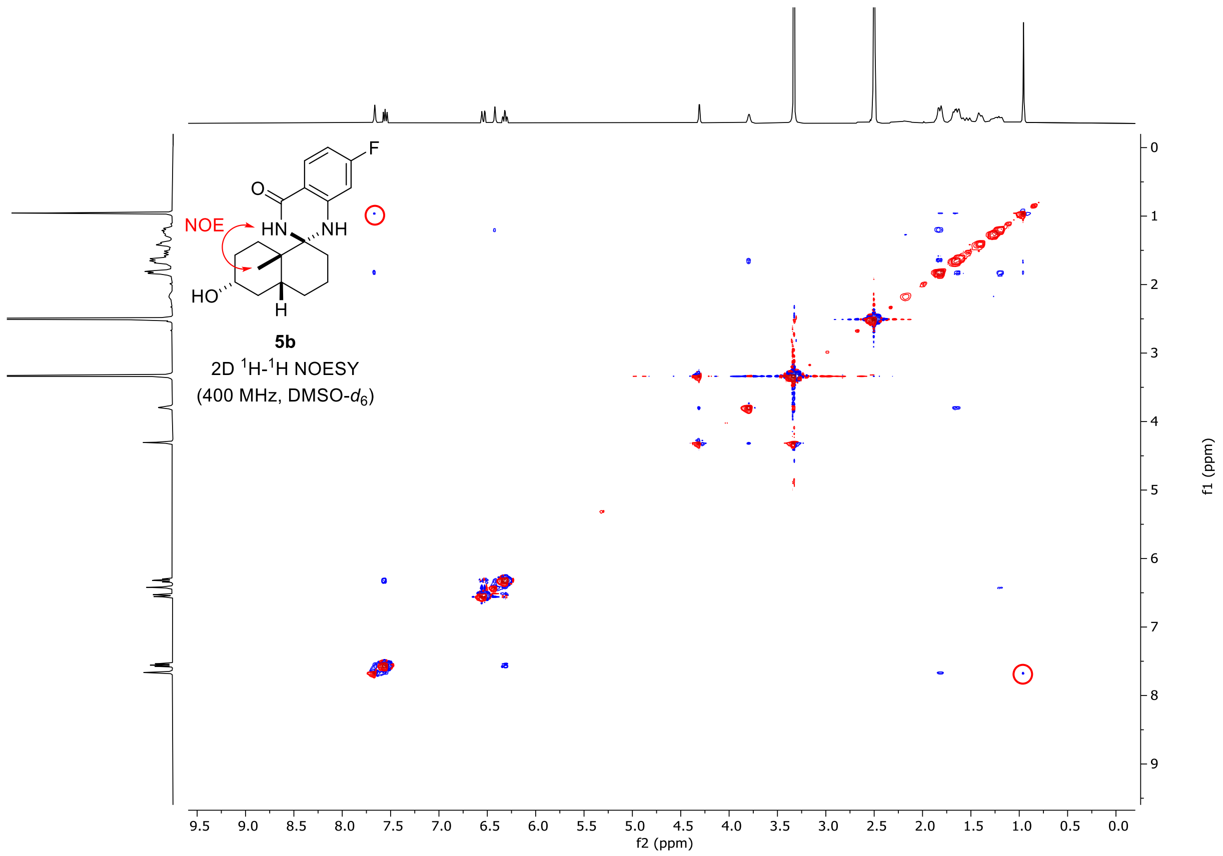

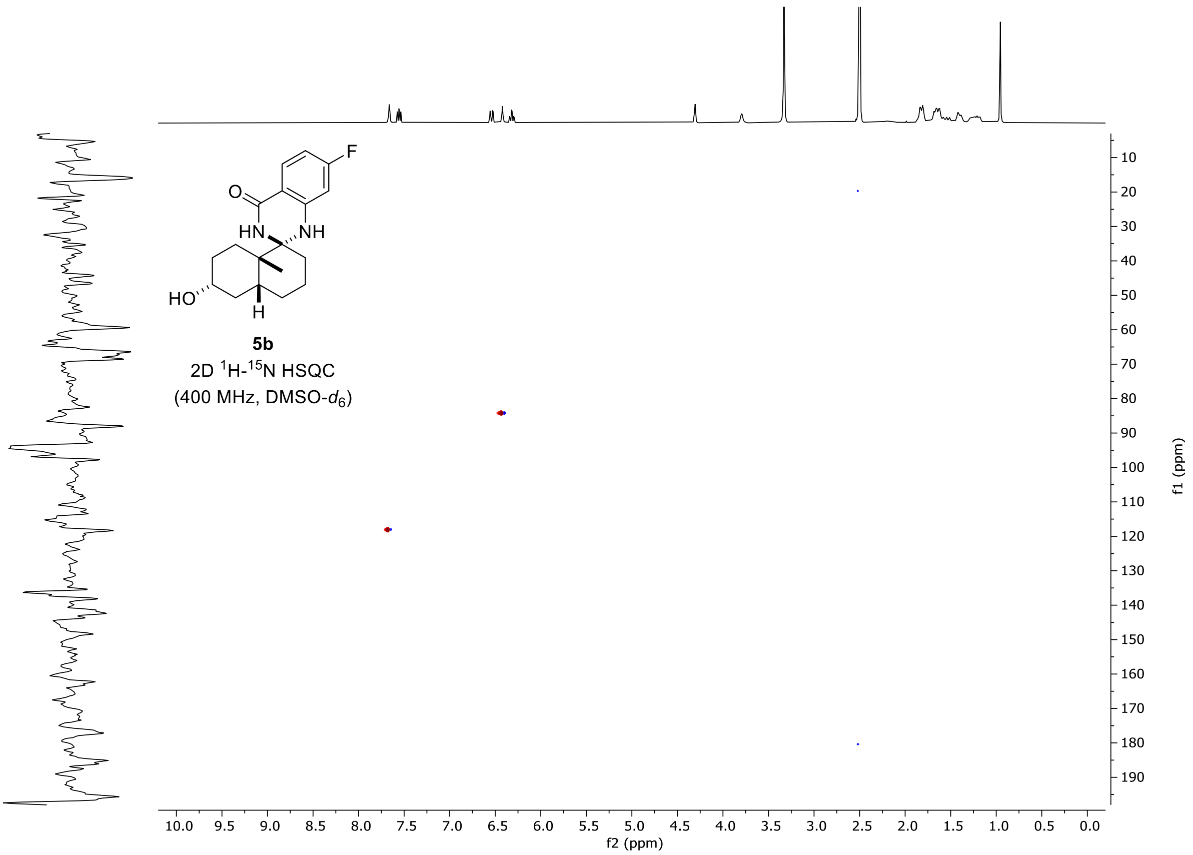
F
O
HN
NH
HO
H
5b
2D 1H-15N HSQC
(400 MHz, DMSO-d6)
f1 (ppm)
f2 (ppm)

(1*R,4a*R**,6*R**,8a*S**)-7'-Fluoro-6-hydroxy-8a-methyl-3,4,4a,5,6,7,8,8a-octahydro-1'*H*,2*H*-spiro[naphthalene-1,2'-quinazolin]-4'(3'*H*)-one (5b')**

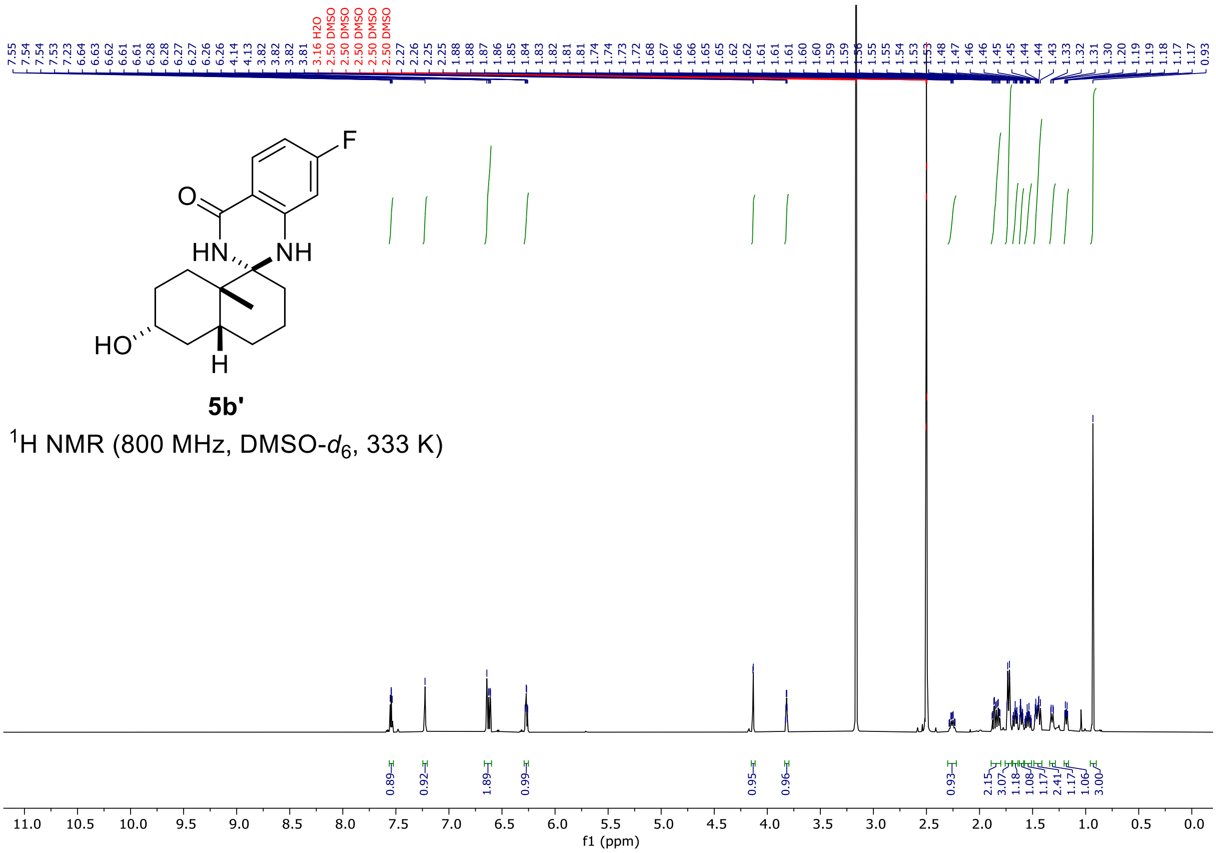

5b'

^{1}H NMR (800 MHz, DMSO-d_6, 333 K)

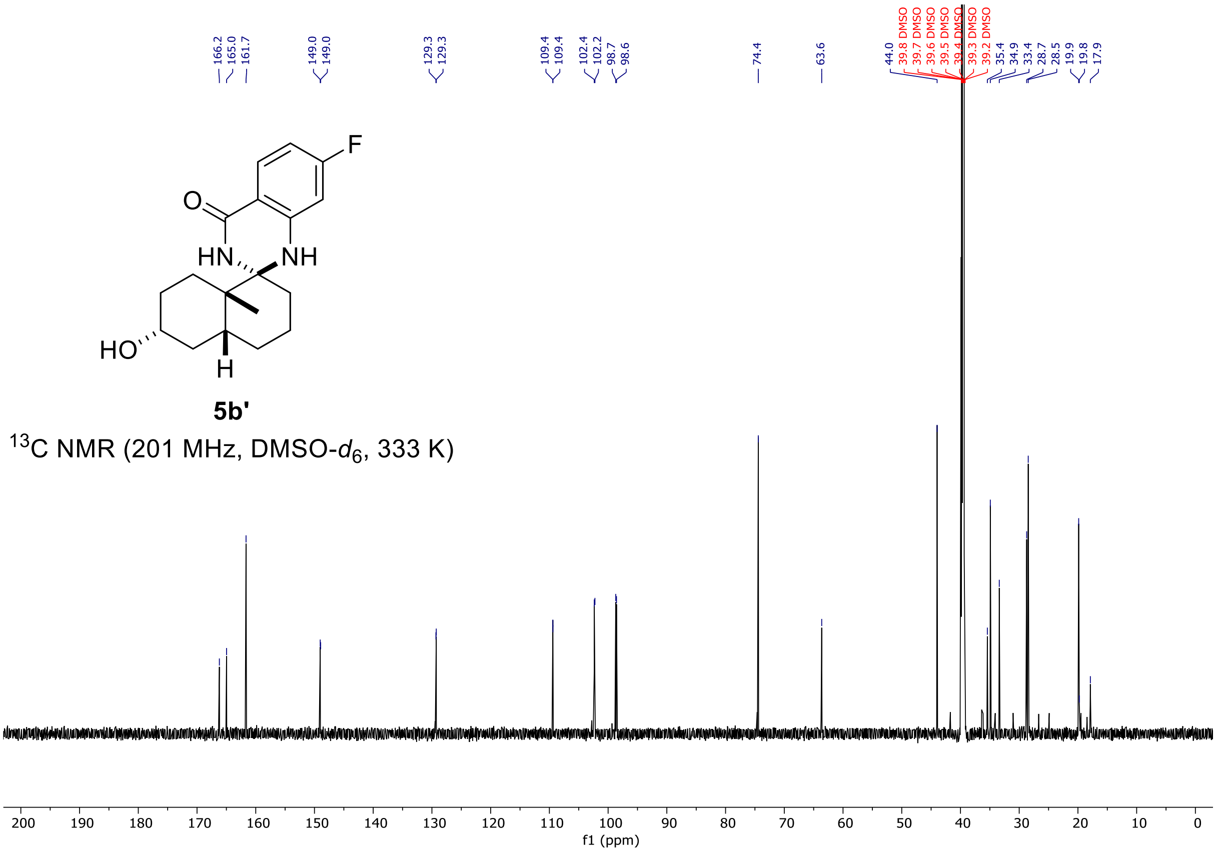

5b'

^{13}C NMR (201 MHz, DMSO-d_6, 333 K)

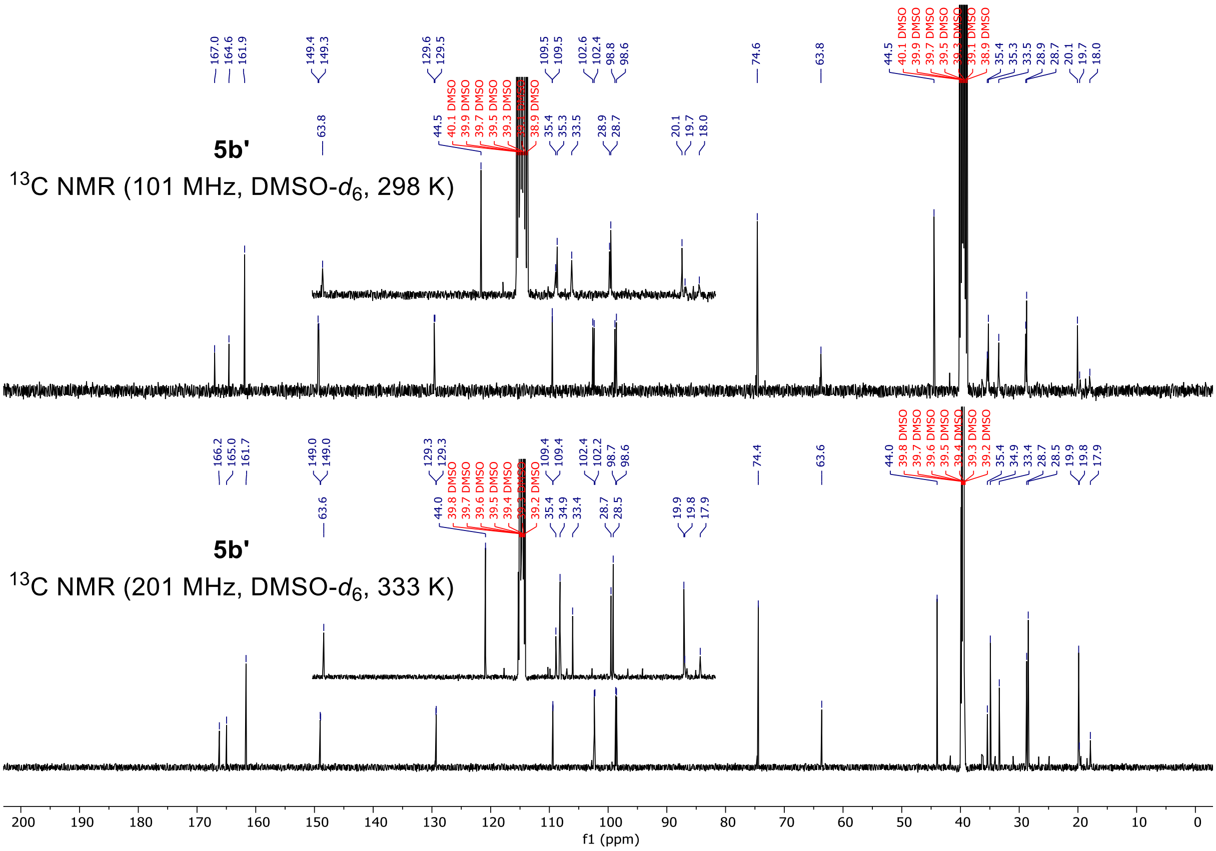

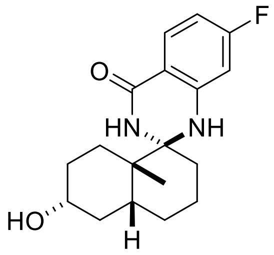

5b'

^{19}F NMR (377 MHz, DMSO-d_6)

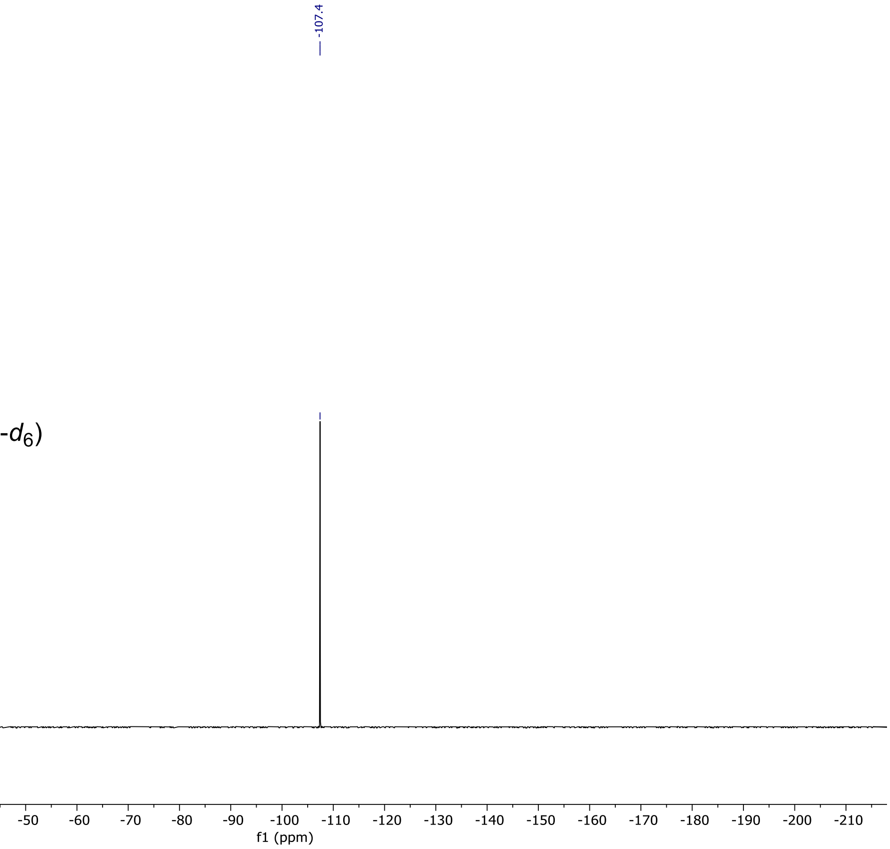

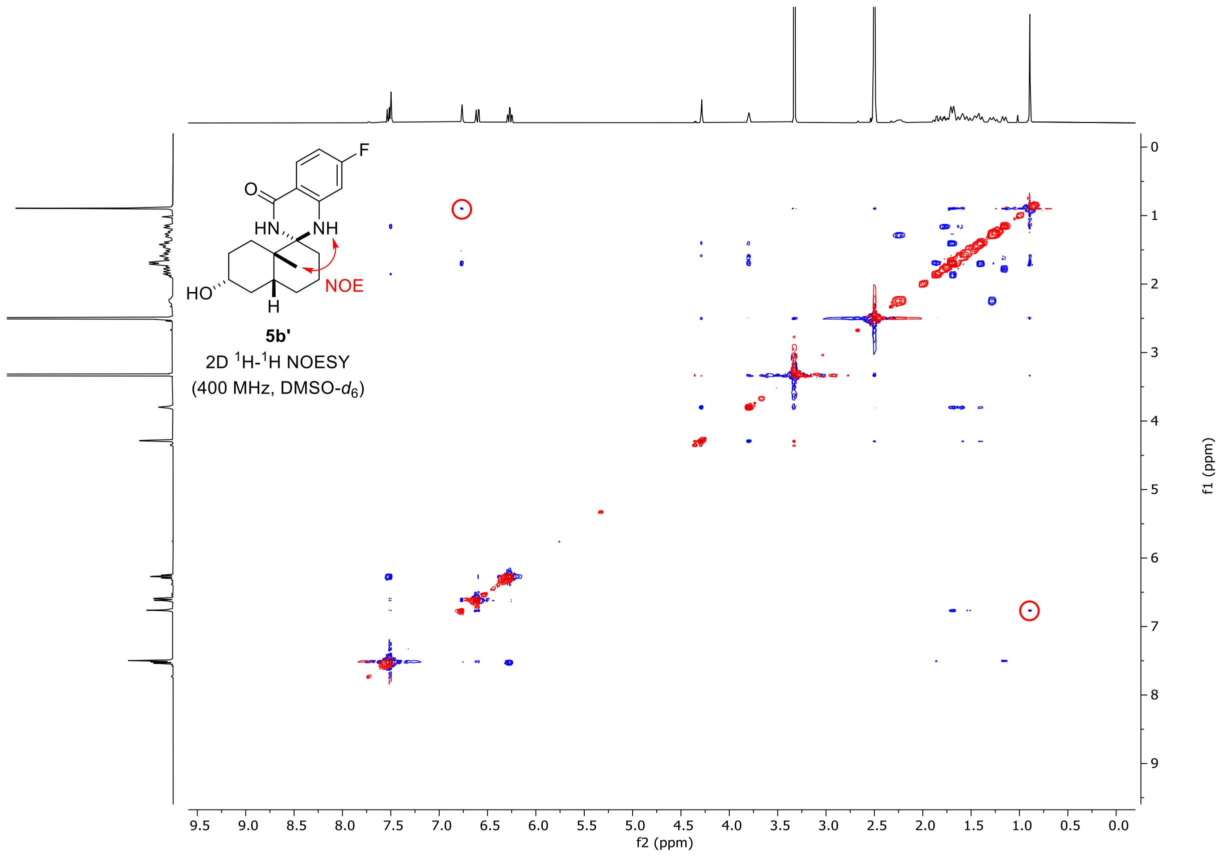
F
O
HN
NH
HO
H
NOE
5b'
2D ¹H-¹H NOESY
(400 MHz, DMSO-d6)
f1 (ppm)
f2 (ppm)

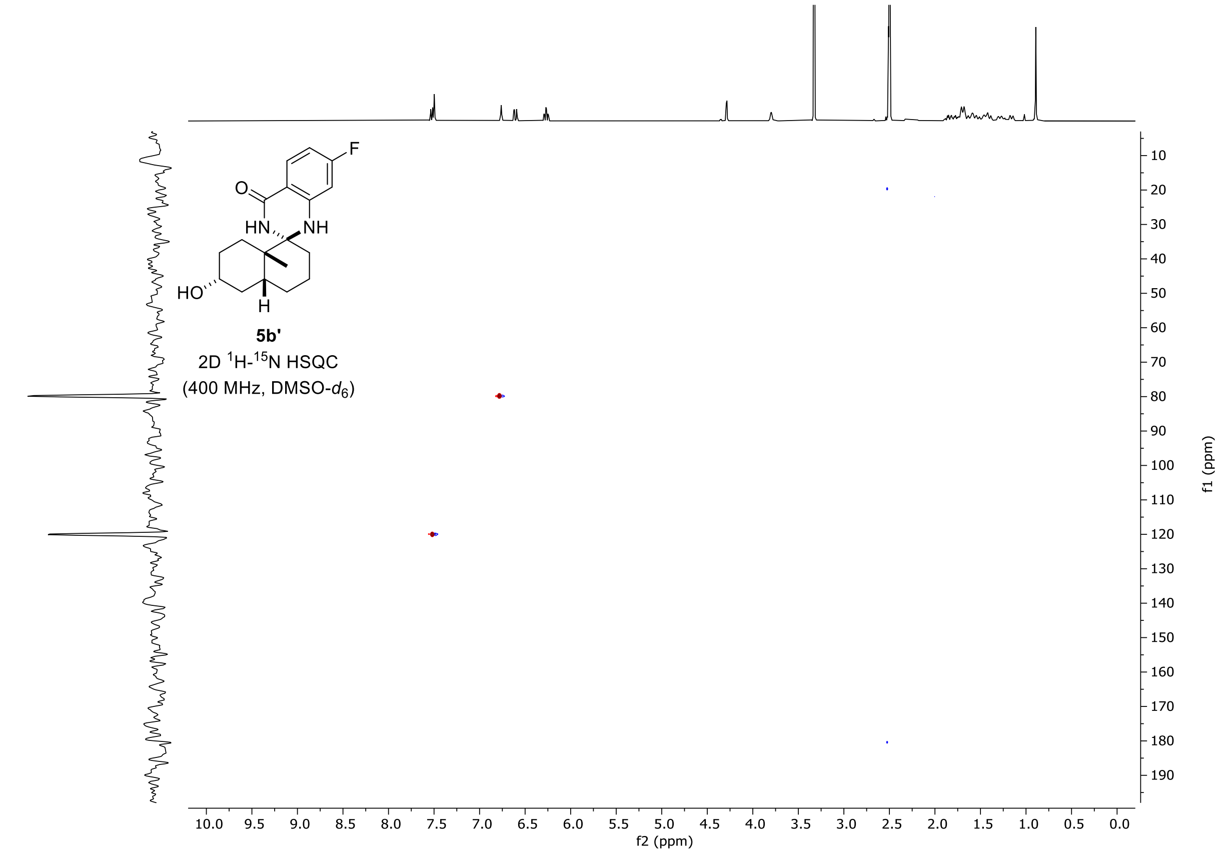
F
O
HN
NH
HO
H
5b'
2D ¹H-¹⁵N HSQC
(400 MHz, DMSO-d6)
f1 (ppm)
f2 (ppm)

(1*S,4a*R**,6*R**,8a*S**)-6-Hydroxy-6'-methoxy-8a-methyl-3,4,4a,5,6,7,8,8a-octahydro-1'*H*,2*H*-spiro[naphthalene-1,2'-quinazolin]-4'(3'*H*)-one (5c)**

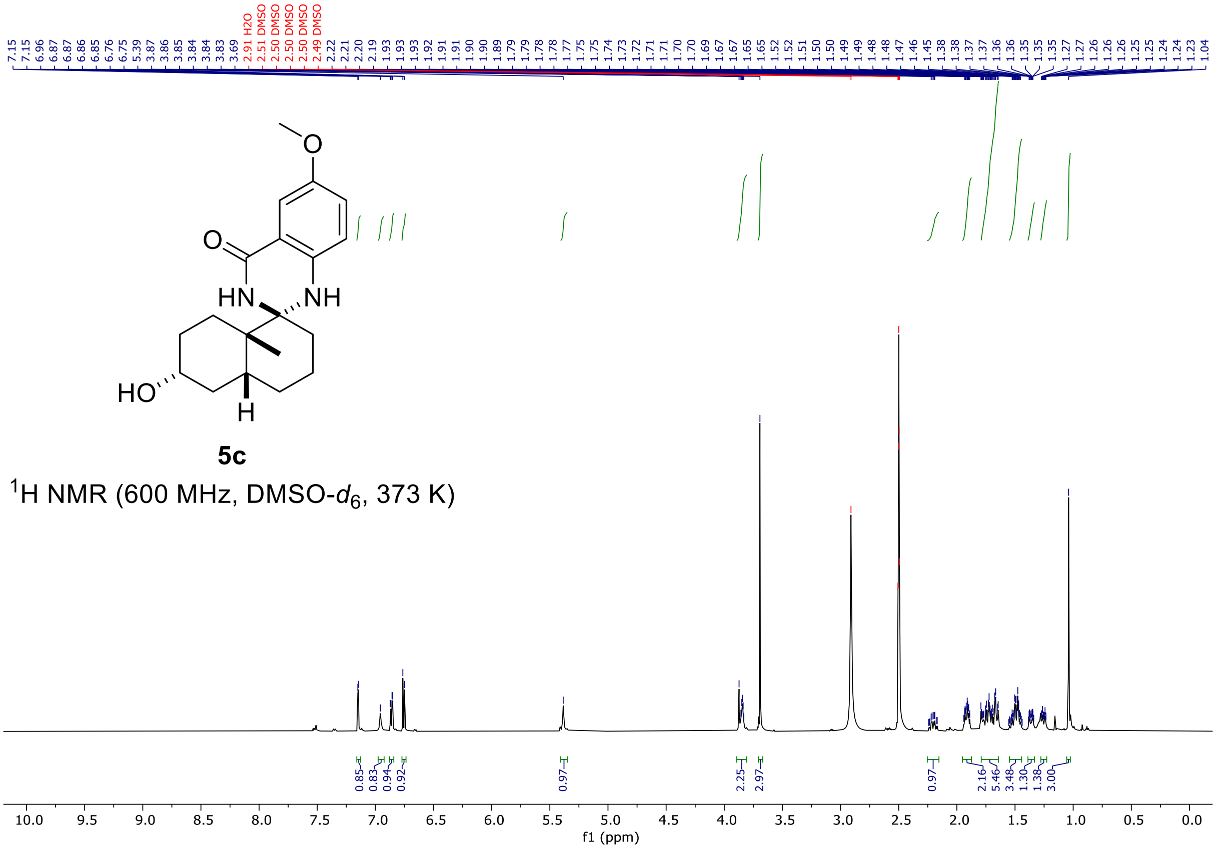

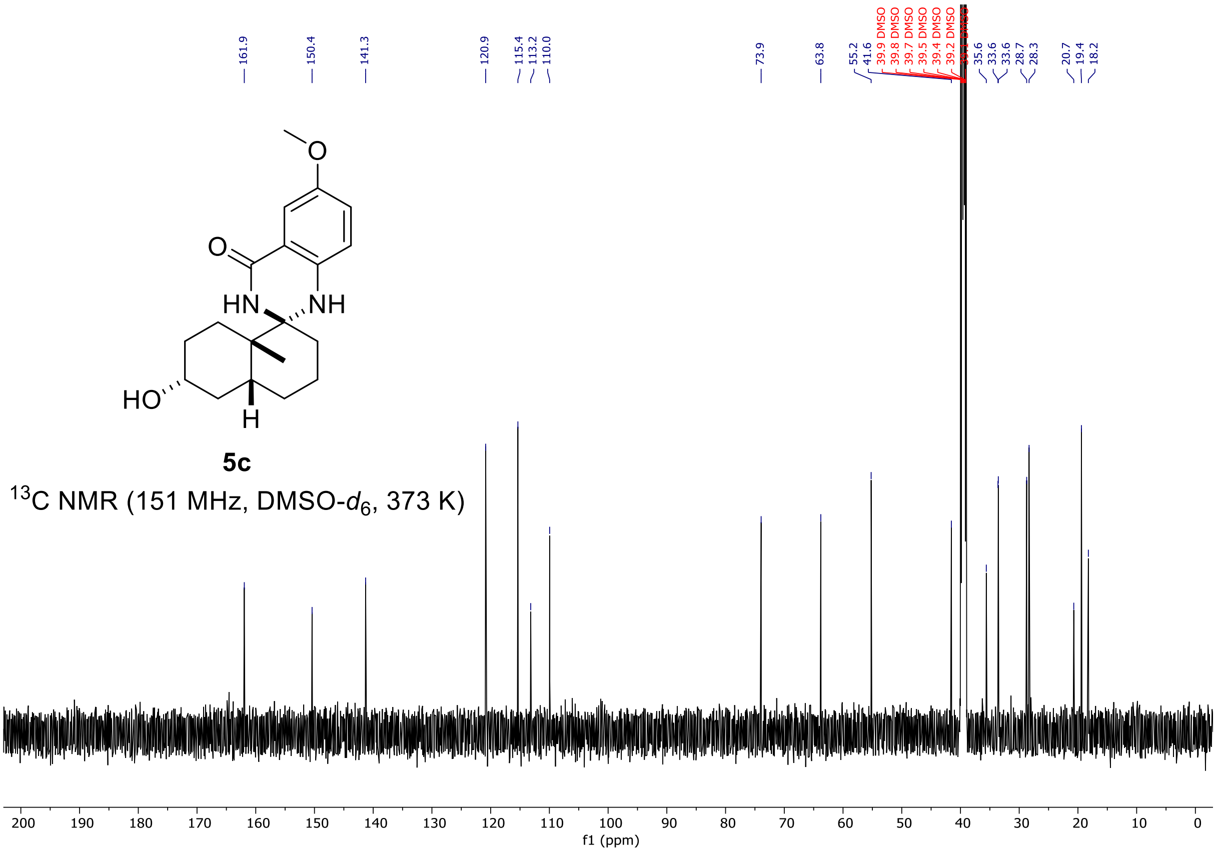

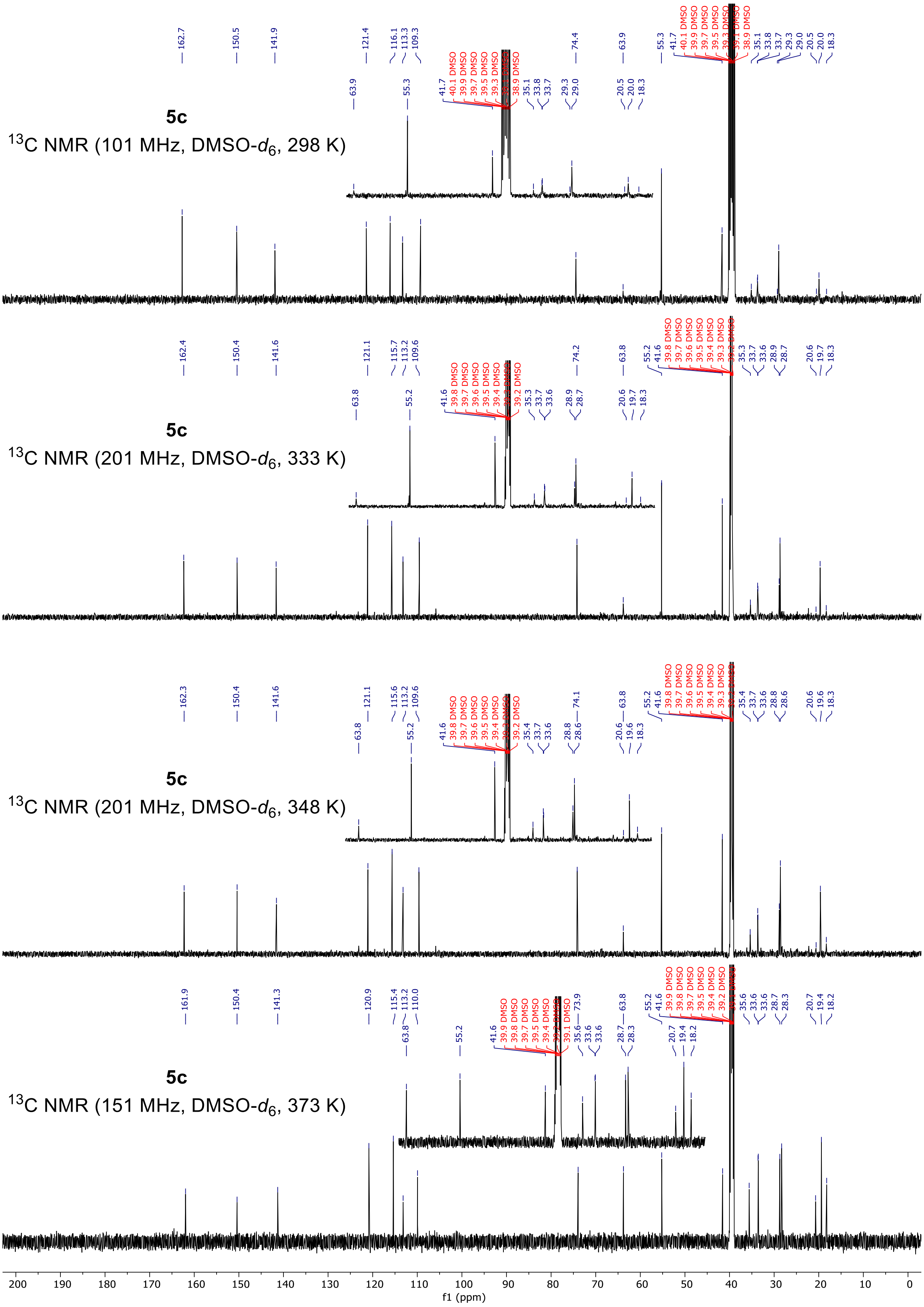
5c
13C NMR (101 MHz, DMSO-d6, 298 K)
5c
13C NMR (201 MHz, DMSO-d6, 333 K)
5c
13C NMR (201 MHz, DMSO-d6, 348 K)
5c
13C NMR (151 MHz, DMSO-d6, 373 K)
f1 (ppm)

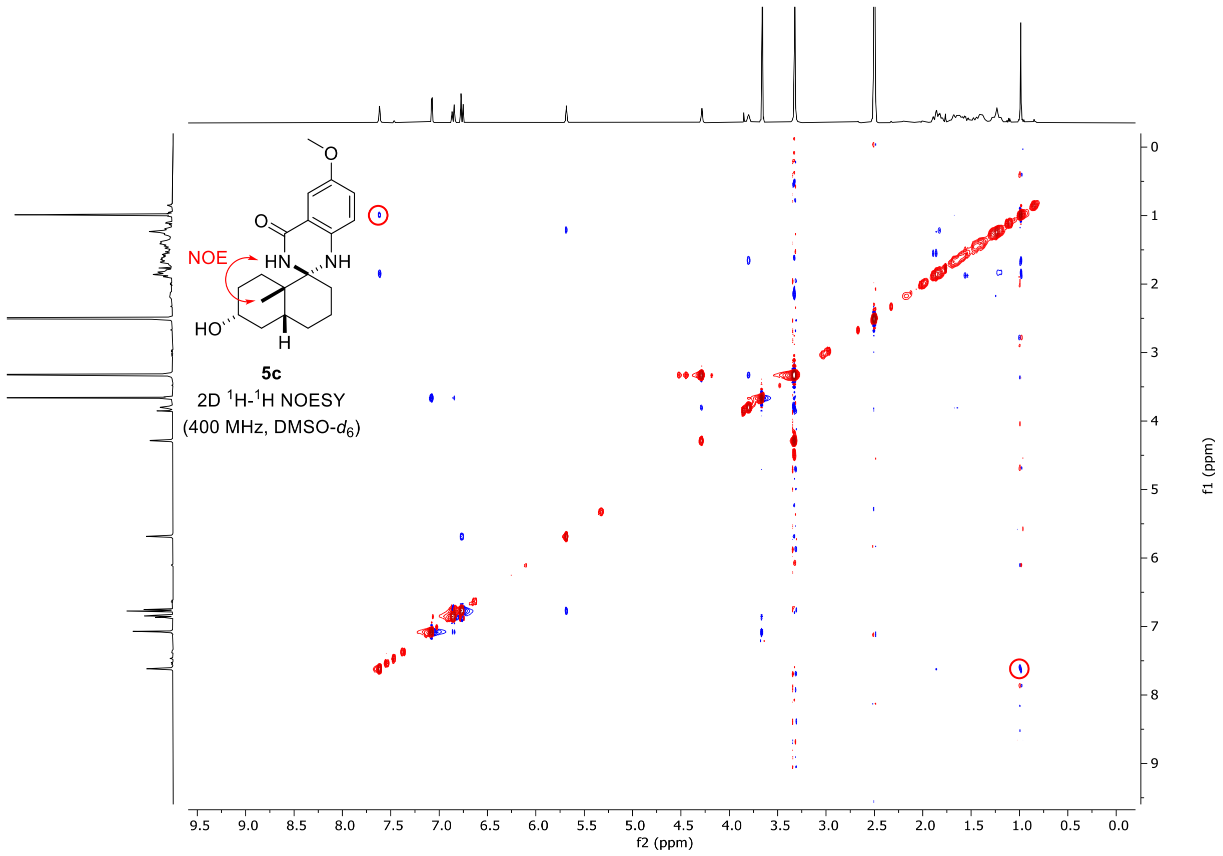
NOE
HN
NH
HO
H
O
5c
2D ¹H-¹H NOESY
(400 MHz, DMSO-d6)
f2 (ppm)
f1 (ppm)

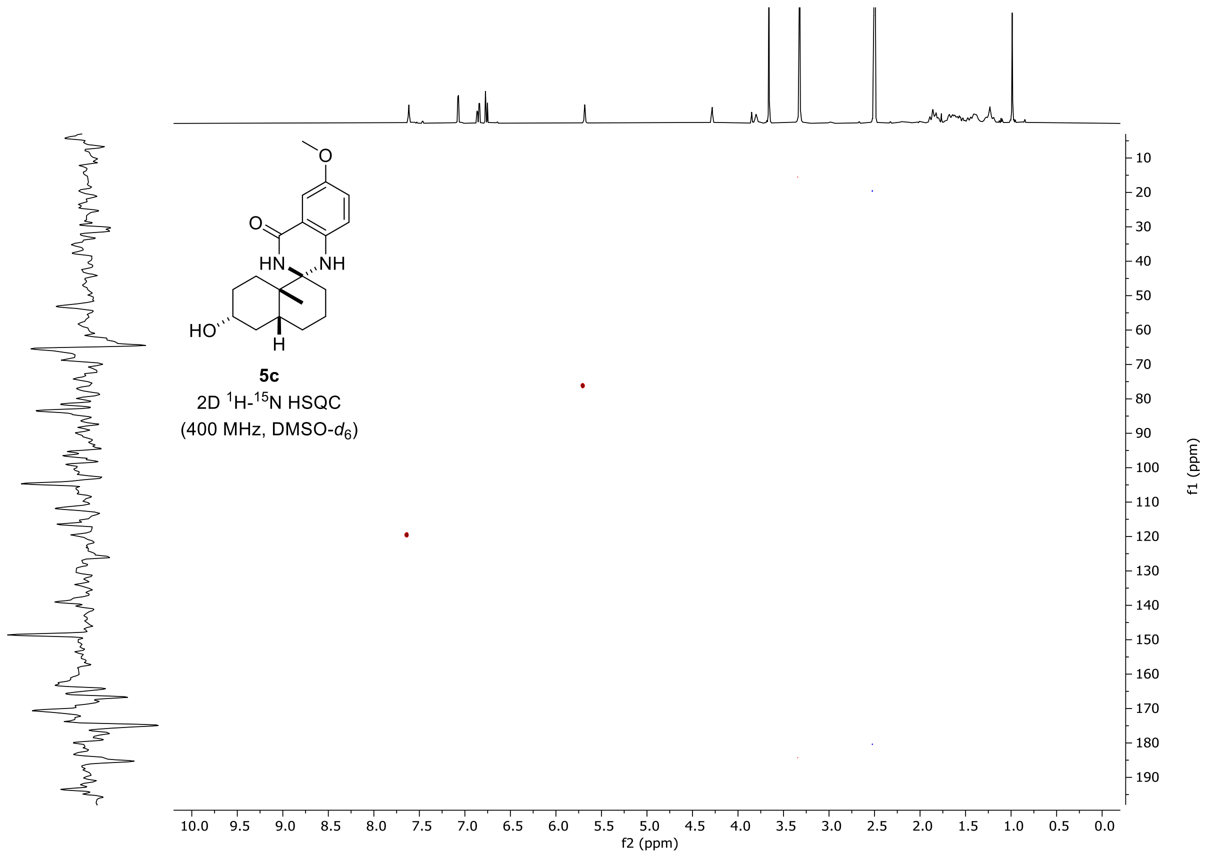
HN
NH
HO
H
O
5c
2D ¹H-¹⁵N HSQC
(400 MHz, DMSO-d6)
f2 (ppm)
f1 (ppm)

(1*R,4a*R**,6*R**,8a*S**)-6-Hydroxy-6'-methoxy-8a-methyl-3,4,4a,5,6,7,8,8a-octahydro-1'*H*,2*H*-spiro[naphthalene-1,2'-quinazolin]-4'(3'*H*)-one (5c')**

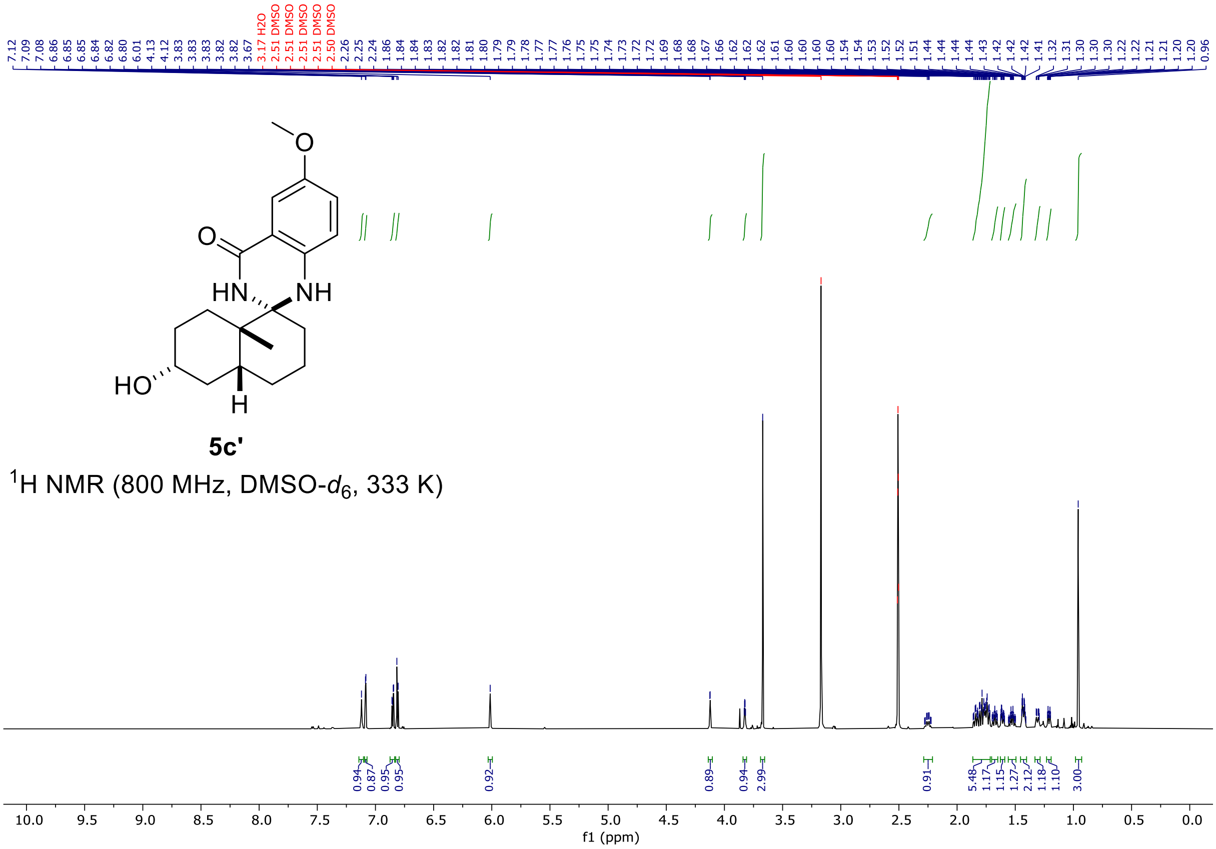

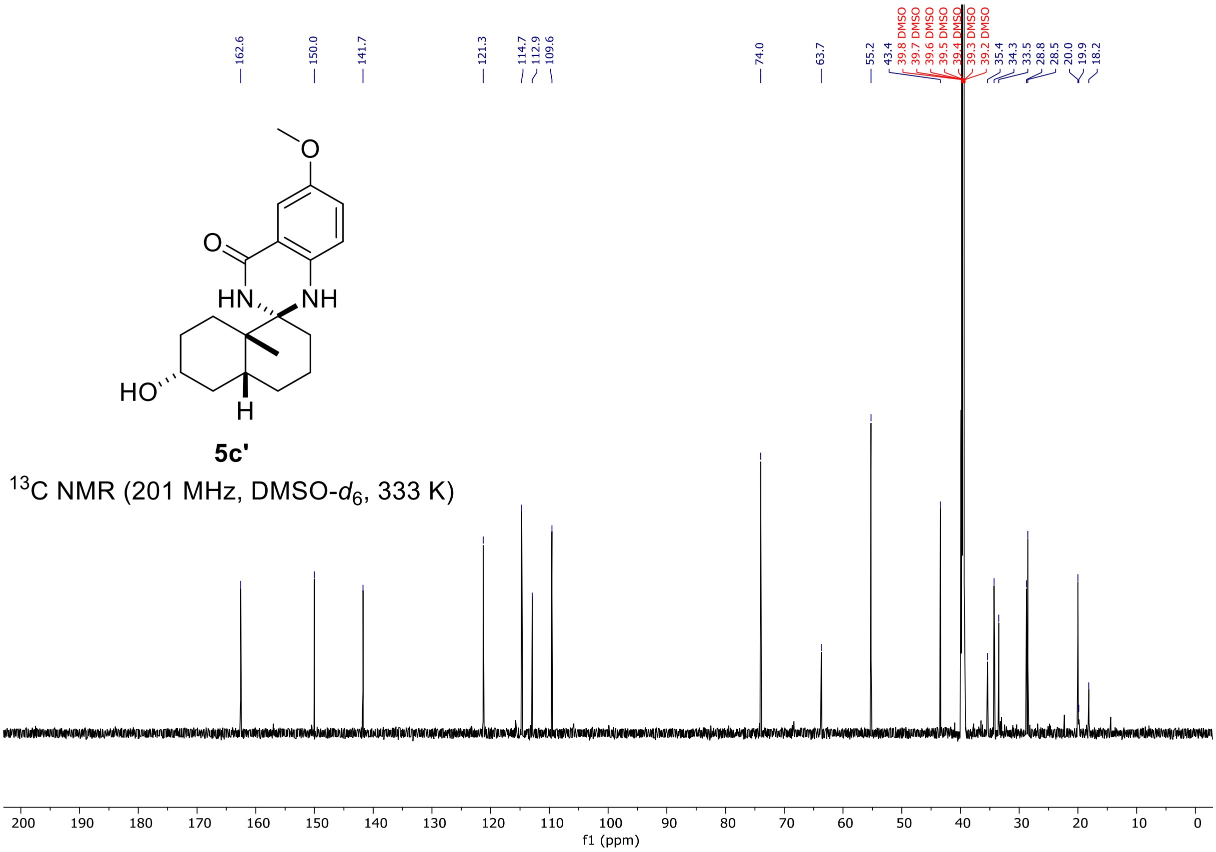

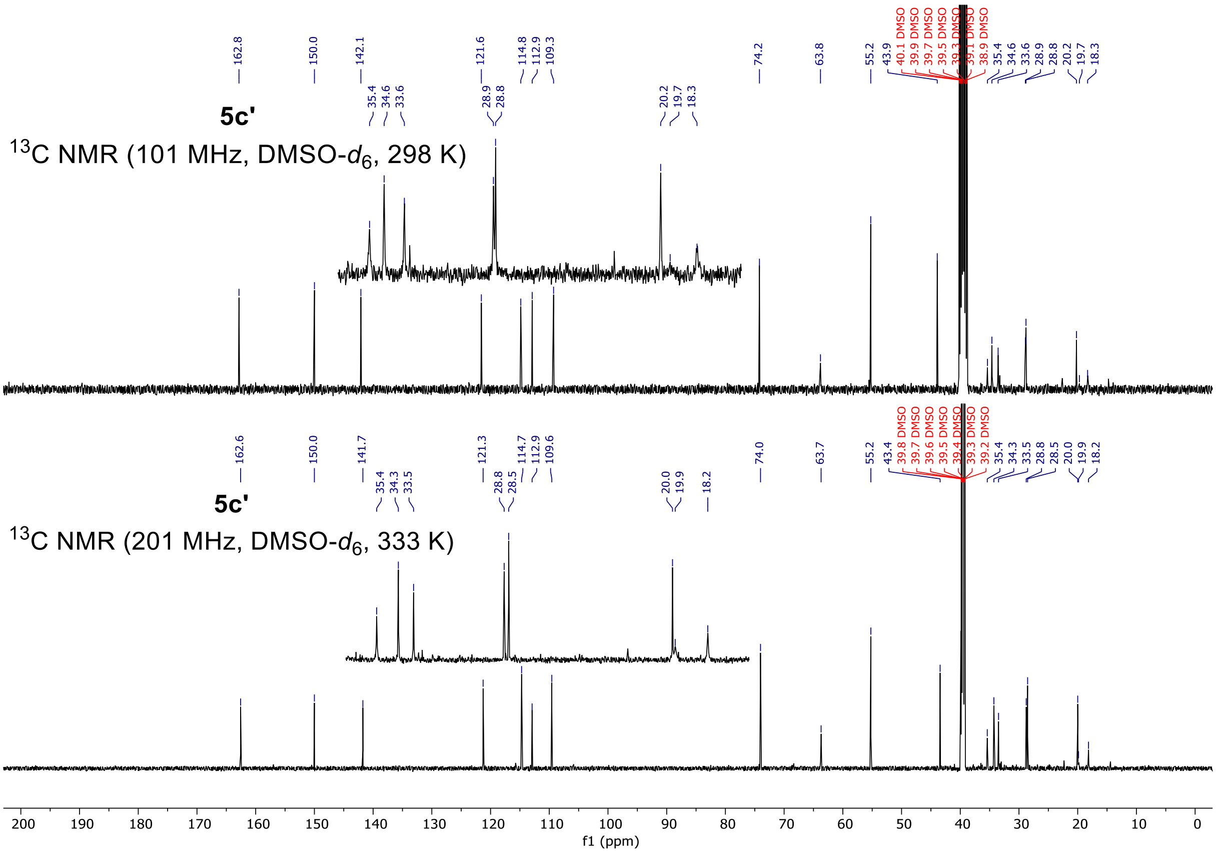
5c'
13C NMR (101 MHz, DMSO-d6, 298 K)
5c'
13C NMR (201 MHz, DMSO-d6, 333 K)
f1 (ppm)

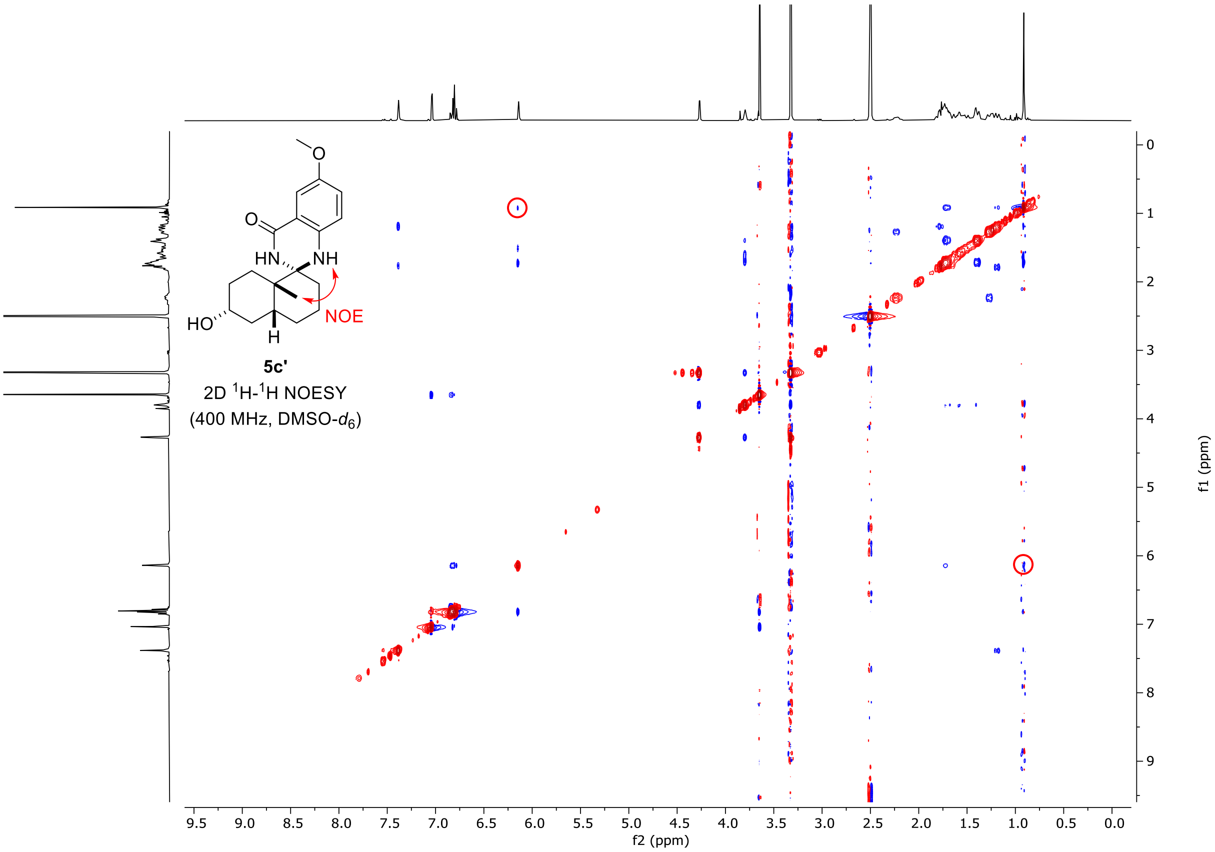
HN
NH
O
HO
H
NOE
5c'
2D 1H-1H NOESY
(400 MHz, DMSO-d6)
f2 (ppm)
f1 (ppm)

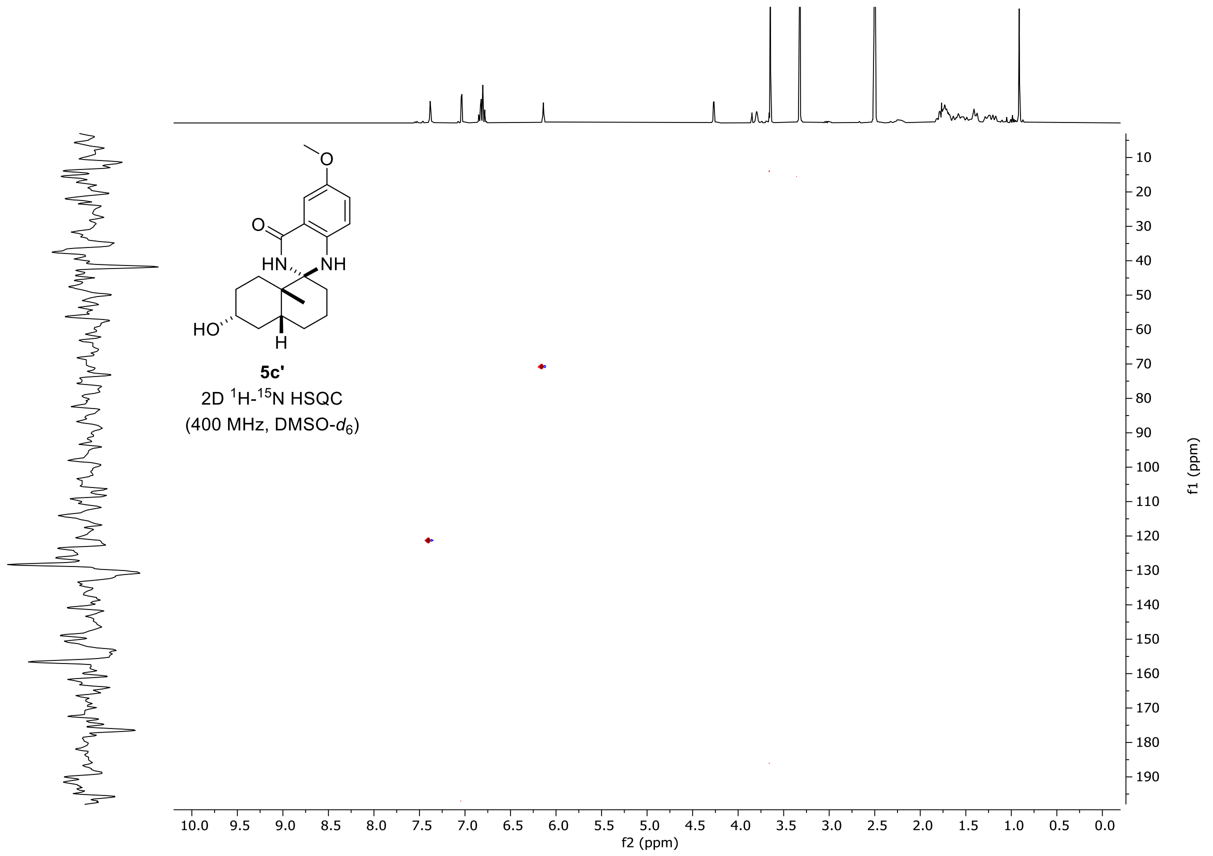
HO
H
HN
NH
O
O
5c'
2D ¹H-¹⁵N HSQC
(400 MHz, DMSO-d6)
f1 (ppm)
f2 (ppm)
10.0 9.5 9.0 8.5 8.0 7.5 7.0 6.5 6.0 5.5 5.0 4.5 4.0 3.5 3.0 2.5 2.0 1.5 1.0 0.5 0.0
10 20 30 40 50 60 70 80 90 100 110 120 130 140 150 160 170 180 190

(3*R,4a*R**,12b*S**)-12b-Methyl-1,2,3,4,4a,5,6,12b-octahydrobenzo[*a*]phenazin-3-ol (7a)**

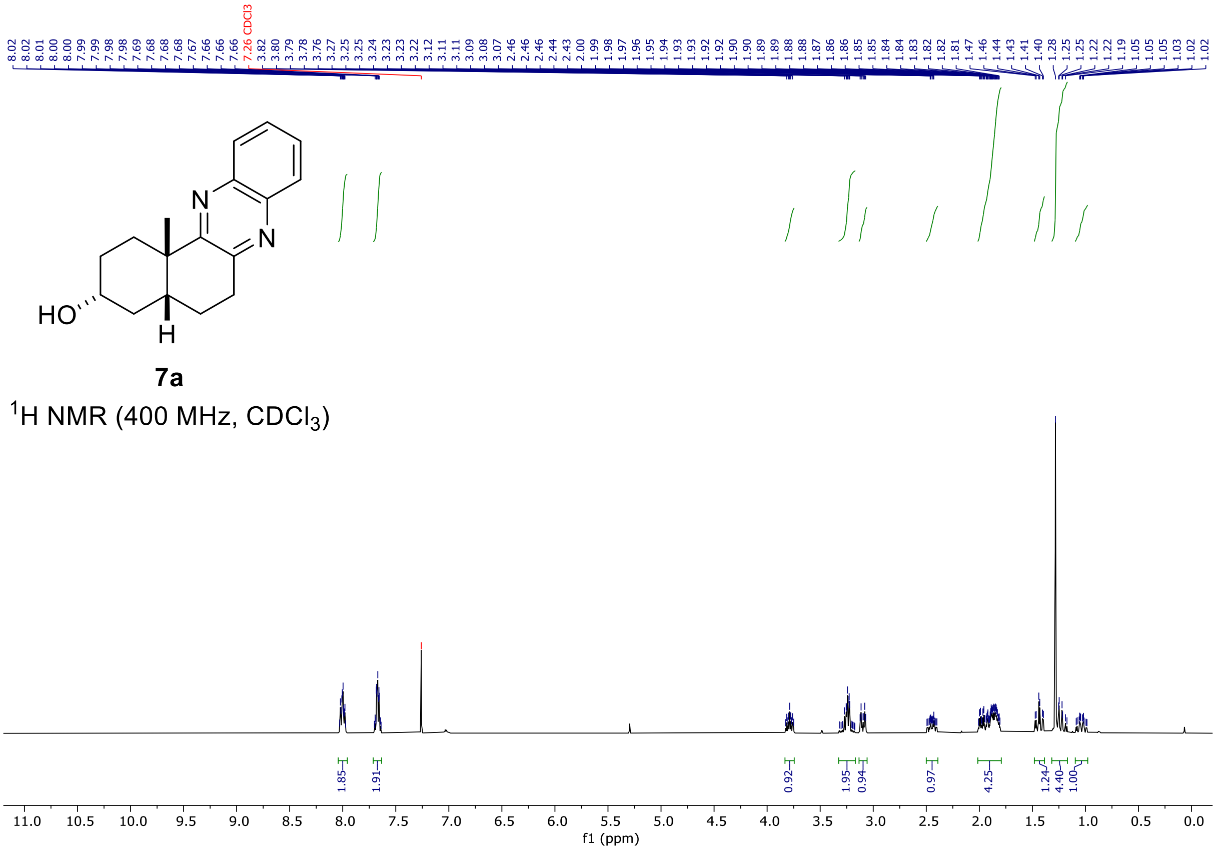

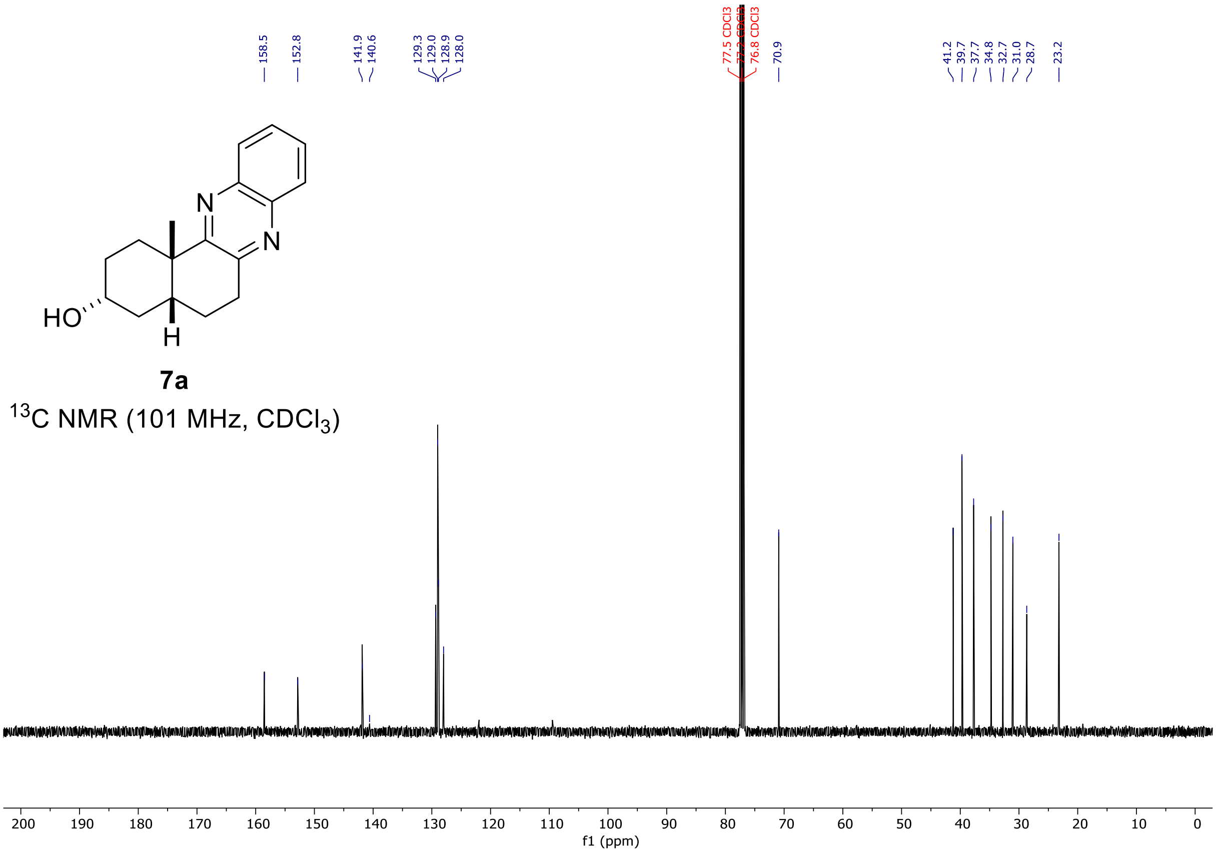

(3*R,4a*R**,12b*S**)-9,10,12b-Trimethyl-1,2,3,4,4a,5,6,12b-octahydrobenzo[*a*]phenazin-3-ol (7b)**

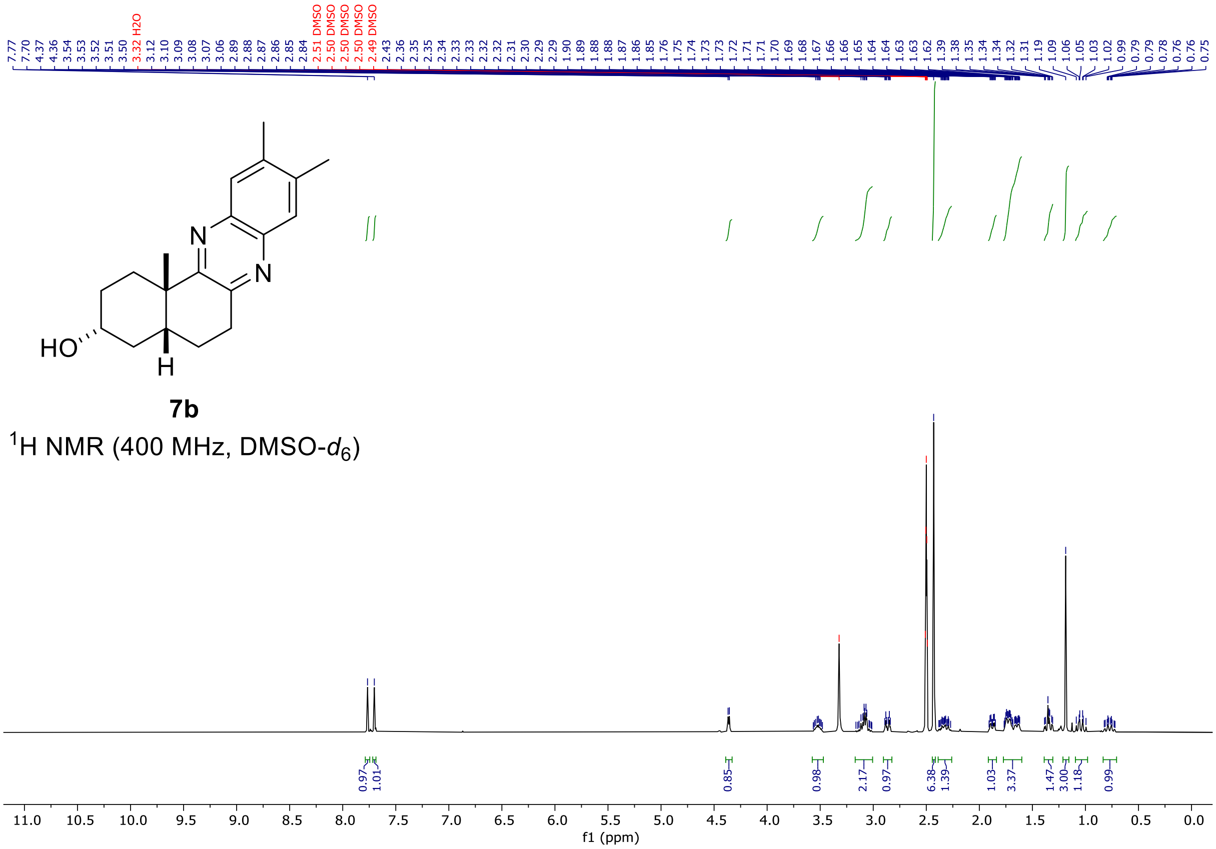

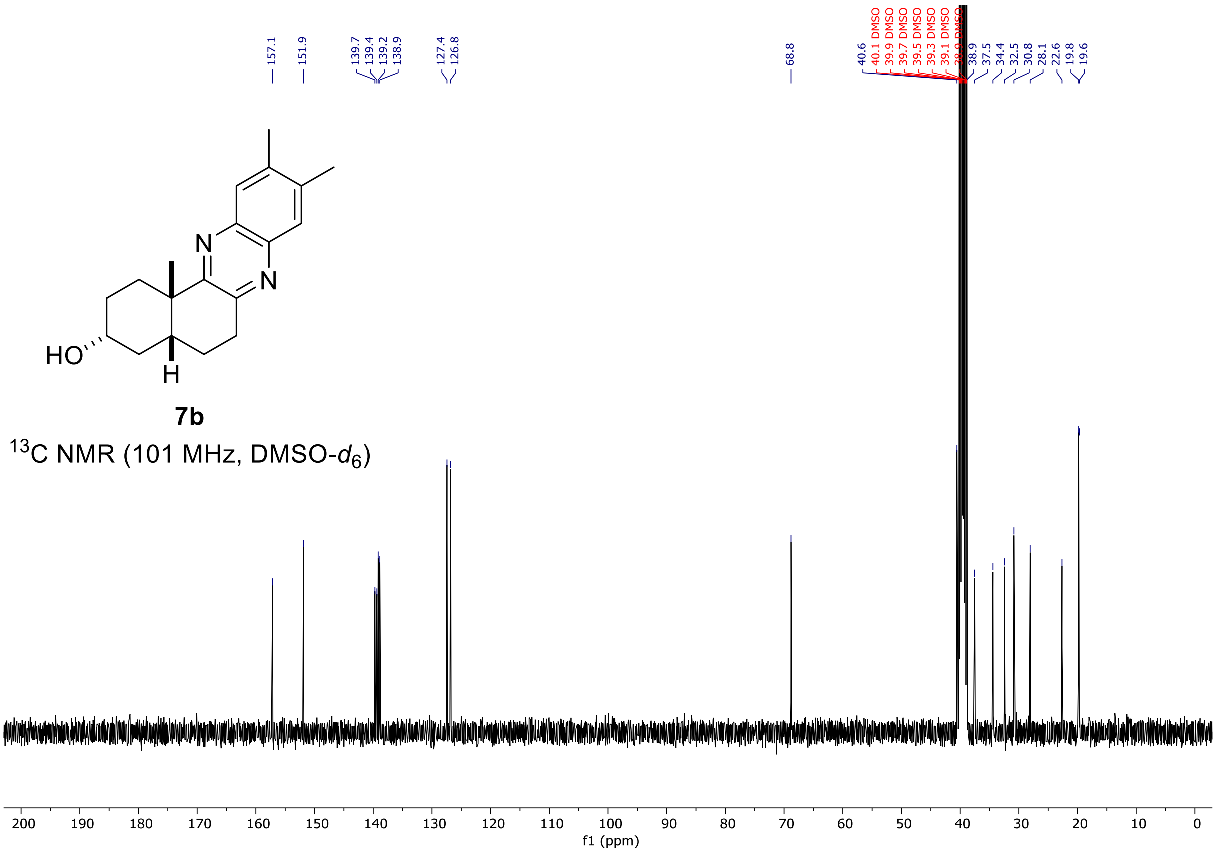

(3*R,4a*R**,12b*S**)-9,10-Difluoro-12b-methyl-1,2,3,4,4a,5,6,12b-octahydrobenzo[*a*]phenazin-3-ol (7c)**

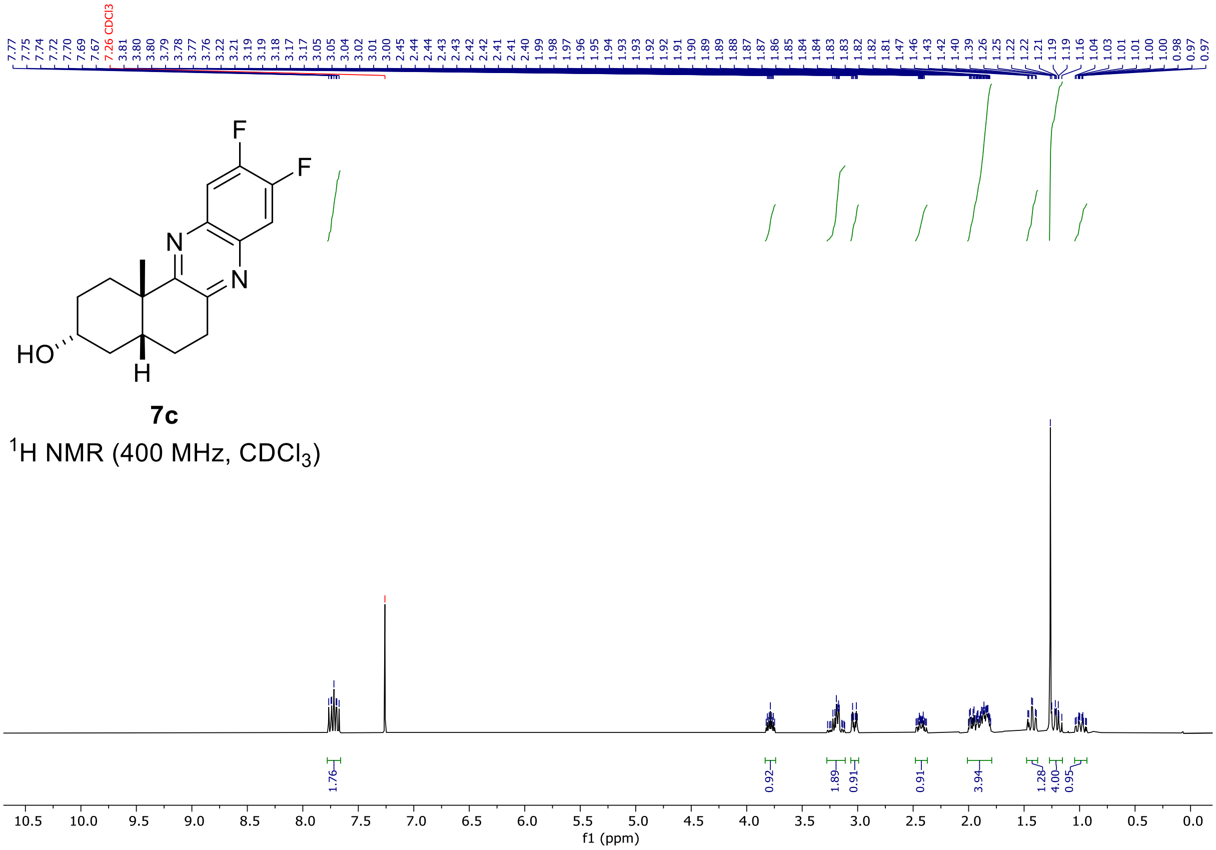

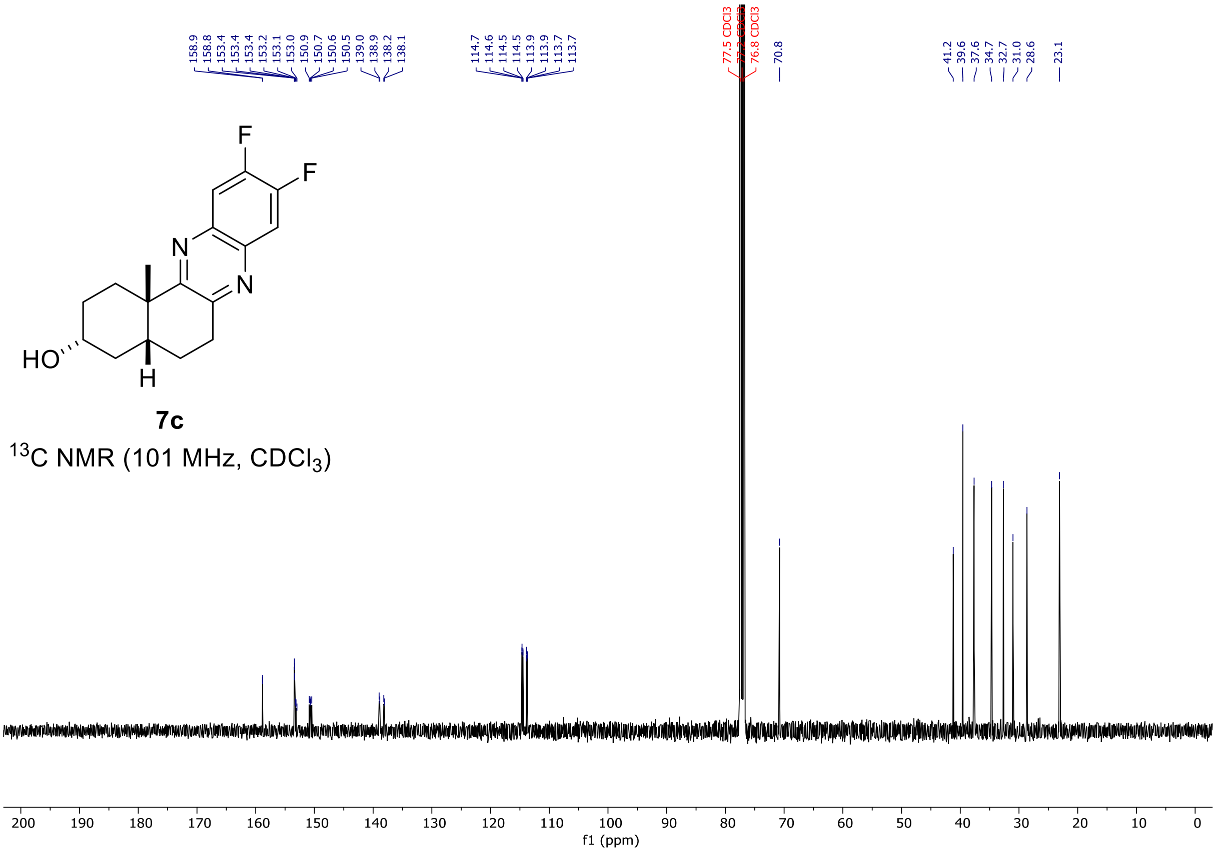

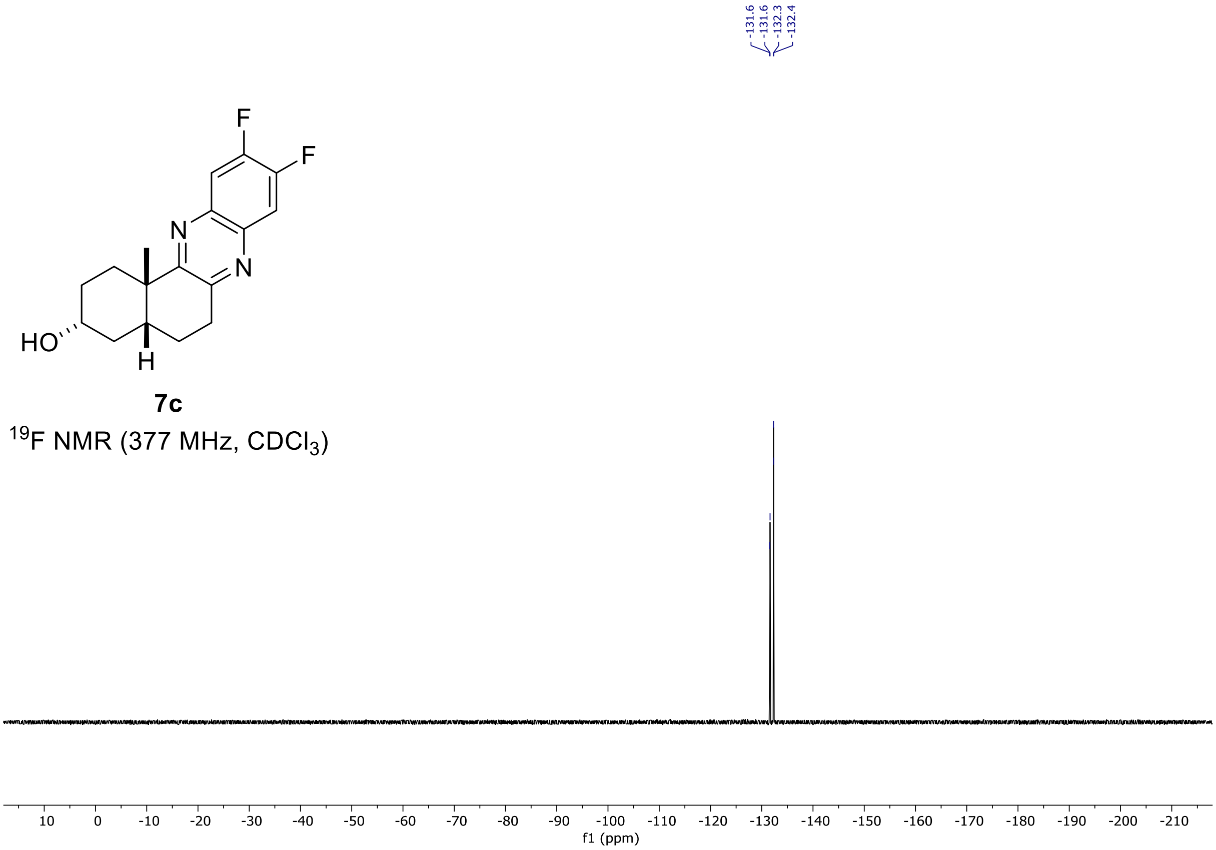

7c

^{19}F NMR (377 MHz, $CDCl_3$)

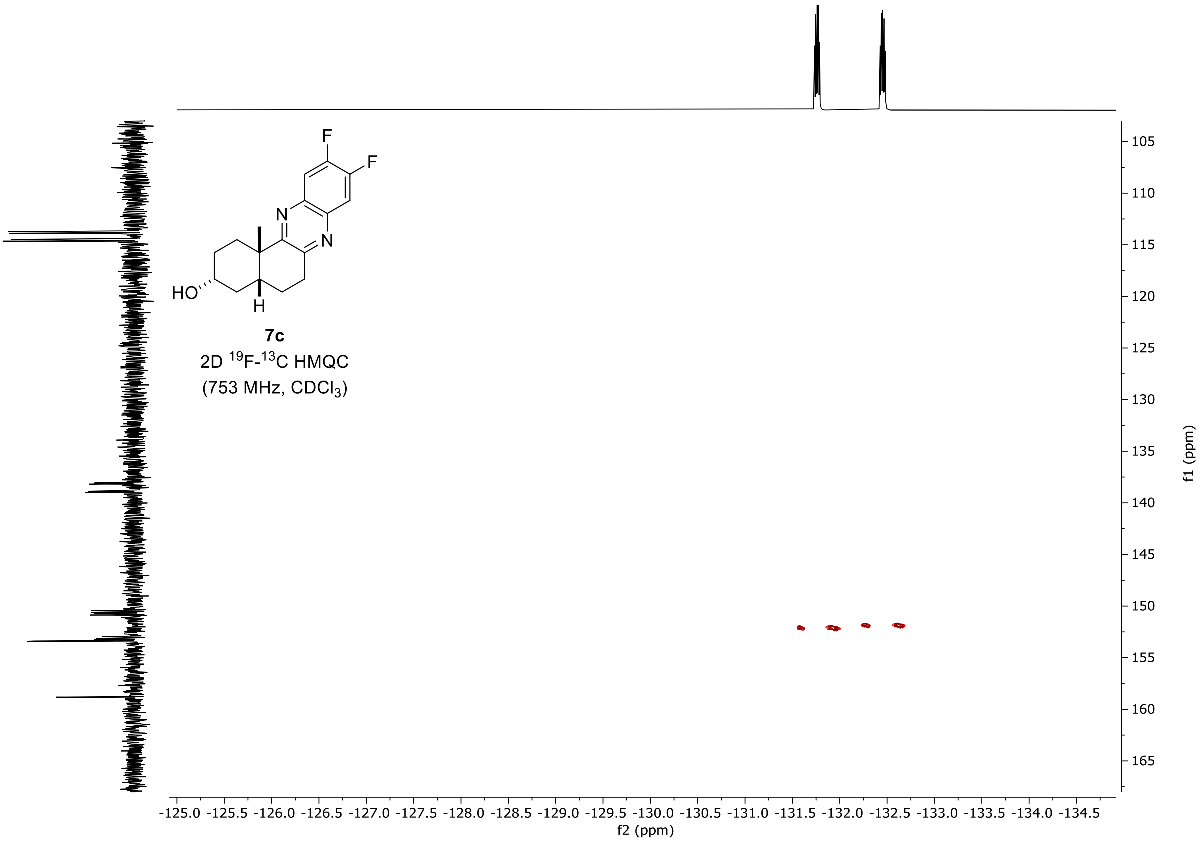

7c

2D ^{19}F-^{13}C HMQC

(753 MHz, $CDCl_3$)

(3*R,4a*R**,12b*S**)-9,10-Dichloro-12b-methyl-1,2,3,4,4a,5,6,12b-octahydrobenzo[*a*]phenazin-3-ol (7d)**

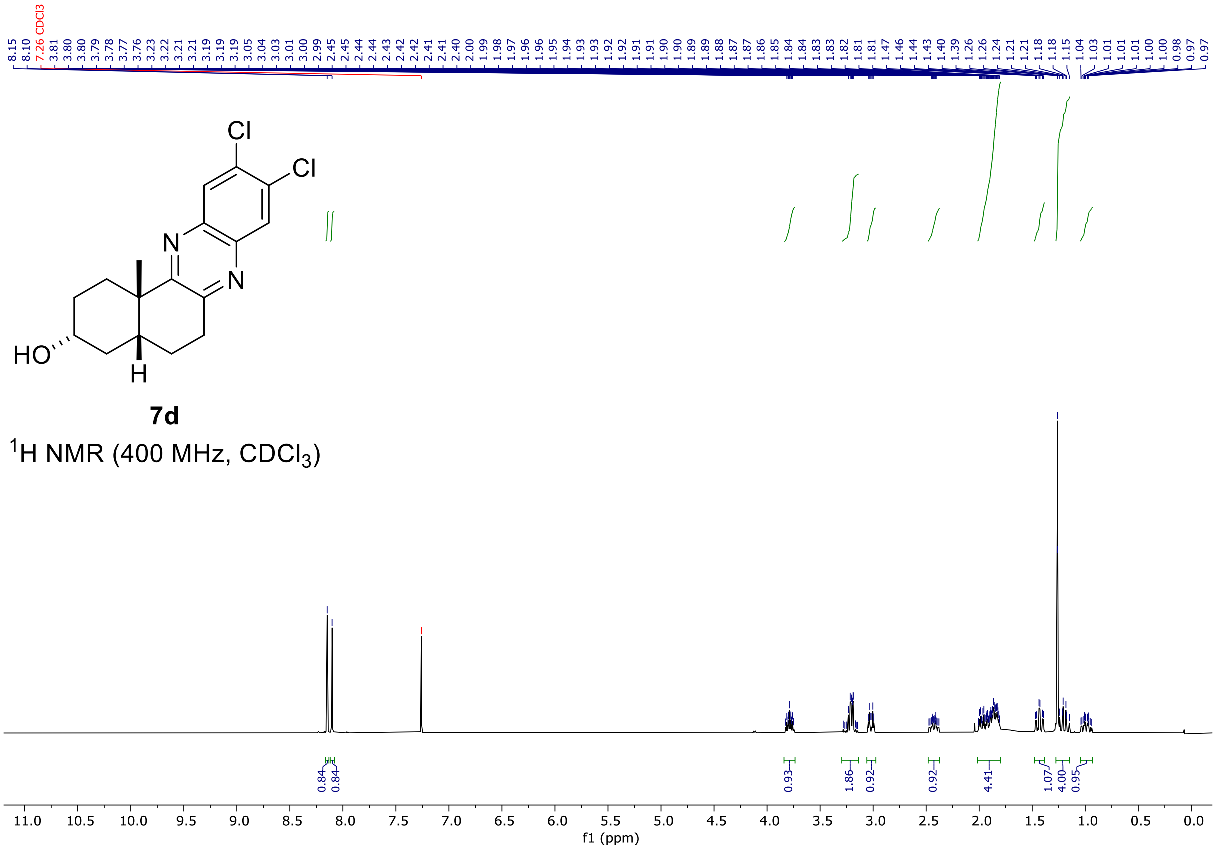

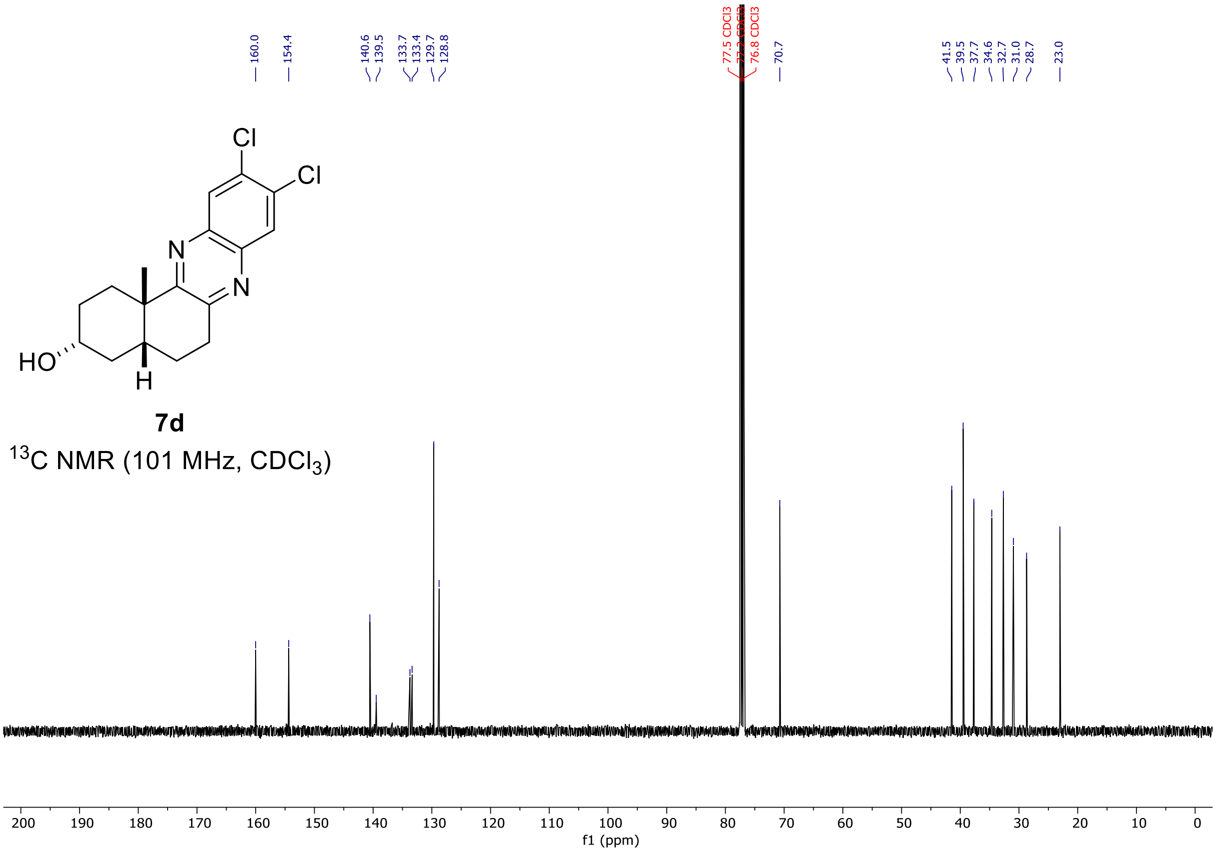

(3*R,4a*R**,12b*S**)-9,10-Dibromo-12b-methyl-1,2,3,4,4[a],5,6,12b-octahydrobenzo[*a*]phenazin-3-ol (7e)**

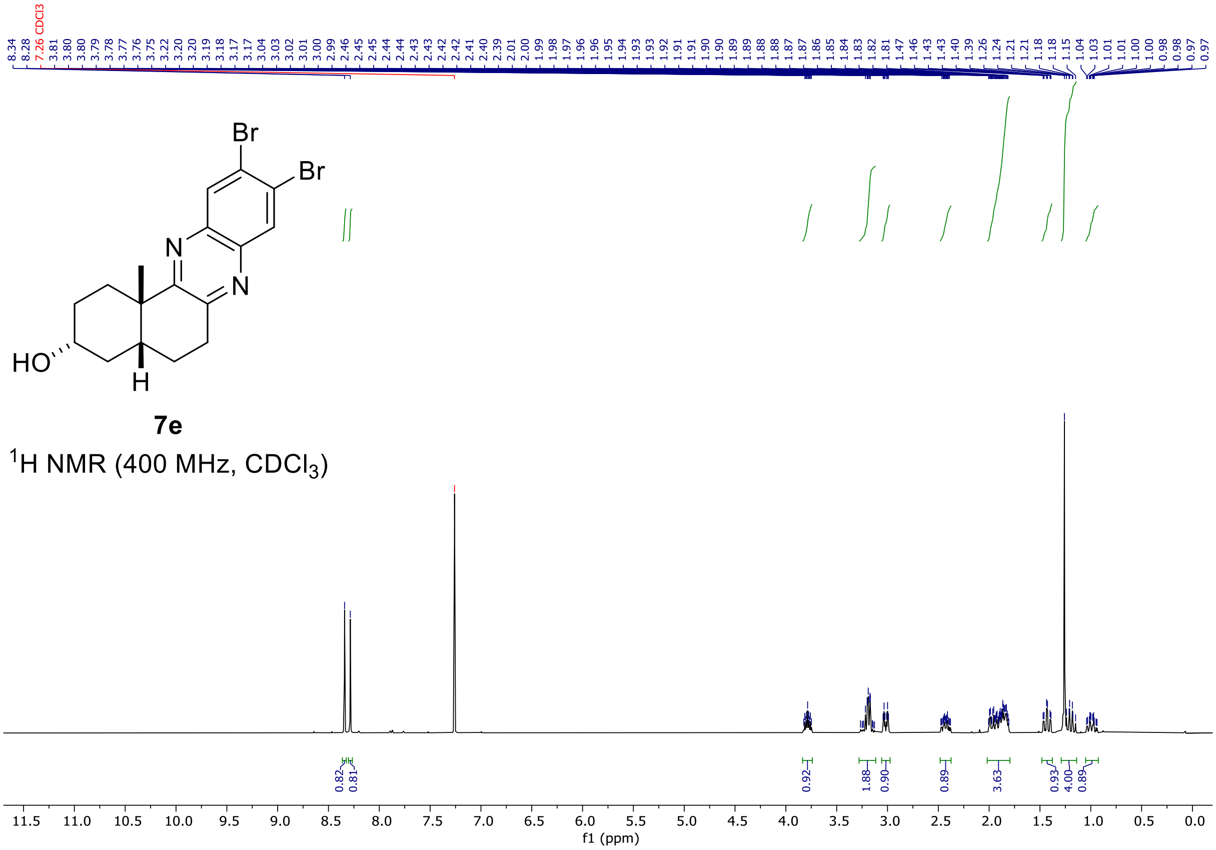

7e

^{1}H NMR (400 MHz, $CDCl_3$)

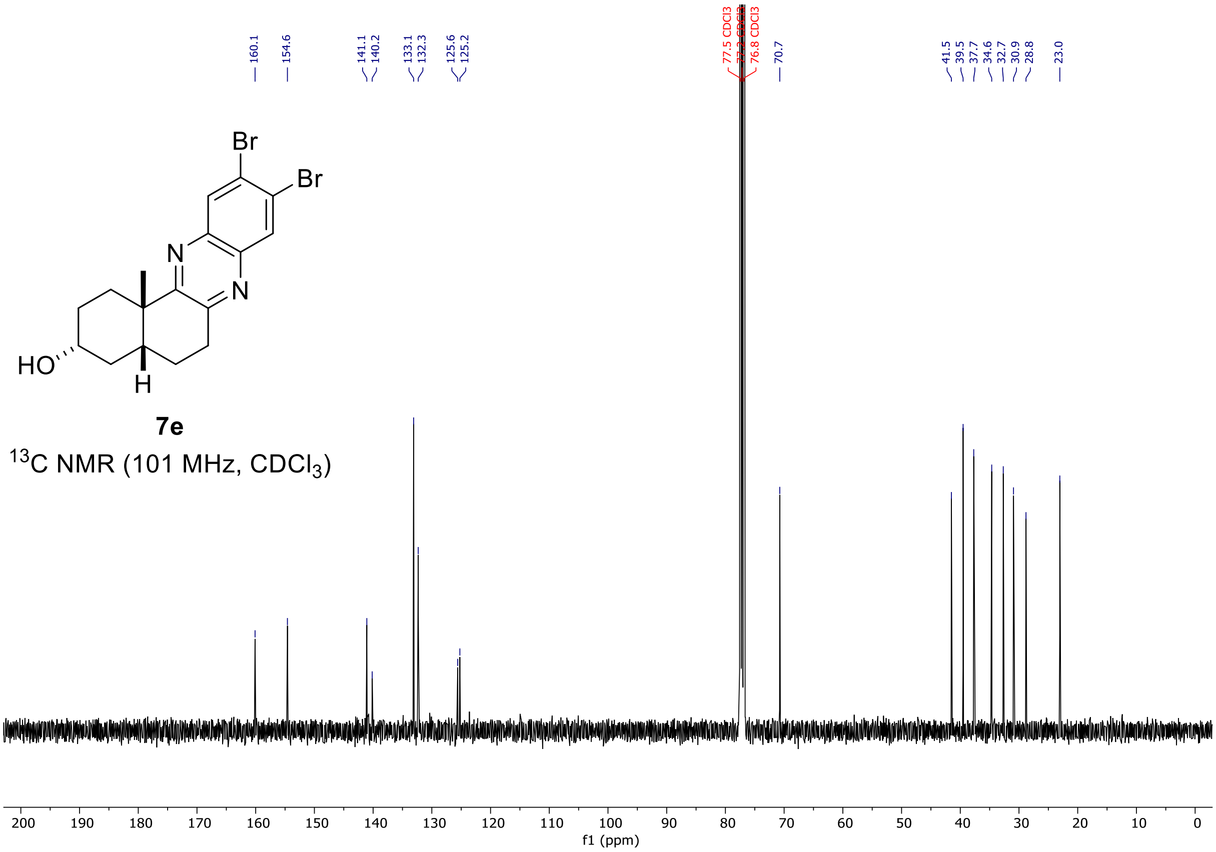

7e

^{13}C NMR (101 MHz, $CDCl_3$)

(5a*R**,7*R**,9a*S**)-2-Amino-9a-methyl-4,5,5a,6,7,8,9,9a-octahydronaphtho[1,2-*d*]thiazol-7-ol (8a)

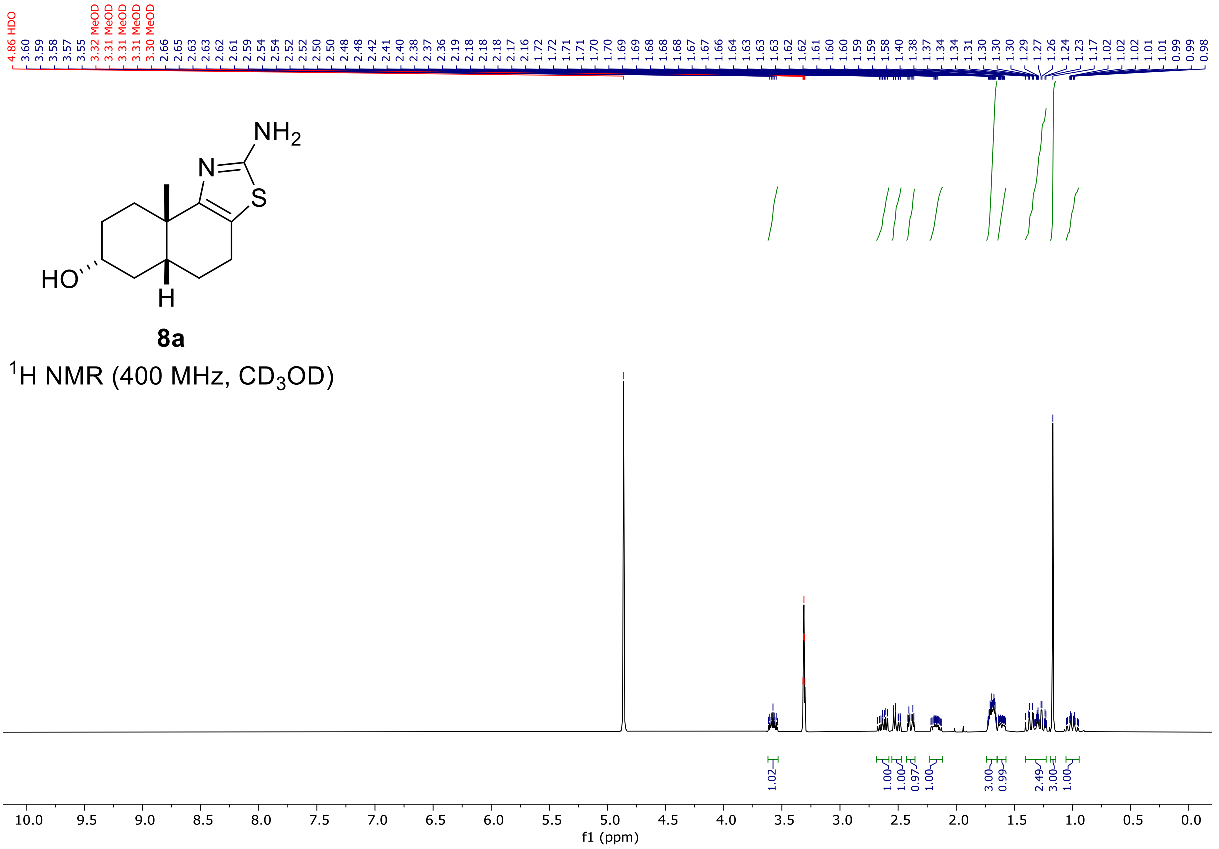

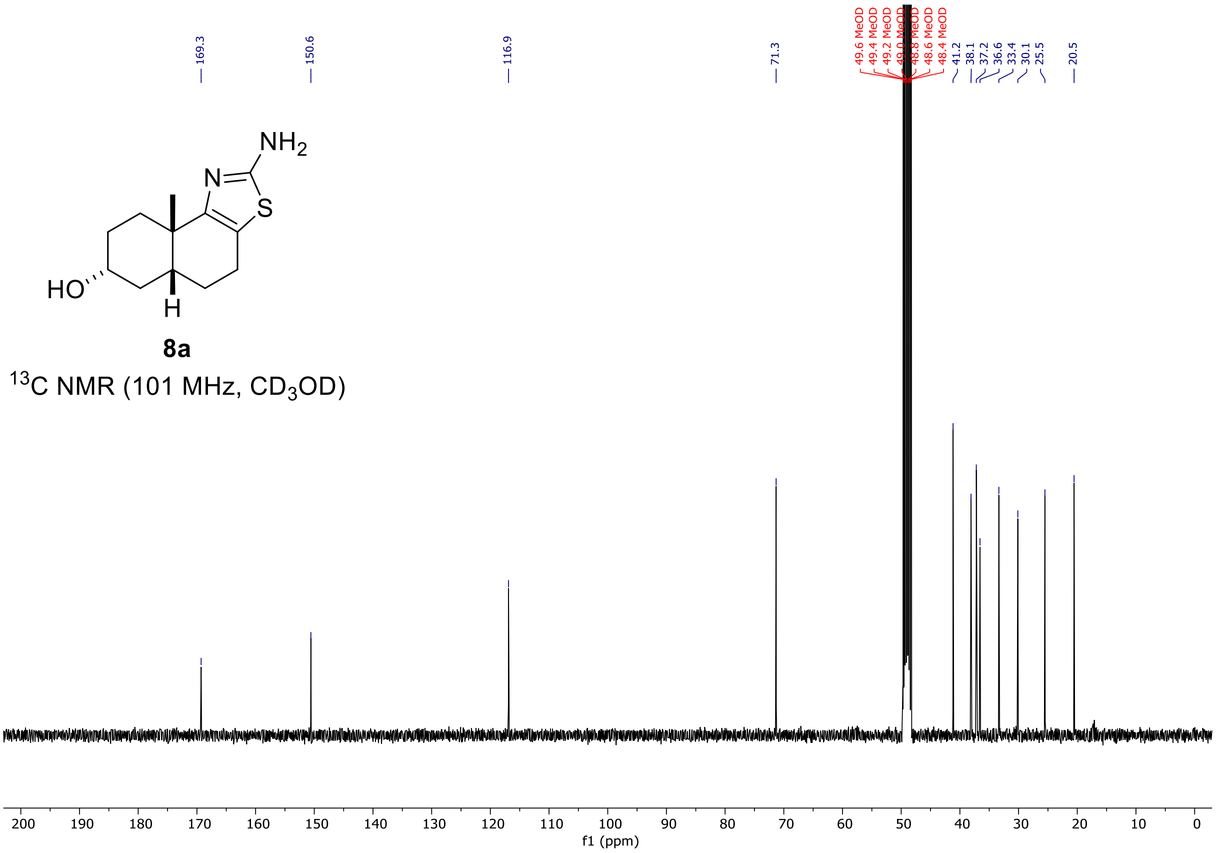

(5a*R**,7*R**,9a*S**)-2,9a-Dimethyl-4,5,5a,6,7,8,9,9a-octahydronaphtho[1,2-*d*]thiazol-7-ol (8b)

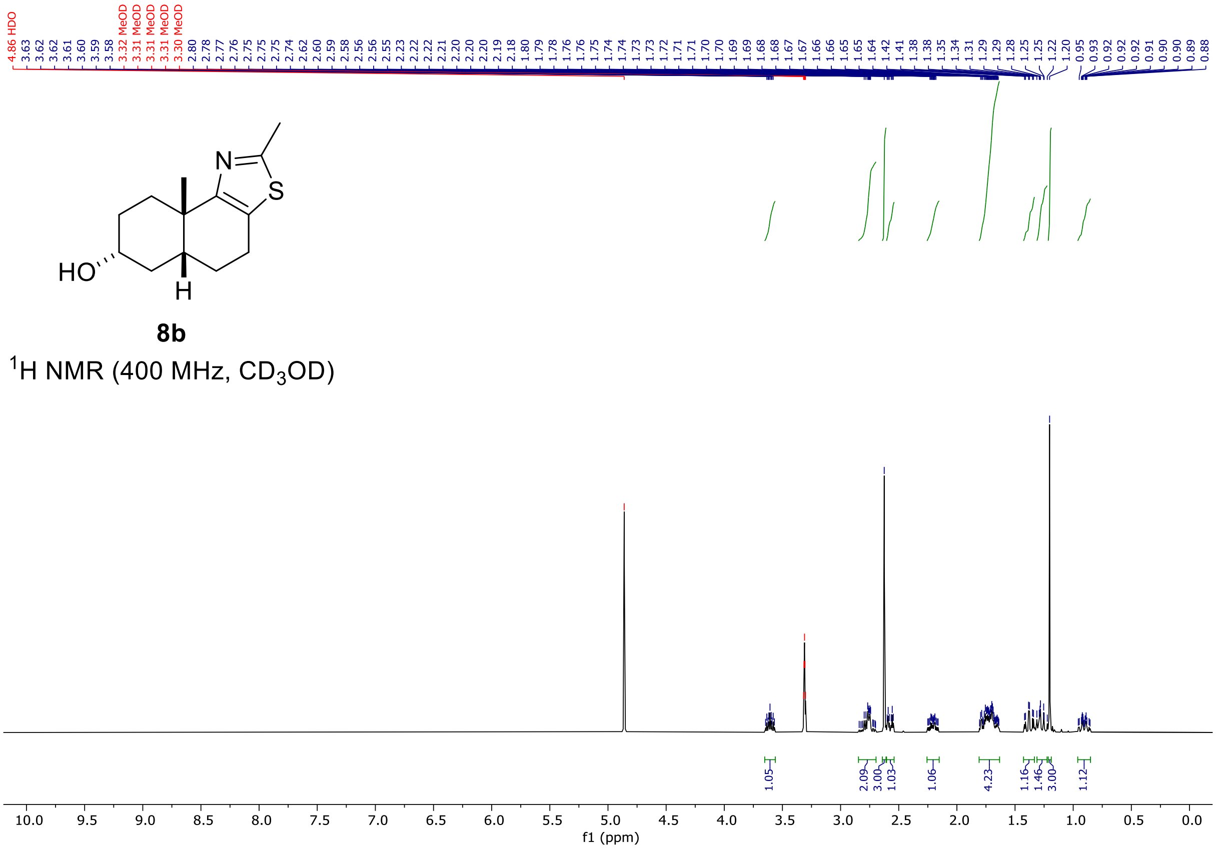

8b

^{1}H NMR (400 MHz, CD_3OD)

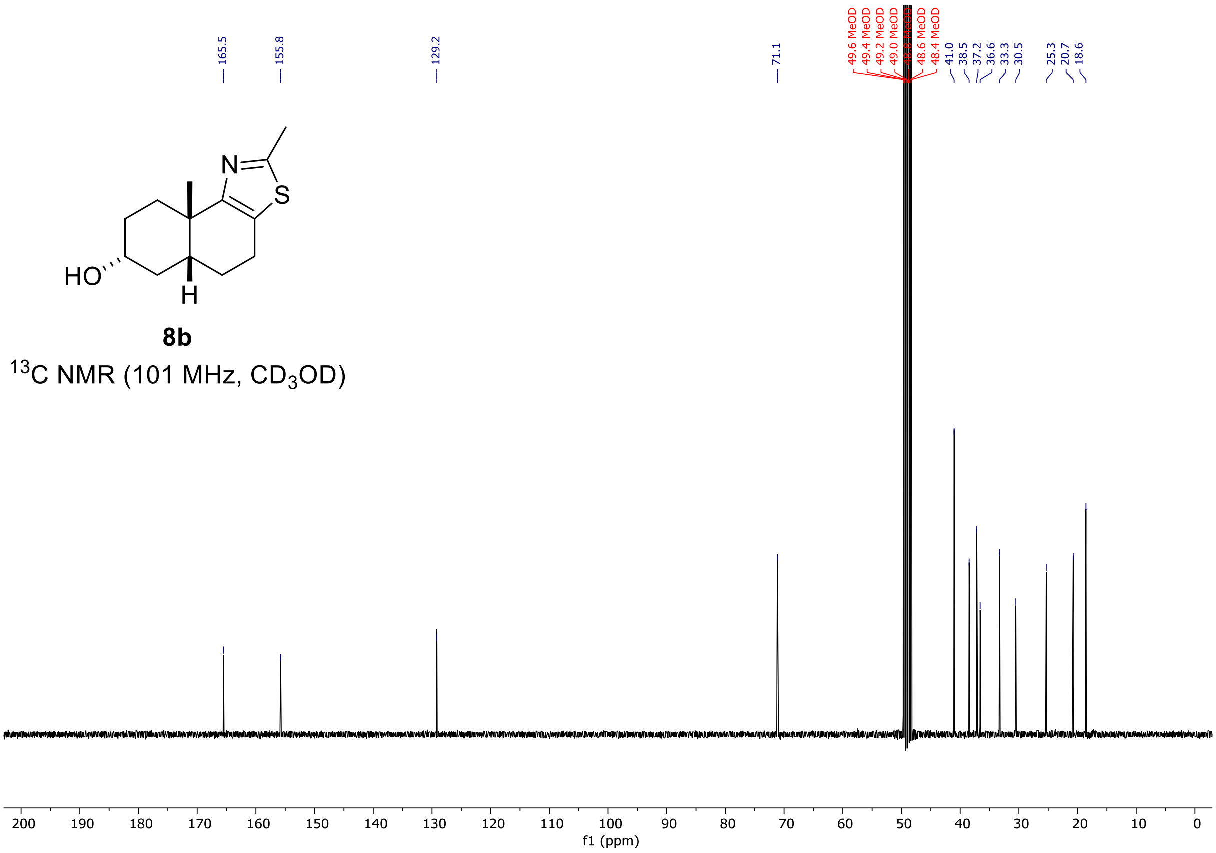

8b

^{13}C NMR (101 MHz, CD_3OD)

(5a*R,7*R**,9a*S**)-9a-Methyl-2-(pyridin-3-yl)-4,5,5a,6,7,8,9,9a-octahydronaphtho[1,2-*d*]thiazol-7-ol (8c)**

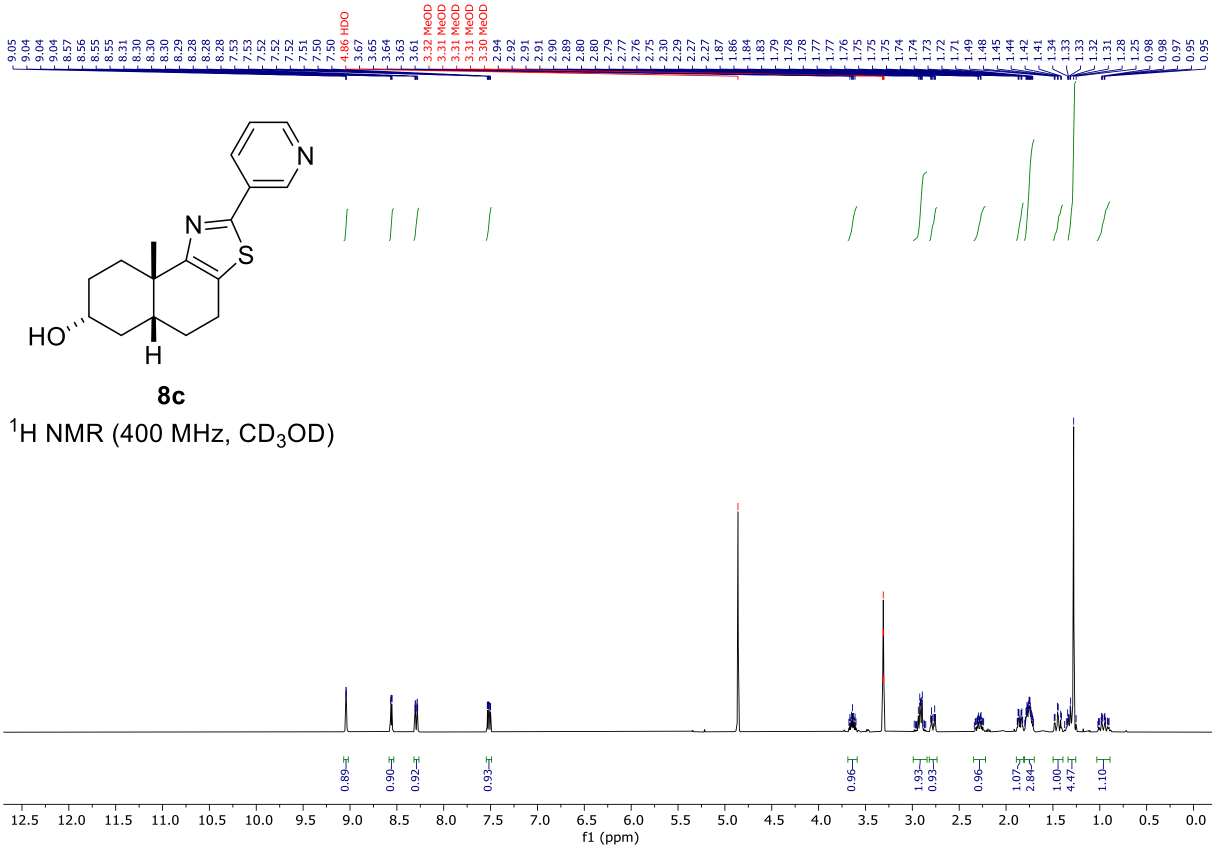

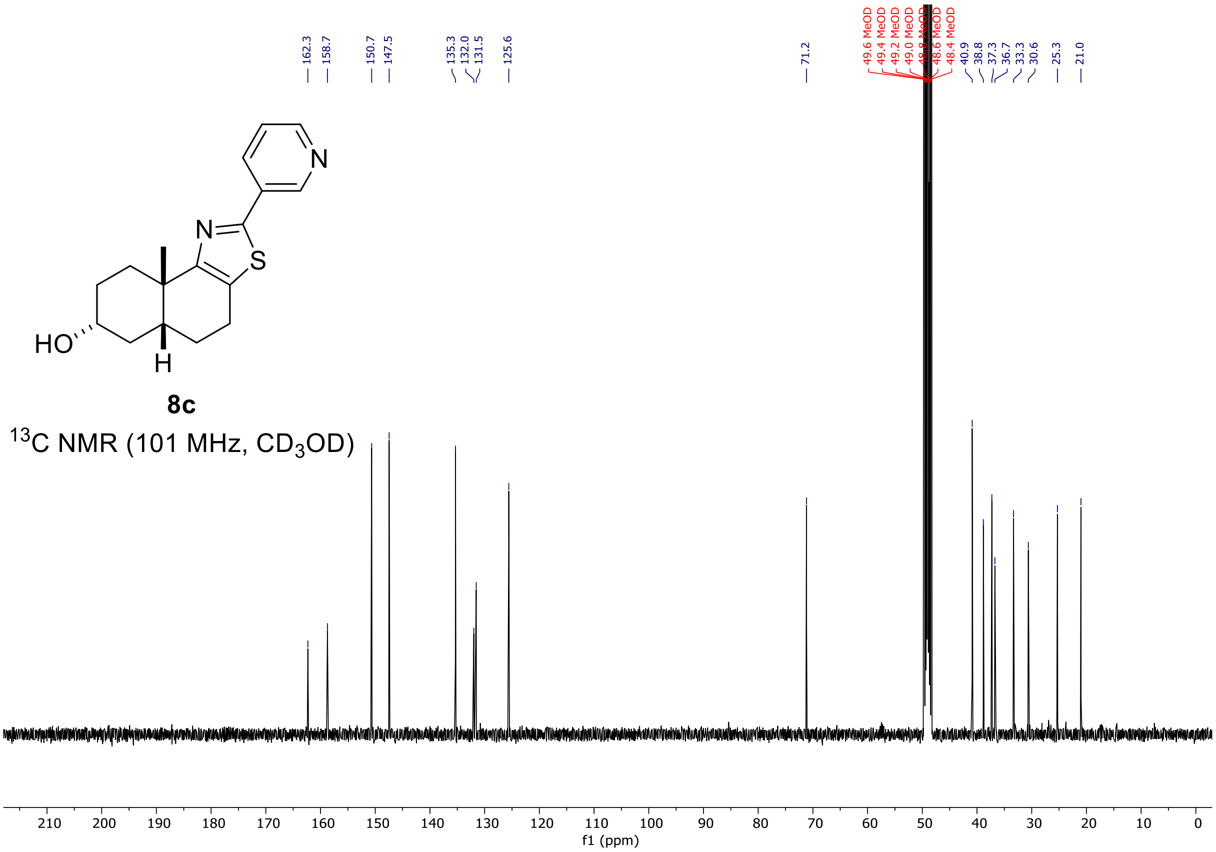

(5a*R,7*R**,9a*S**)-2-(4-Methoxyphenyl)-9a-methyl-4,5,5a,6,7,8,9,9a-octahydronaphtho[1,2-*d*]thiazol-7-ol (8d)**

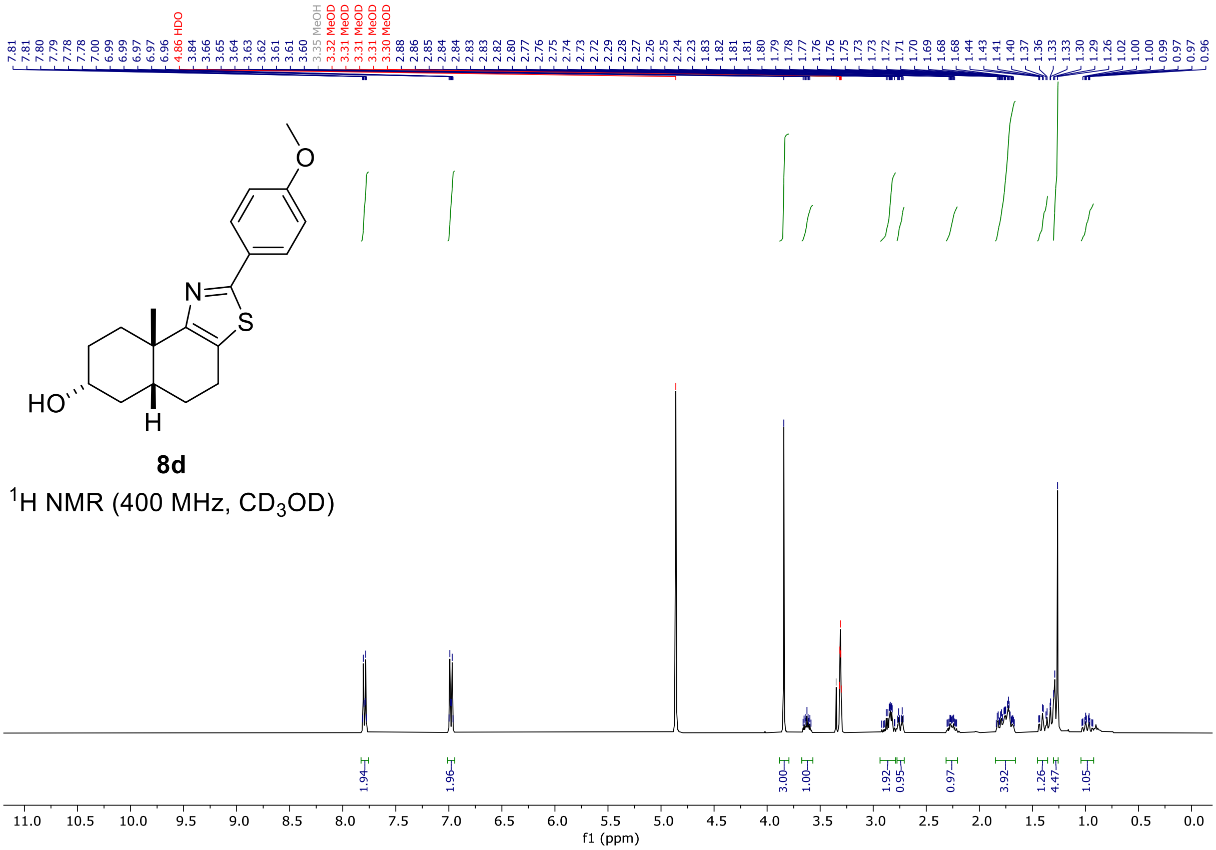

^{1}H NMR (400 MHz, CD_3OD)

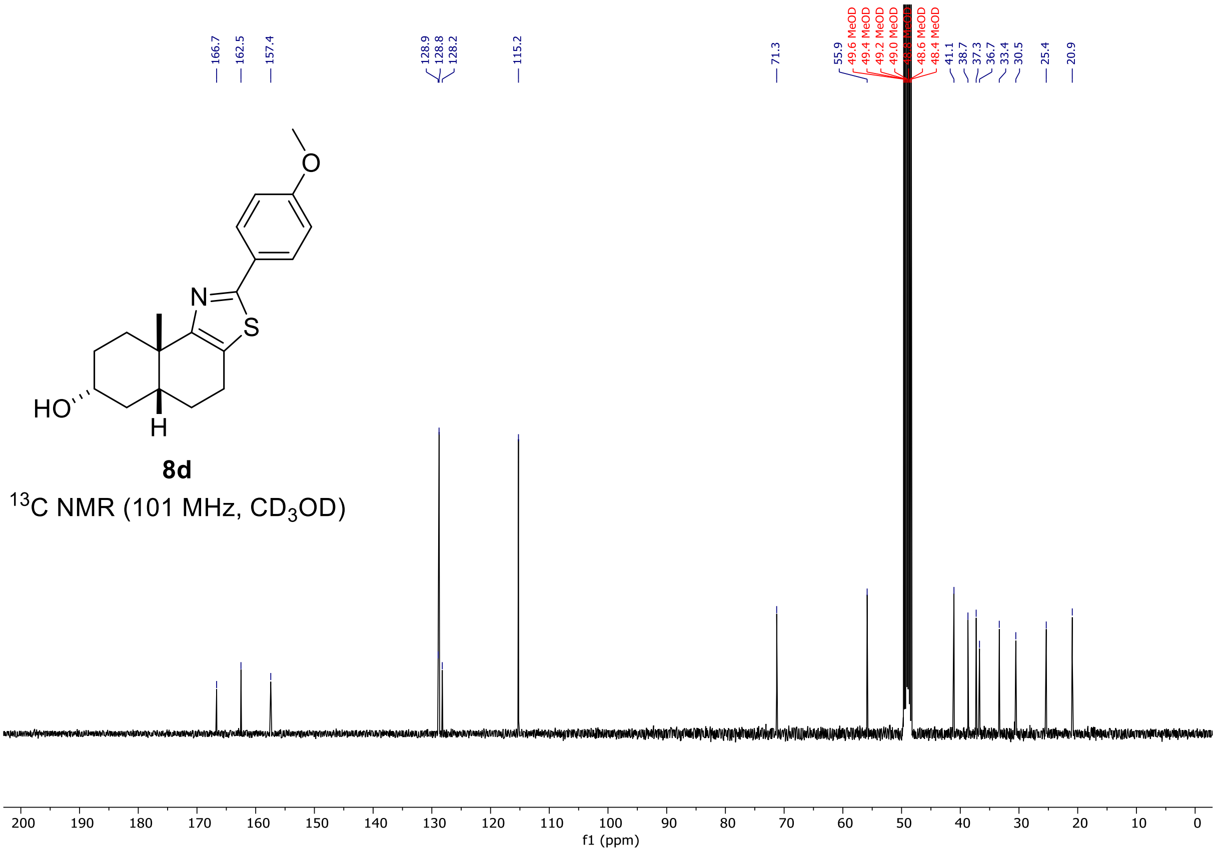

^{13}C NMR (101 MHz, CD_3OD)

(5a*R,7*R**,9a*S**)-2-(4-Fluorophenyl)-9a-methyl-4,5,5a,6,7,8,9,9a-octahydronaphtho[1,2-*d*]thiazol-7-ol (8e)**

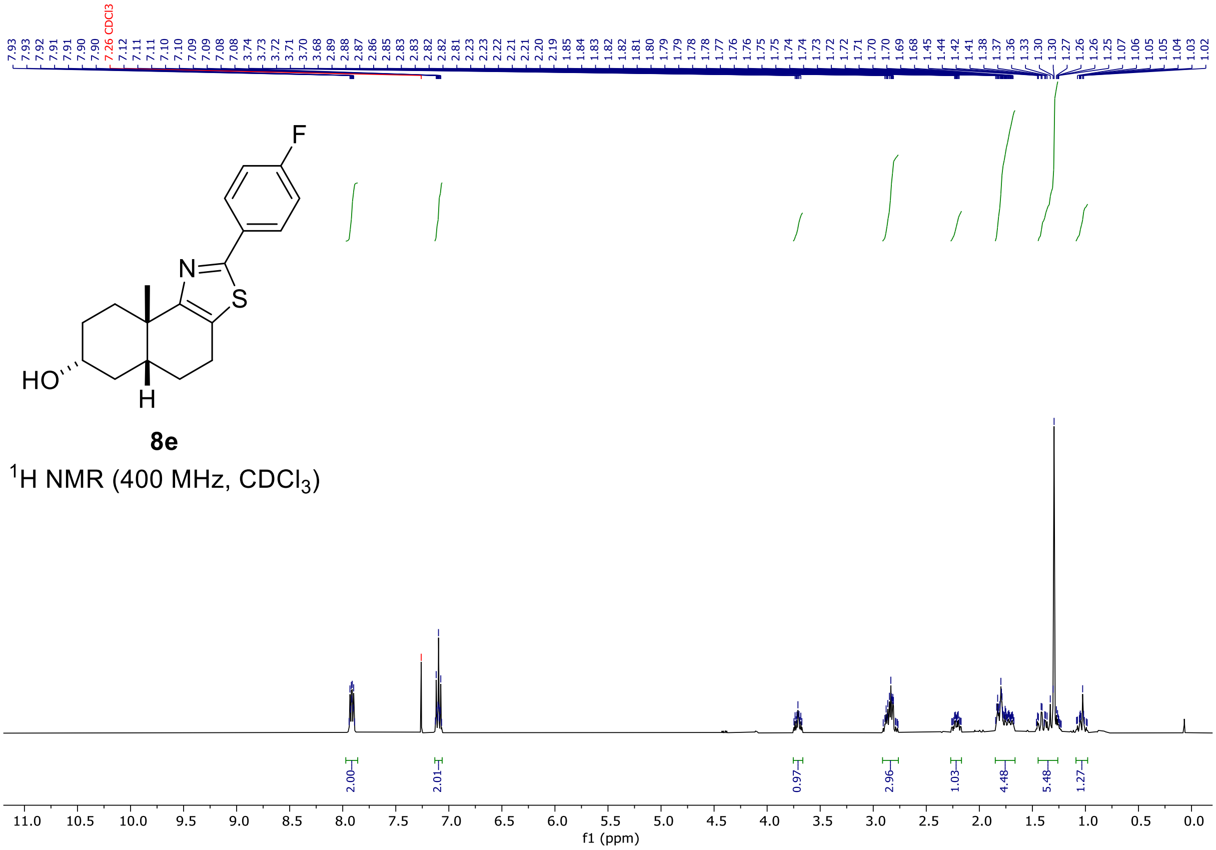

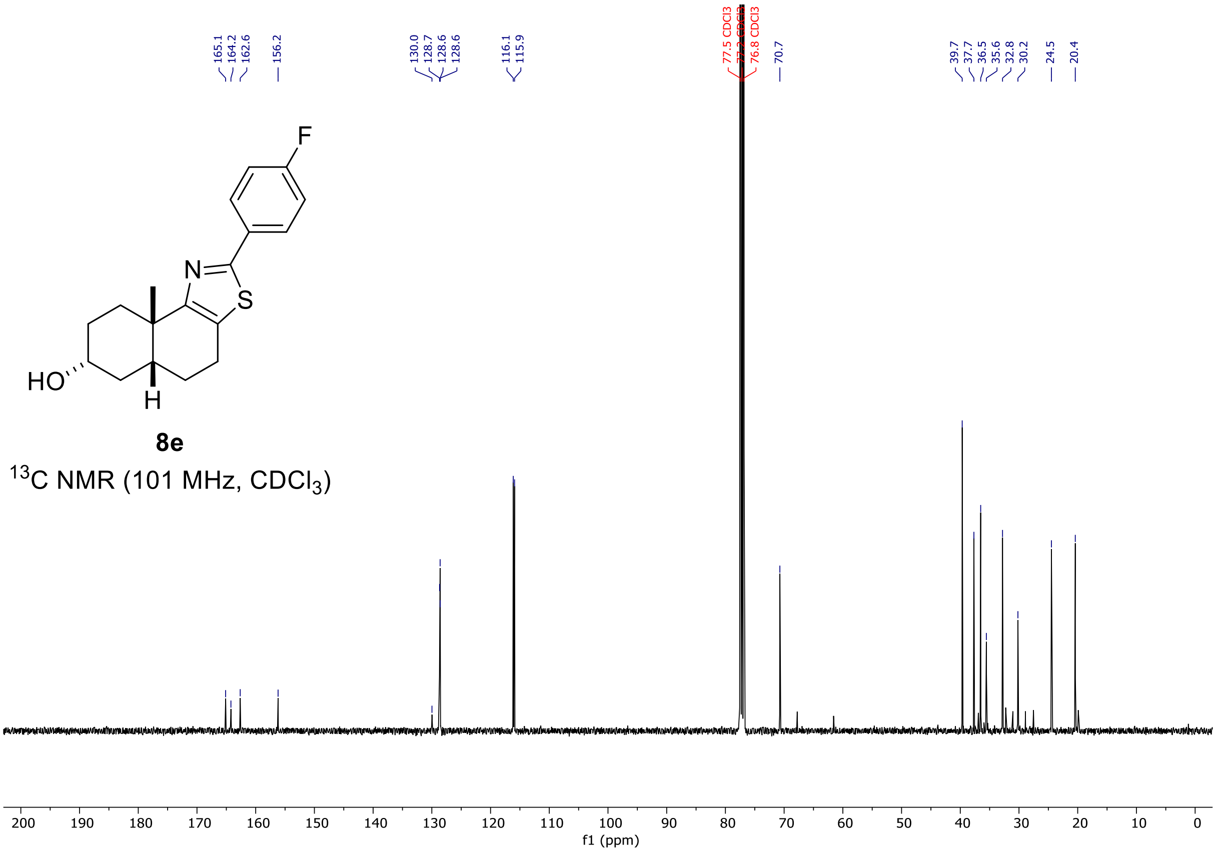

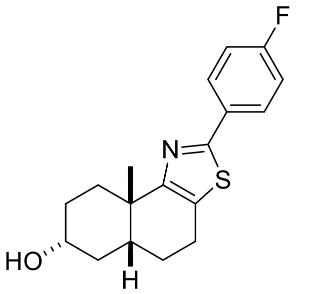

8e

^{19}F NMR (377 MHz, $CDCl_3$)

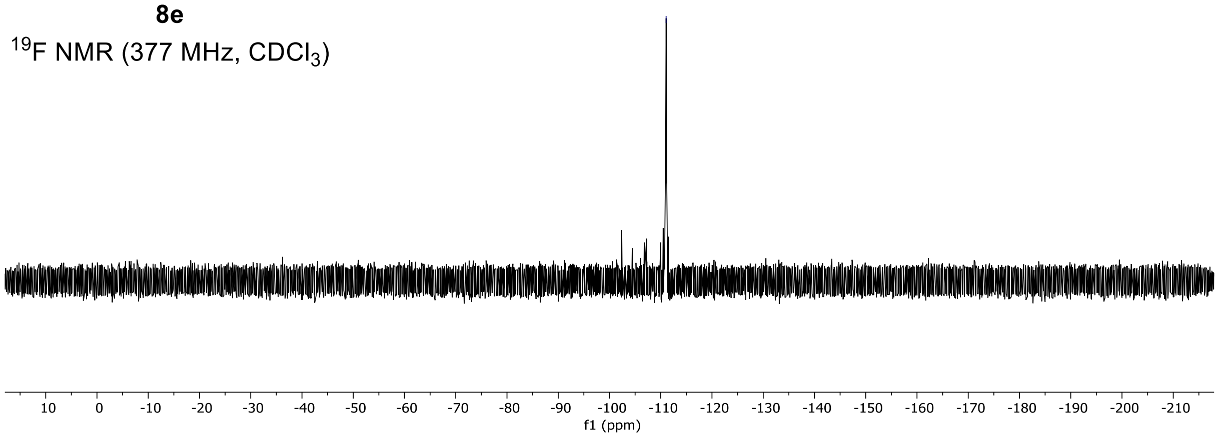

(5a*R,7*R**,9a*S**)-9a-Methyl-2-phenyl-4,5,5a,6,7,8,9,9a-octahydro-3*H*-naphtho[1,2-*d*]imidazol-7-ol (9)**

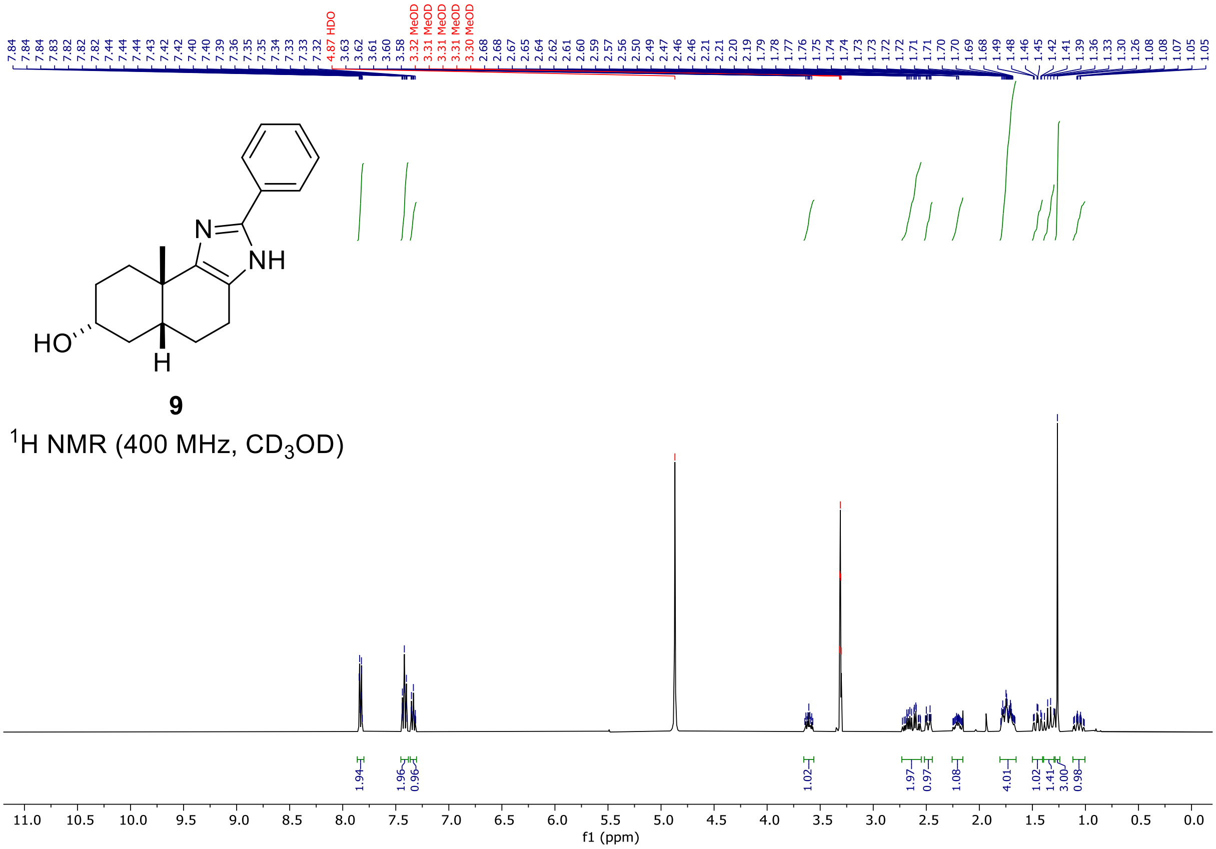

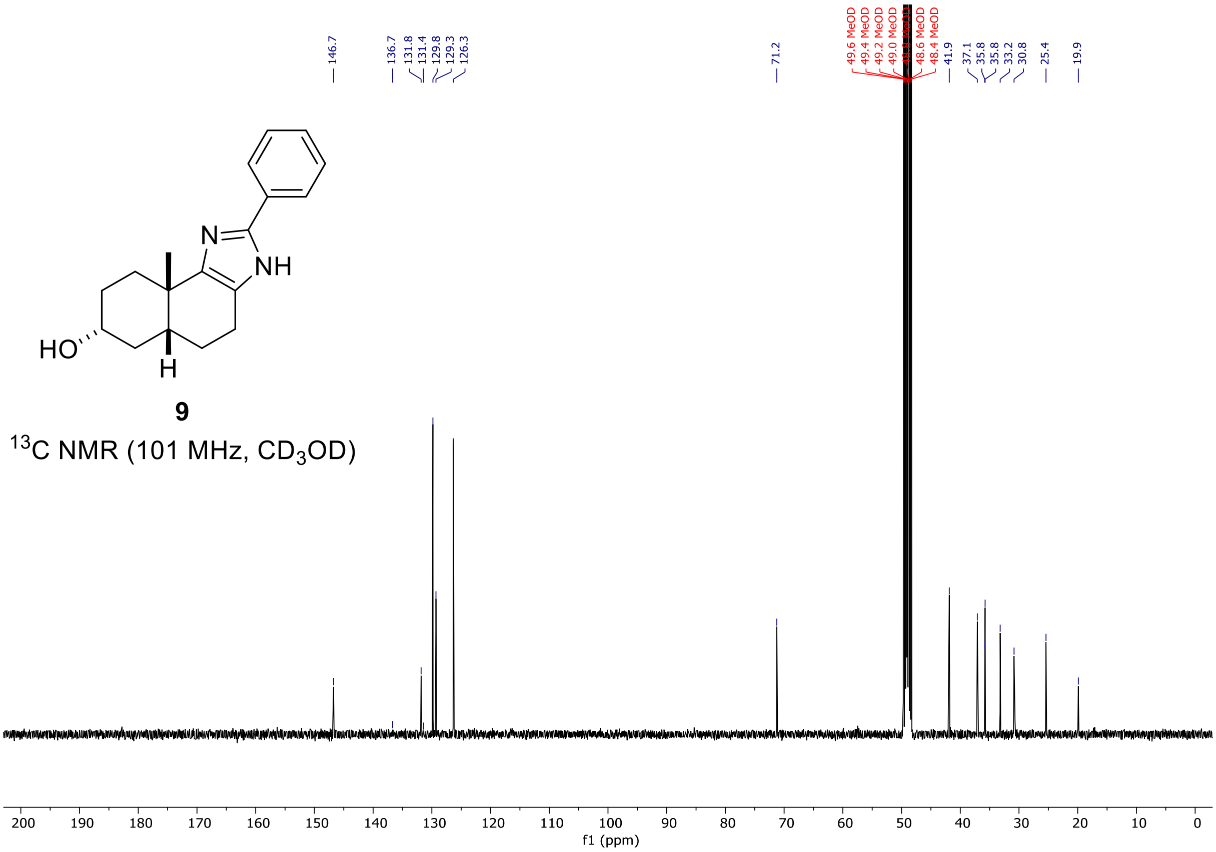

(3*R**,4a*R**,11b*S**)-11b-Methyl-2,3,4,4a,5,6,11,11b-octahydro-1*H*-benzo[*a*]carbazol-3-ol (10a)

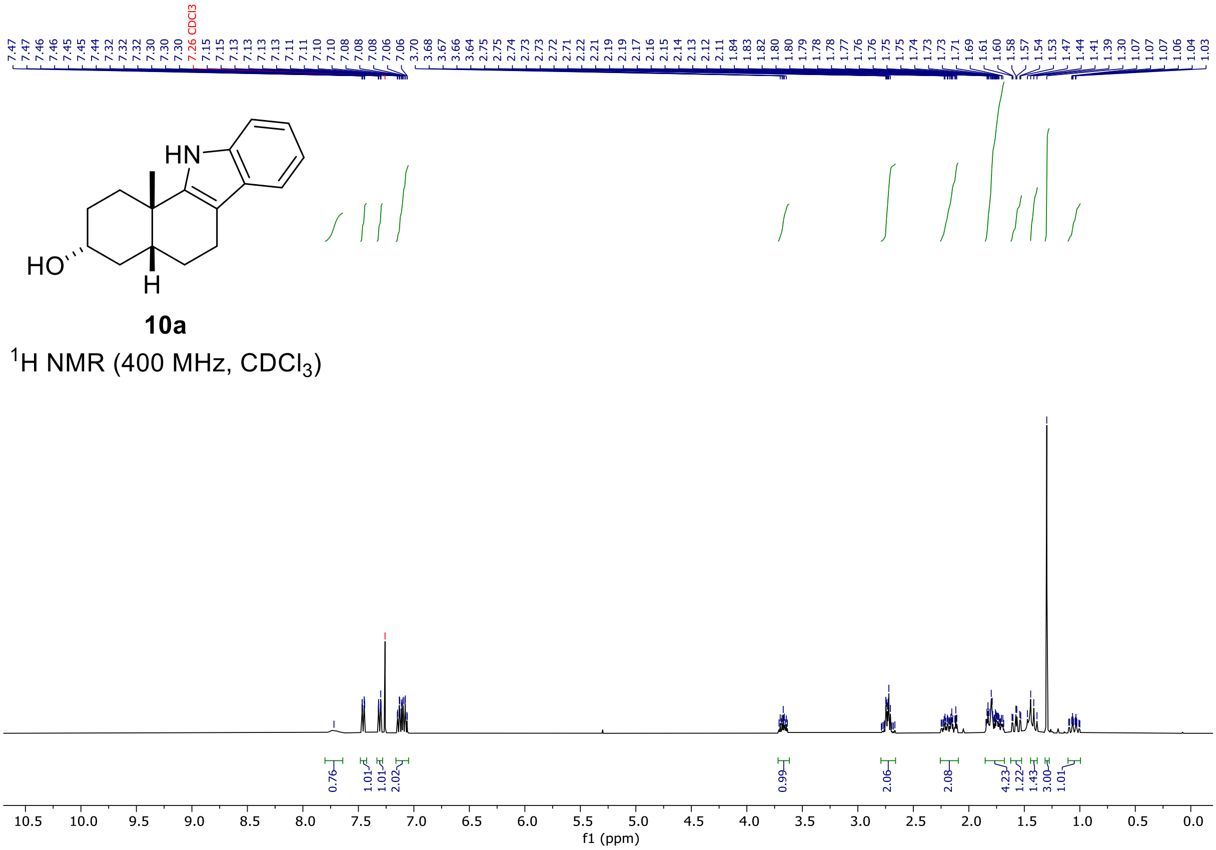

10a

^{1}H NMR (400 MHz, $CDCl_3$)

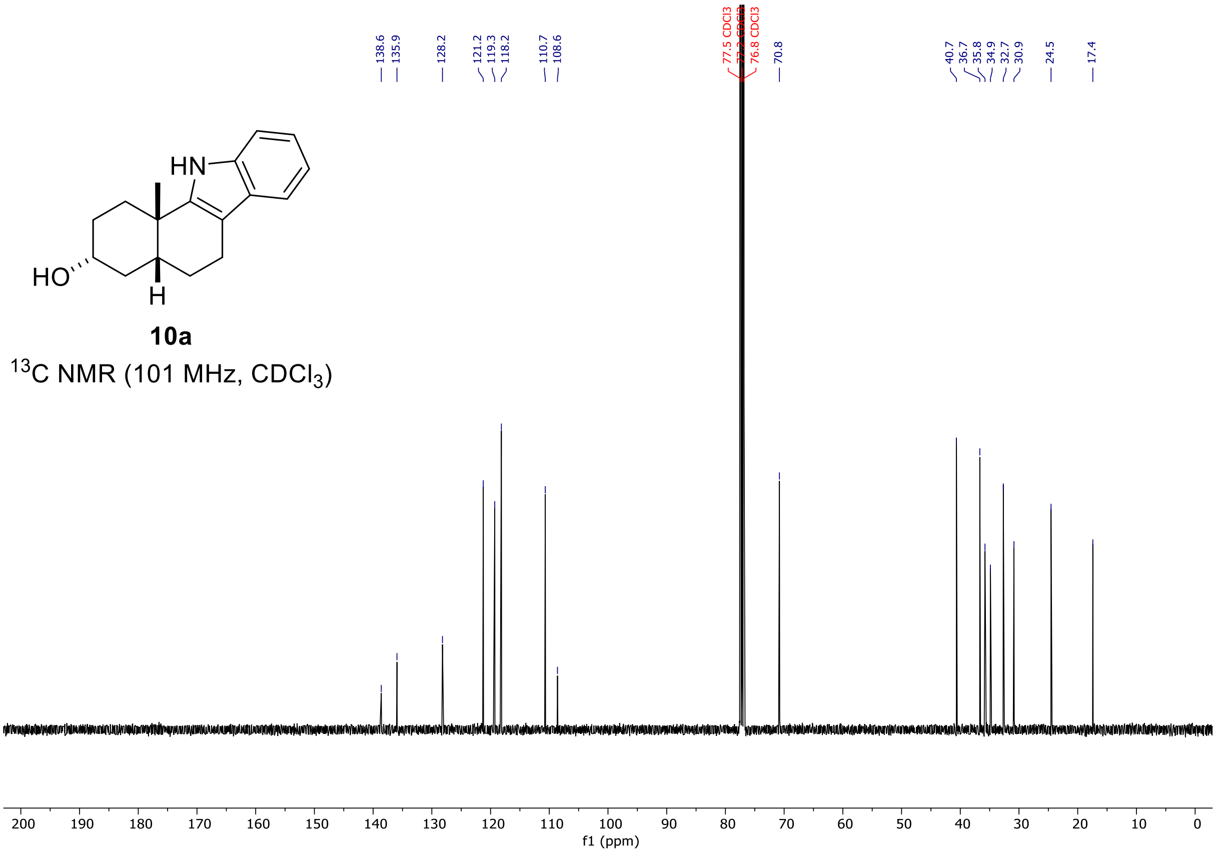

10a

^{13}C NMR (101 MHz, $CDCl_3$)

(3*R,4a*R**,11b*S**)-8-Bromo-11b-methyl-2,3,4,4ª,5,6,11,11b-octahydro-1*H*-benzo[*a*]carbazol-3-ol (10b)**

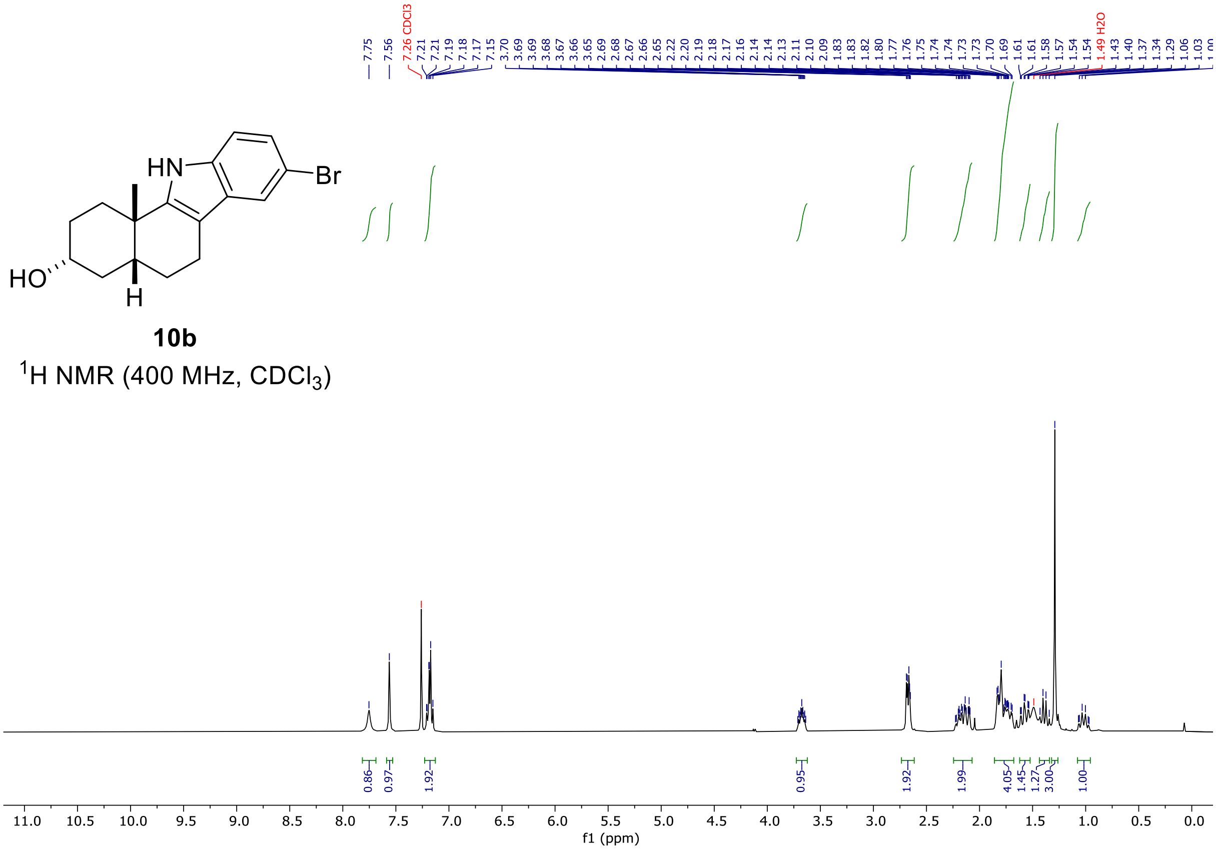

10b

^{1}H NMR (400 MHz, $CDCl_3$)

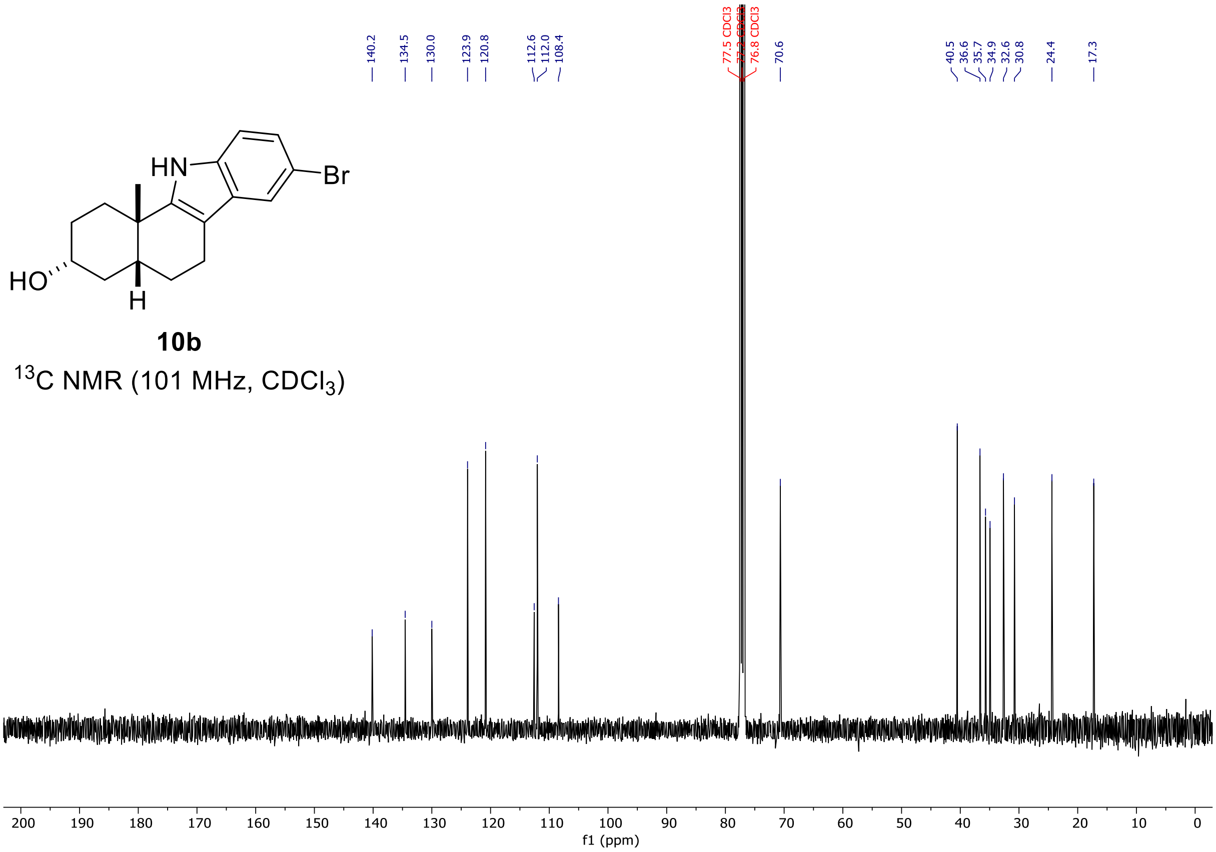

10b

^{13}C NMR (101 MHz, $CDCl_3$)

(3*R,4a*R**,11b*S**)-8-Chloro-11b-methyl-2,3,4,4a,5,6,11,11b-octahydro-1*H*-benzo[*a*]carbazol-3-ol (10c)**

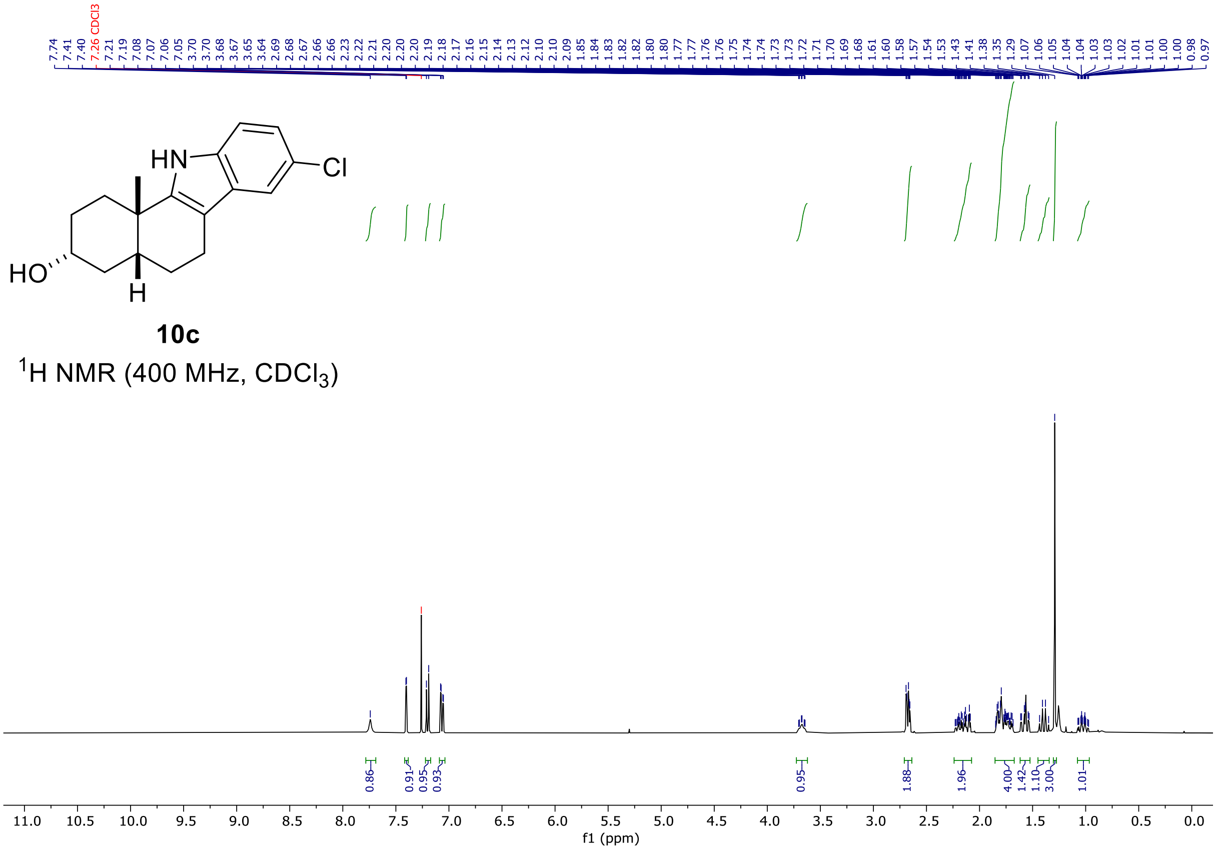

10c

^{1}H NMR (400 MHz, $CDCl_3$)

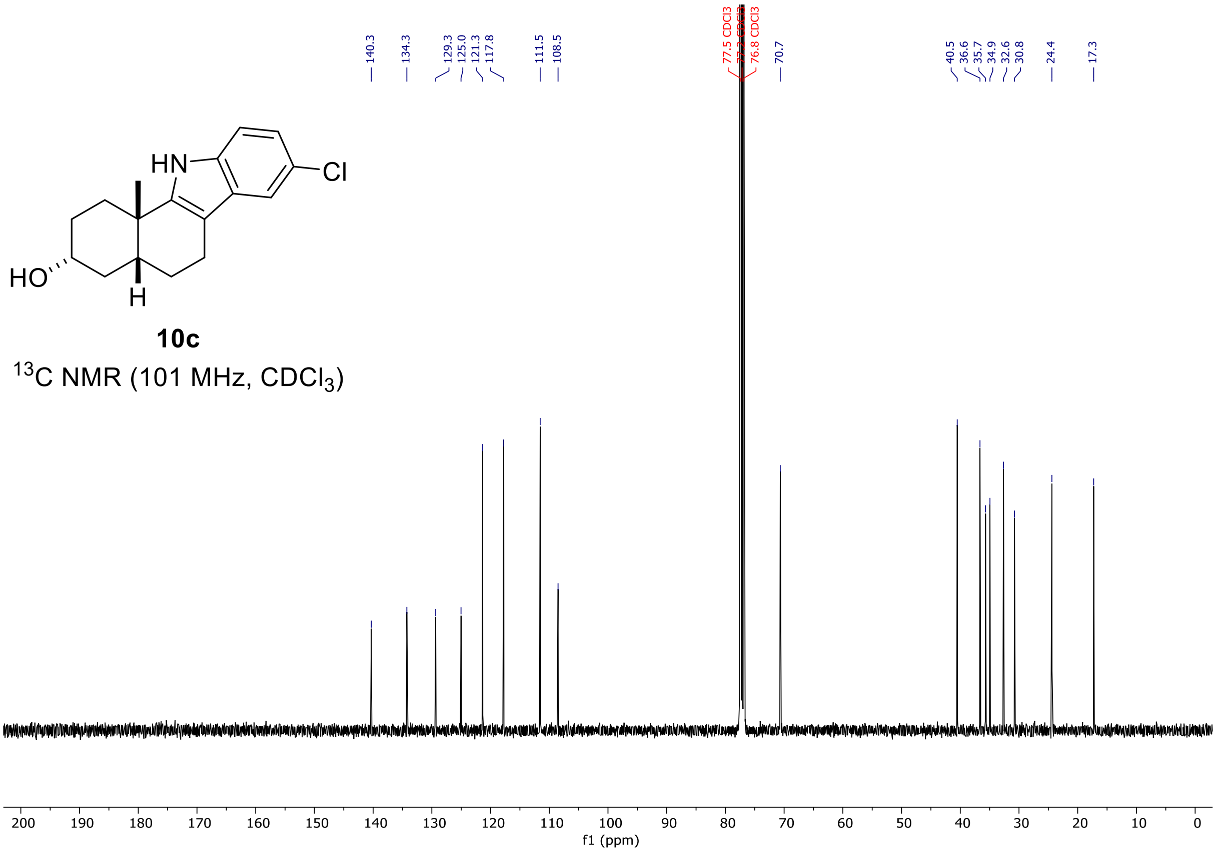

10c

^{13}C NMR (101 MHz, $CDCl_3$)

(3*R,4a*R**,11b*S**)-8-Fluoro-11b-methyl-2,3,4,4^{a},5,6,11,11b-octahydro-1*H*-benzo[*a*]150arbazole-3-ol (10d)**

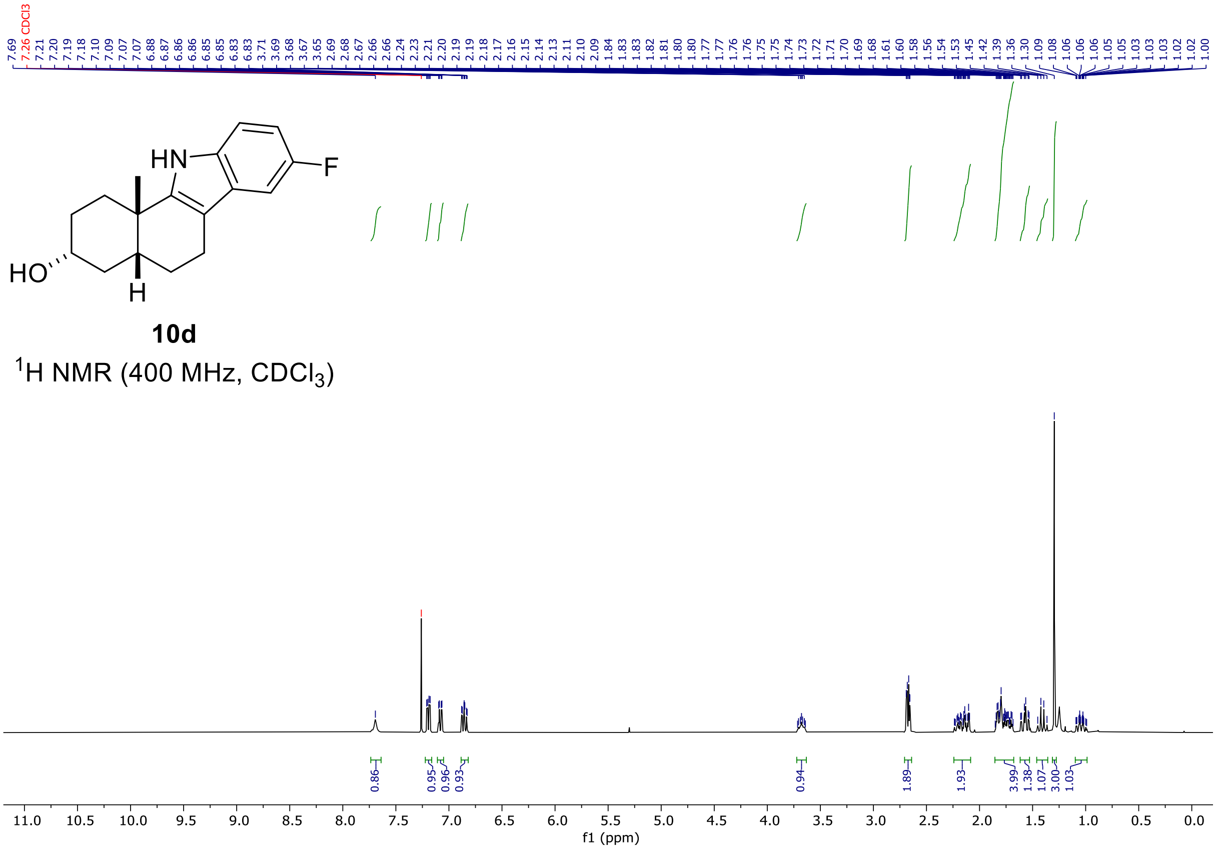

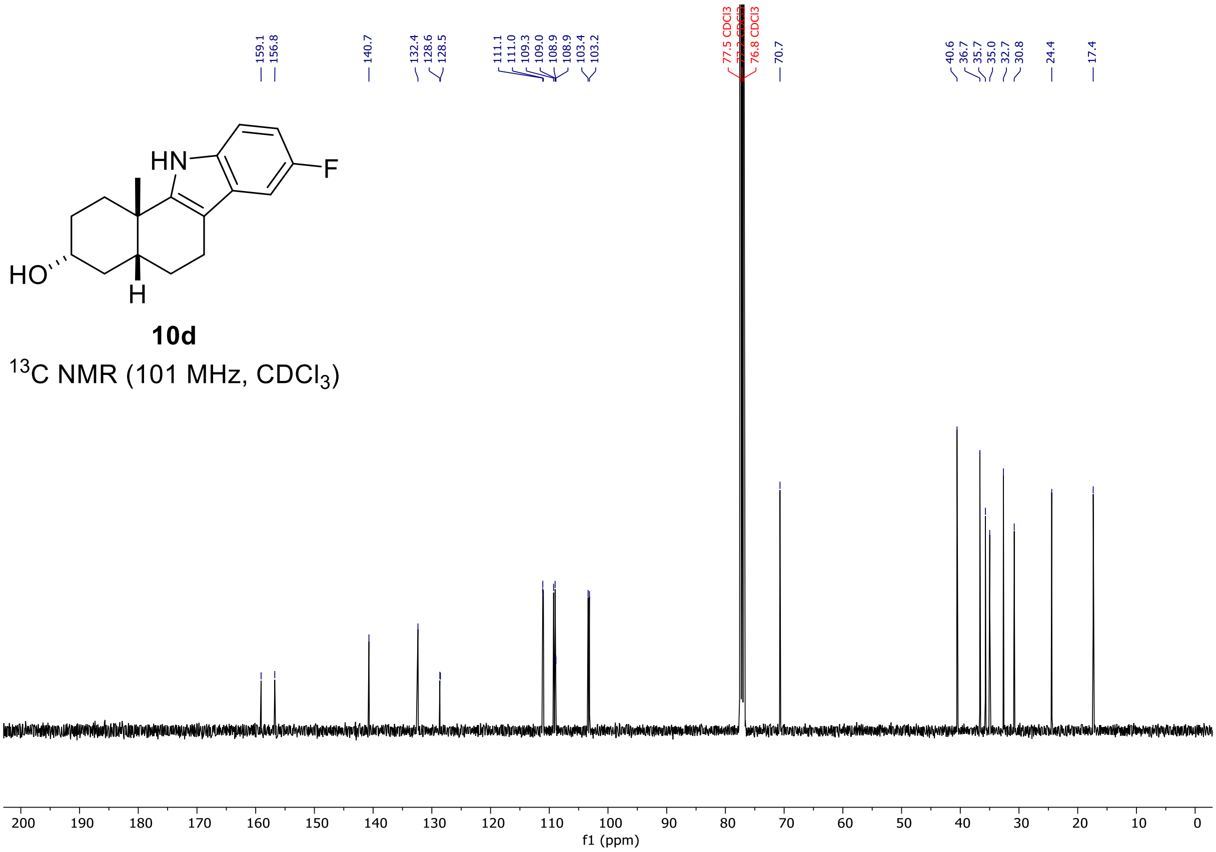

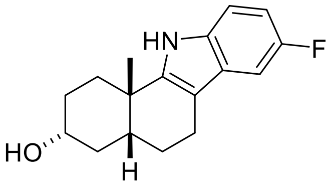

10d

^{19}F NMR (377 MHz, $CDCl_3$)

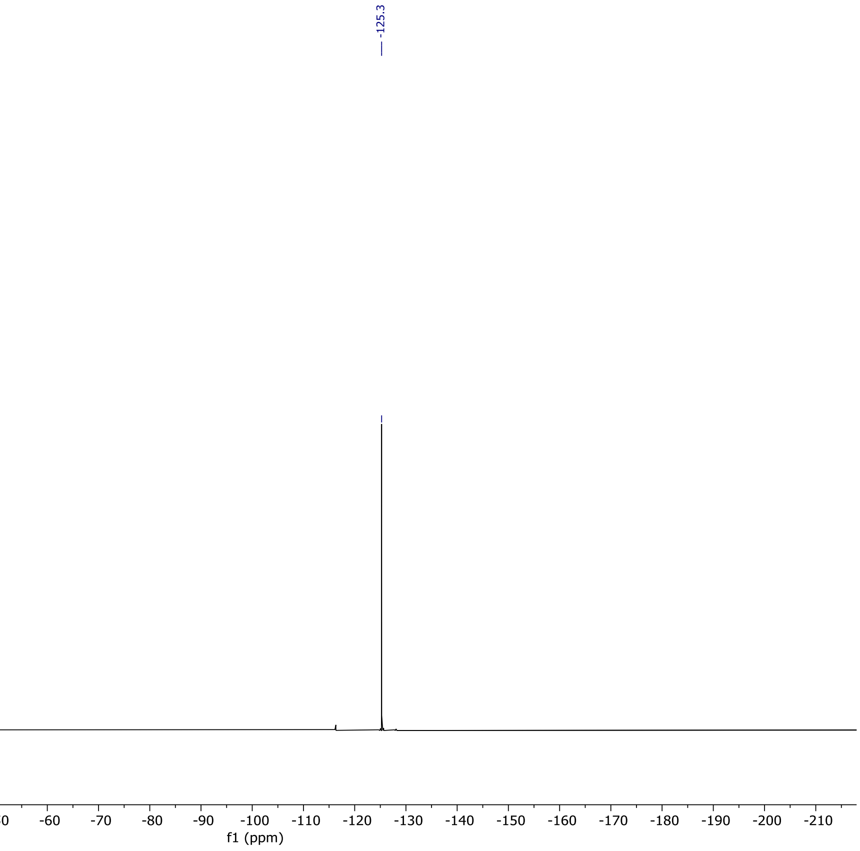

(3*R,4a*R**,11b*S**)-8,11b-Dimethyl-2,3,4,4a,5,6,11,11b-octahydro-1*H*-benzo[*a*]carbazol-3-ol (10e)**

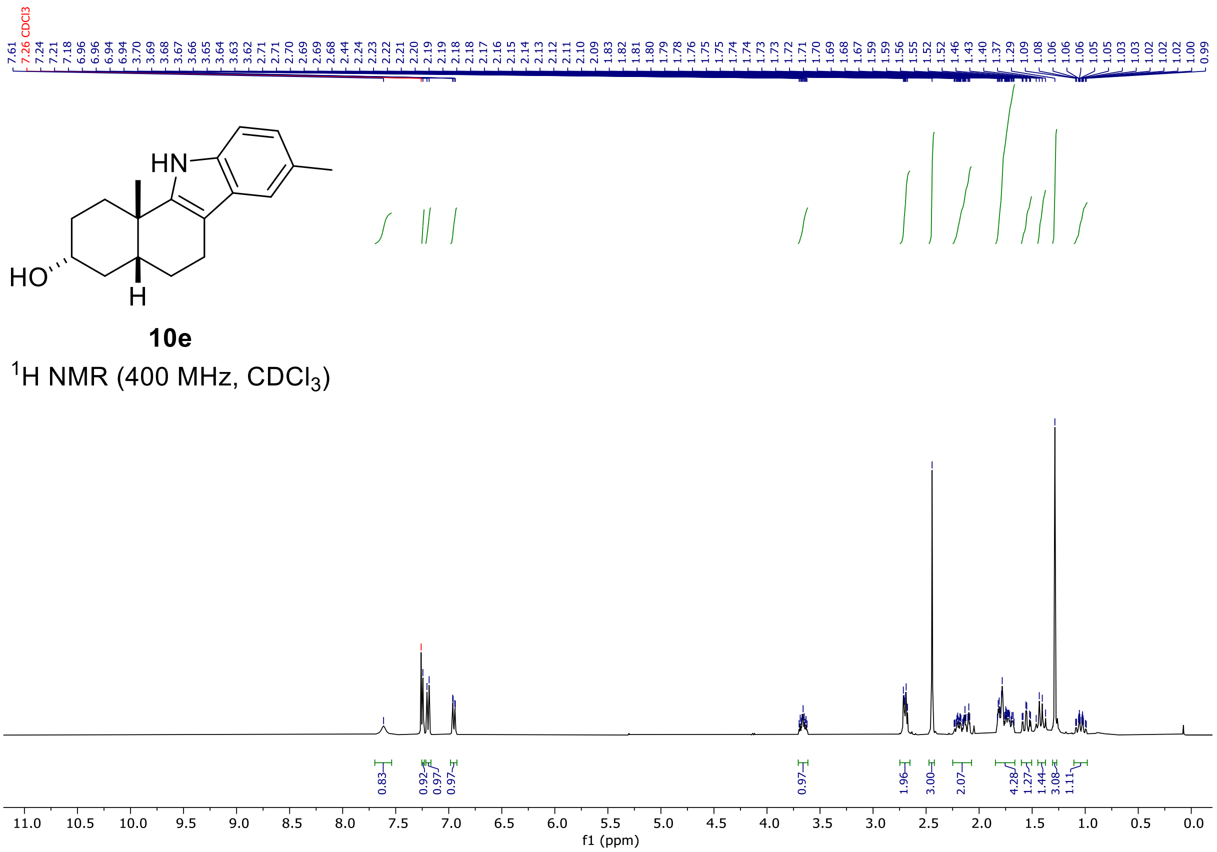

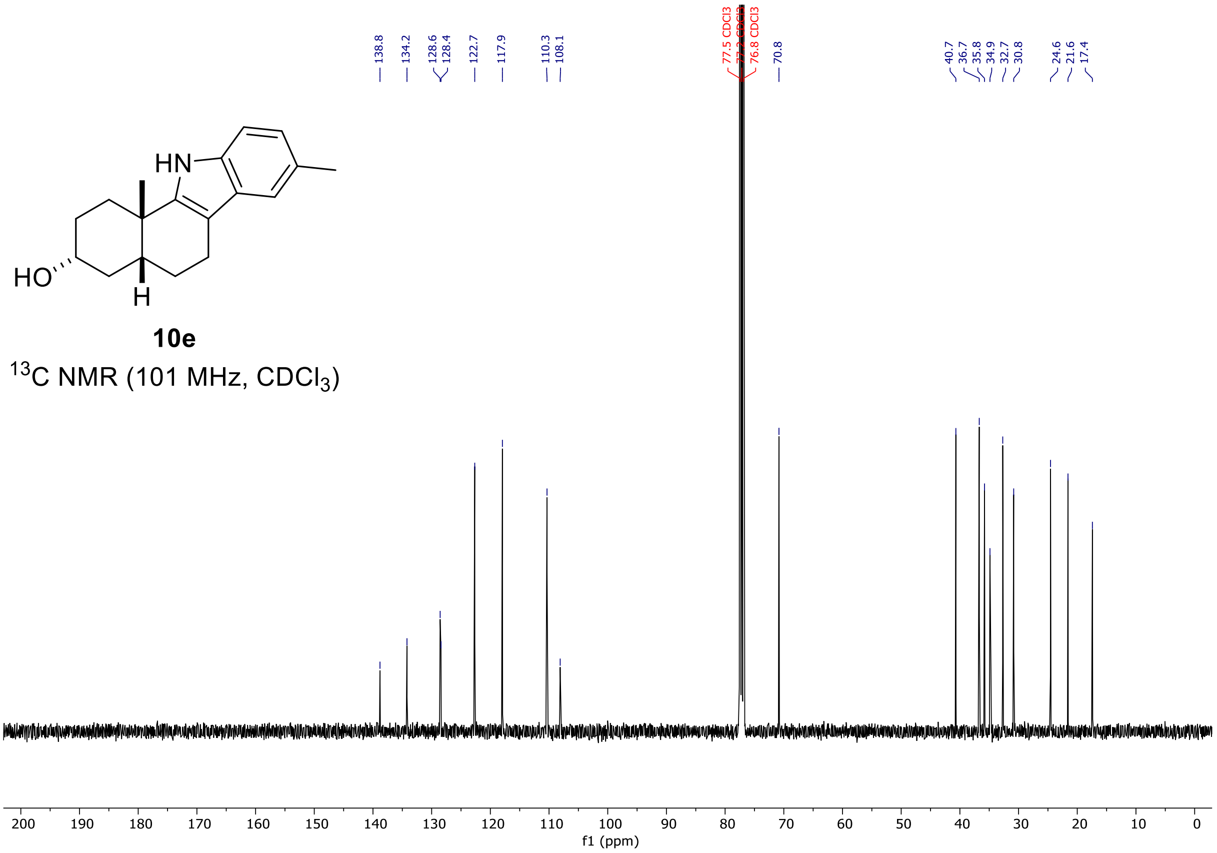

(3*R,4a*R**,11b*S**)-8-Methoxy-11b-methyl-2,3,4,4a,5,6,11,11b-octahydro-1*H*-benzo[*a*]carbazol-3-ol (10f)**

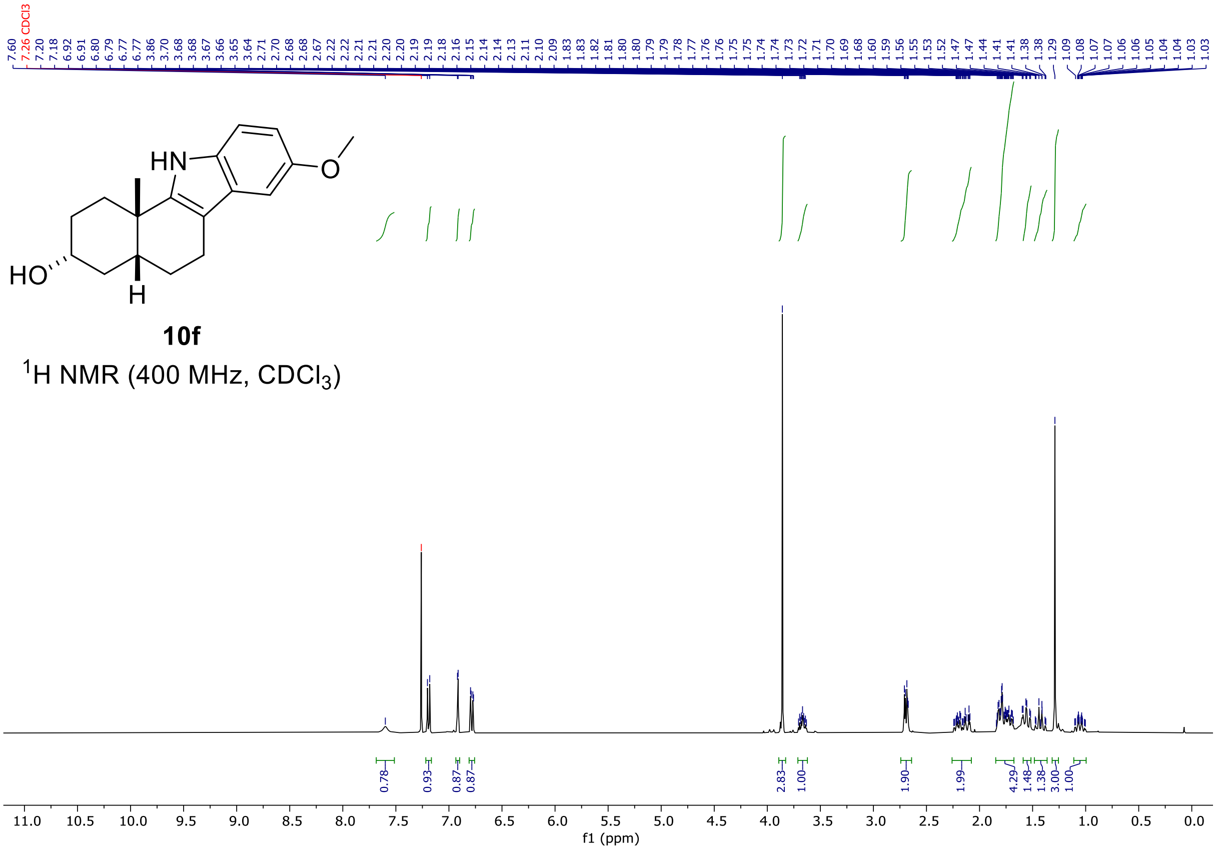

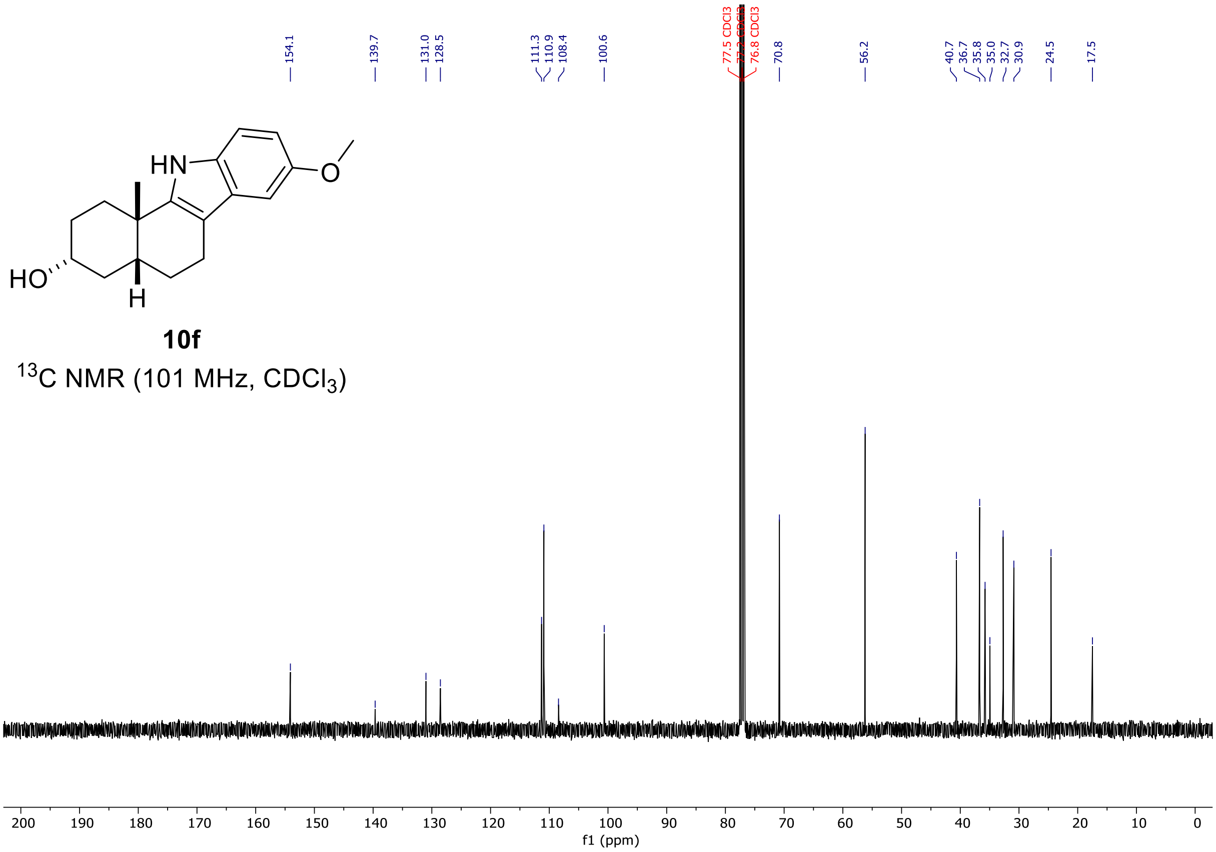

11b-Methyl-8-(trifluoromethyl)-2,3,4,4a,5,6,11,11b-octahydro-1*H*-benzo[*a*]carbazol-3-ol (10g)

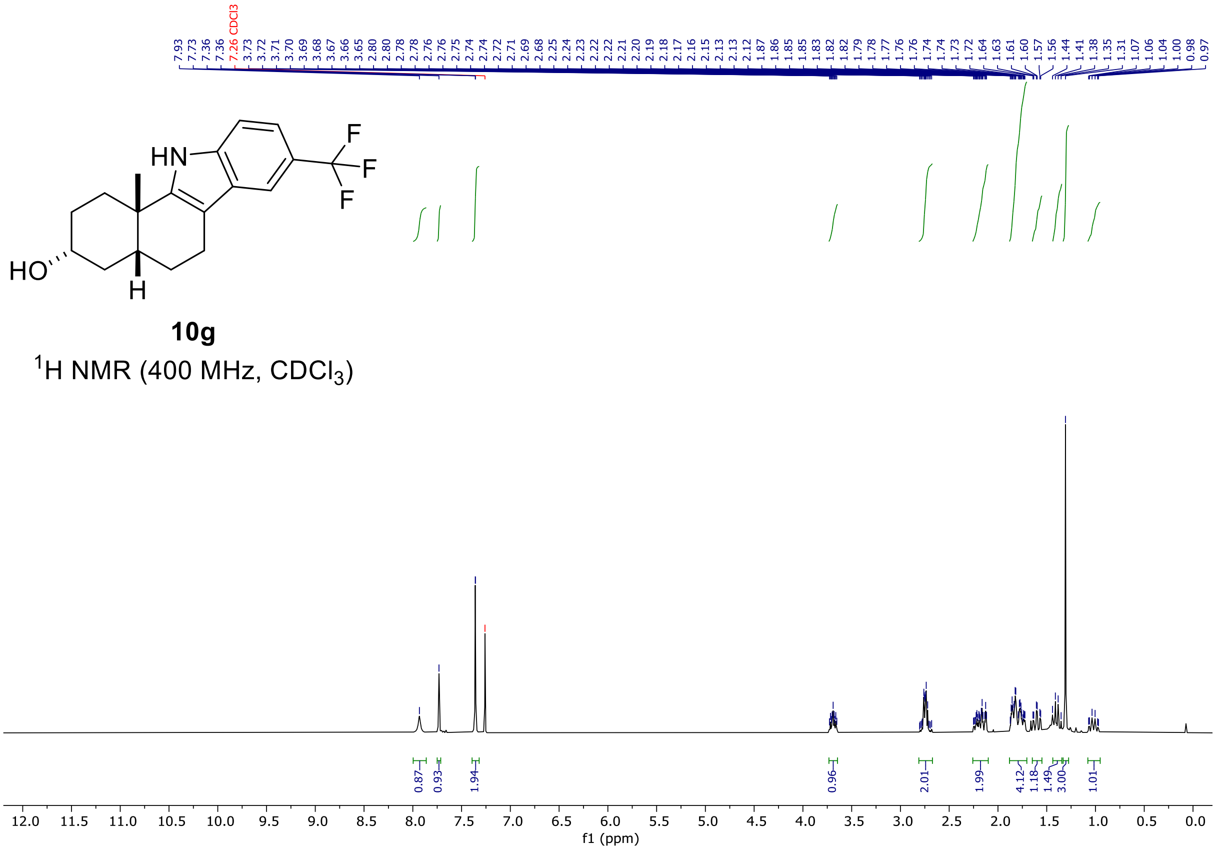

10g

^{1}H NMR (400 MHz, $CDCl_3$)

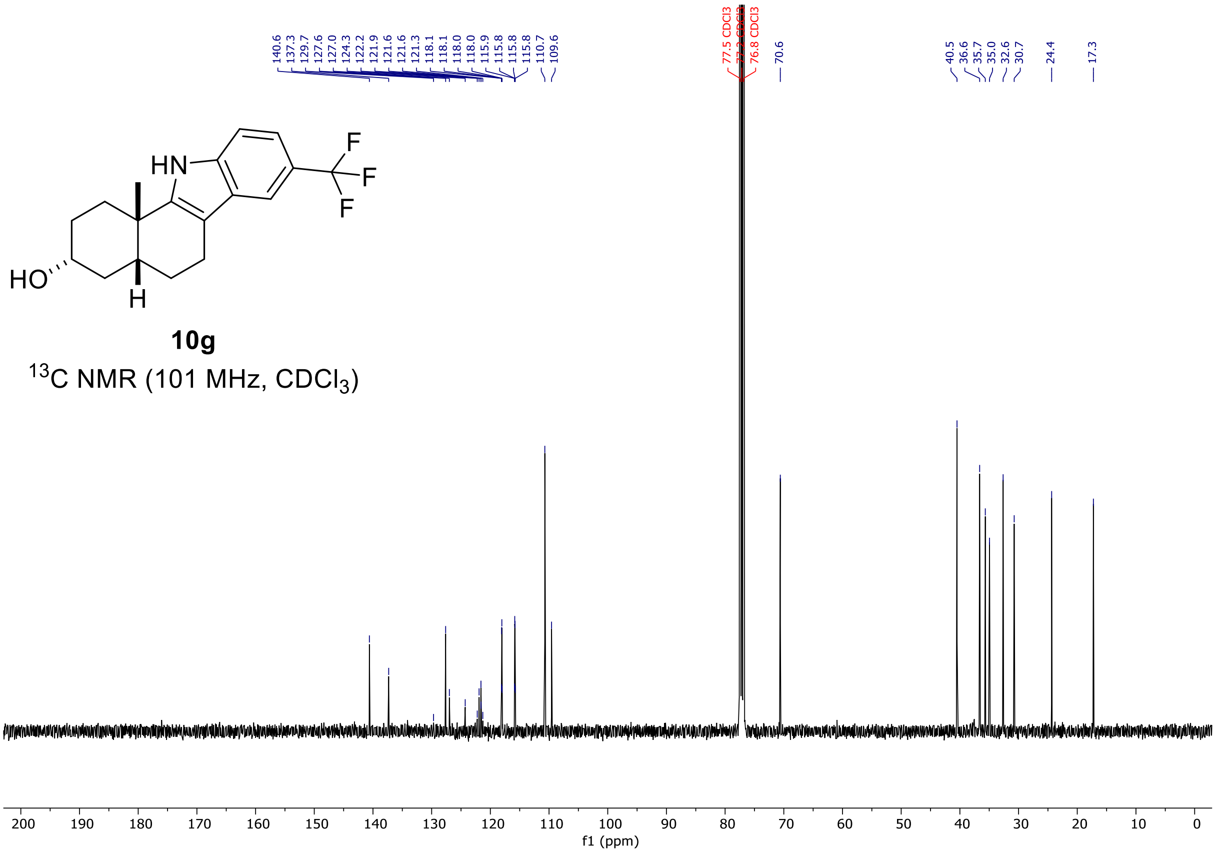

10g

^{13}C NMR (101 MHz, $CDCl_3$)

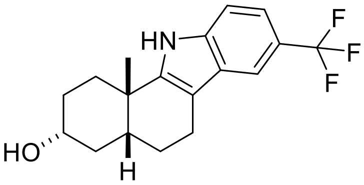

10g

^{19}F NMR (377 MHz, $CDCl_3$)

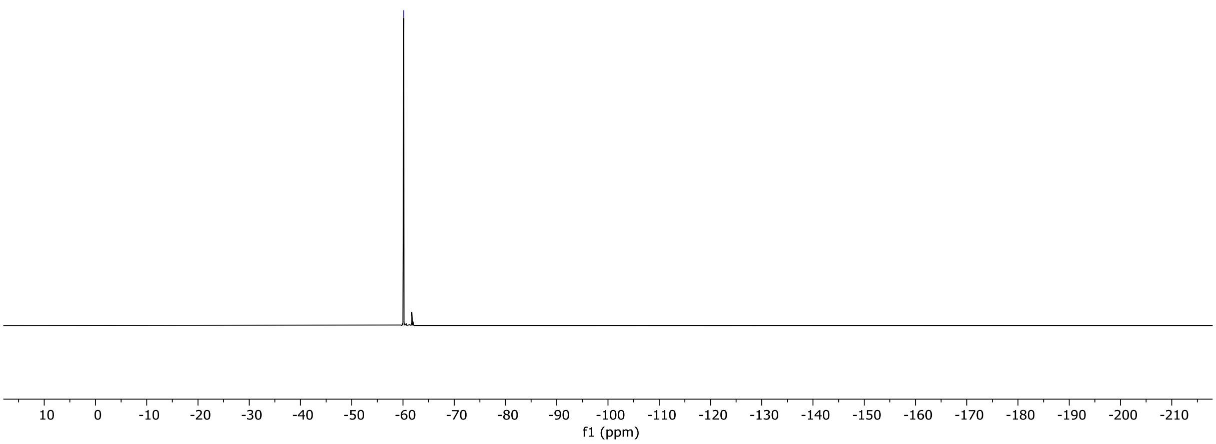

(3*R,4a*R**,11b*S**)-8-Iodo-11b-methyl-2,3,4,4^{a},5,6,11,11b-octahydro-1*H*-benzo[*a*]156arbazole-3-ol (10h)**

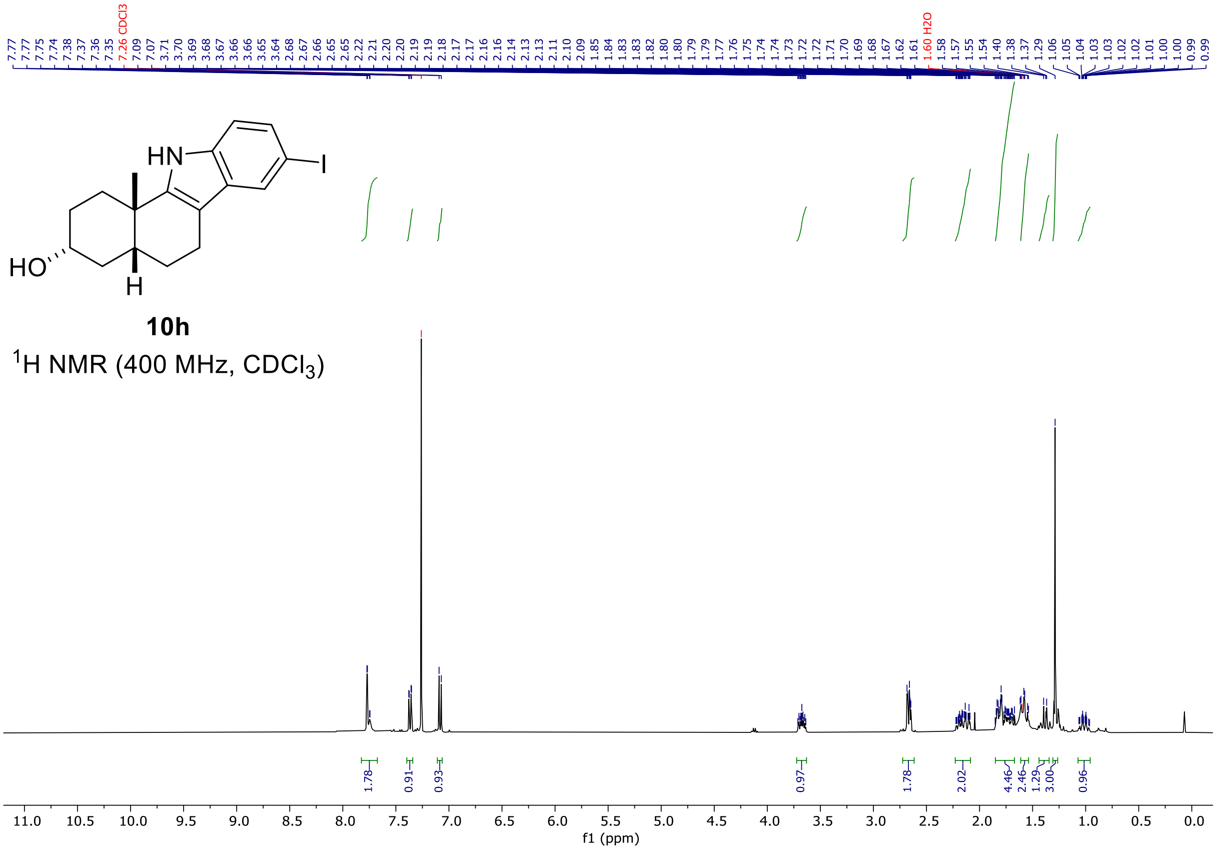

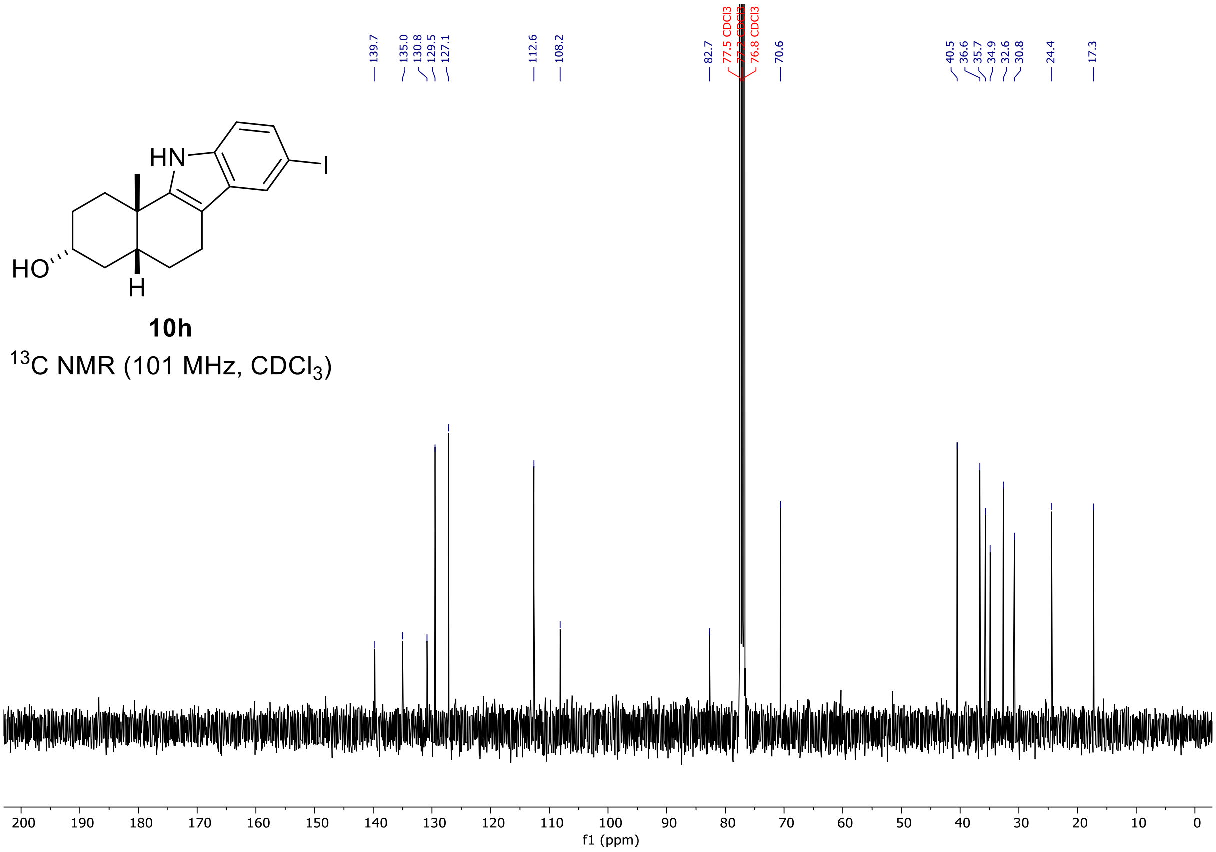

(3*R,4a*R**,11b*S**)-8-Chloro-10-fluoro-11b-methyl-2,3,4,4^{a},5,6,11,11b-octahydro-1*H*-benzo[*a*]157arbazole-3-ol (10i)**

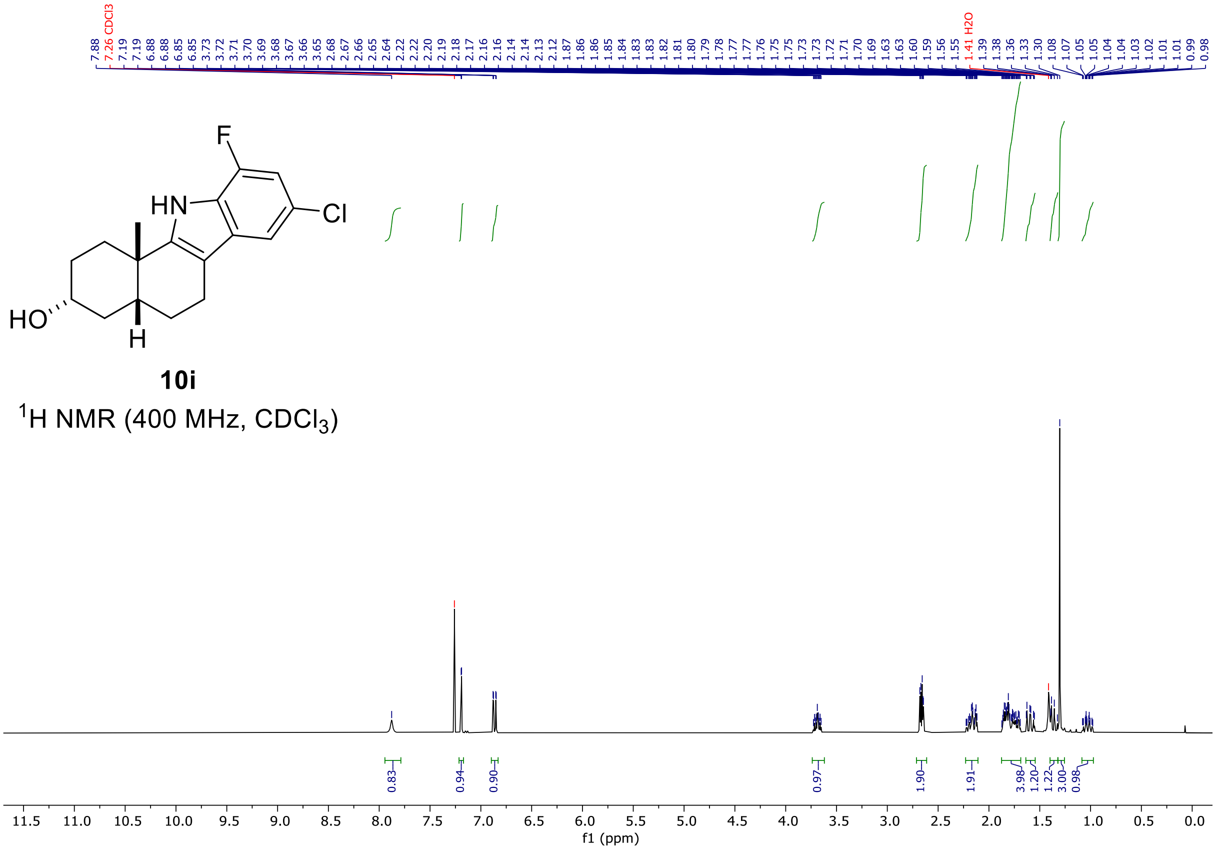

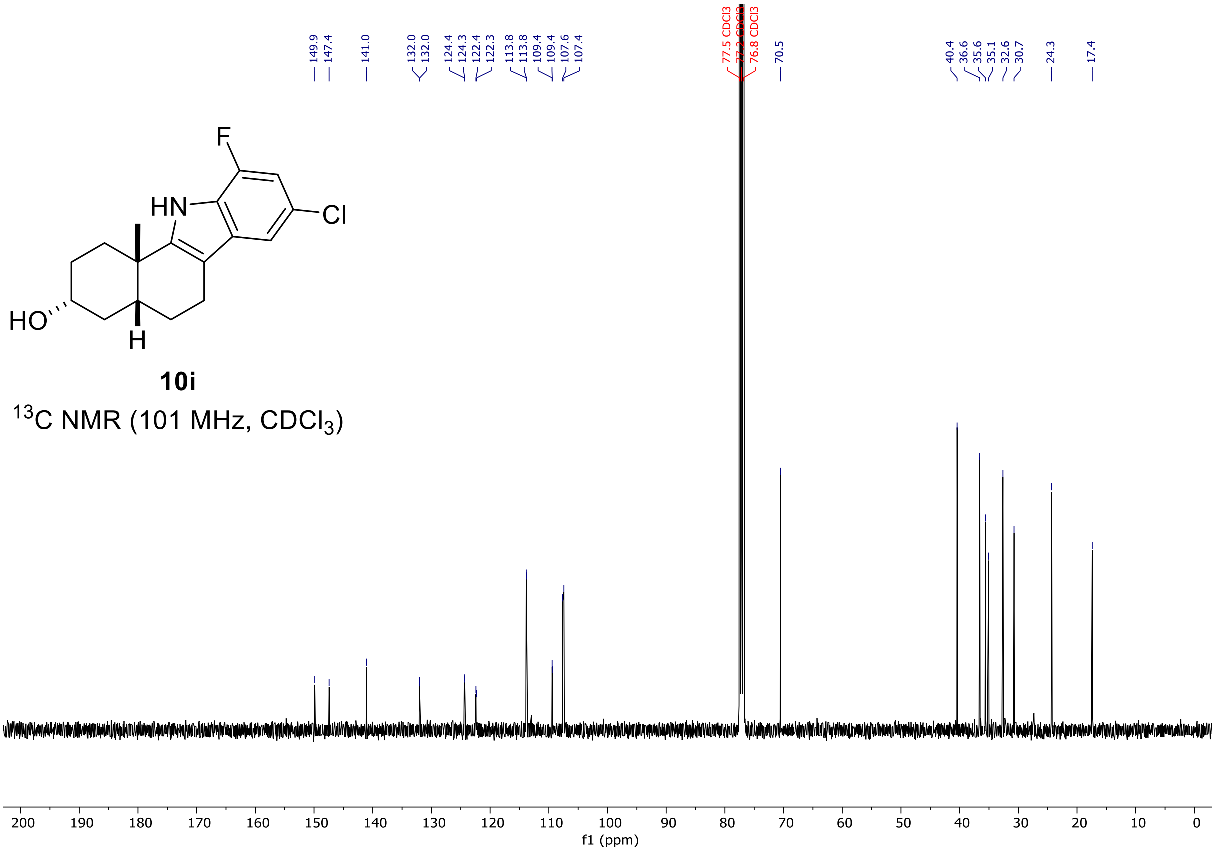

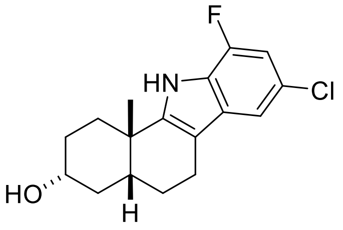

10i

^{19}F NMR (377 MHz, $CDCl_3$)

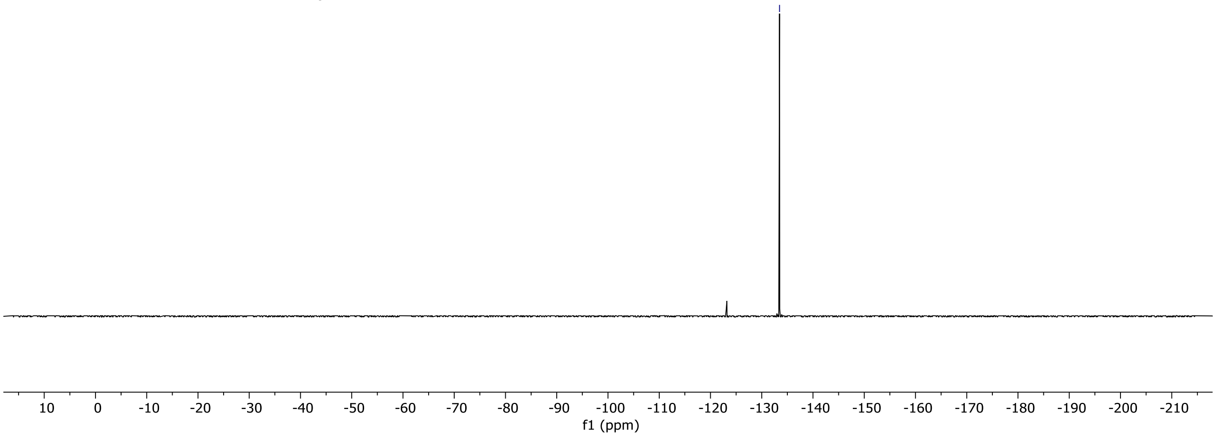

(3*R,4a*R**,11b*S**)-7,9,11b-Trimethyl-2,3,4,4a,5,6,11,11b-octahydro-1*H*-benzo[*a*]carbazol-3-ol (10j)**

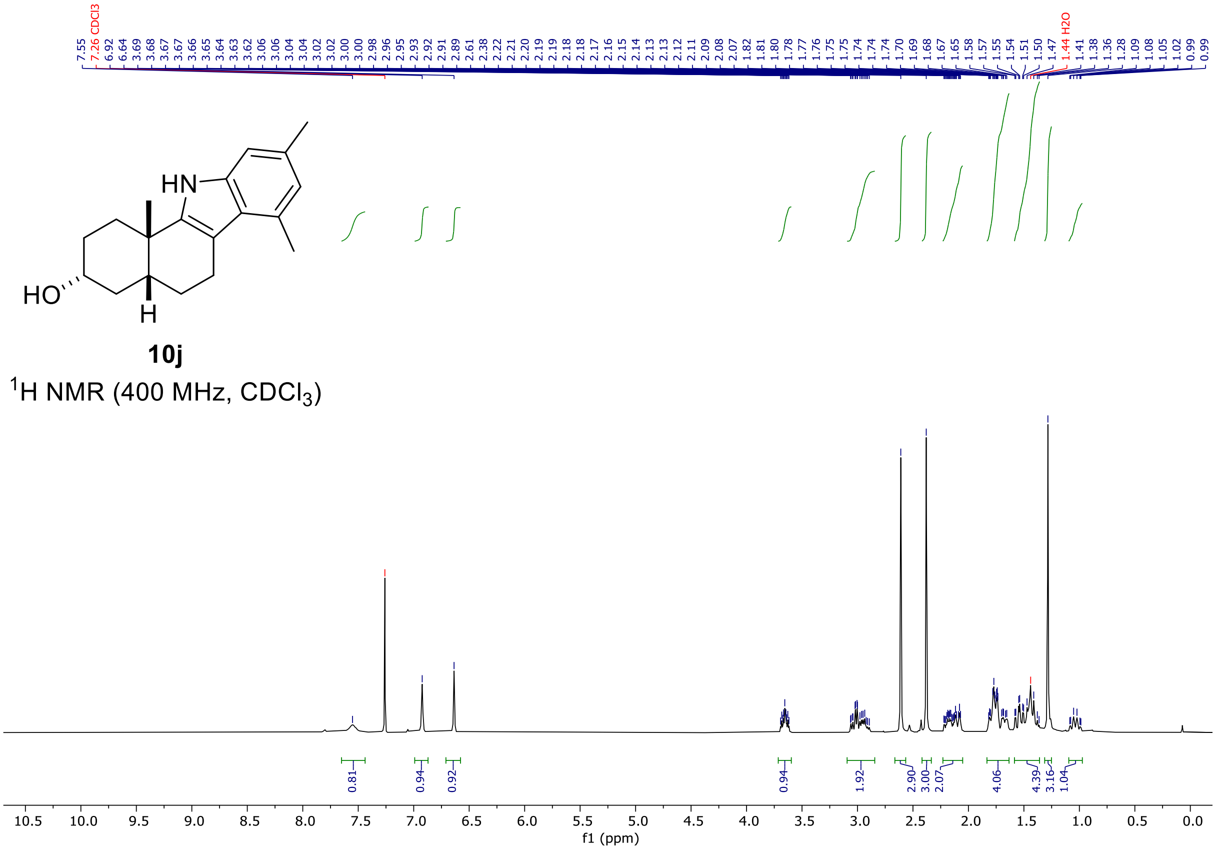

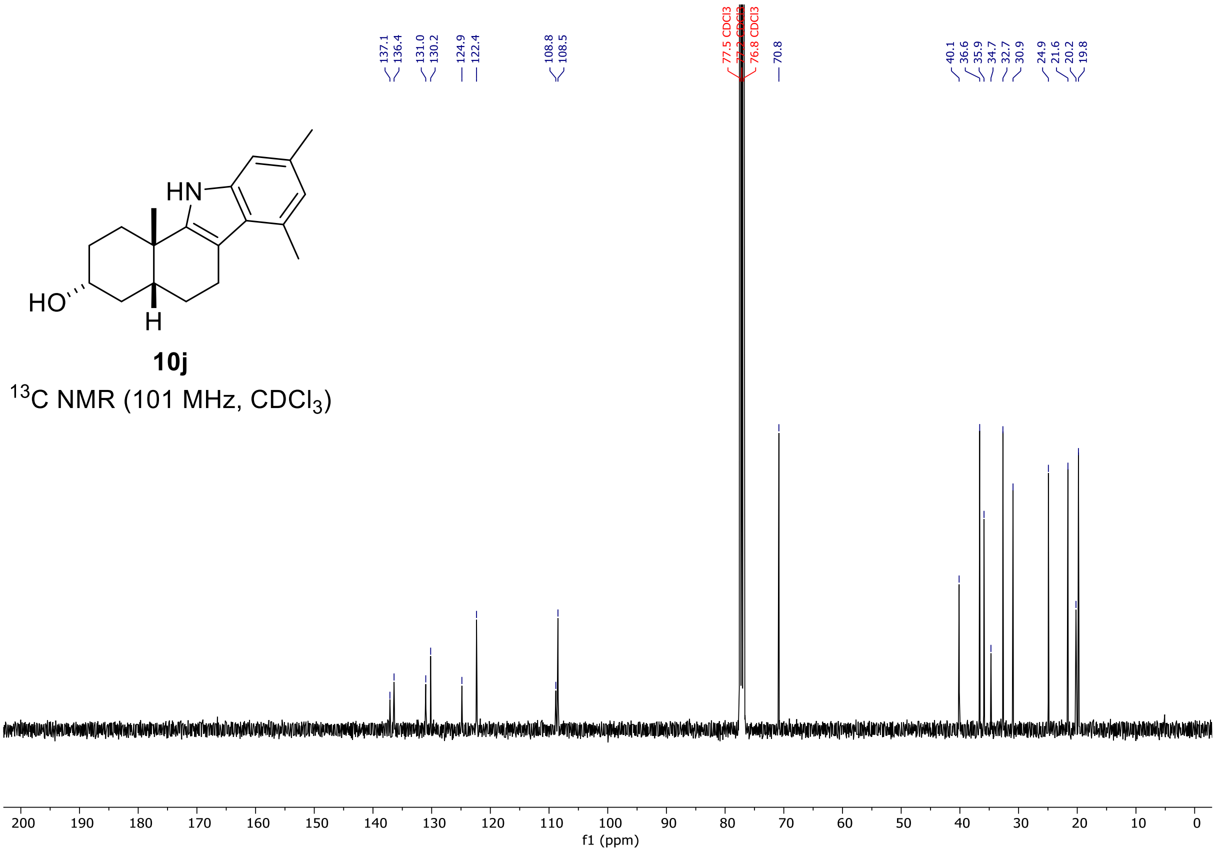

Ethyl (3*R,4a*R**,11b*S**)-3-hydroxy-11b-methyl-2,3,4,4a,5,6,11,11b-octahydro-1*H*-benzo[*a*]carbazole-8-carboxylate (10k)**

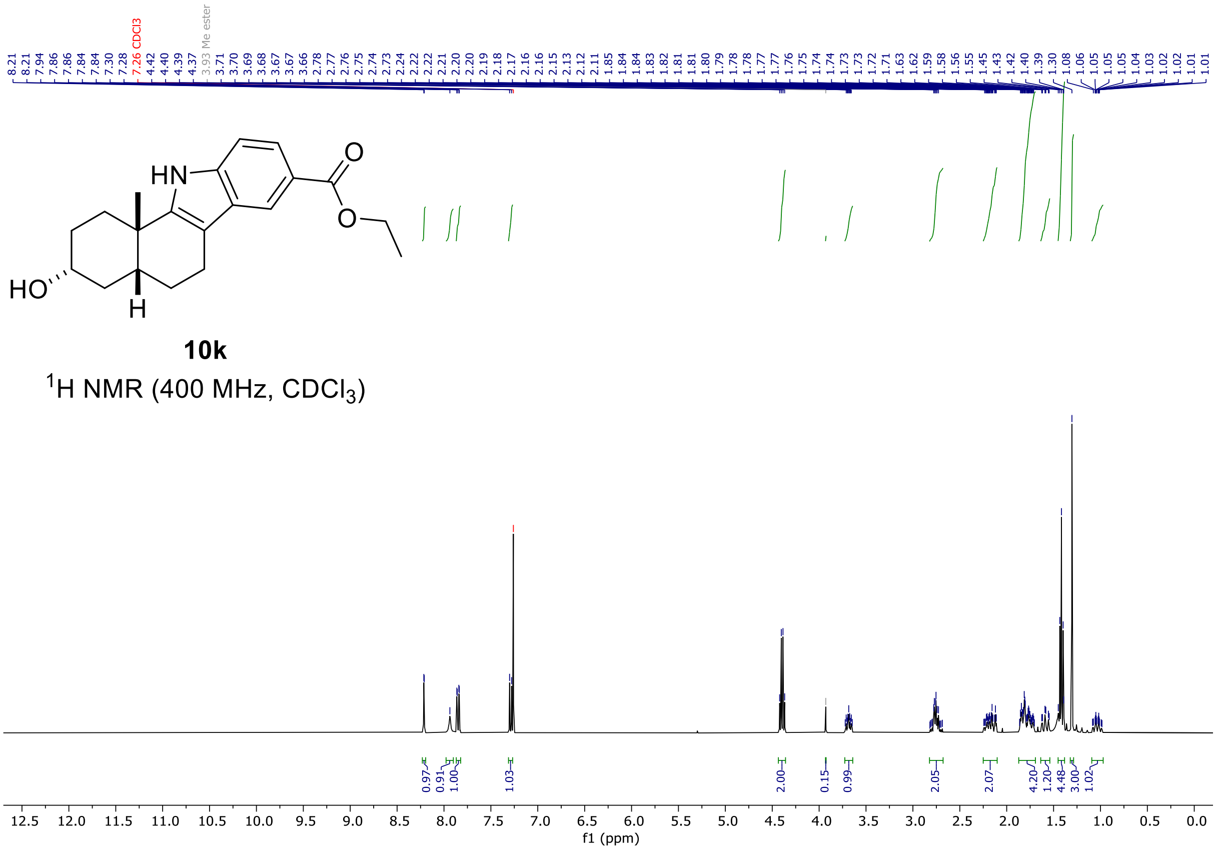

10k

^{1}H NMR (400 MHz, $CDCl_3$)

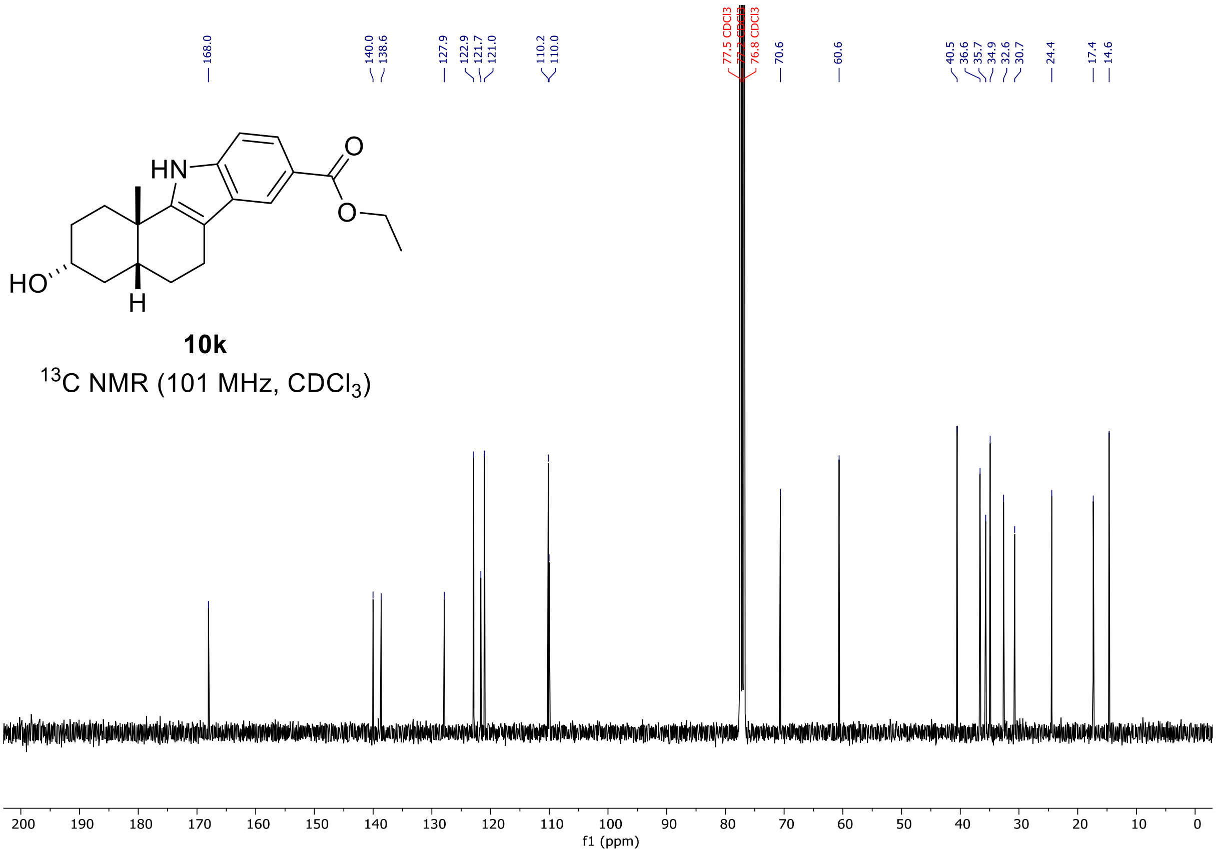

10k

^{13}C NMR (101 MHz, $CDCl_3$)

(6a*S,9*R**,10a*R**)-9-Hydroxy-6a-methyl-6a,7,8,9,10,10a,11,12-octahydro-5*H*-dibenzo[*b*,*g*]azonine-6,13-dione (11a)**

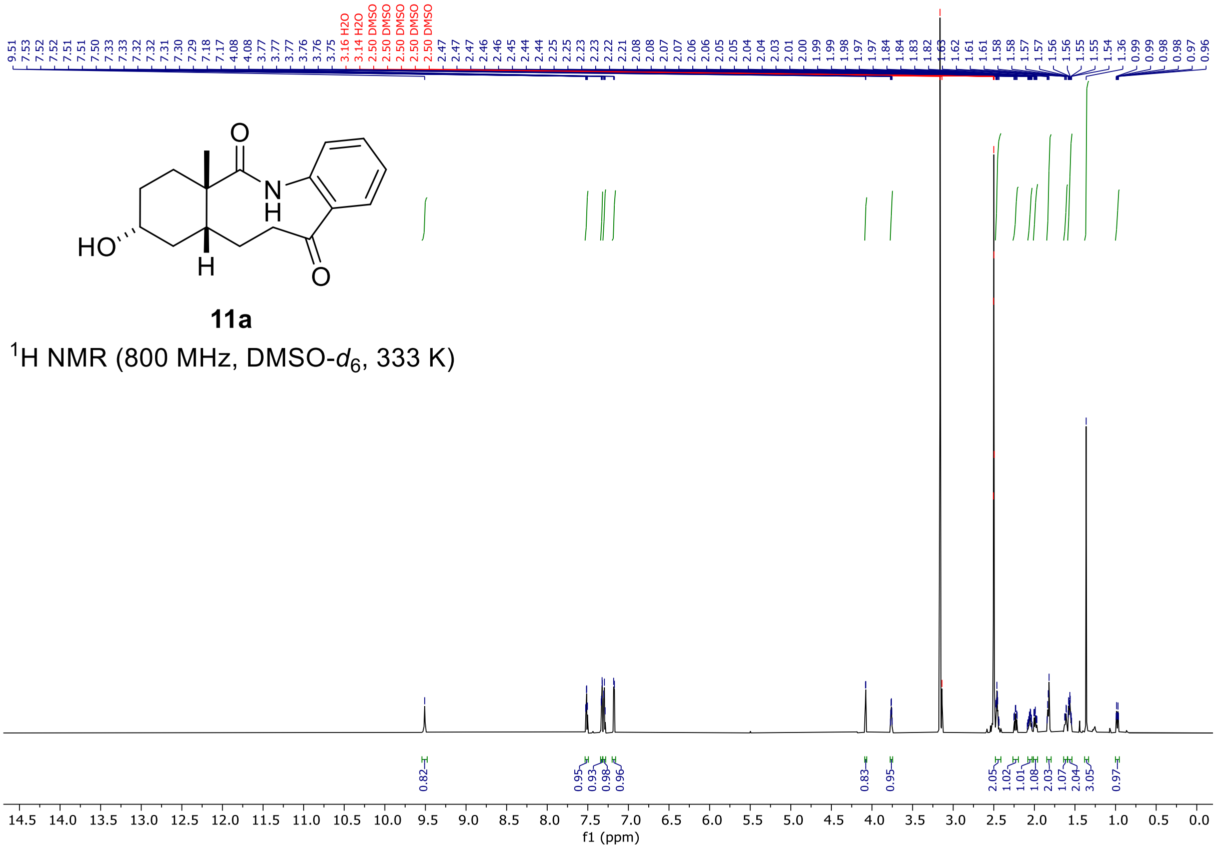

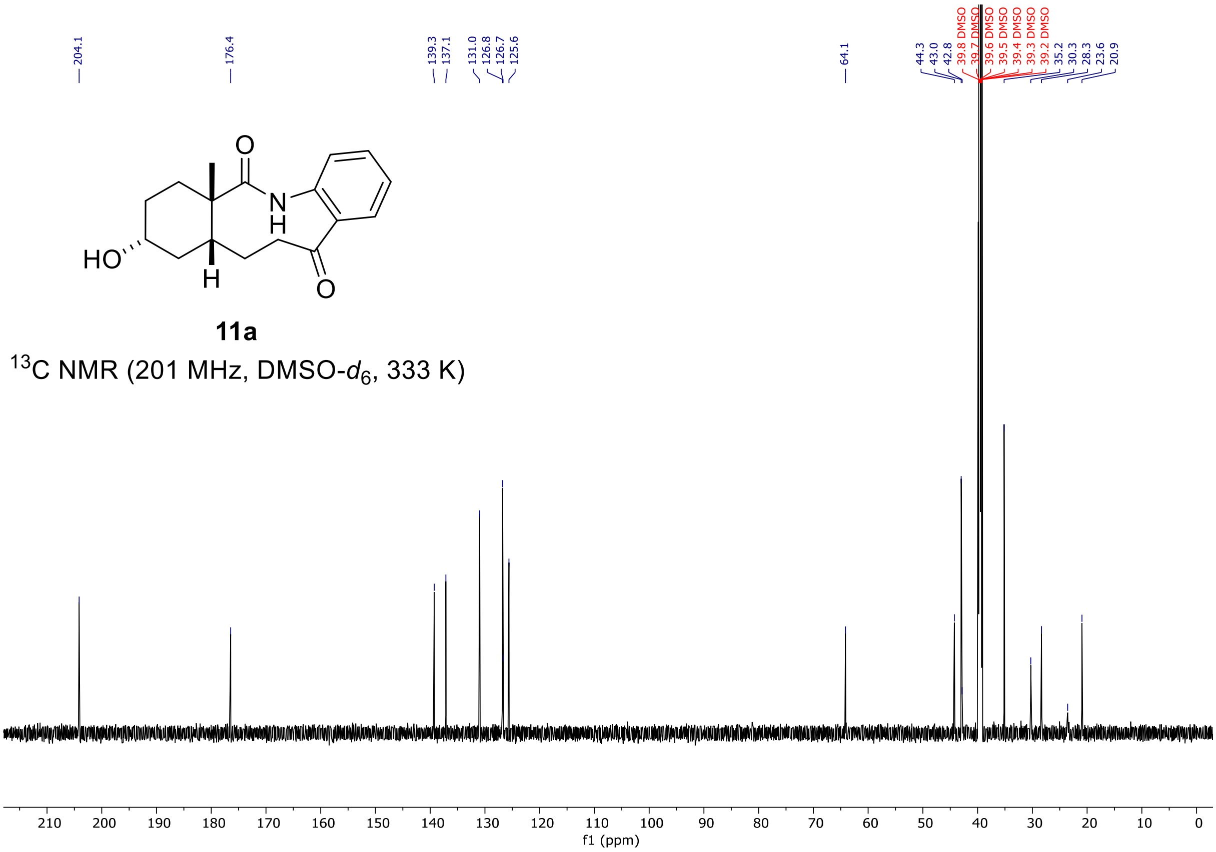

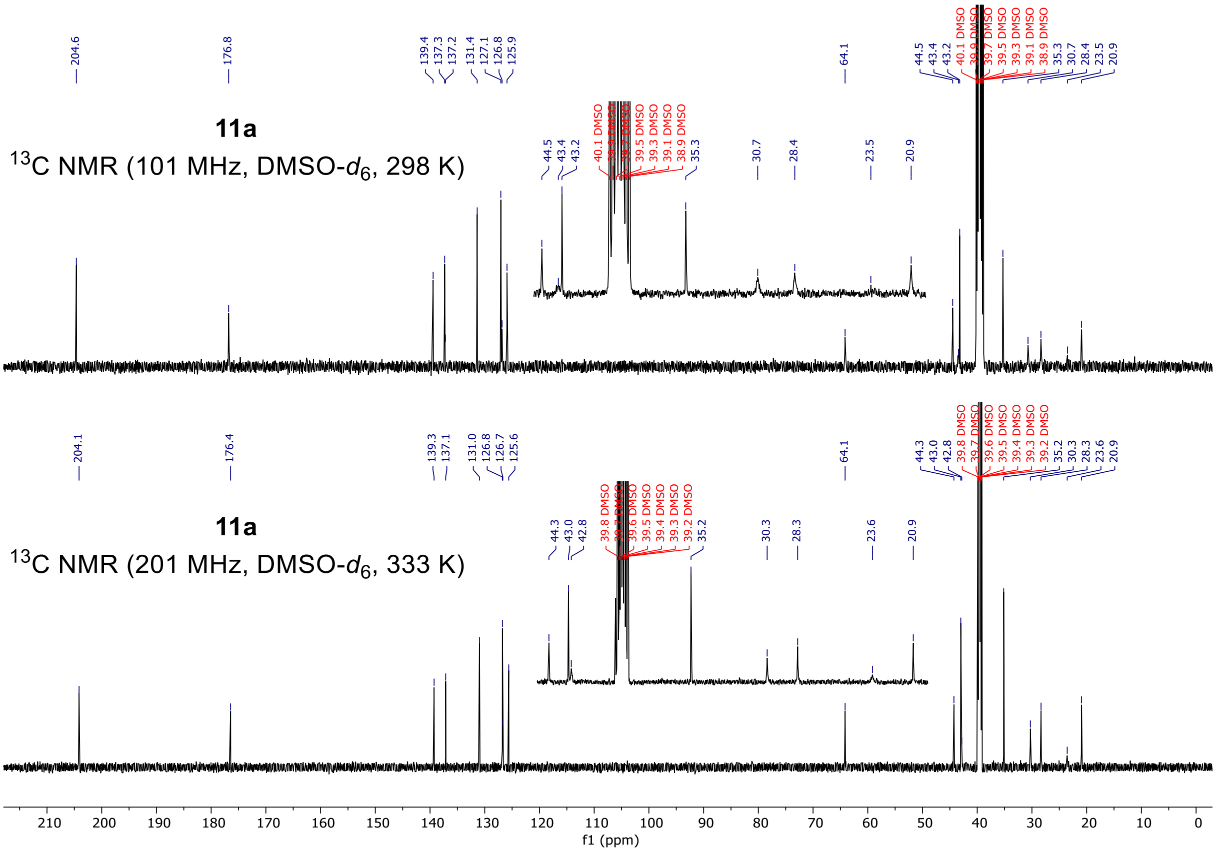
11a
^{13}C NMR (101 MHz, DMSO-d_6, 298 K)
11a
^{13}C NMR (201 MHz, DMSO-d_6, 333 K)
f1 (ppm)

(6a*S,9*R**,10a*R**)-2-Bromo-9-hydroxy-6a-methyl-6a,7,8,9,10,10a,11,12-octahydro-5*H*-dibenzo[*b*,*g*]azonine-6,13-dione (11b)**

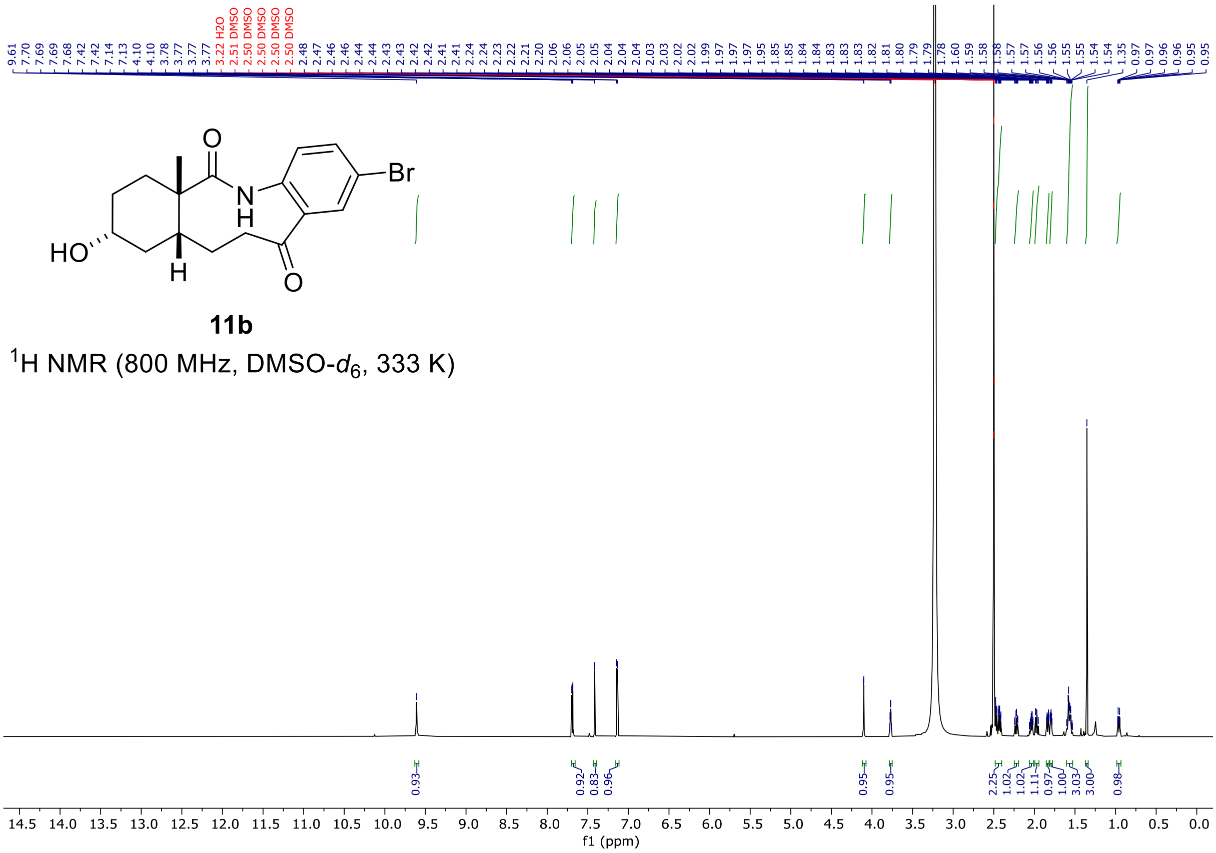

11b

^{1}H NMR (800 MHz, DMSO-d_6, 333 K)

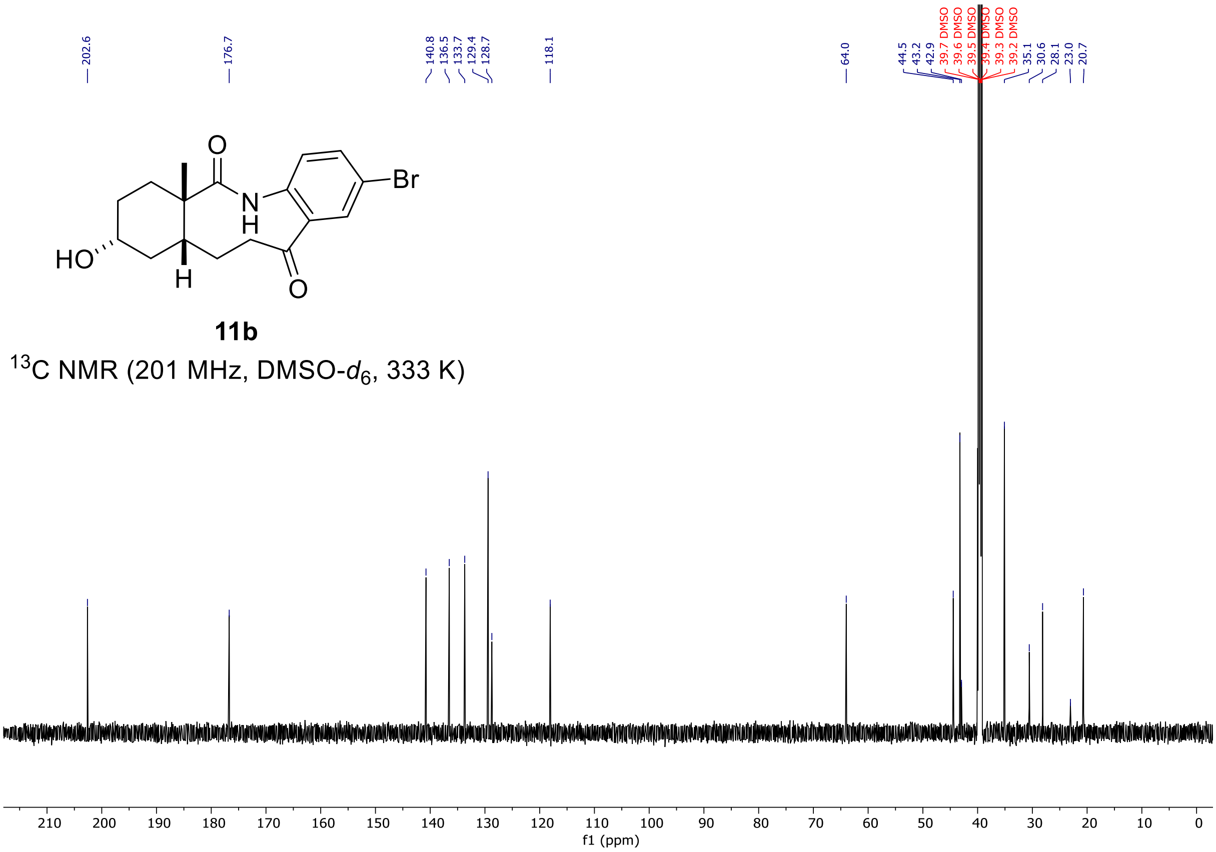

11b

^{13}C NMR (201 MHz, DMSO-d_6, 333 K)

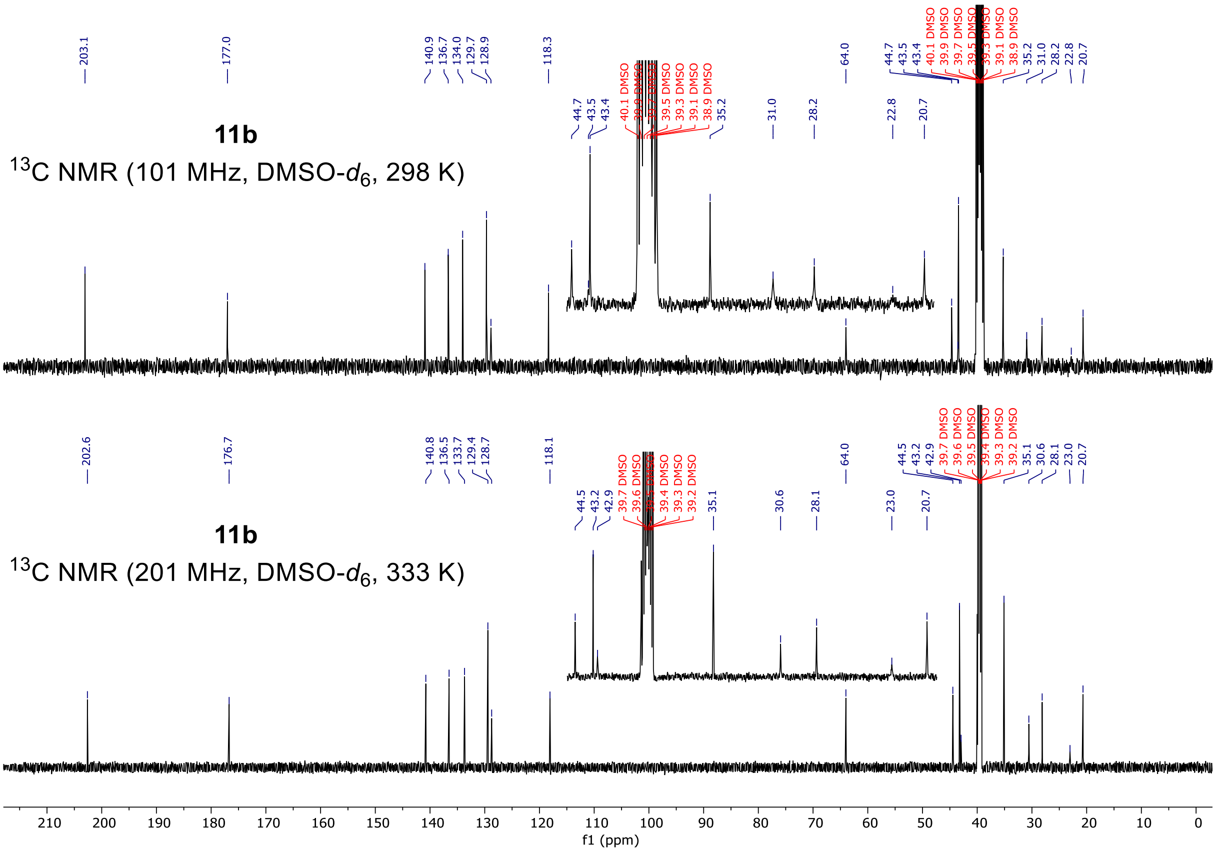

11b
^{13}C NMR (101 MHz, DMSO-d_6, 298 K)
11b
^{13}C NMR (201 MHz, DMSO-d_6, 333 K)
f1 (ppm)

(6a*S,9*R**,10a*R**)-9-Hydroxy-2,6a-dimethyl-6a,7,8,9,10,10a,11,12-octahydro-5*H*-dibenzo[*b*,*g*]azonine-6,13-dione (11c)**

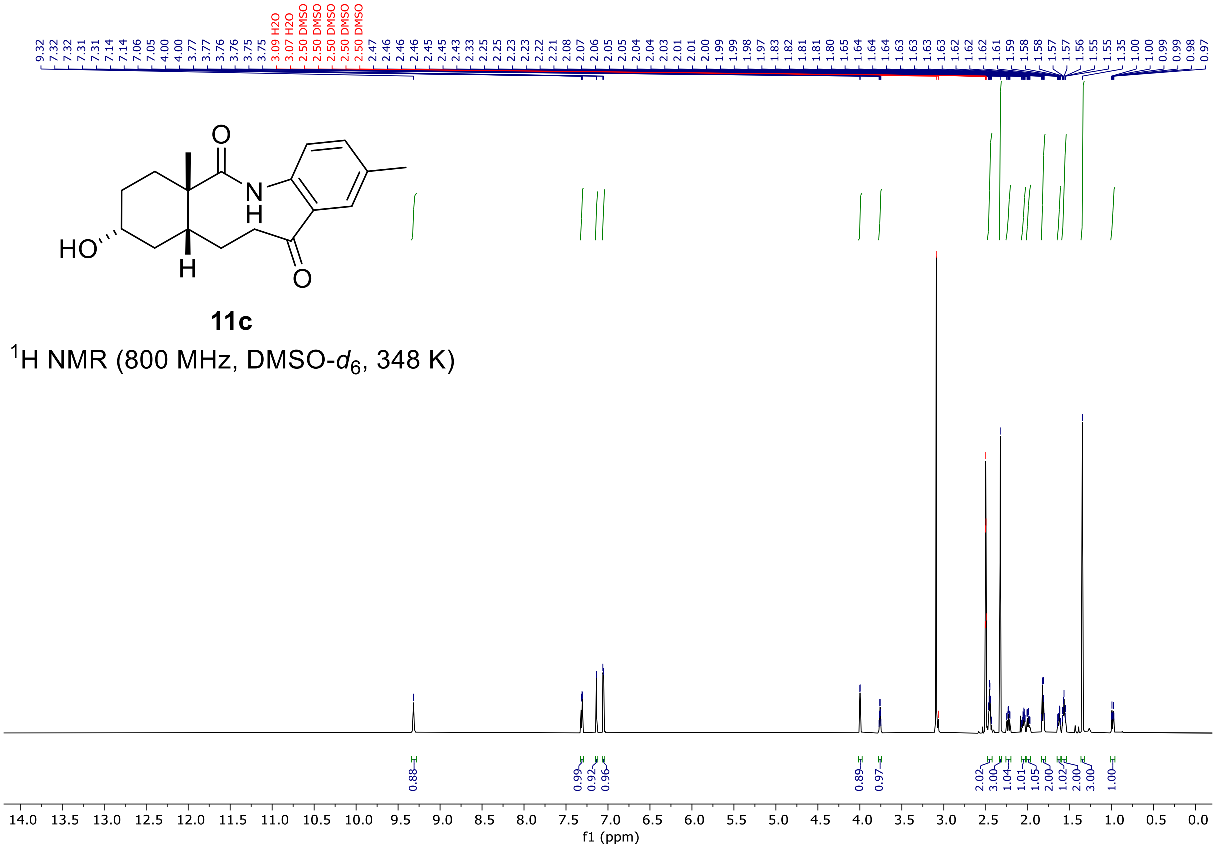

11c

^{1}H NMR (800 MHz, DMSO-d_6, 348 K)

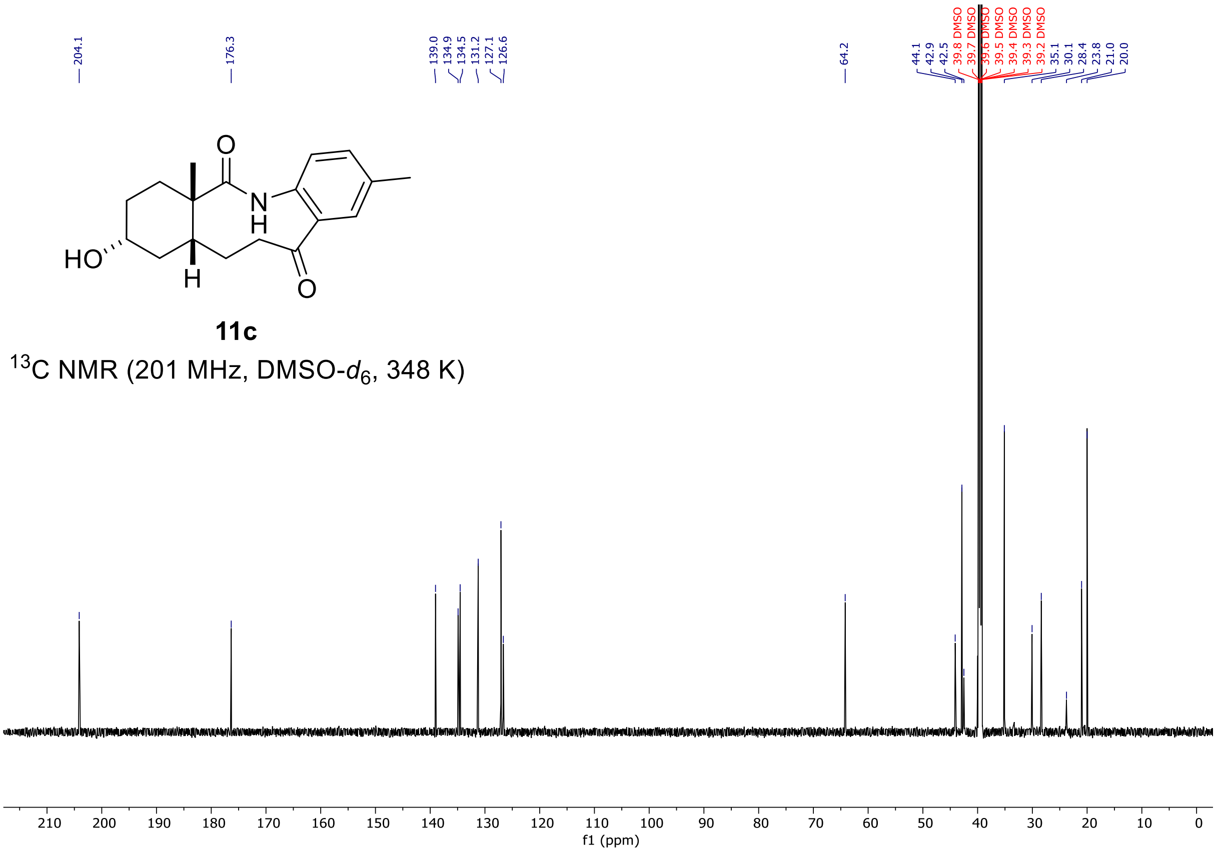

11c

^{13}C NMR (201 MHz, DMSO-d_6, 348 K)

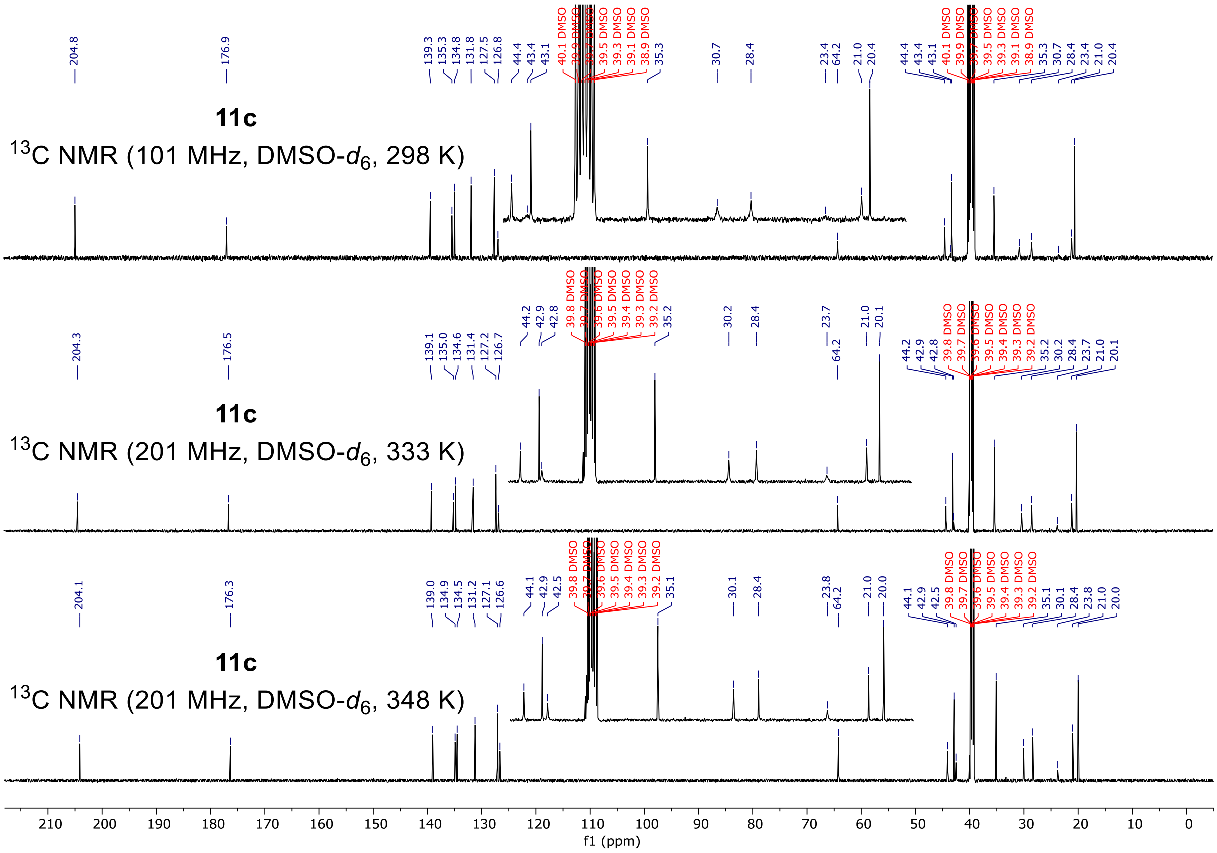
11c
^{13}C NMR (101 MHz, DMSO-d_6, 298 K)
11c
^{13}C NMR (201 MHz, DMSO-d_6, 333 K)
11c
^{13}C NMR (201 MHz, DMSO-d_6, 348 K)
f1 (ppm)

(6a*S,9*R**,10a*R**)-9-Hydroxy-2-methoxy-6a-methyl-6a,7,8,9,10,10a,11,12-octahydro-5*H*-dibenzo[*b*,*g*]azonine-6,13-dione (11d)**

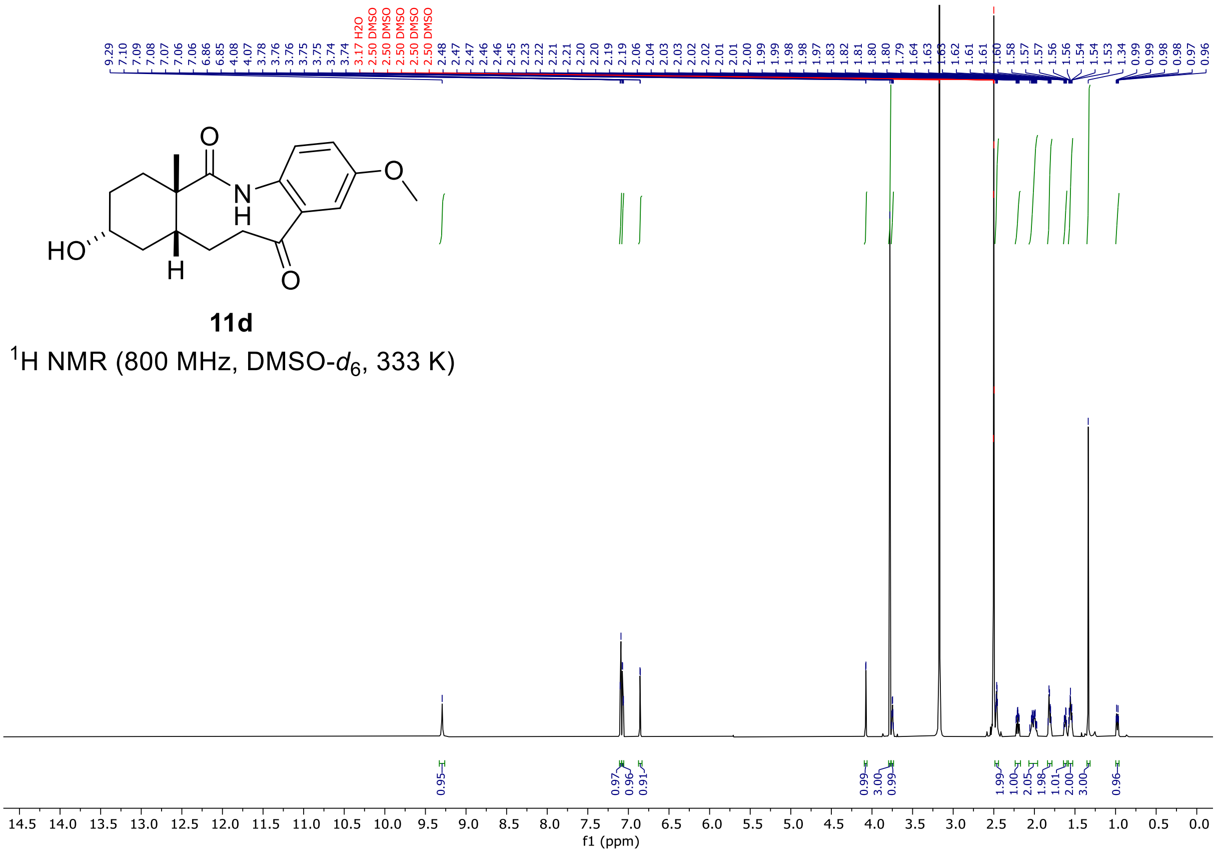

11d

^{1}H NMR (800 MHz, DMSO-d_6, 333 K)

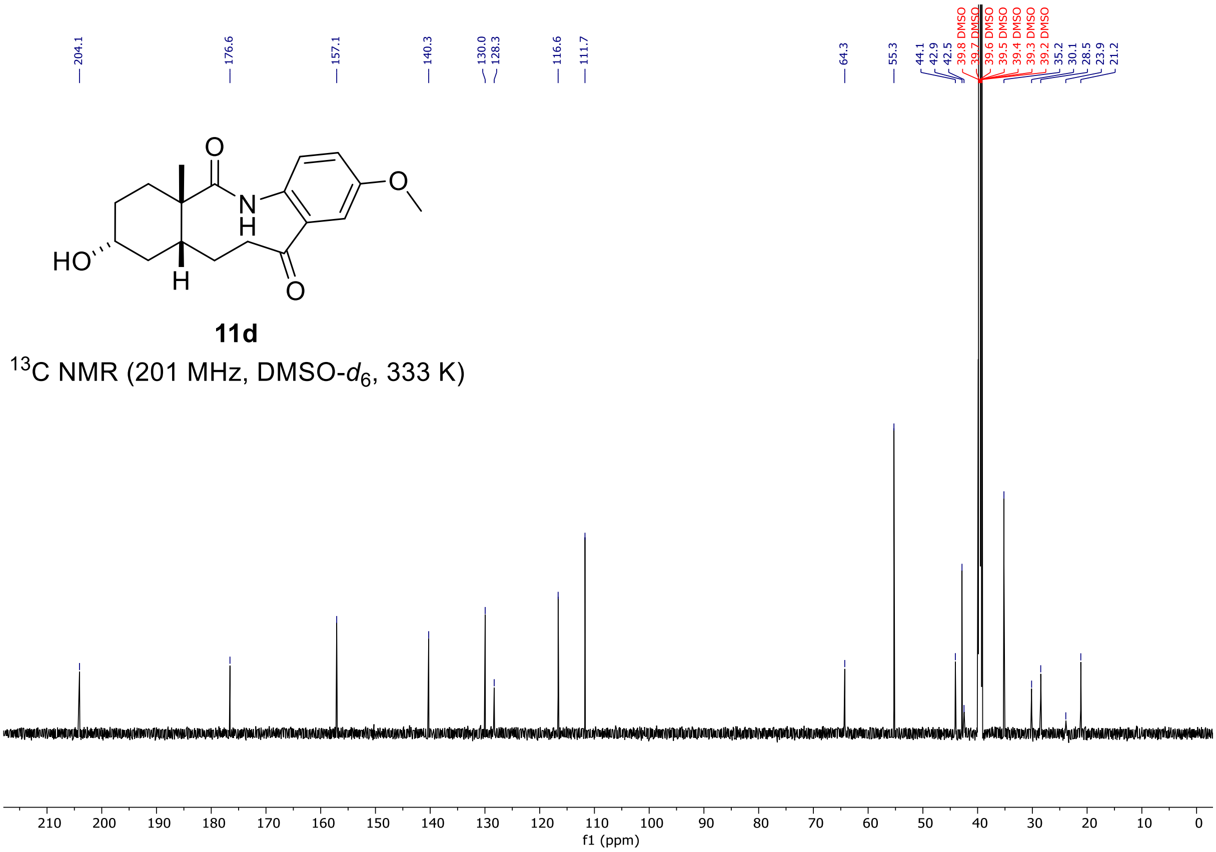

11d

^{13}C NMR (201 MHz, DMSO-d_6, 333 K)

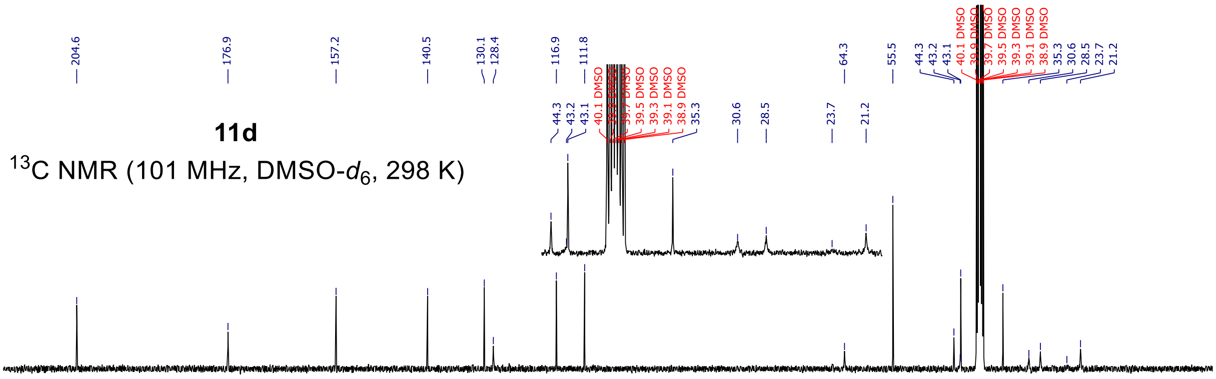

11d
13C NMR (101 MHz, DMSO-d6, 298 K)
204.6
176.9
157.2
140.5
130.1
128.4
116.9
111.8
64.3
55.5
44.3
43.2
43.1
40.1 DMSO
39.9 DMSO
39.7 DMSO
39.5 DMSO
39.3 DMSO
39.1 DMSO
38.9 DMSO
35.3
30.6
28.5
23.7
21.2

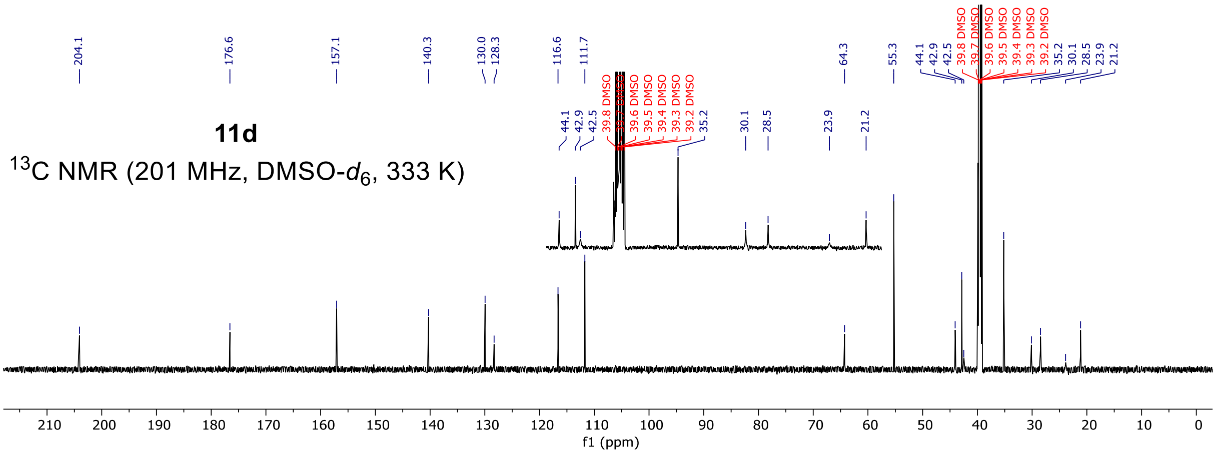

11d
13C NMR (201 MHz, DMSO-d6, 333 K)
204.1
176.6
157.1
140.3
130.0
128.3
116.6
111.7
64.3
55.3
44.1
42.9
42.5
39.8 DMSO
39.7 DMSO
39.6 DMSO
39.5 DMSO
39.4 DMSO
39.3 DMSO
39.2 DMSO
35.2
30.1
28.5
23.9
21.2
210 200 190 180 170 160 150 140 130 120 110 100 90 80 70 60 50 40 30 20 10 0
f1 (ppm)

(6a*S,9*R**,10a*R**)-9-Hydroxy-6a-methyl-2-(trifluoromethyl)-6a,7,8,9,10,10a,11,12-octahydro-5*H*-dibenzo[*b*,*g*]azonine-6,13-dione (11e)**

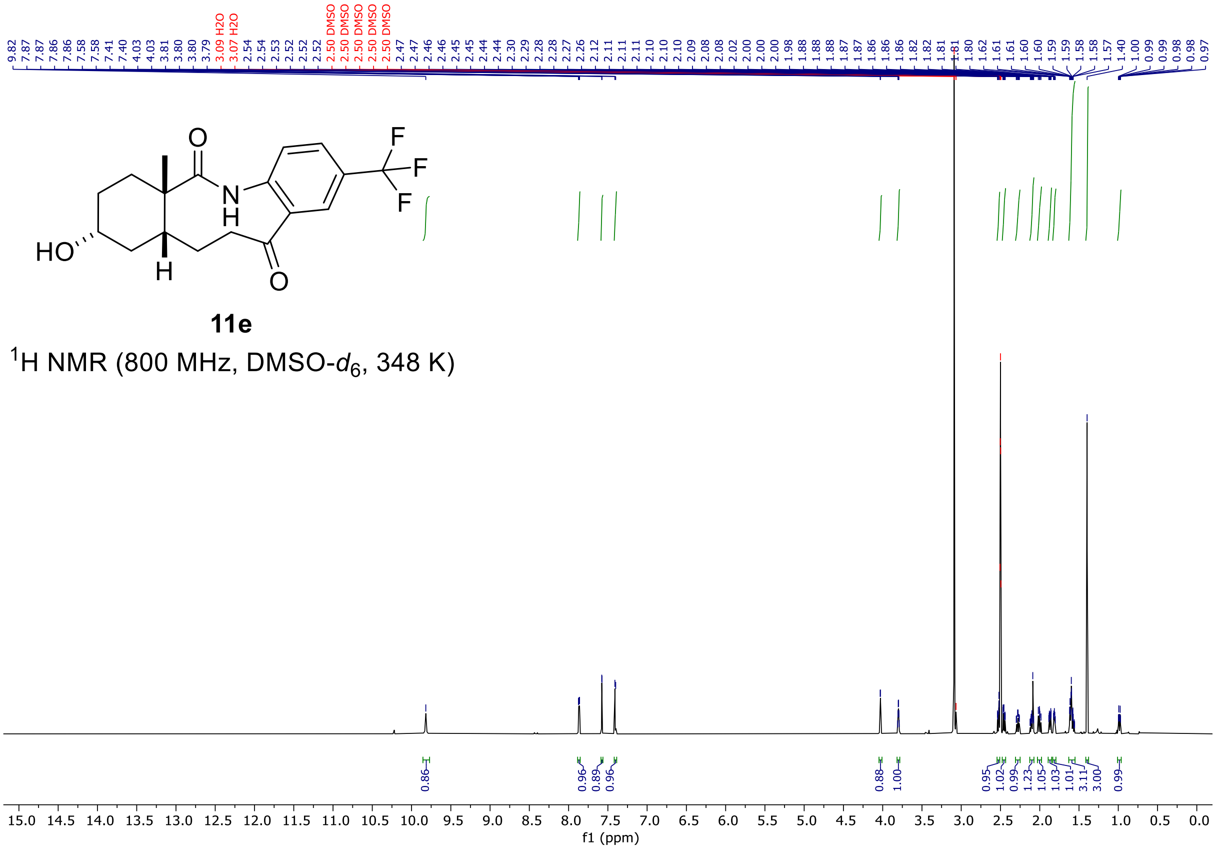

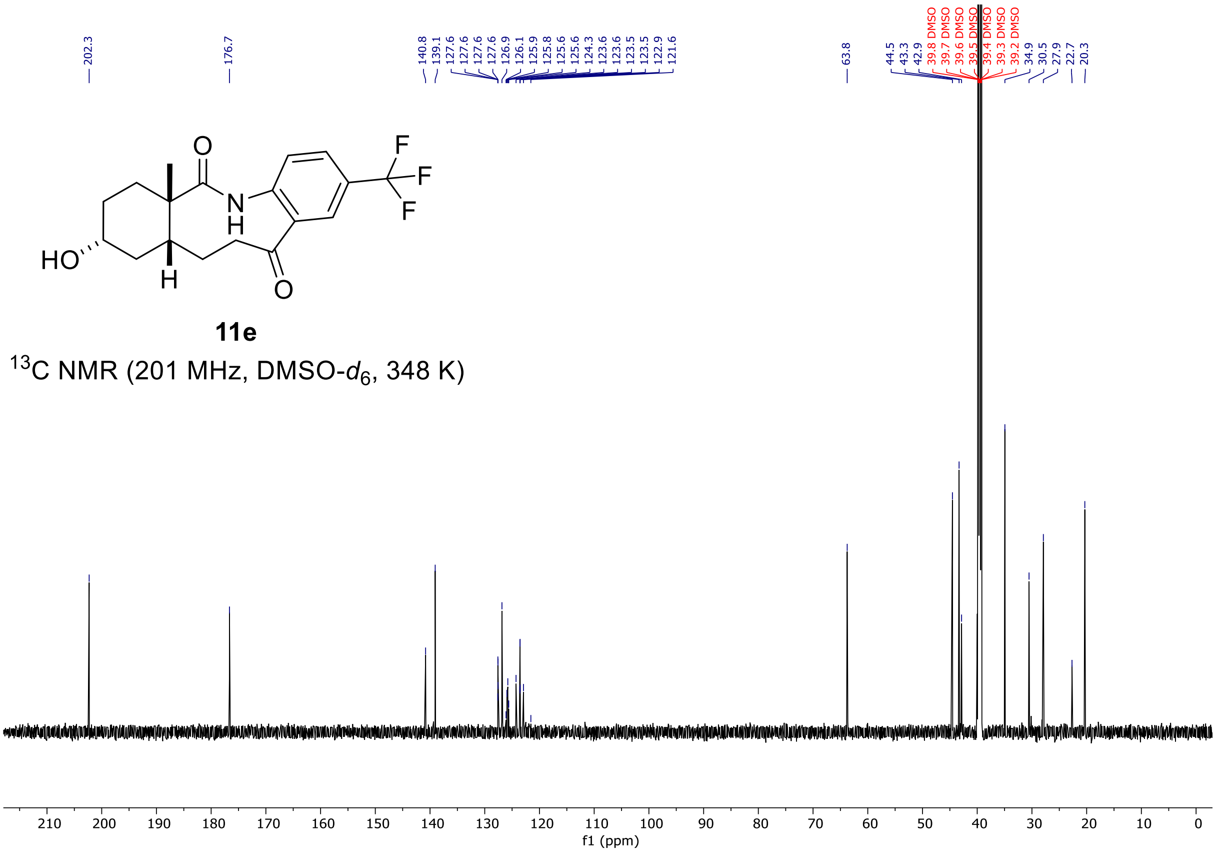

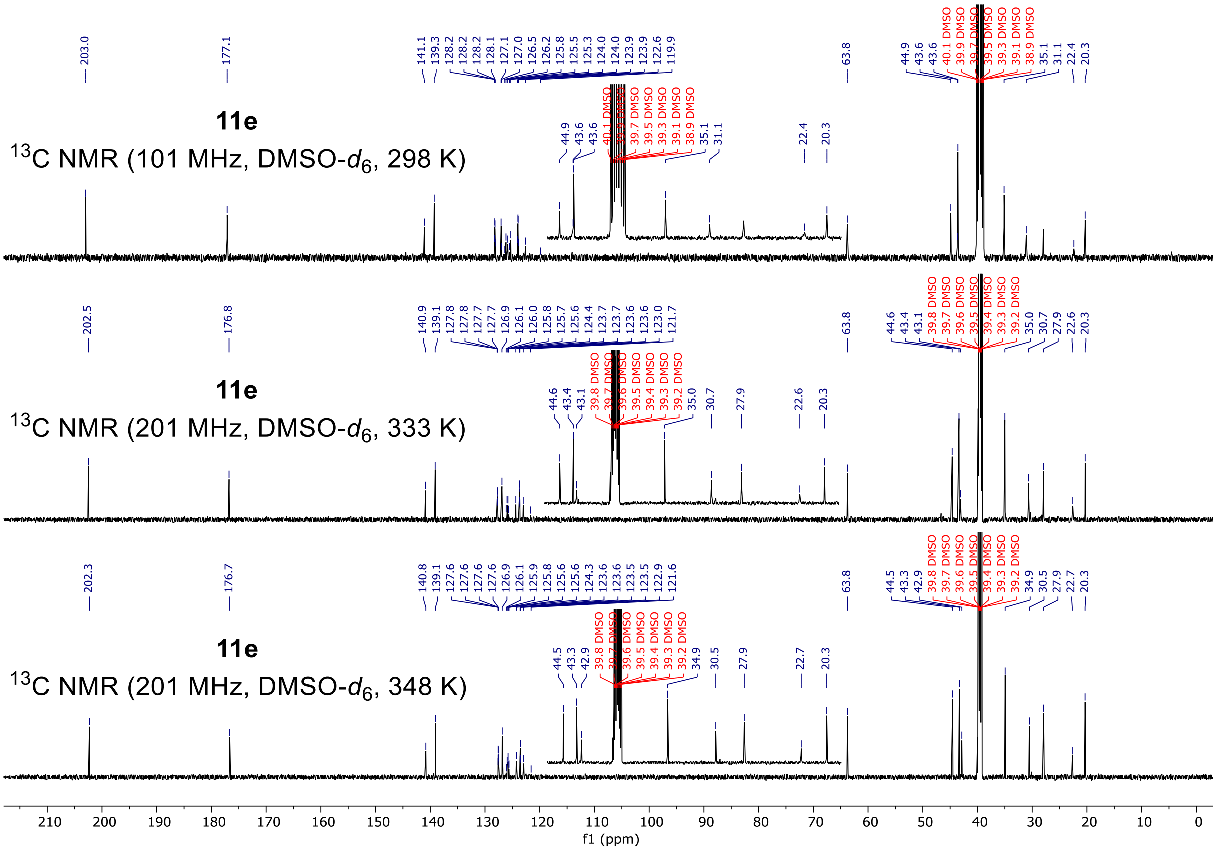
11e
^{13}C NMR (101 MHz, DMSO-d_6, 298 K)
11e
^{13}C NMR (201 MHz, DMSO-d_6, 333 K)
11e
^{13}C NMR (201 MHz, DMSO-d_6, 348 K)
f1 (ppm)

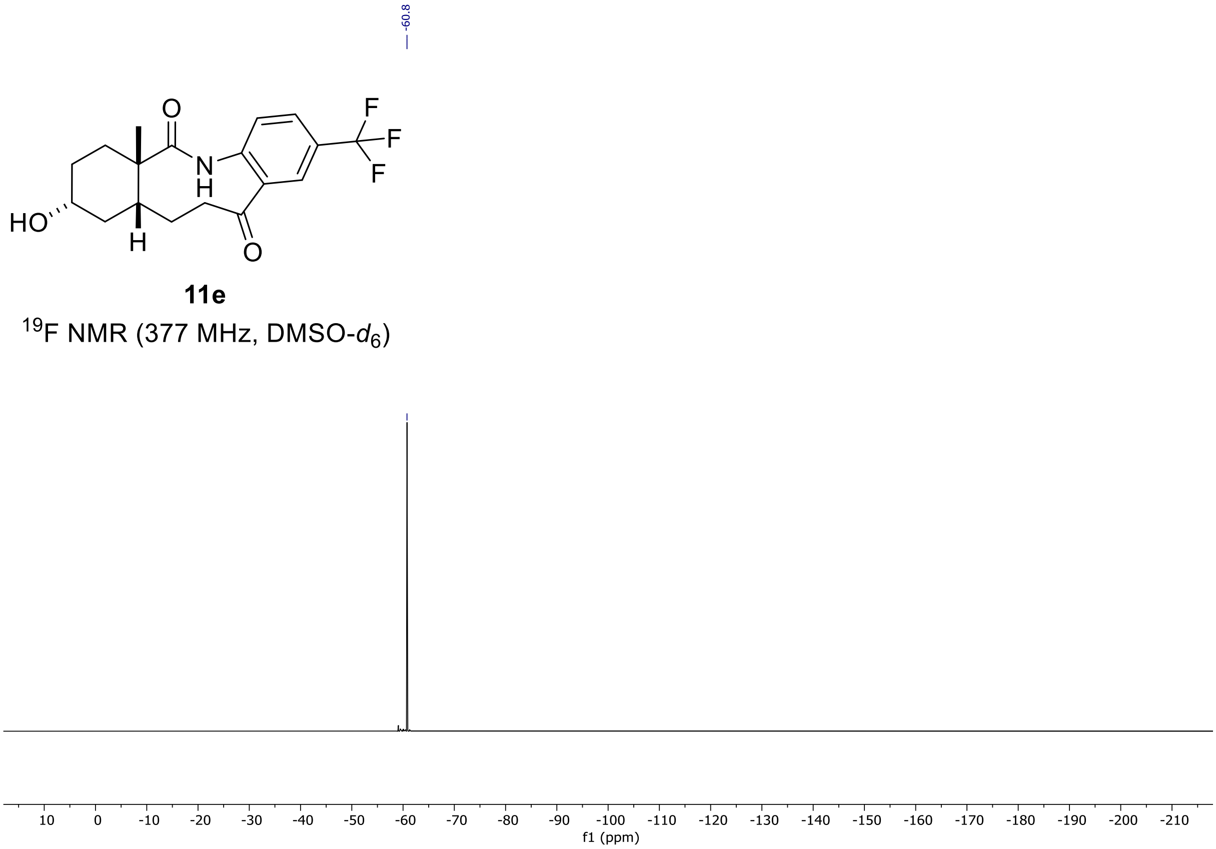
-60.8
HO
H
N
H
O
O
F
F
F
11e
^{19}F NMR (377 MHz, DMSO-d_6)
f1 (ppm)

(6a*S,9*R**,10a*R**)-2-Chloro-4-fluoro-9-hydroxy-6a-methyl-6a,7,8,9,10,10a,11,12-octahydro-5*H*-dibenzo[*b*,*g*]azonine-6,13-dione (11f)**

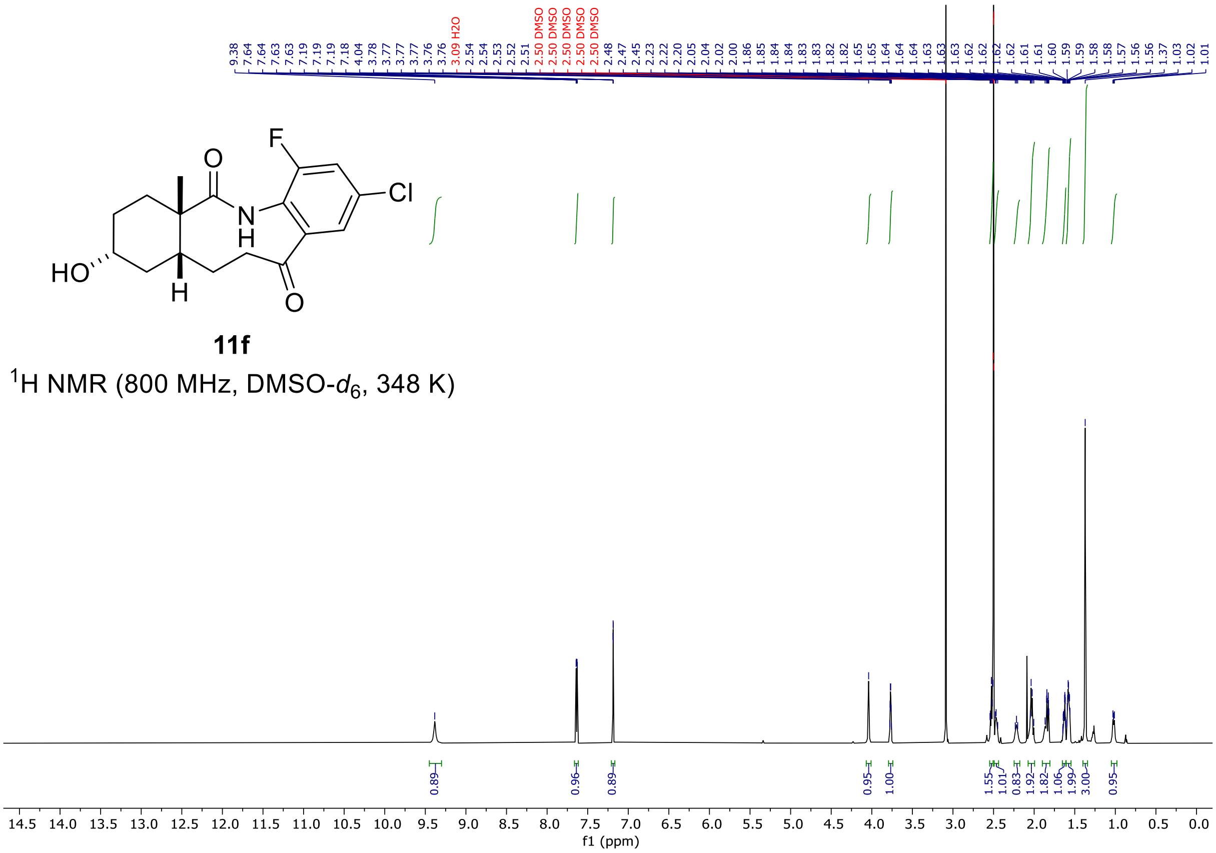

11f

^{1}H NMR (800 MHz, DMSO-d_6, 348 K)

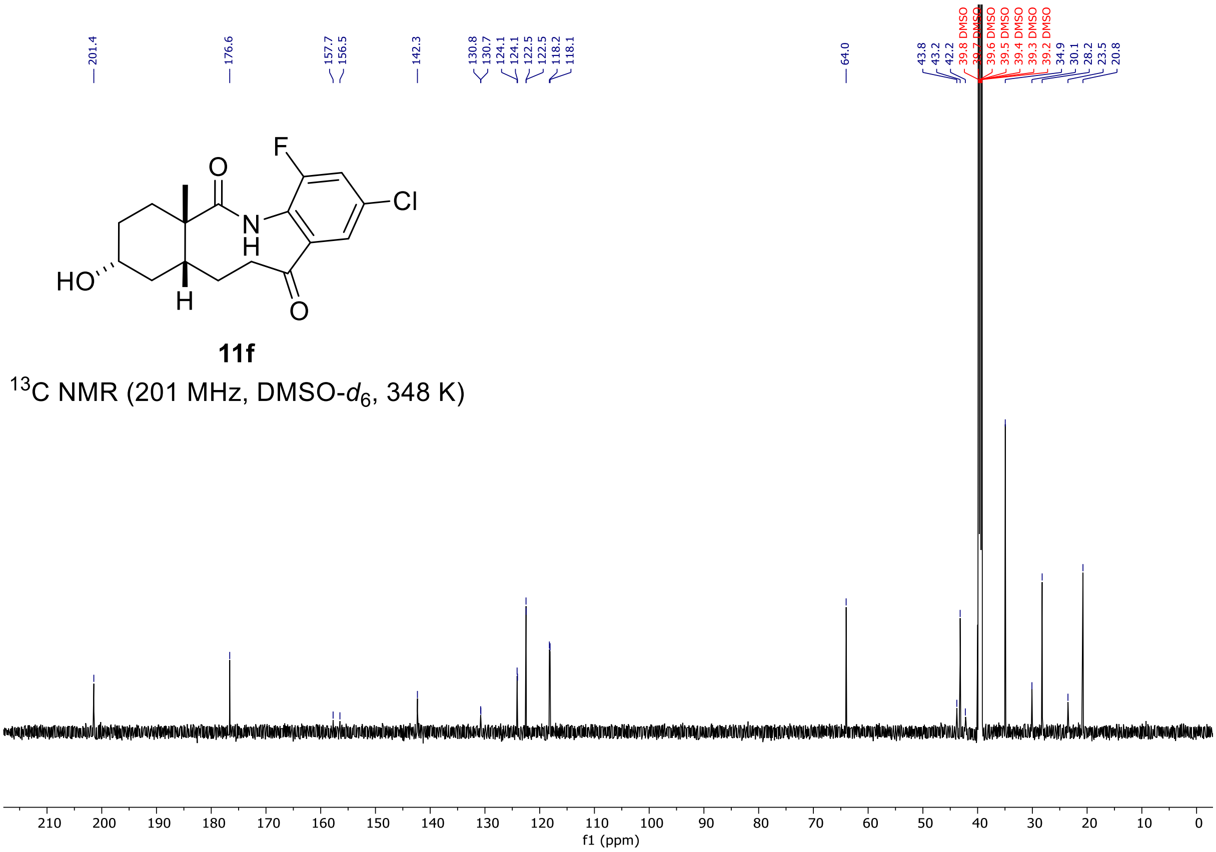

11f

^{13}C NMR (201 MHz, DMSO-d_6, 348 K)

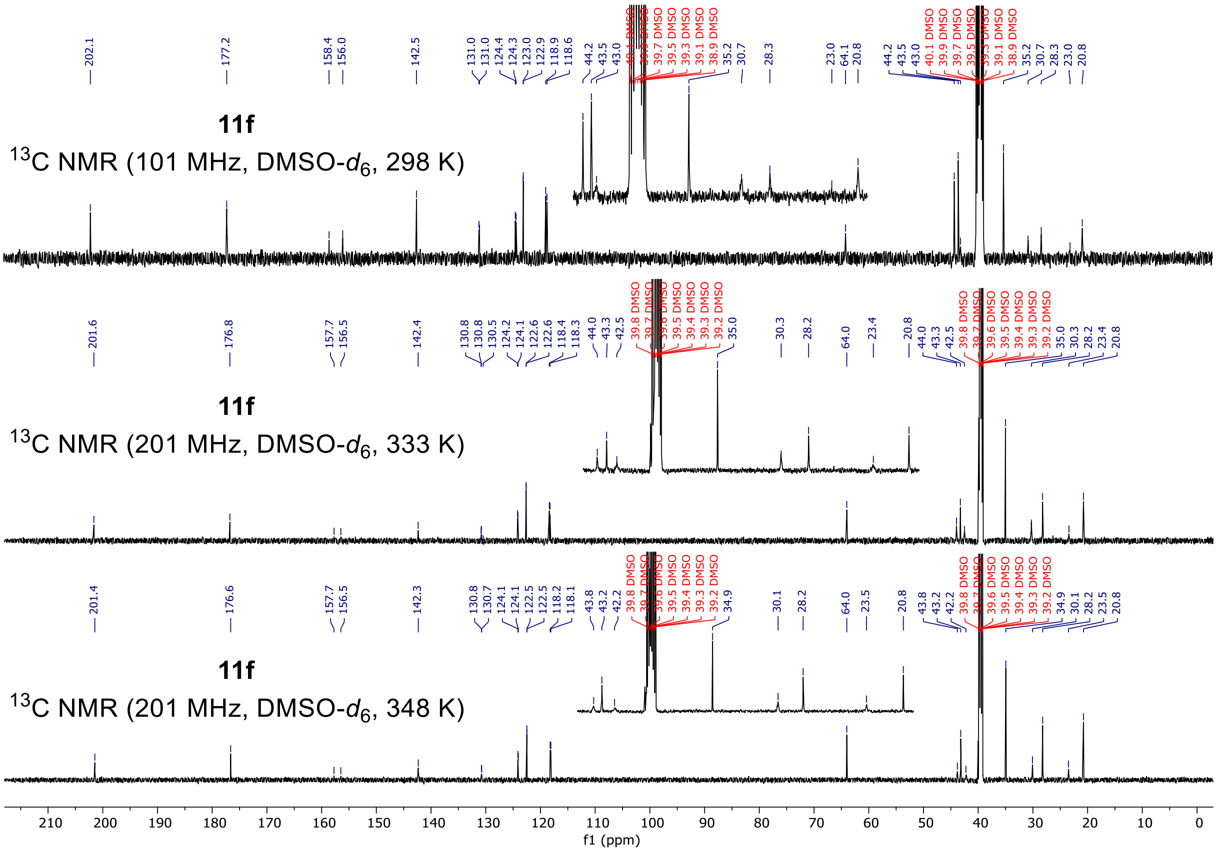

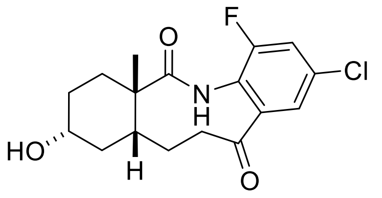

11f

^{19}F NMR (377 MHz, DMSO-d_6)

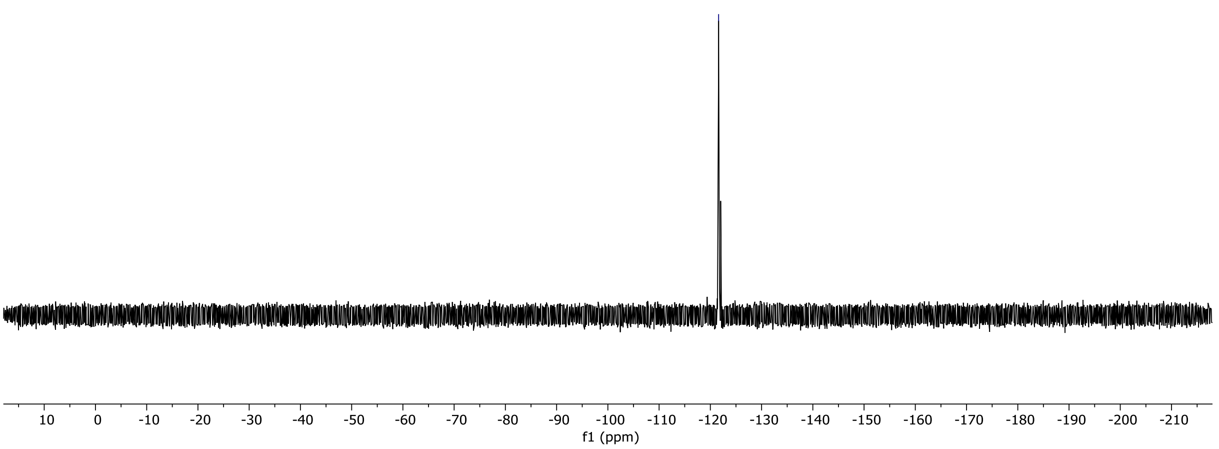

(6a*S,9*R**,10a*R**)-9-Hydroxy-1,3,6a-trimethyl-6a,7,8,9,10,10a,11,12-octahydro-5*H*-dibenzo[*b*,*g*]azonine-6,13-dione (11g)**

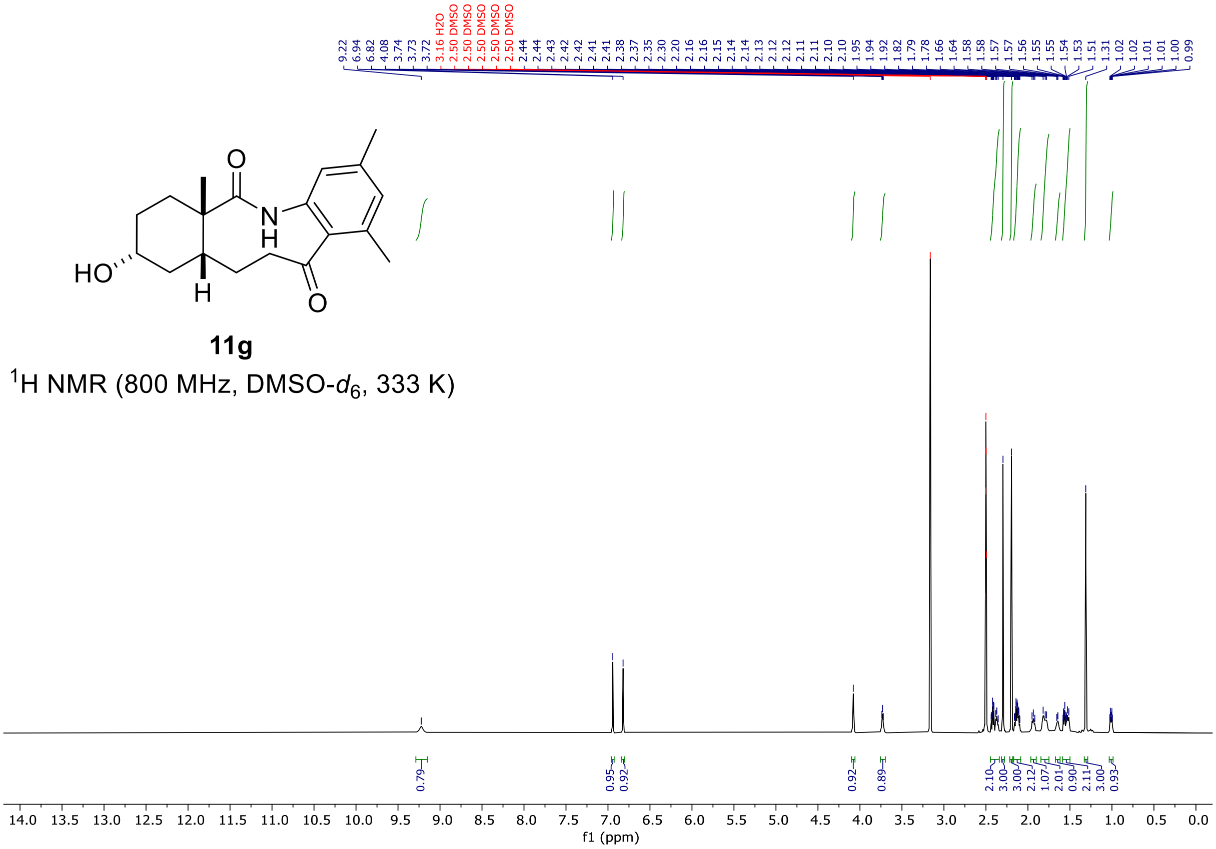

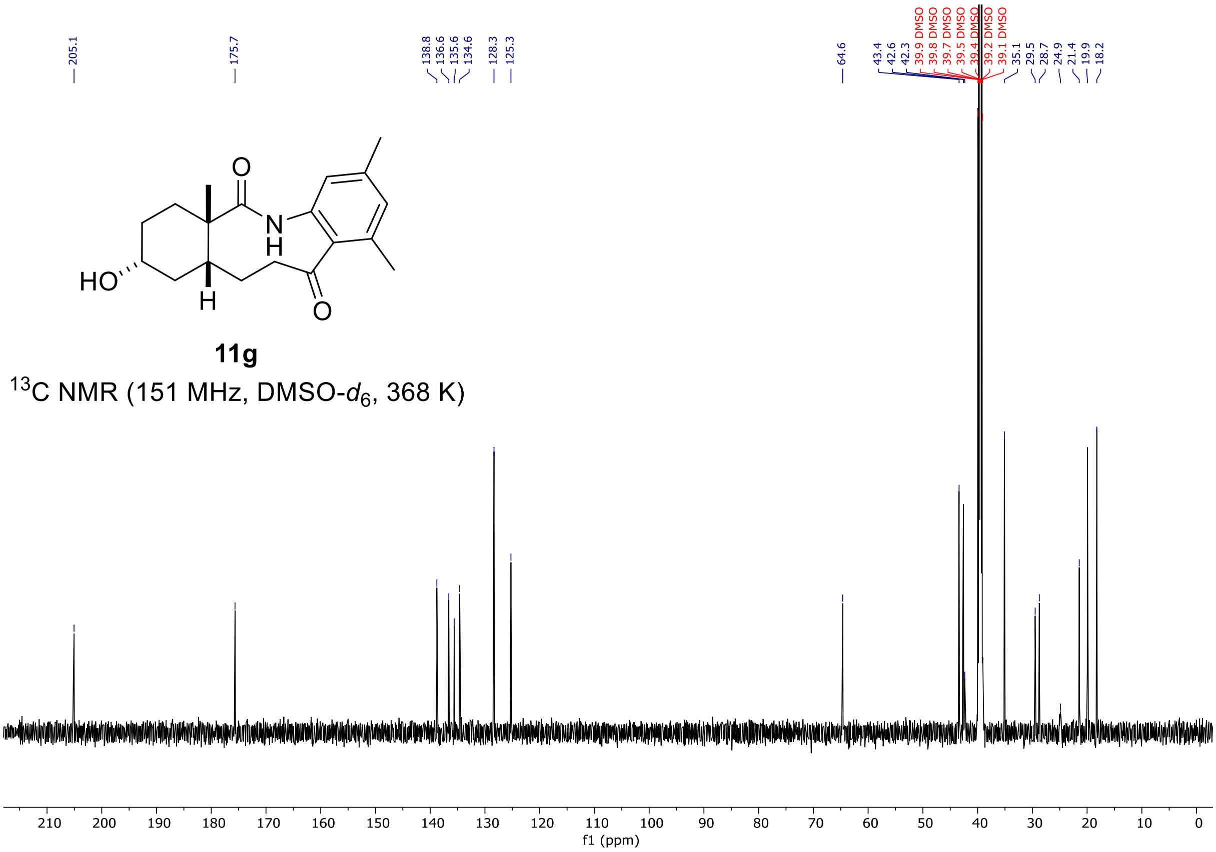

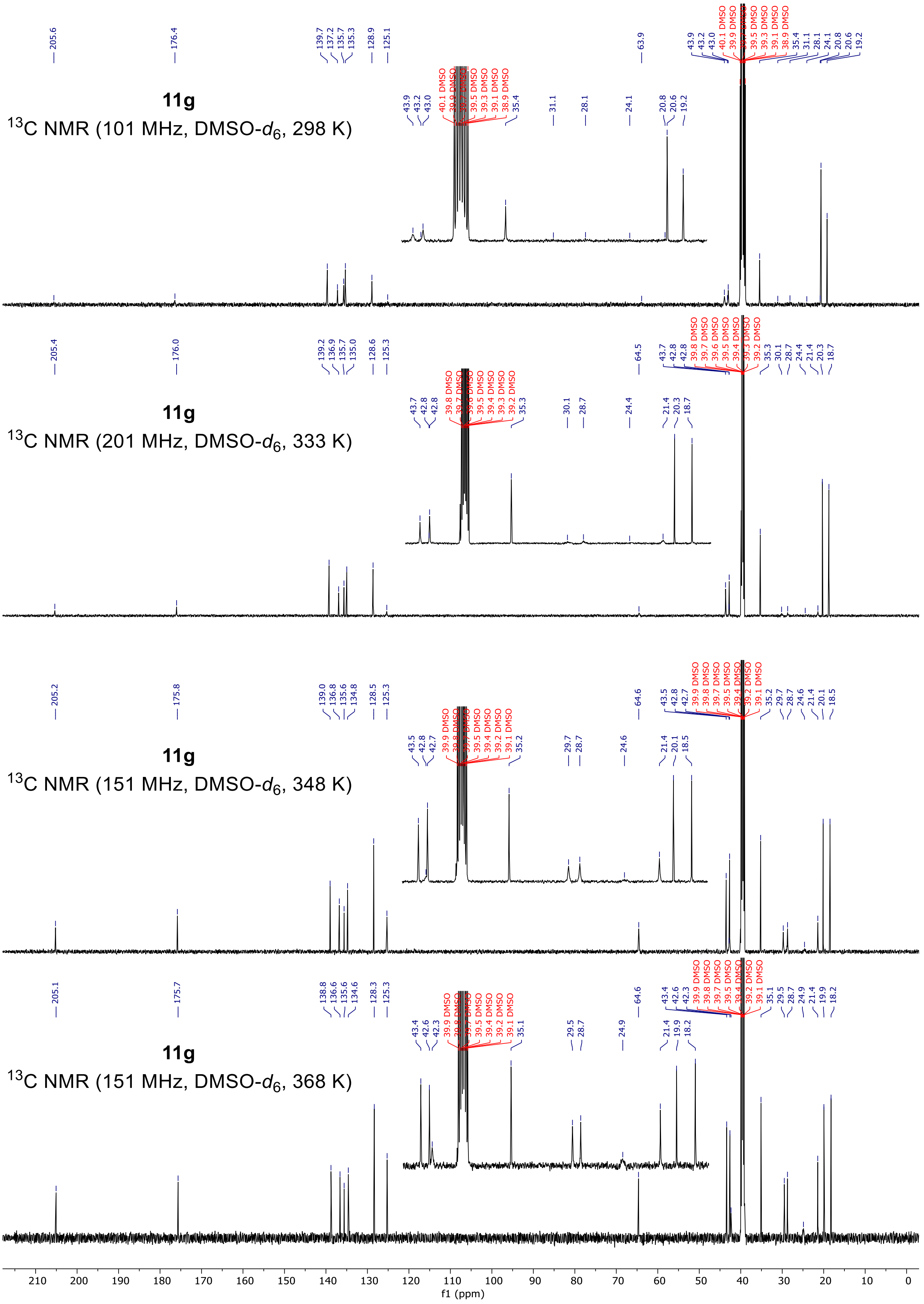
11g
13C NMR (101 MHz, DMSO-d6, 298 K)
11g
13C NMR (201 MHz, DMSO-d6, 333 K)
11g
13C NMR (151 MHz, DMSO-d6, 348 K)
11g
13C NMR (151 MHz, DMSO-d6, 368 K)
f1 (ppm)

(2*R,4a*S**,11a*R**)-2-Hydroxy-4a,8-dimethyl-1,2,3,4,4a,5,11,11a-octahydro-10*H*-indeno[1,2-*b*] quinolin-10-one (12)**

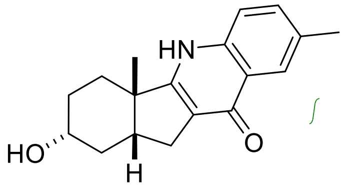

12

^{1}H NMR (400 MHz, DMSO-d_6)

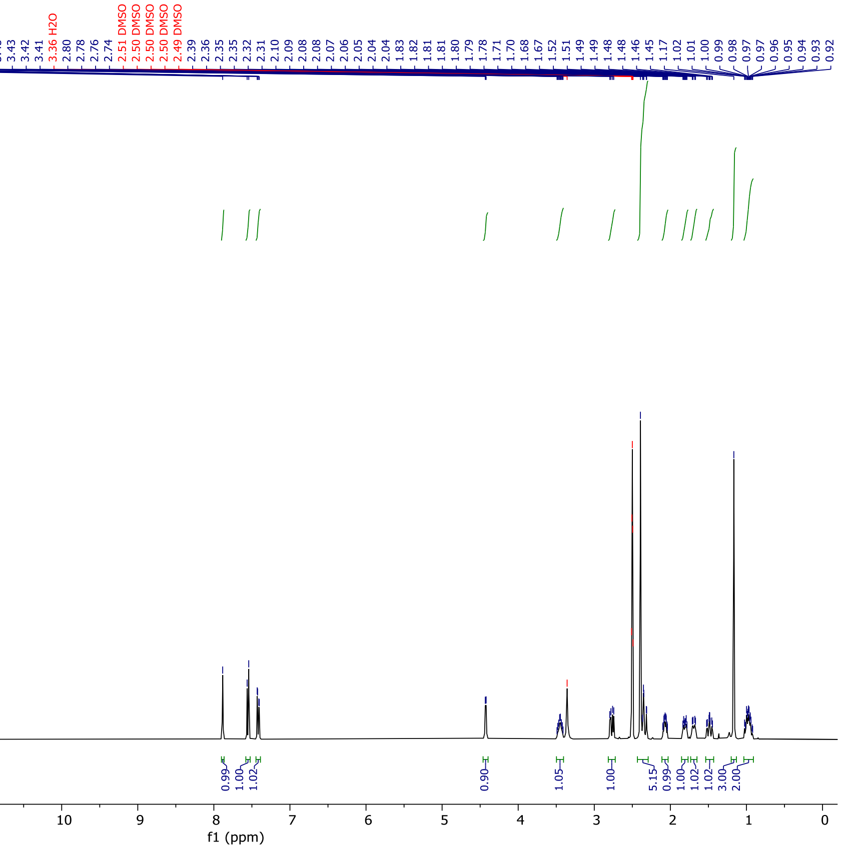

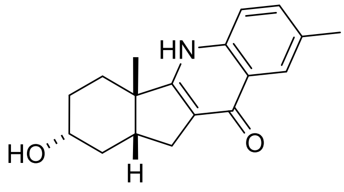

12

^{13}C NMR (101 MHz, DMSO-d_6)

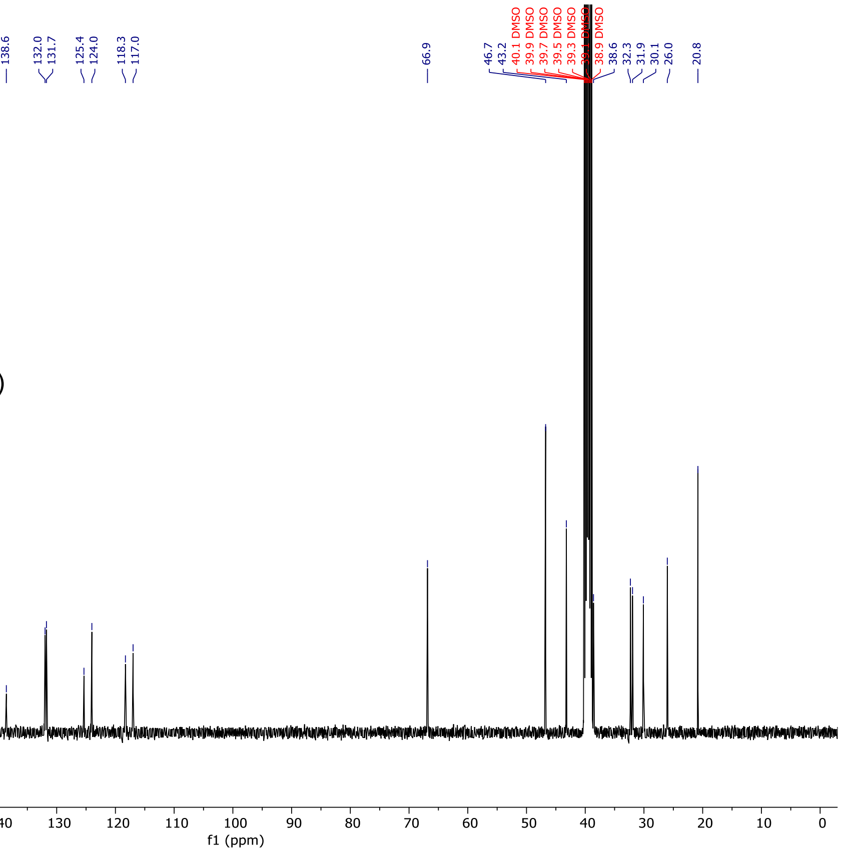

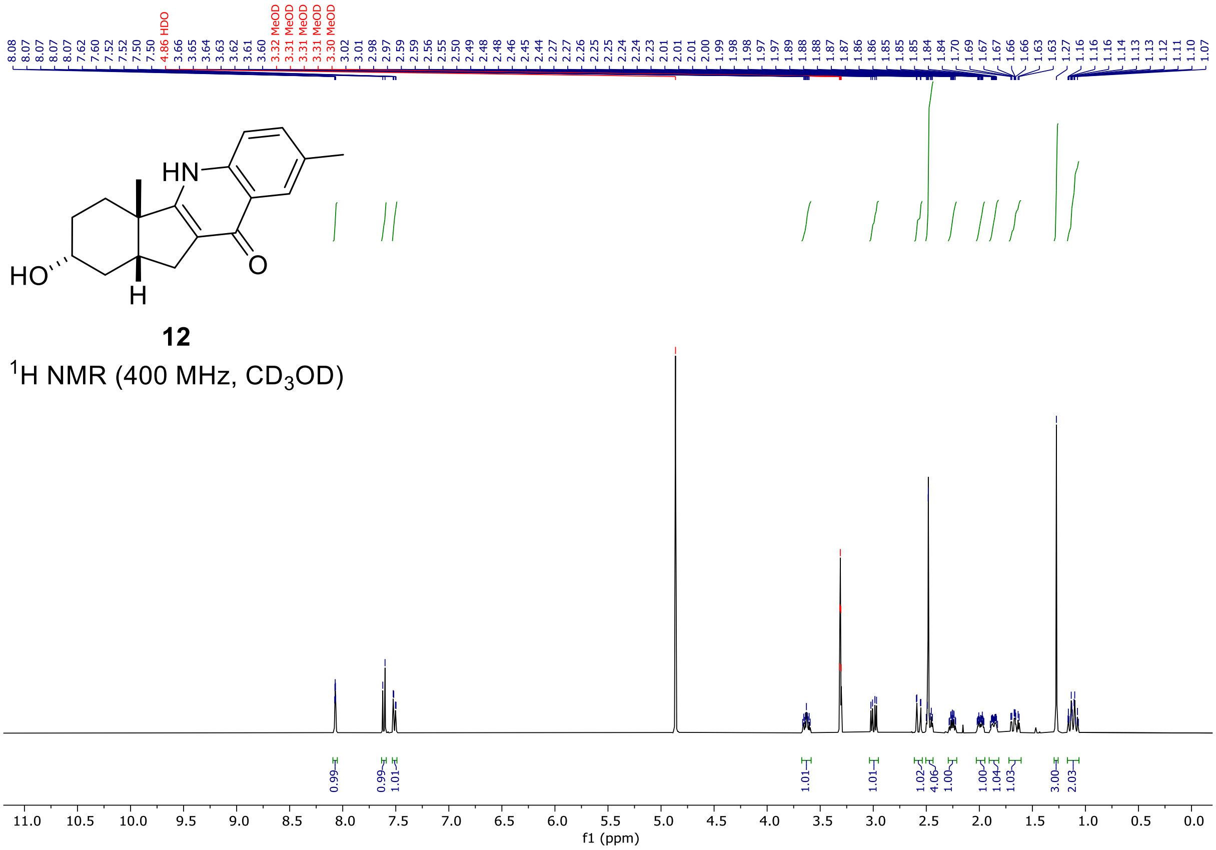

12

^{1}H NMR (400 MHz, CD_3OD)

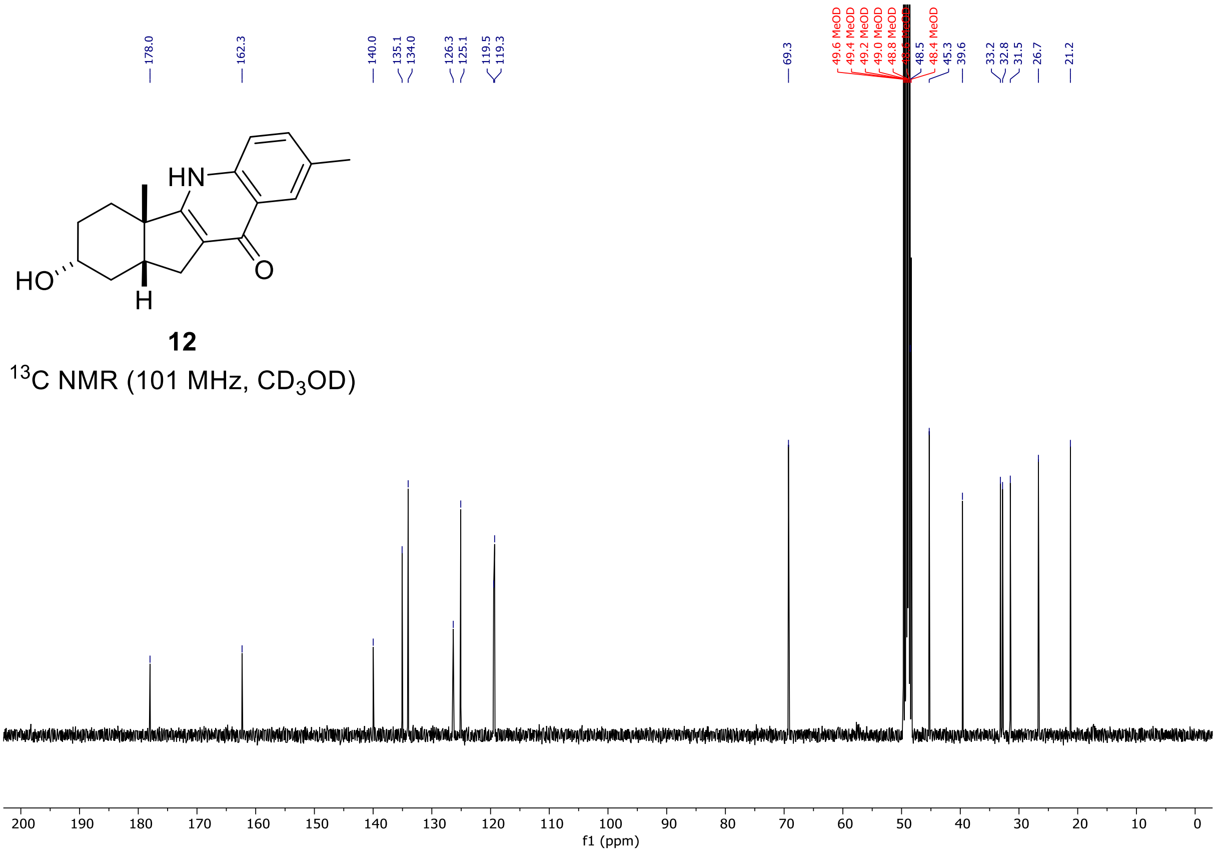

12

^{13}C NMR (101 MHz, CD_3OD)

(1*R,3a*R**,5*R**,7a*S**)-5-Hydroxy-5’,7a-dimethyl-2,3,3a,4,5,6,7,7a-octahydrospiro[indene-1,2’-indolin]-3’-one (13)**

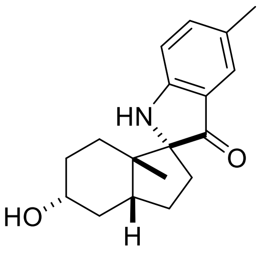

13

^{1}H NMR (400 MHz, DMSO-d_6)

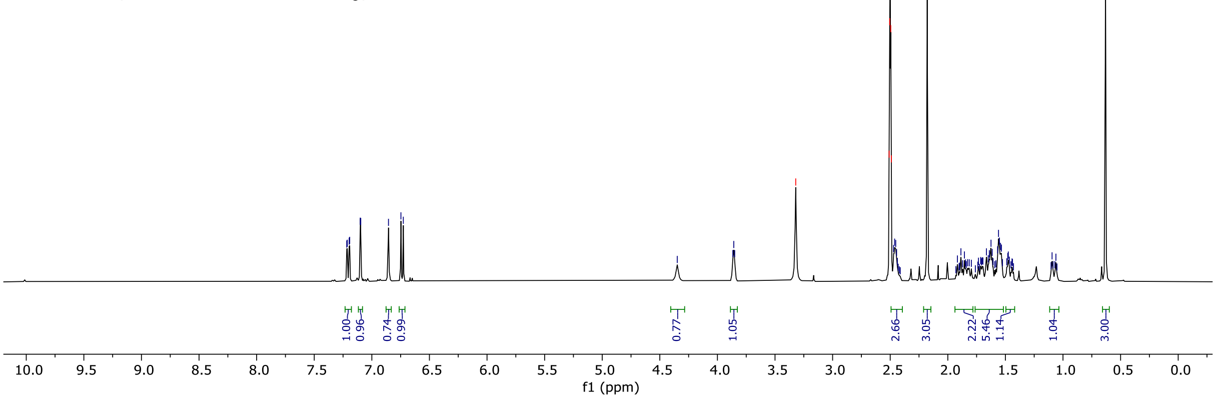

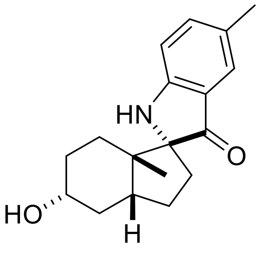

13

^{13}C NMR (101 MHz, DMSO-d_6)

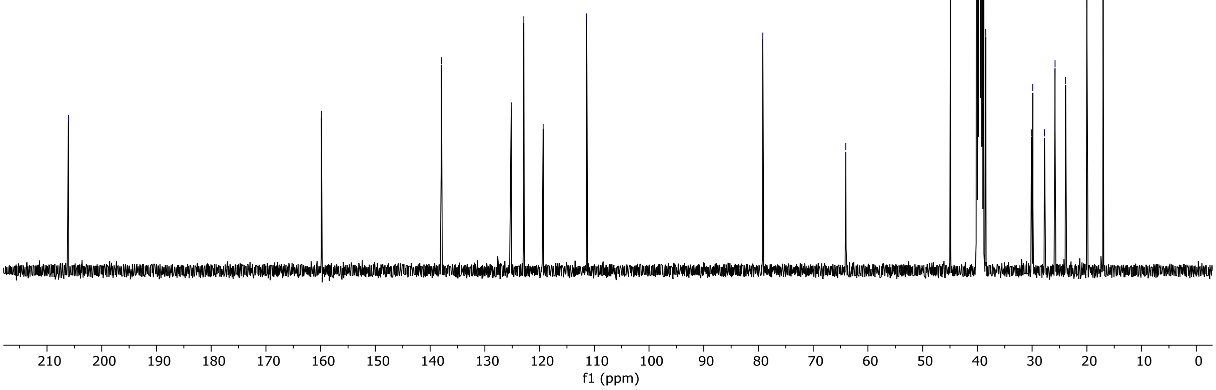

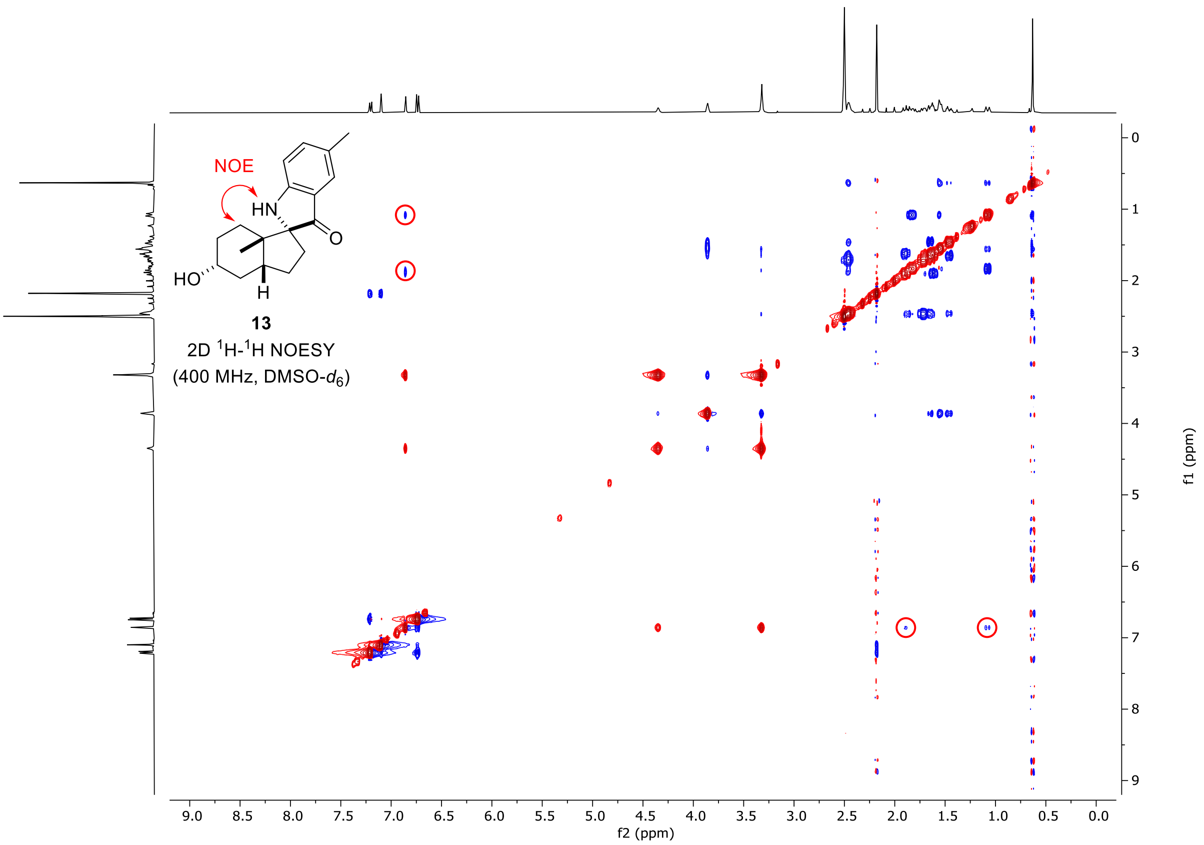
NOE
HN
O
HO
H
13
2D 1H-1H NOESY
(400 MHz, DMSO-d6)
f1 (ppm)
f2 (ppm)

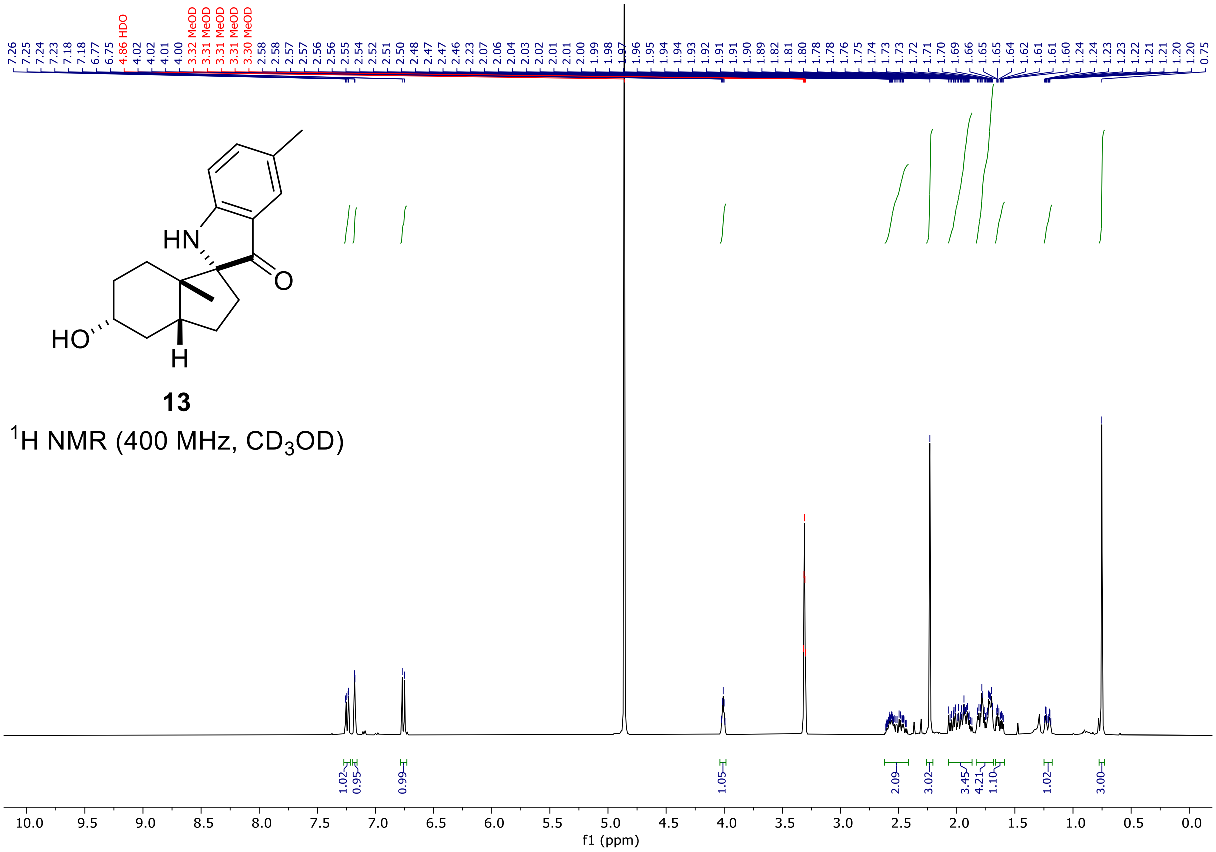
7.26 7.25 7.24 7.23 7.18 7.18 6.77 6.75 4.86 HDO 4.02 4.02 4.01 4.00 3.32 MeOD 3.31 MeOD 3.31 MeOD 3.31 MeOD 3.30 MeOD 2.58 2.58 2.57 2.57 2.56 2.55 2.54 2.52 2.51 2.50 2.48 2.47 2.47 2.46 2.23 2.07 2.06 2.04 2.03 2.02 2.01 2.01 2.00 1.99 1.98 1.97 1.96 1.95 1.94 1.94 1.93 1.92 1.91 1.91 1.90 1.89 1.82 1.81 1.80 1.78 1.76 1.75 1.74 1.73 1.73 1.72 1.71 1.70 1.69 1.66 1.65 1.65 1.64 1.62 1.61 1.61 1.60 1.24 1.24 1.23 1.23 1.22 1.21 1.21 1.20 1.20 0.75
HN
O
HO
H
13
1H NMR (400 MHz, CD3OD)
1.02 0.95 0.99 1.05 2.09 3.02 3.45 4.21 1.10 1.02 3.00
f1 (ppm)

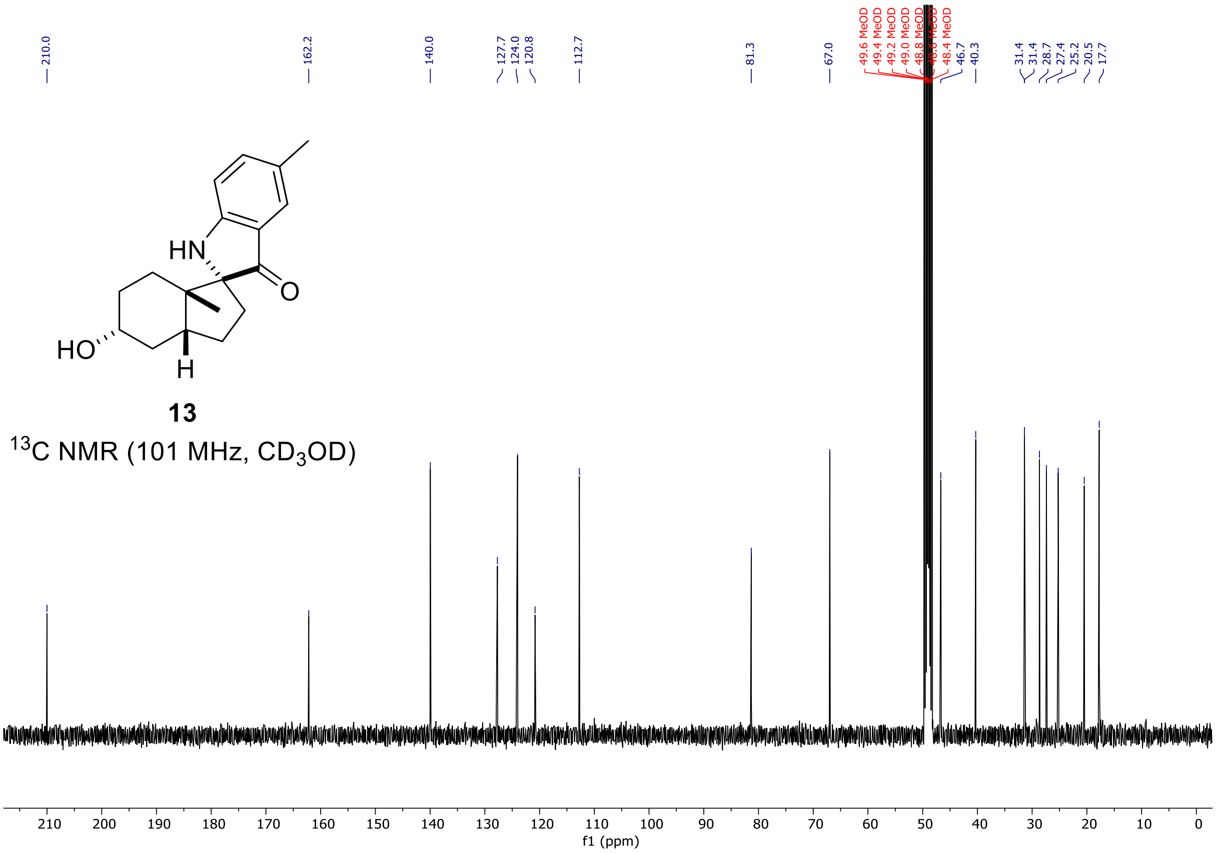

13

^{13}C NMR (101 MHz, CD_3OD)

(3*R,4a*R**,6a*R**,11b*S**)-8,11b-Dimethyl-1,2,3,4,4a,5,6,11b-octahydro-6a*H*-benzo[*a*]carbazole-3,6a-diol (14a)**

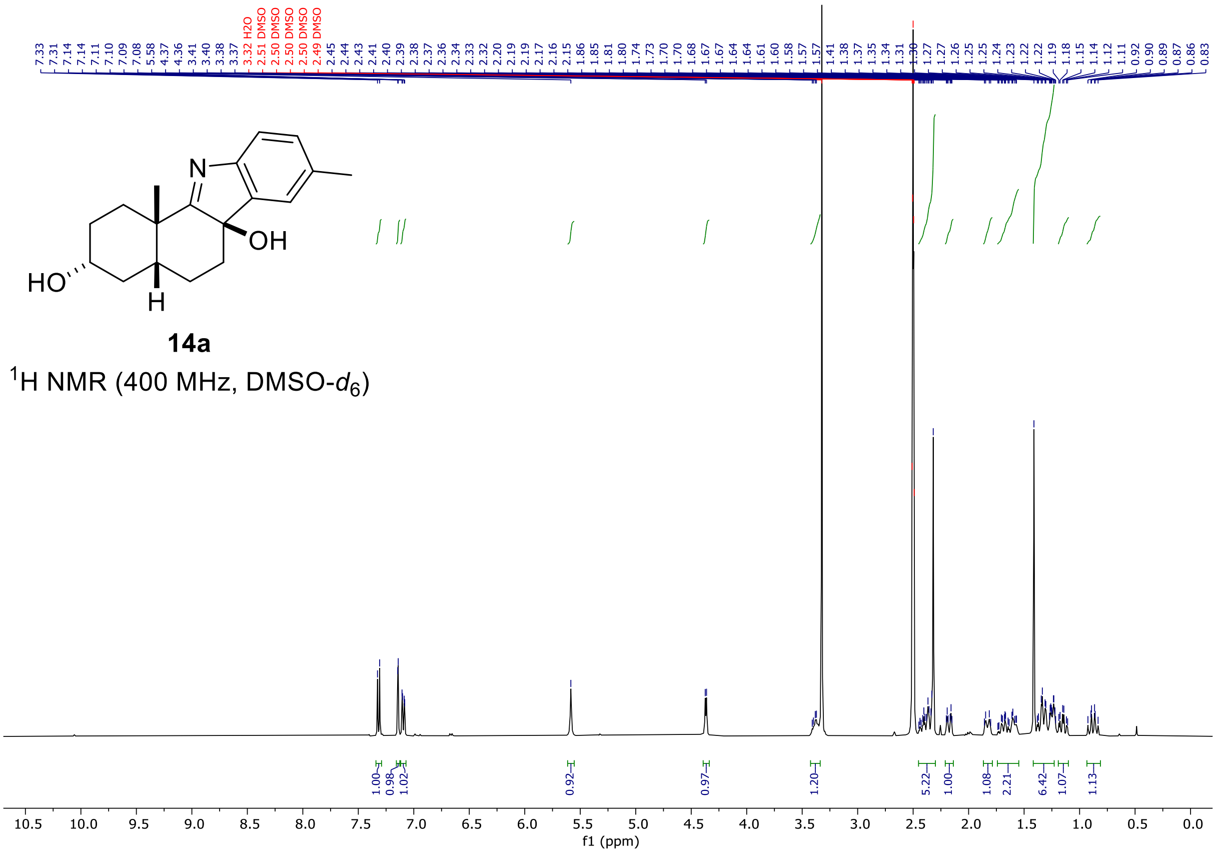

14a

^{1}H NMR (400 MHz, DMSO-d_6)

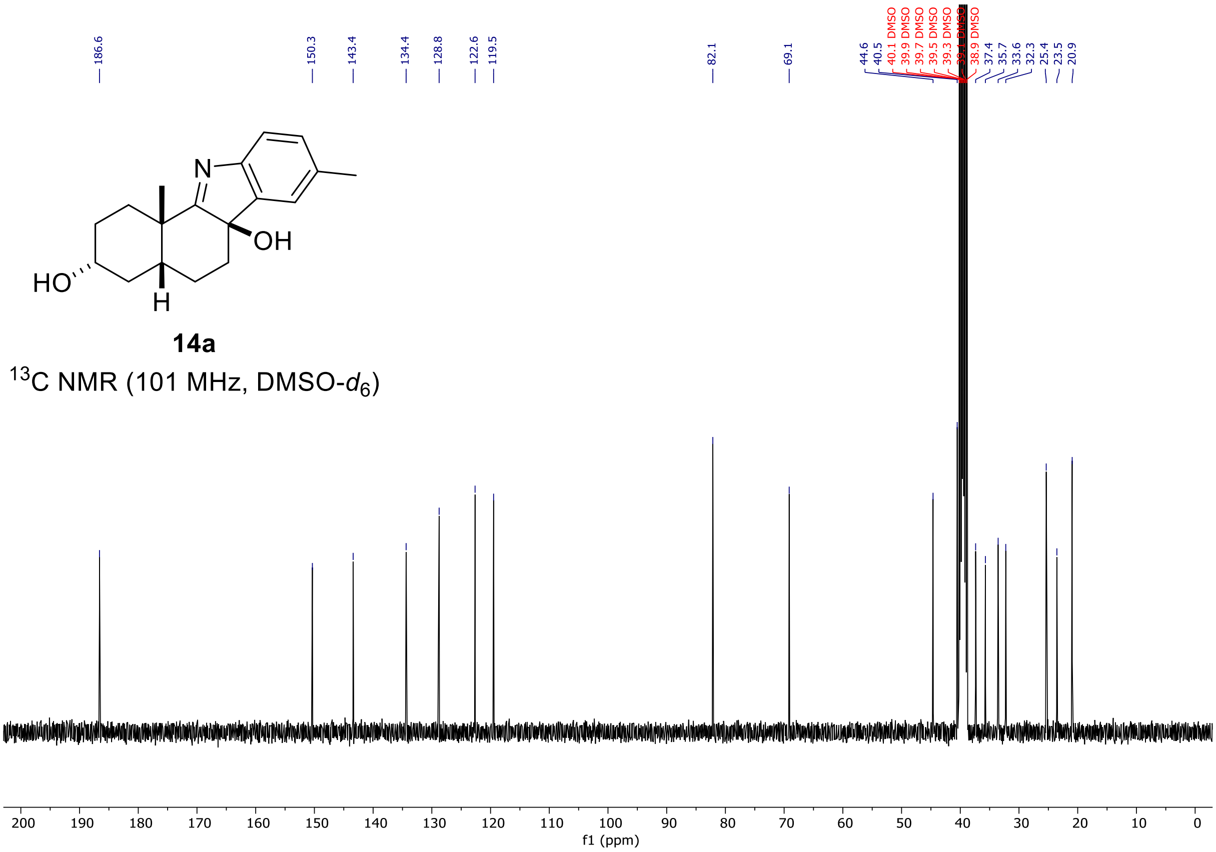

14a

^{13}C NMR (101 MHz, DMSO-d_6)

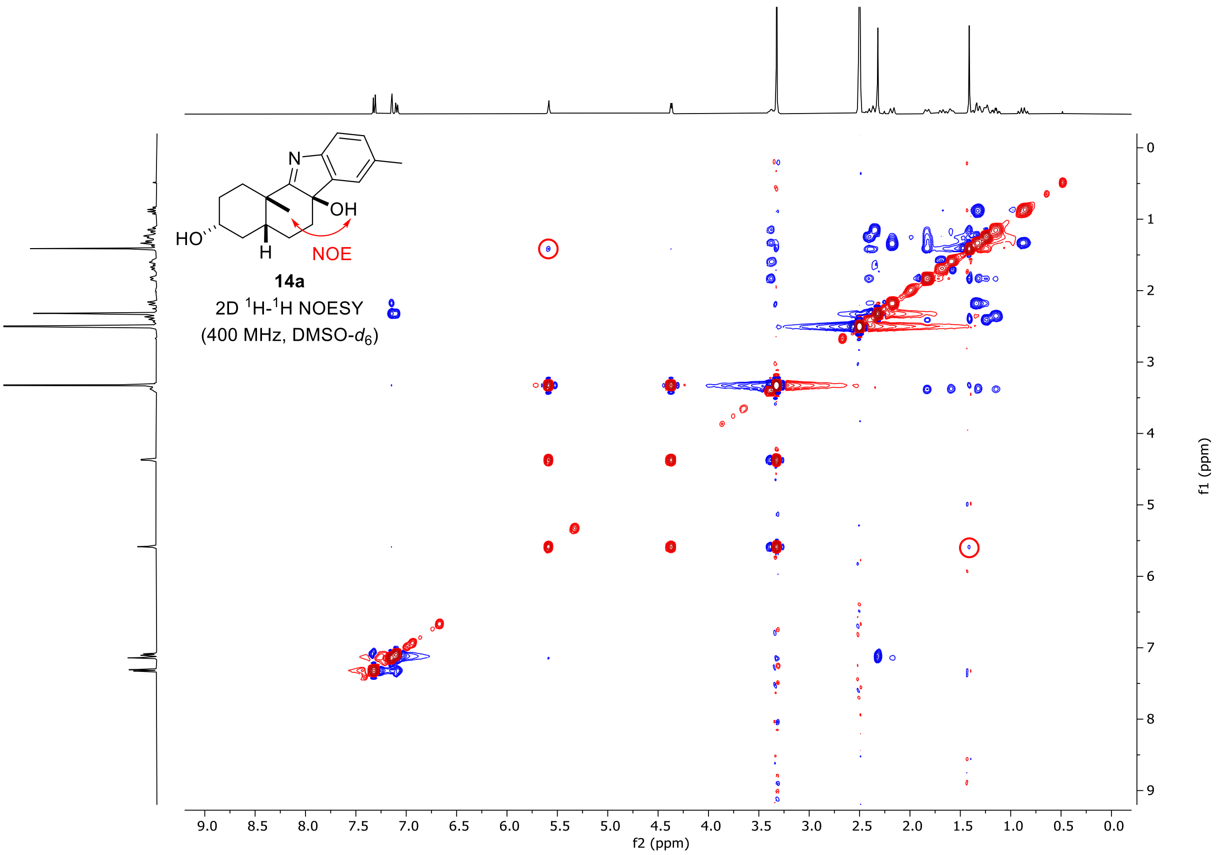

14a
NOE
2D ¹H-¹H NOESY
(400 MHz, DMSO-d6)
f2 (ppm)
f1 (ppm)

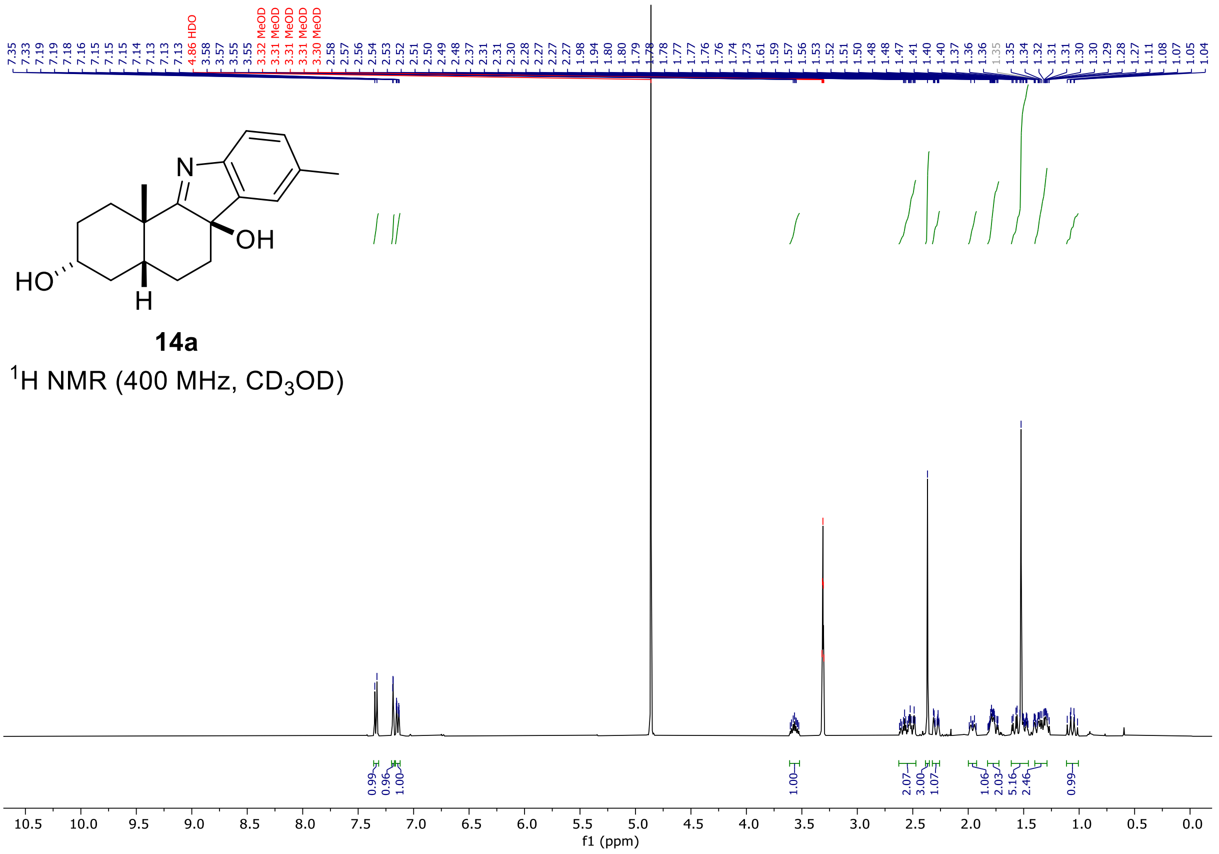

14a
¹H NMR (400 MHz, CD3OD)
f1 (ppm)

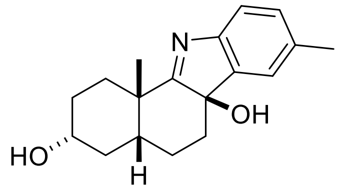

14a

^{13}C NMR (101 MHz, CD_3OD)

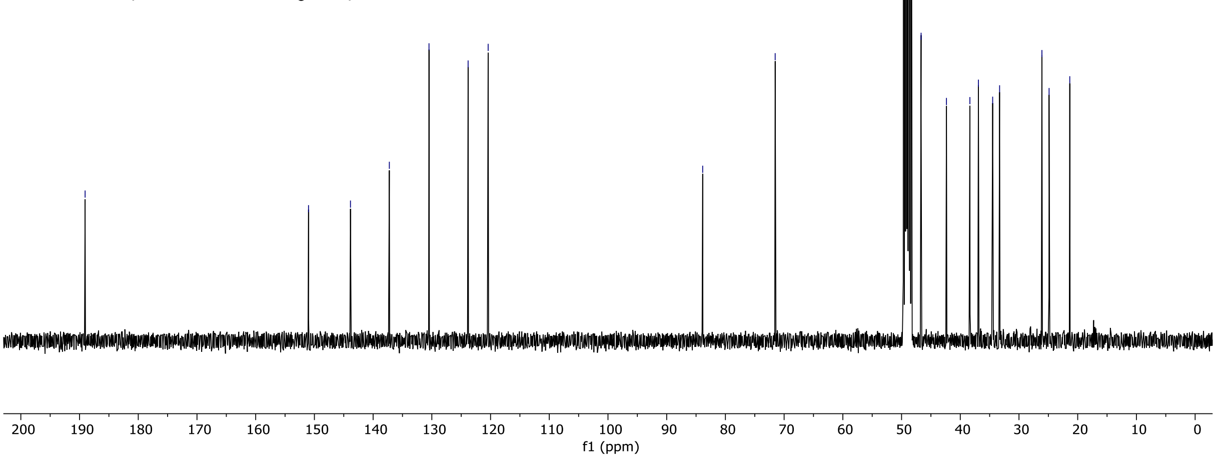

(3*R,4a*R**,6a*R**,11b*S**)-8-Methoxy-11b-methyl-1,2,3,4,4a,5,6,11b-octahydro-6a*H*-benzo[*a*]carbazole-3,6a-diol (14b)**

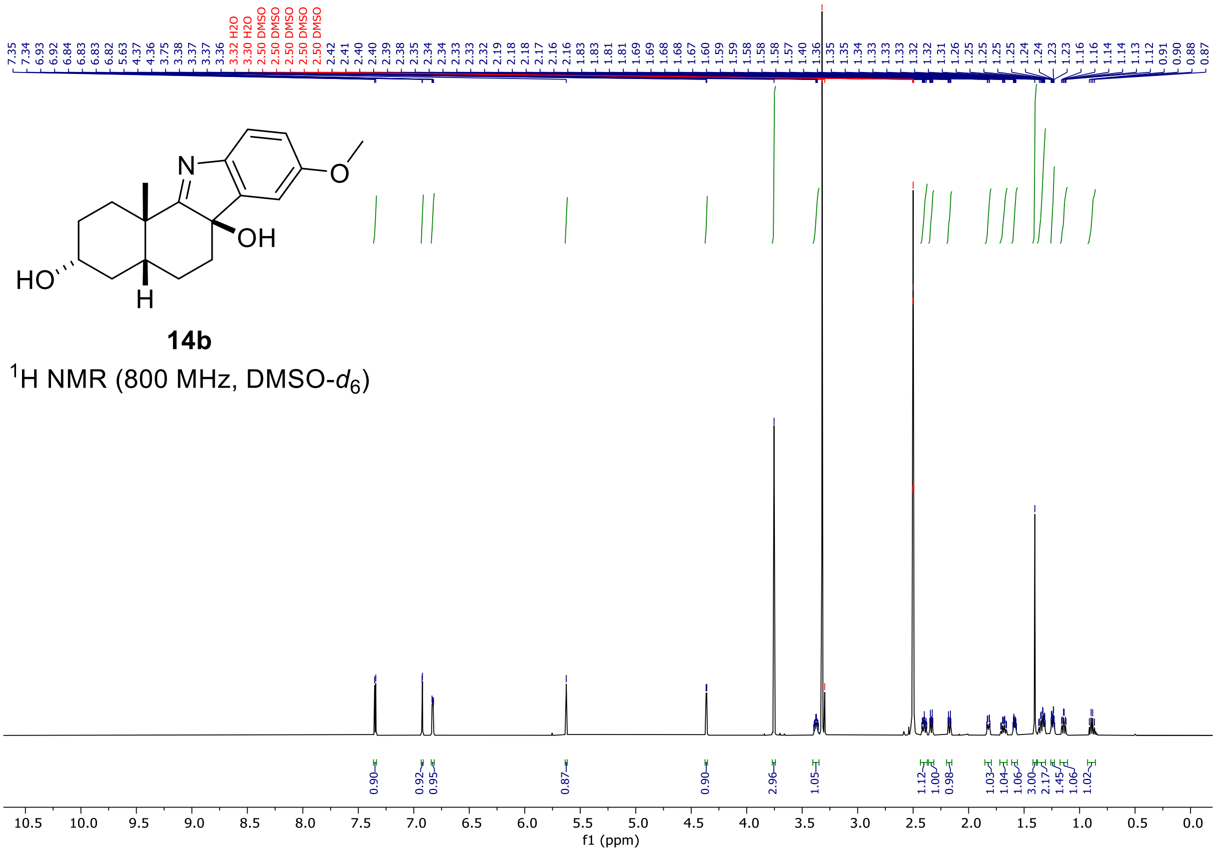

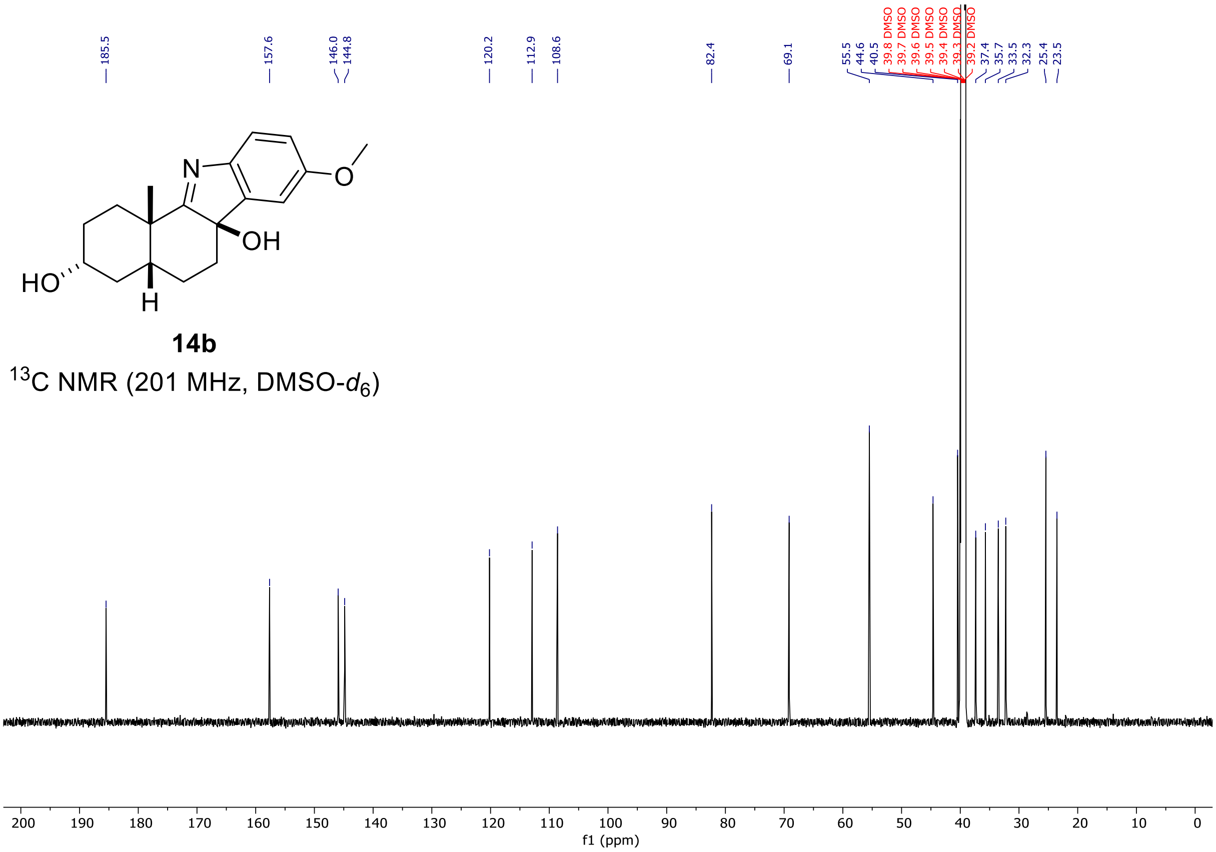

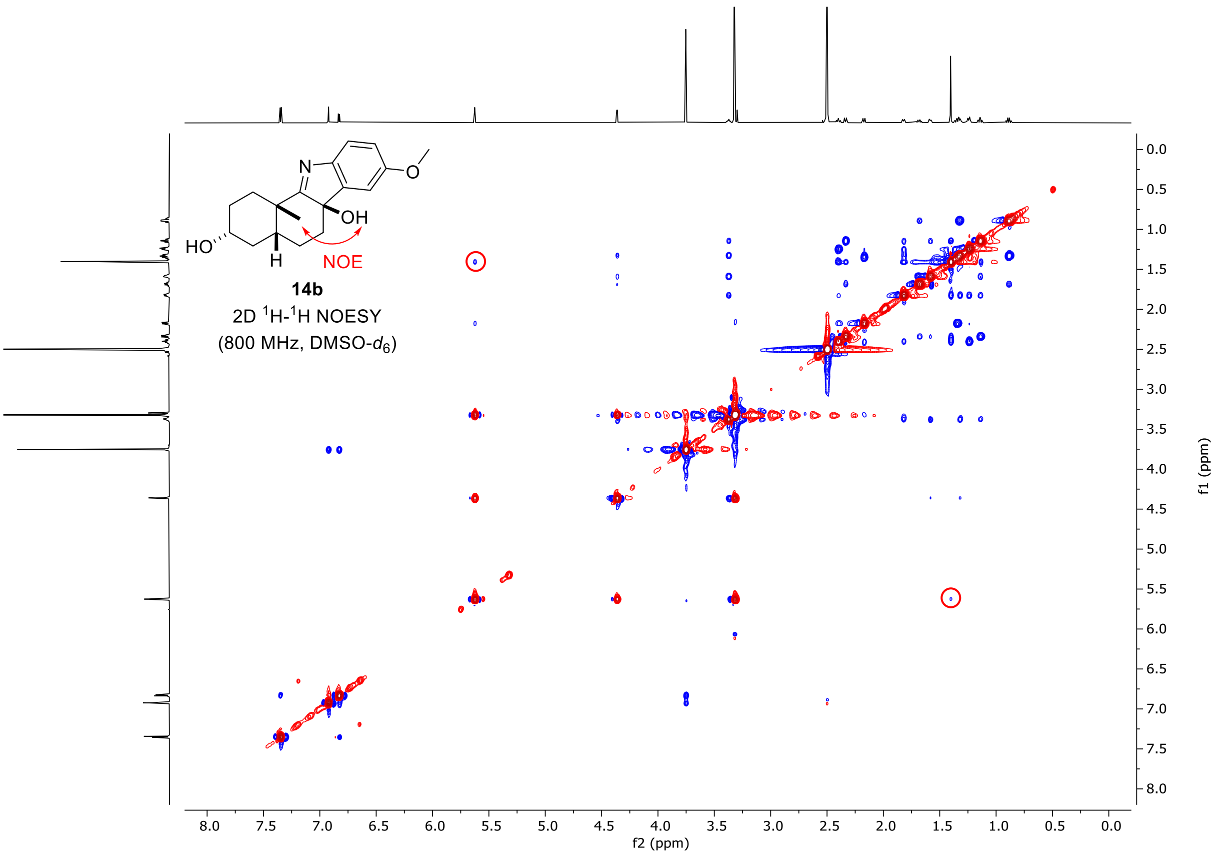
HO
OH
N
O
H
NOE
14b
2D 1H-1H NOESY
(800 MHz, DMSO-d6)
f1 (ppm)
f2 (ppm)

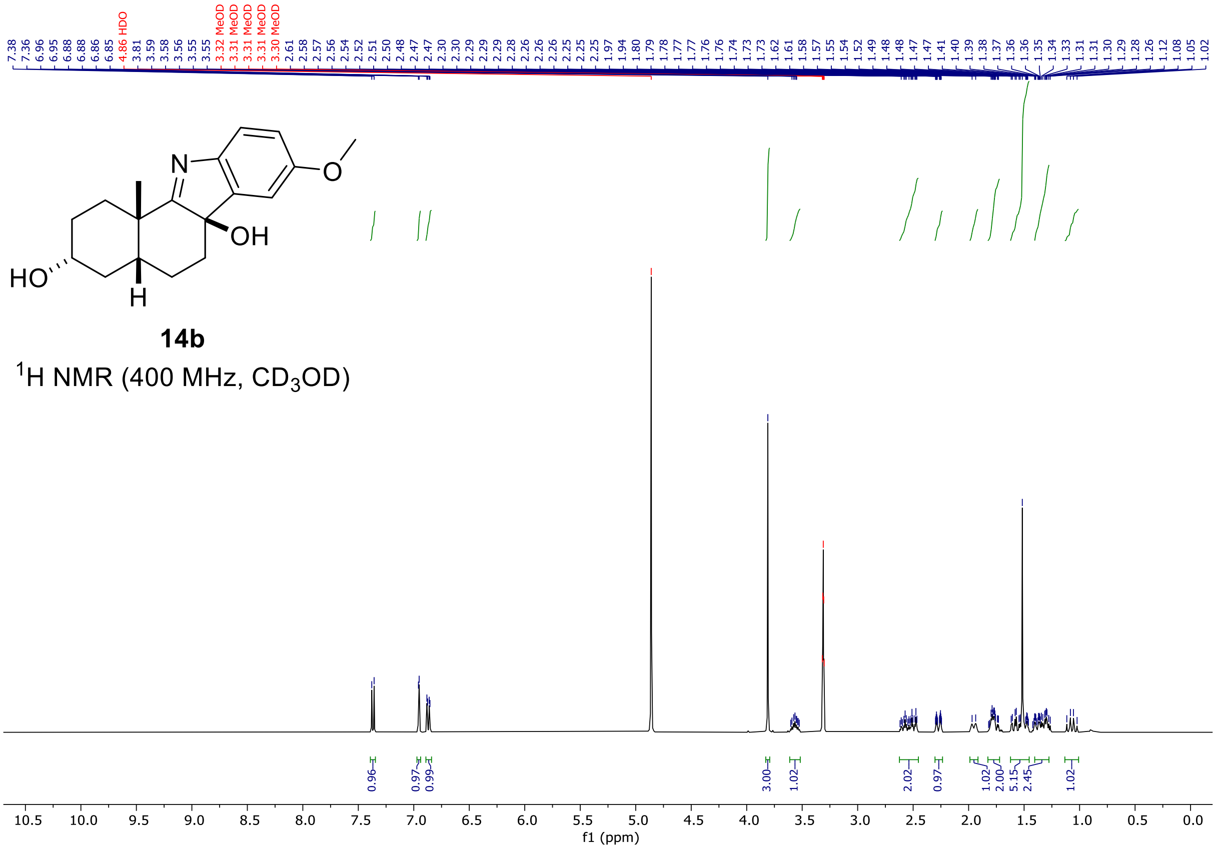
N
O
OH
HO
H
14b
1H NMR (400 MHz, CD3OD)
4.86 HDO
3.32 MeOD
3.31 MeOD
3.31 MeOD
3.31 MeOD
3.30 MeOD
f1 (ppm)

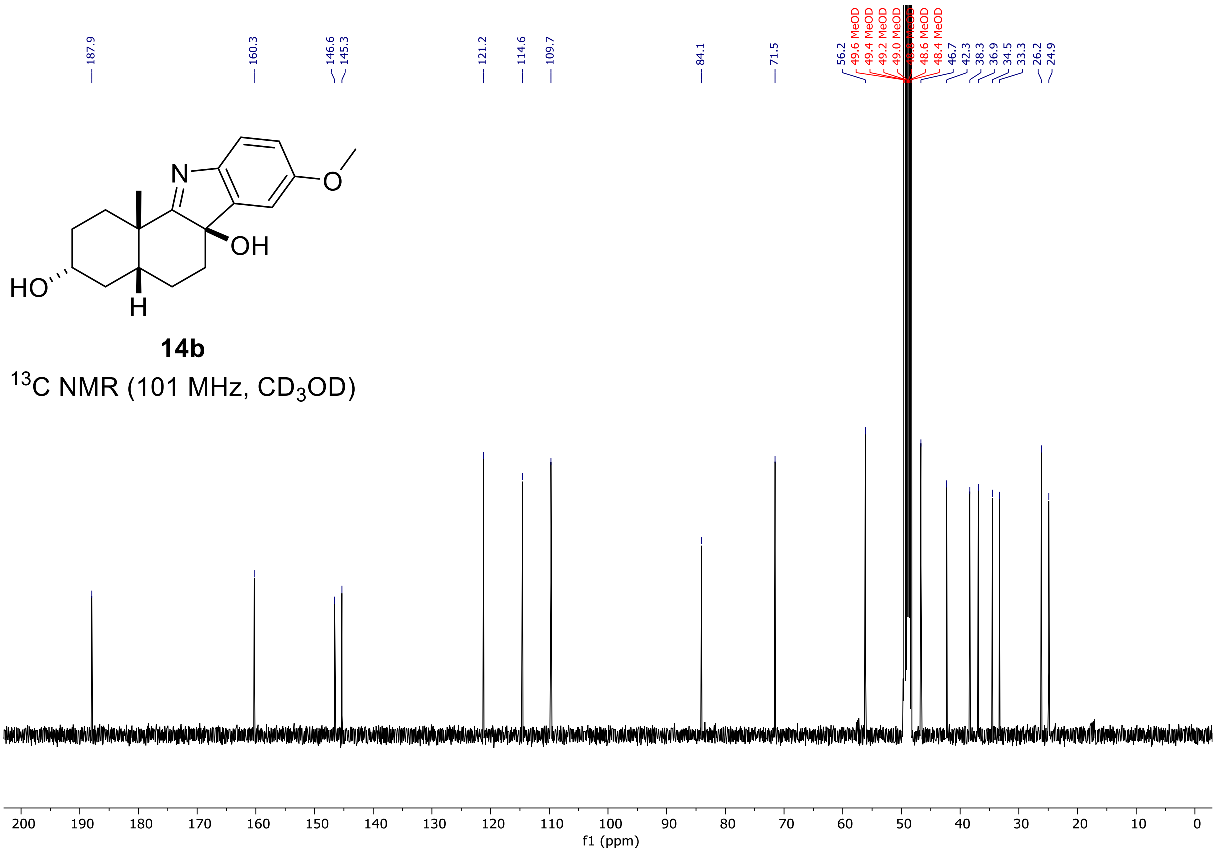

14b

^{13}C NMR (101 MHz, CD_3OD)

(3*R,4a*R**,6a*R**,11b*S**)-7,9,11b-Trimethyl-1,2,3,4,4a,5,6,11b-octahydro-6a*H*-benzo[*a*]carbazole-3,6a-diol (14c)**

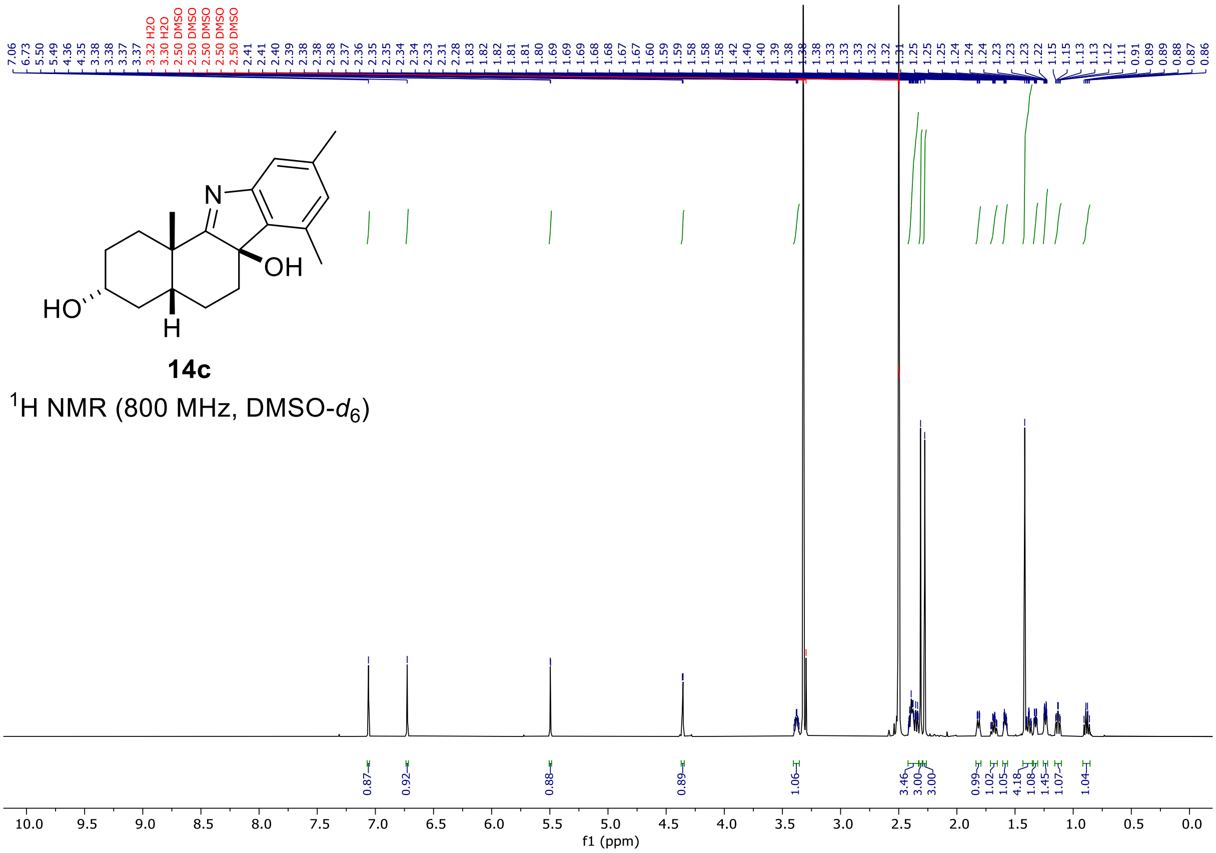

14c

^{1}H NMR (800 MHz, DMSO-d_6)

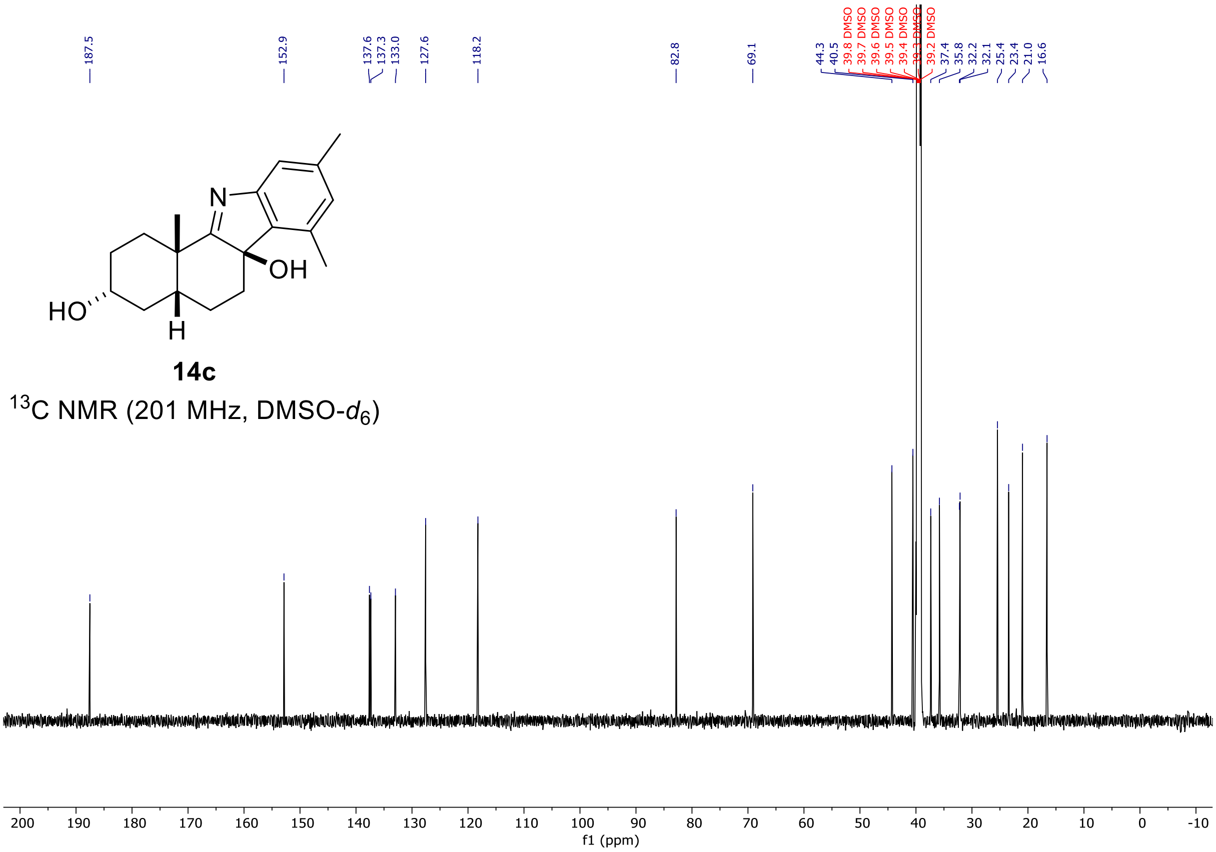

14c

^{13}C NMR (201 MHz, DMSO-d_6)

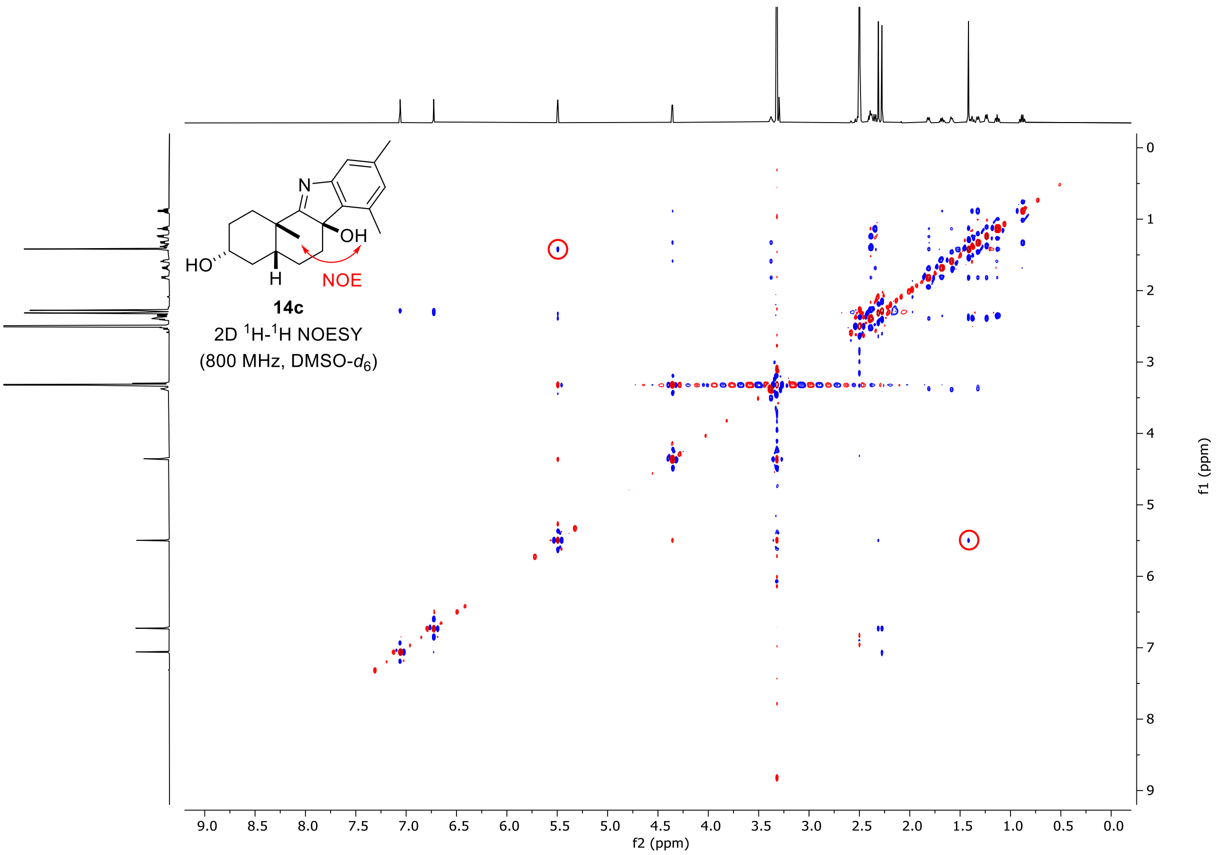
HO
H
OH
N
NOE
14c
2D ^{1}H-^{1}H NOESY
(800 MHz, DMSO-d_6)
f2 (ppm)
f1 (ppm)

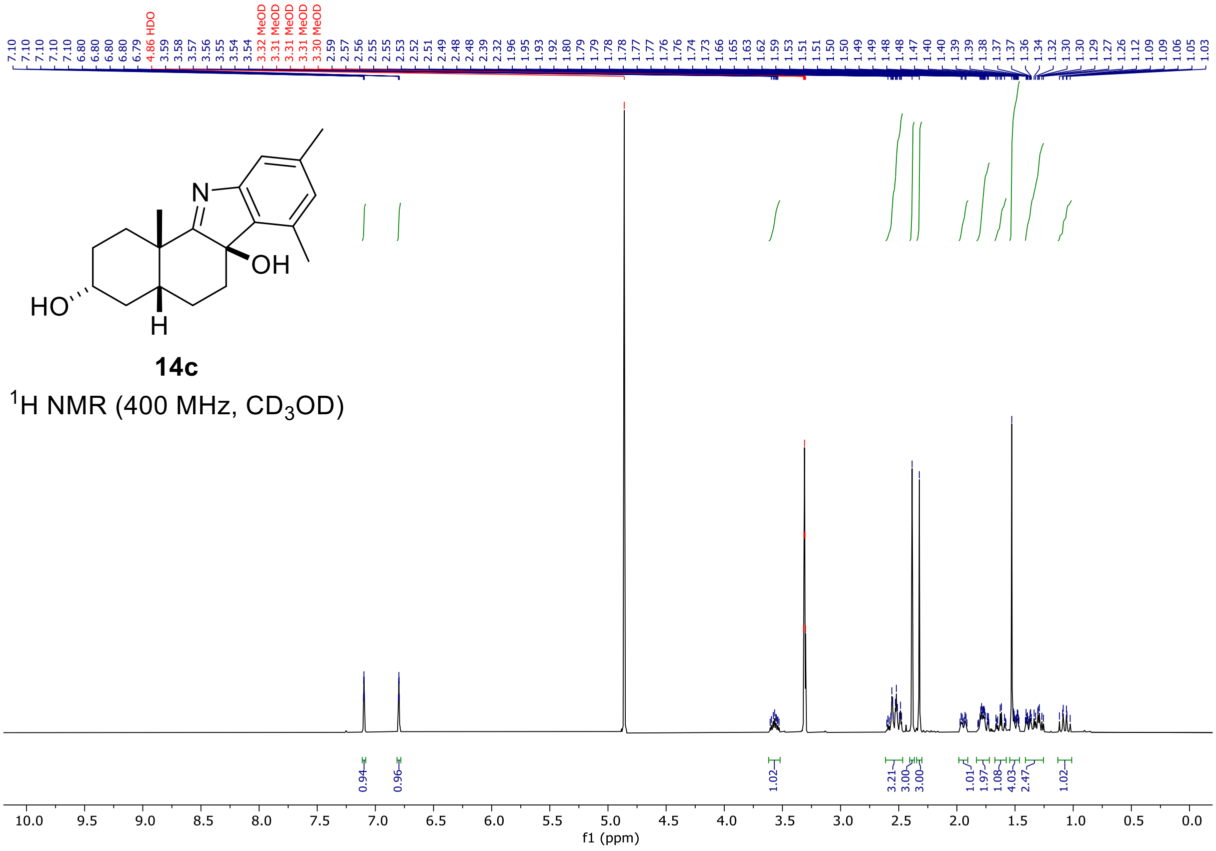
HO
H
OH
N
14c
^{1}H NMR (400 MHz, CD_3OD)
f1 (ppm)

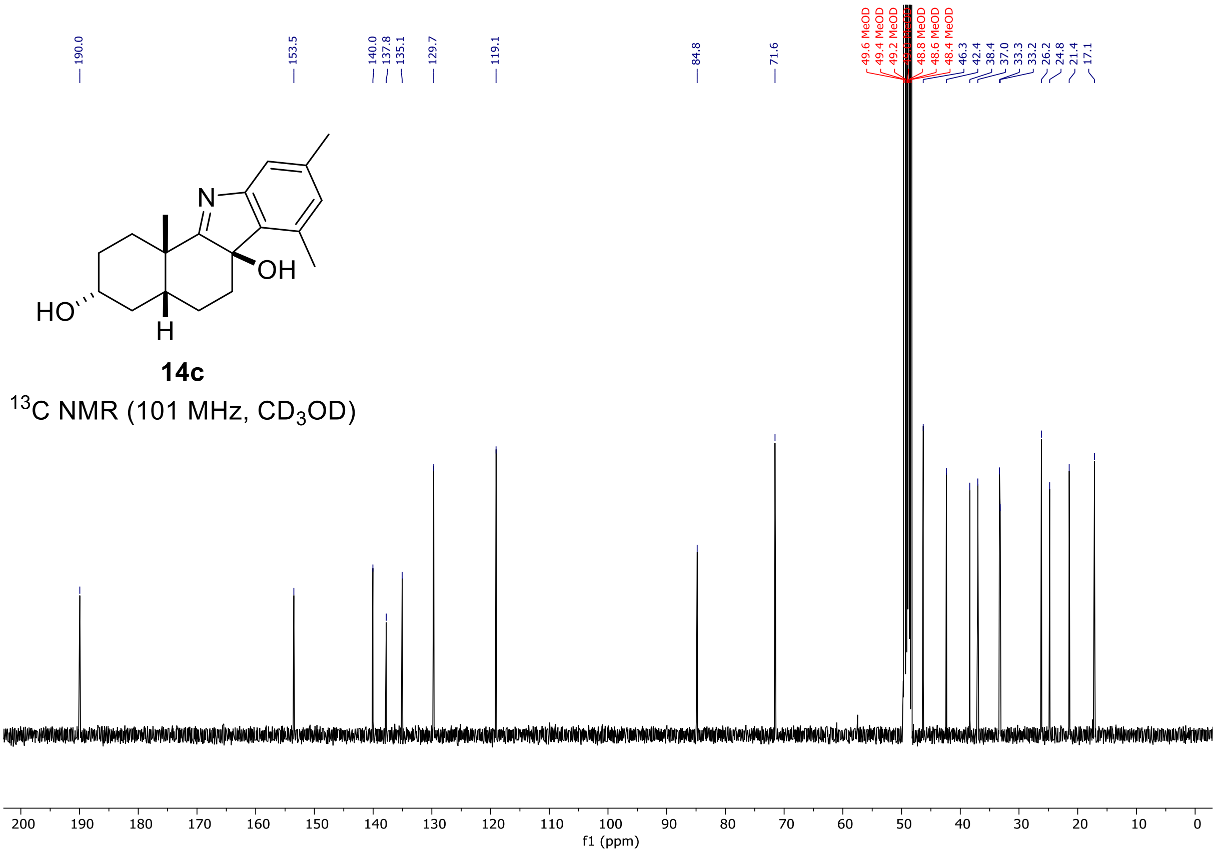
190.0
153.5
140.0
137.8
135.1
129.7
119.1
84.8
71.6
49.6 MeOD
49.4 MeOD
49.2 MeOD
48.8 MeOD
48.6 MeOD
48.4 MeOD
46.3
42.4
38.4
37.0
33.3
33.2
26.2
24.8
21.4
17.1
N
OH
HO
H
14c
^{13}C NMR (101 MHz, CD_3OD)
200 190 180 170 160 150 140 130 120 110 100 90 80 70 60 50 40 30 20 10 0
f1 (ppm)

(3*R,3a*S**,6*R**,12b*R**)-3a-Methyl-2,3,3a,4,5,6-hexahydro-1*H*-3,6-methanocyclopenta[3,4]oxepino[2,3-*b*]indole (15a)**

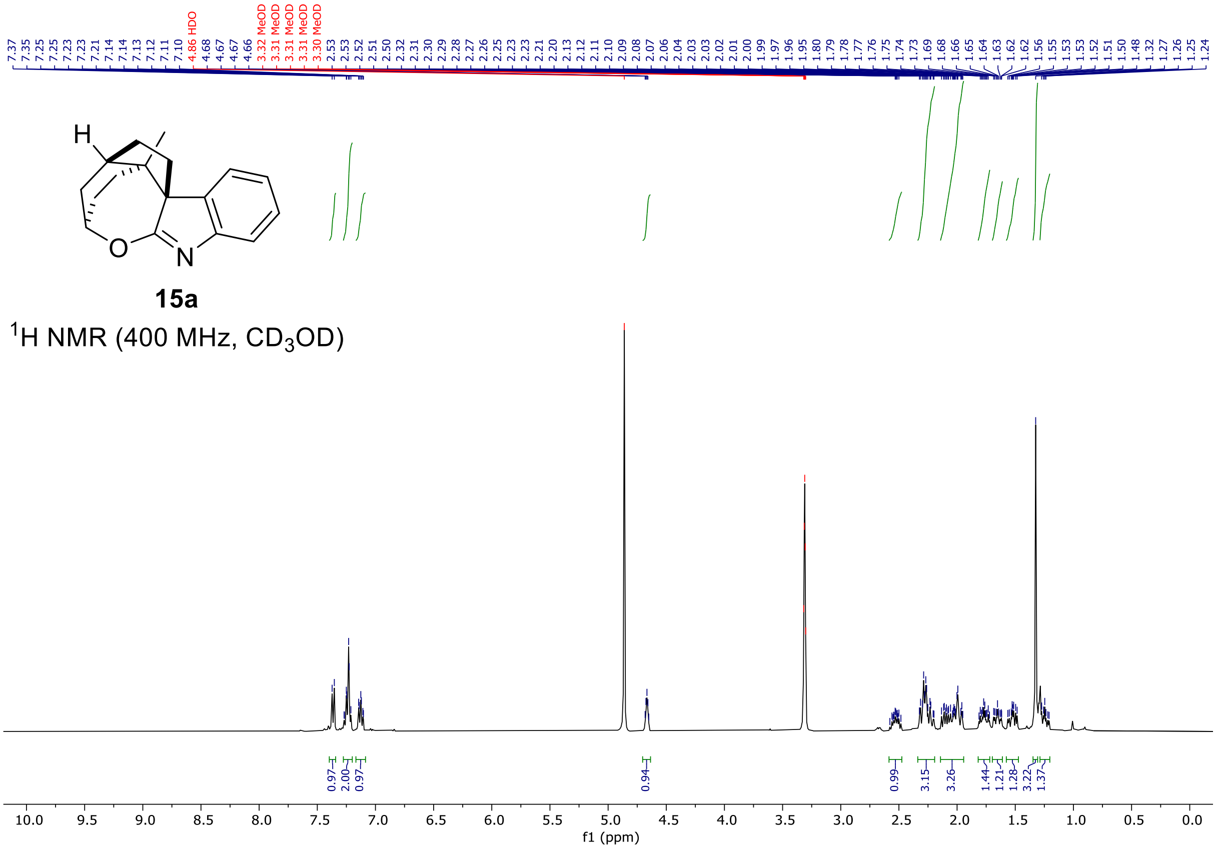

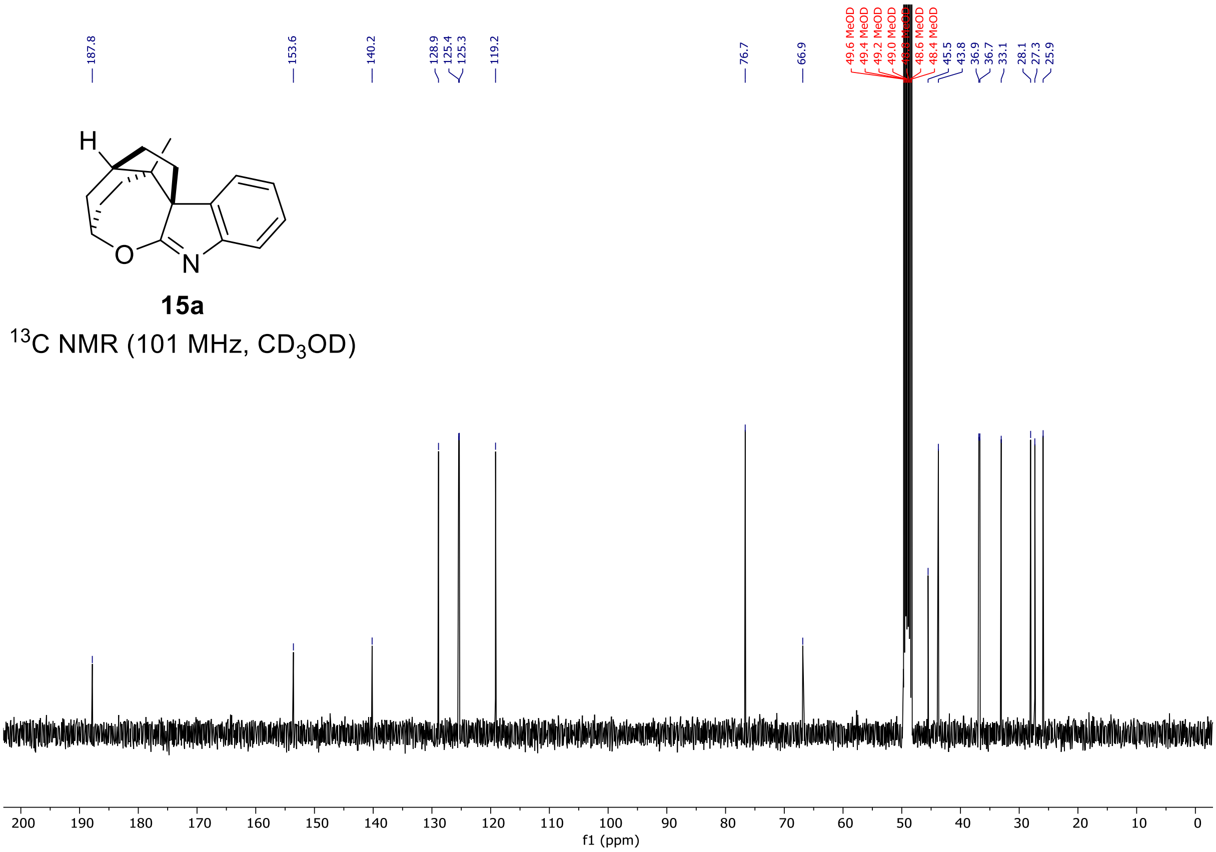

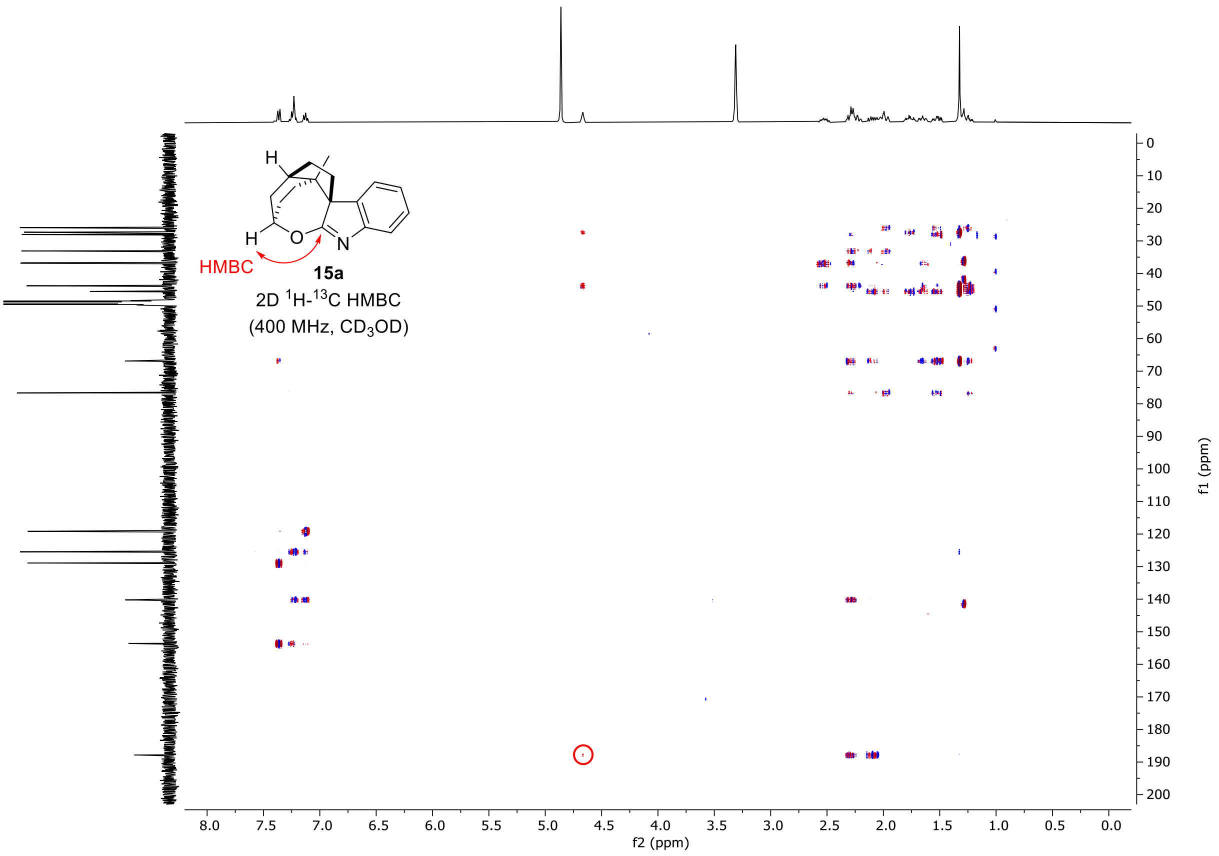
H
H
O
N
HMBC
15a
2D ^{1}H-^{13}C HMBC
(400 MHz, CD_3OD)
f1 (ppm)
f2 (ppm)

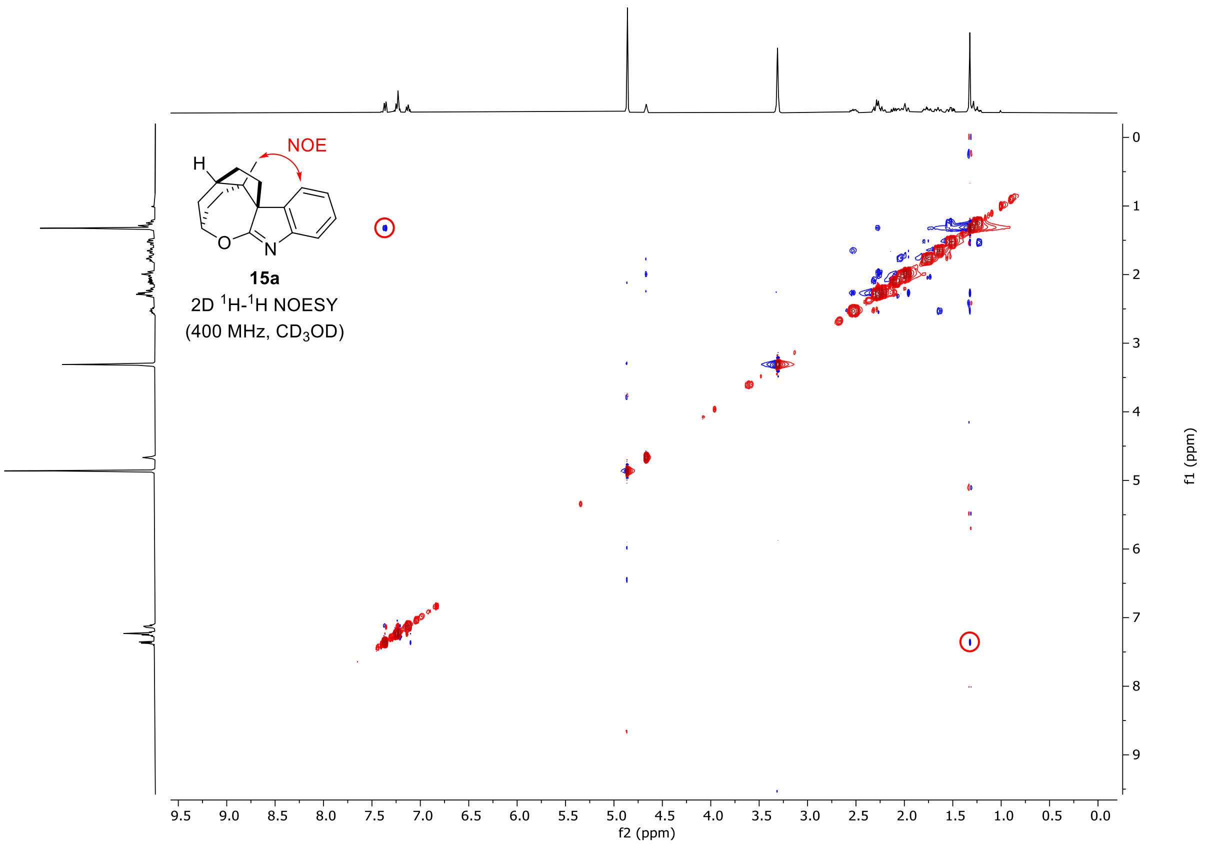
NOE
H
O
N
15a
2D ^{1}H-^{1}H NOESY
(400 MHz, CD_3OD)
f1 (ppm)
f2 (ppm)

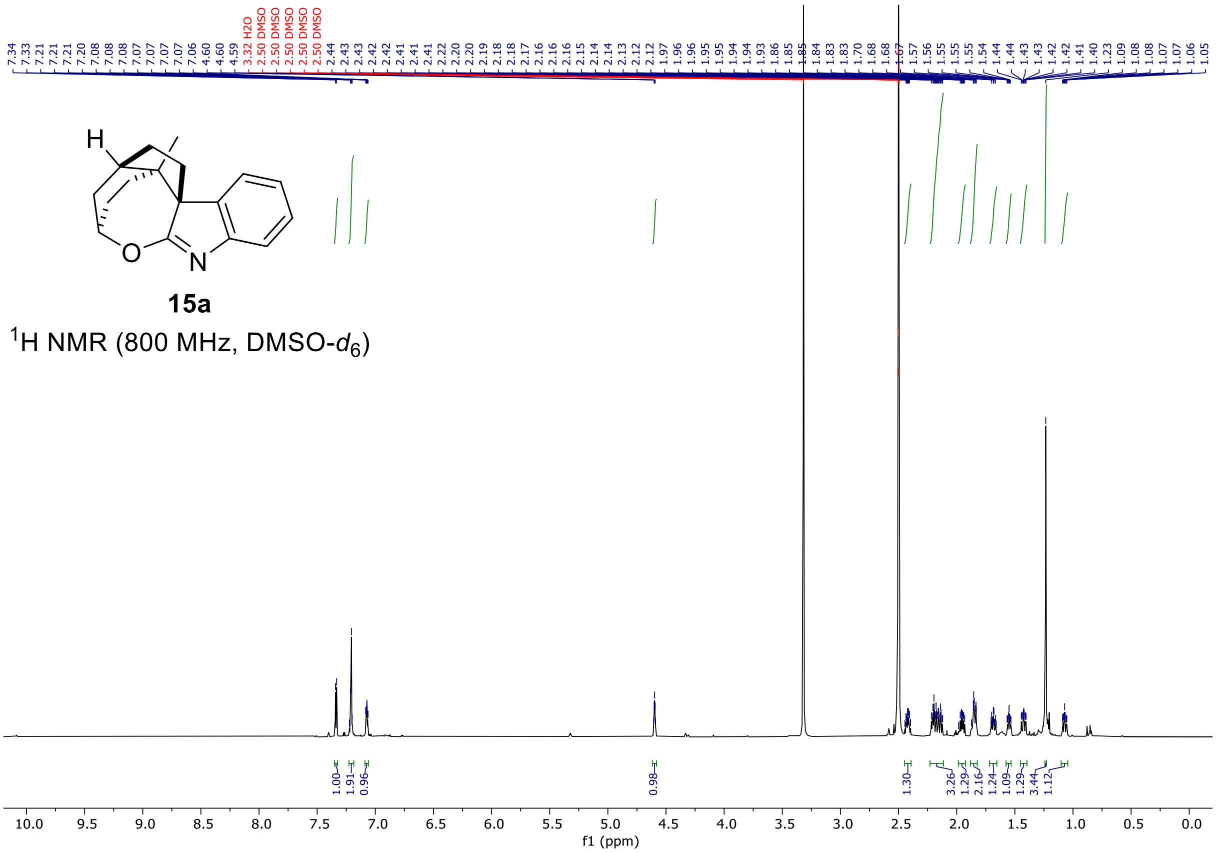

15a

^{1}H NMR (800 MHz, DMSO-d_6)

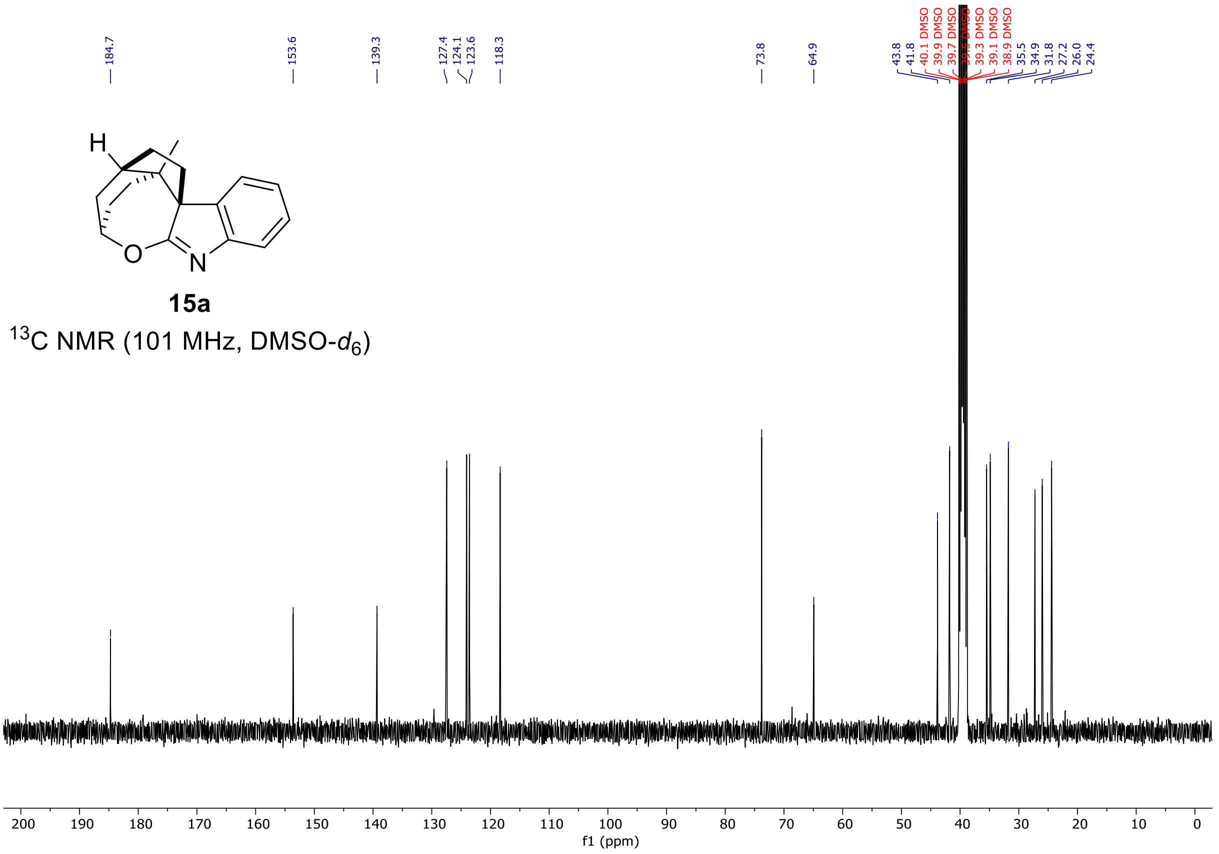

15a

^{13}C NMR (101 MHz, DMSO-d_6)

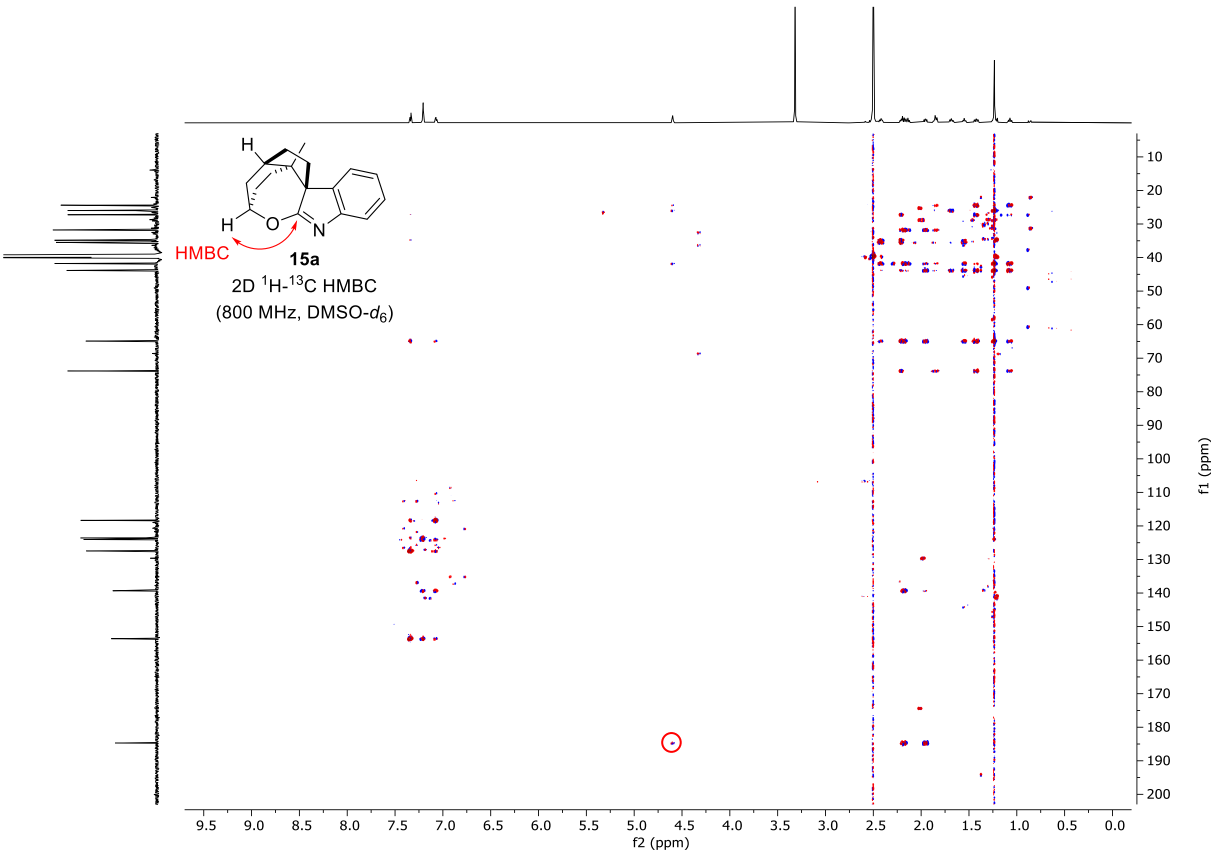
HMBC
15a
2D 1H-13C HMBC
(800 MHz, DMSO-d6)
f1 (ppm)
f2 (ppm)

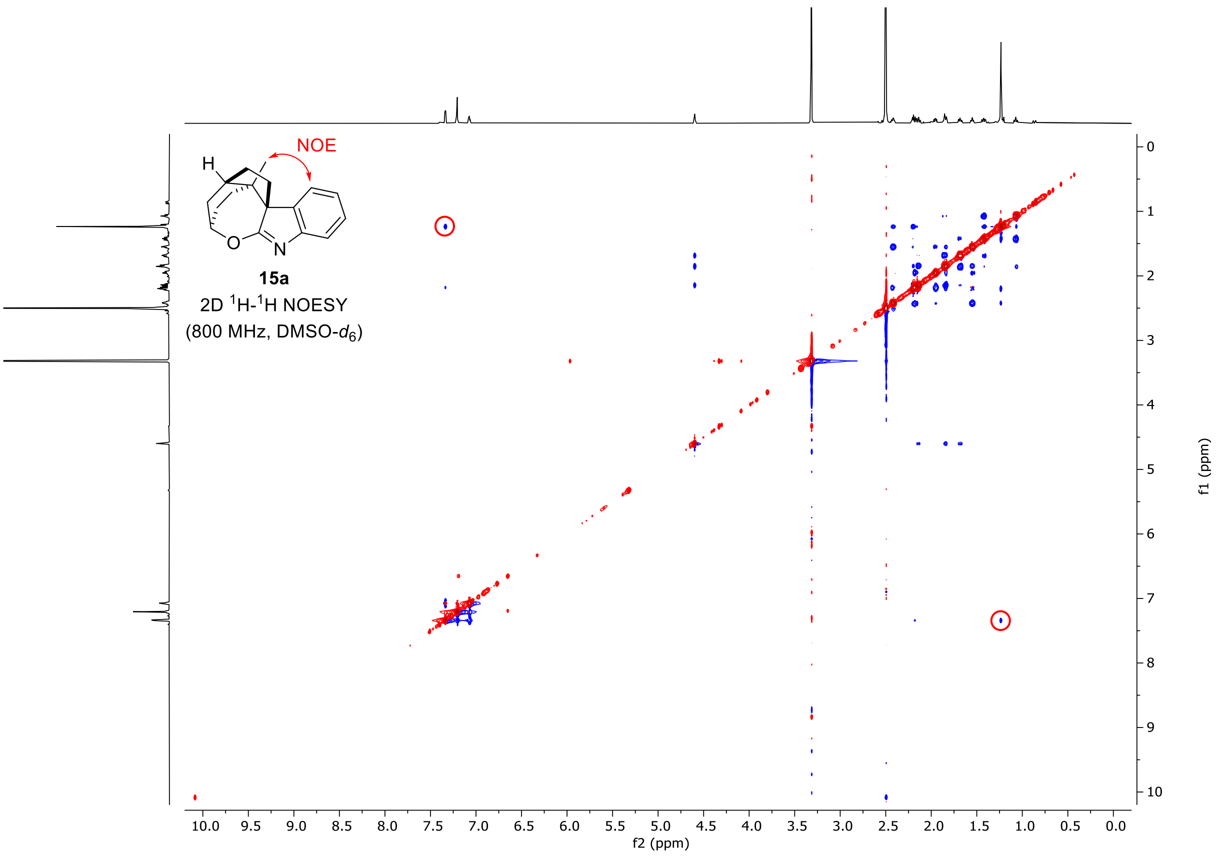
NOE
15a
2D 1H-1H NOESY
(800 MHz, DMSO-d6)
f1 (ppm)
f2 (ppm)

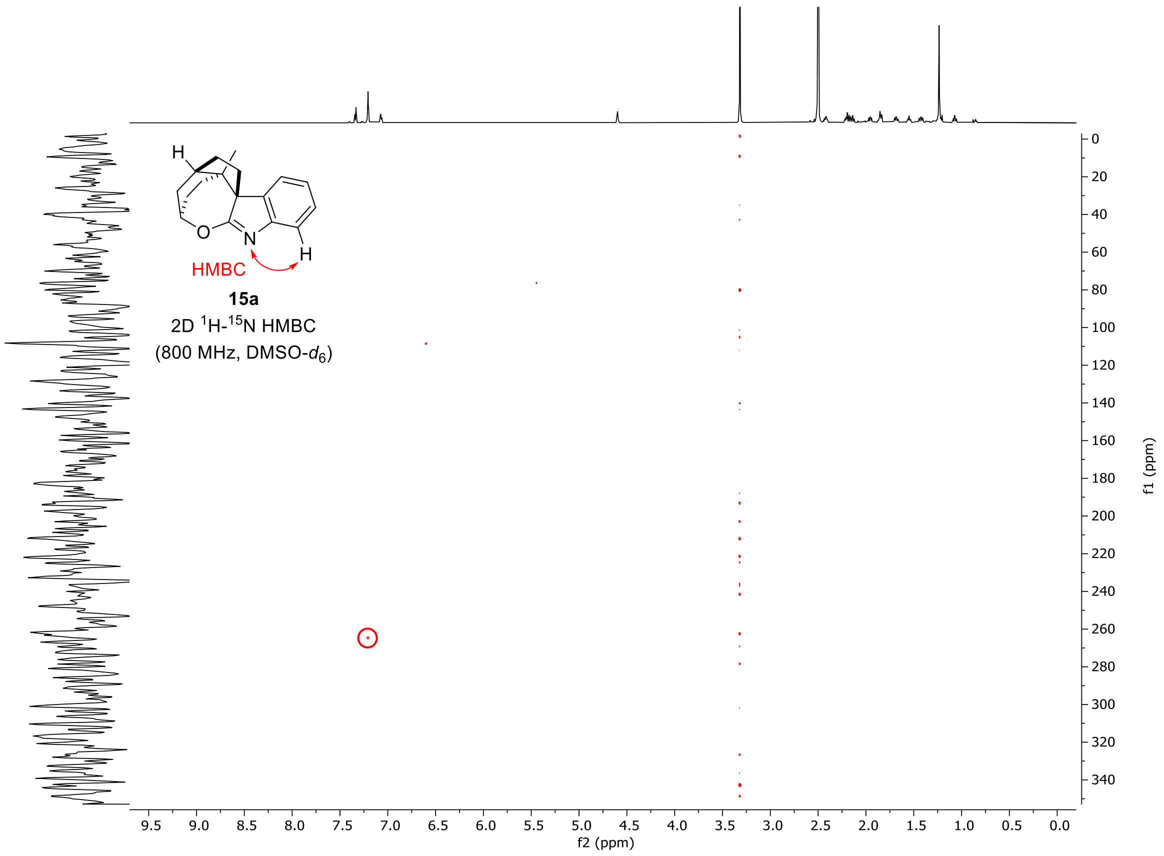

15a
2D ^{1}H-^{15}N HMBC
(800 MHz, DMSO-d_6)

(3*R,3a*S**,6*R**,12b*R**)-11-Bromo-3a-methyl-2,3,3a,4,5,6-hexahydro-1*H*-3,6-methanocyclopenta[3,4]oxepino[2,3-*b*]indole (15b)**

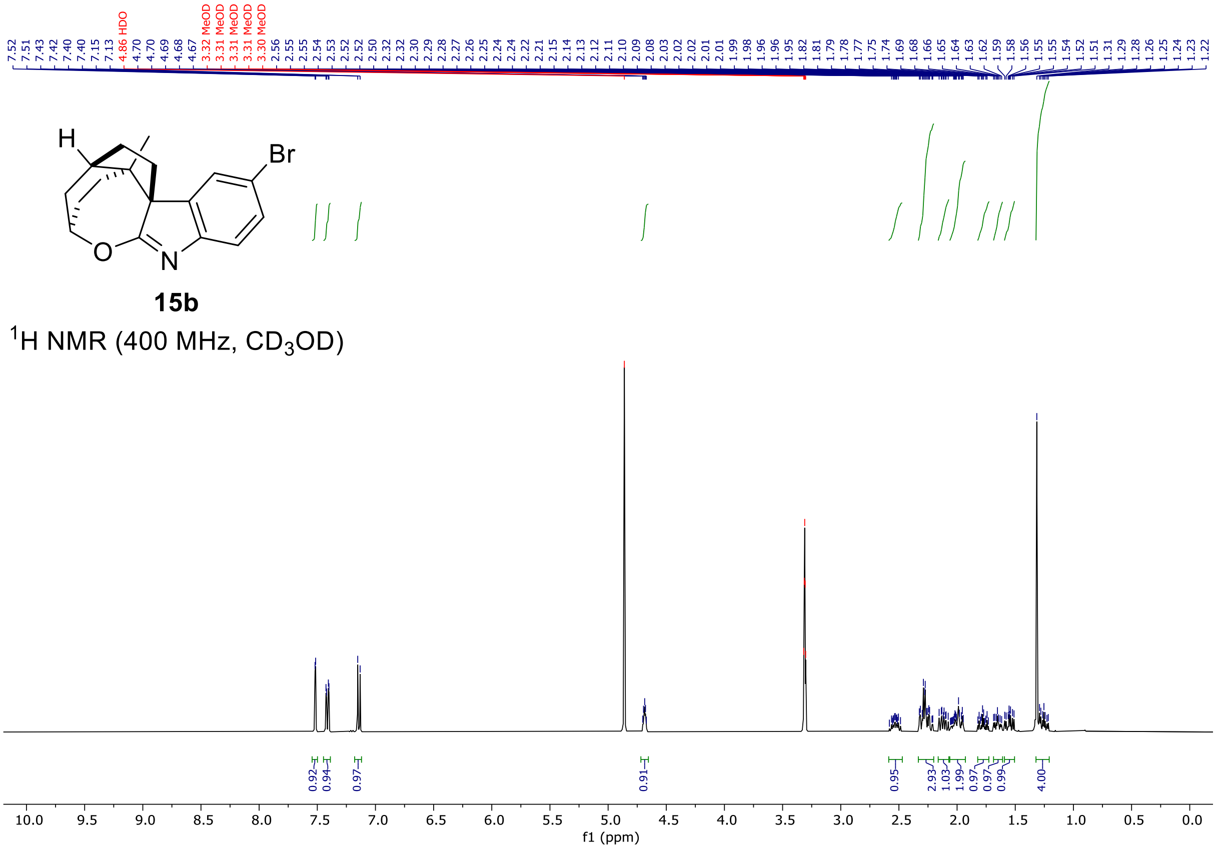

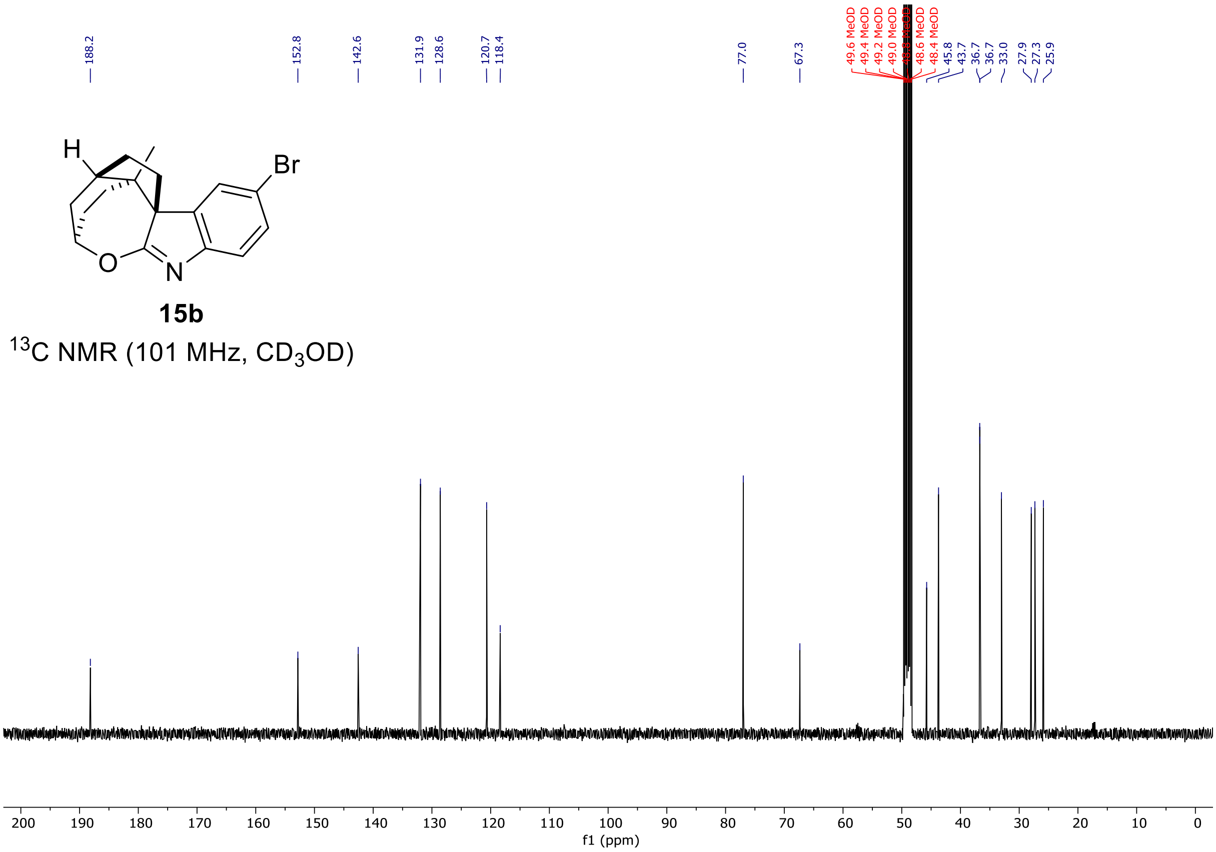

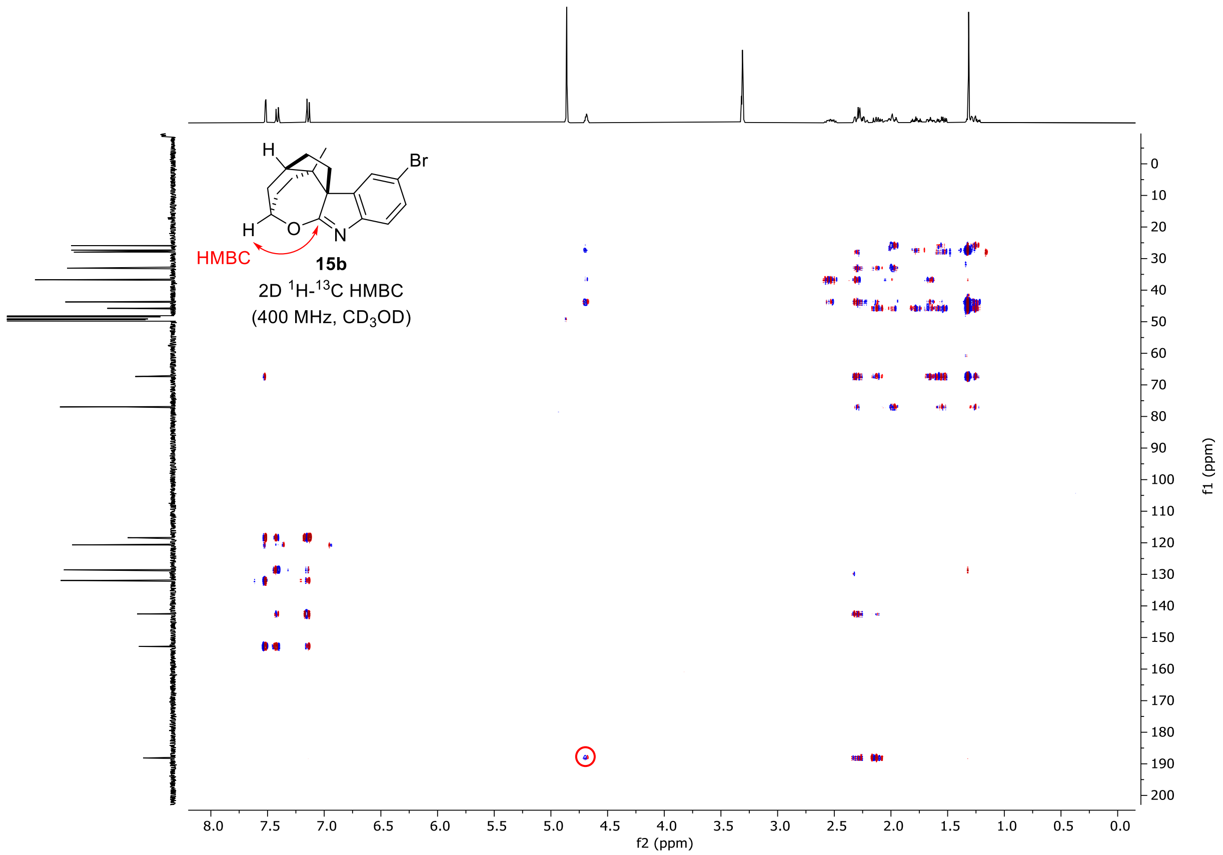
H
Br
H
O
N
HMBC
15b
2D ¹H-¹³C HMBC
(400 MHz, CD3OD)
f1 (ppm)
f2 (ppm)

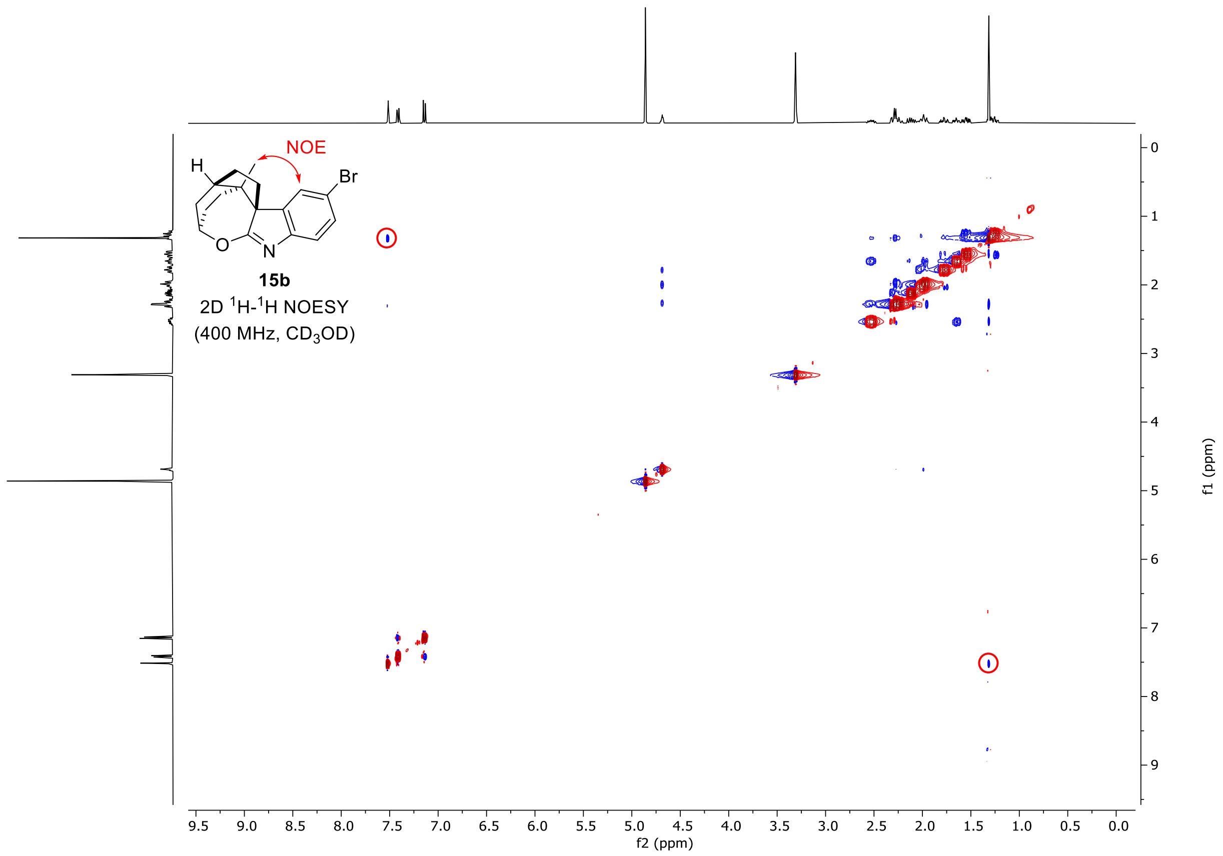
NOE
H
Br
O
N
15b
2D ¹H-¹H NOESY
(400 MHz, CD3OD)
f1 (ppm)
f2 (ppm)

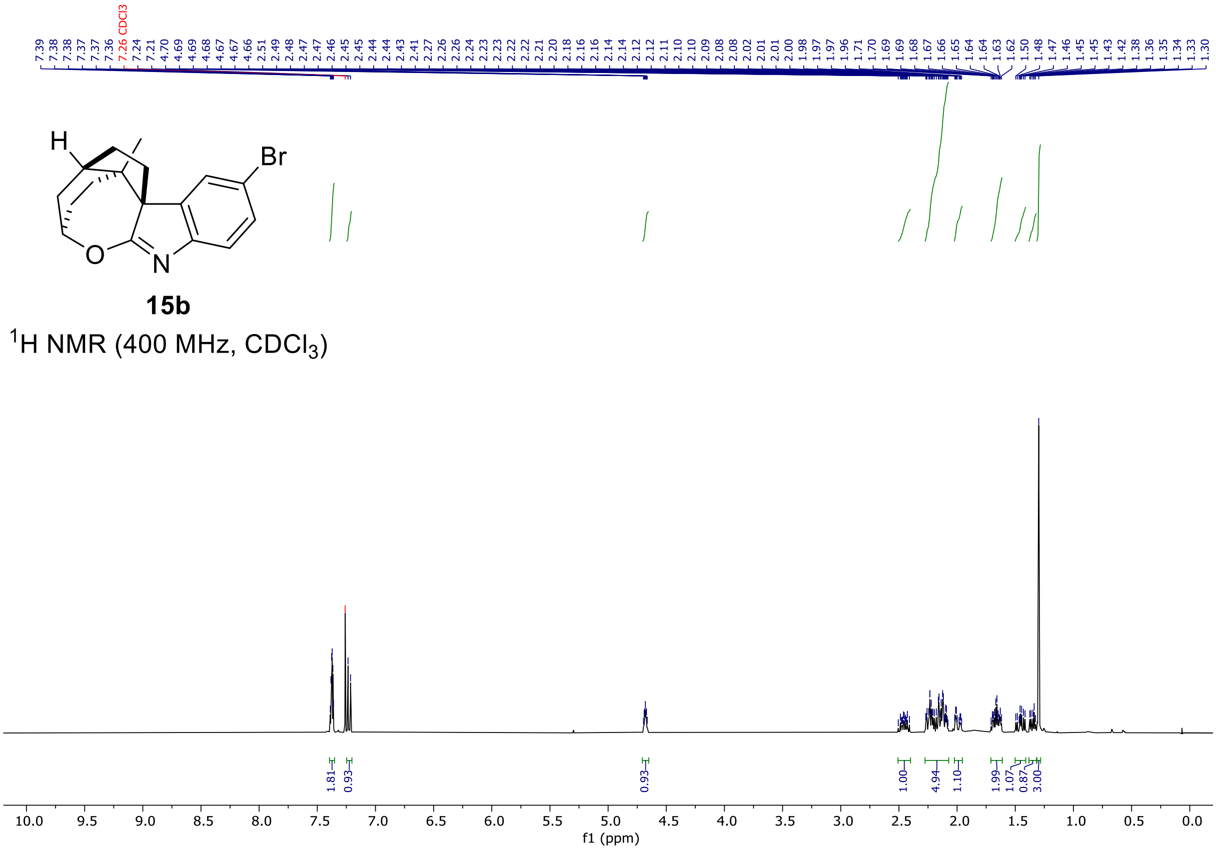

15b

^{1}H NMR (400 MHz, $CDCl_3$)

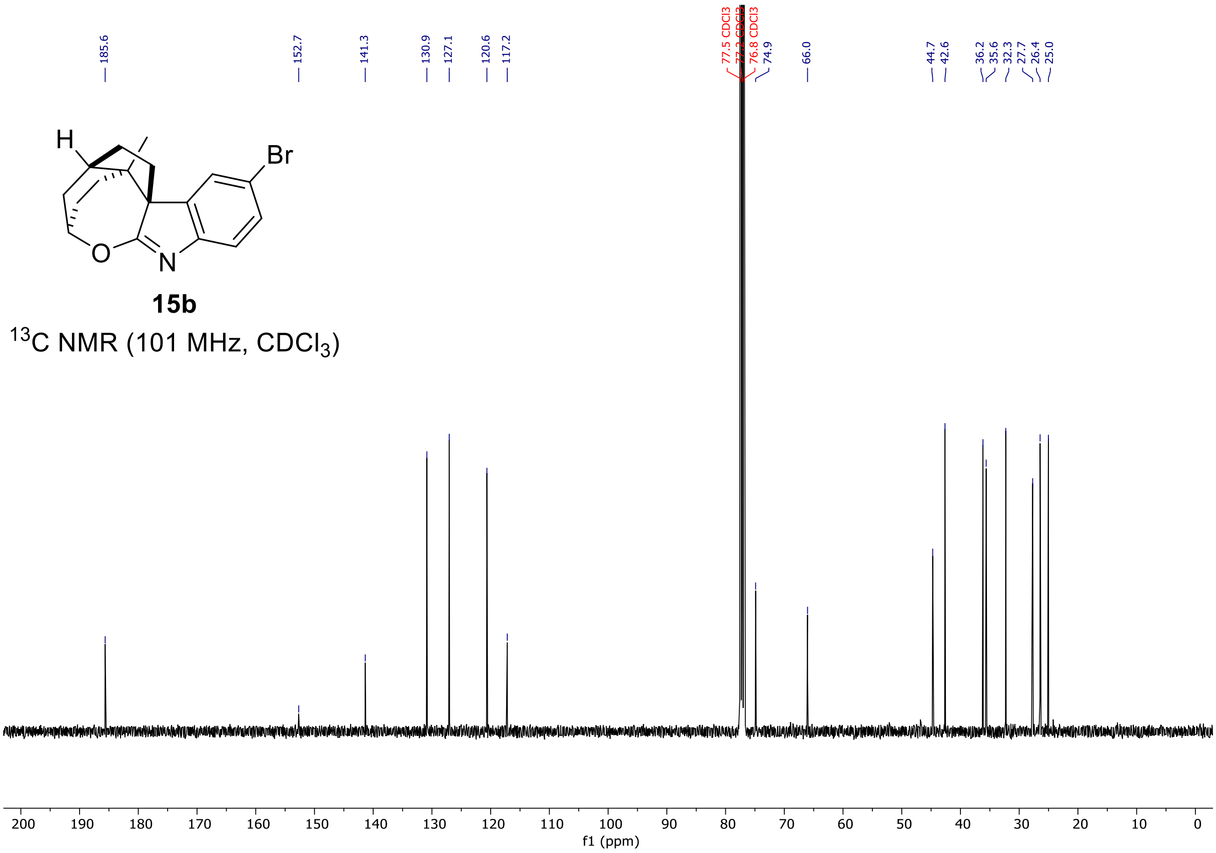

15b

^{13}C NMR (101 MHz, $CDCl_3$)

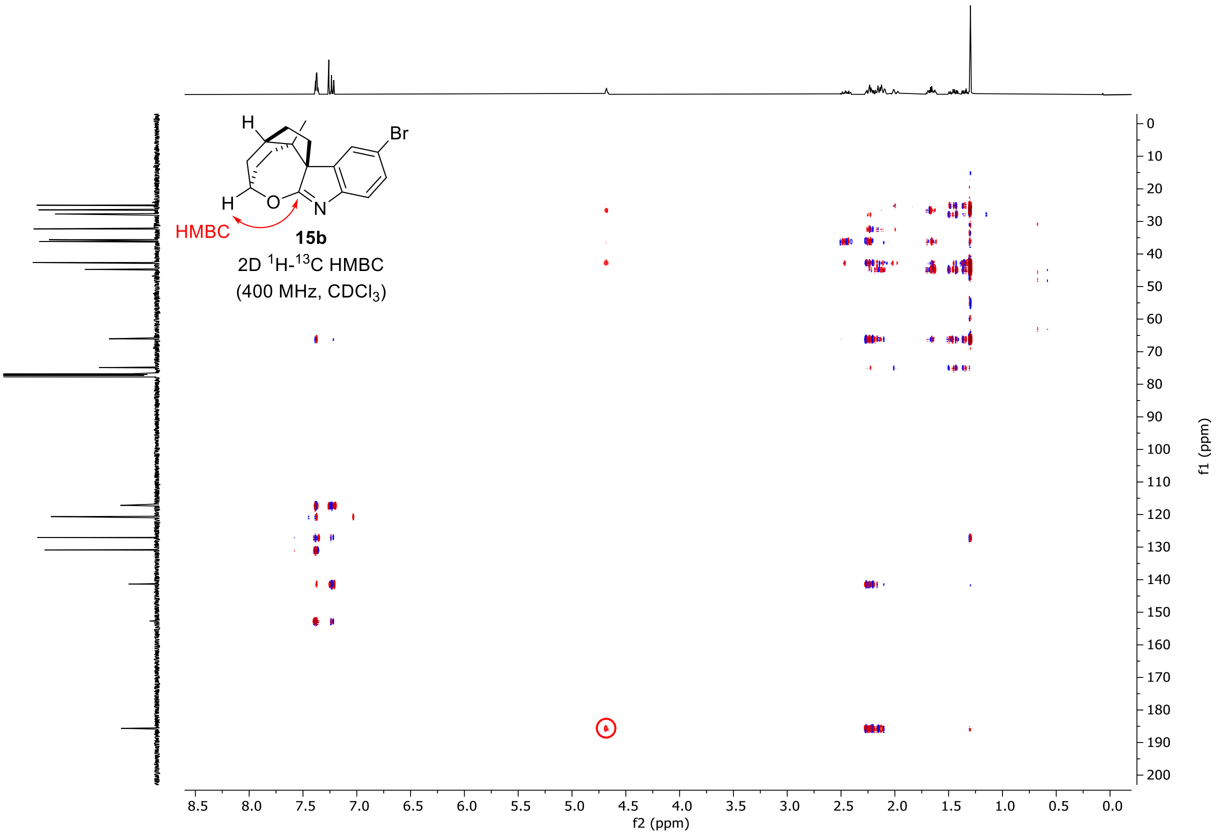

Br
H
H
O
N
HMBC
15b
2D ¹H-¹³C HMBC
(400 MHz, CDCl₃)
f2 (ppm)
f1 (ppm)

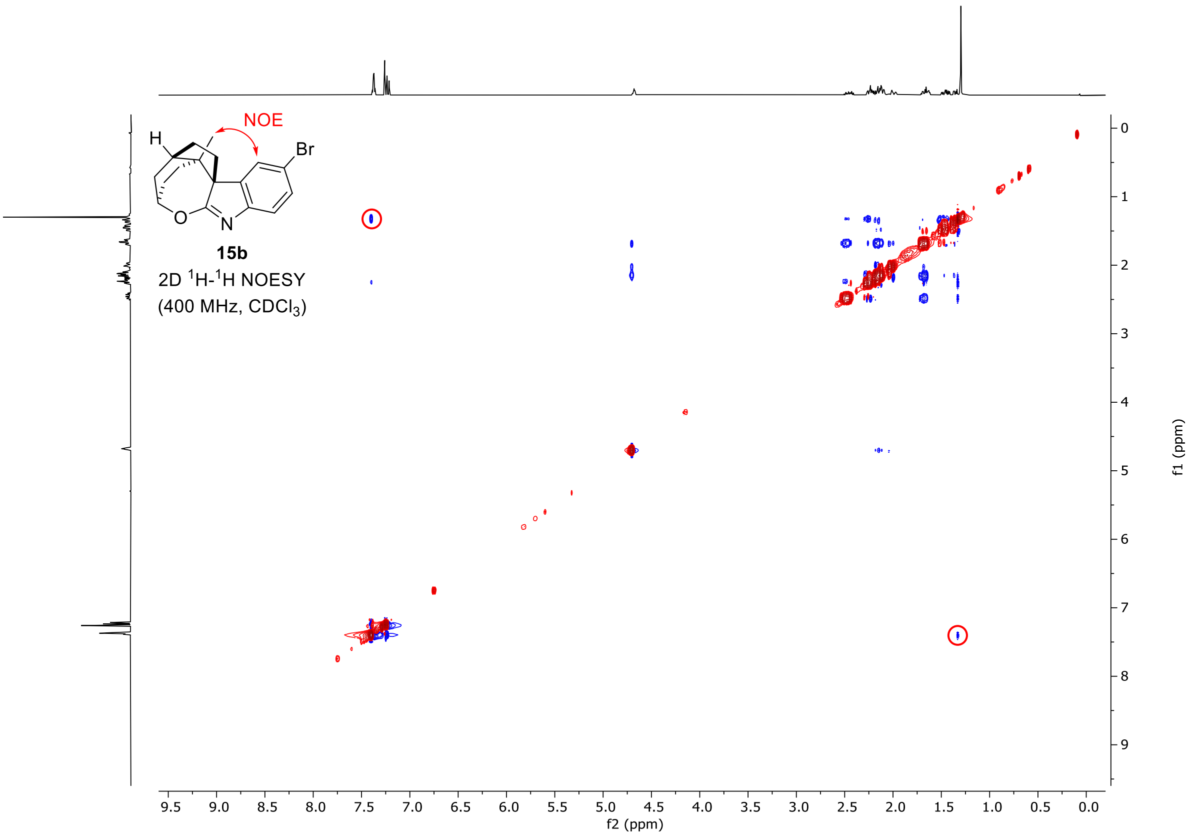

NOE
H
Br
O
N
15b
2D ¹H-¹H NOESY
(400 MHz, CDCl₃)
f2 (ppm)
f1 (ppm)

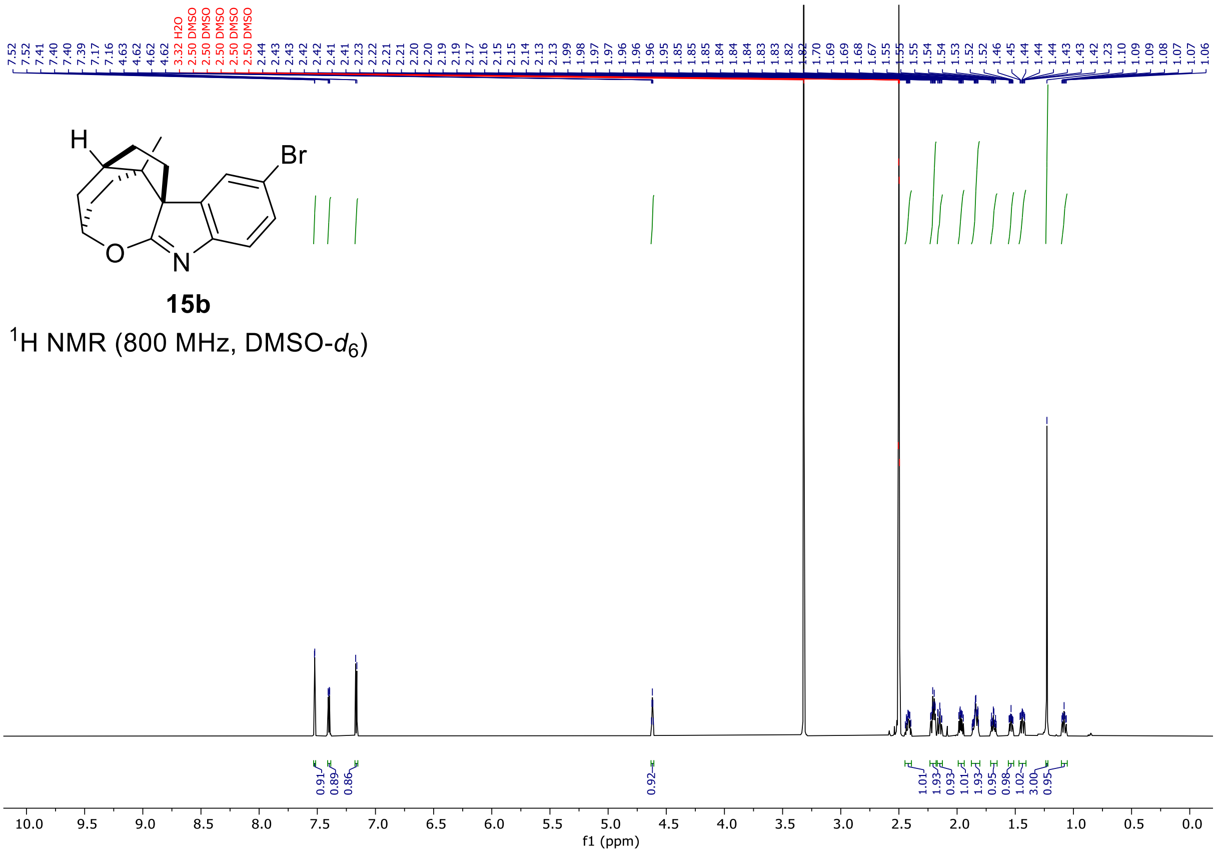

15b

^{1}H NMR (800 MHz, DMSO-d_6)

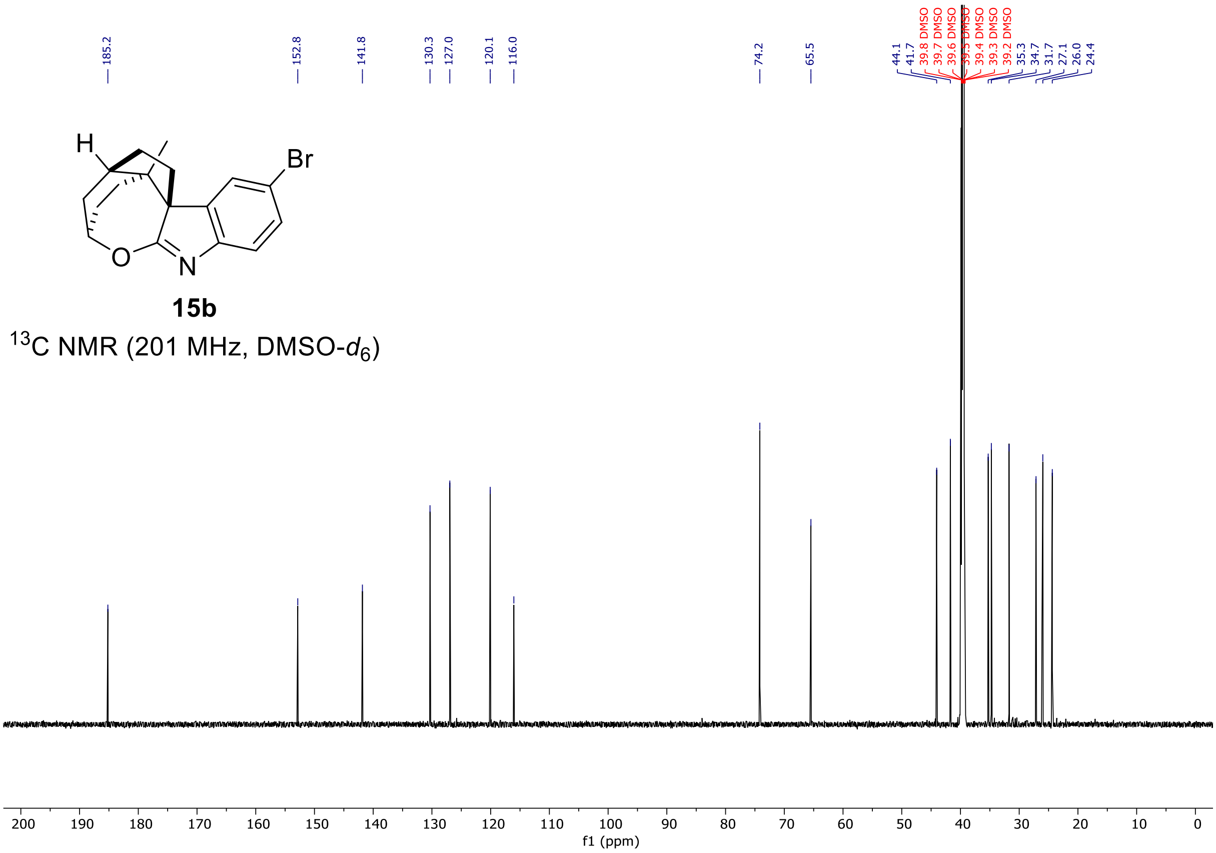

15b

^{13}C NMR (201 MHz, DMSO-d_6)

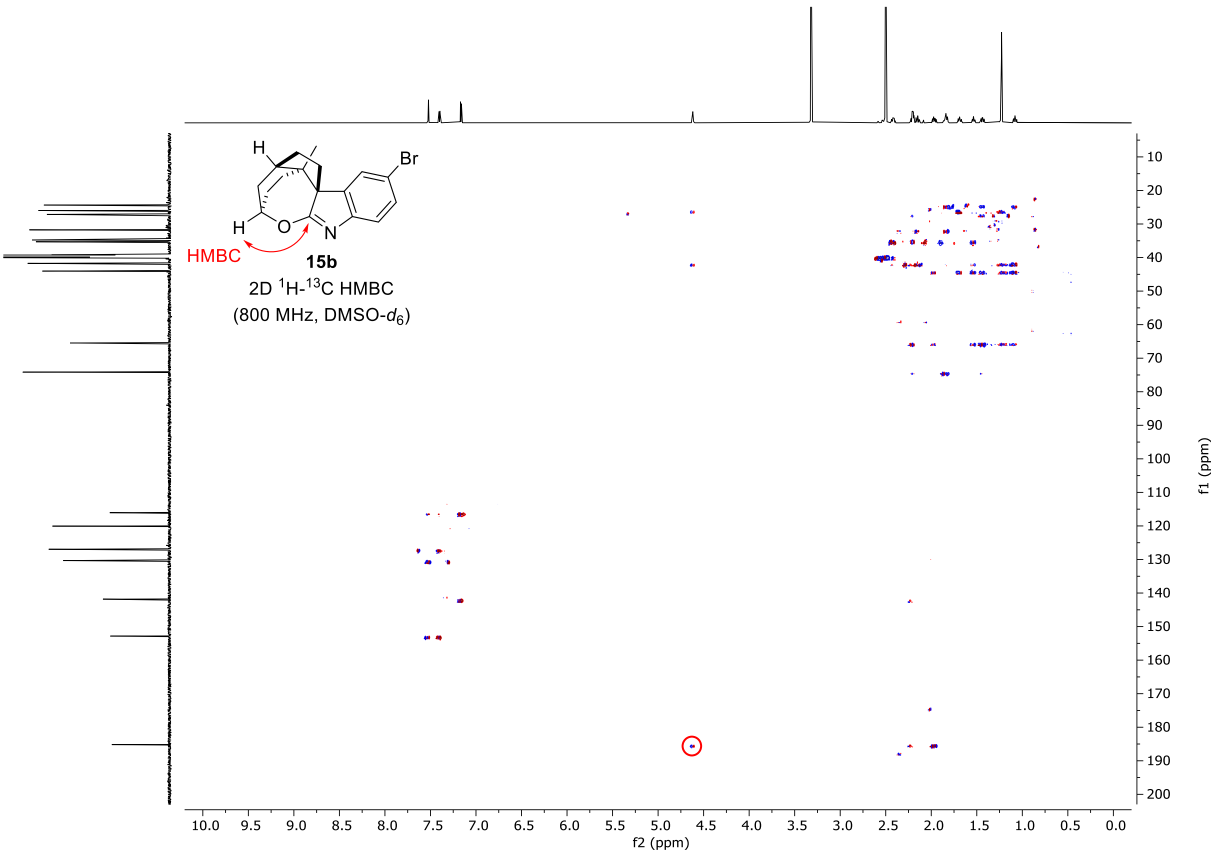

H
Br
H
O
N
HMBC
15b
2D 1H-13C HMBC
(800 MHz, DMSO-d6)
f2 (ppm)
f1 (ppm)

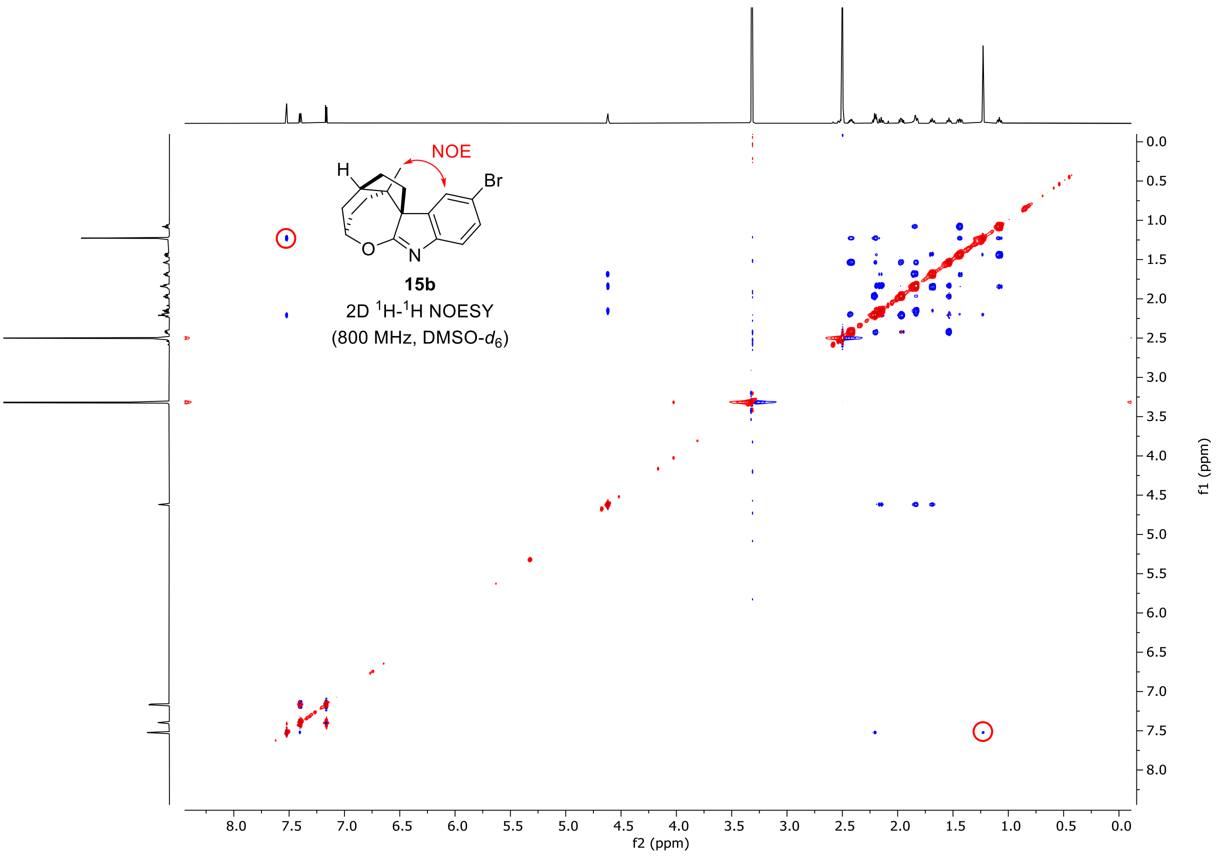

NOE
H
Br
O
N
15b
2D 1H-1H NOESY
(800 MHz, DMSO-d6)
f2 (ppm)
f1 (ppm)

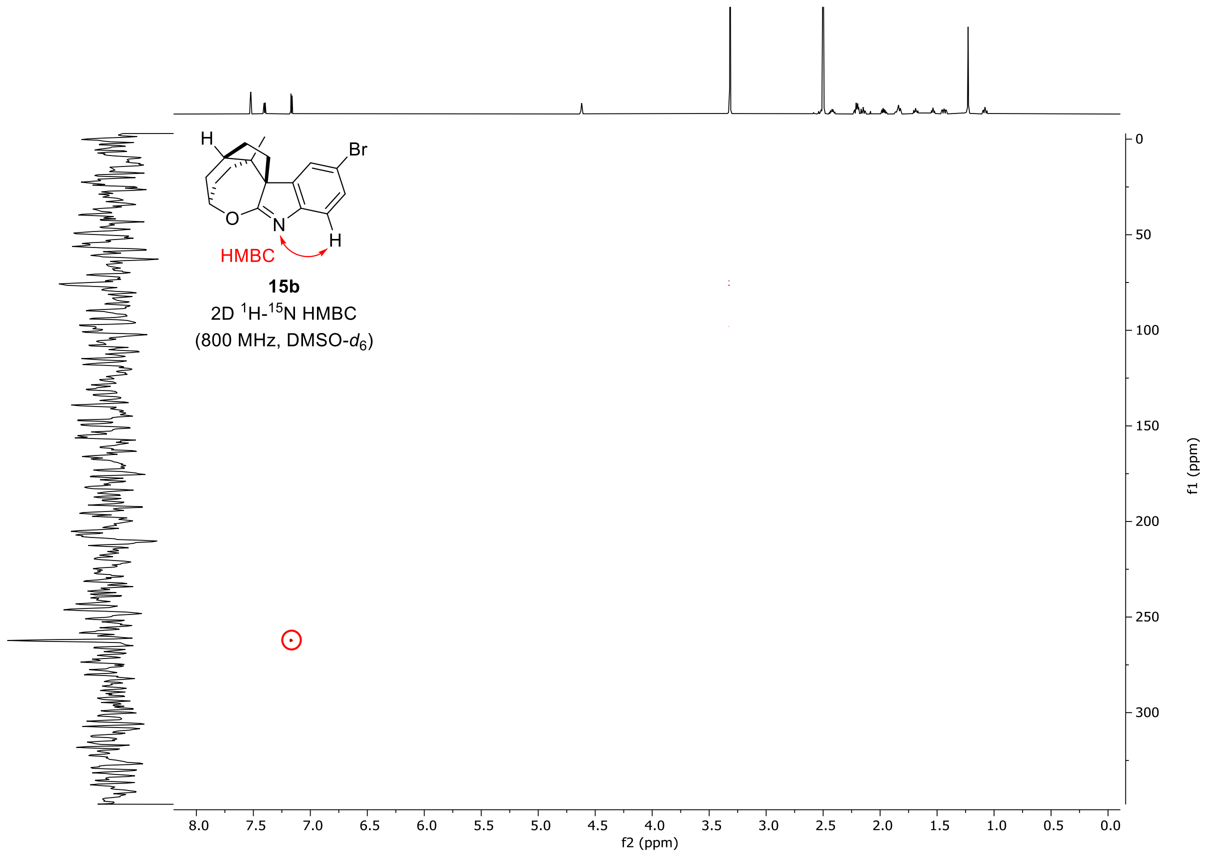
H
Br
O
N
H
HMBC
15b
2D 1H-15N HMBC
(800 MHz, DMSO-d6)
f1 (ppm)
f2 (ppm)

(3*R,3a*S**,6*R**,12b*R**)-11-Chloro-3a-methyl-2,3,3a,4,5,6-hexahydro-1*H*-3,6-methanocyclopenta[3,4]oxepino[2,3-*b*]indole (15c)**

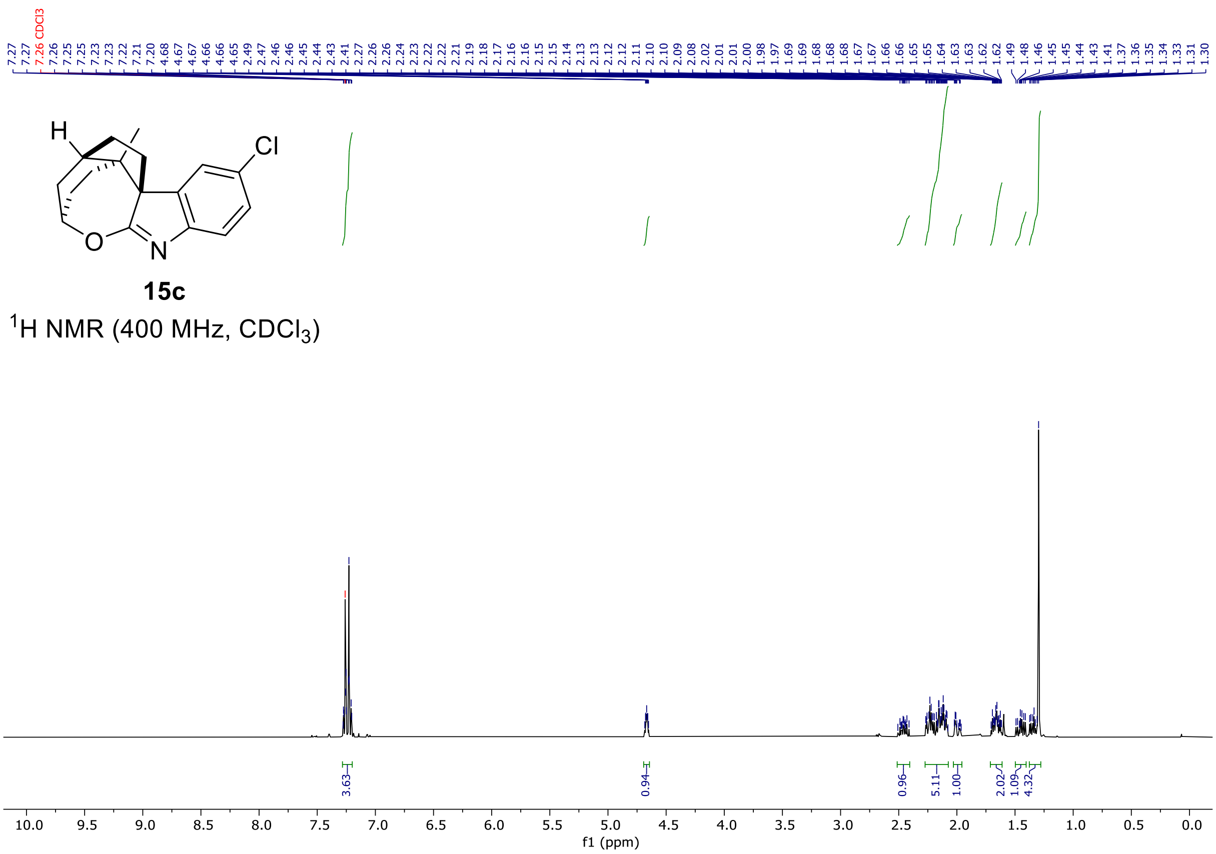

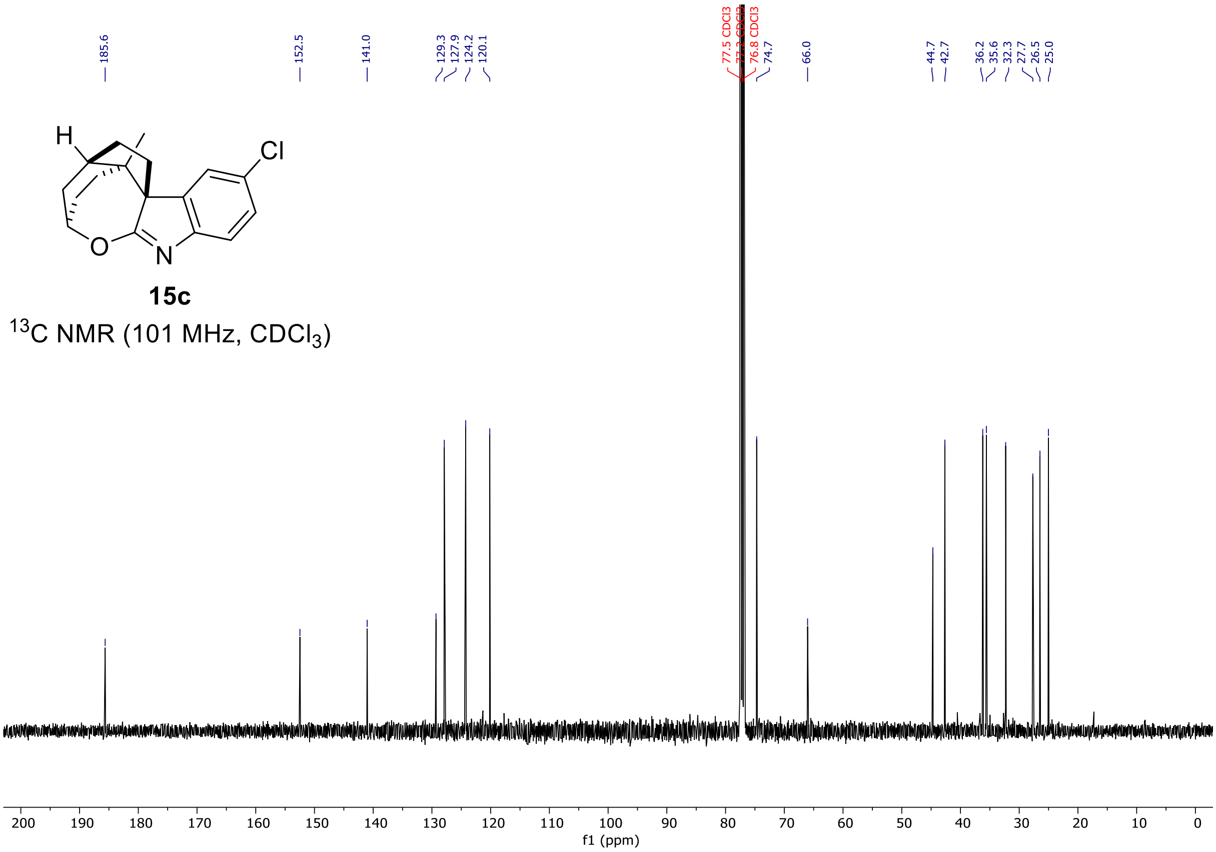

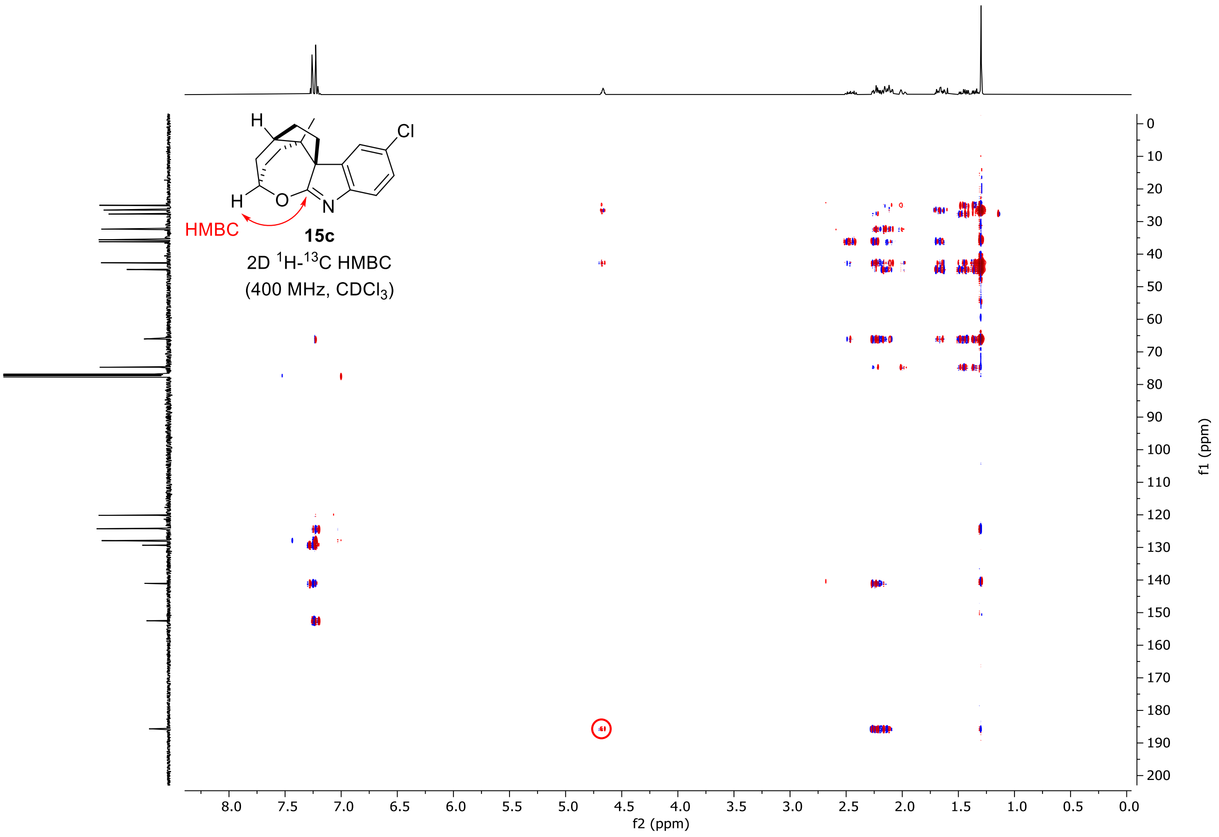
H
Cl
H
O
N
HMBC
15c
2D ¹H-¹³C HMBC
(400 MHz, CDCl3)
f1 (ppm)
f2 (ppm)

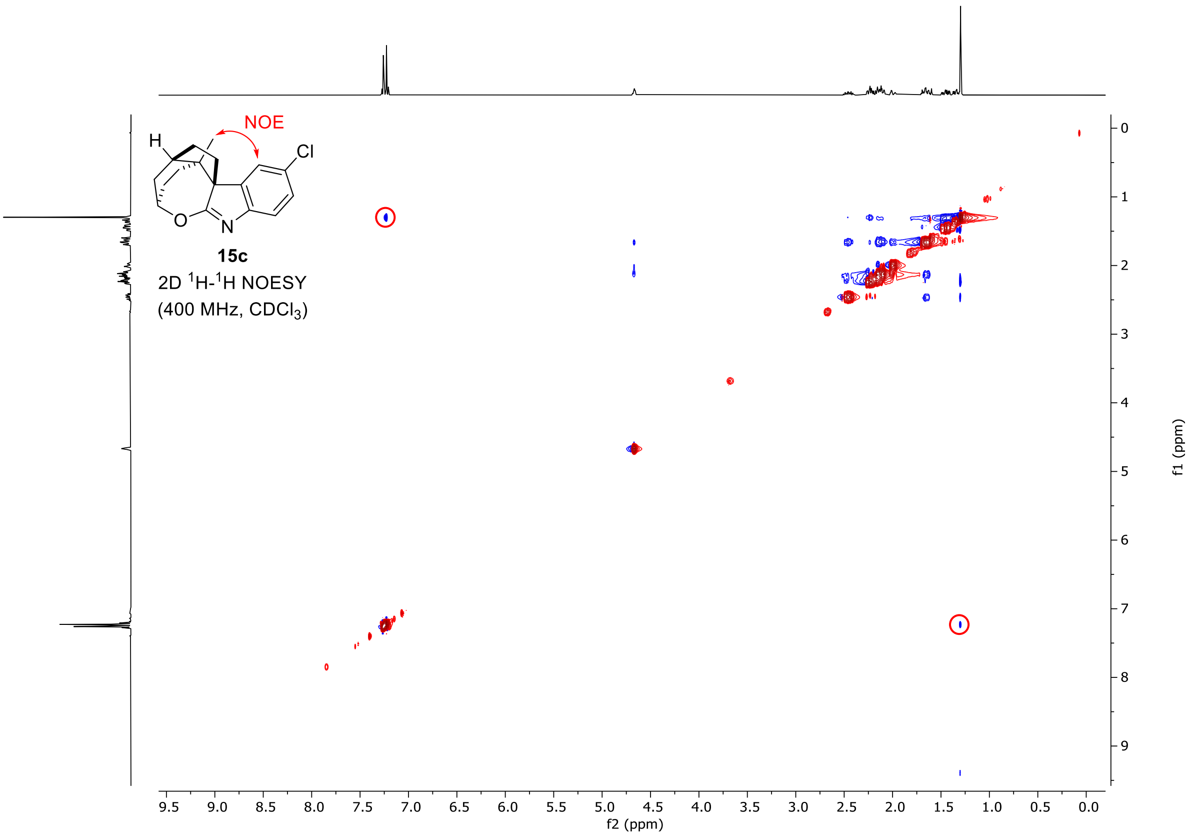
NOE
H
Cl
O
N
15c
2D ¹H-¹H NOESY
(400 MHz, CDCl3)
f1 (ppm)
f2 (ppm)

(3*R,3a*S**,6*R**,12b*R**)-11-Fluoro-3a-methyl-2,3,3a,4,5,6-hexahydro-1*H*-3,6-methanocyclopenta[3,4]oxepino[2,3-*b*]indole (15d)**

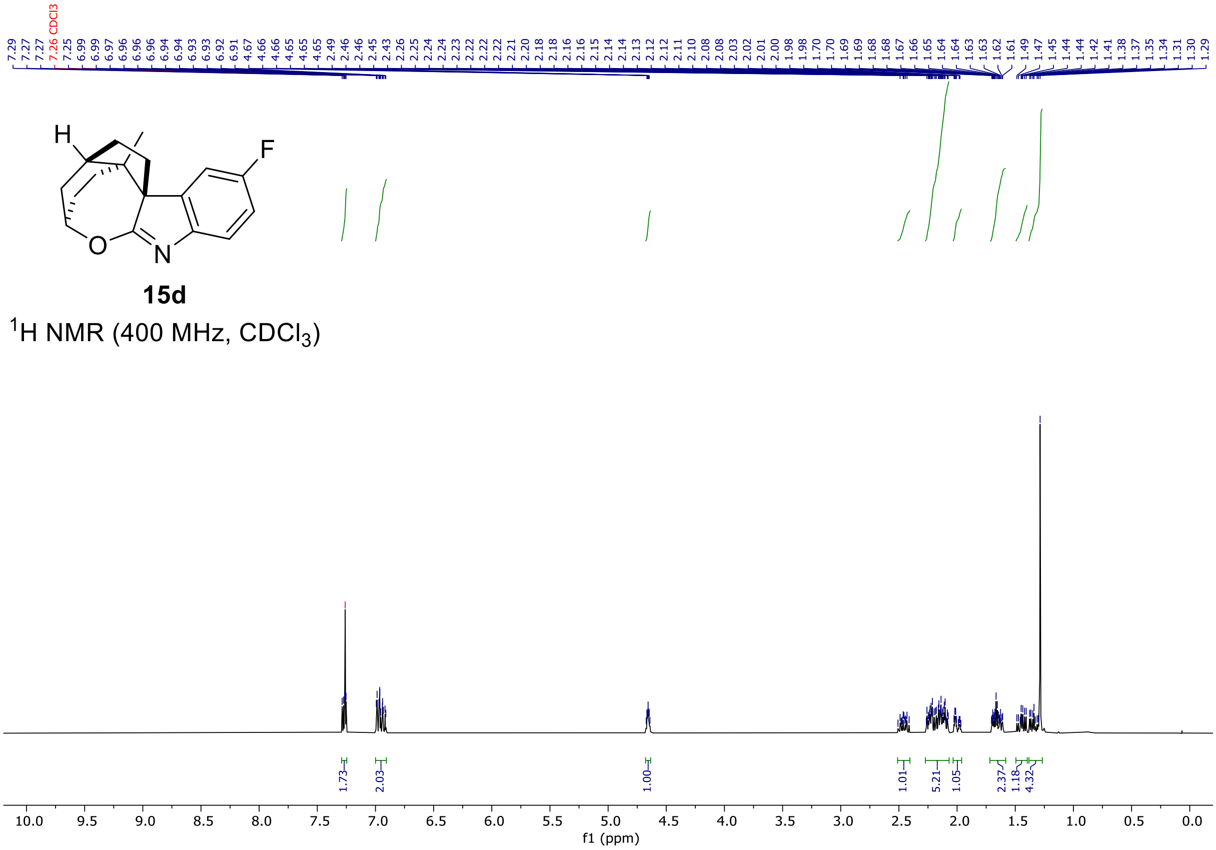

15d

^{1}H NMR (400 MHz, $CDCl_3$)

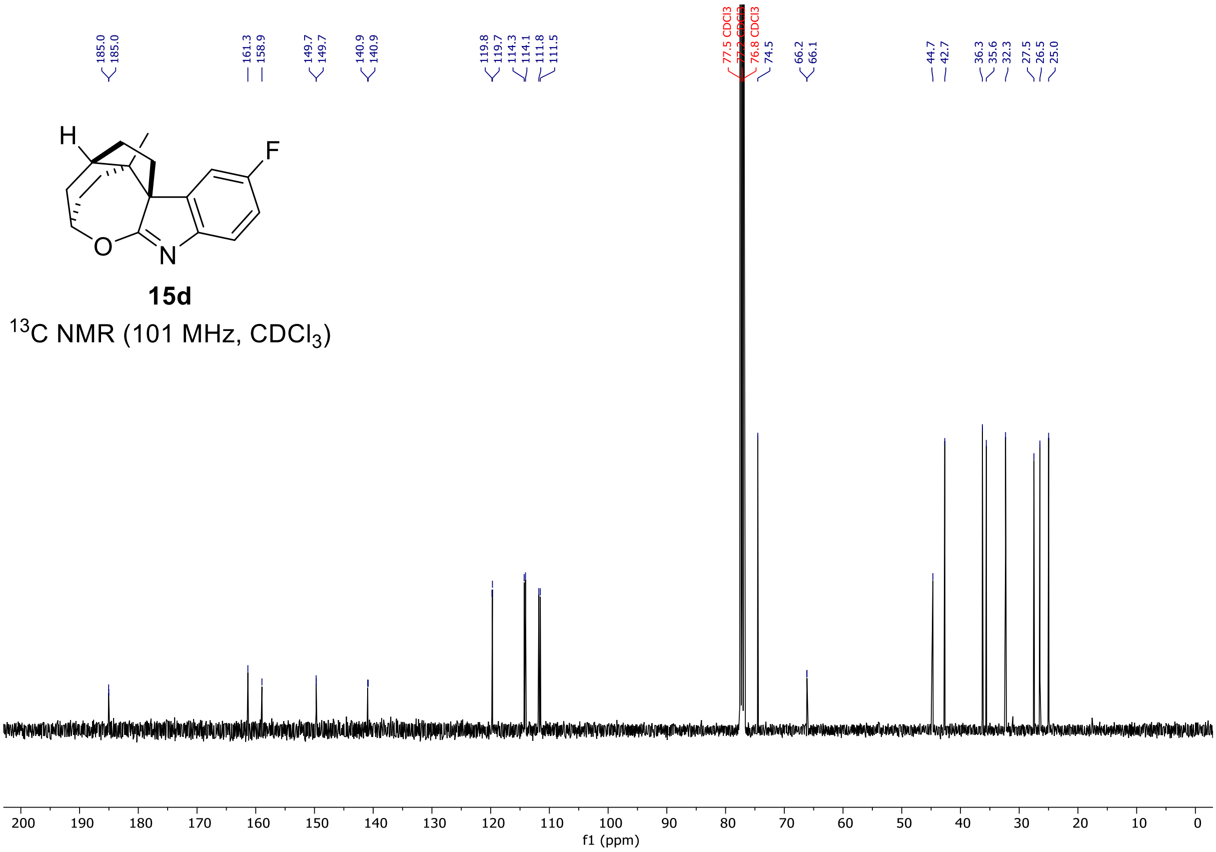

15d

^{13}C NMR (101 MHz, $CDCl_3$)

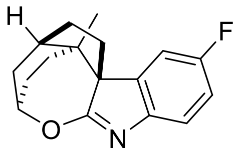

15d

^{19}F NMR (377 MHz, $CDCl_3$)

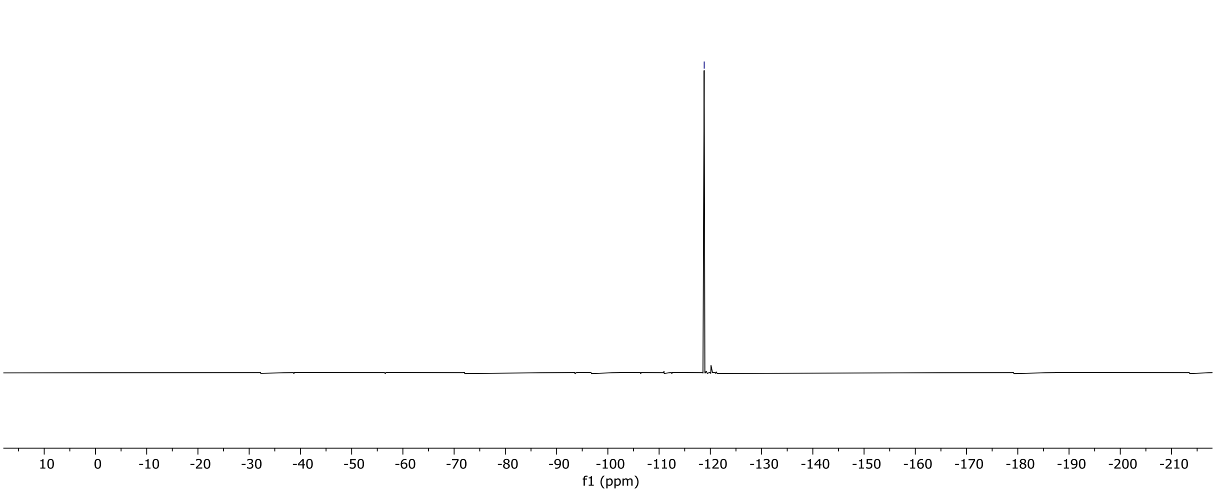

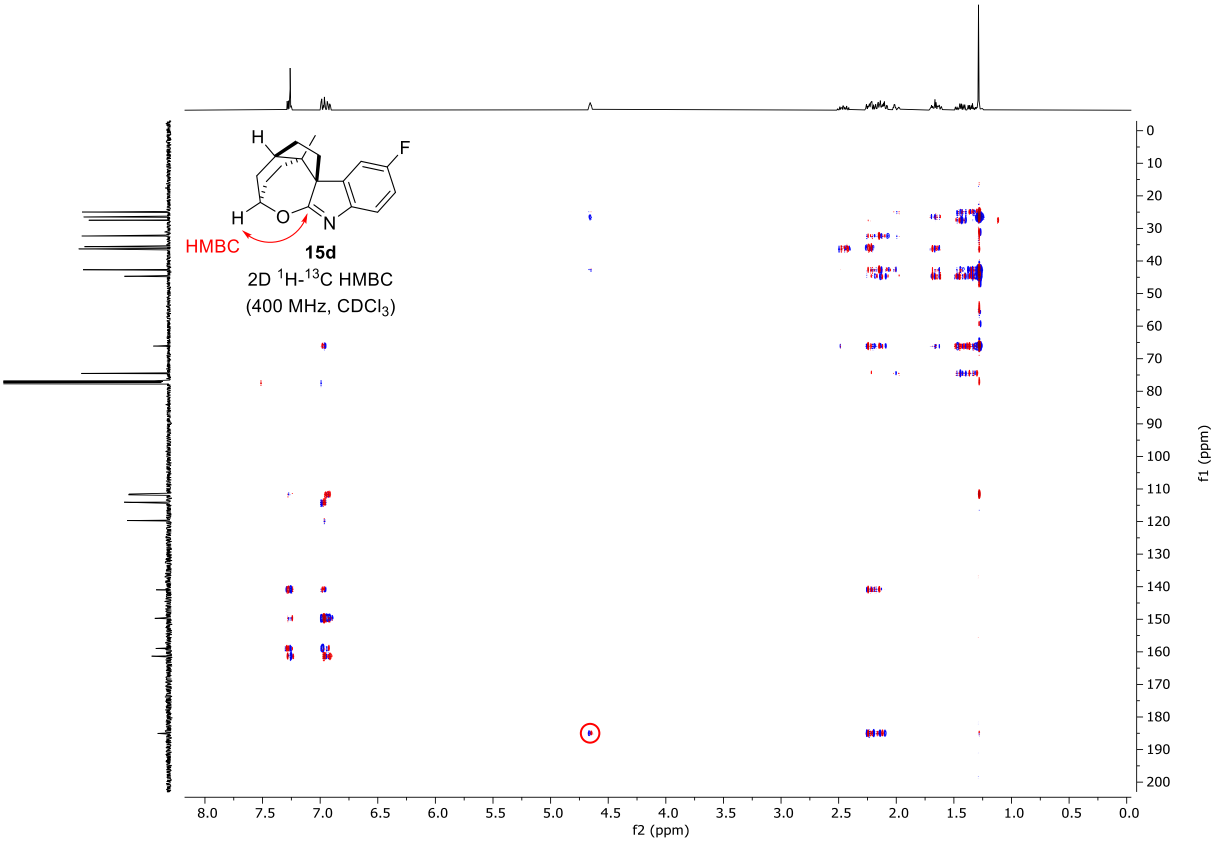

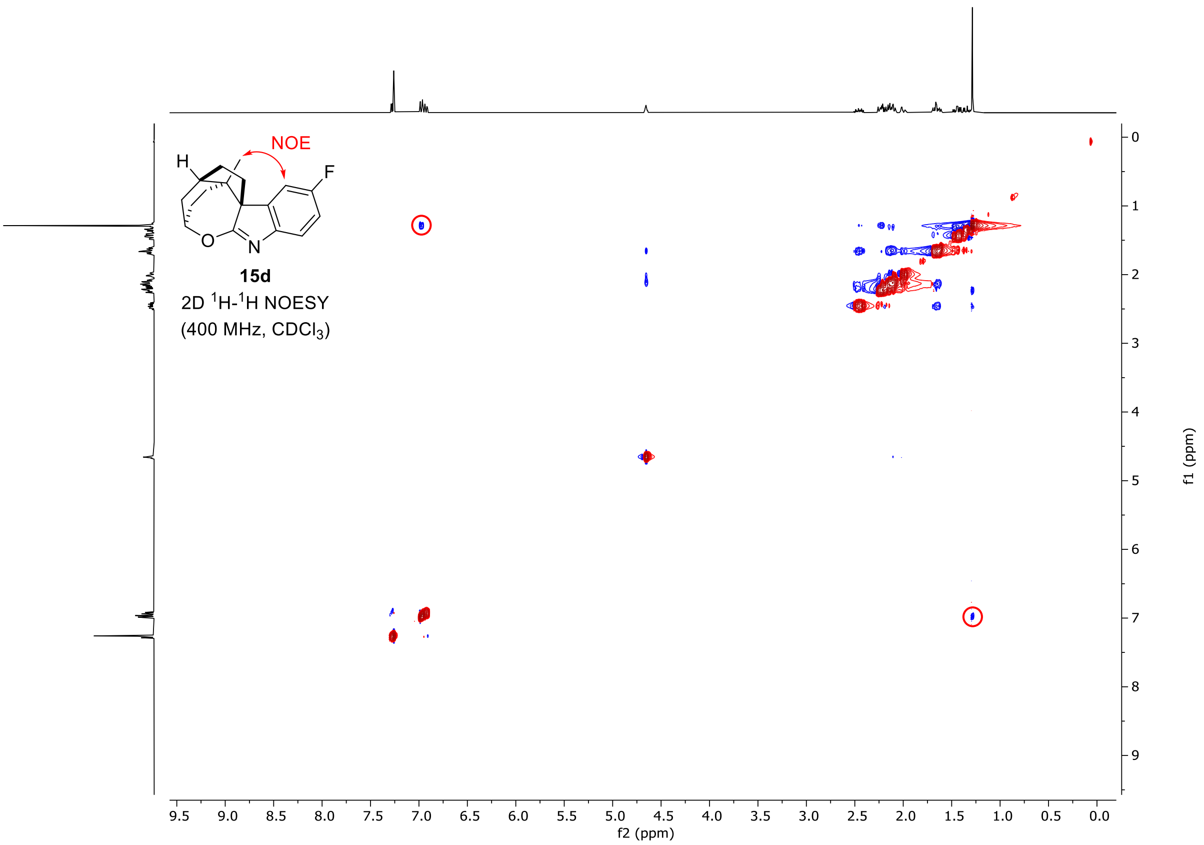

NOE
H
F
O
N
15d
2D 1H-1H NOESY
(400 MHz, CDCl3)
f1 (ppm)
f2 (ppm)

(3*R,3a*S**,6*R**,12b*R**)-3a,11-Dimethyl-2,3,3a,4,5,6-hexahydro-1*H*-3,6-methanocyclopenta[3,4]oxepino[2,3-*b*]indole (15e)**

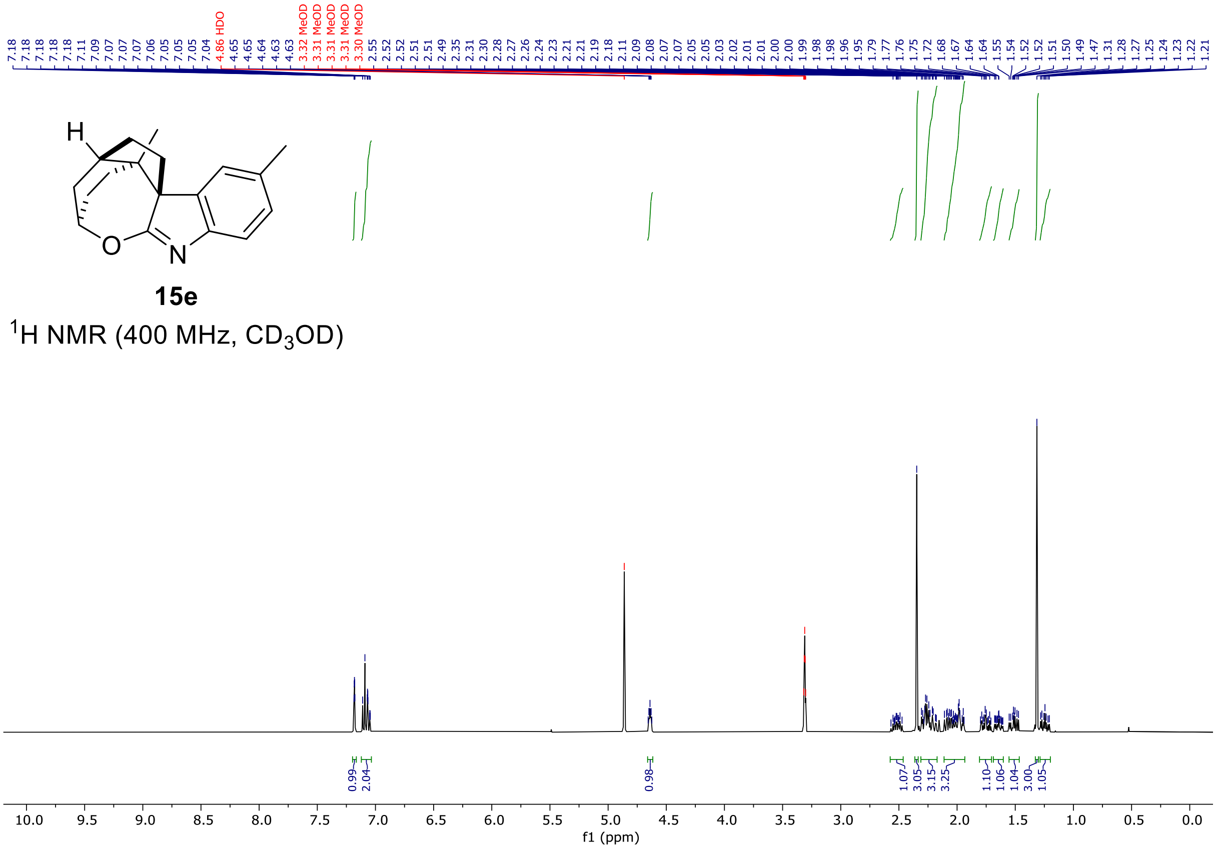

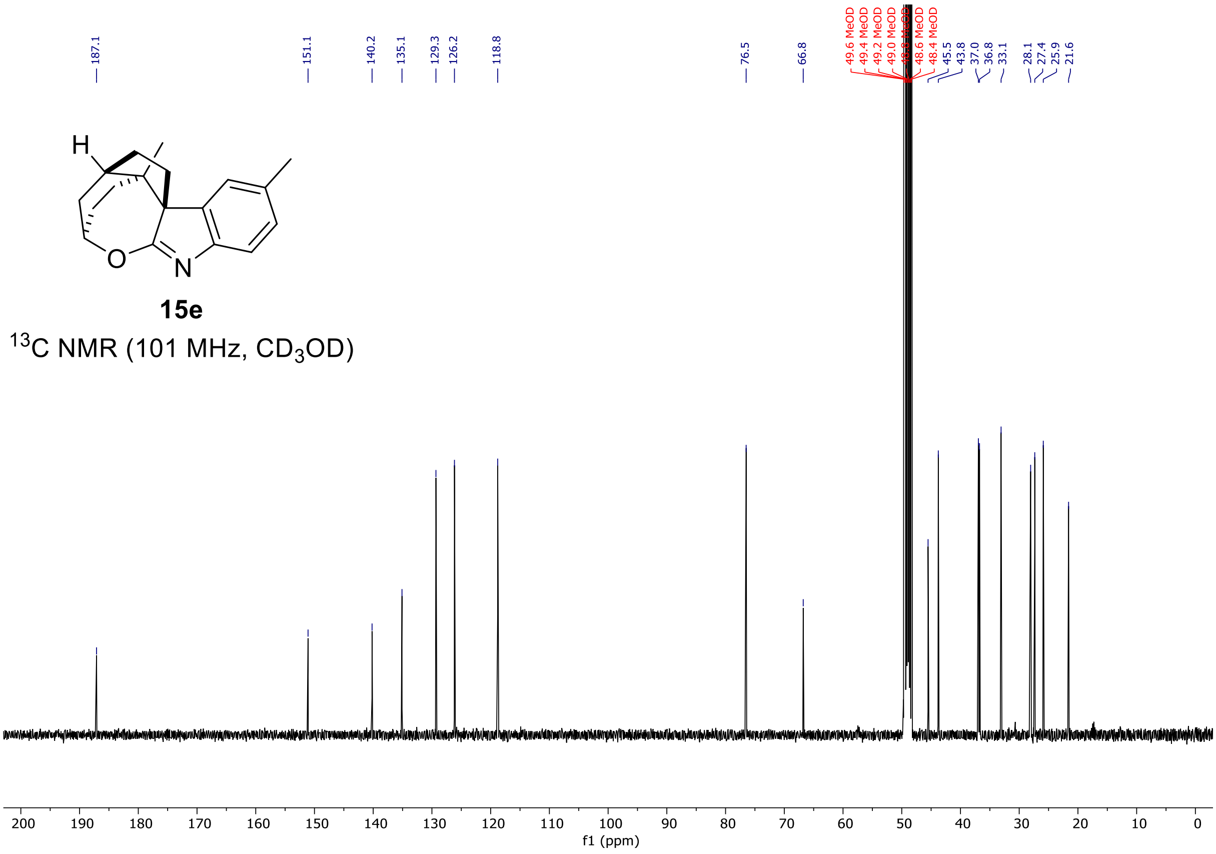

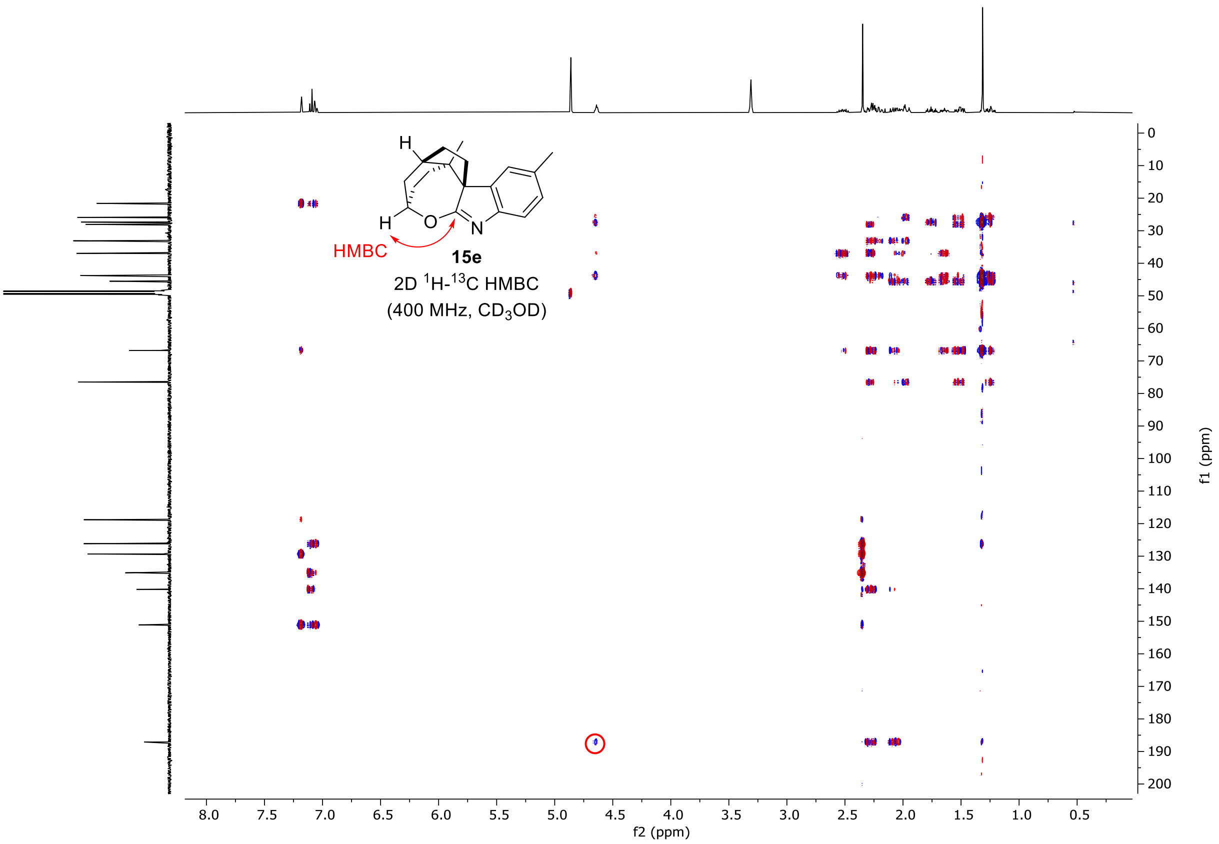
H
H
O
N
HMBC
15e
2D 1H-13C HMBC
(400 MHz, CD3OD)
f2 (ppm)
f1 (ppm)

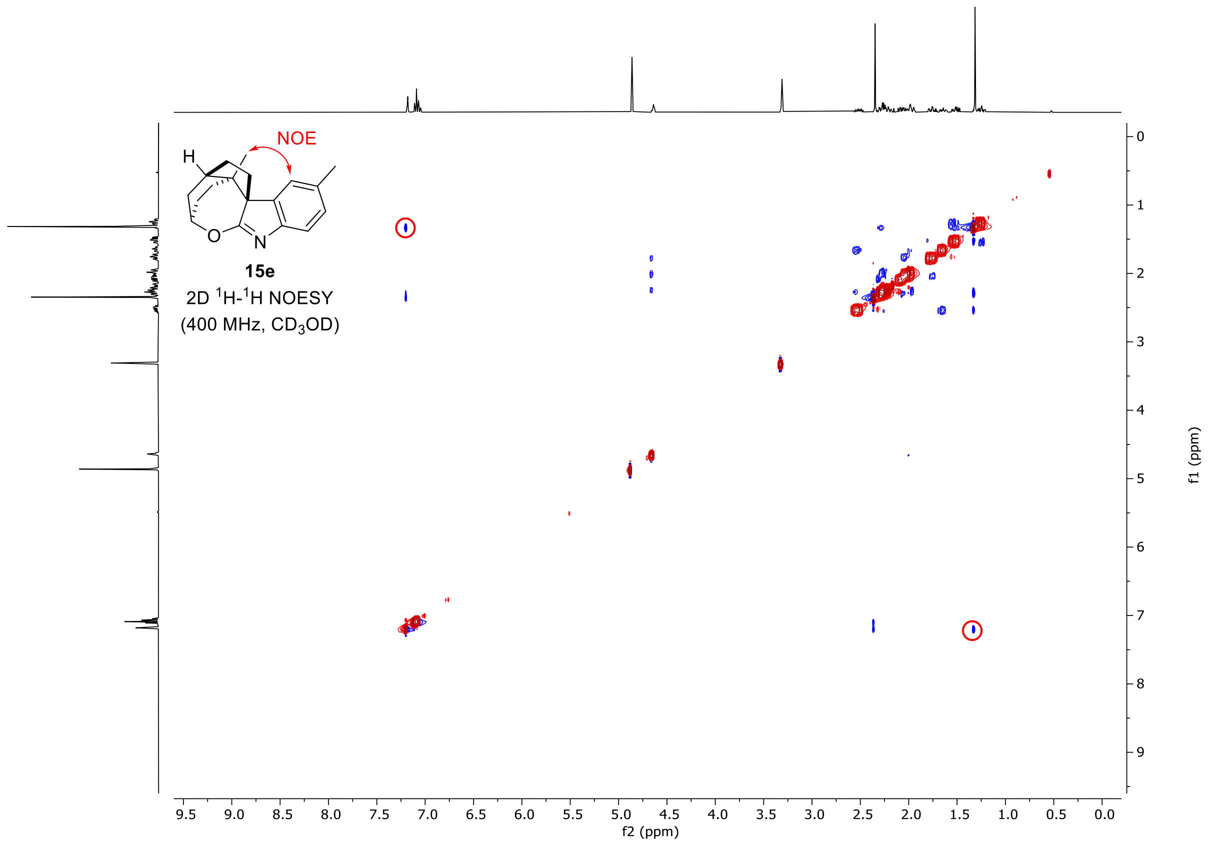
NOE
H
O
N
15e
2D 1H-1H NOESY
(400 MHz, CD3OD)
f2 (ppm)
f1 (ppm)

(3*R,3a*S**,6*R**,12b*R**)-11-Methoxy-3a-methyl-2,3,3a,4,5,6-hexahydro-1*H*-3,6-methanocyclopenta[3,4]oxepino[2,3-*b*]indole (15f)**

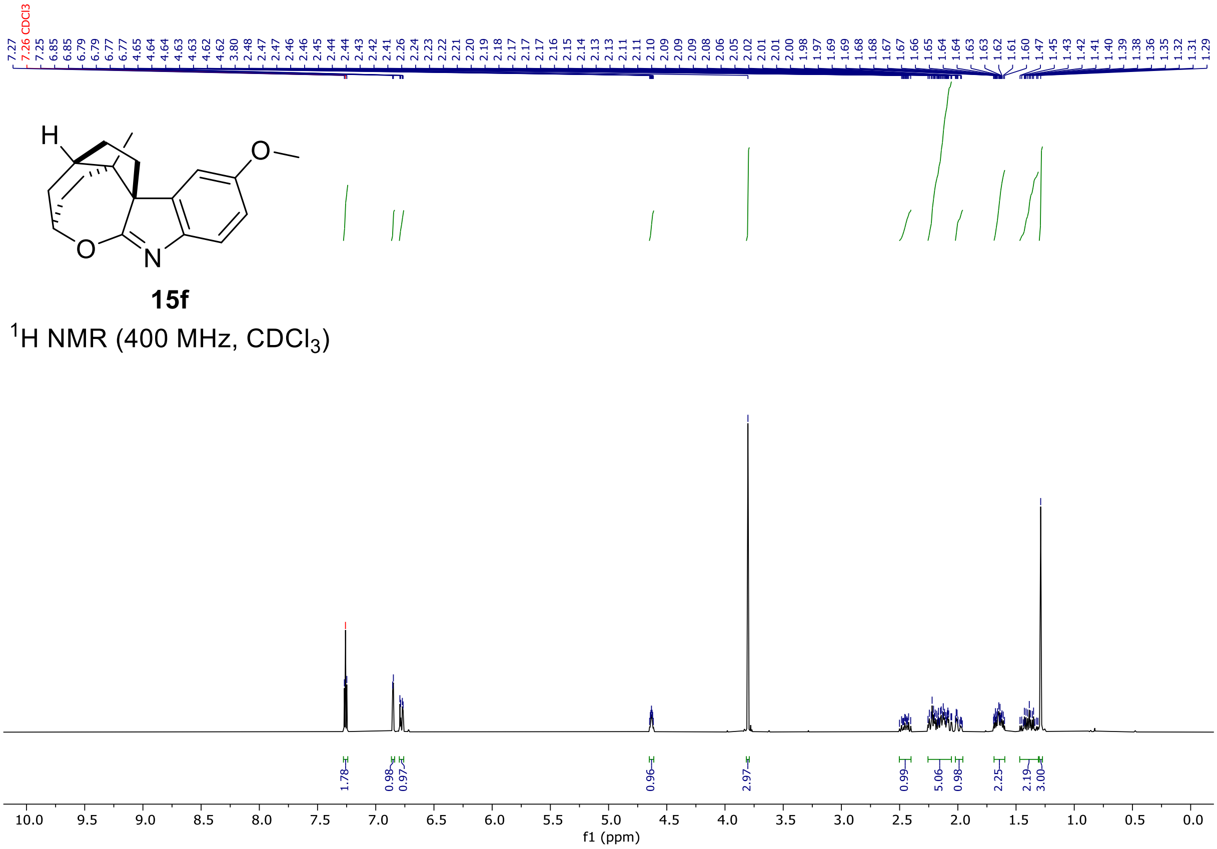

15f

^{1}H NMR (400 MHz, $CDCl_3$)

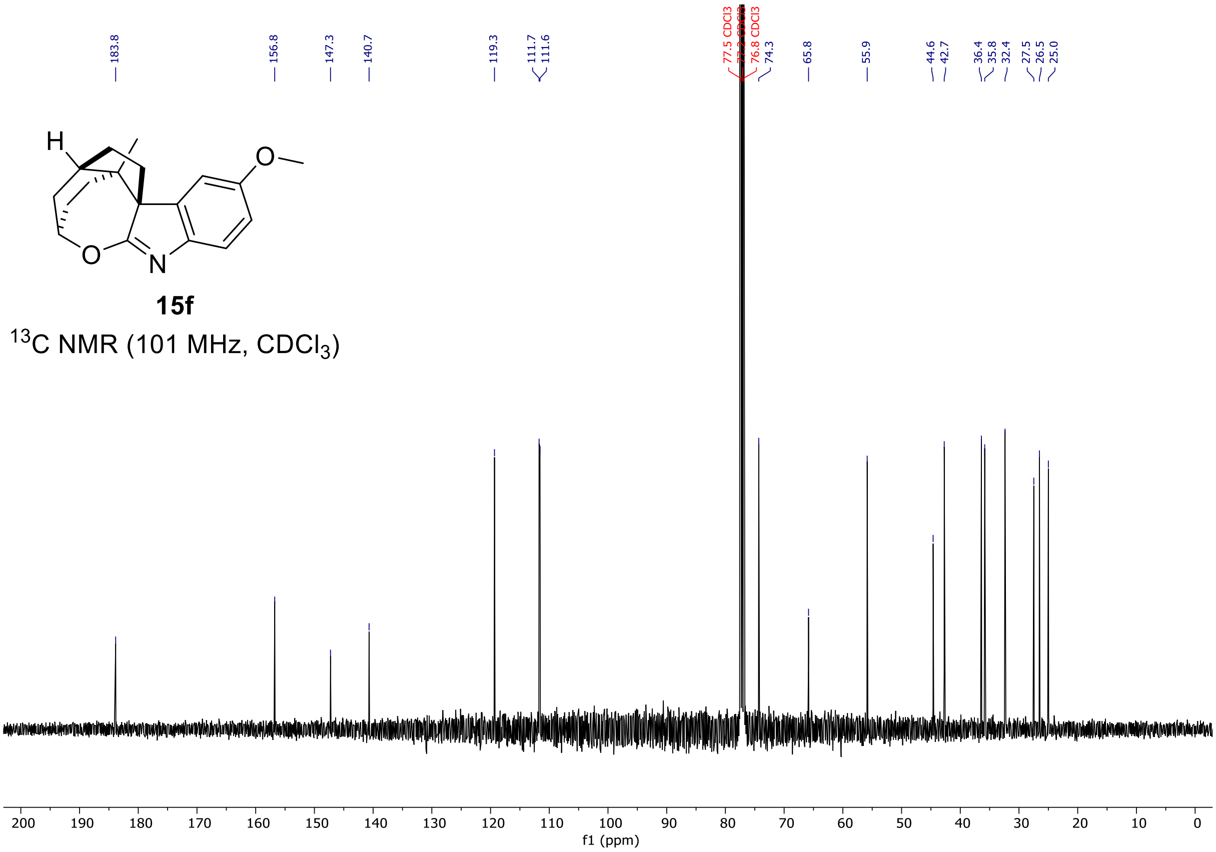

15f

^{13}C NMR (101 MHz, $CDCl_3$)

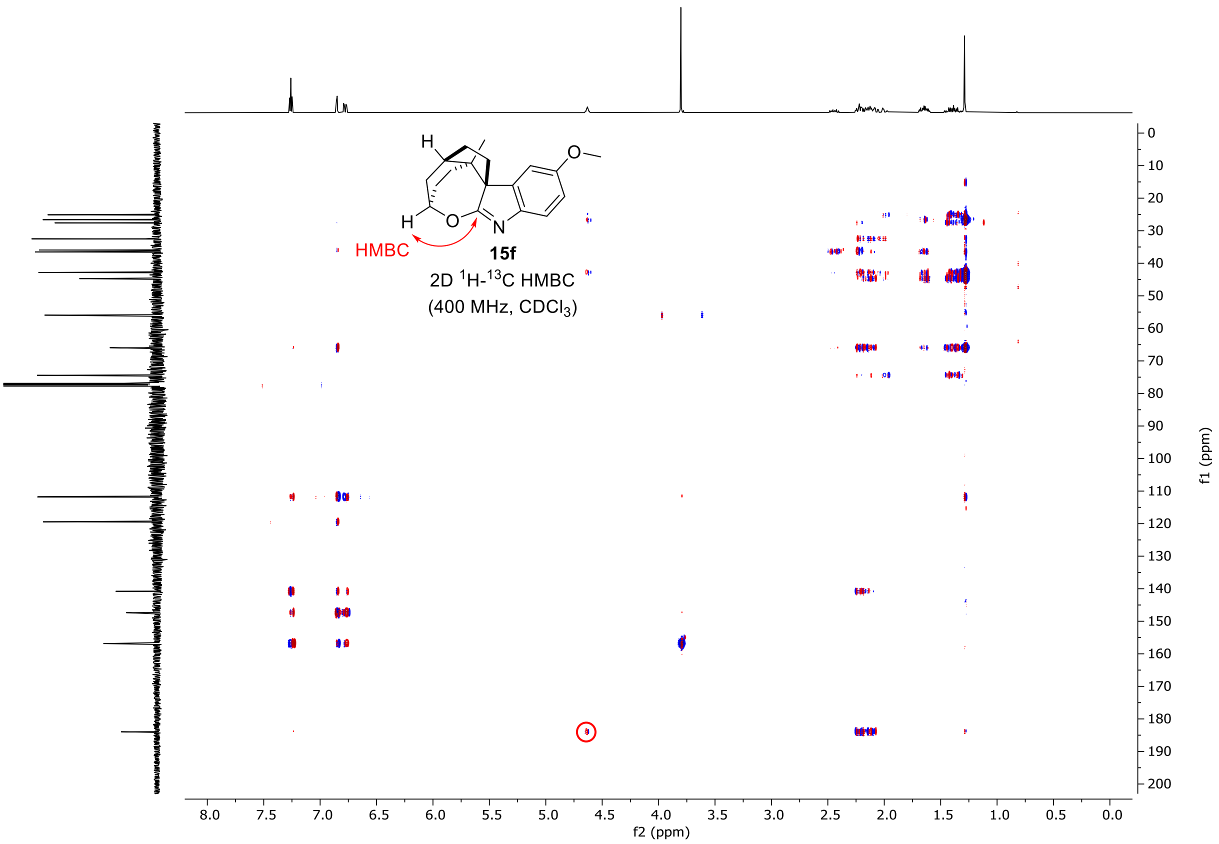
HMBC
15f
2D ¹H-¹³C HMBC
(400 MHz, CDCl3)
f2 (ppm)
f1 (ppm)

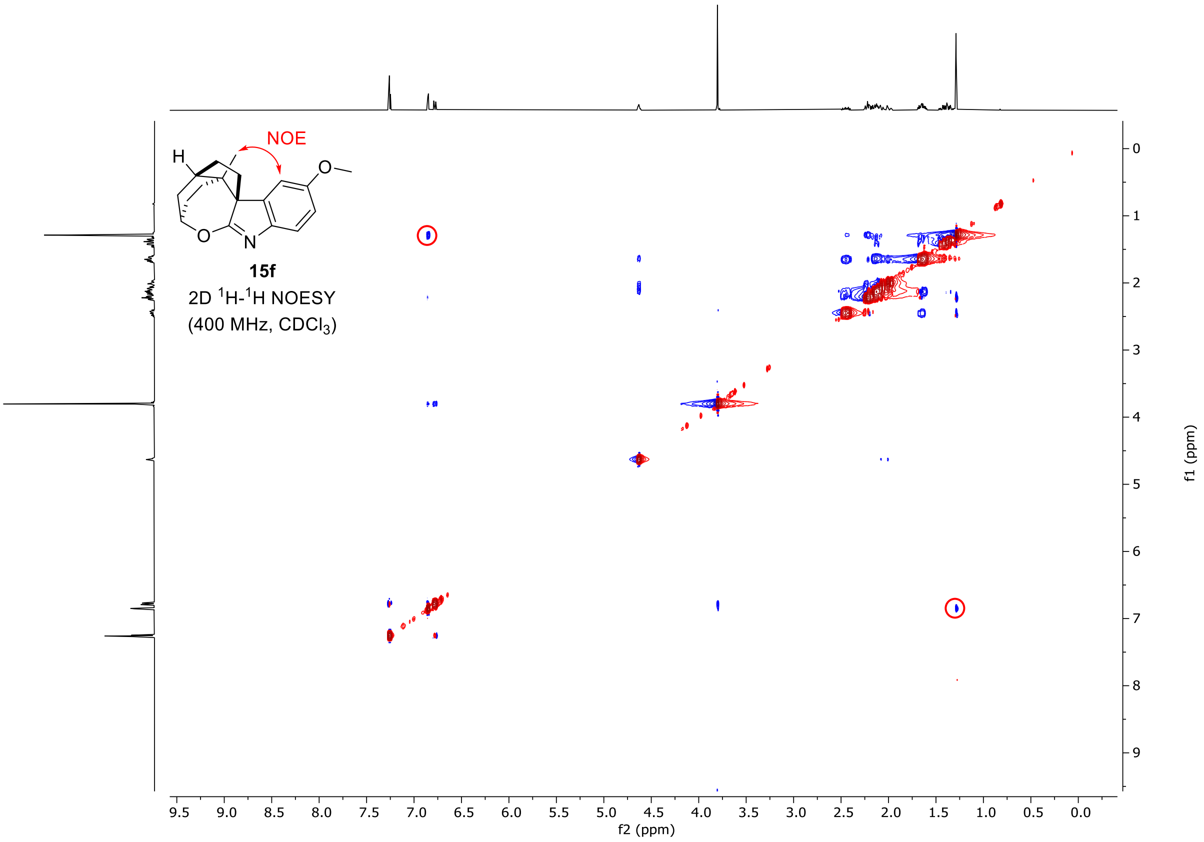
NOE
15f
2D ¹H-¹H NOESY
(400 MHz, CDCl3)
f2 (ppm)
f1 (ppm)

(3*R,3a*S**,6*R**,12b*R**)-3a-Methyl-11-(trifluoromethyl)-2,3,3a,4,5,6-hexahydro-1*H*-3,6-methanocyclopenta[3,4]oxepino[2,3-*b*]indole (15g)**

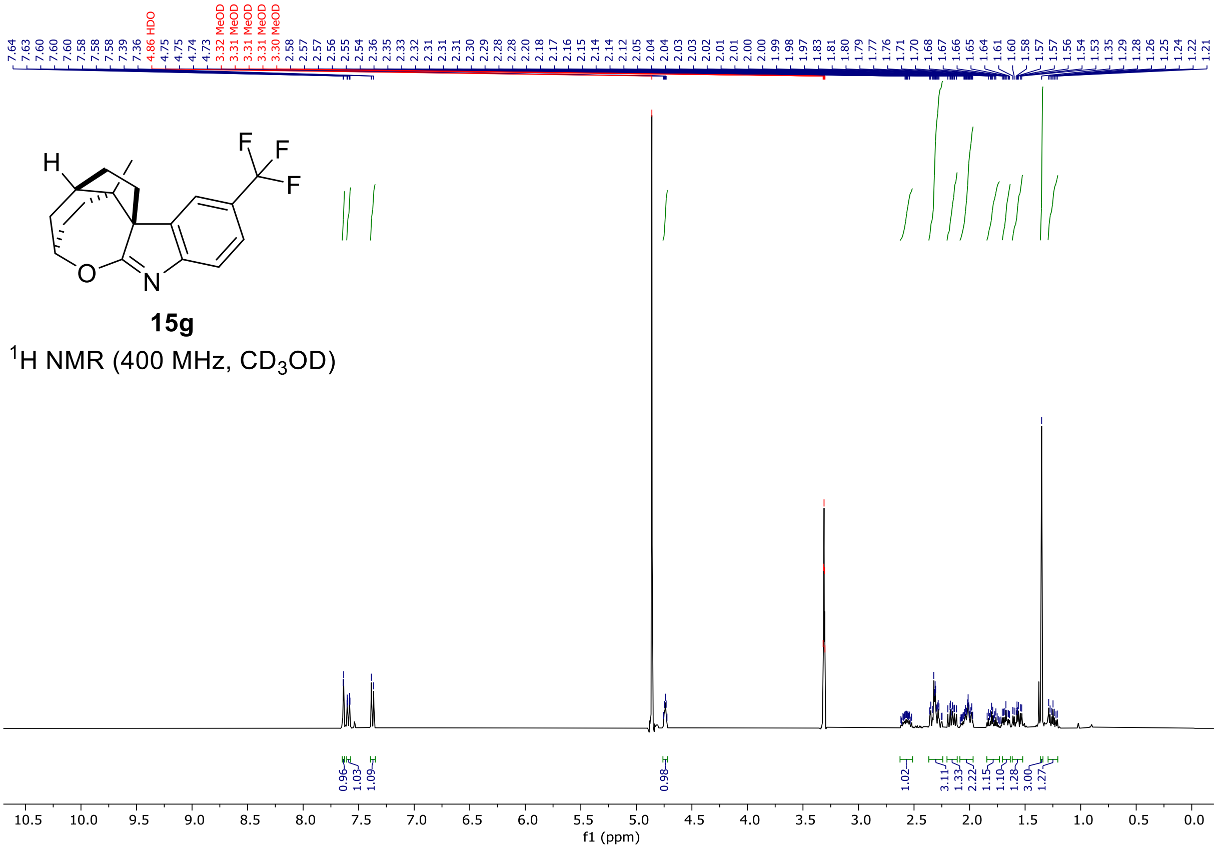

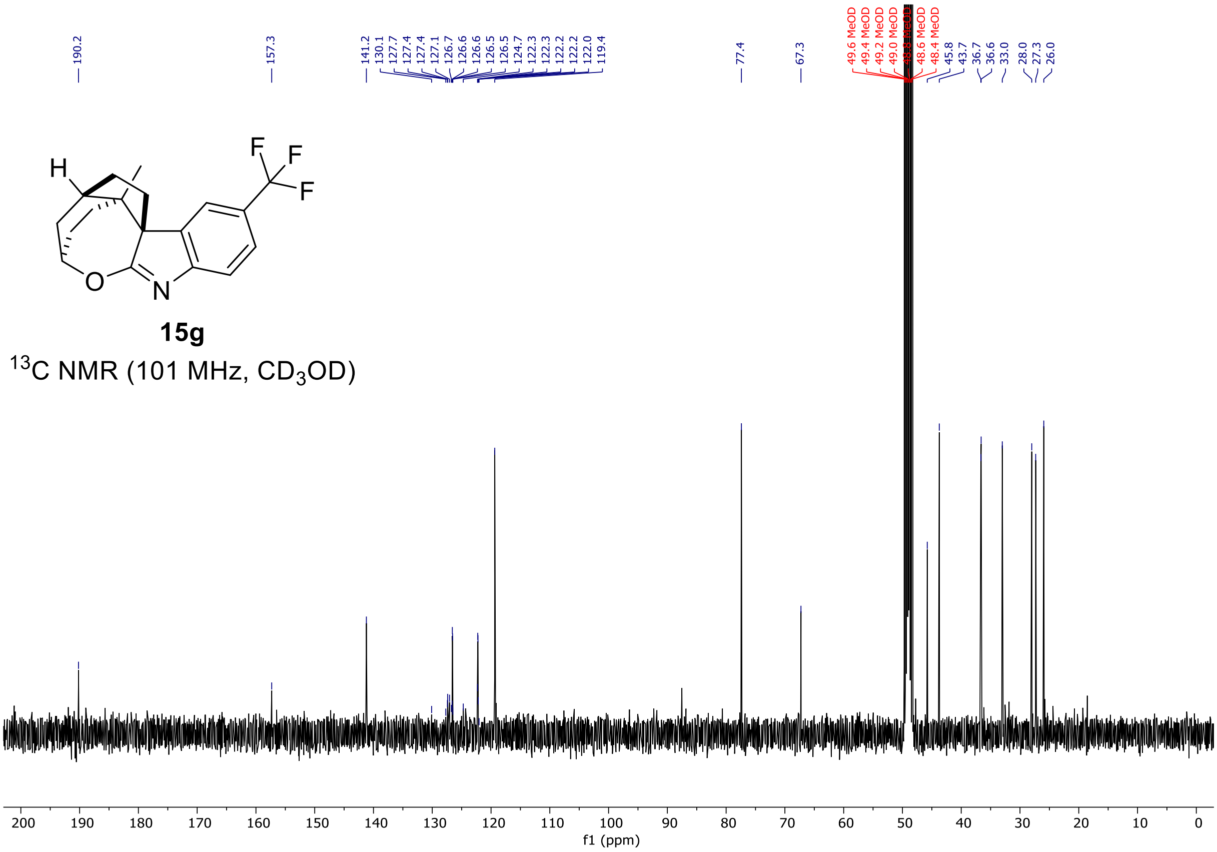

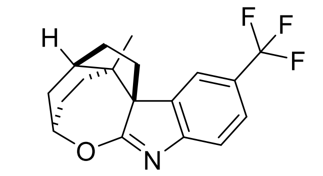

15g

^{19}F NMR (377 MHz, CD_3OD)

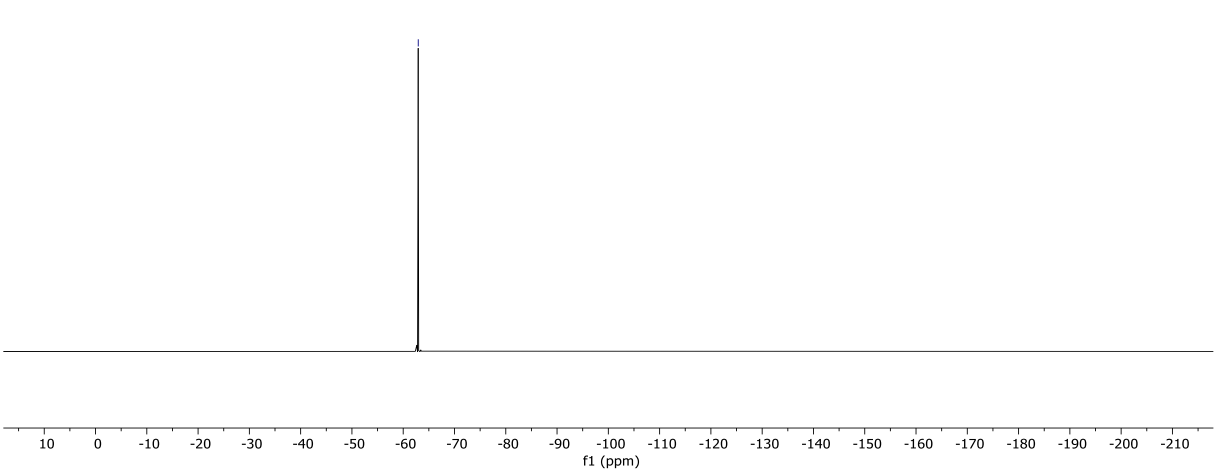

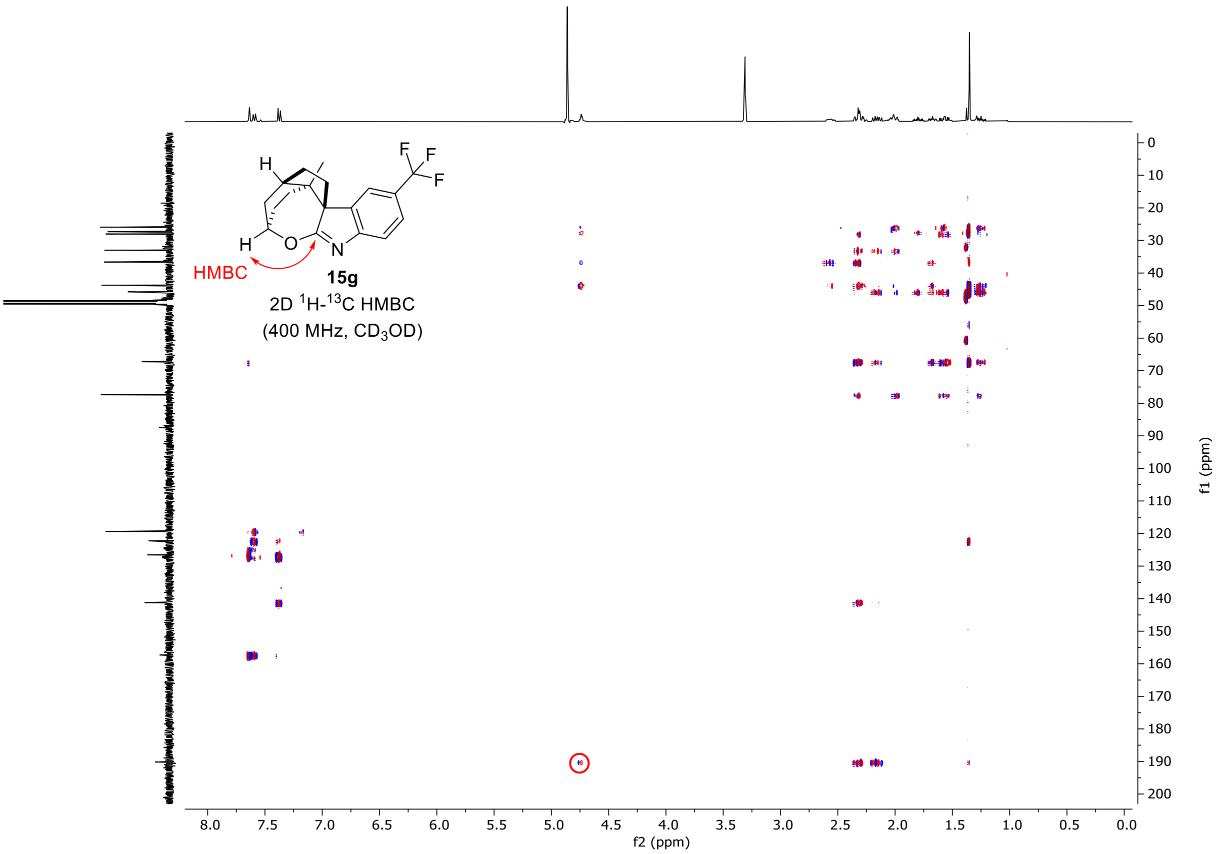

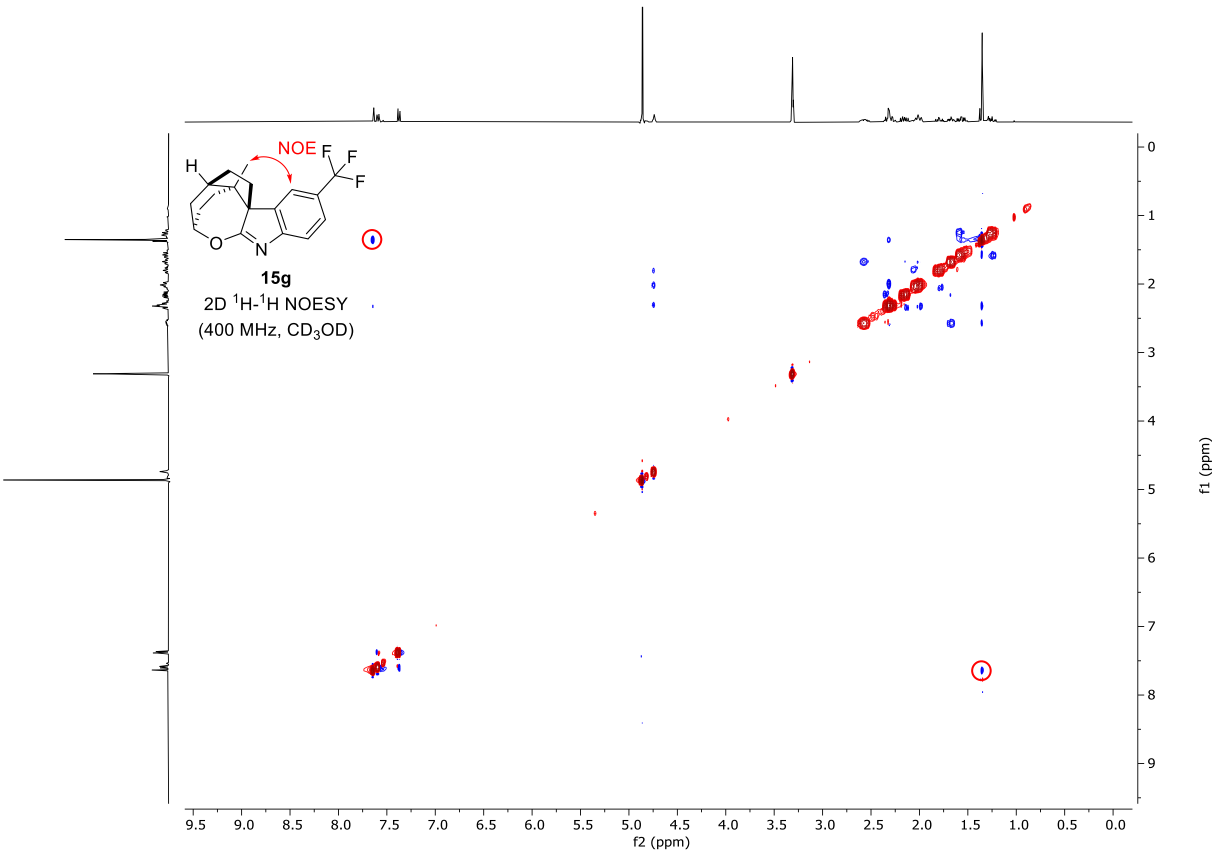
NOE
15g
2D 1H-1H NOESY
(400 MHz, CD3OD)
f2 (ppm)
f1 (ppm)

(3*R**,3a*S**,6*R**,12b*R**)-9-Bromo-3a-methyl-11-(trifluoromethyl)-2,3,3a,4,5,6-hexahydro-1*H*-3,6-methanocyclopenta[3,4]oxepino[2,3-*b*]indole (15h)

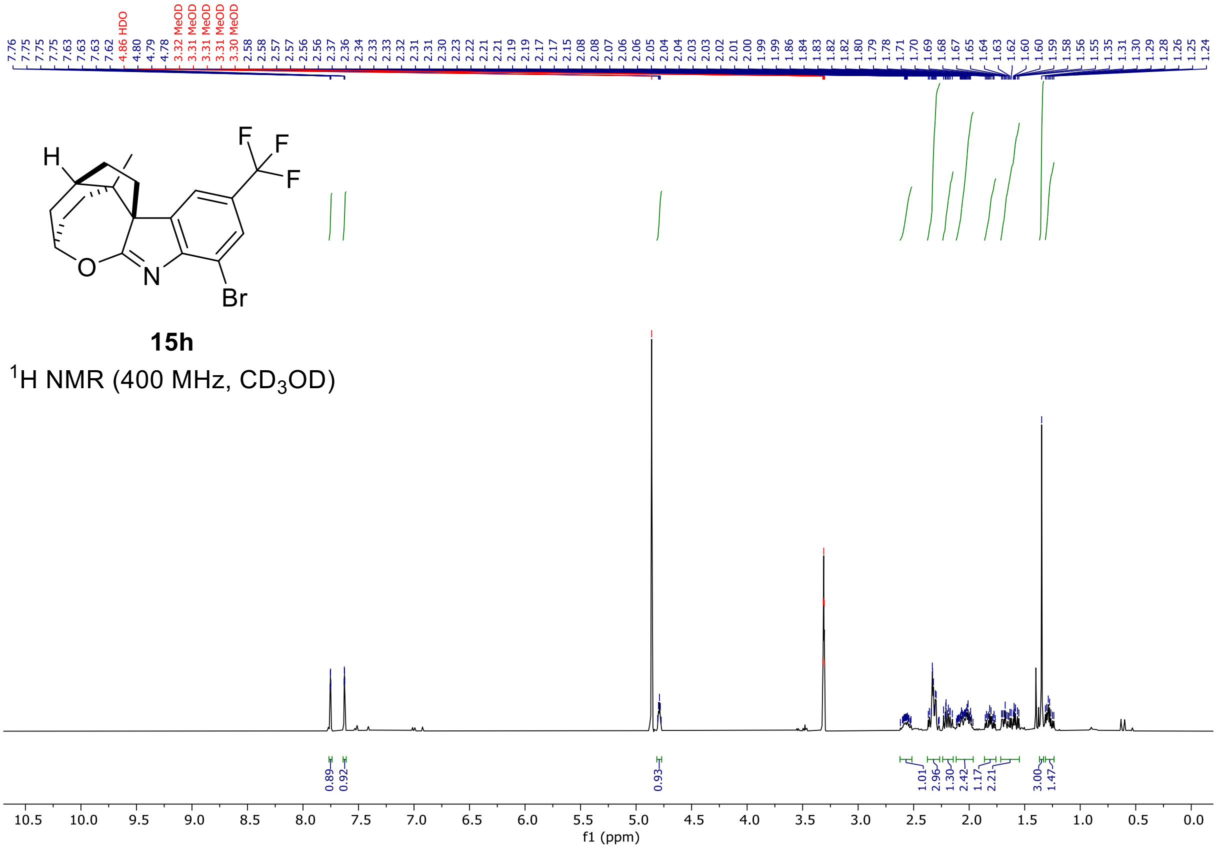

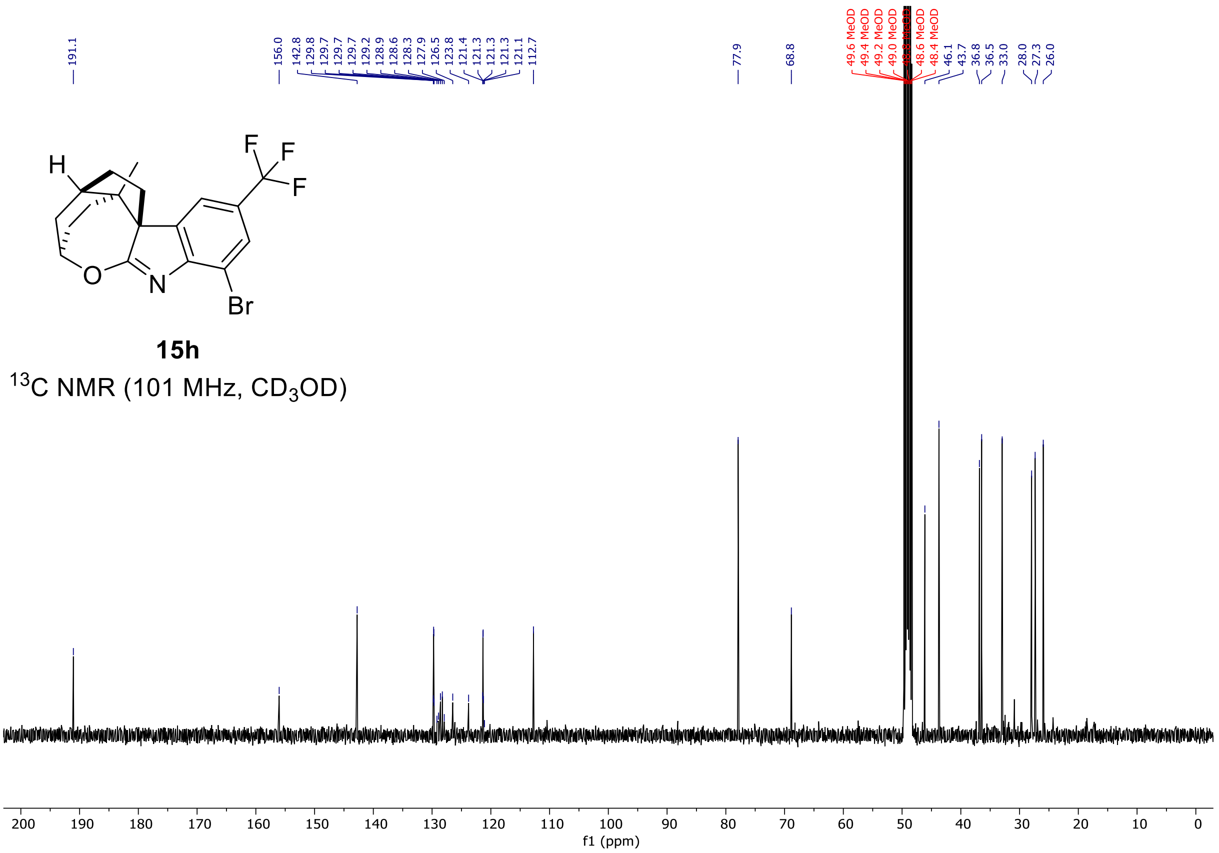

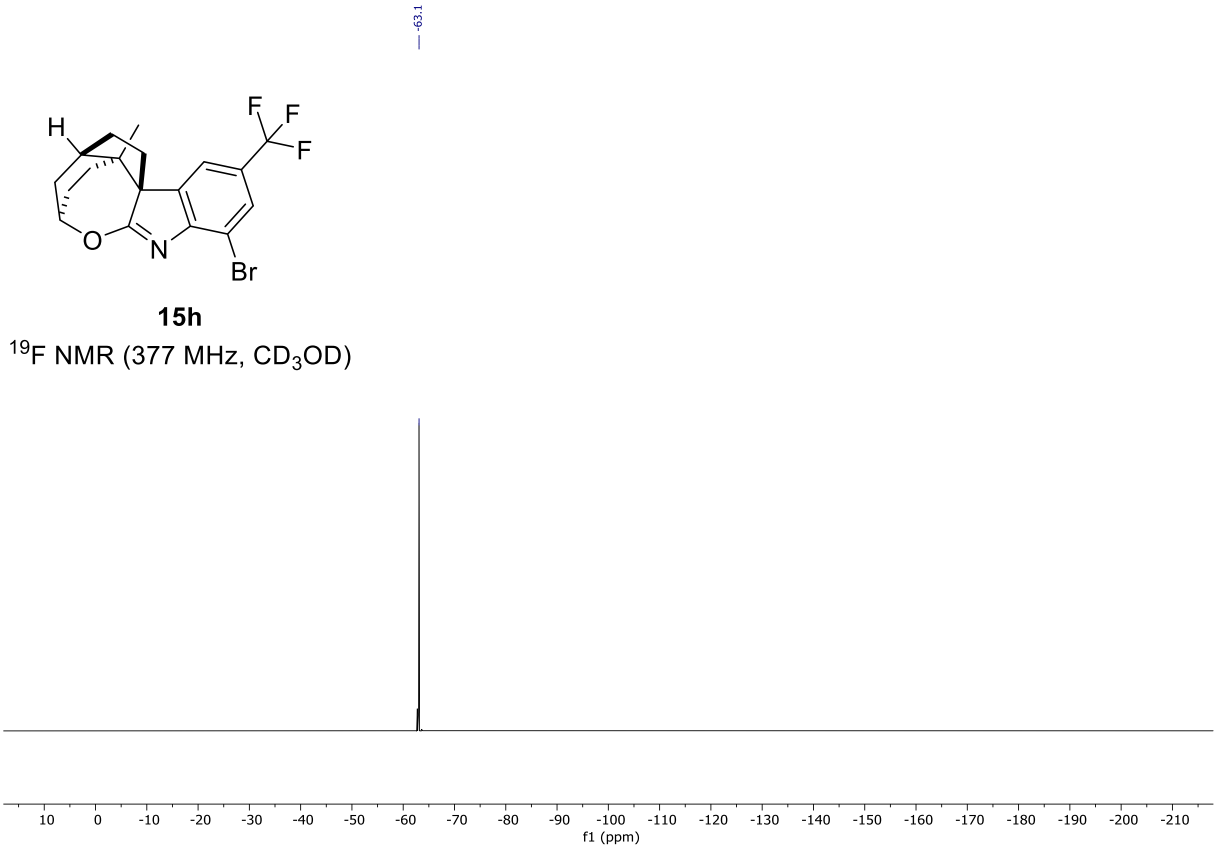
-63.1
15h
^{19}F NMR (377 MHz, CD$_3$OD)
f1 (ppm)

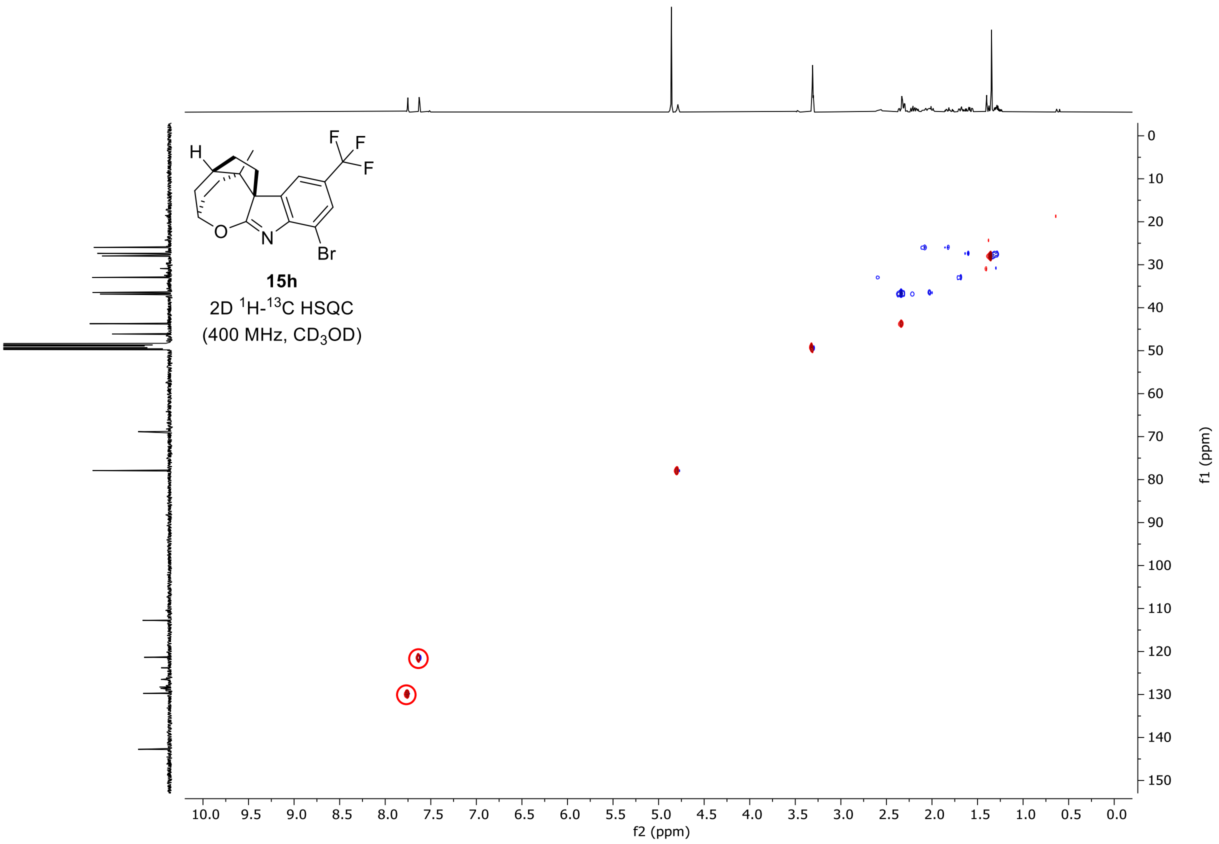
15h
2D ^{1}H-^{13}C HSQC
(400 MHz, CD$_3$OD)
f2 (ppm)
f1 (ppm)

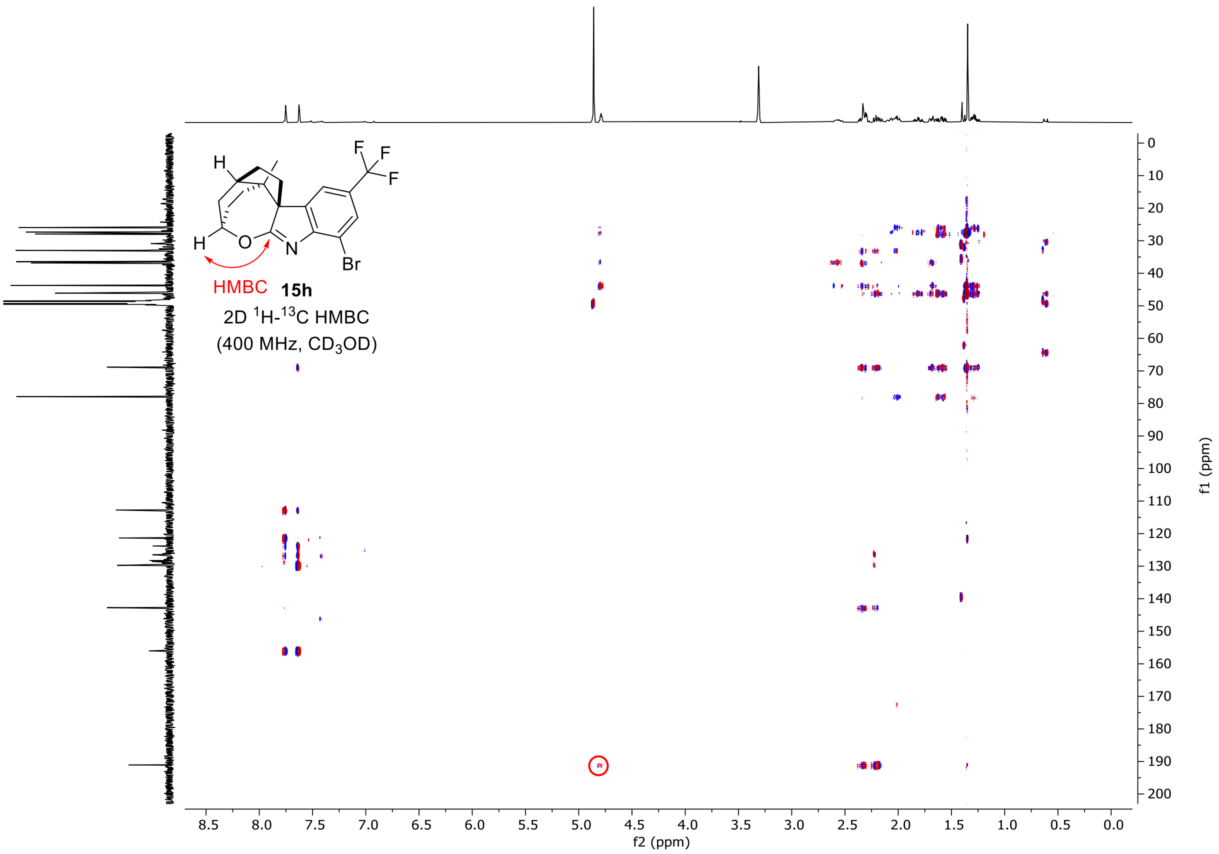
H
H
O
N
Br
F
F
F
HMBC
15h
2D ¹H-¹³C HMBC
(400 MHz, CD3OD)
f1 (ppm)
f2 (ppm)

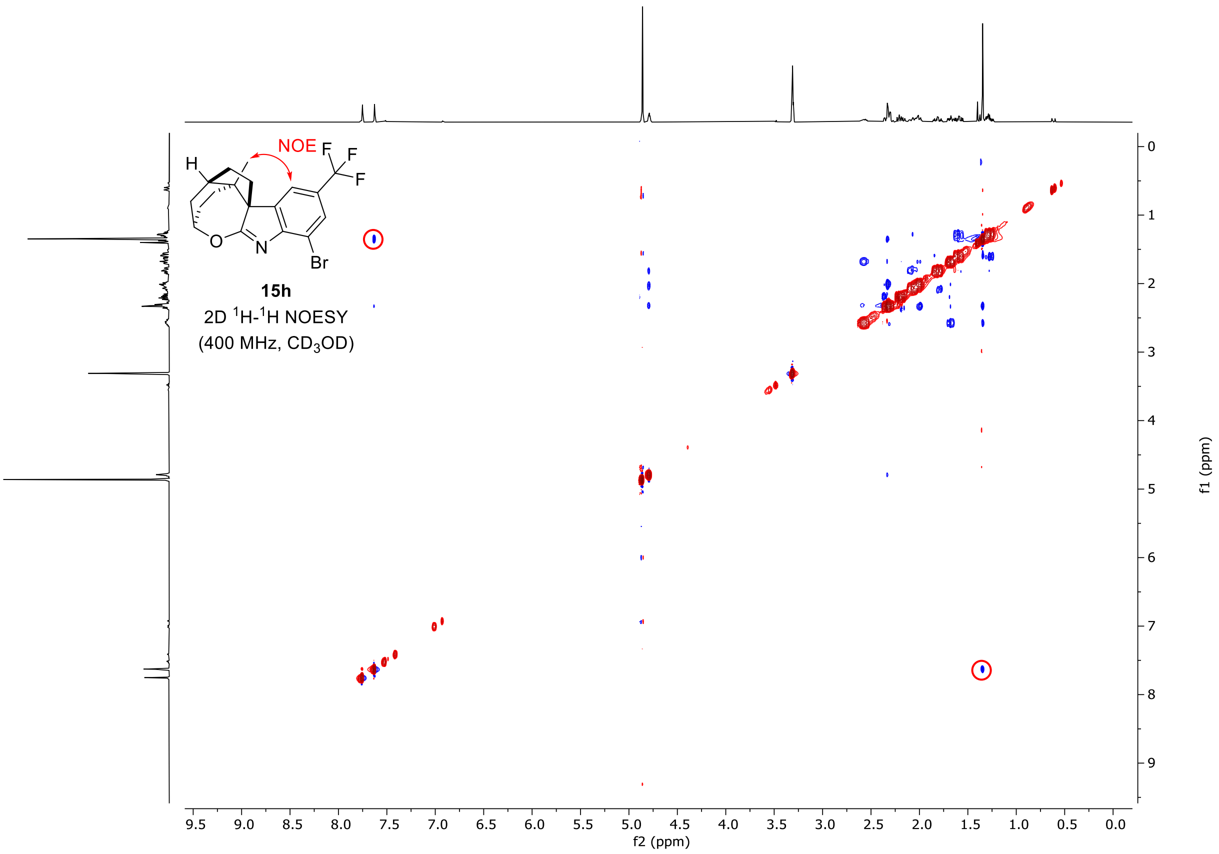
H
NOE
F
F
F
O
N
Br
15h
2D ¹H-¹H NOESY
(400 MHz, CD3OD)
f1 (ppm)
f2 (ppm)

(3*R,3a*S**,6*R**,12b*R**)-11-Chloro-9-fluoro-3a-methyl-2,3,3a,4,5,6-hexahydro-1*H*-3,6-methanocyclopenta[3,4]oxepino[2,3-*b*]indole (15i)**

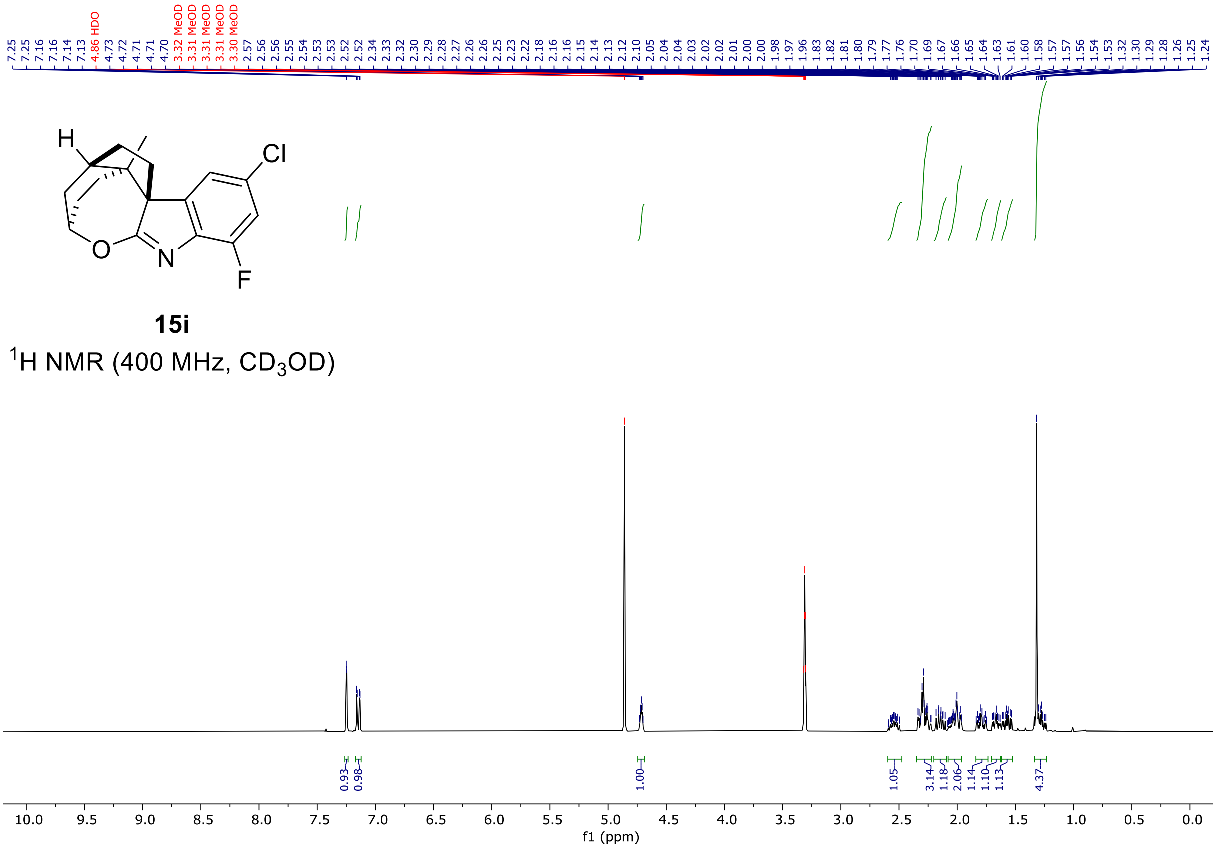

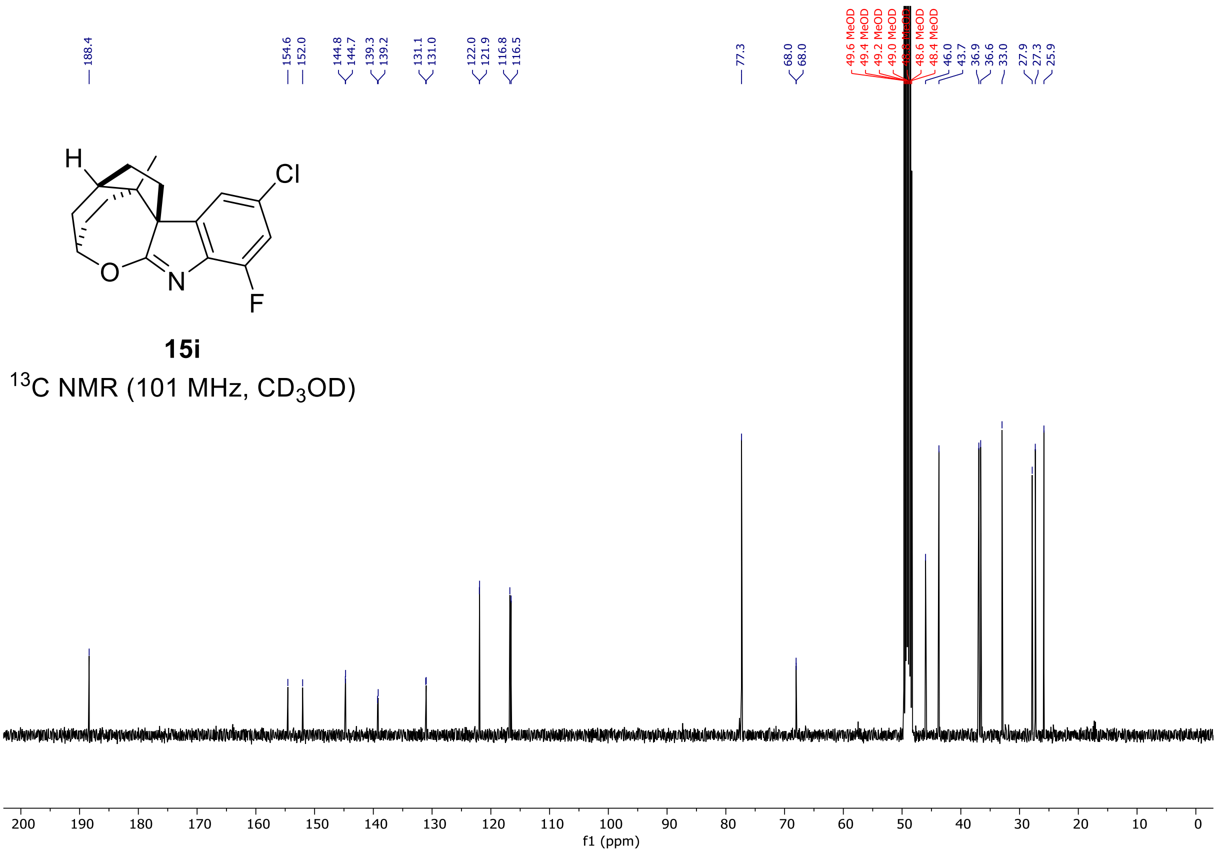

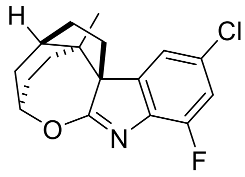

15i

^{19}F NMR (377 MHz, CD_3OD)

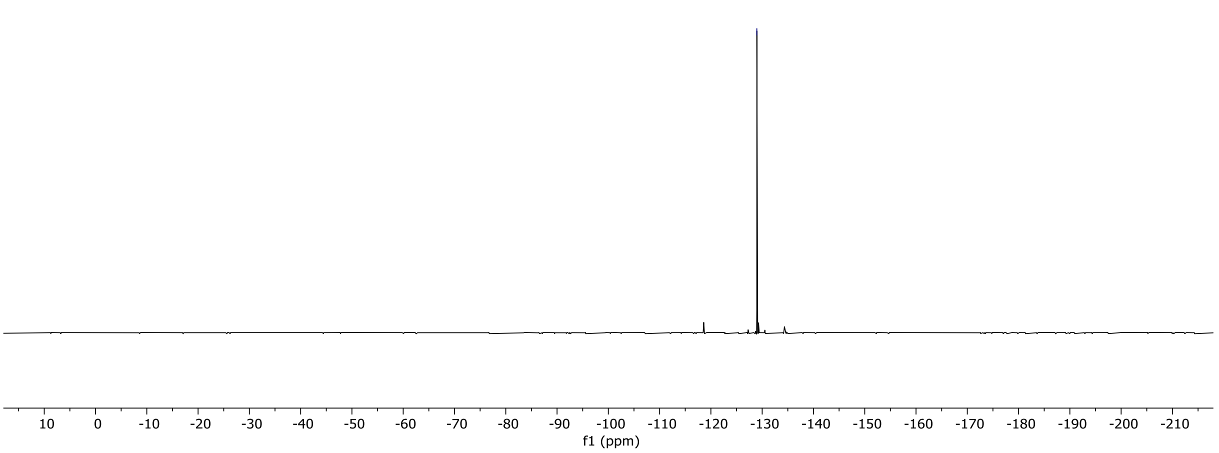

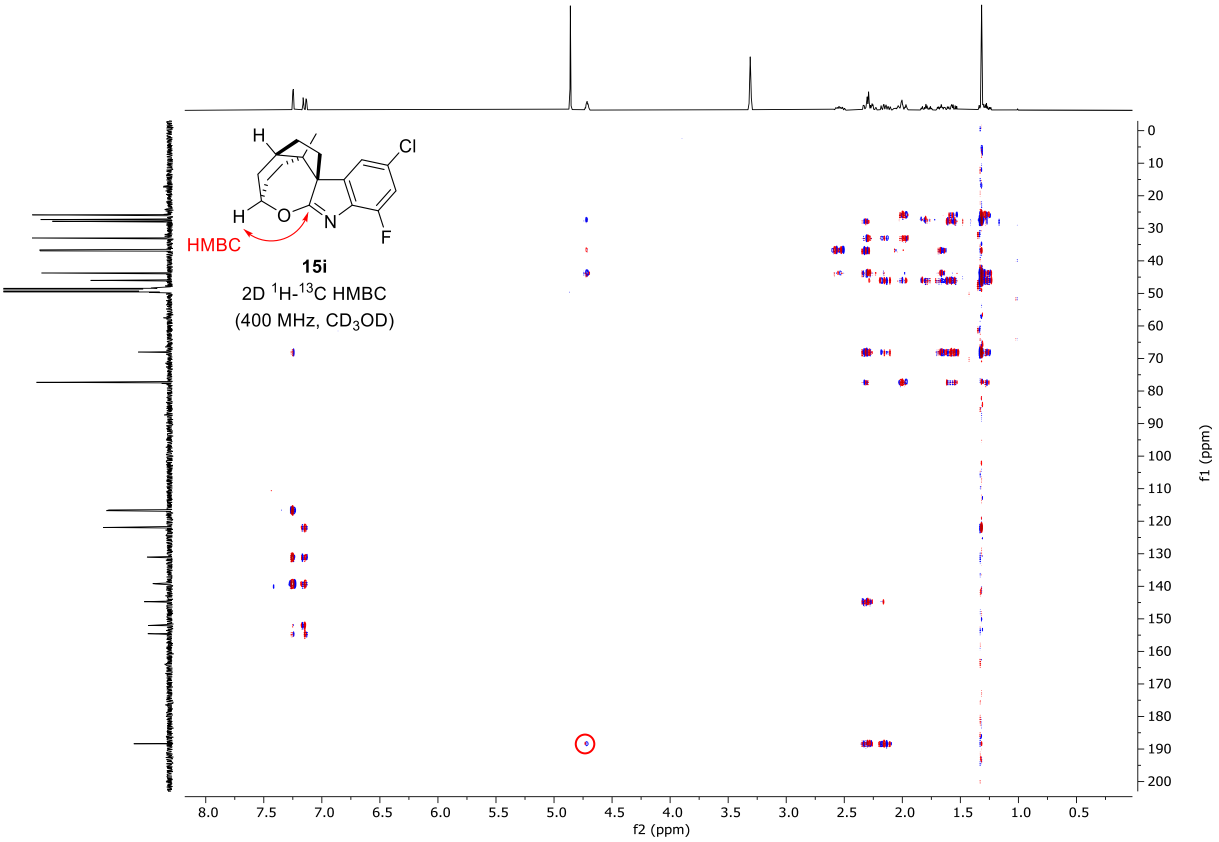

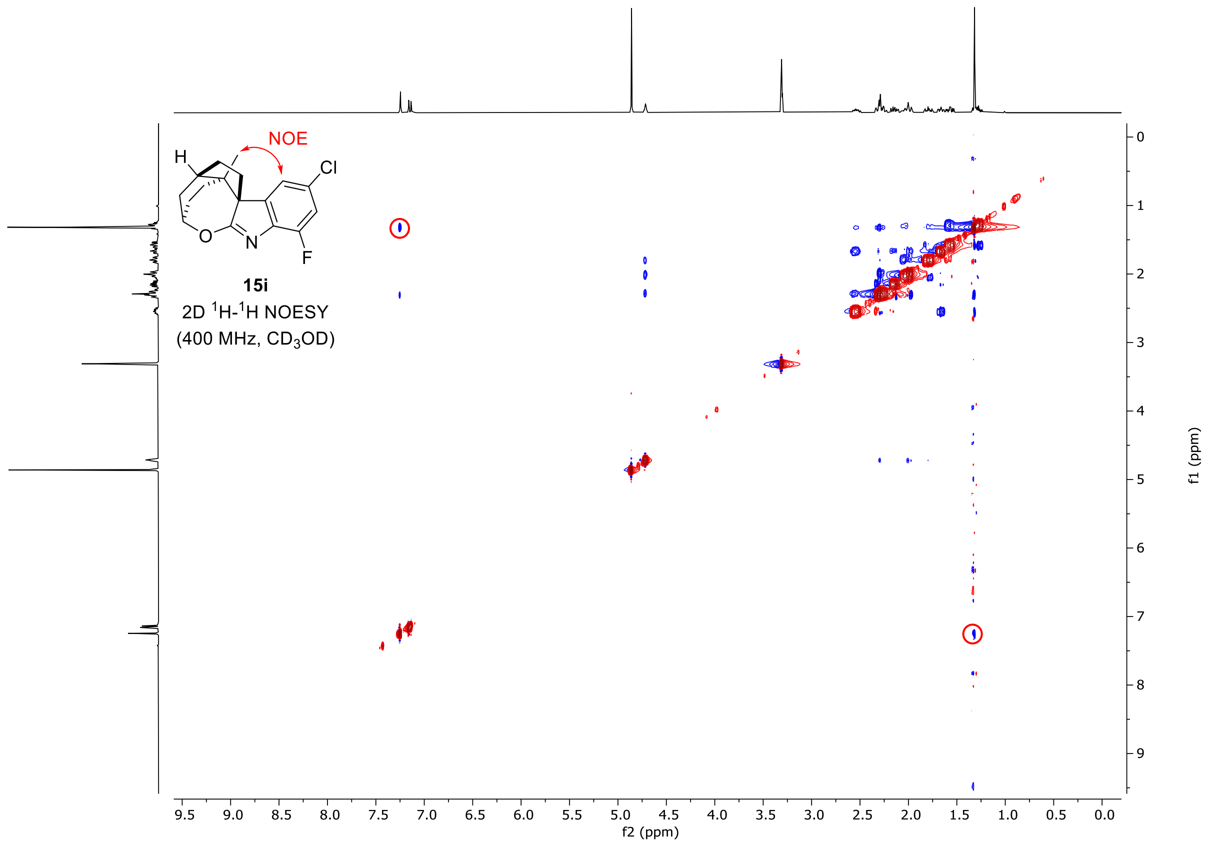
NOE
H
Cl
O
N
F
15i
2D 1H-1H NOESY
(400 MHz, CD3OD)
f2 (ppm)
f1 (ppm)

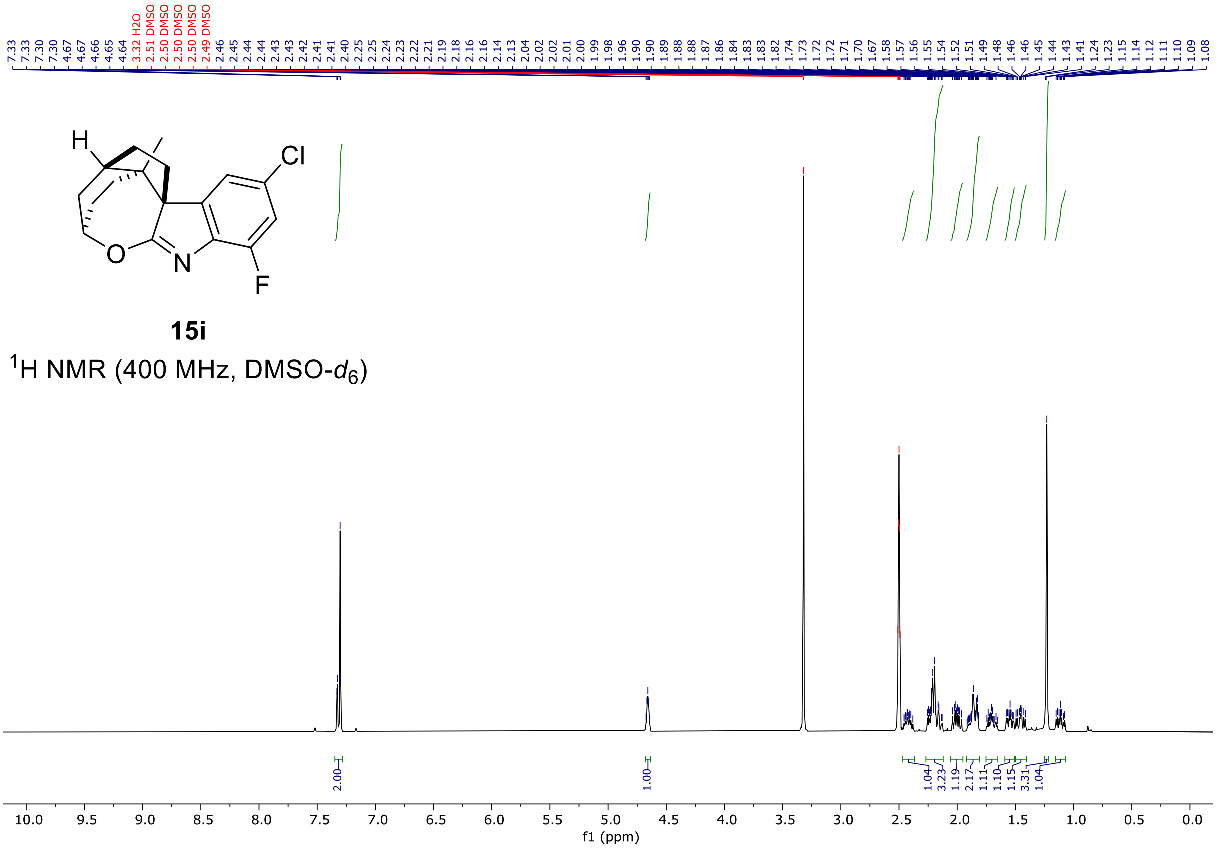
7.33 7.33 7.30 7.30 4.67 4.66 4.65 4.64 3.32 H2O 2.51 DMSO 2.50 DMSO 2.50 DMSO 2.50 DMSO 2.49 DMSO 2.46 2.45 2.44 2.44 2.43 2.43 2.42 2.41 2.41 2.40 2.25 2.25 2.24 2.23 2.22 2.21 2.19 2.18 2.16 2.16 2.14 2.13 2.04 2.02 2.02 2.01 2.00 1.99 1.98 1.96 1.90 1.90 1.89 1.88 1.88 1.87 1.86 1.84 1.83 1.83 1.82 1.74 1.73 1.72 1.72 1.71 1.70 1.67 1.58 1.57 1.56 1.55 1.54 1.52 1.51 1.49 1.48 1.46 1.46 1.45 1.44 1.43 1.41 1.24 1.23 1.15 1.14 1.12 1.11 1.10 1.09 1.08
H
Cl
O
N
F
15i
1H NMR (400 MHz, DMSO-d6)
2.00
1.00
1.04 3.23 1.19 2.17 1.11 1.10 1.15 3.31 1.04
f1 (ppm)

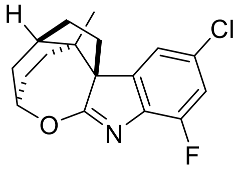

15i

^{13}C NMR (101 MHz, DMSO-d_6)

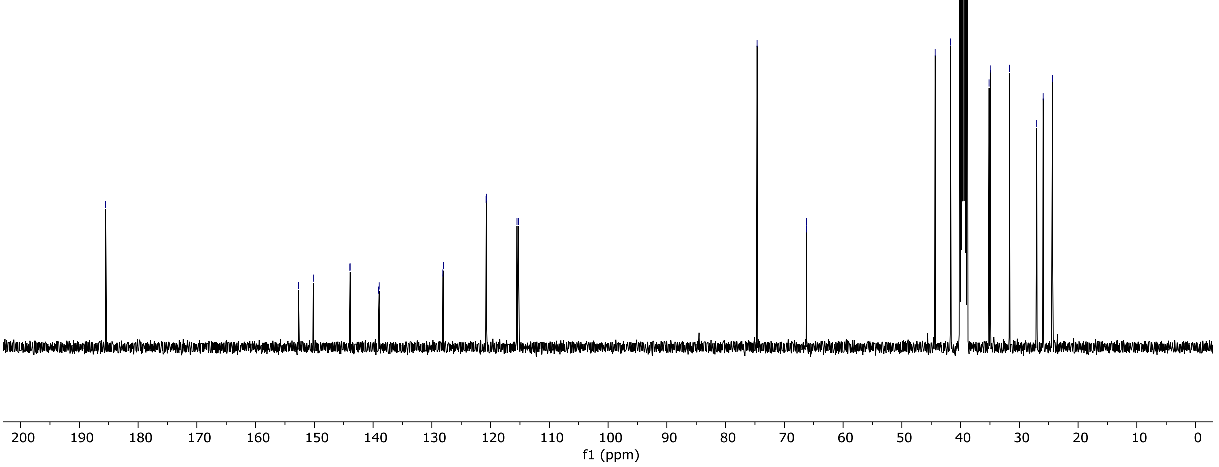

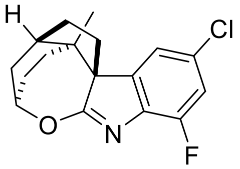

15i

^{19}F NMR (377 MHz, DMSO-d_6)

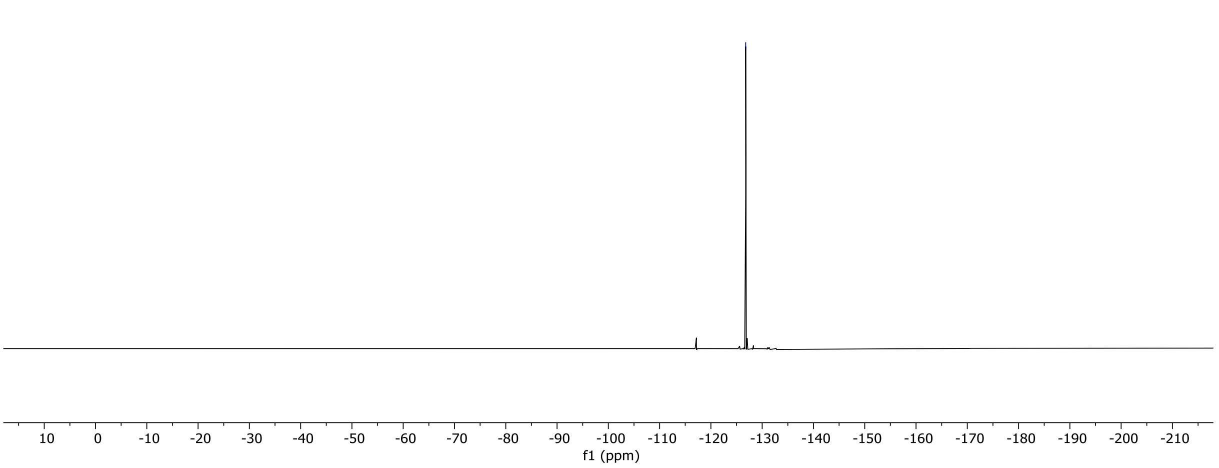

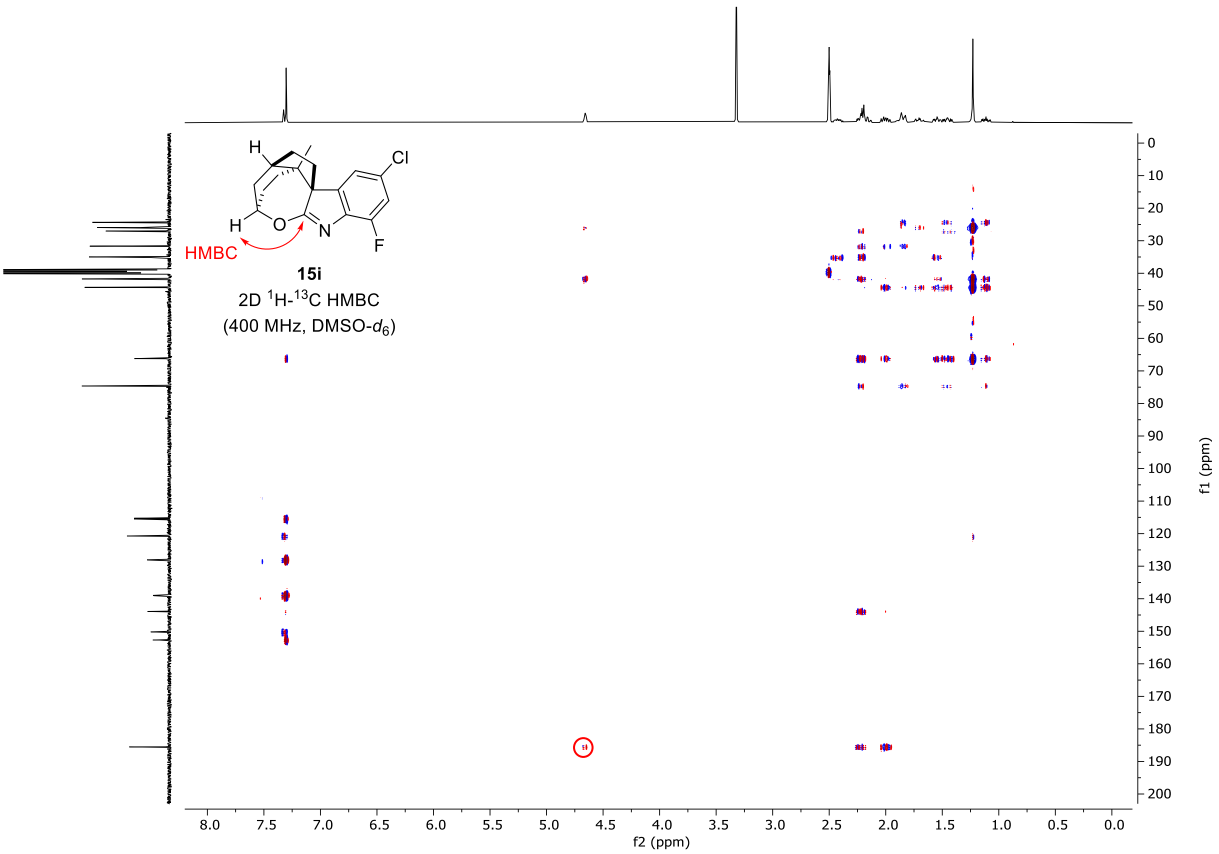
Cl
H
H
O
N
F
HMBC
15i
2D ¹H-¹³C HMBC
(400 MHz, DMSO-d6)
f2 (ppm)
f1 (ppm)

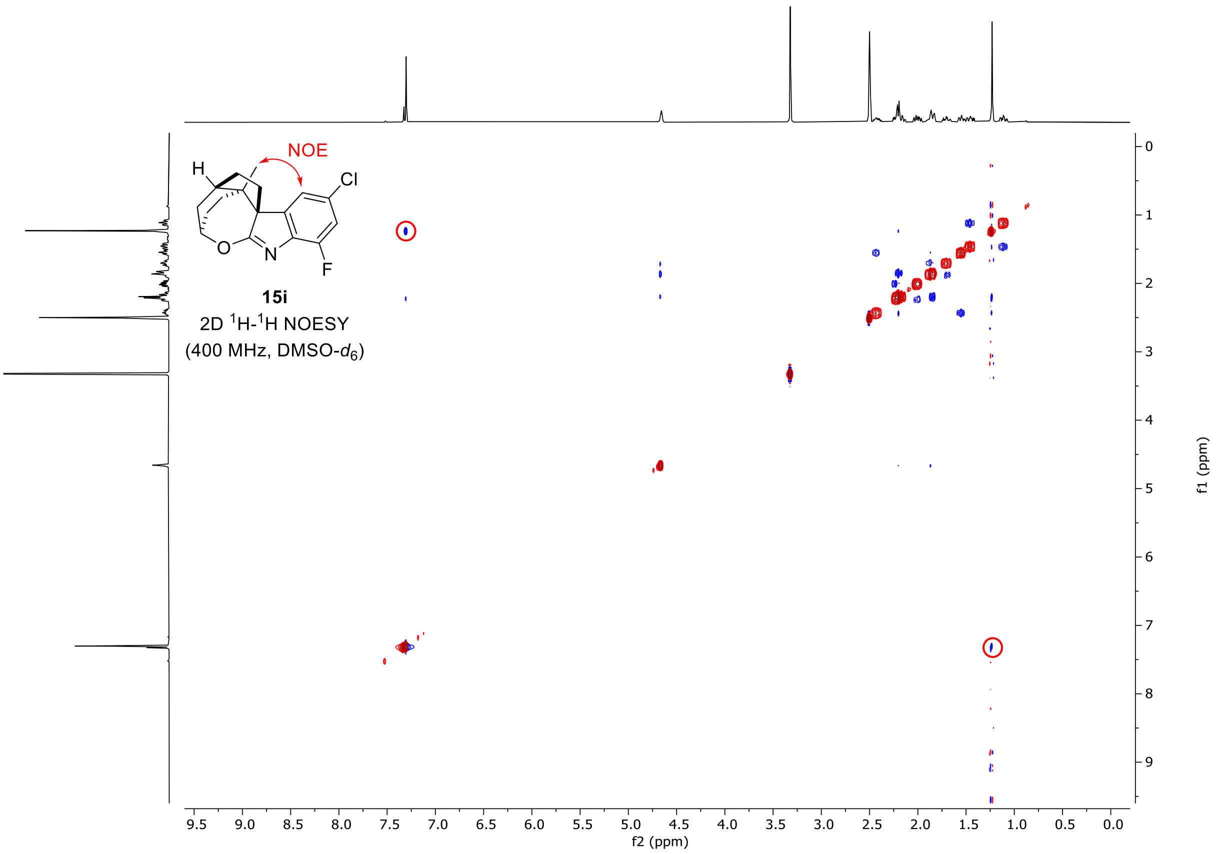
NOE
H
Cl
O
N
F
15i
2D ¹H-¹H NOESY
(400 MHz, DMSO-d6)
f2 (ppm)
f1 (ppm)

(3*R,3a*S**,6*R**,12b*R**)-3a-Methyl-11-morpholino-2,3,3a,4,5,6-hexahydro-1*H*-3,6-methanocyclopenta[3,4]oxepino[2,3-*b*]indole, (±)-asteroxin-1 (19a)**

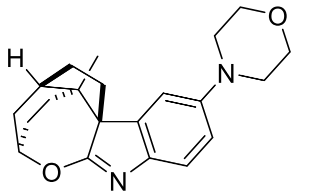

19a

^{1}H NMR (400 MHz, $CDCl_3$)

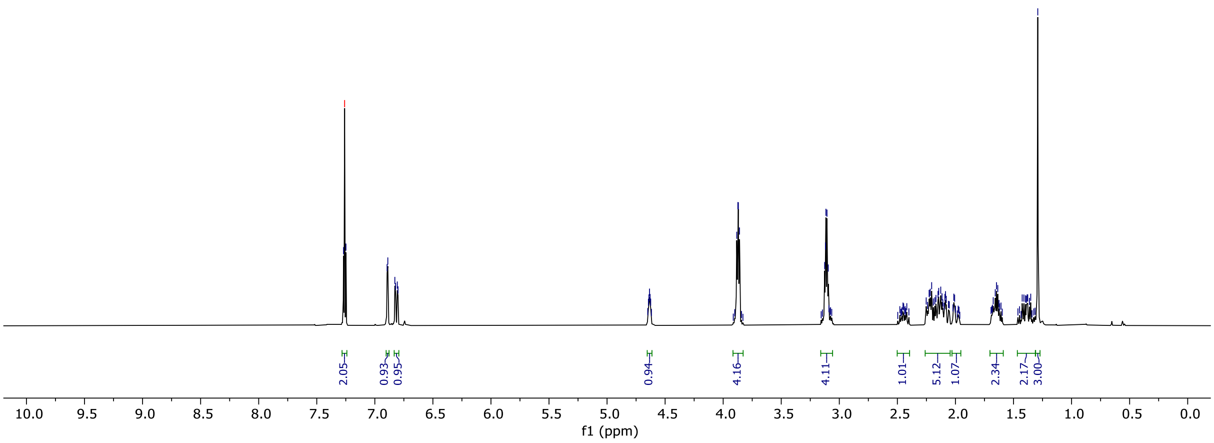

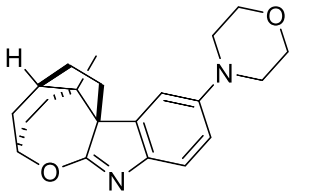

19a

^{13}C NMR (101 MHz, $CDCl_3$)

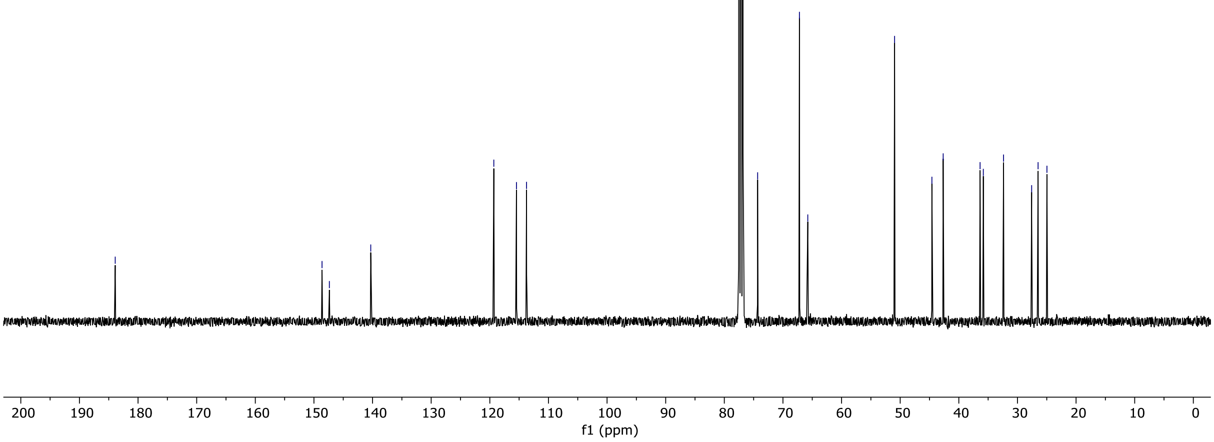

(3*R,3a*S**,6*R**,12b*R**)-*N*-Benzyl-3a-methyl-2,3,3a,4,5,6-hexahydro-1*H*-3,6-methanocyclopenta[3,4]oxepino[2,3-*b*]indol-11-amine (19b)**

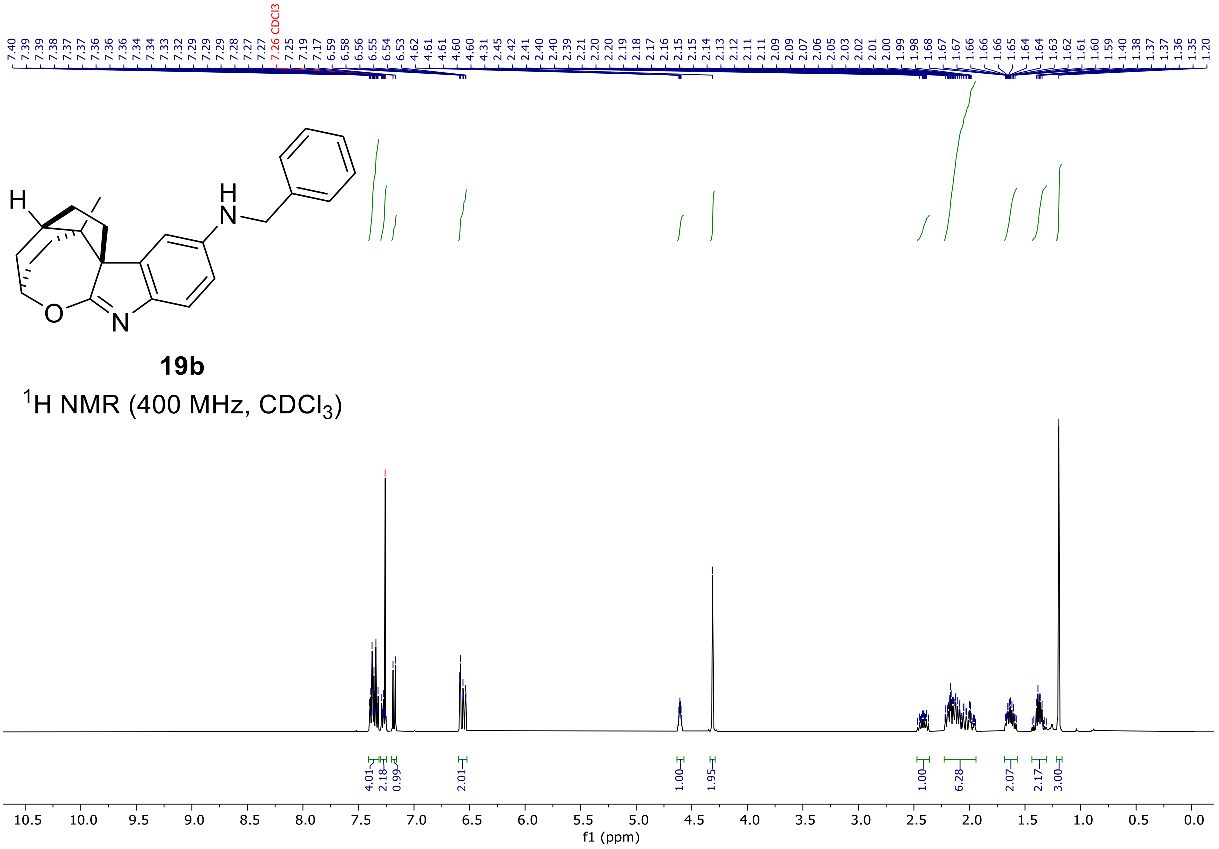

19b

^{1}H NMR (400 MHz, $CDCl_3$)

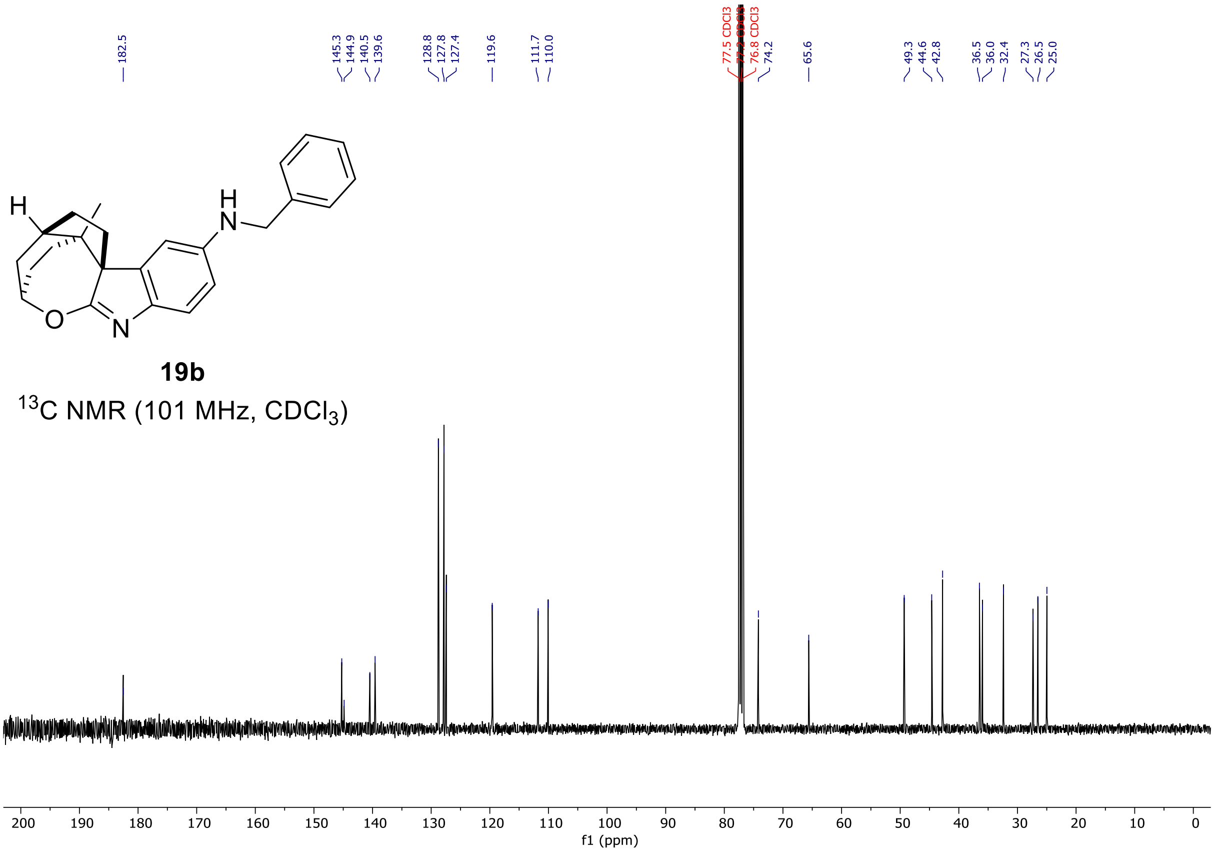

19b

^{13}C NMR (101 MHz, $CDCl_3$)

(3*R,3a*S**,6*R**,12b*R**)-*N*-Benzyl-3a-methyl-*N*-((3*R**,3a*S**,6*R**,12b*R**)-3a-methyl-2,3,3a,4,5,6-hexahydro-1*H*-3,6-methanocyclopenta[3,4]oxepino[2,3-*b*]indol-11-yl)-2,3,3a,4,5,6-hexahydro-1*H*-3,6-methanocyclopenta[3,4]oxepino[2,3-*b*]indol-11-amine (20a)**

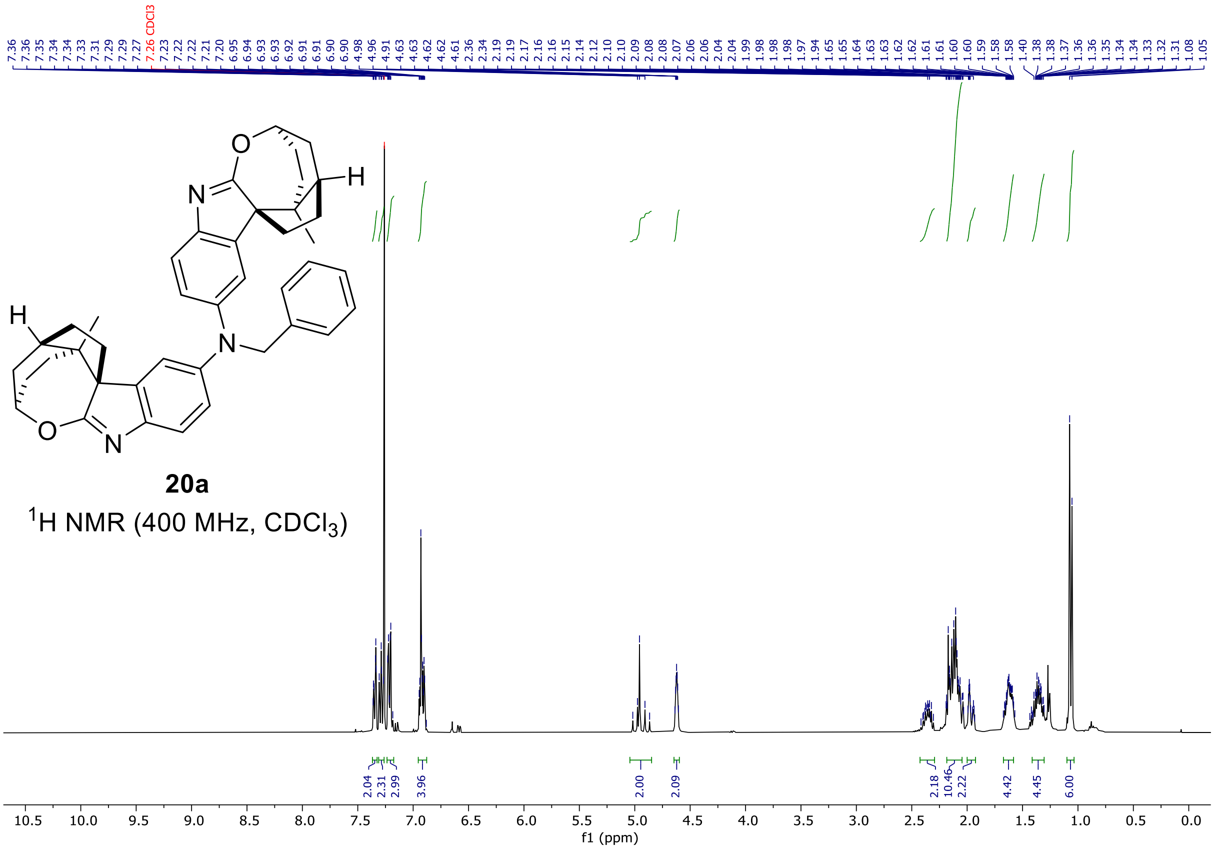

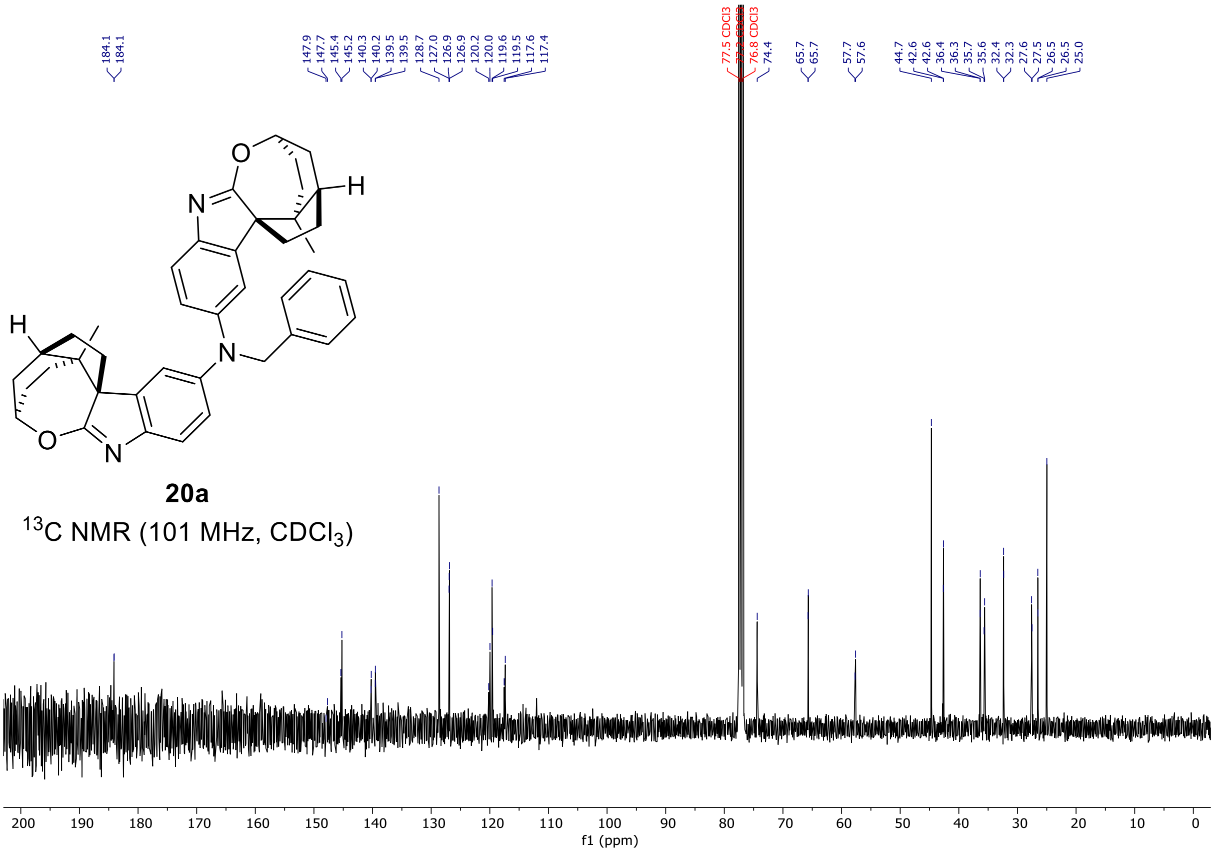

(3R*,3aS*,6R*,12bR*)-3a-Methyl-N-pentyl-2,3,3a,4,5,6-hexahydro-1H-3,6-methanocyclopenta[3,4]oxepino[2,3-b]indol-11-amine (19c)

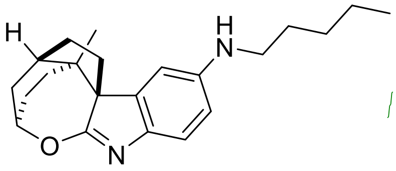

19c

^{1}H NMR (400 MHz, $CDCl_3$)

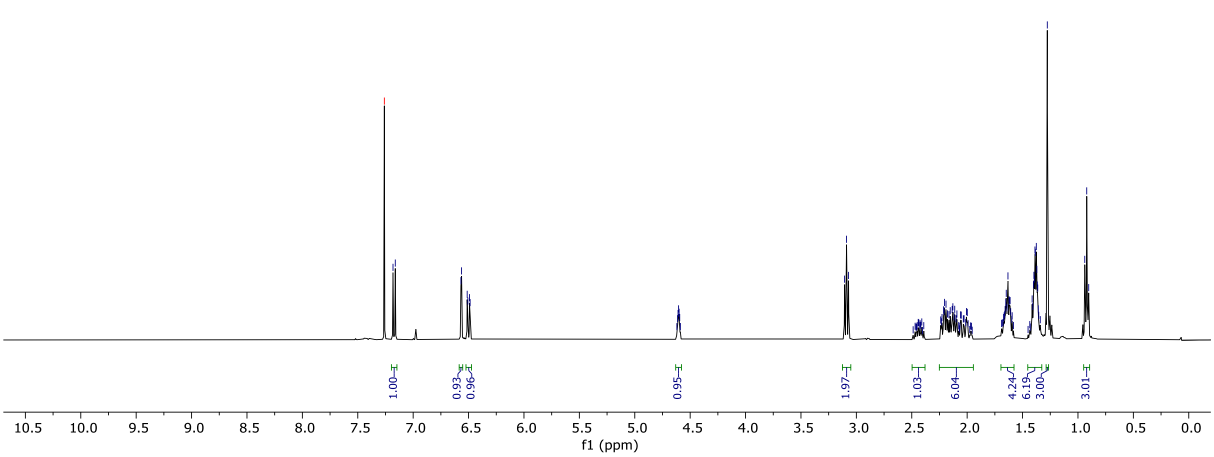

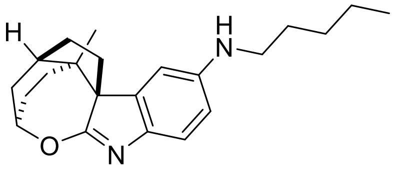

19c

^{13}C NMR (101 MHz, $CDCl_3$)

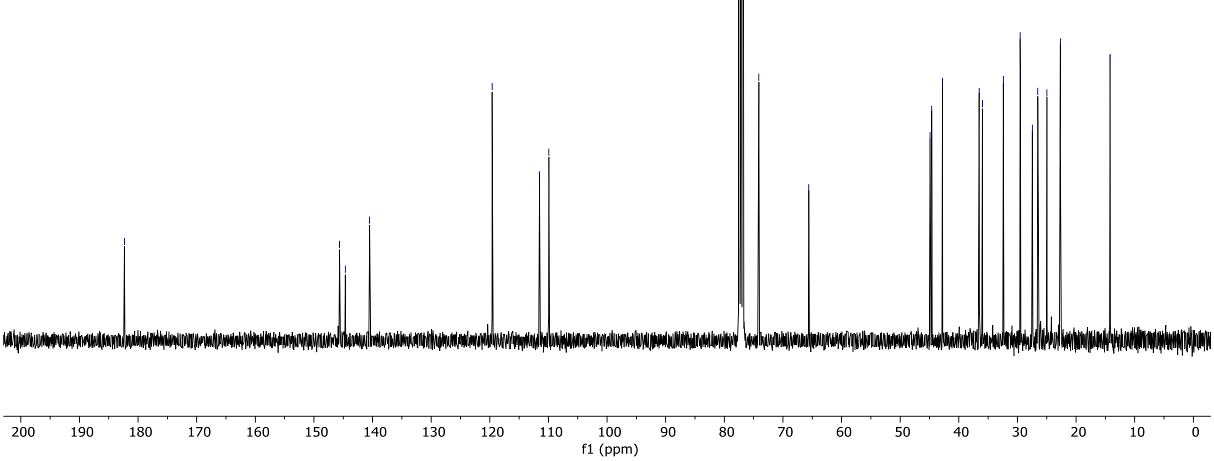

(3*R,3a*S**,6*R**,12b*R**)-3a-Methyl-*N*-((3*R**,3a*S**,6*R**,12b*R**)-3a-methyl-2,3,3a,4,5,6-hexahydro-1*H*-3,6-methanocyclopenta[3,4]oxepino[2,3-*b*]indol-11-yl)-*N*-pentyl-2,3,3a,4,5,6-hexahydro-1*H*-3,6-methanocyclopenta[3,4]oxepino[2,3-*b*]indol-11-amine (20b)**

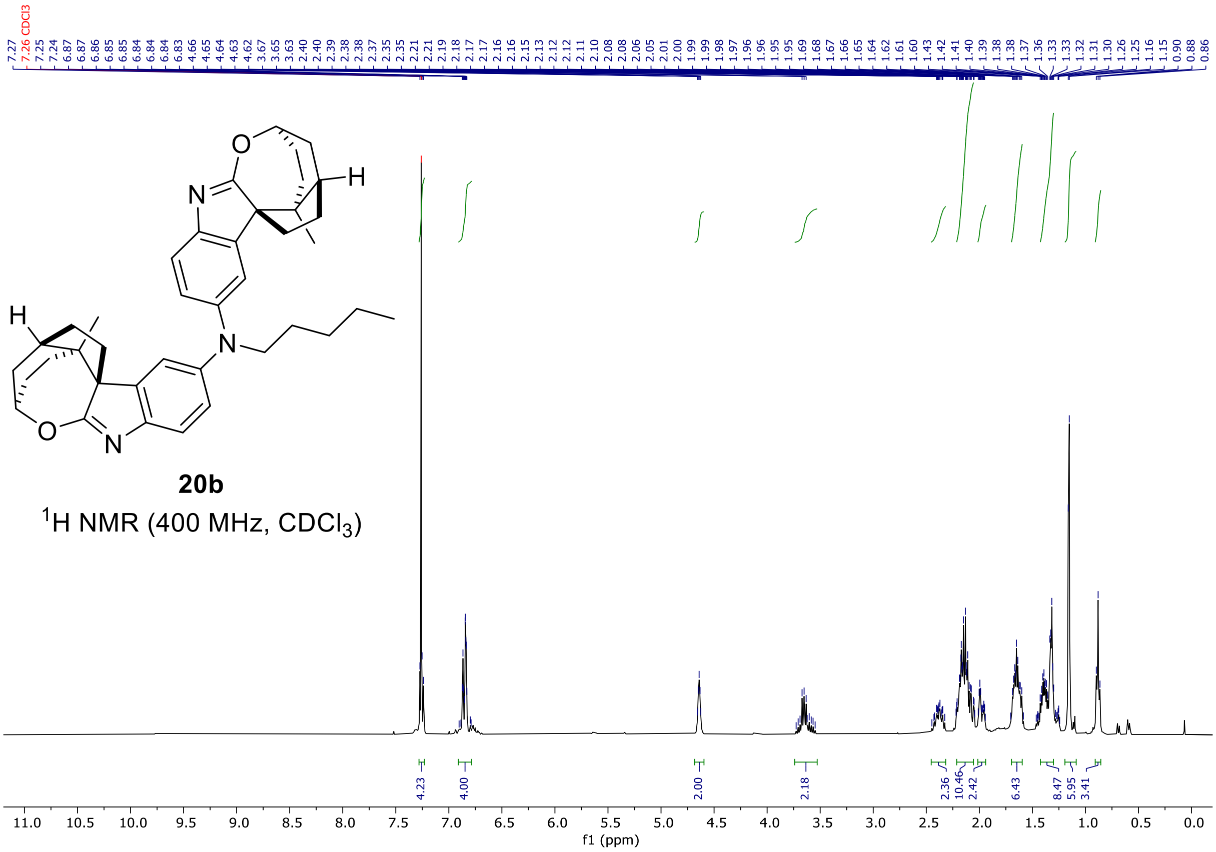

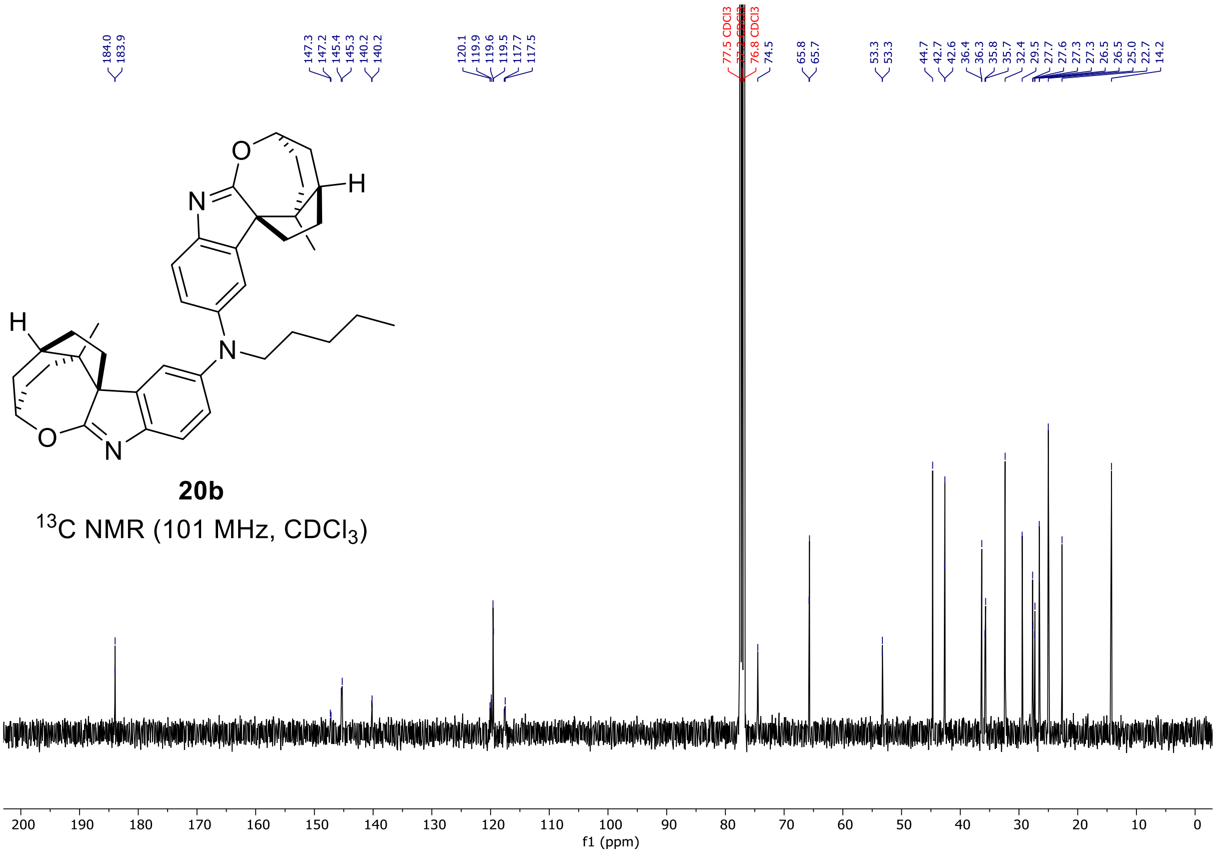

(3*R,3a*S**,6*R**,12b*R**)-*N*-Butyl-*N*,3a-dimethyl-2,3,3a,4,5,6-hexahydro-1*H*-3,6-methanocyclopenta[3,4]oxepino[2,3-*b*]indol-11-amine (19d)**

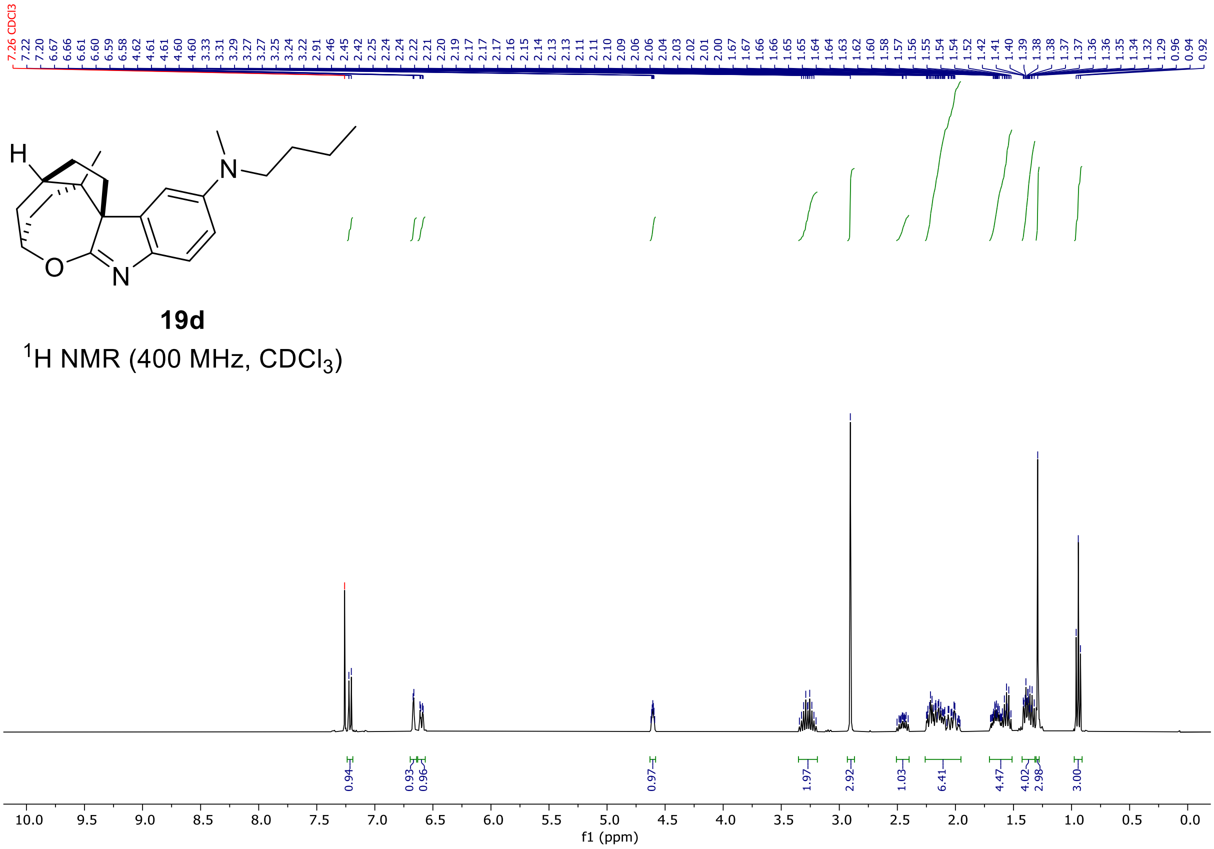

19d

^1H NMR (400 MHz, $CDCl_3$)

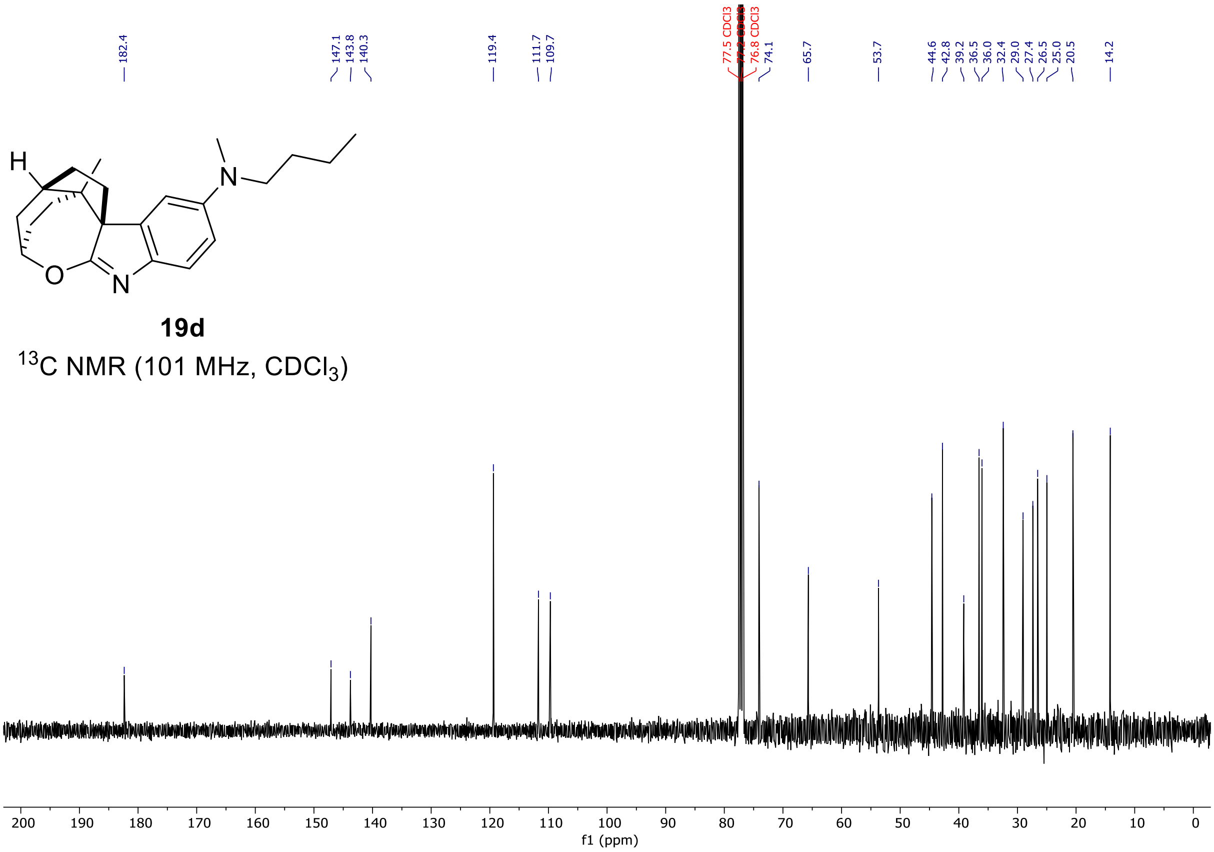

19d

^{13}C NMR (101 MHz, $CDCl_3$)

***tert*-Butyl 4-((3*R**,3a*S**,6*R**,12b*R**)-3a-methyl-2,3,3a,4,5,6-hexahydro-1*H*-3,6-methanocyclopenta[3,4]oxepino[2,3-*b*]indol-11-yl)piperazine-1-carboxylate (19e)**

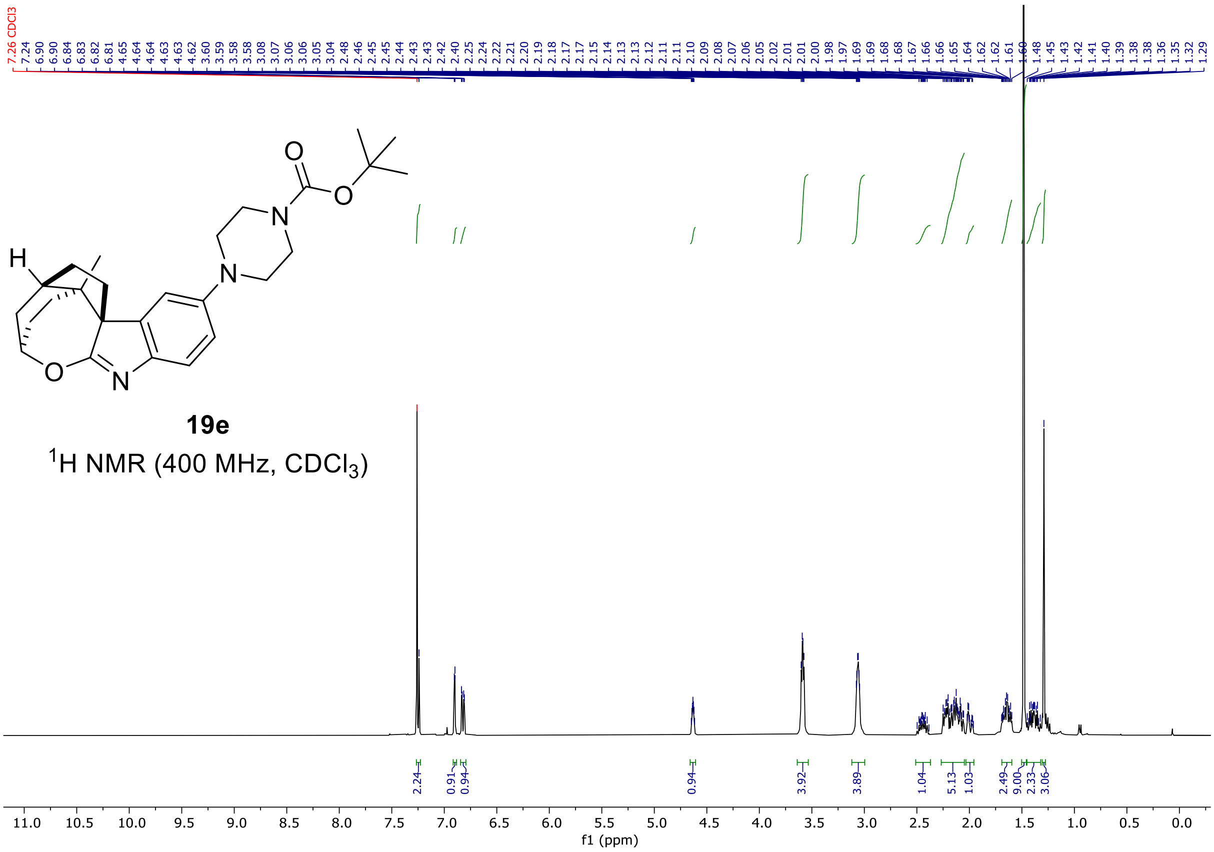

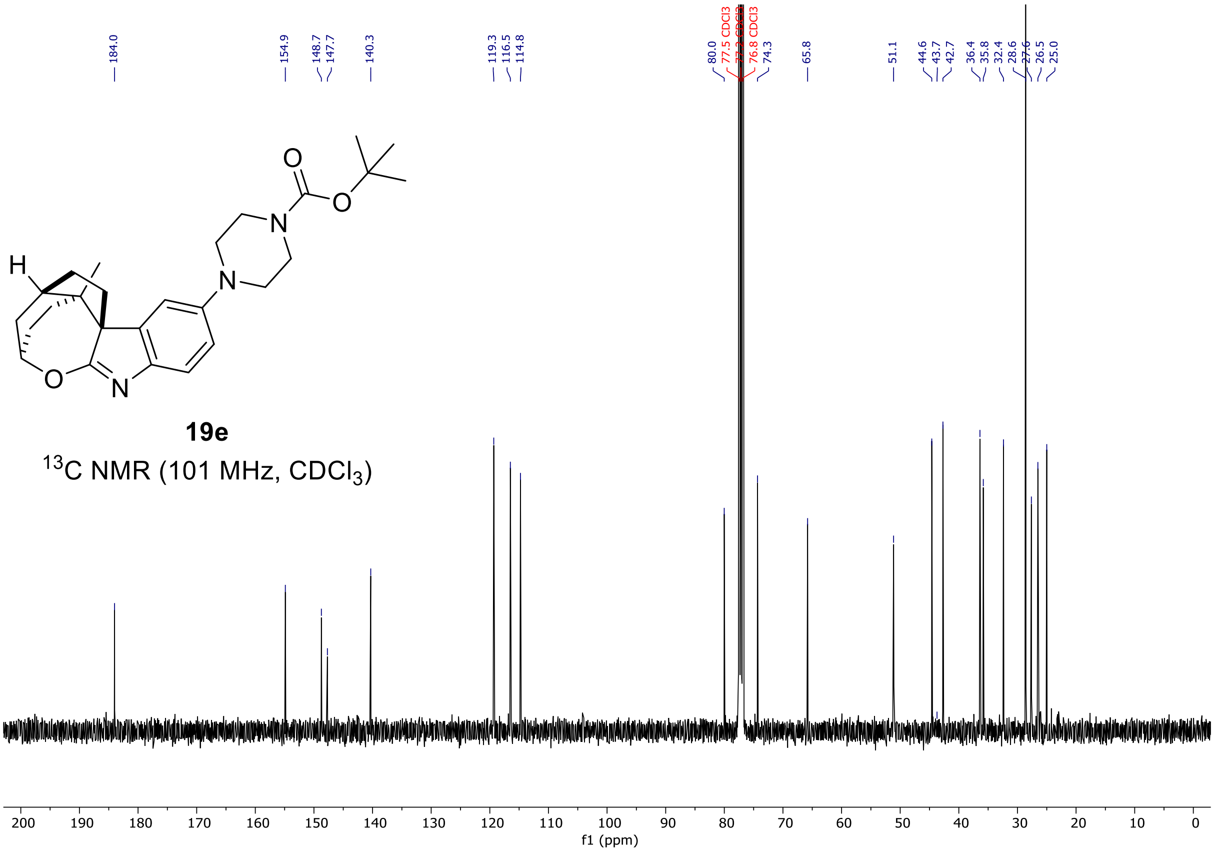

(3*R,3a*S**,6*R**,12b*R**)-3a-Methyl-11-phenyl-2,3,3a,4,5,6-hexahydro-1*H*-3,6-methanocyclopenta[3,4]oxepino[2,3-*b*]indole (21a)**

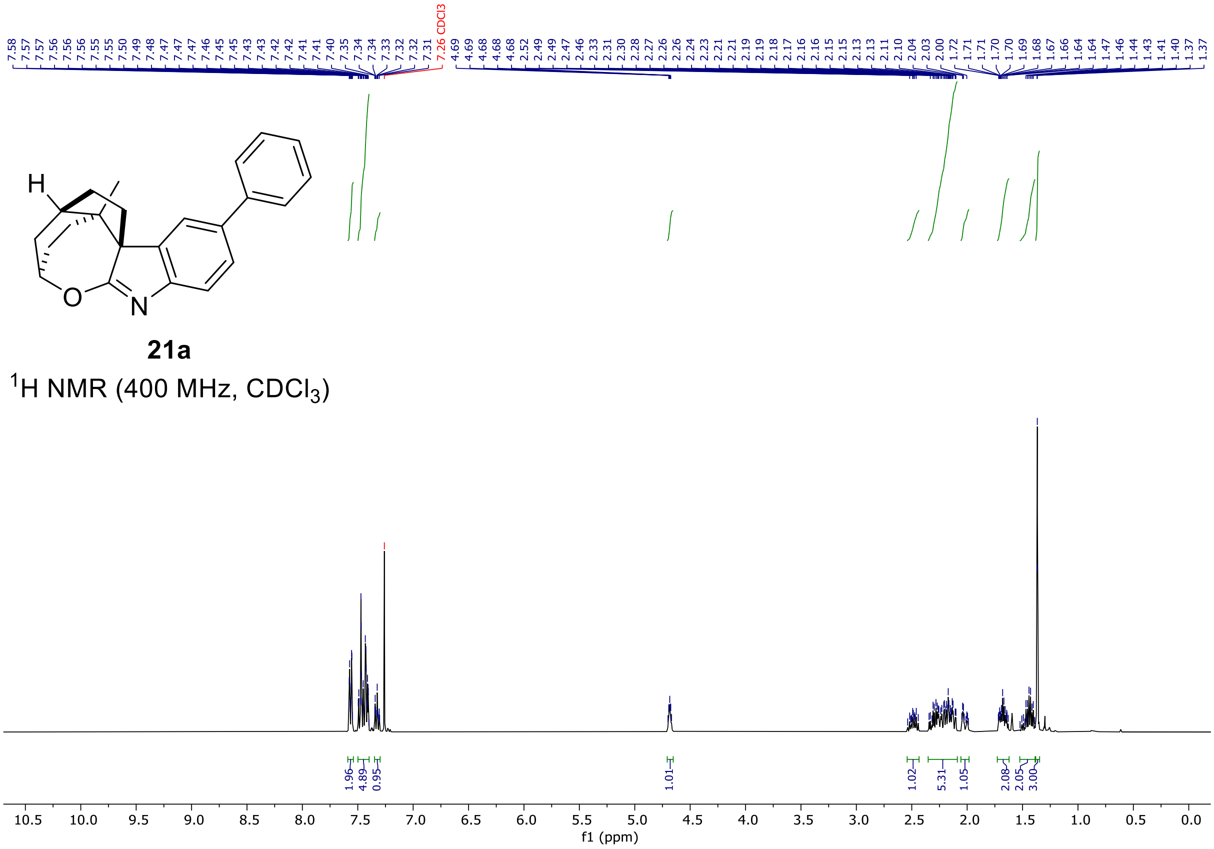

21a

^{1}H NMR (400 MHz, $CDCl_3$)

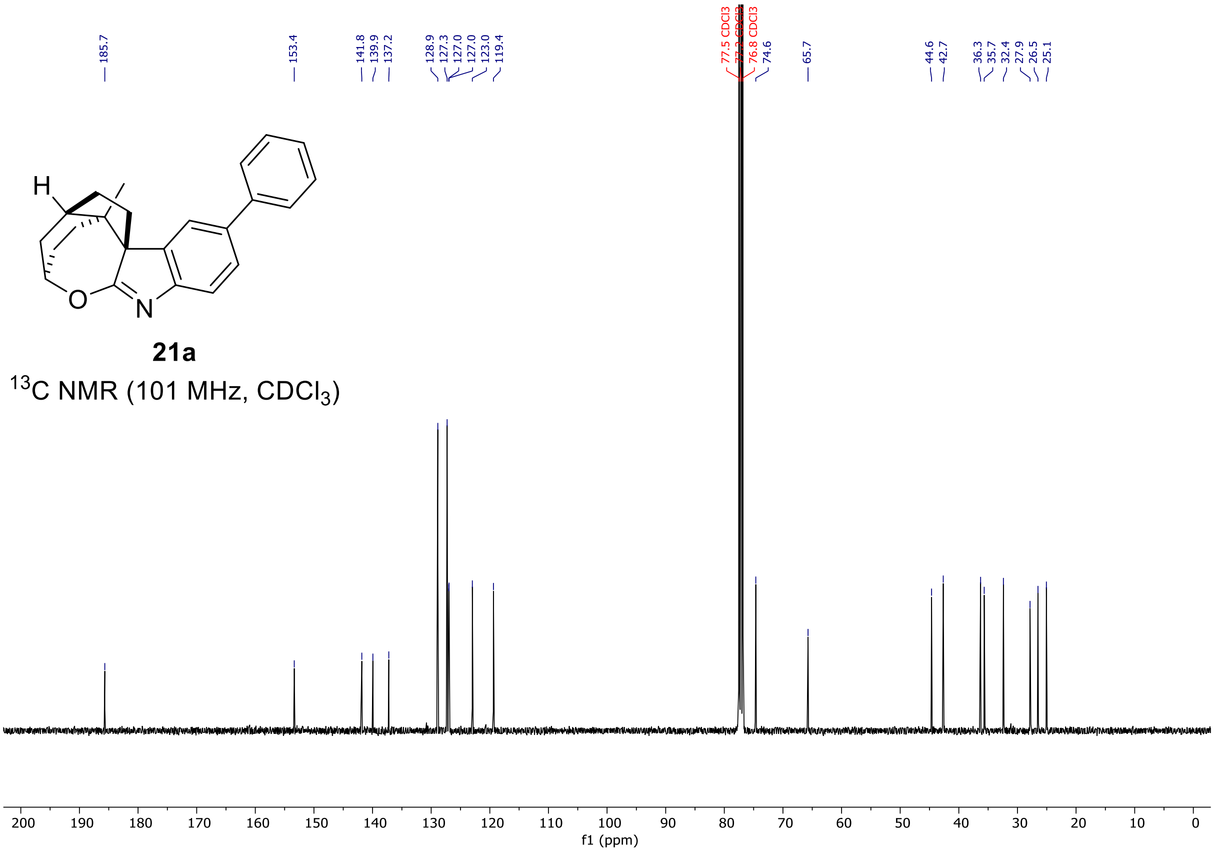

21a

^{13}C NMR (101 MHz, $CDCl_3$)

(3*R,3a*S**,6*R**,12b*R**)-11-(4-Methoxyphenyl)-3a-methyl-11-phenyl-2,3,3a,4,5,6-hexahydro-1*H*-3,6-methanocyclopenta[3,4]oxepino[2,3-*b*]indole (21b)**

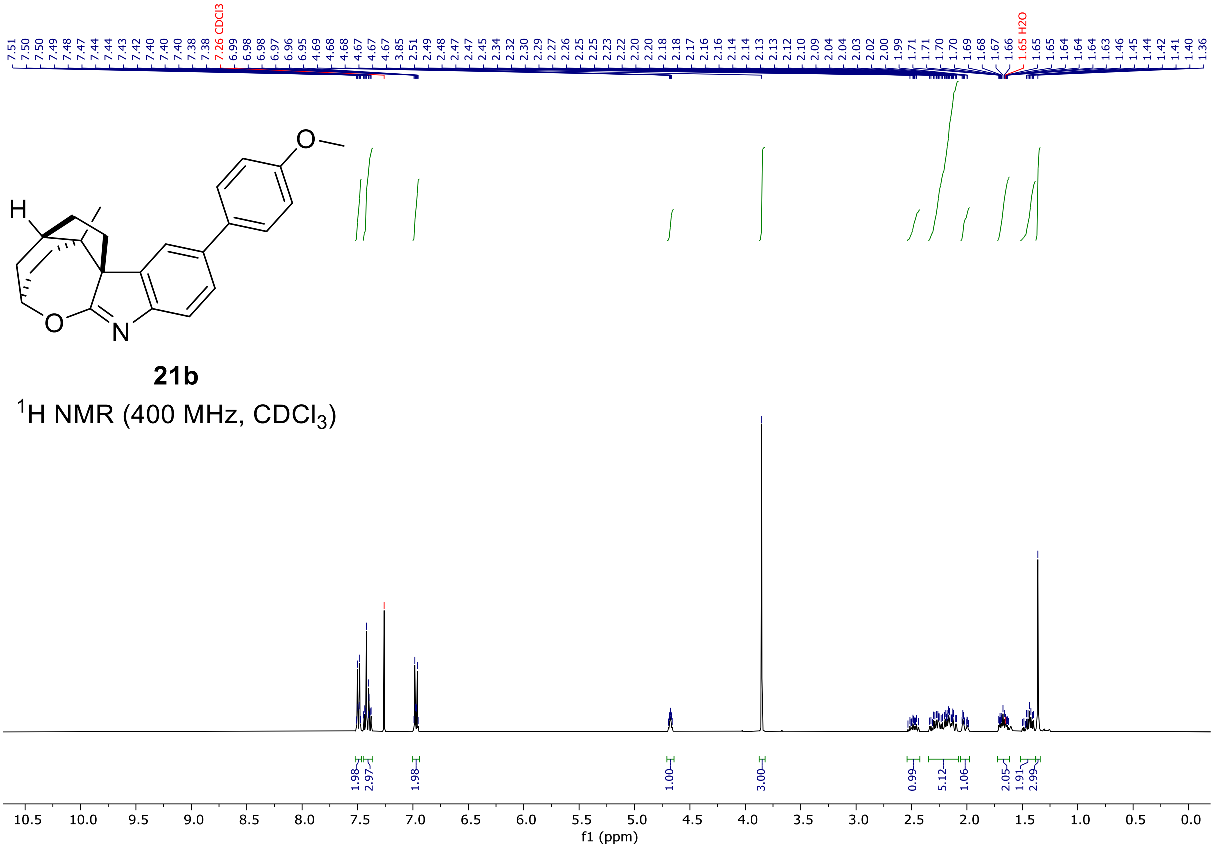

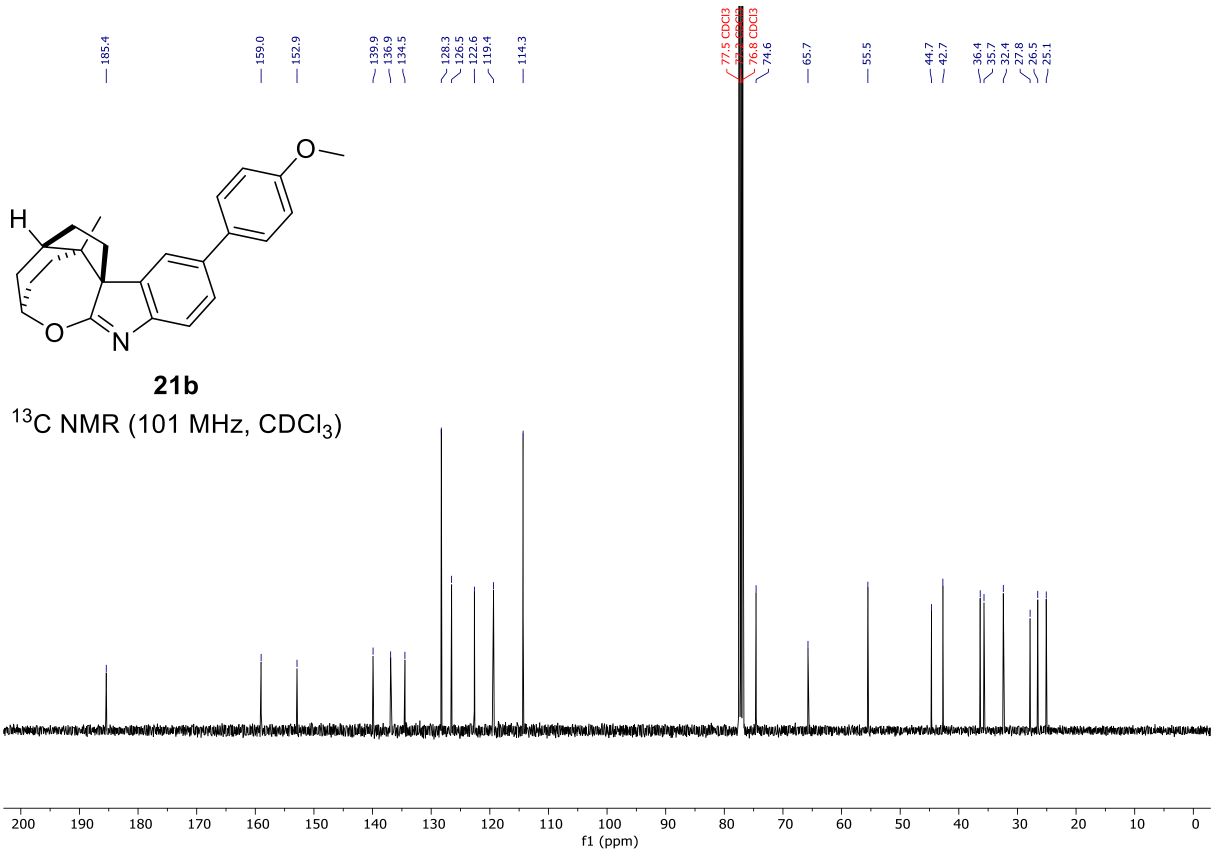

(3*R,3a*S**,6*R**,12b*R**)-3a-Methyl-11-(piperazin-1-yl)-2,3,3a,4,5,6-hexahydro-1*H*-3,6-methanocyclopenta[3,4]oxepino[2,3-*b*]indole (22)**

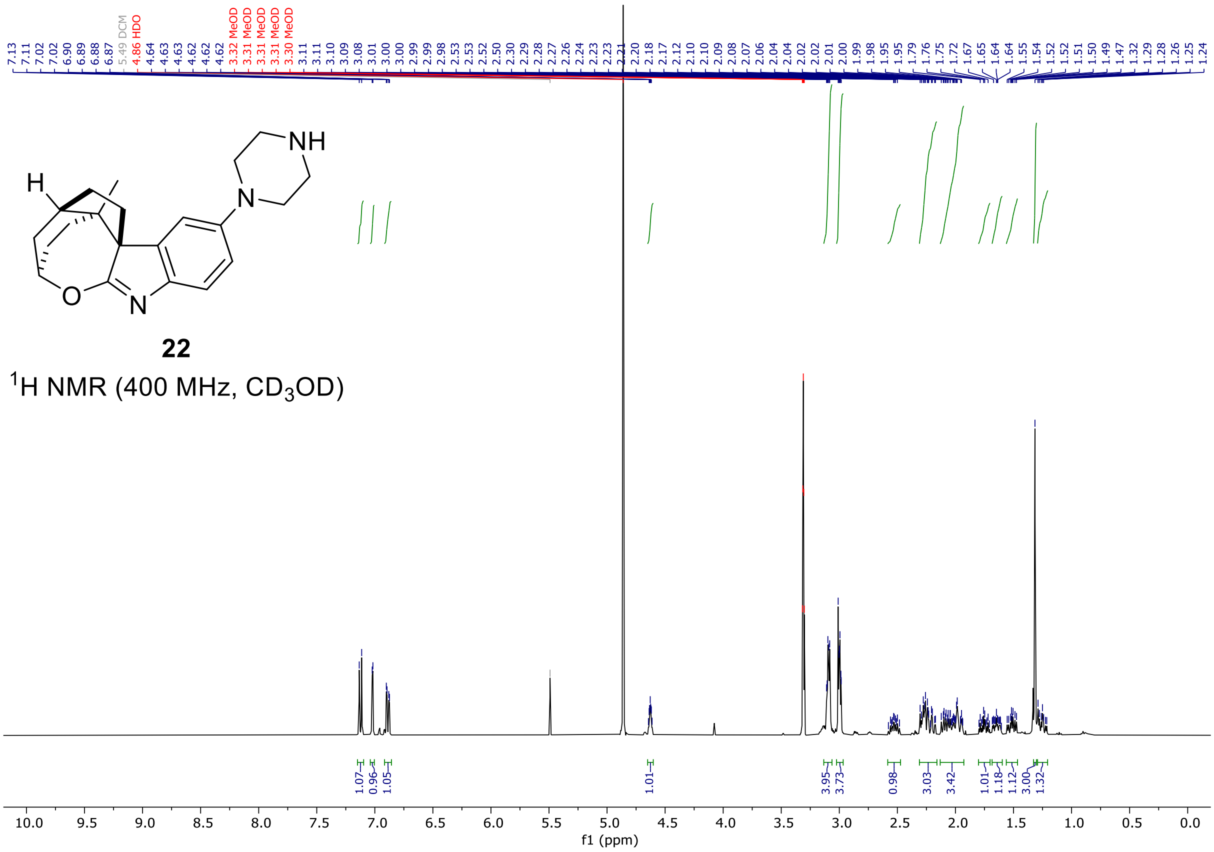

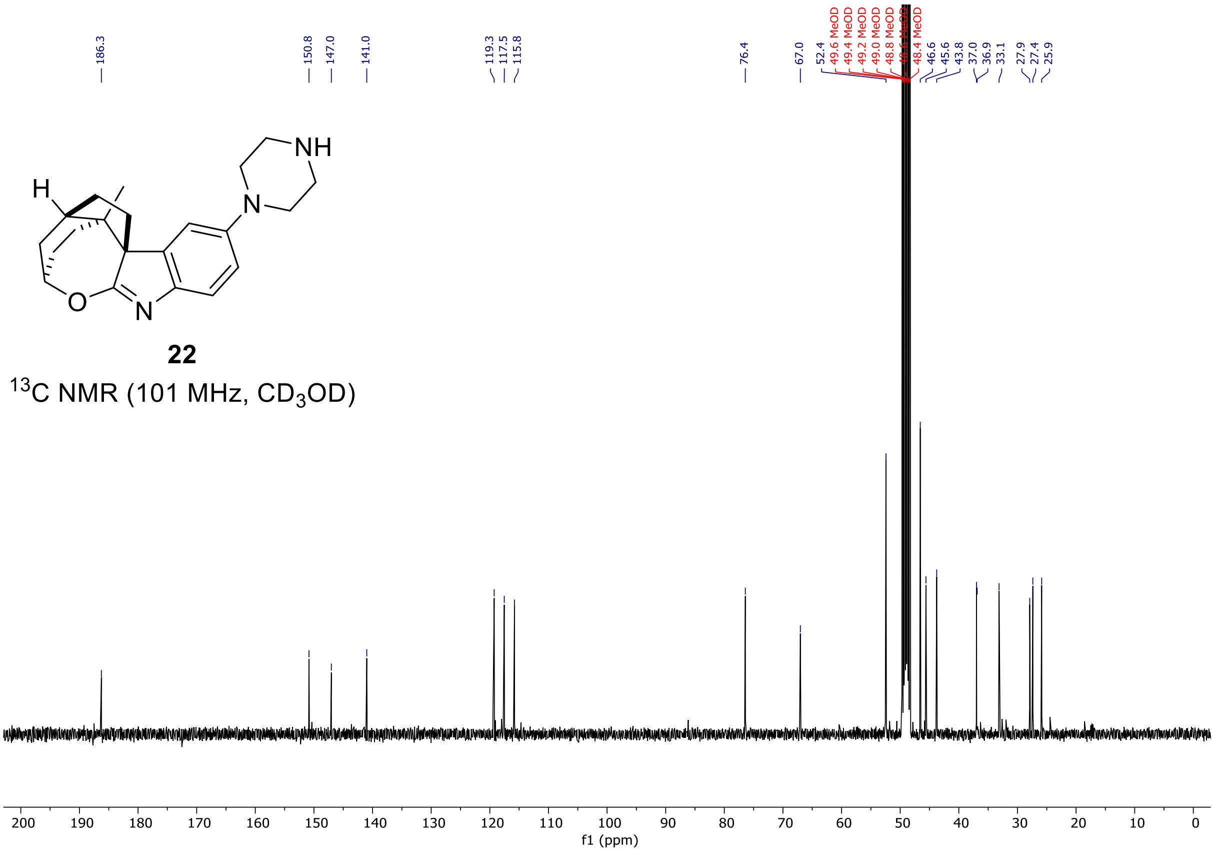

1-(4-((3*R,3a*S**,6*R**,12b*R**)-3a-Methyl-2,3,3a,4,5,6-hexahydro-1*H*-3,6-methanocyclopenta[3,4]oxepino[2,3-*b*]indol-11-yl)piperazin-1-yl)butan-1-one (23)**

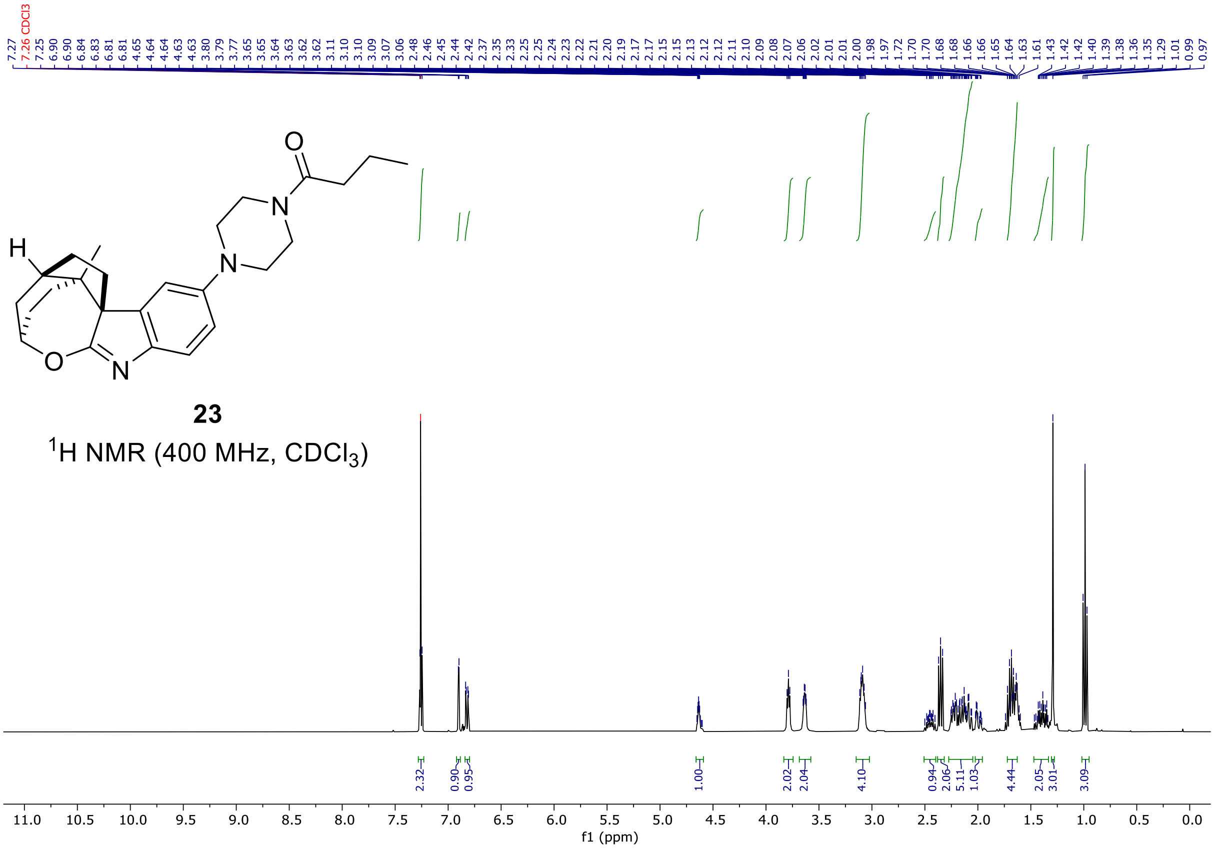

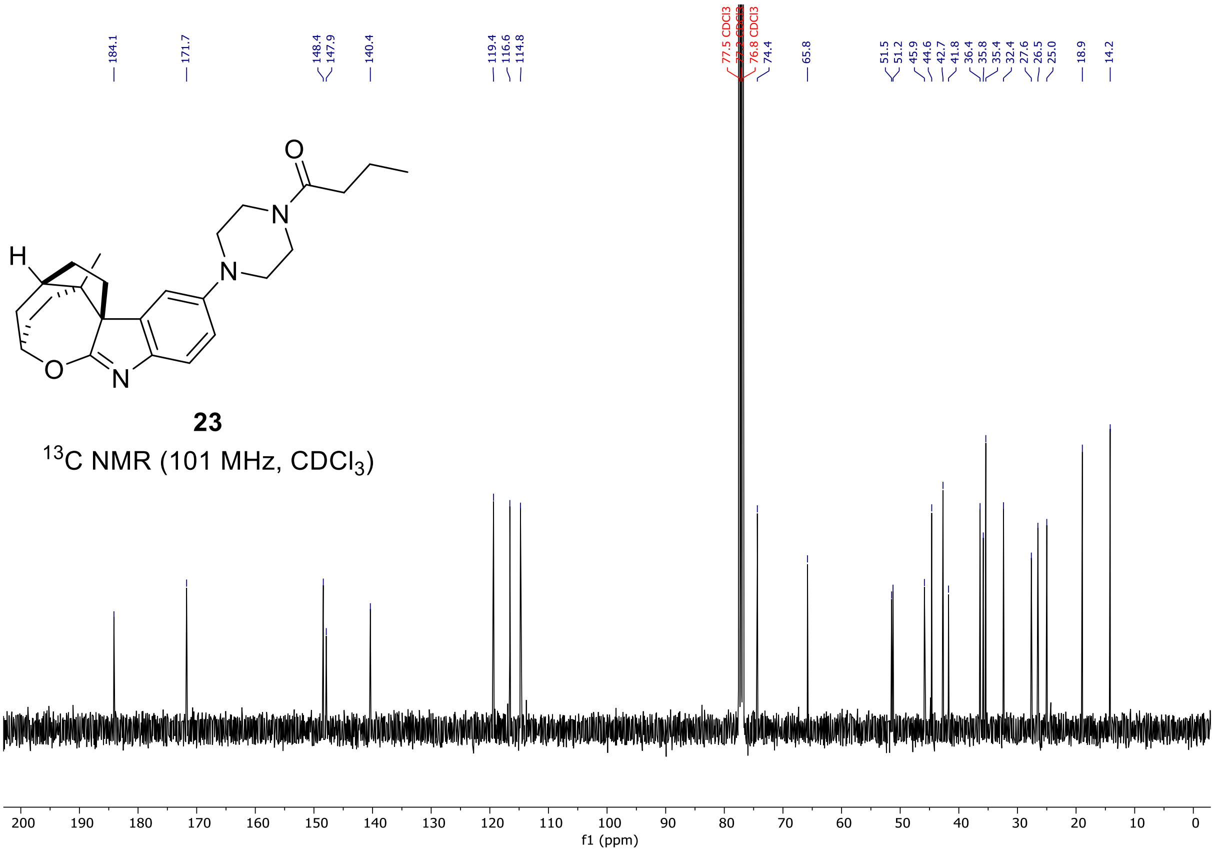

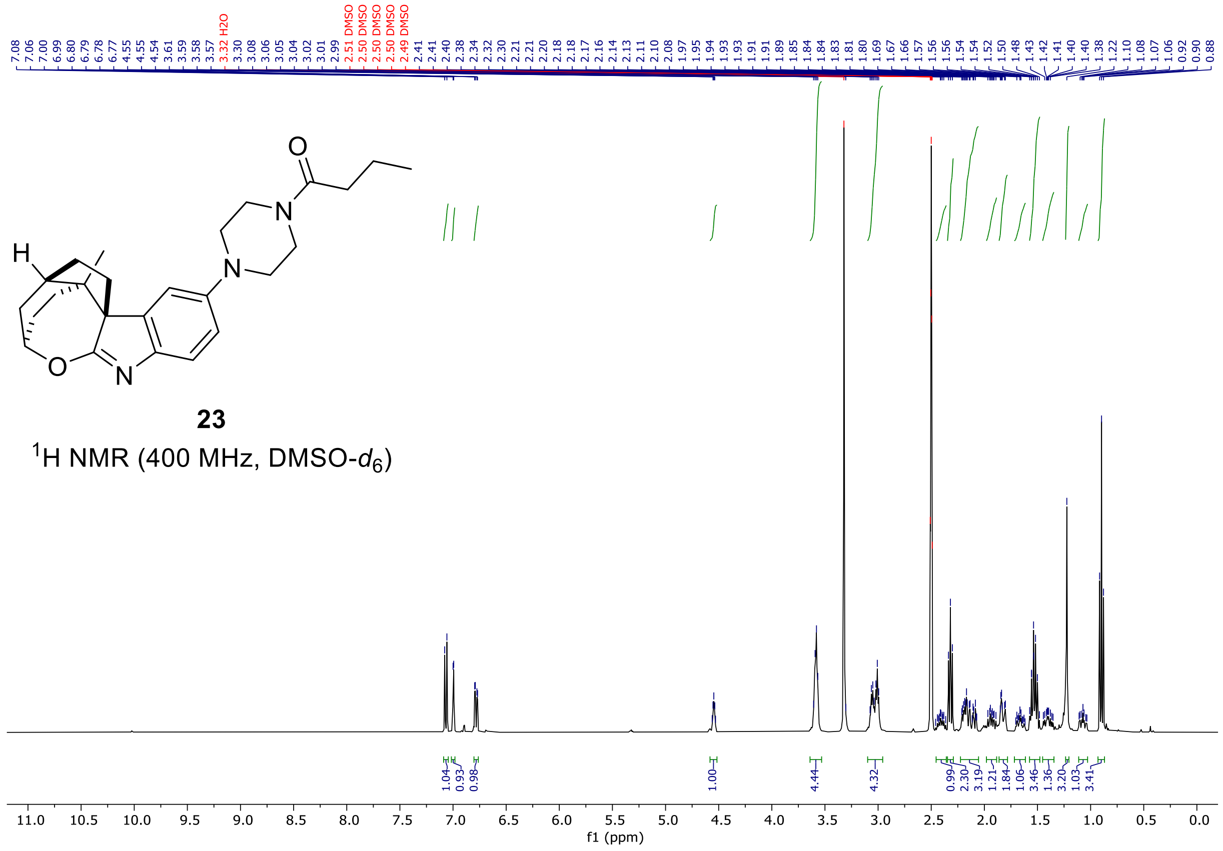

23

^{1}H NMR (400 MHz, DMSO-d_6)

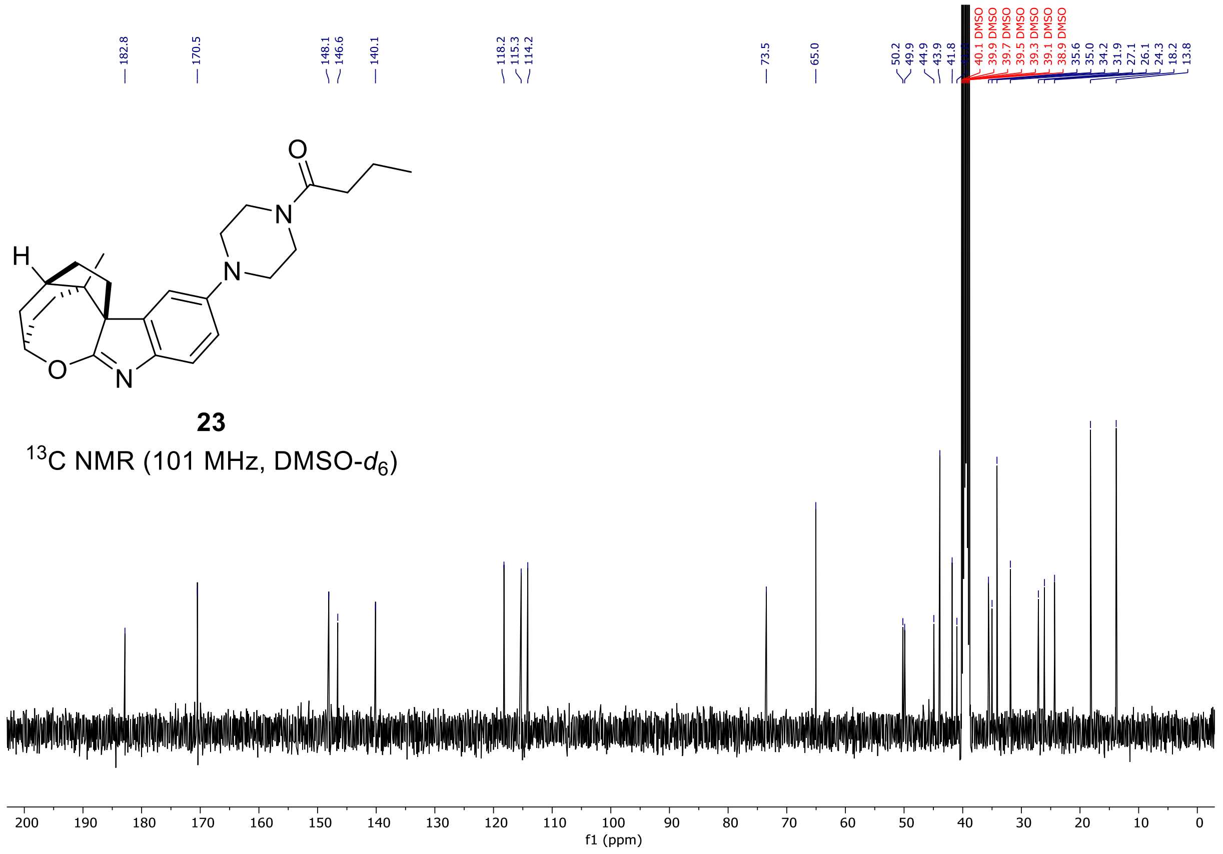

23

^{13}C NMR (101 MHz, DMSO-d_6)

(3*R,3a*S**,6*R**,12b*R**)-3a-Methyl-11-(4-(pyridin-4-ylmethyl)piperazin-1-yl)-2,3,3a,4,5,6-hexahydro-1H-3,6-methanocyclopenta[3,4]oxepino[2,3-b]índole (24)**

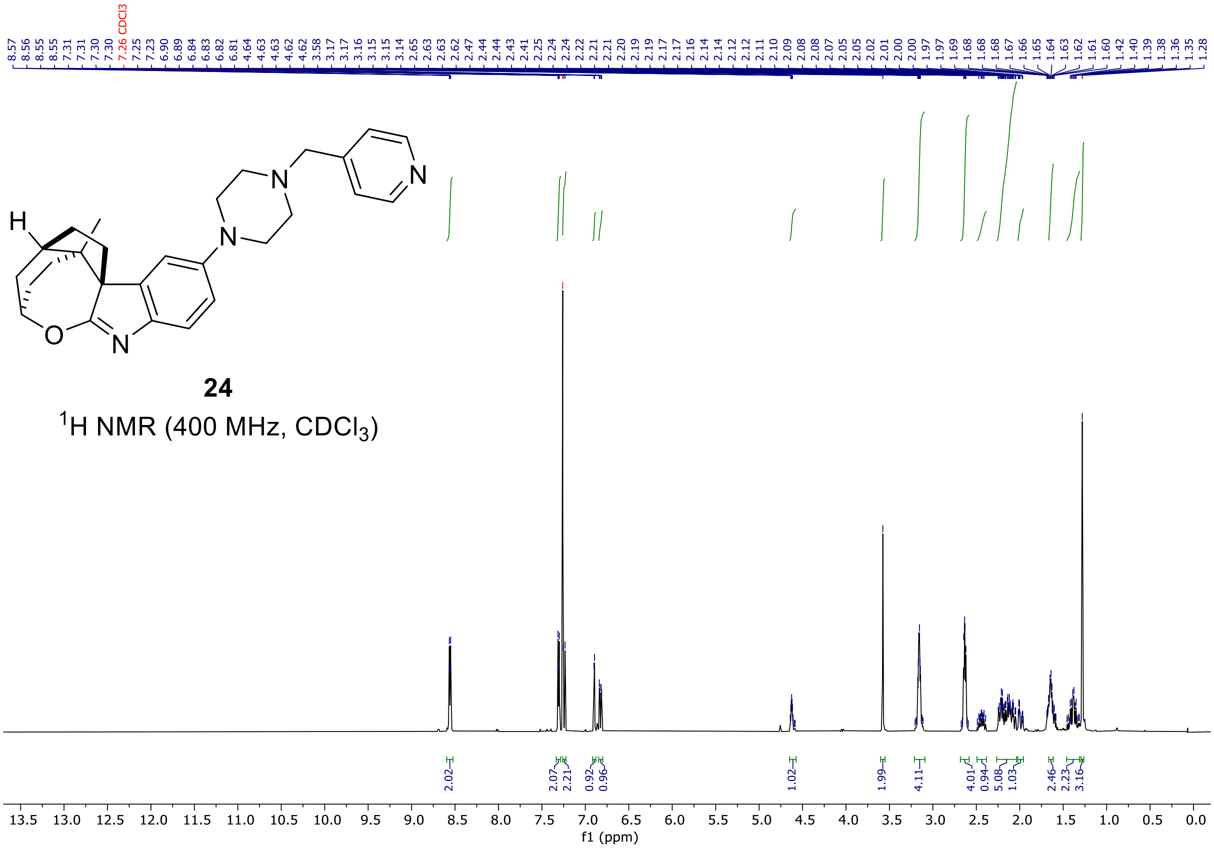

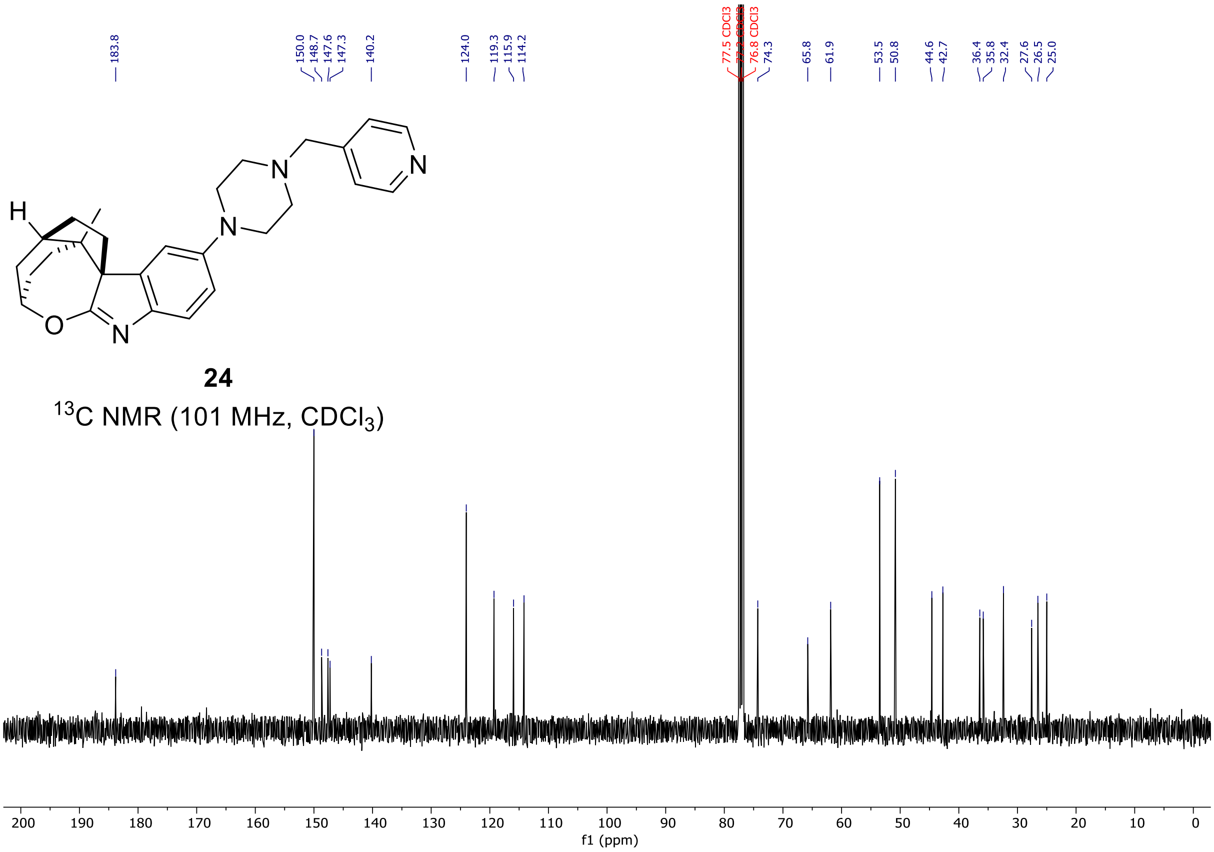

(3*R*,3a*S*,6*R*,12b*R*)-11-Bromo-3a-methyl-2,3,3a,4,5,6-hexahydro-1*H*-3,6-methanocyclopenta[3,4]oxepino[2,3-*b*]indole ((+)-15b)

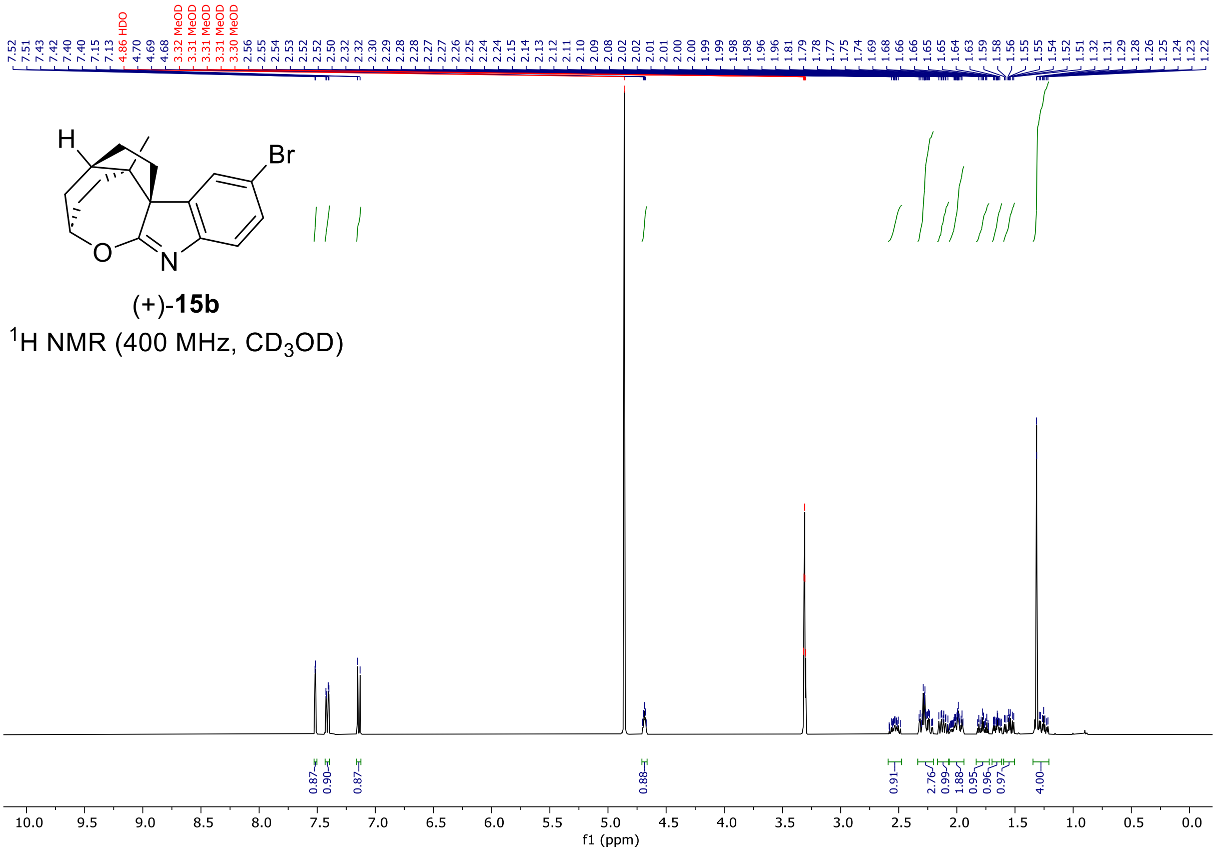

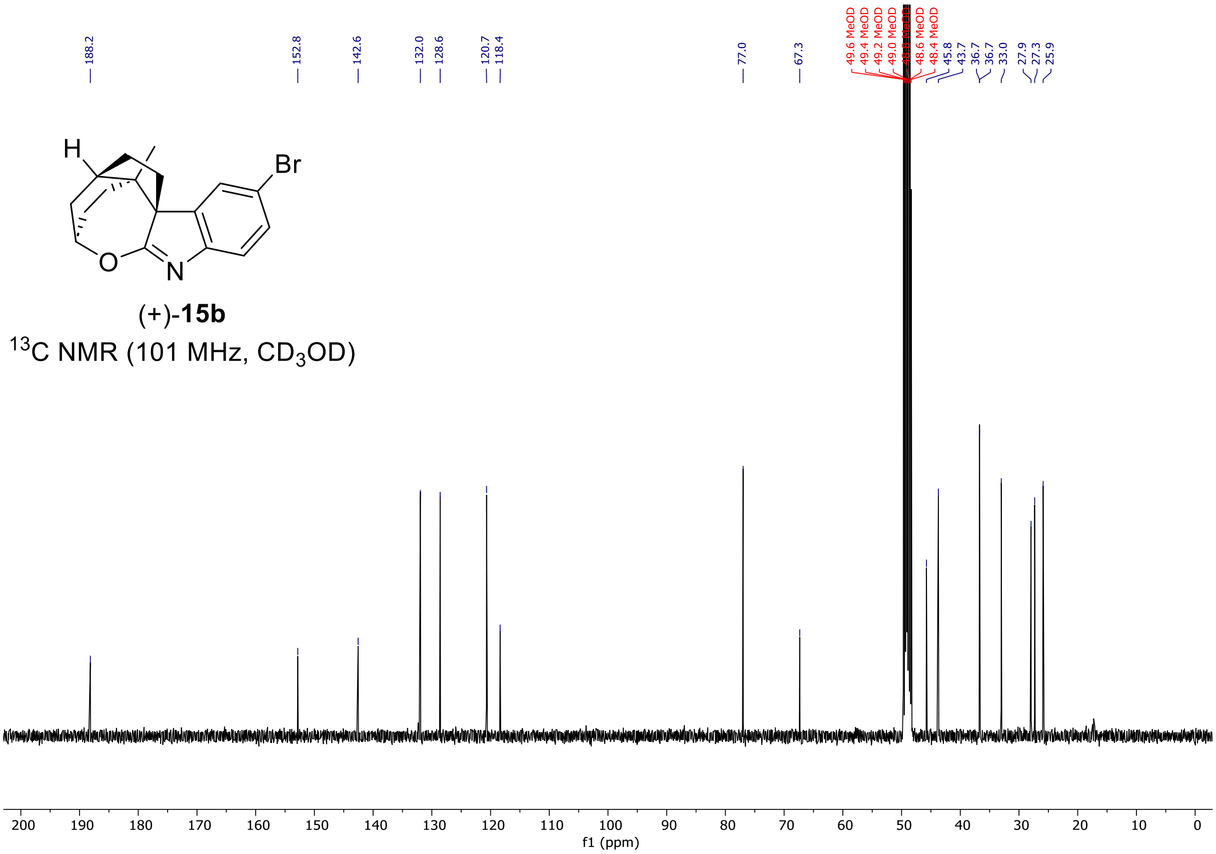

(3*S*,3a*R*,6*S*,12b*S*)-11-Bromo-3a-methyl-2,3,3a,4,5,6-hexahydro-1*H*-3,6-methanocyclopenta[3,4]oxepino[2,3-*b*]indole ((–)-15b)

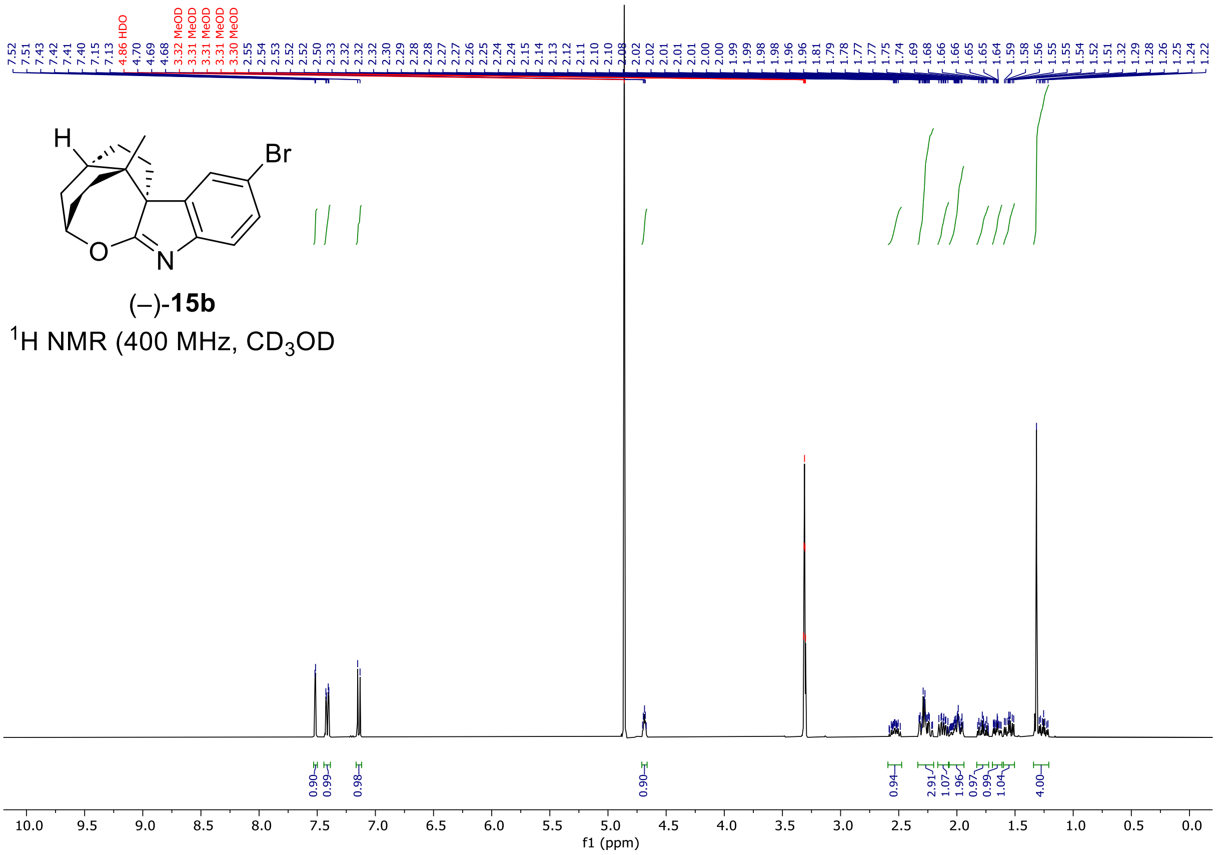

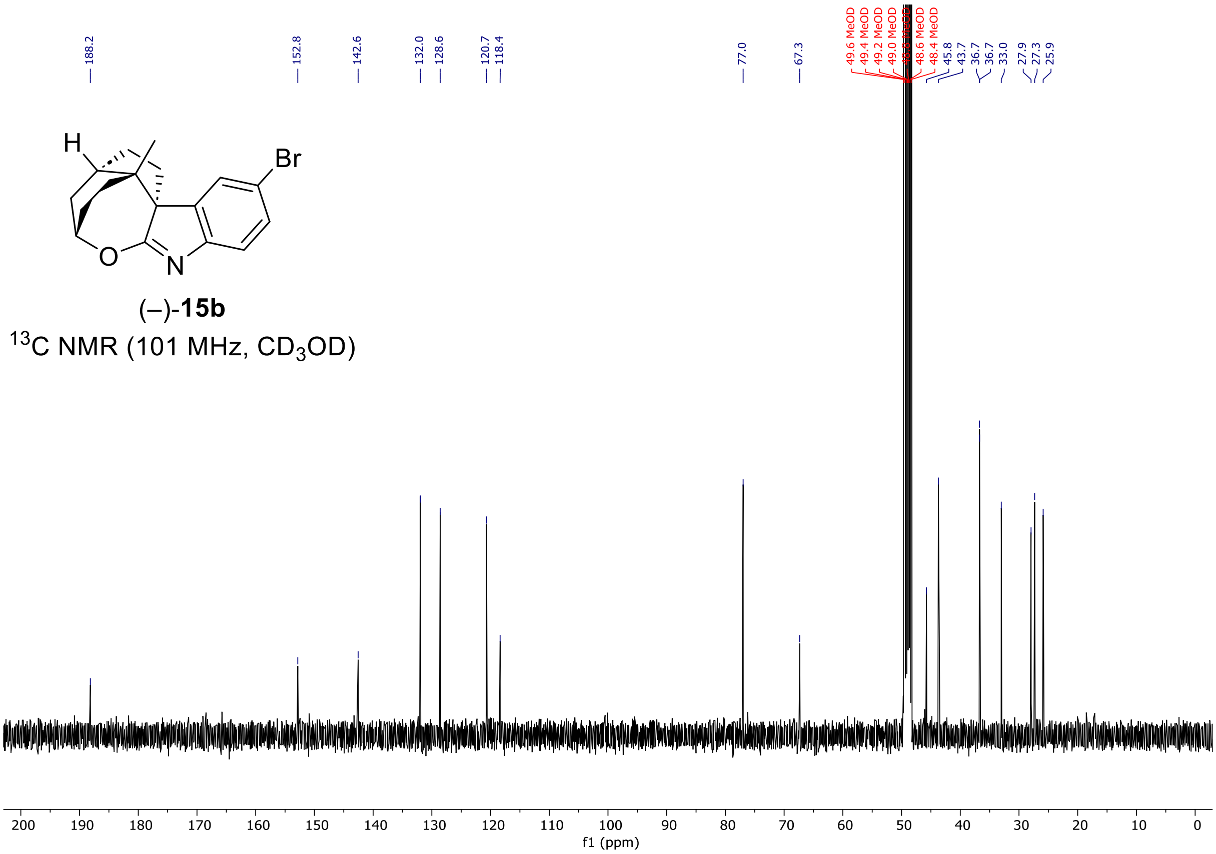

(3*R*,3a*S*,6*R*,12b*R*)-3a-Methyl-11-morpholino-2,3,3a,4,5,6-hexahydro-1*H*-3,6-methanocyclopenta[3,4]oxepino[2,3-*b*]indole, (+)-asteroxin-1 ((+)-19a)

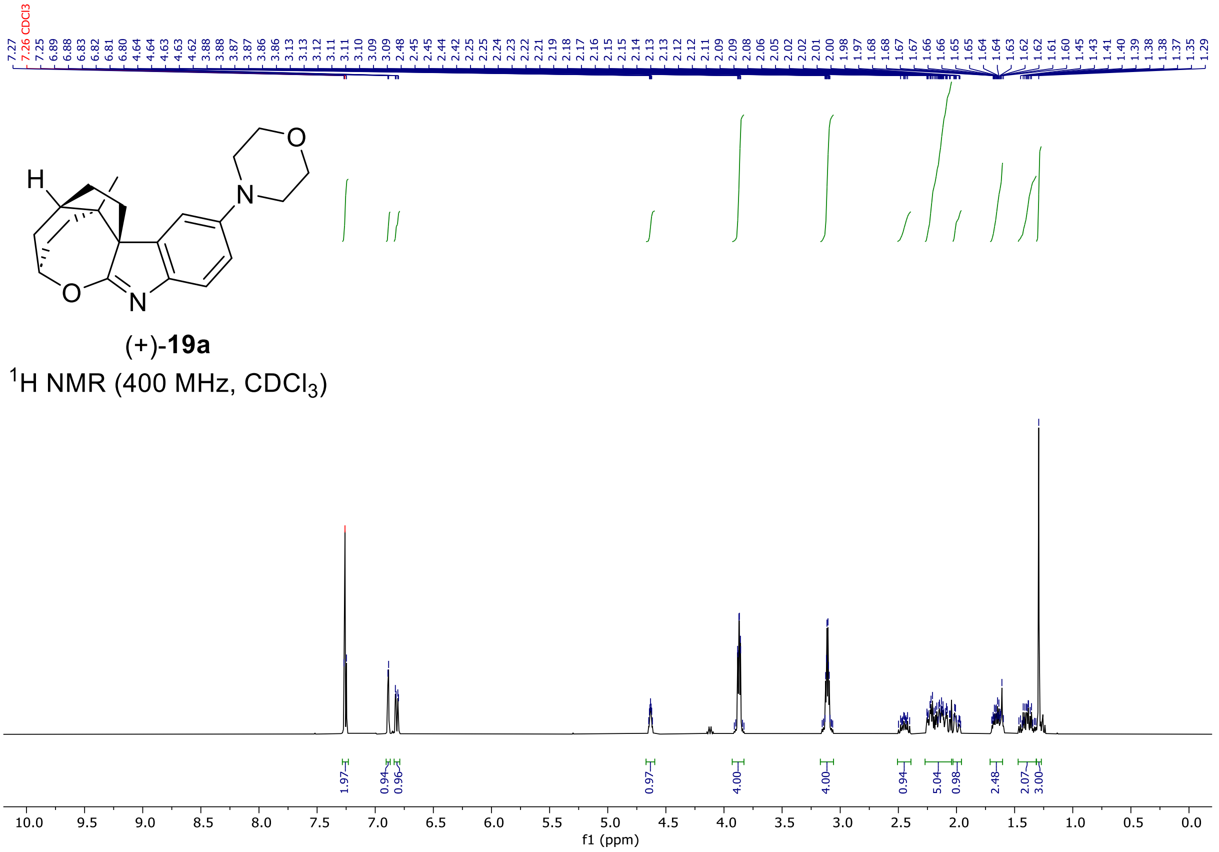

(+)-**19a**

^{1}H NMR (400 MHz, $CDCl_3$)

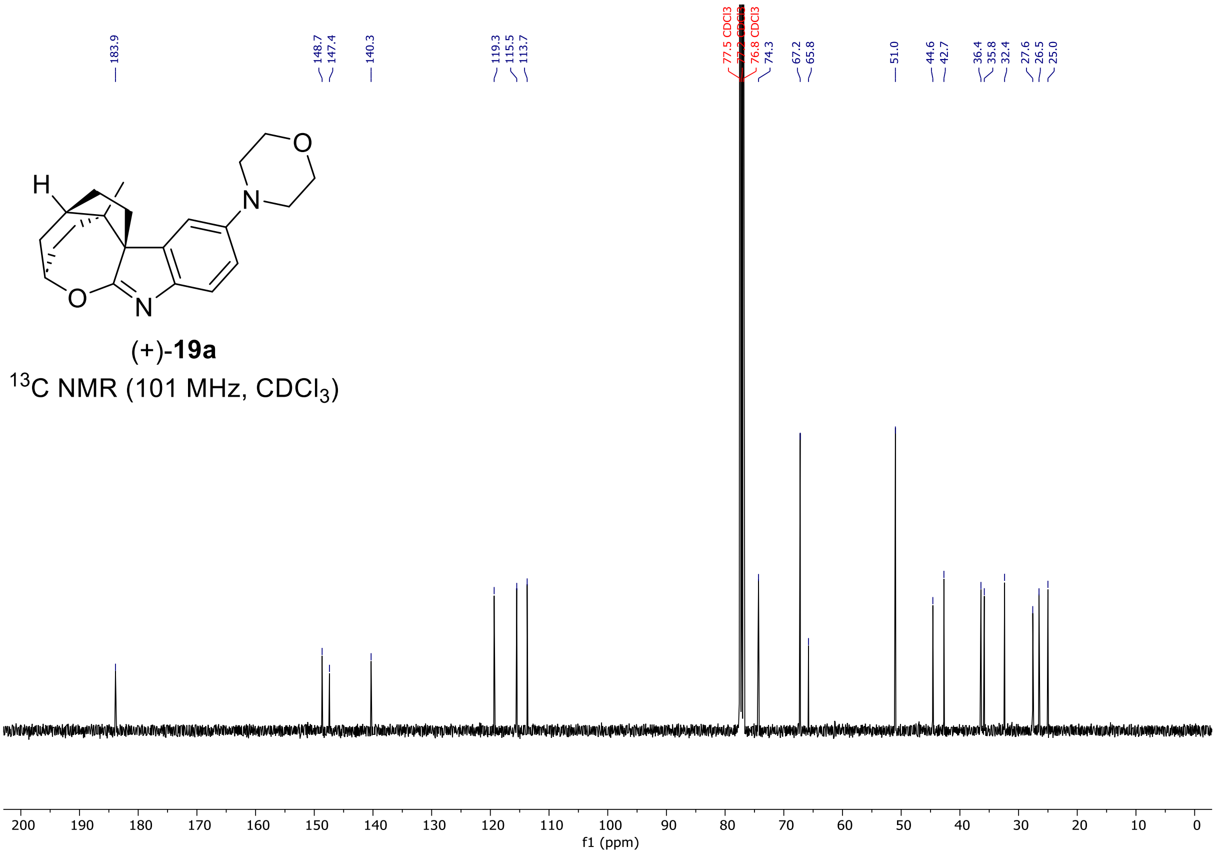

(+)-**19a**

^{13}C NMR (101 MHz, $CDCl_3$)

(3*S*,3a*R*,6*S*,12b*S*)-3a-Methyl-11-morpholino-2,3,3a,4,5,6-hexahydro-1*H*-3,6-methanocyclopenta[3,4]oxepino[2,3-*b*]indole, (–)-asteroxin-1 ((–)-19a)

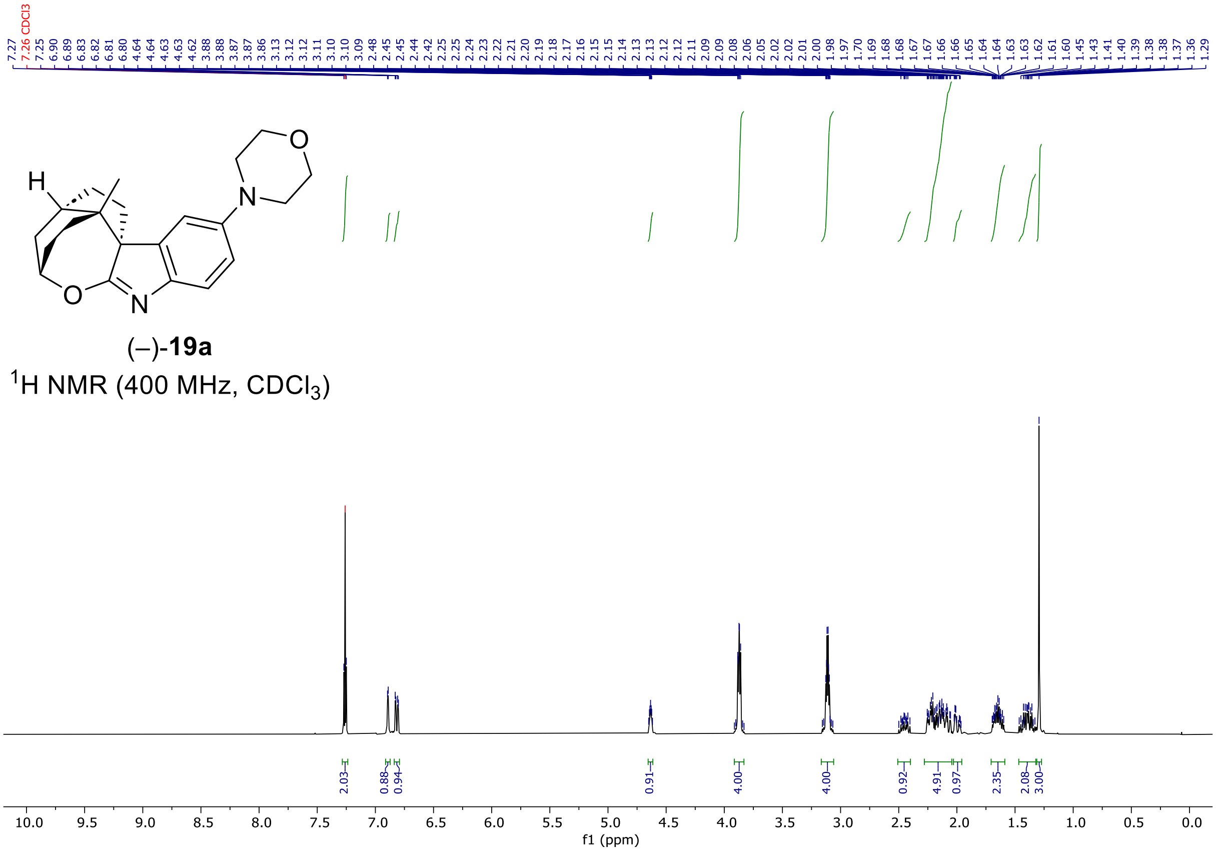

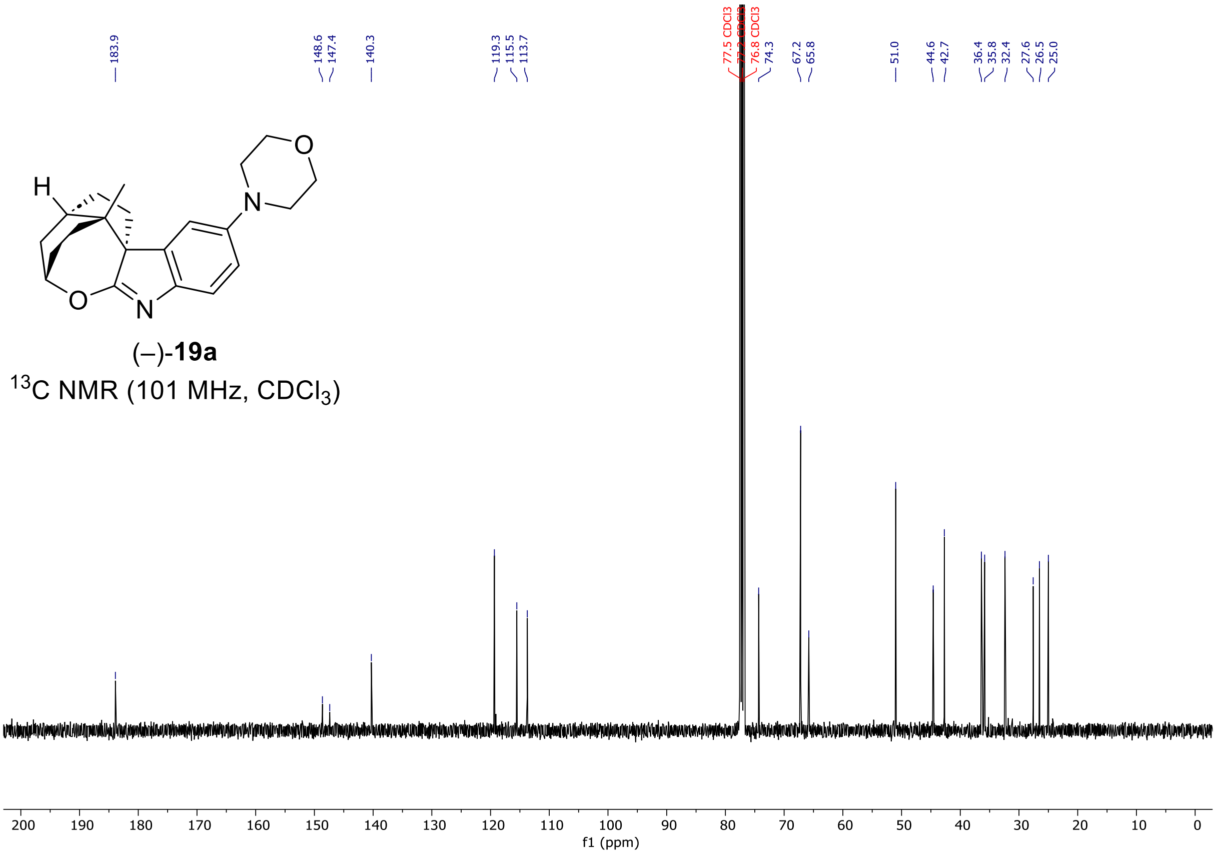

IR spectra

(3*R,3a*S**,6*R**,12b*R**)-3a-Methyl-2,3,3a,4,5,6-hexahydro-1*H*-3,6-methanocyclopenta[3,4]oxepino[2,3-*b*]indole (15a)**

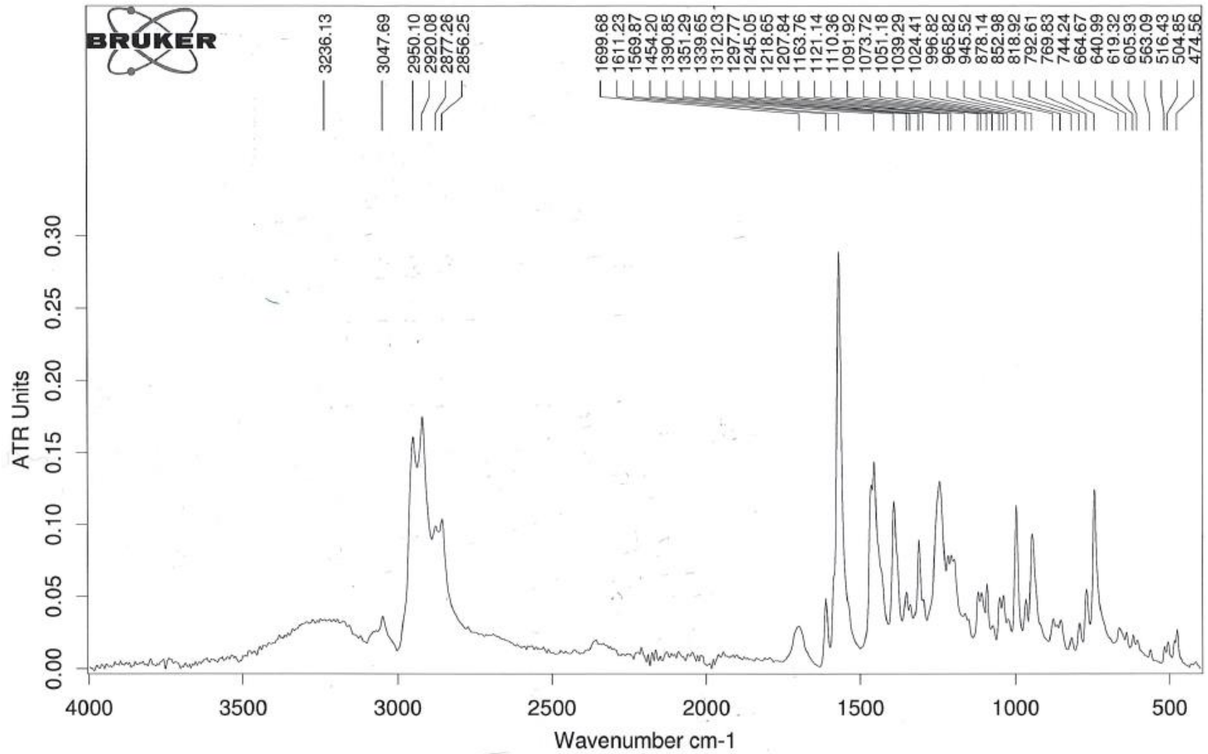

(3*R,3a*S**,6*R**,12b*R**)-11-Bromo-3a-methyl-2,3,3a,4,5,6-hexahydro-1*H*-3,6-methanocyclopenta[3,4]oxepino[2,3-*b*]indole (15b)**

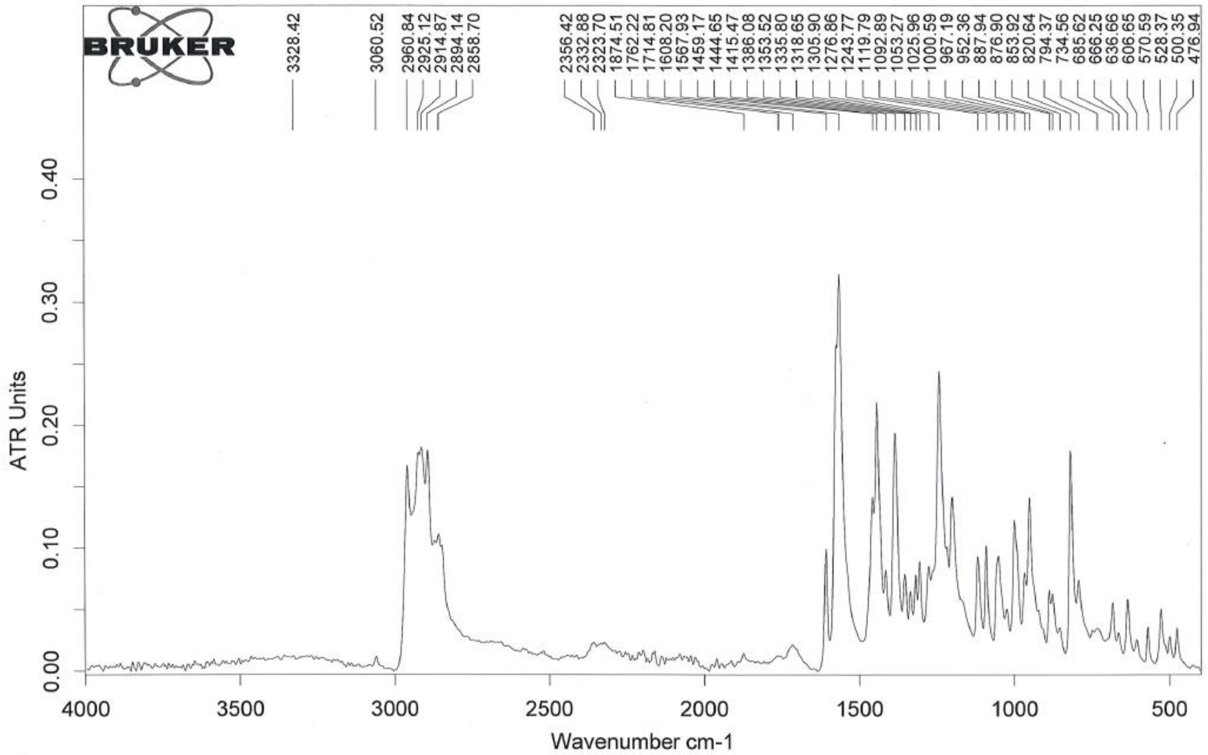

(3*R,3a*S**,6*R**,12b*R**)-11-Chloro-3a-methyl-2,3,3a,4,5,6-hexahydro-1*H*-3,6-methanocyclopenta[3,4]oxepino[2,3-*b*]indole (15c)**

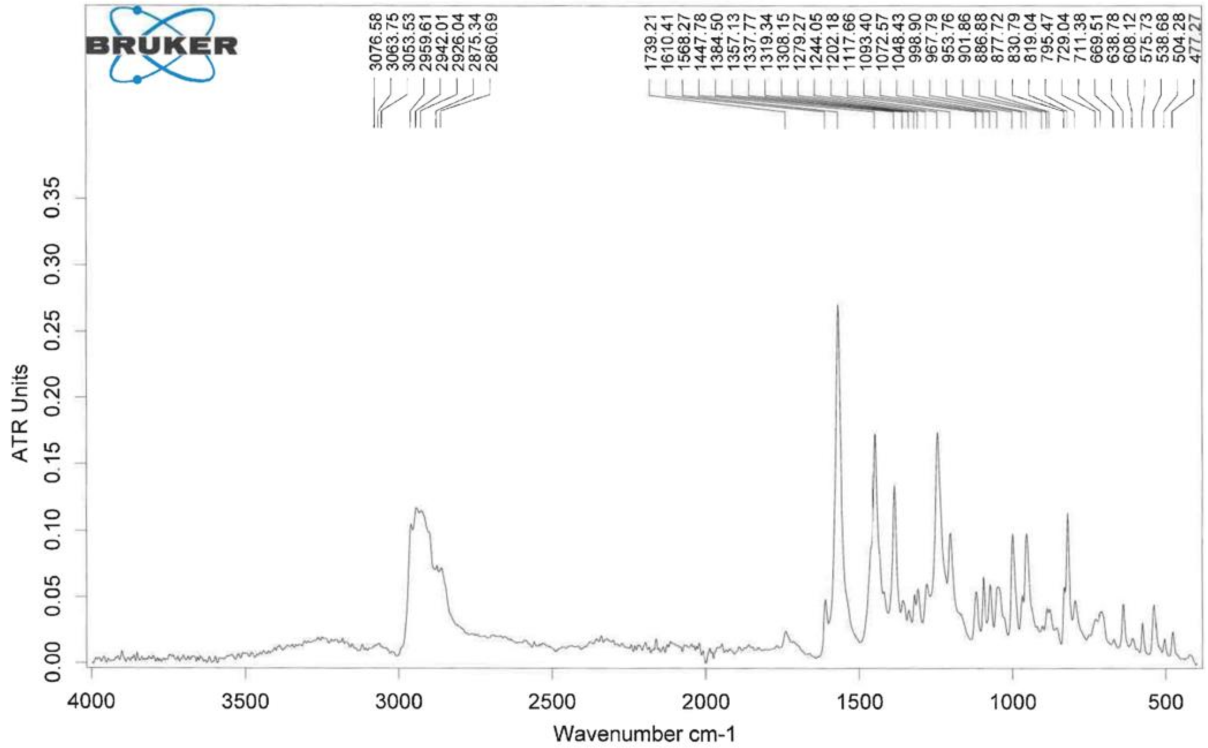

(3*R,3a*S**,6*R**,12b*R**)-11-Fluoro-3a-methyl-2,3,3a,4,5,6-hexahydro-1*H*-3,6-methanocyclopenta[3,4]oxepino[2,3-*b*]indole (15d)**

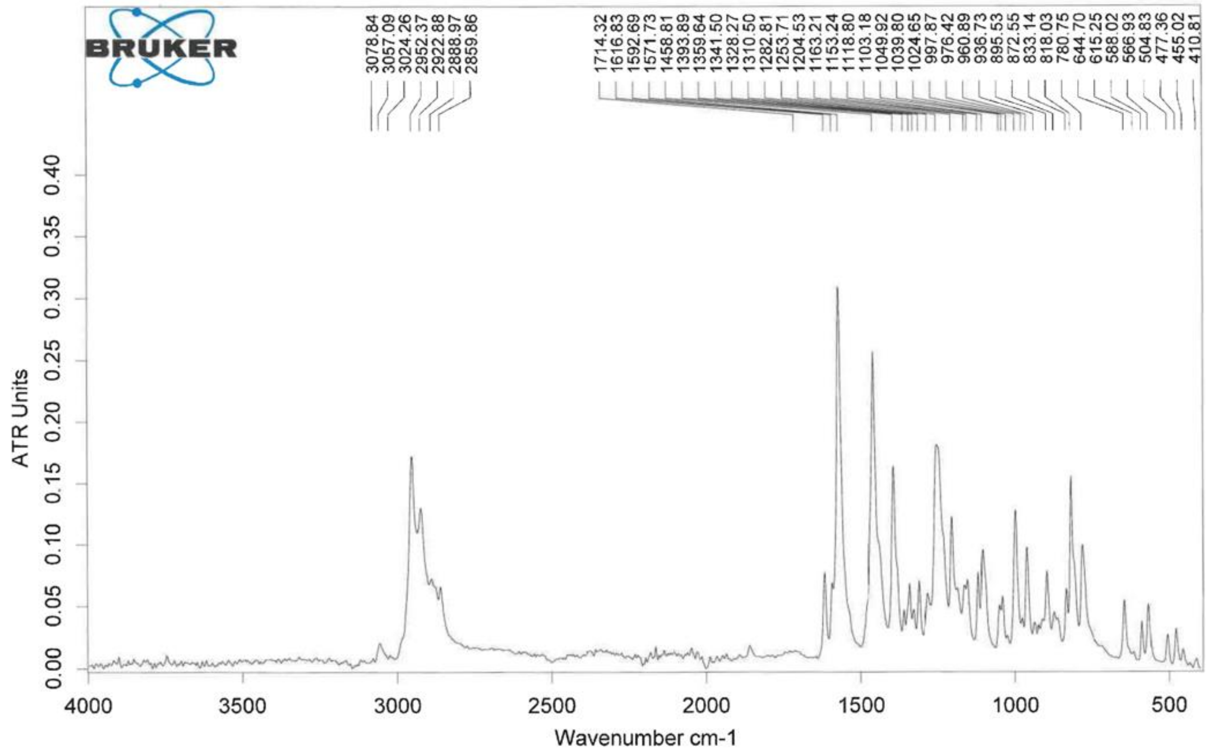

(3*R,3a*S**,6*R**,12b*R**)-3a,11-Dimethyl-2,3,3a,4,5,6-hexahydro-1*H*-3,6-methanocyclopenta[3,4]oxepino[2,3-*b*]indole (15e)**

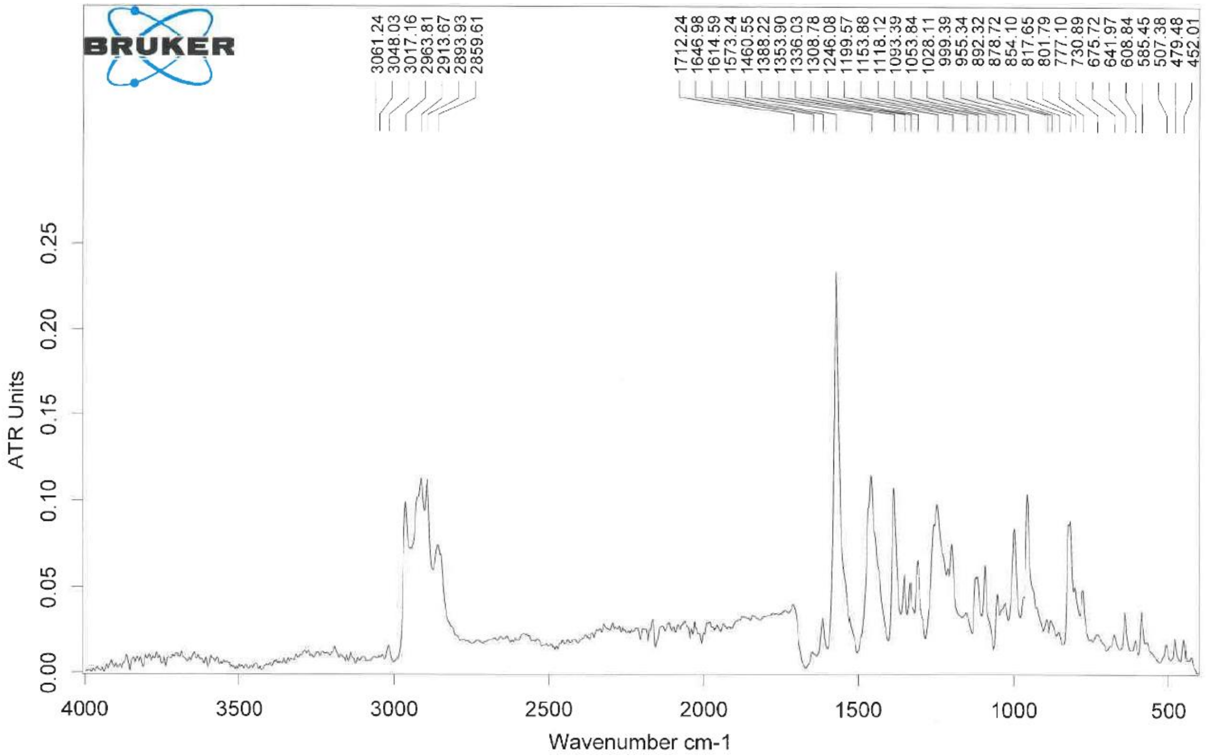

(3*R,3a*S**,6*R**,12b*R**)-11-Methoxy-3a-methyl-2,3,3a,4,5,6-hexahydro-1*H*-3,6-methanocyclopenta[3,4]oxepino[2,3-*b*]indole (15f)**

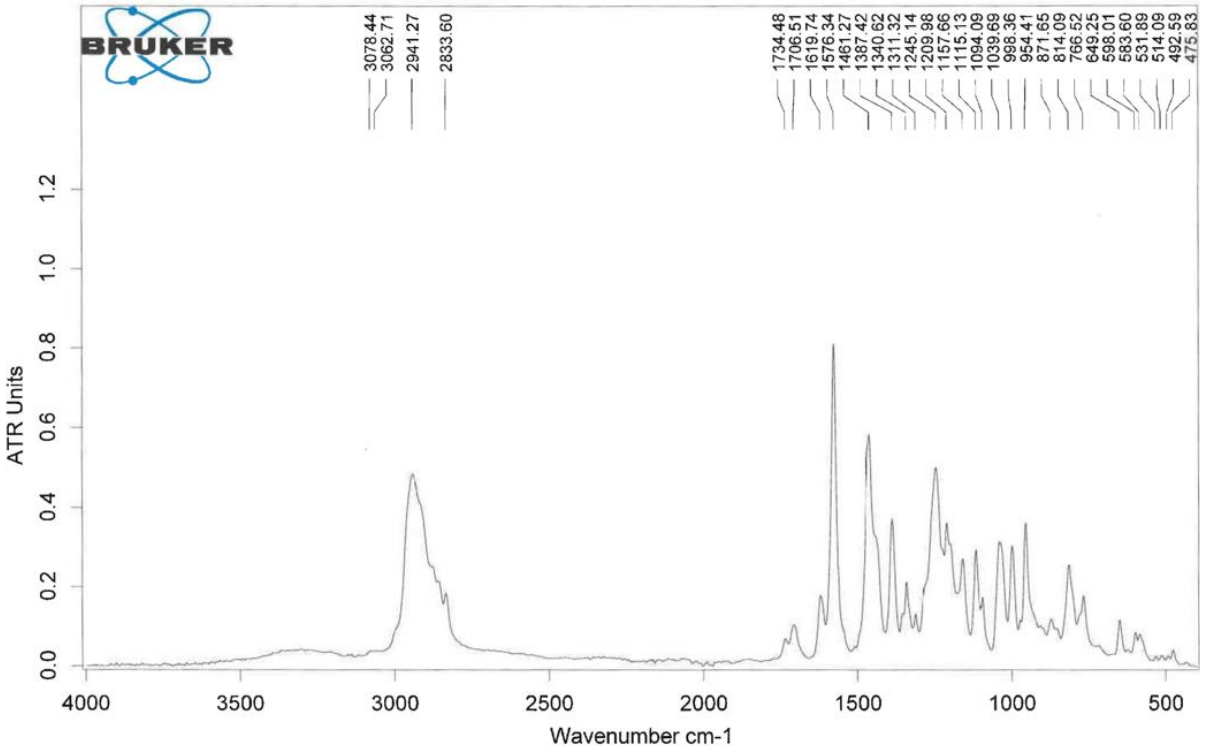

(3*R,3a*S**,6*R**,12b*R**)-3a-Methyl-11-(trifluoromethyl)-2,3,3a,4,5,6-hexahydro-1*H*-3,6-methanocyclopenta[3,4]oxepino[2,3-*b*]indole (15g)**

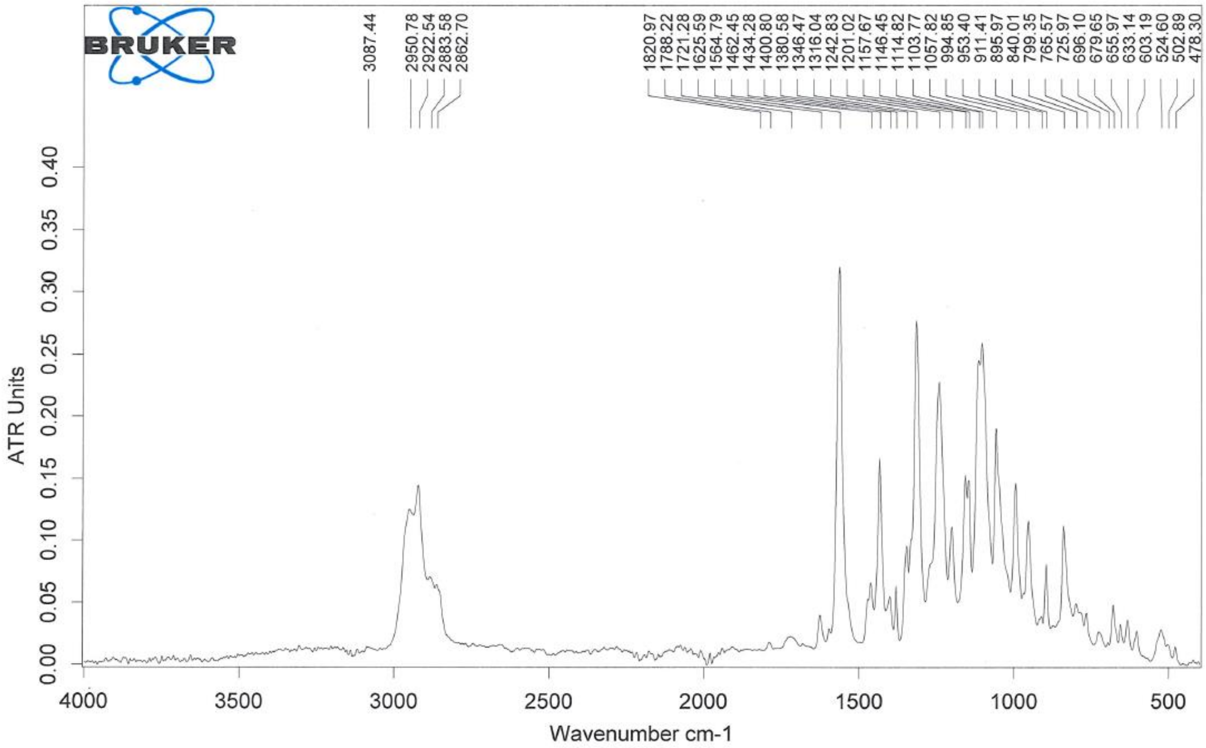

(3*R,3a*S**,6*R**,12b*R**)-9-Bromo-3a-methyl-11-(trifluoromethyl)-2,3,3a,4,5,6-hexahydro-1*H*-3,6-methanocyclopenta[3,4]oxepino[2,3-*b*]indole (15h)**

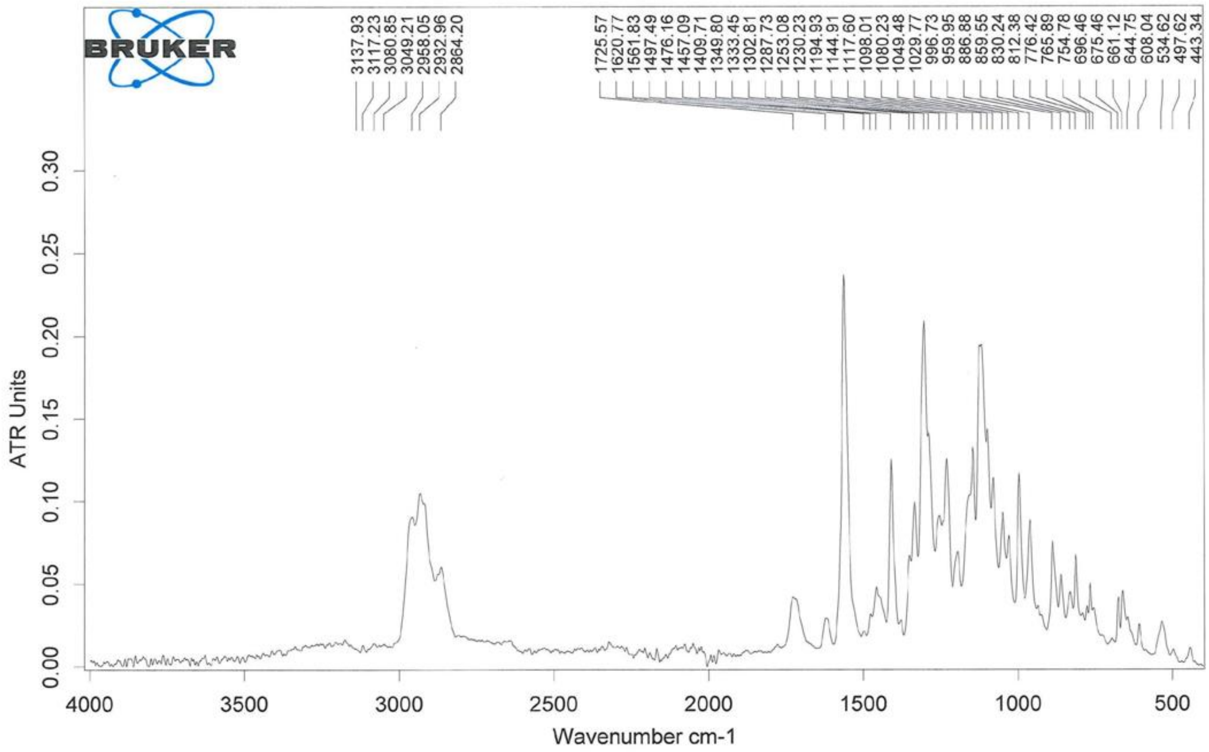

(3*R,3a*S**,6*R**,12b*R**)-11-Chloro-9-fluoro-3a-methyl-2,3,3a,4,5,6-hexahydro-1*H*-3,6-methanocyclopenta[3,4]oxepino[2,3-*b*]indole (15i)**

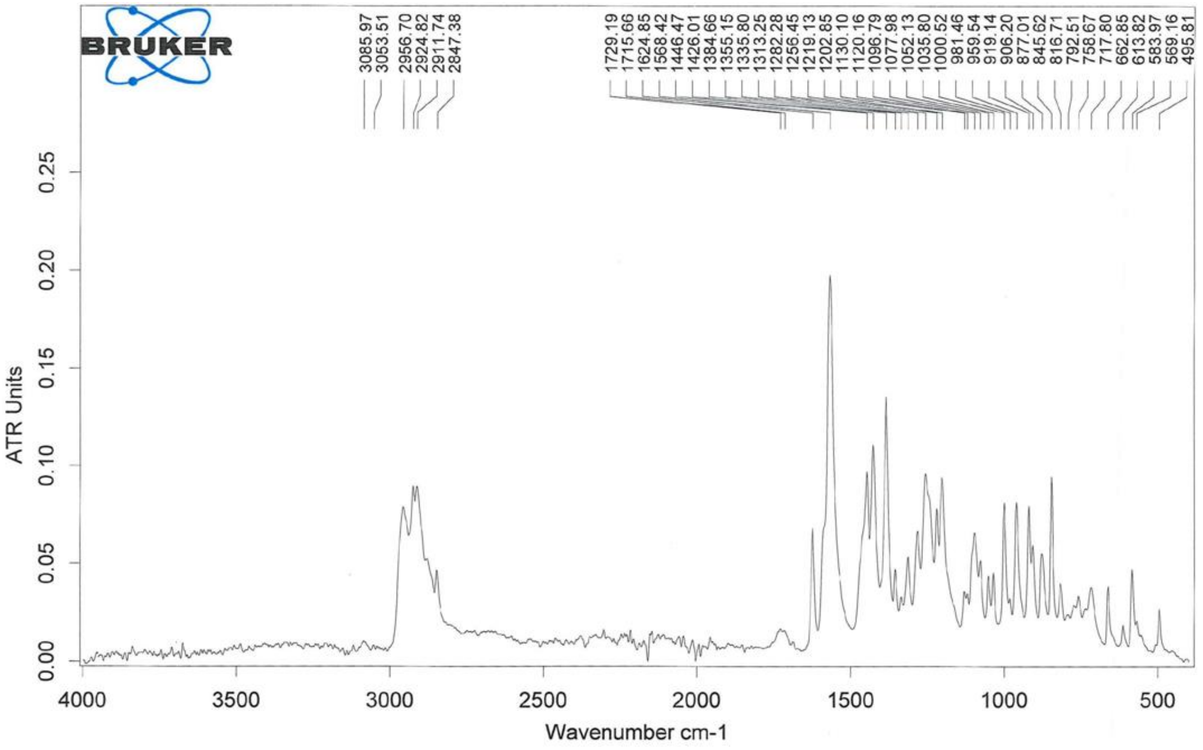

LC-LRMS traces

(1*R**,4a*R**,6*R**,8a*S**)-8a-Methyl-3,4,4a,5,6,7,8,8a-octahydro-2*H*,5'*H*-spiro[naphthalene-1,4'-pyrrolo[1,2-*a*]quinoxalin]-6-ol (4’)

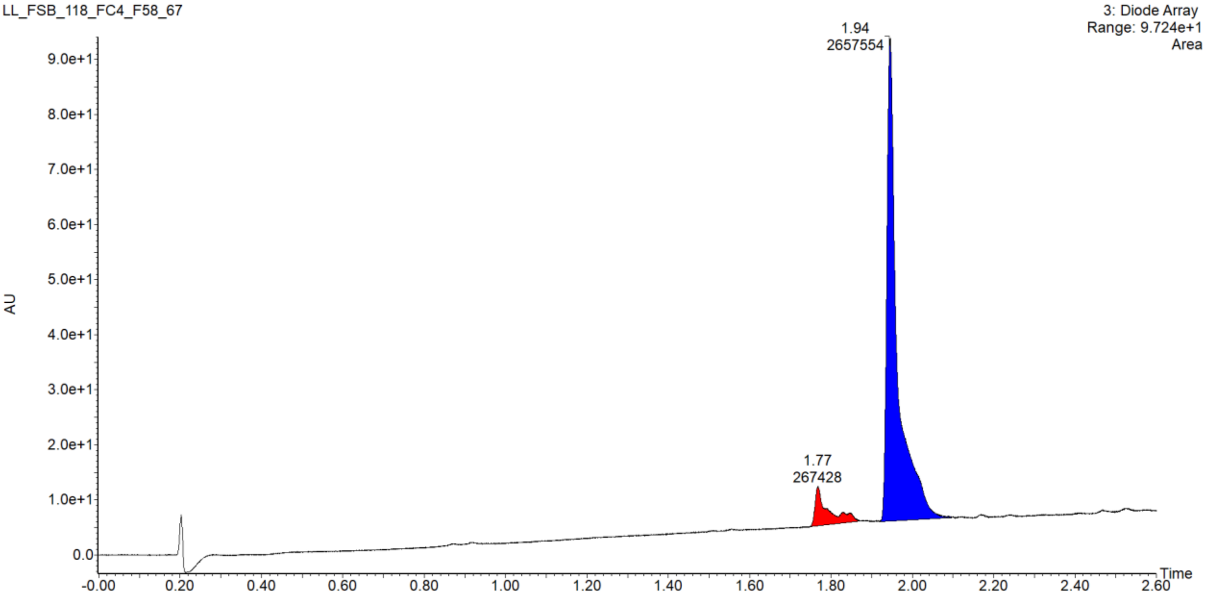

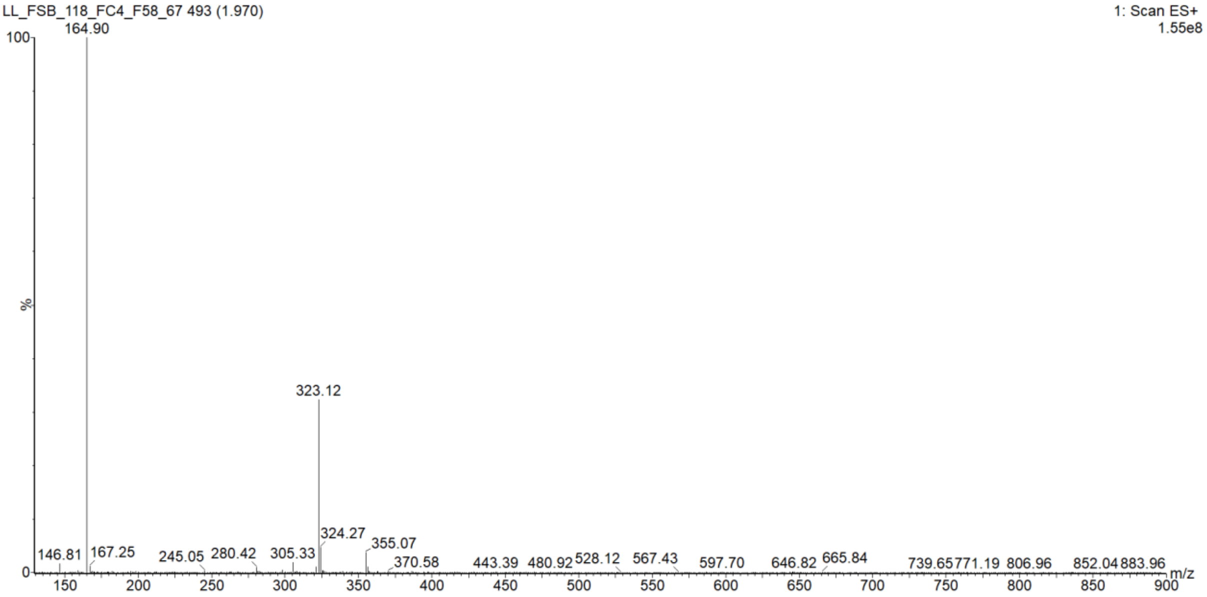

Retention time (min)	*m/z* found	Calcd. for [M+H]$^+$	Area	Rel. area (%)
1.75 – 1.87	*several*	-	267428	9
1.92 – 2.11	323.12	323.21	2657554	91

(1*R,4a*R**,6*R**,8a*S**)-6-Hydroxy-8a-methyl-3,4,4a,5,6,7,8,8a-octahydro-1'*H*,2*H*-spiro[naphthalene-1,2'-quinazolin]-4'(3'*H*)-one (5a')**

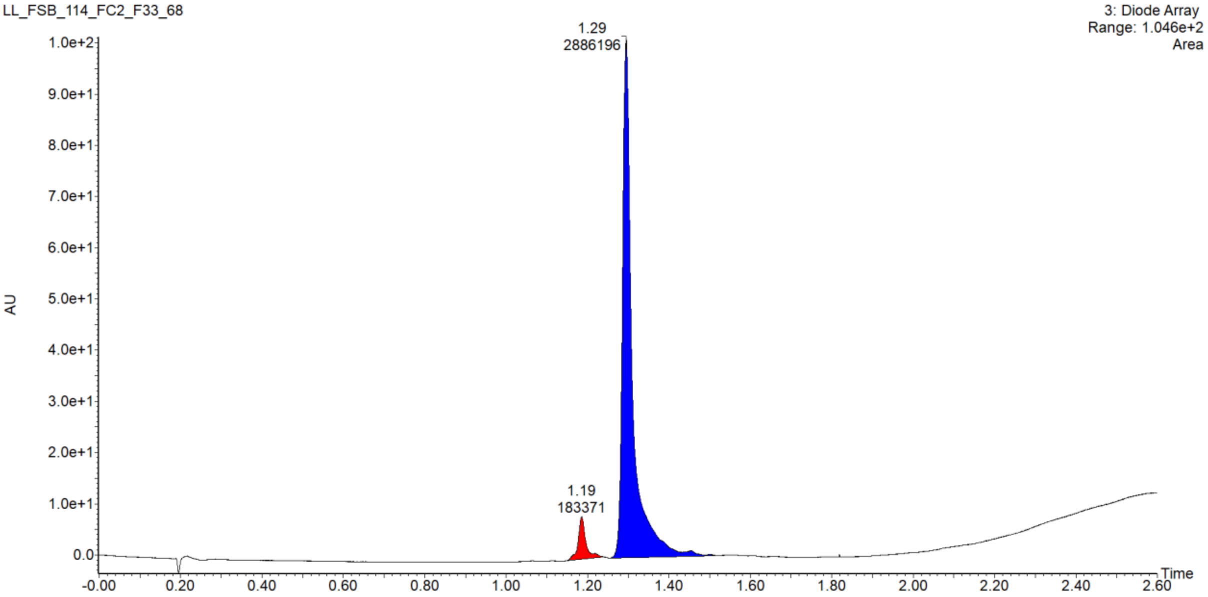

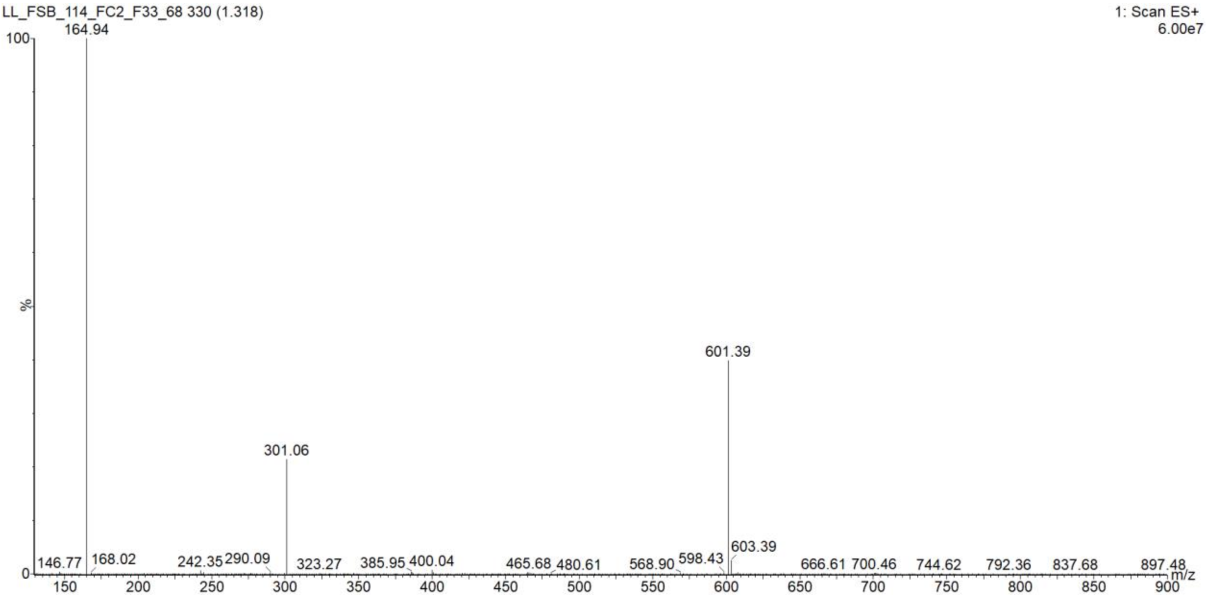

Retention time (min)	*m*/*z* found	Calcd. for [M+H]$^+$	Area	Rel. area (%)
1.15 – 1.24	301.10	-	183371	6
1.25 – 1.52	301.06	301.19	2886196	94

(1*R,4a*R**,6*R**,8a*S**)-7'-Fluoro-6-hydroxy-8a-methyl-3,4,4a,5,6,7,8,8a-octahydro-1'*H*,2*H*-spiro[naphthalene-1,2'-quinazolin]-4'(3'*H*)-one (5b')**

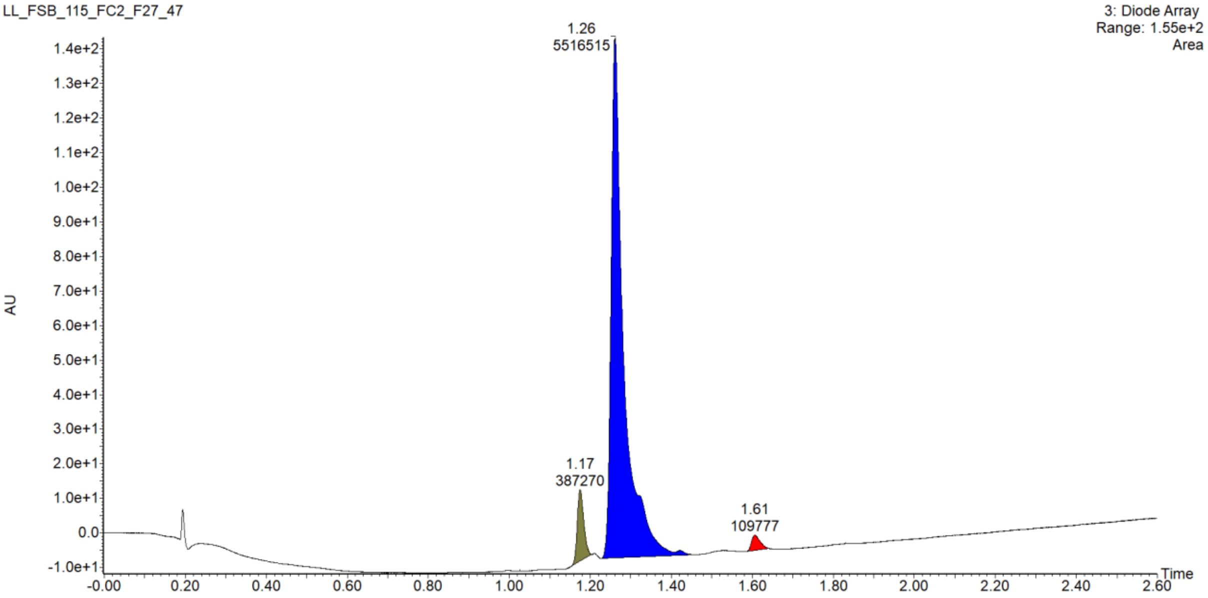

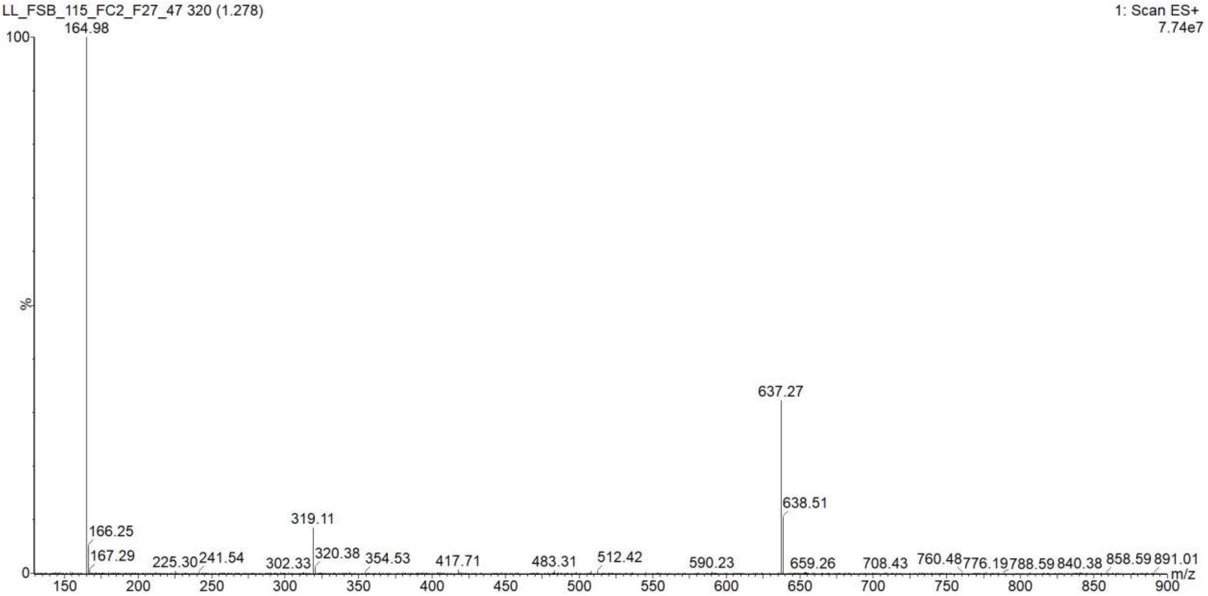

Retention time (min)	*m/z* found	Calcd. for [M+H]+	Area	Rel. area (%)
1.14 – 1.21	319.04	-	387270	6
1.23 – 1.46	319.11	319.18	5516515	92
1.59 – 1.64	601.16	-	108777	2

(1*R,4a*R**,6*R**,8a*S**)-6-Hydroxy-6'-methoxy-8a-methyl-3,4,4a,5,6,7,8,8a-octahydro-1'*H*,2*H*-spiro[naphthalene-1,2'-quinazolin]-4'(3'*H*)-one (5c')**

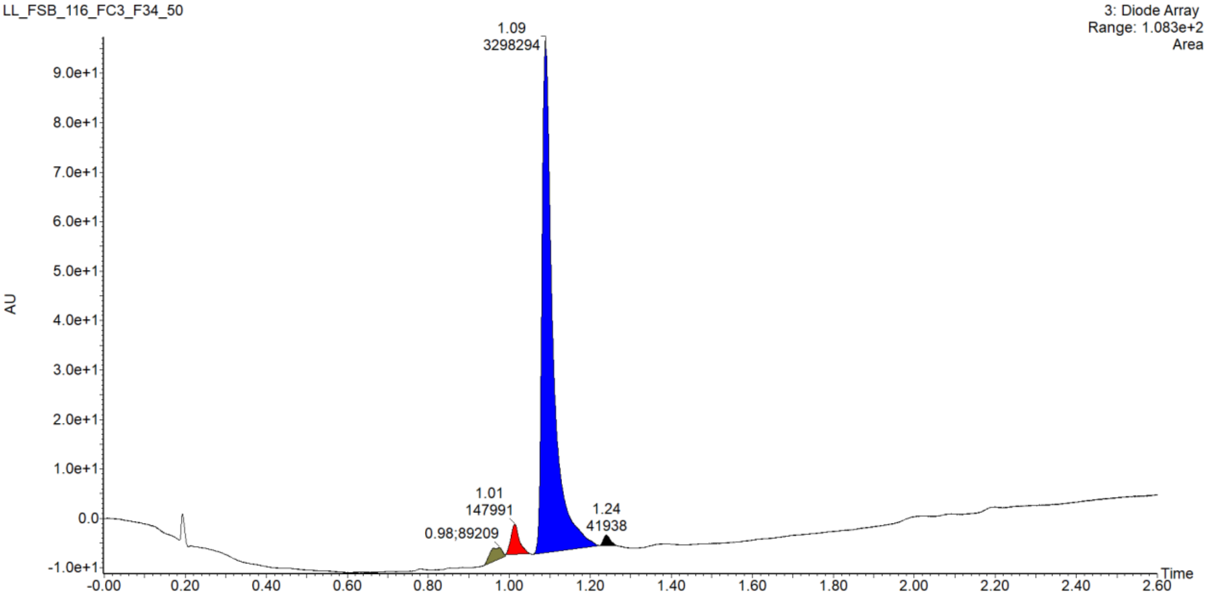

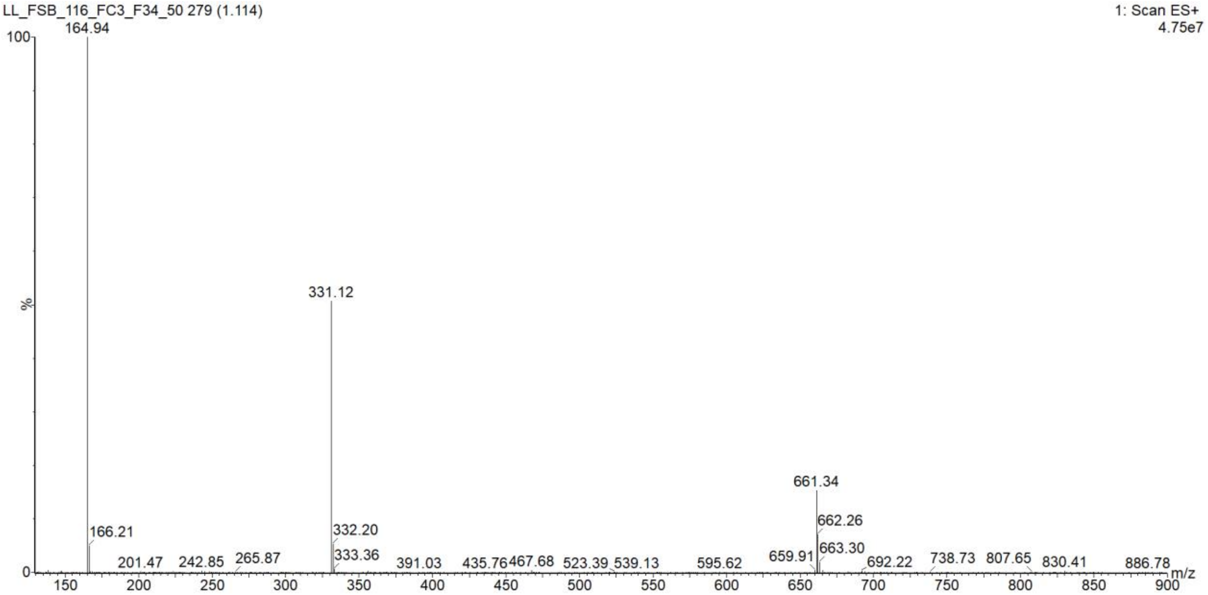

Retention time (min)	*m/z* found	Calcd. for [M+H]$^+$	Area	Rel. area (%)
0.93 – 0.99	363.58	-	89209	3
0.99 – 1.05	331.12	-	147991	4
1.06 – 1.22	331.12	331.20	3298294	92
1.22 – 1.27	331.12	-	41938	1

(5a*R,7*R**,9a*S**)-2-(4-Fluorophenyl)-9a-methyl-4,5,5a,6,7,8,9,9a-octahydronaphtho[1,2-*d*]thiazol-7-ol (8e)**

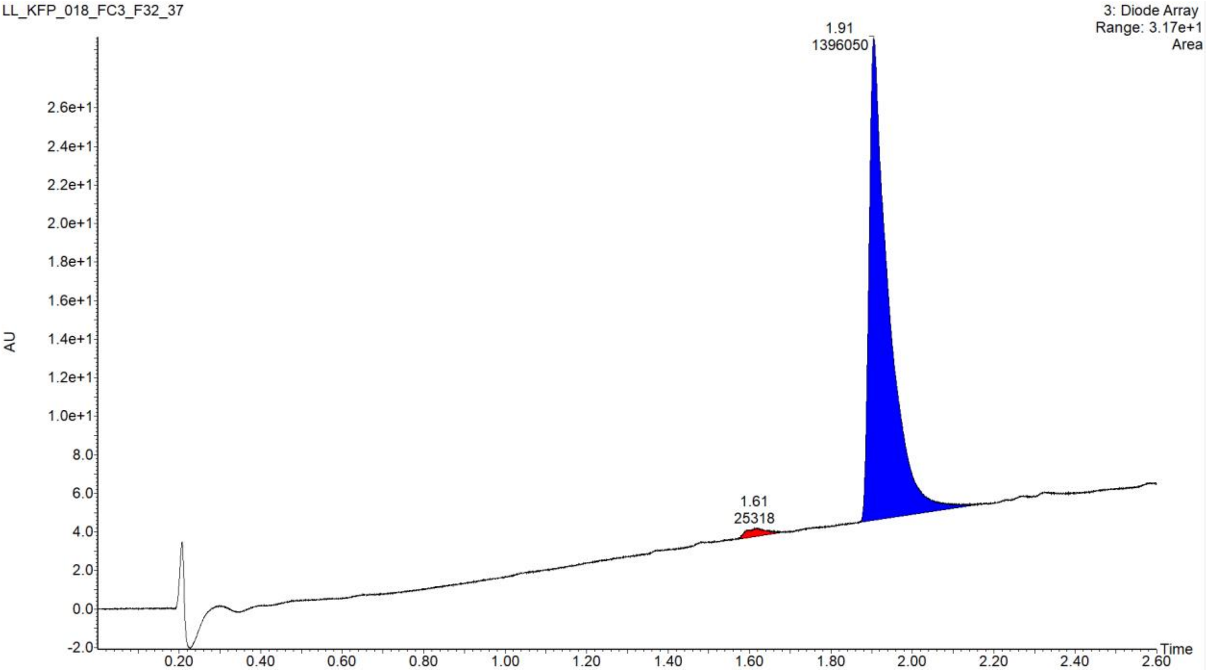

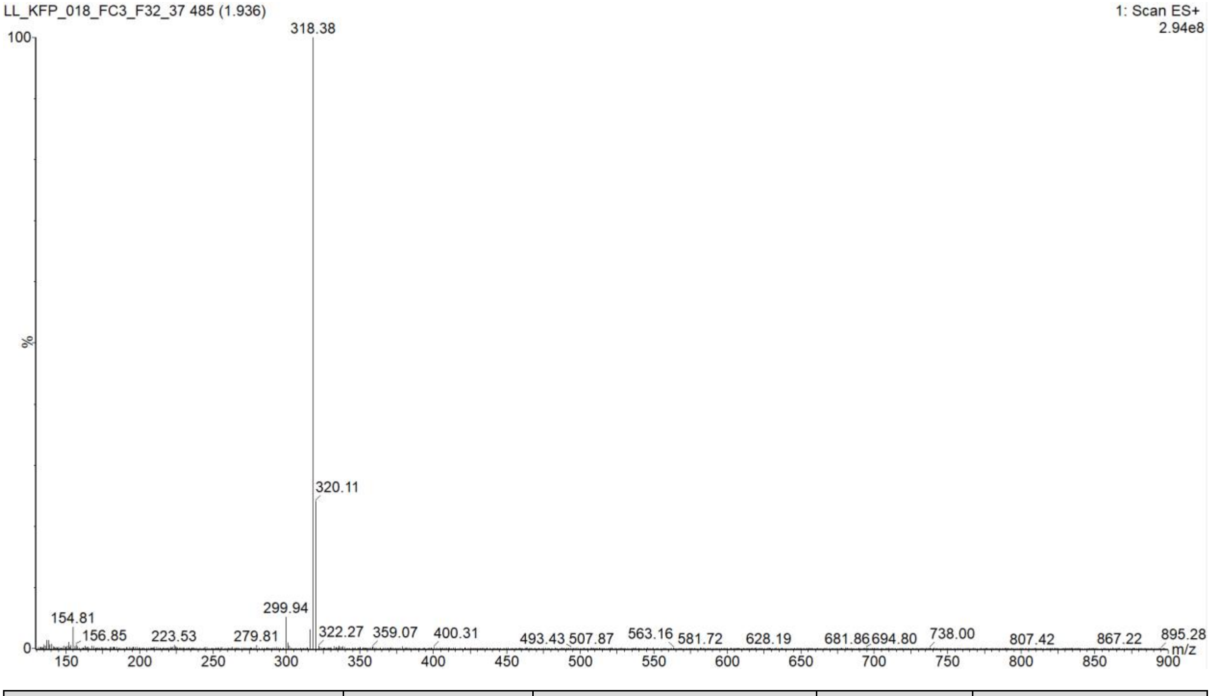

Retention time (min)	*m/z* found	Calcd. for $[M+H]^+$	Area	Rel. area (%)
1.57 – 1.68	332.16	-	25318	2
1.87 – 2.18	318.38	318.13	1396050	98

Ethyl (3*R,4a*R**,11b*S**)-3-hydroxy-11b-methyl-2,3,4,4a,5,6,11,11b-octahydro-1*H*-benzo[*a*]carbazole-8-carboxylate (10k)**

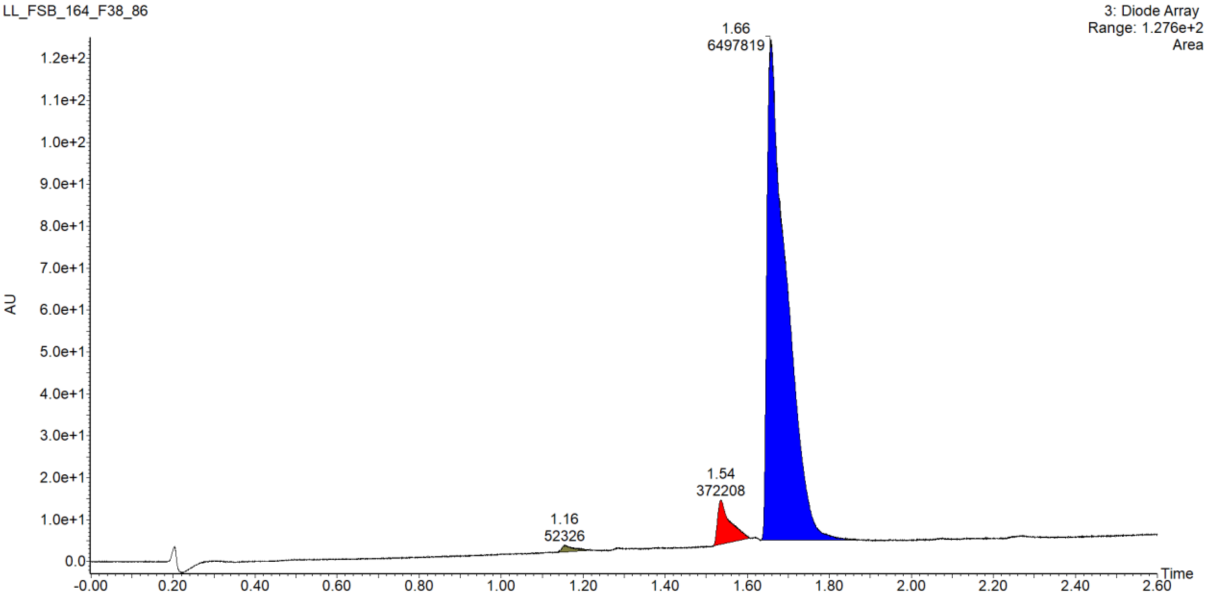

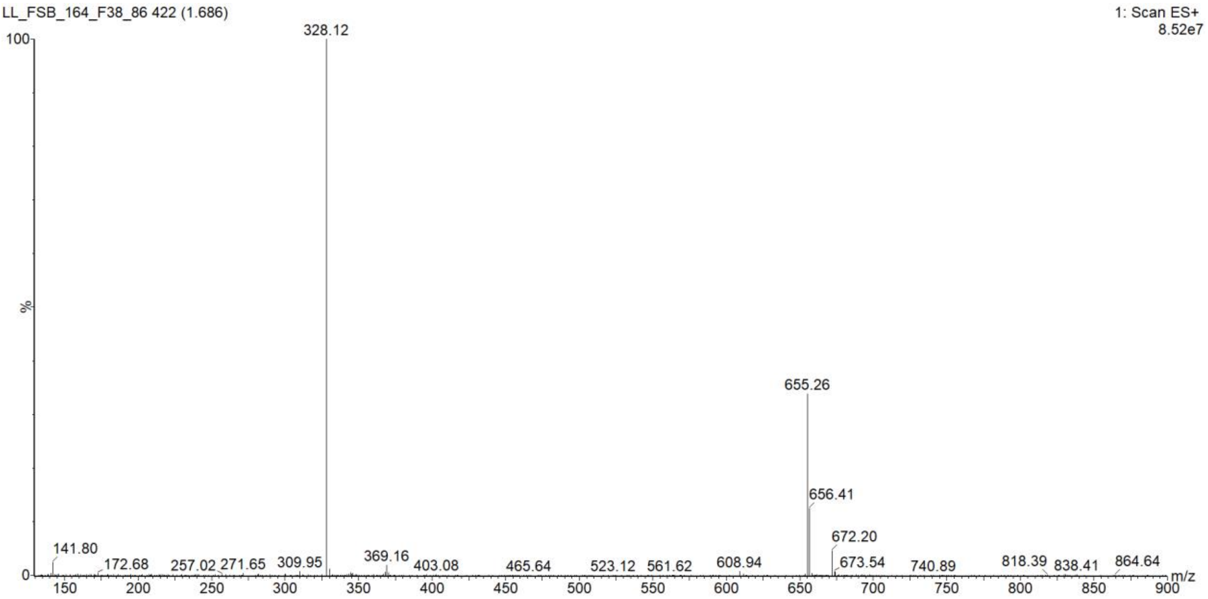

Retention time (min)	*m/z* found	Calcd. for [M+H]$^+$	Area	Rel. area (%)
1.14 – 1.21	233.88	-	52326	1
1.51 – 1.61	314.11	314.18 (methyl ester)	372208	5
1.63 – 1.85	328.11	328.19	6497819	94

(1*R,3a*R**,5*R**,7a*S**)-5-Hydroxy-5’,7a-dimethyl-2,3,3a,4,5,6,7,7a-octahydrospiro[indene-1,2’-indolin]-3’-one (13)**

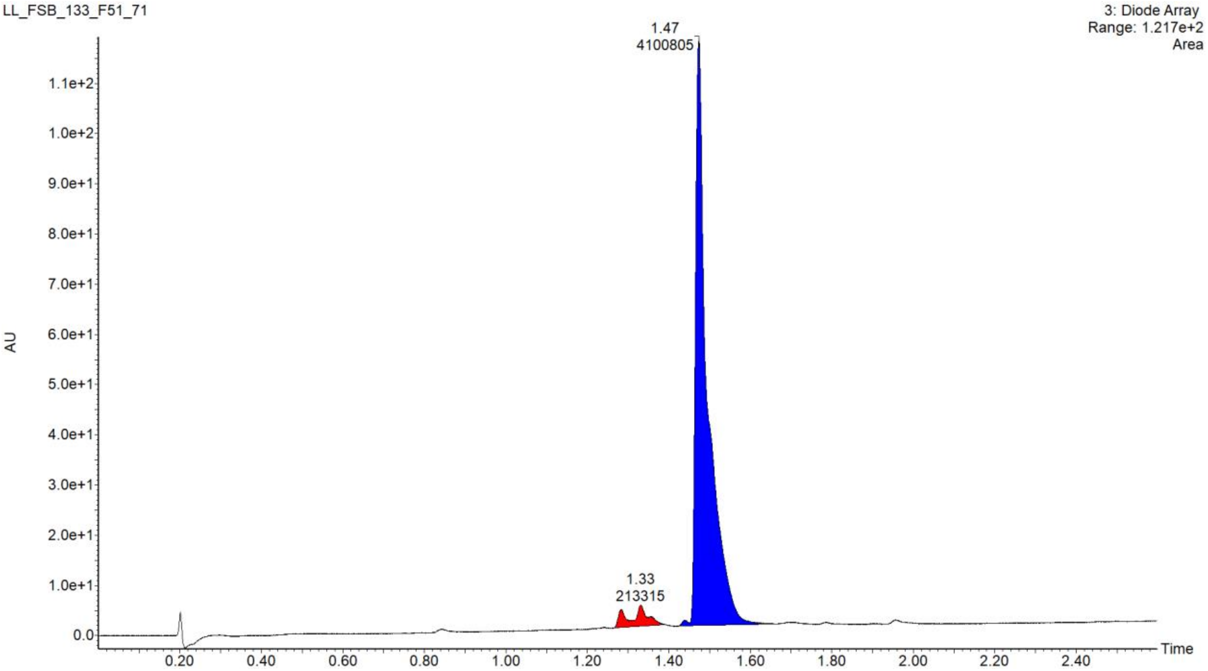

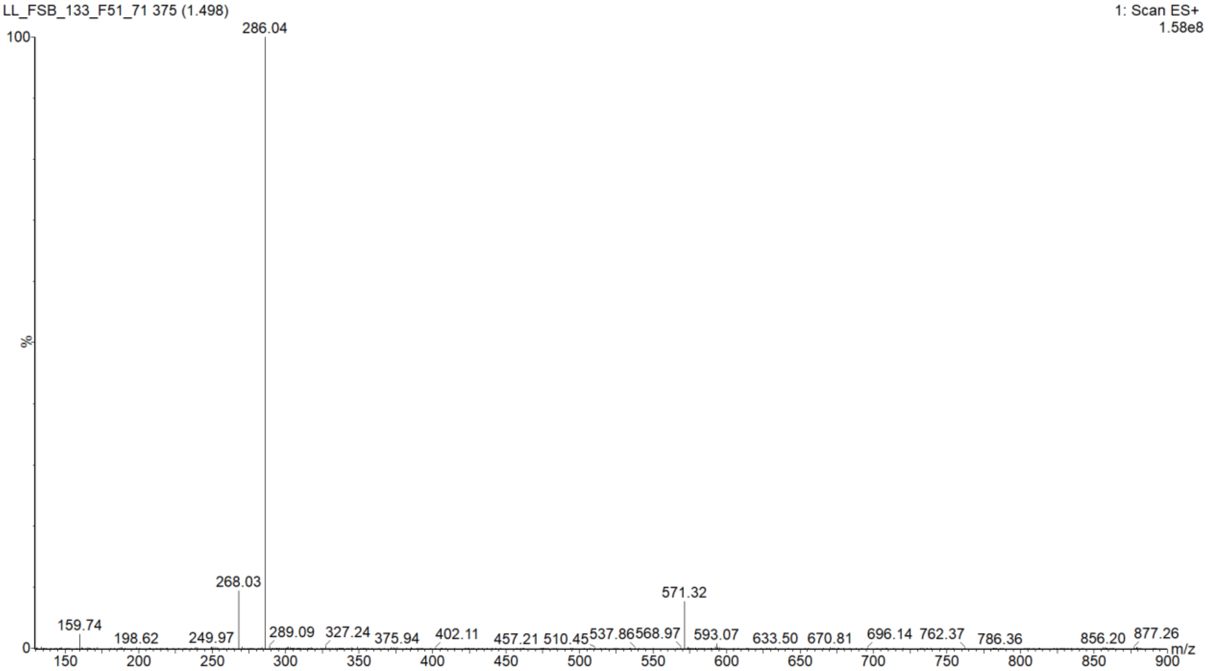

Retention time (min)	*m*/*z* found	Calcd. for $[M+H]^+$	Area	Rel. area (%)
1.26 – 1.39	*several*	-	213315	5
1.42 – 1.66	286.04	286.18	4100805	95

(3*R,3a*S**,6*R**,12b*R**)-11-Bromo-3a-methyl-2,3,3a,4,5,6-hexahydro-1*H*-3,6-methanocyclopenta[3,4]oxepino[2,3-*b*]indole (15b)**

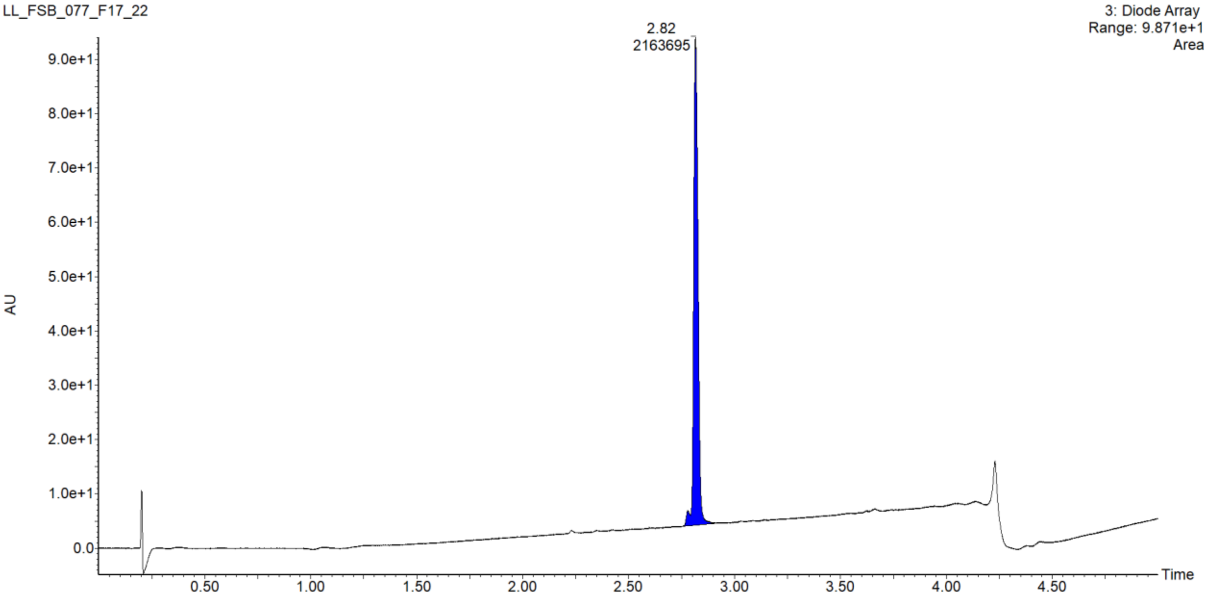

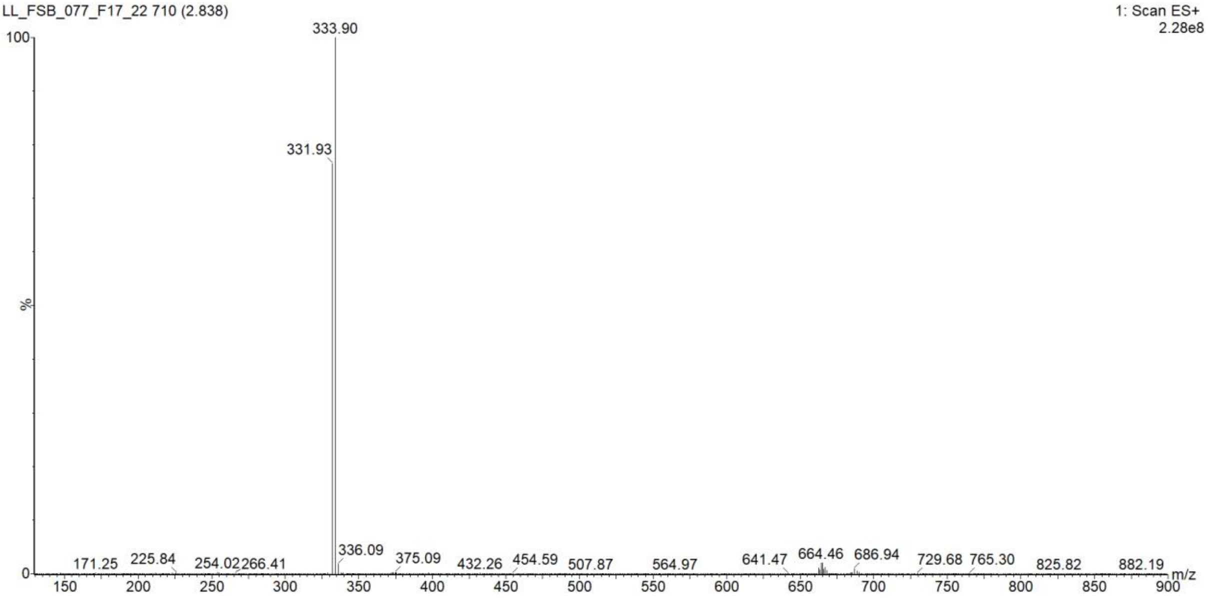

Retention time (min)	*m/z* found	Calcd. for $[M+H]^+$	Area	Rel. area (%)
2.75 – 2.92	331.93	332.06	2163695	100

(3*R,3a*S**,6*R**,12b*R**)-3a-Methyl-11-(trifluoromethyl)-2,3,3a,4,5,6-hexahydro-1*H*-3,6-methanocyclopenta[3,4]oxepino[2,3-*b*]indole (15g)**

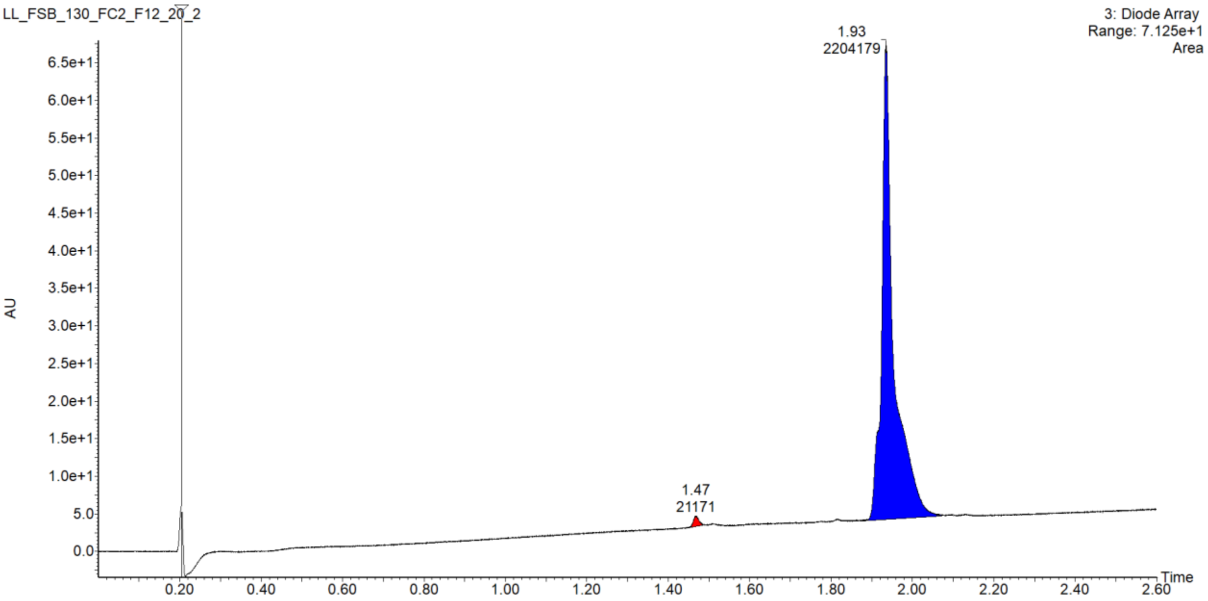

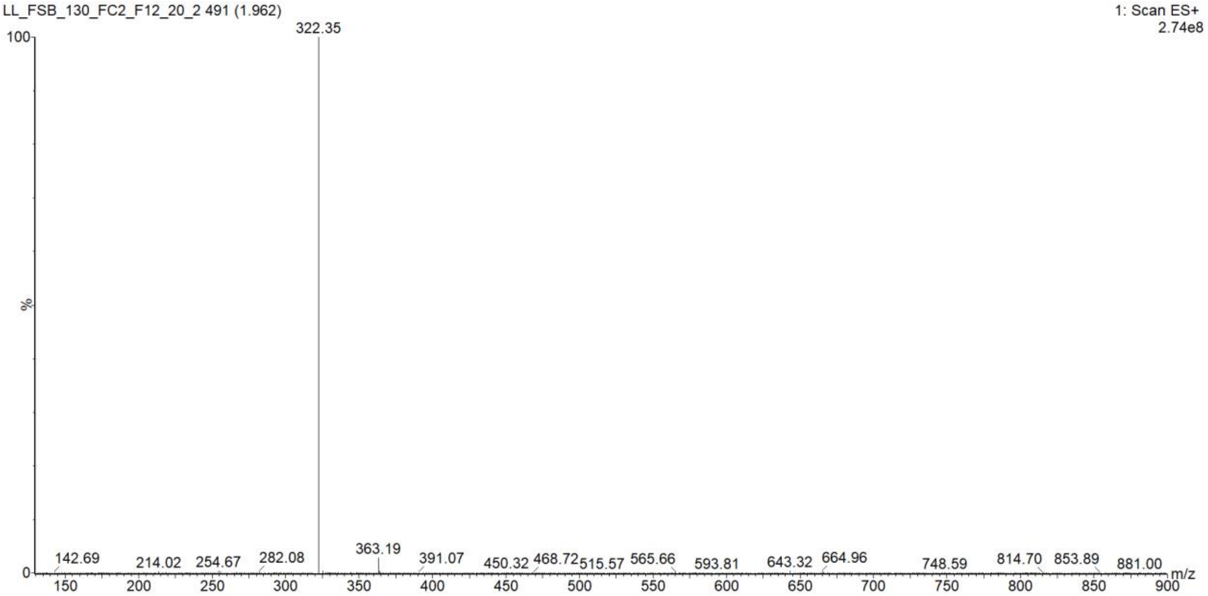

Retention time (min)	*m*/*z* found	Calcd. for [M+H]$^+$	Area	Rel. area (%)
1.45 – 1.49	282.12	-	21171	1
1.87 – 2.07	322.35	322.14	2204179	99

(3*R,3a*S**,6*R**,12b*R**)-9-Bromo-3a-methyl-11-(trifluoromethyl)-2,3,3a,4,5,6-hexahydro-1*H*-3,6-methanocyclopenta[3,4]oxepino[2,3-*b*]indole (15h)**

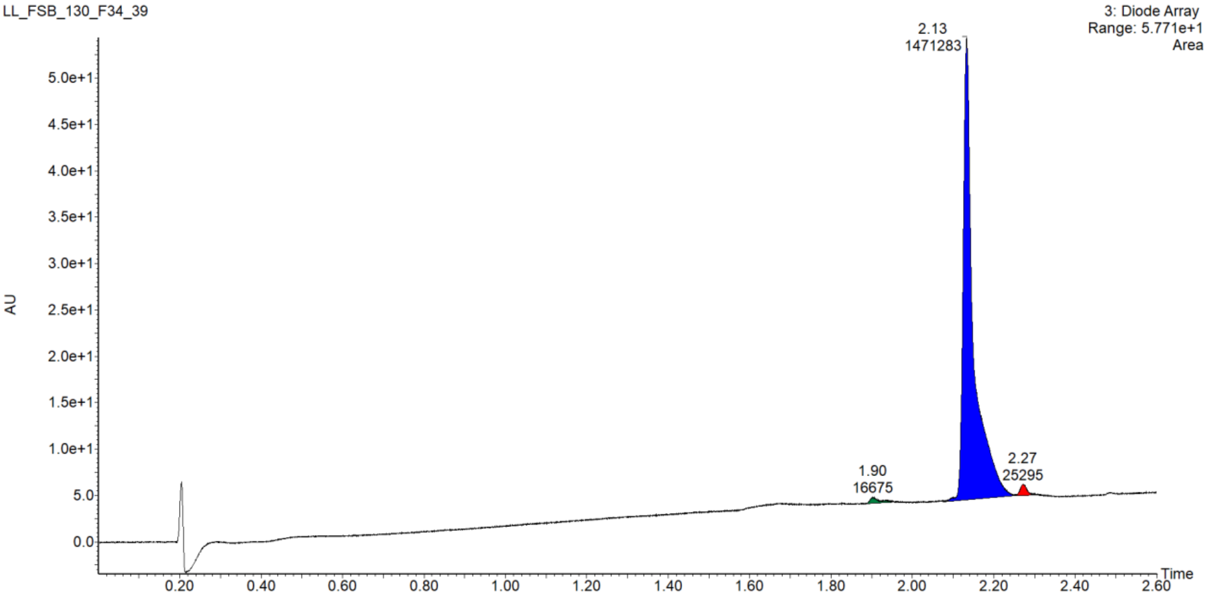

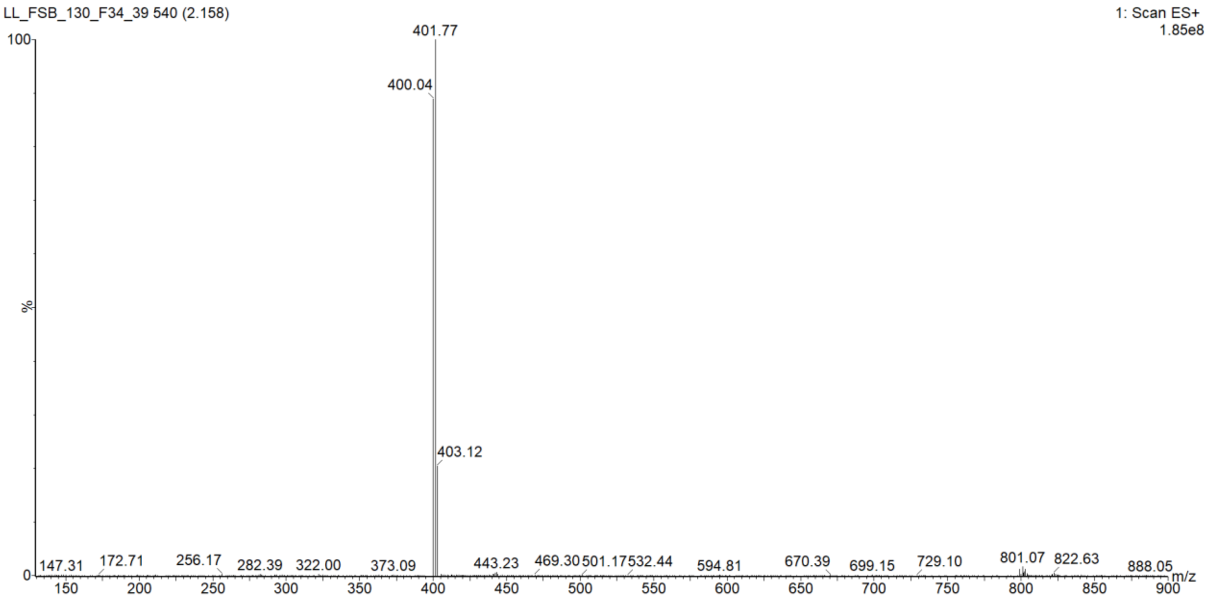

Retention time (min)	*m/z* found	Calcd. for [M+H]$^+$	Area	Rel. area (%)
1.88 – 1.96	332.08	322.14 (**15g**)	16675	1
2.08 – 2.25	400.04	400.05	1471283	97
2.25 – 3.32	399.96	400.05<br>(probably regioisomer of **15h**)	25295	2

(3*R,3a*S**,6*R**,12b*R**)-3a-Methyl-11-morpholino-2,3,3a,4,5,6-hexahydro-1*H*-3,6-methanocyclopenta[3,4]oxepino[2,3-*b*]indole, (±)-asteroxin-1 (19a)**

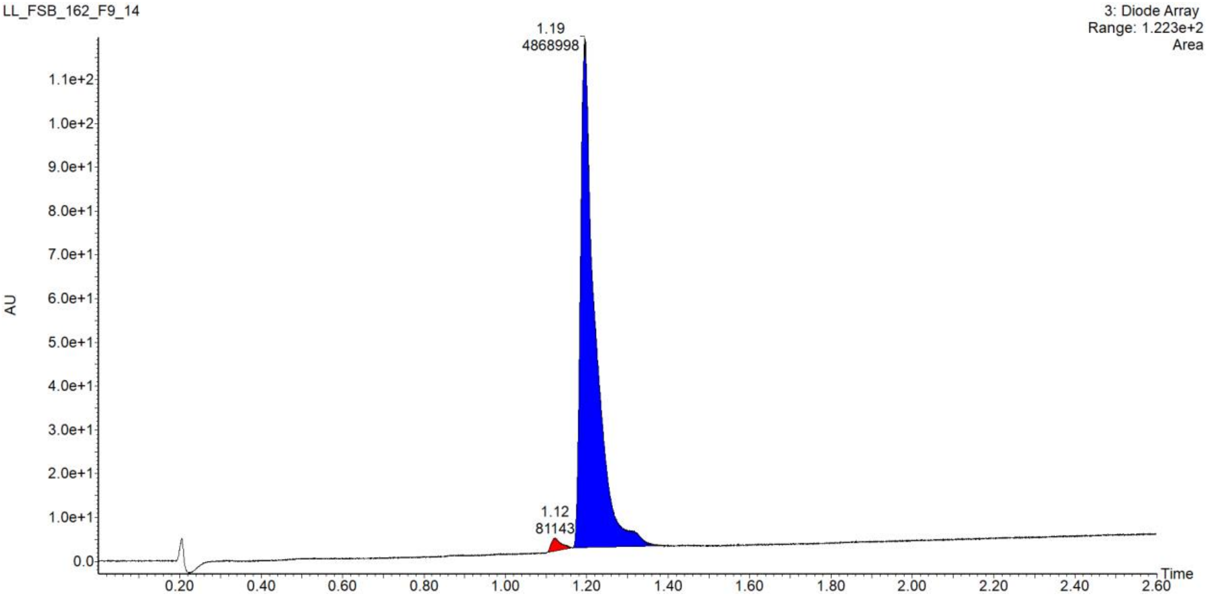

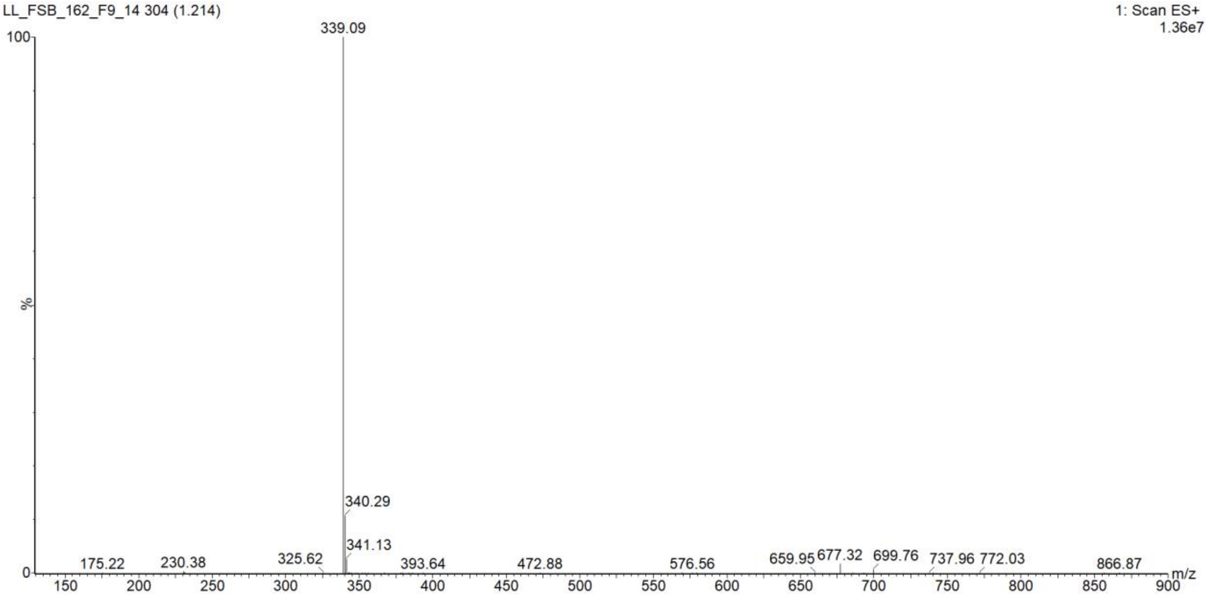

Retention time (min)	*m*/*z* found	Calcd. for $[M+H]^+$	Area	Rel. area (%)
1.01 – 1.16	339.17	-	81143	2
1.17 – 1.38	339.09	339.21	4868998	98

(3*R,3a*S**,6*R**,12b*R**)-3a-Methyl-11-(piperazin-1-yl)-2,3,3a,4,5,6-hexahydro-1*H*-3,6-methanocyclopenta[3,4]oxepino[2,3-*b*]indole (22)**

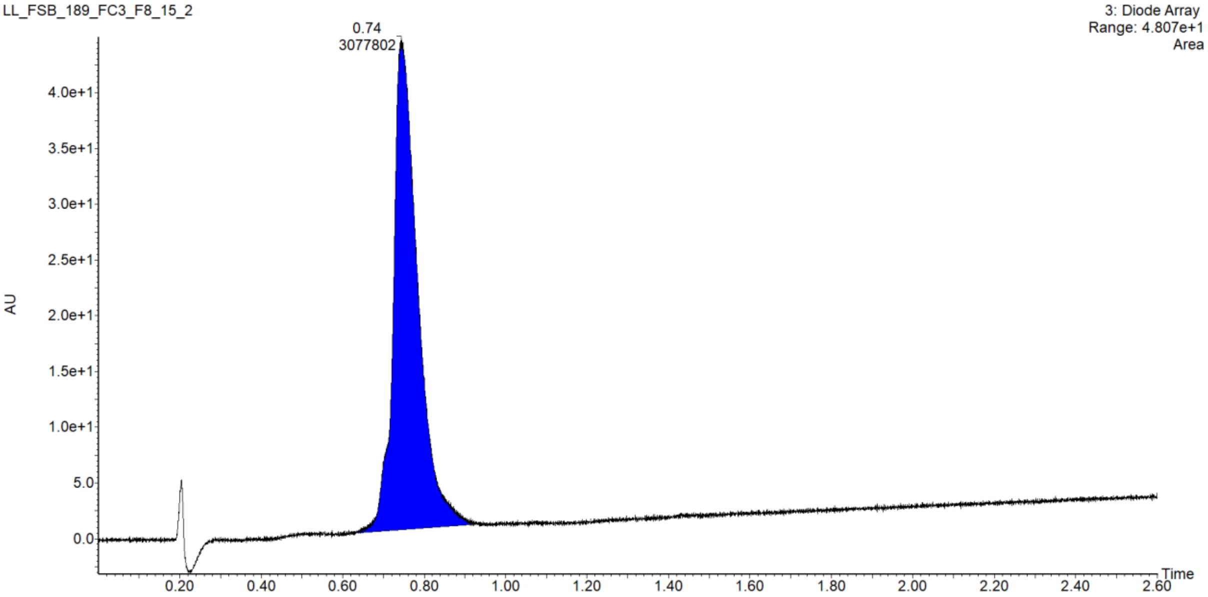

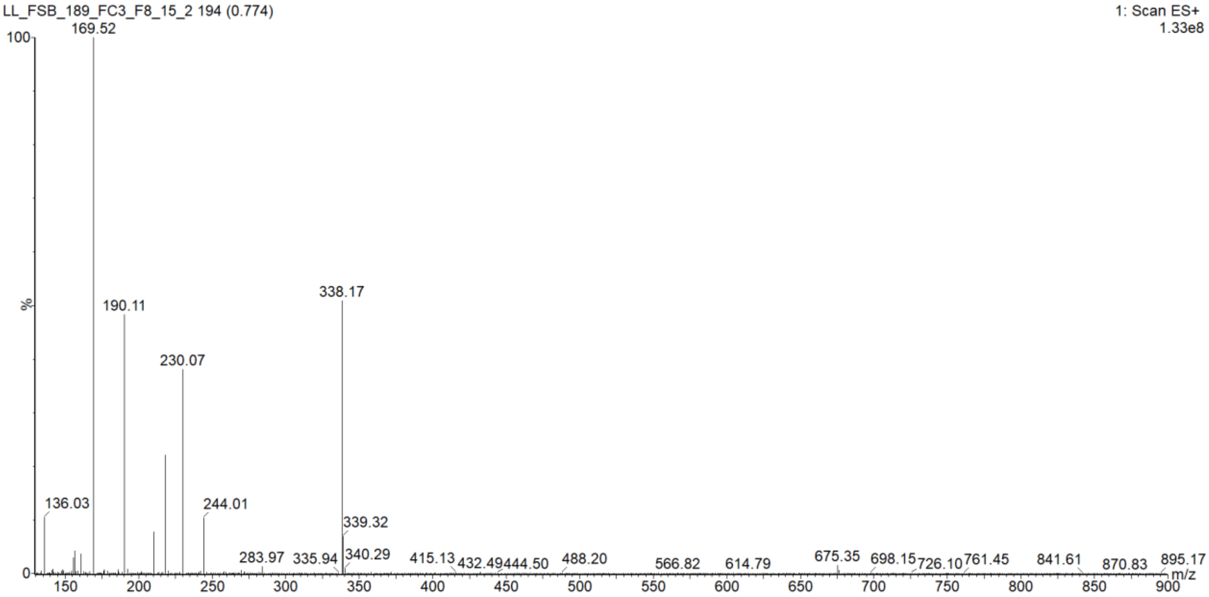

Retention time (min)	*m*/*z* found	Calcd. for [M+H]$^+$	Area	Rel. area (%)
0.60 – 0.94	338.17	338.22	3077802	100

(3*R*,3a*S*,6*R*,12b*R*)-11-Bromo-3a-methyl-2,3,3a,4,5,6-hexahydro-1*H*-3,6-methanocyclopenta[3,4]oxepino[2,3-*b*]indole ((+)-15b)

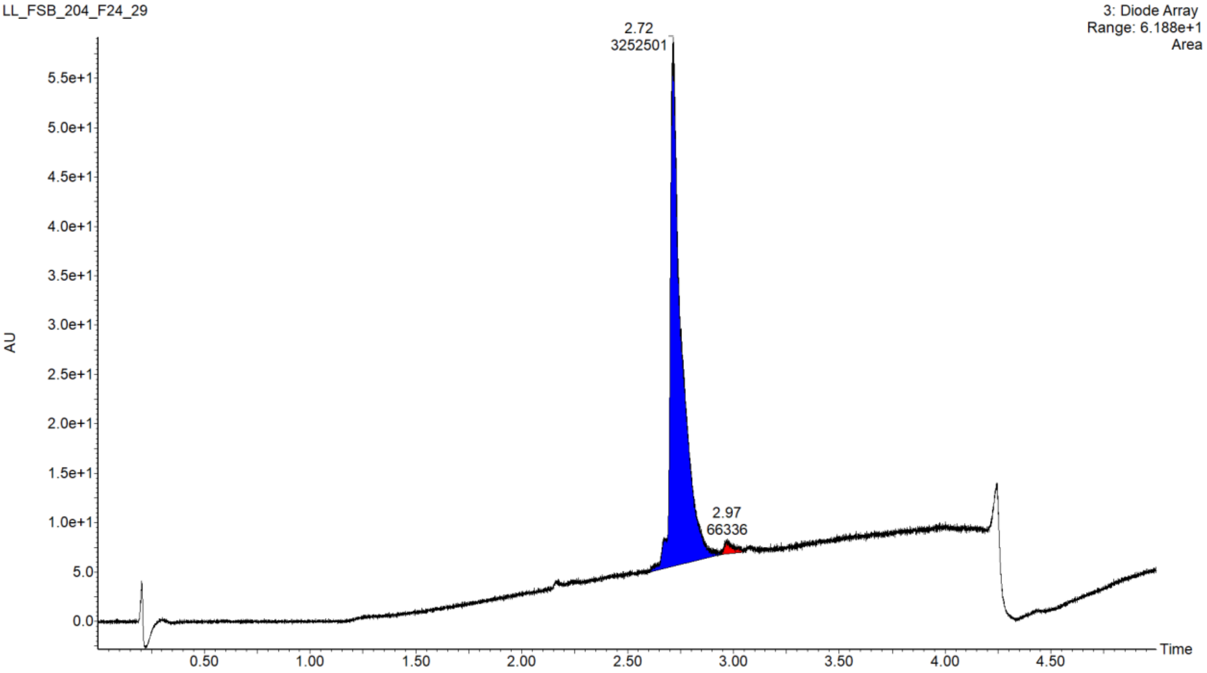

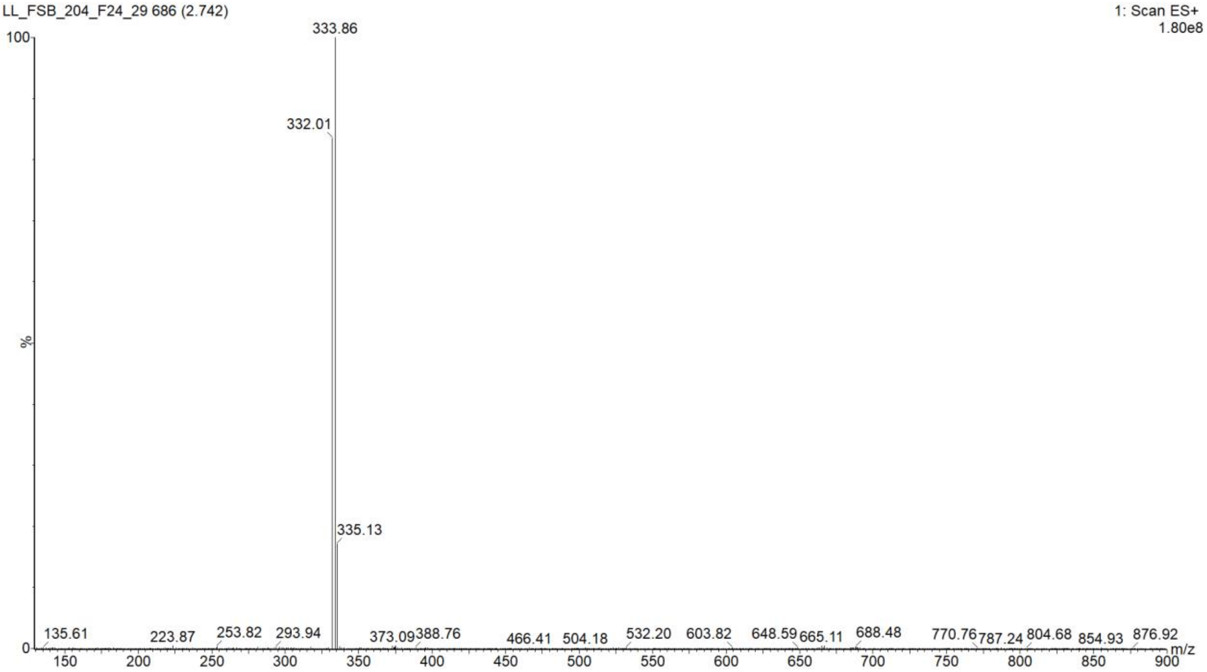

Retention time (min)	*m*/*z* found	Calcd. for $[M+H]^+$	Area	Rel. area (%)
2.59 – 2.94	332.01	332.06	3252501	98
2.94 – 3.05	282.16	-	66336	2

(3*S*,3a*R*,6*S*,12b*S*)-11-Bromo-3a-methyl-2,3,3a,4,5,6-hexahydro-1*H*-3,6-methanocyclopenta[3,4]oxepino[2,3-*b*]indole ((–)-15b)

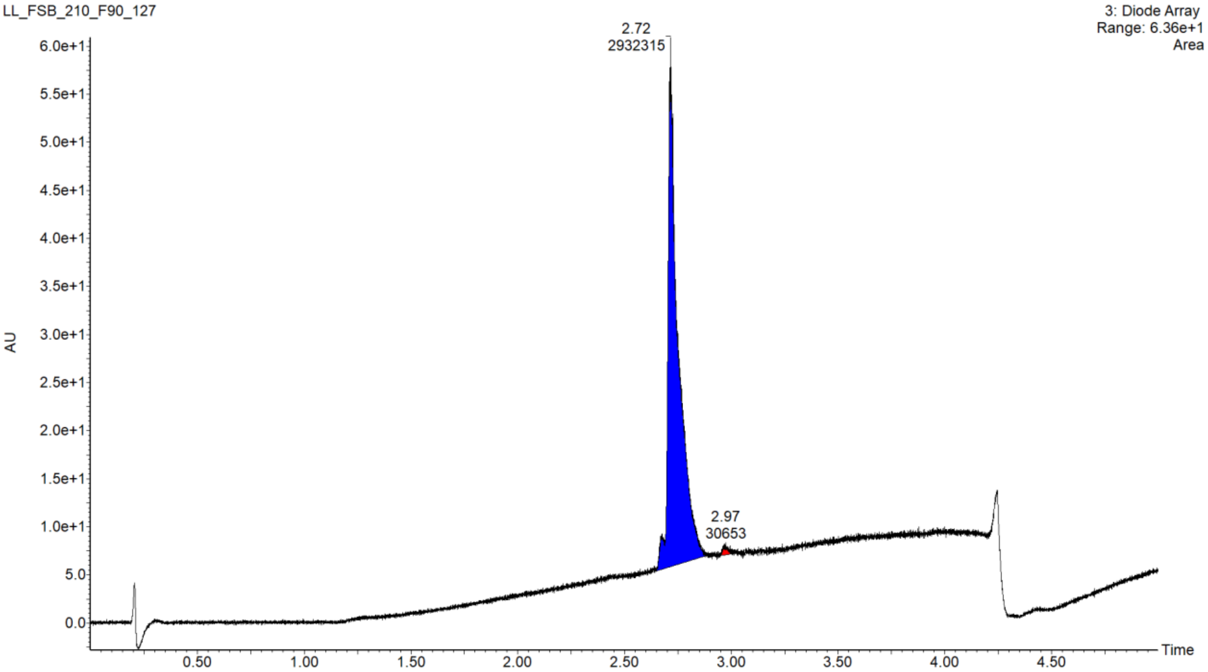

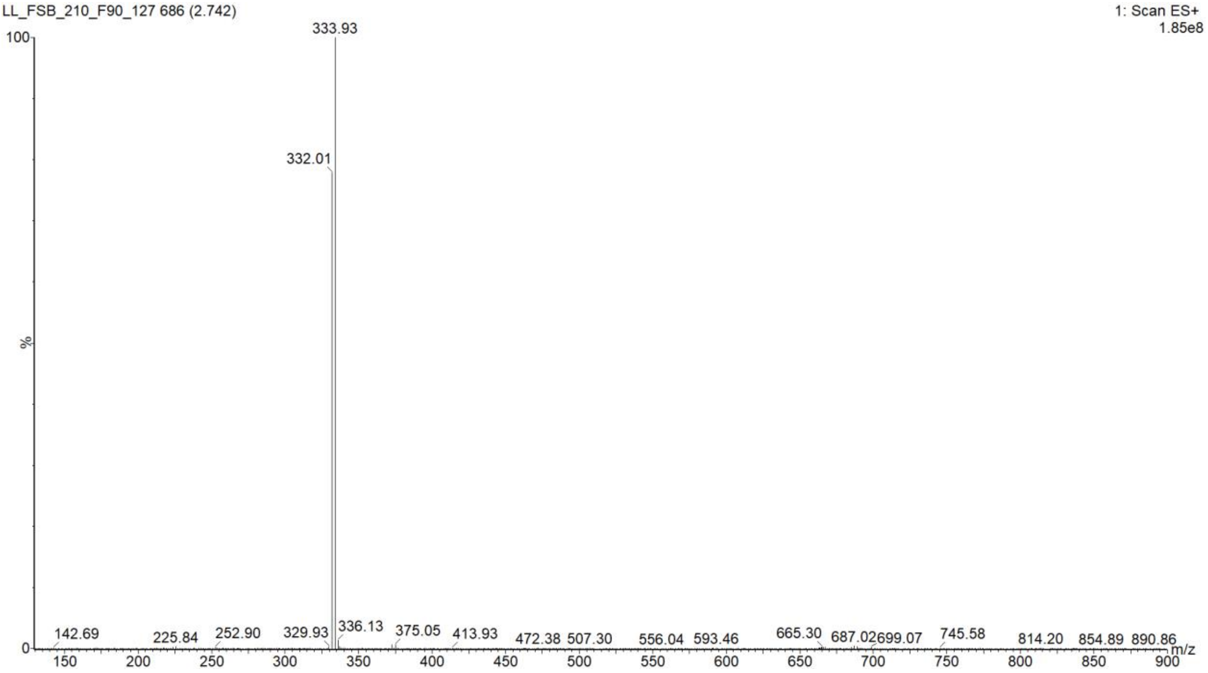

Retention time (min)	*m/z* found	Calcd. for [M+H]$^+$	Area	Rel. area (%)
2.65 – 2.88	332.01	332.06	2932315	99
2.93 – 3.00	282.16	-	30653	1

(3*R*,3a*S*,6*R*,12b*R*)-3a-Methyl-11-morpholino-2,3,3a,4,5,6-hexahydro-1*H*-3,6-methanocyclopenta[3,4]oxepino[2,3-*b*]indole, (+)-asteroxin-1 ((+)-19a)

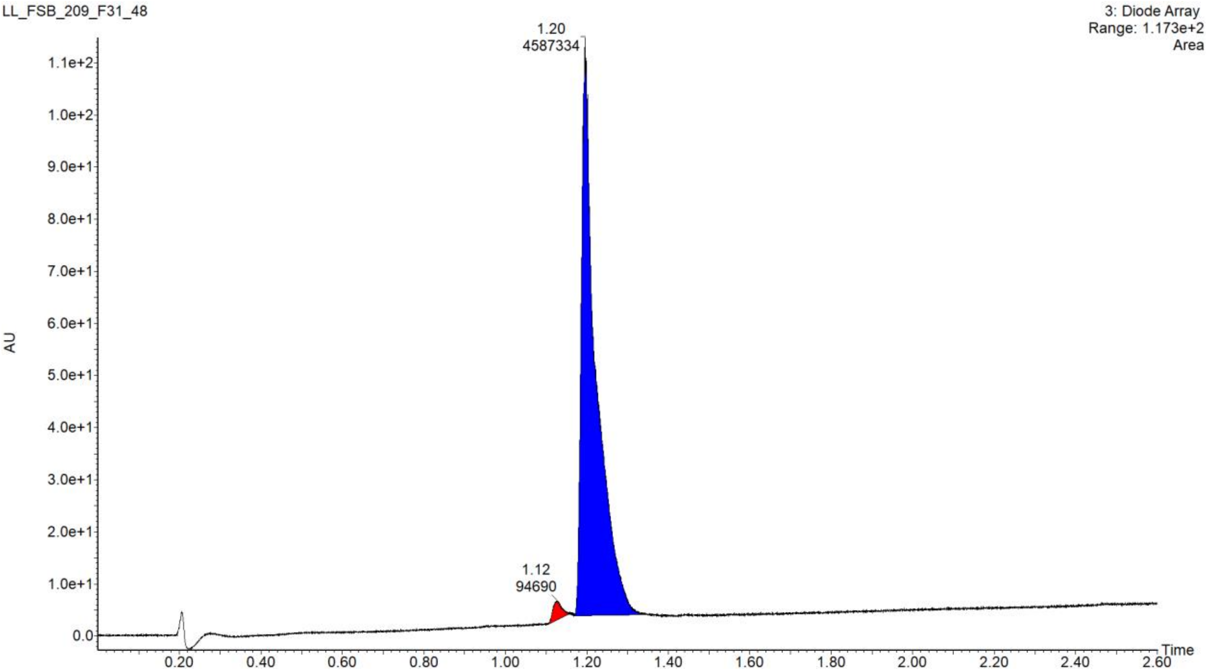

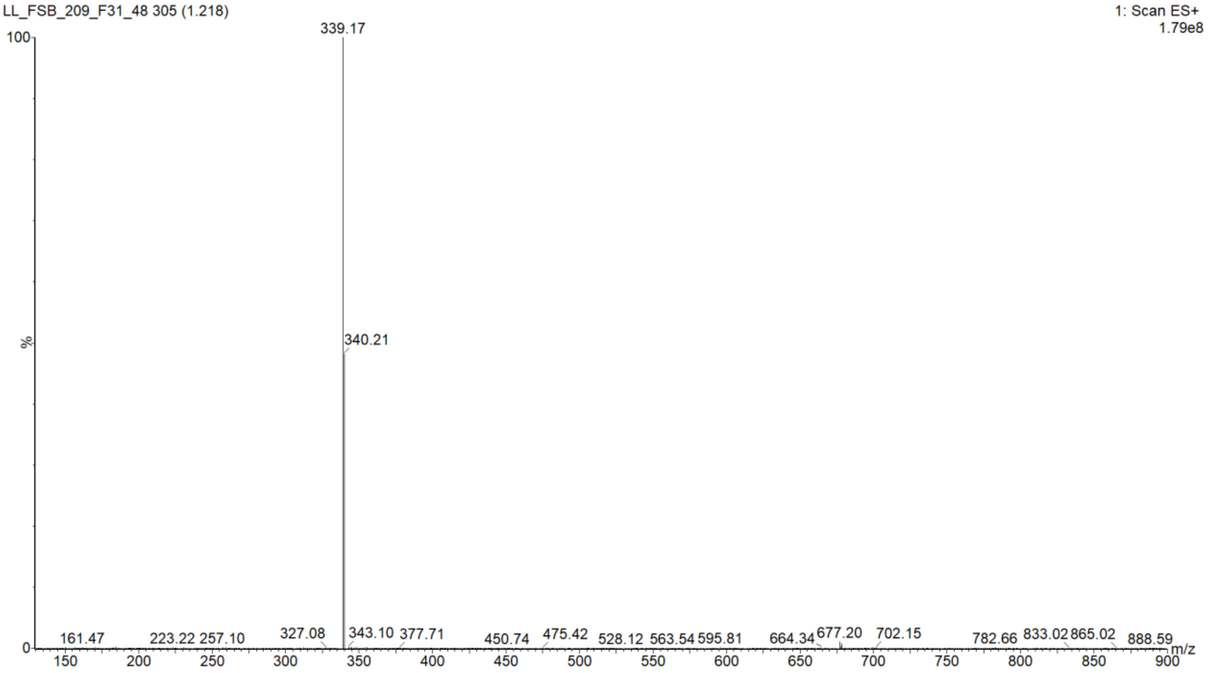

Retention time (min)	*m*/*z* found	Calcd. for [M+H]$^+$	Area	Rel. area (%)
1.11 – 1.16	339.17	-	94690	2
1.17 – 1.35	339.17	339.21	4587334	98

(3*S*,3a*R*,6*S*,12b*S*)-3a-Methyl-11-morpholino-2,3,3a,4,5,6-hexahydro-1*H*-3,6-methanocyclopenta[3,4]oxepino[2,3-*b*]indole, (–)-asteroxin-1 ((–)-19a)

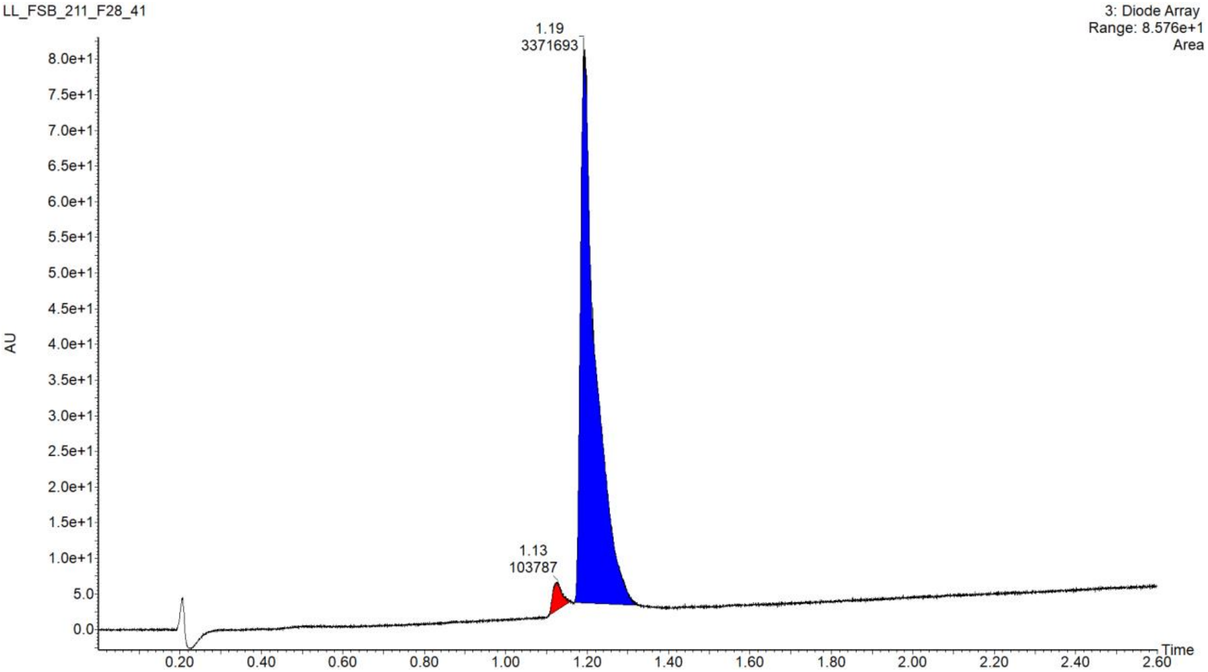

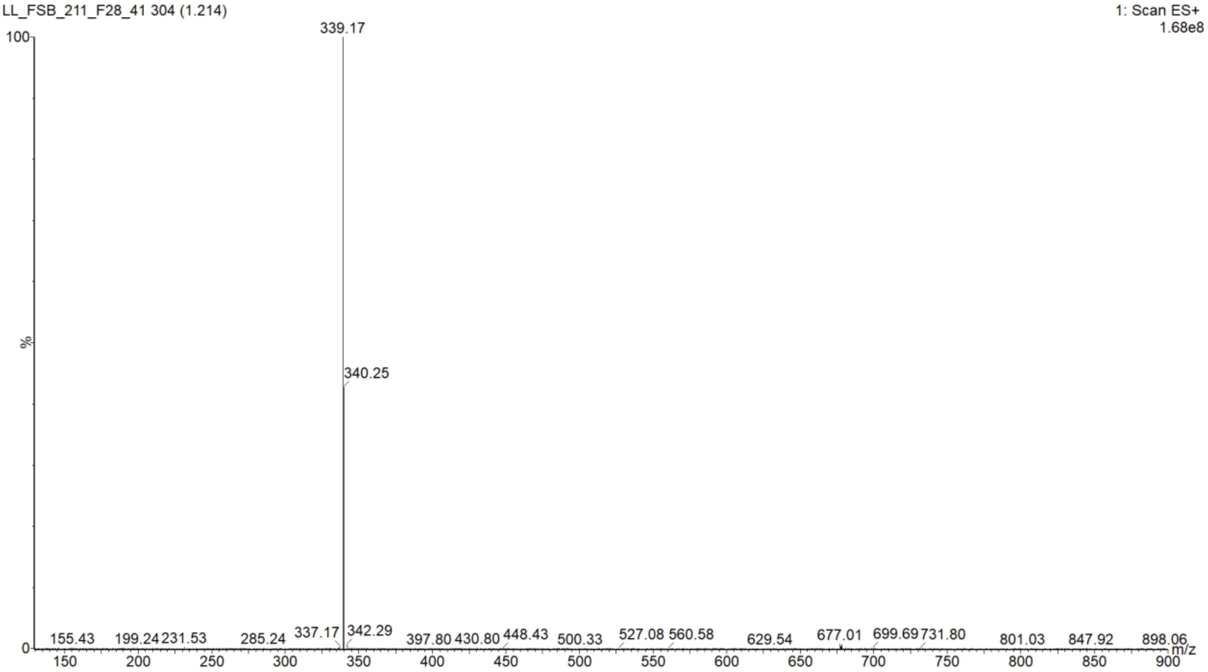

Retention time (min)	*m*/*z* found	Calcd. for [M+H]$^+$	Area	Rel. area (%)
1.11 – 1.16	339.17	-	103787	3
1.17 – 1.34	339.17	339.21	3371693	97

Chiral HPLC chromatograms

Compound S13

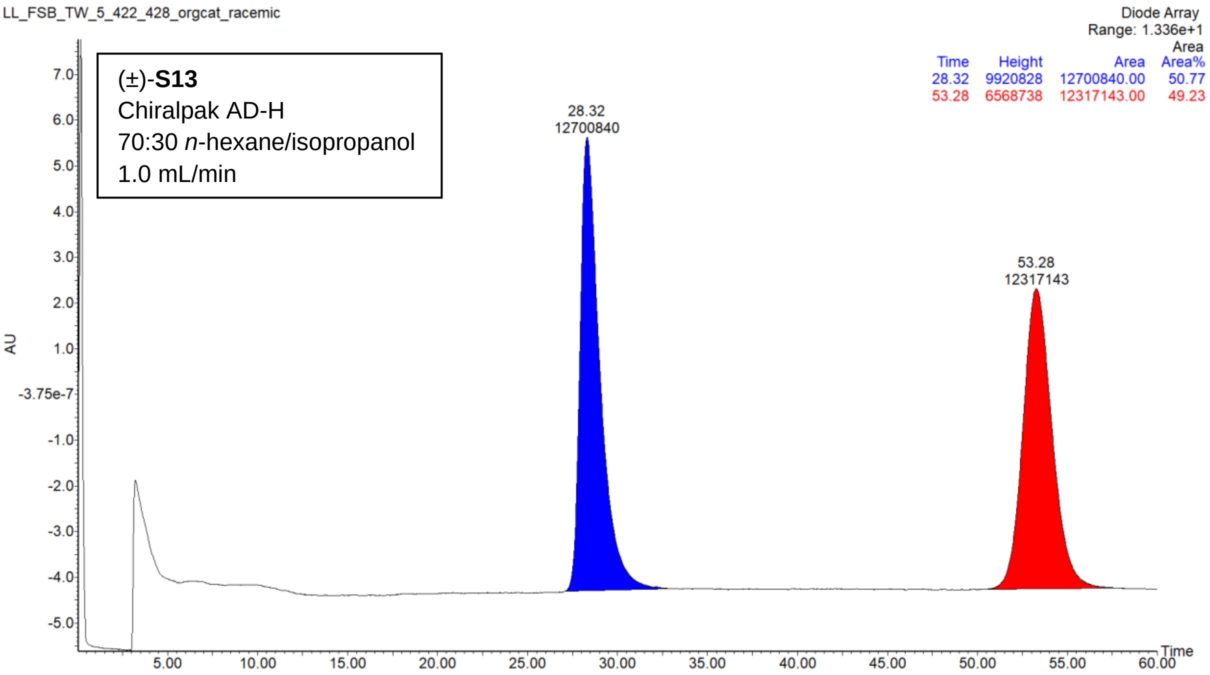

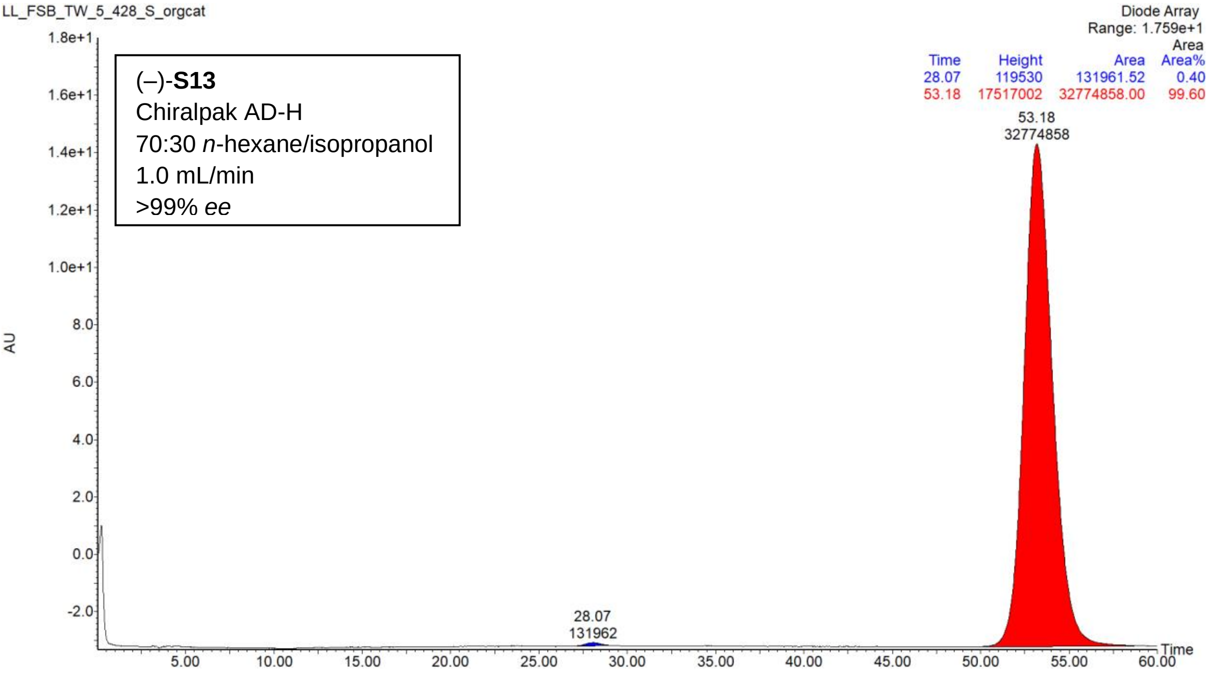

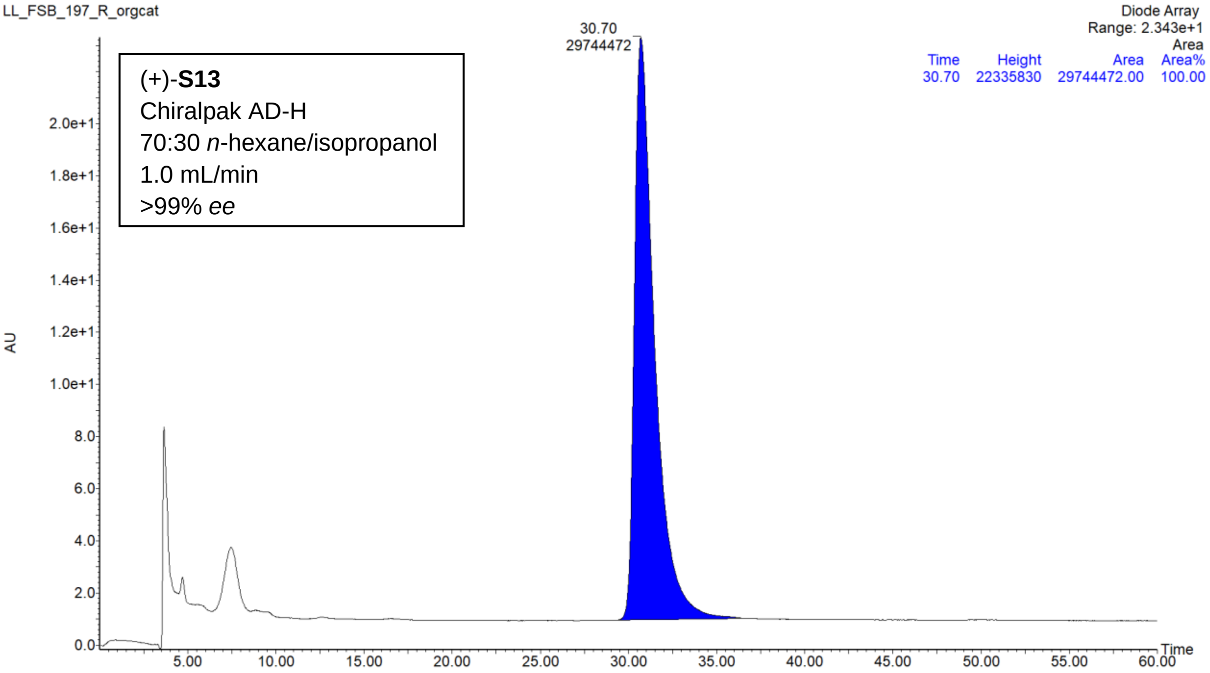
LL_FSB_197_R_orgcat
(+)-**S13**
Chiralpak AD-H
70:30 *n*-hexane/isopropanol
1.0 mL/min
>99% *ee*
Diode Array
Range: 2.343e+1
Time Height Area Area%
30.70 22335830 29744472.00 100.00
30.70
29744472
AU
Time

Compound S2

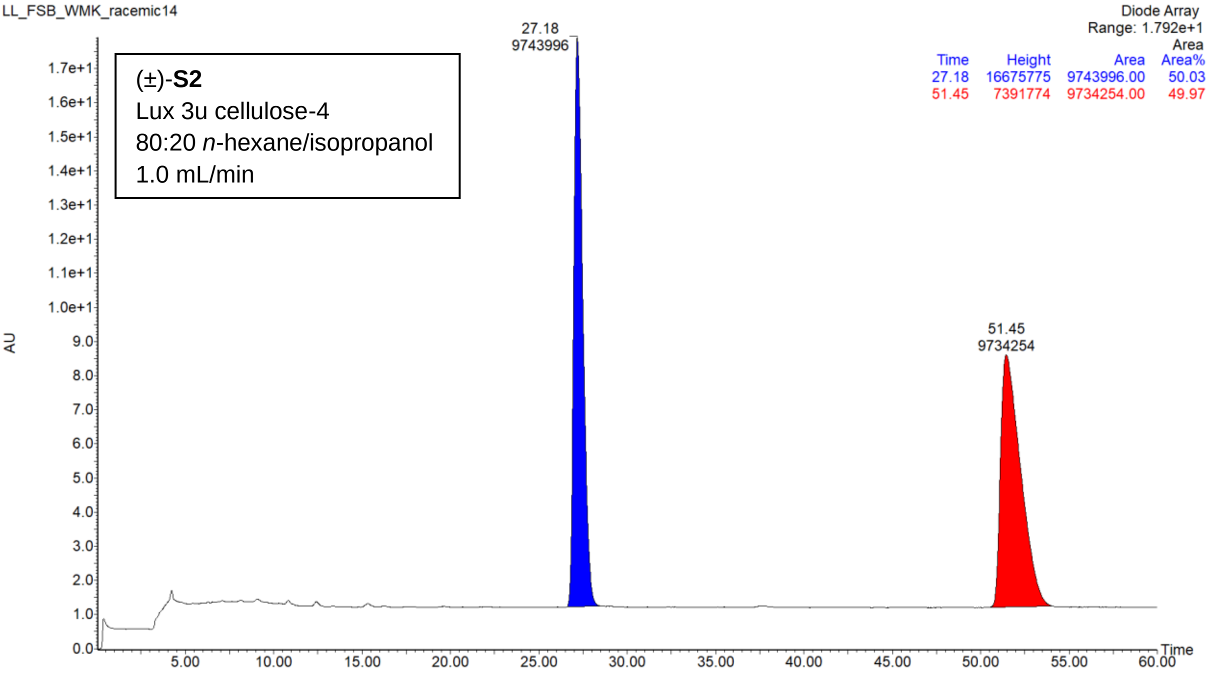
LL_FSB_WMK_racemic14
(±)-**S2**
Lux 3u cellulose-4
80:20 *n*-hexane/isopropanol
1.0 mL/min
Diode Array
Range: 1.792e+1
Time Height Area Area%
27.18 16675775 9743996.00 50.03
51.45 7391774 9734254.00 49.97
27.18
9743996
51.45
9734254
AU
Time

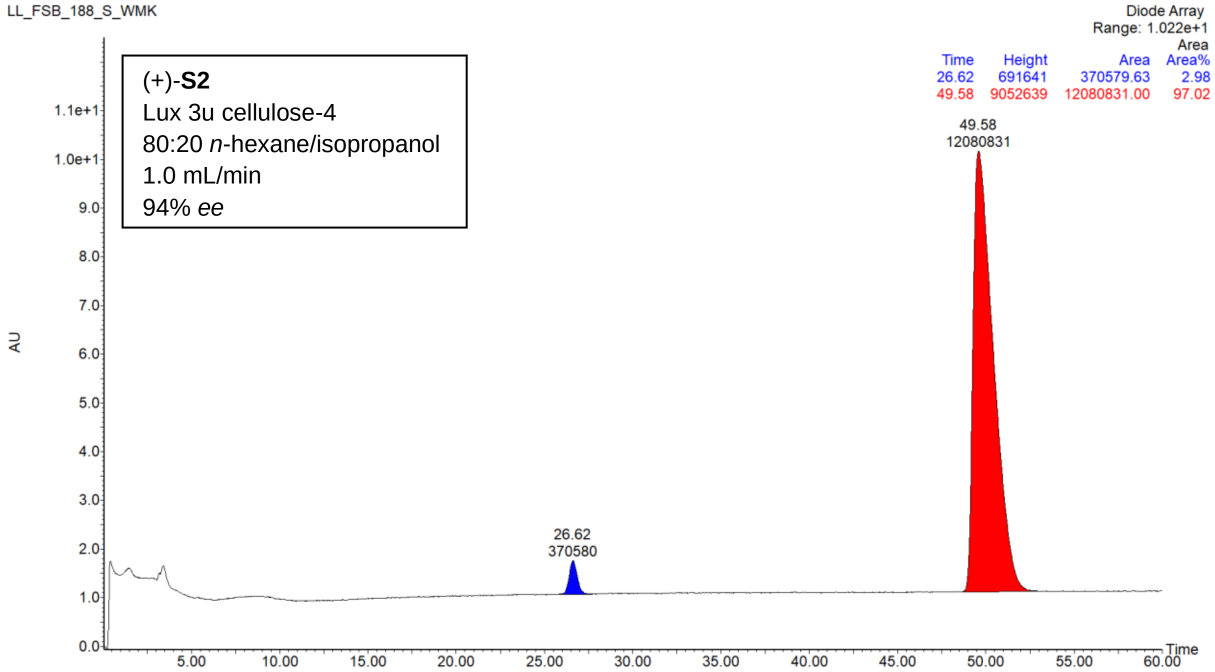
LL_FSB_188_S_WMK
(+)-**S2**
Lux 3u cellulose-4
80:20 *n*-hexane/isopropanol
1.0 mL/min
94% *ee*
Diode Array
Range: 1.022e+1
Time Height Area Area%
26.62 691641 370579.63 2.98
49.58 9052639 12080831.00 97.02
49.58
12080831
26.62
370580
AU
Time

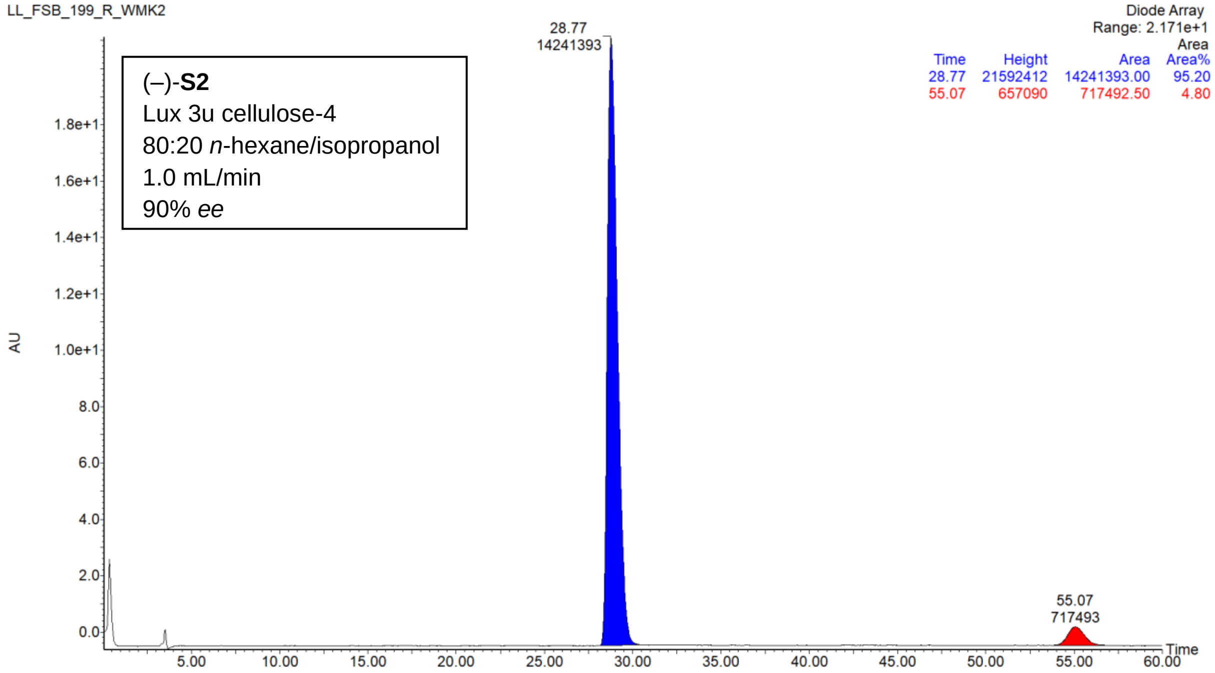
LL_FSB_199_R_WMK2
(–)-**S2**
Lux 3u cellulose-4
80:20 *n*-hexane/isopropanol
1.0 mL/min
90% *ee*
Diode Array
Range: 2.171e+1
Time Height Area Area%
28.77 21592412 14241393.00 95.20
55.07 657090 717492.50 4.80
28.77
14241393
55.07
717493
AU
Time

Compound S3

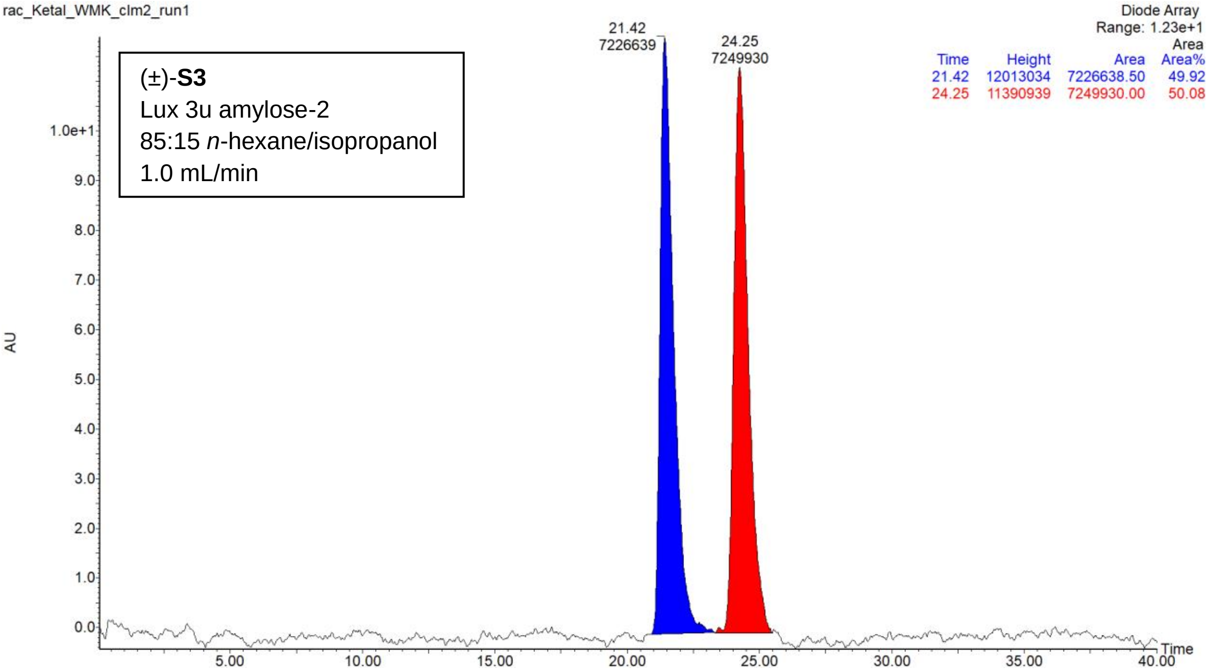
rac_Ketal_WMK_clm2_run1
Diode Array
Range: 1.23e+1
(±)-**S3**
Lux 3u amylose-2
85:15 *n*-hexane/isopropanol
1.0 mL/min
21.42
7226639
24.25
7249930
AU
Time

Time	Height	Area	Area%
21.42	12013034	7226638.50	49.92
24.25	11390939	7249930.00	50.08

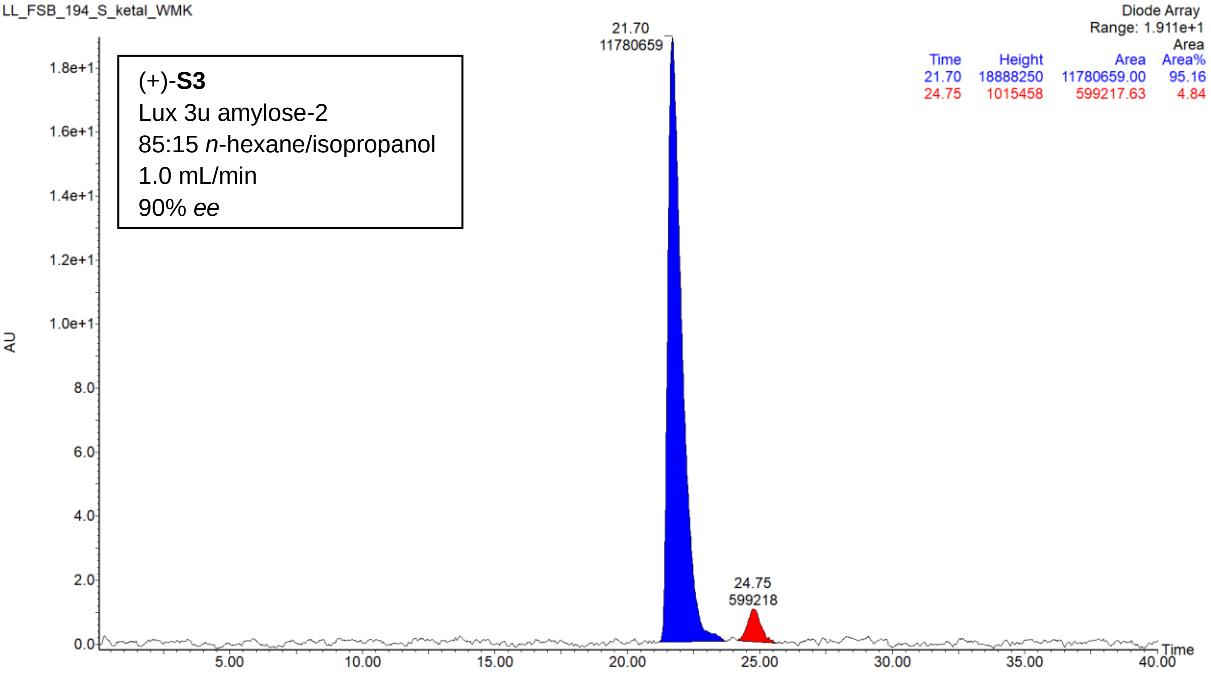
LL_FSB_194_S_ketal_WMK
Diode Array
Range: 1.911e+1
(+)-**S3**
Lux 3u amylose-2
85:15 *n*-hexane/isopropanol
1.0 mL/min
90% *ee*
21.70
11780659
24.75
599218
AU
Time

Time	Height	Area	Area%
21.70	18888250	11780659.00	95.16
24.75	1015458	599217.63	4.84

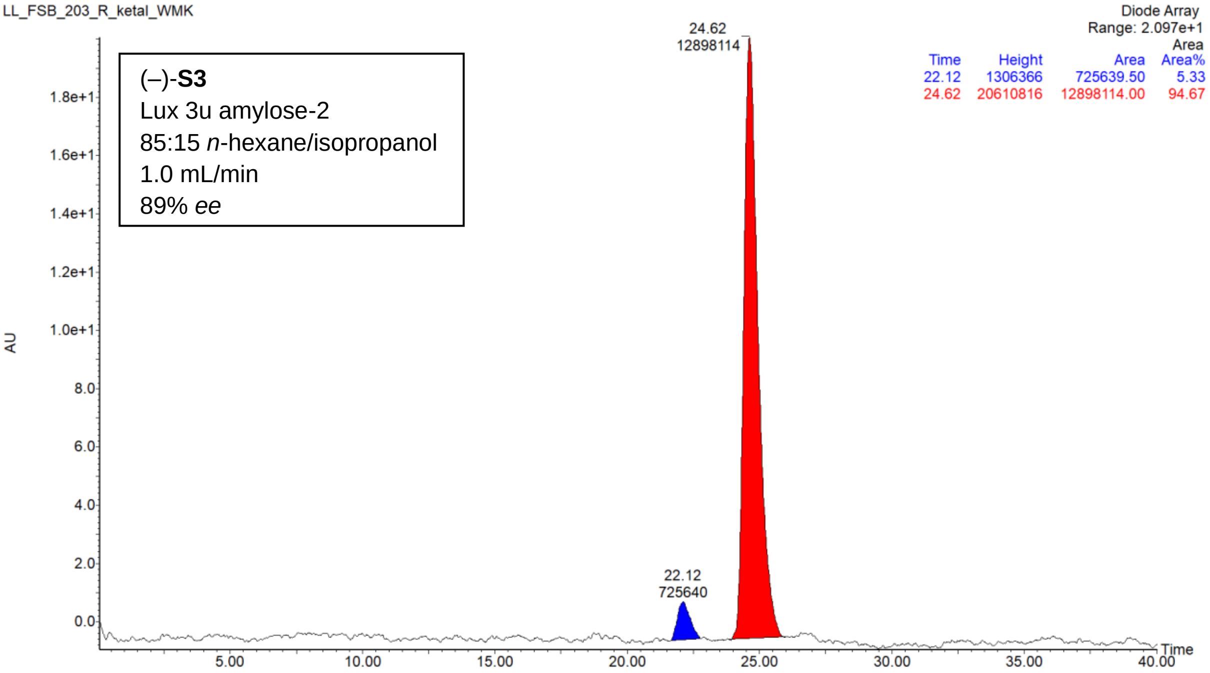

Compound 10b

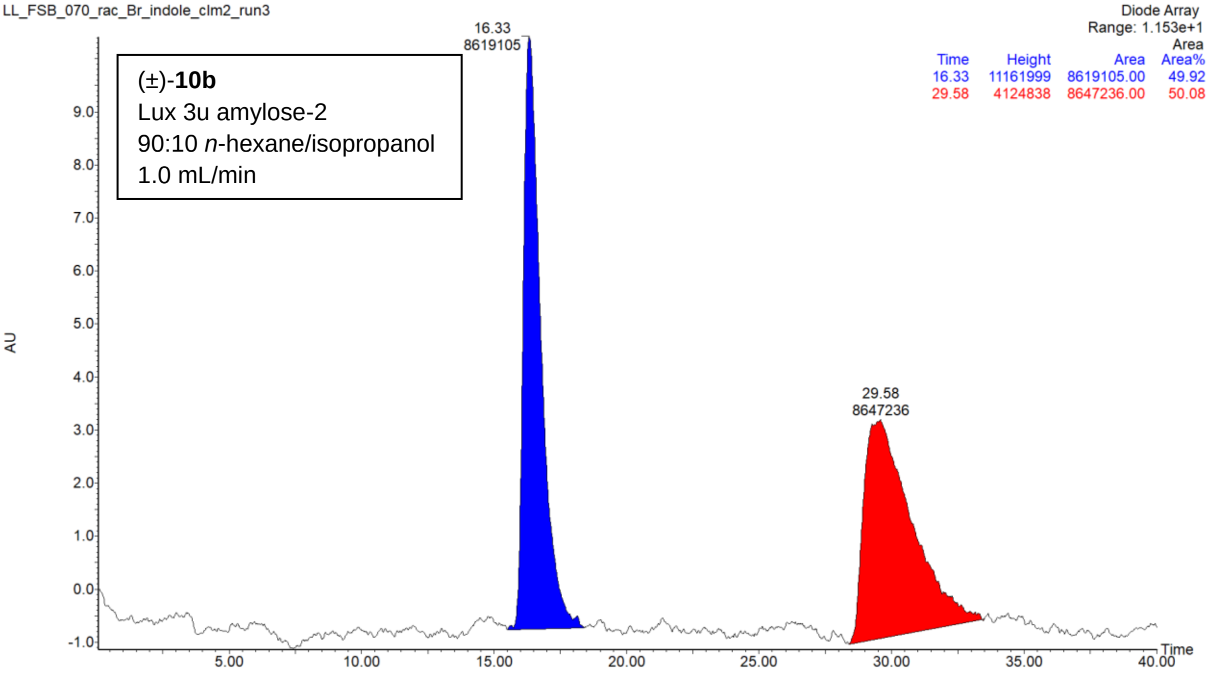

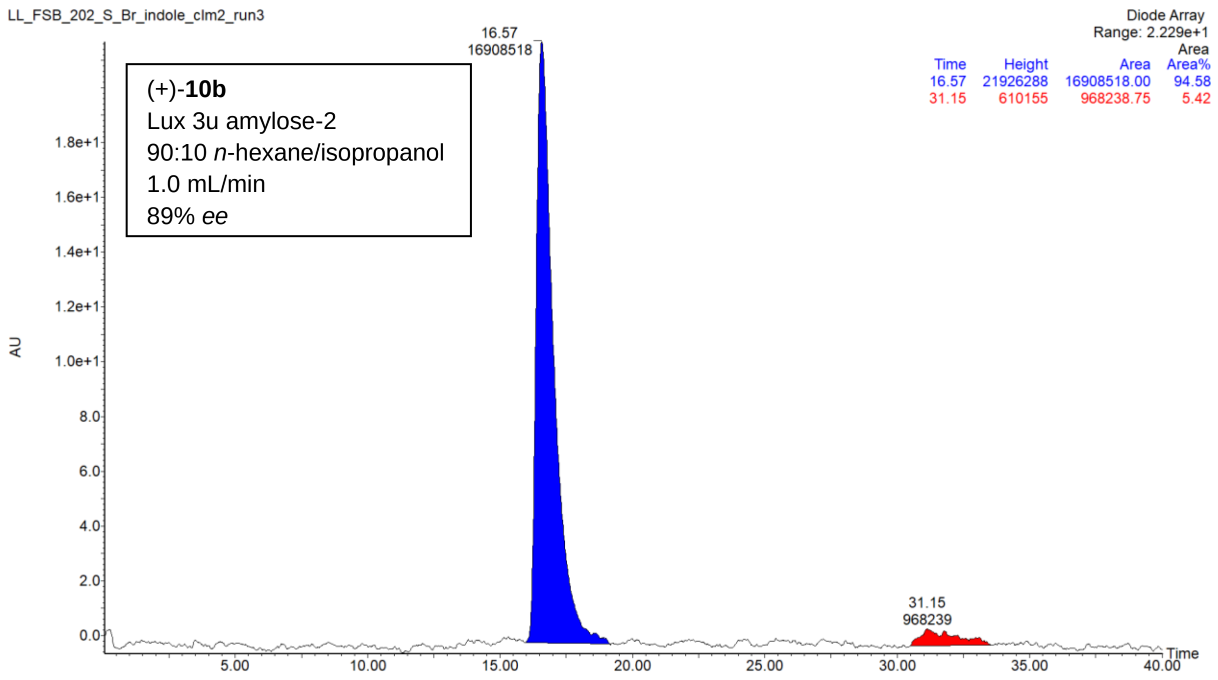
LL_FSB_202_S_Br_indole_clm2_run3
Diode Array
Range: 2.229e+1
(+)-10b
Lux 3u amylose-2
90:10 n-hexane/isopropanol
1.0 mL/min
89% ee
16.57
16908518
31.15
968239
AU
Time
Time Height Area Area%
16.57 21926288 16908518.00 94.58
31.15 610155 968238.75 5.42

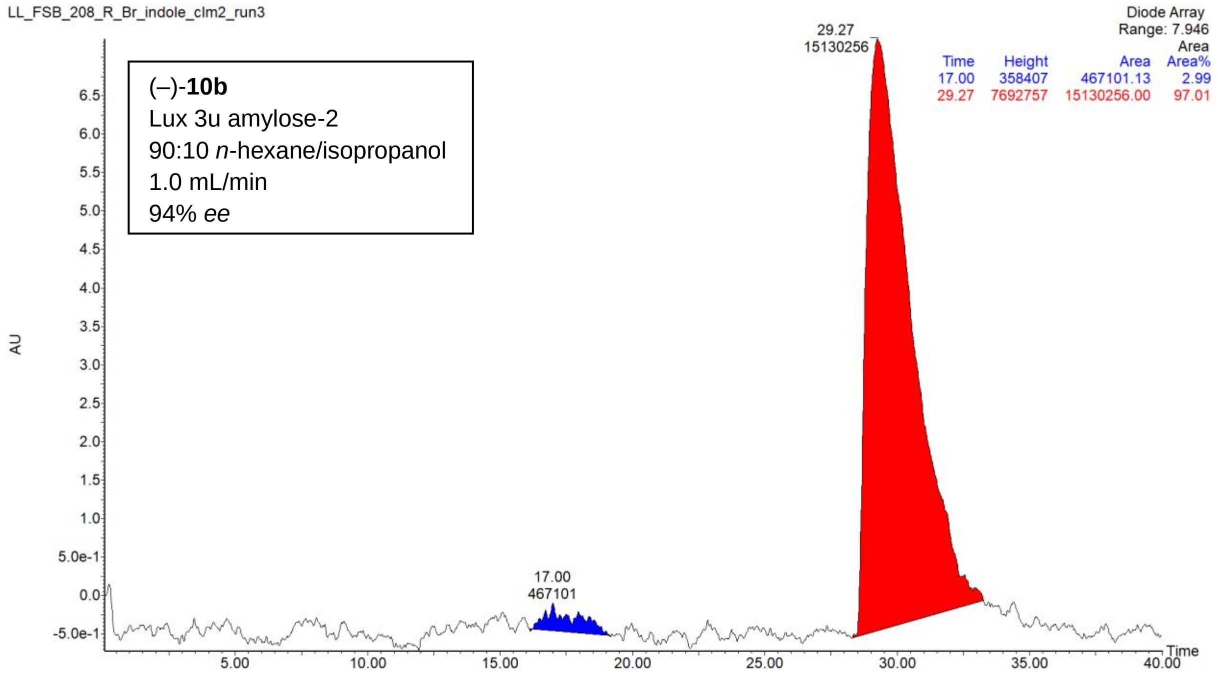
LL_FSB_208_R_Br_indole_clm2_run3
Diode Array
Range: 7.946
(–)-10b
Lux 3u amylose-2
90:10 n-hexane/isopropanol
1.0 mL/min
94% ee
29.27
15130256
17.00
467101
AU
Time
Time Height Area Area%
17.00 358407 467101.13 2.99
29.27 7692757 15130256.00 97.01

Compound 15b

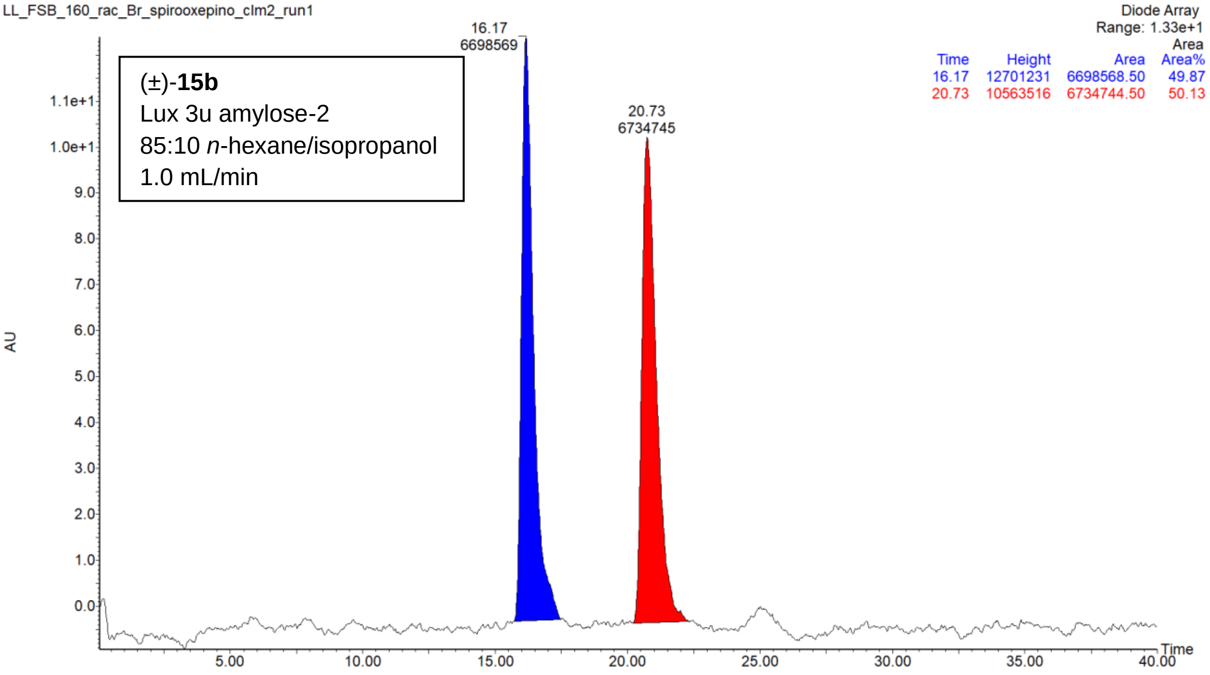

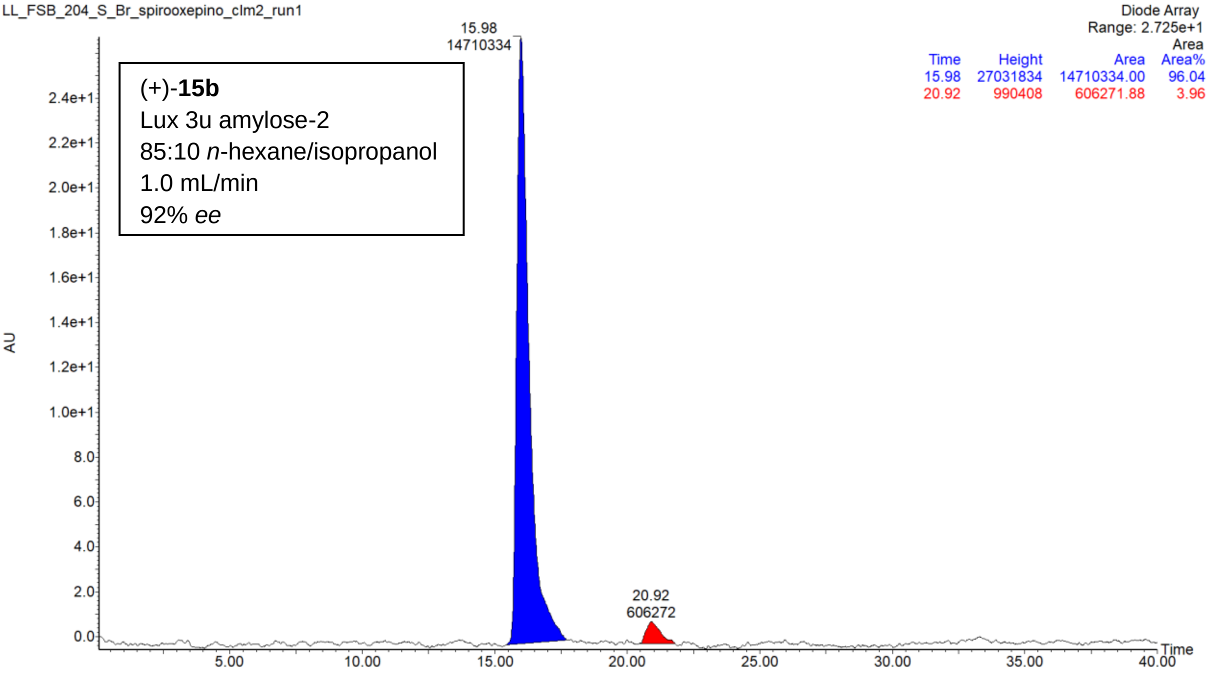

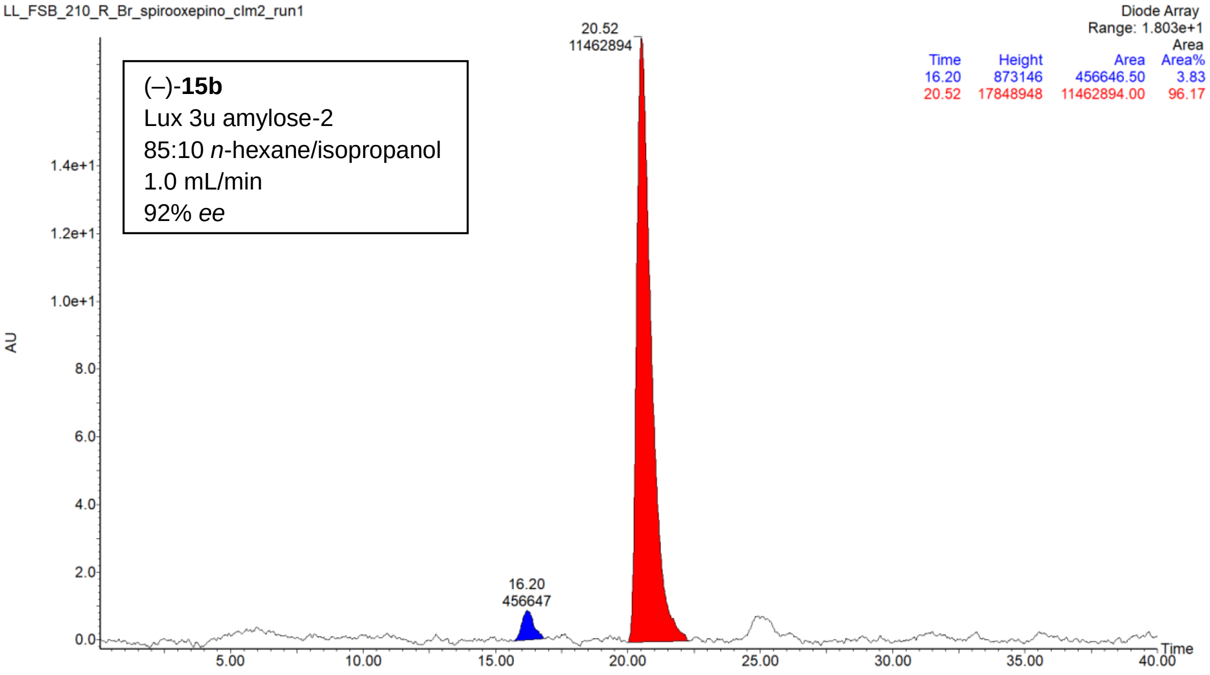

Asteroxin-1 (19a)

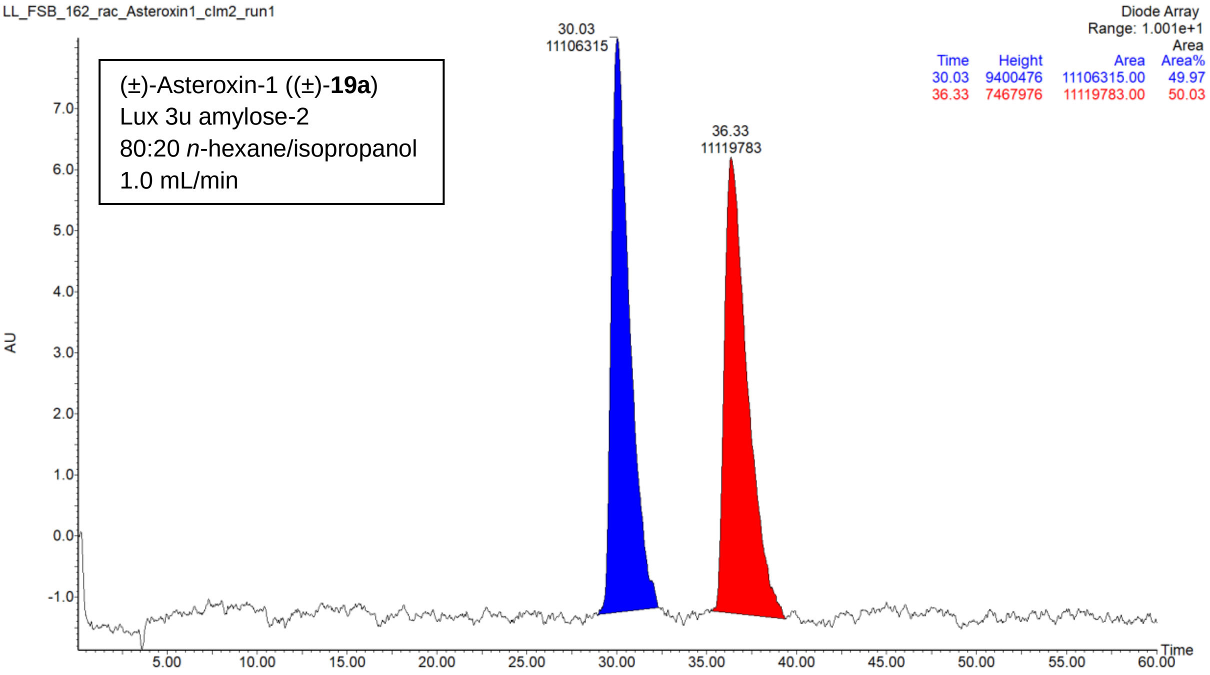

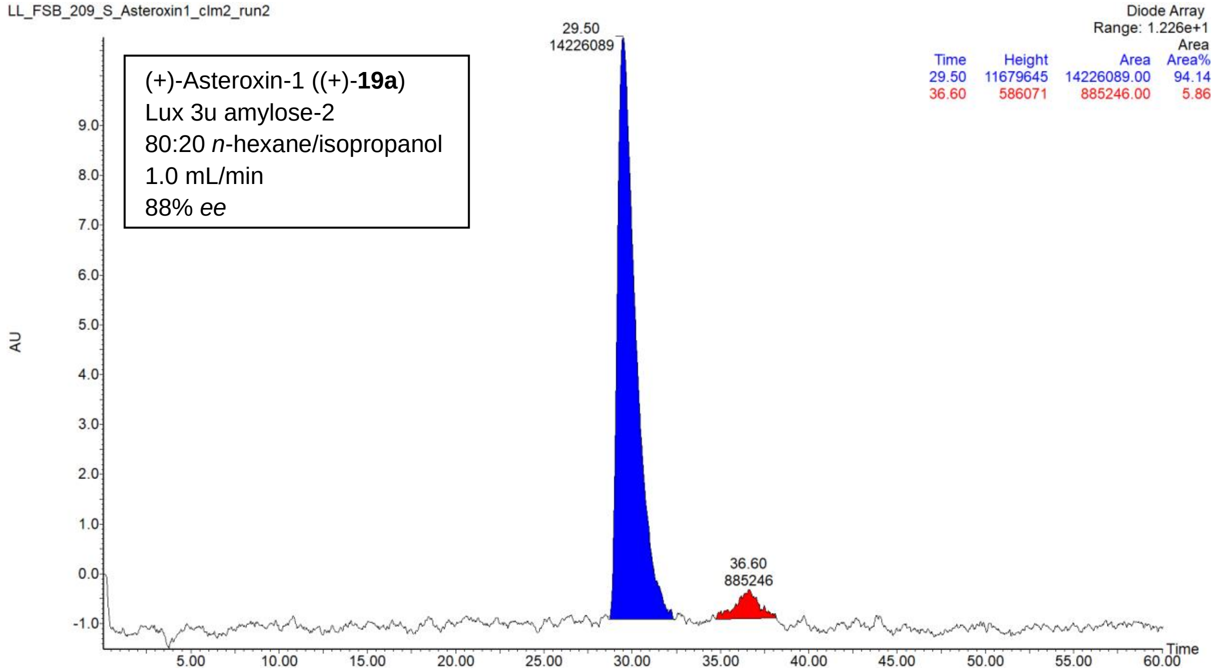
LL_FSB_209_S_Asteroxin1_clm2_run2
Diode Array
Range: 1.226e+1
(+)-Asteroxin-1 ((+)-**19a**)
Lux 3u amylose-2
80:20 *n*-hexane/isopropanol
1.0 mL/min
88% *ee*
Time Height Area Area%
29.50 11679645 14226089.00 94.14
36.60 586071 885246.00 5.86
29.50
14226089
36.60
885246
AU
Time

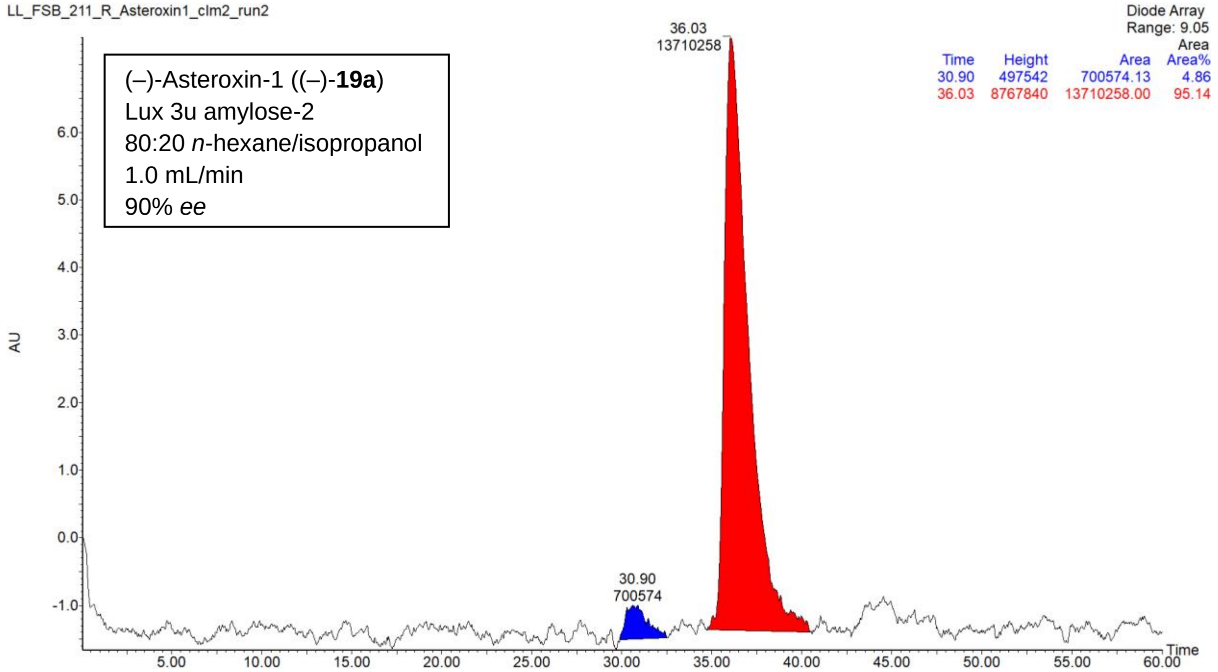
LL_FSB_211_R_Asteroxin1_clm2_run2
Diode Array
Range: 9.05
(–)-Asteroxin-1 ((–)-**19a**)
Lux 3u amylose-2
80:20 *n*-hexane/isopropanol
1.0 mL/min
90% *ee*
Time Height Area Area%
30.90 497542 700574.13 4.86
36.03 8767840 13710258.00 95.14
36.03
13710258
30.90
700574
AU
Time

X-ray crystallographic data

(1*S**,4a*R**,6*R**,8a*S**)-6-Hydroxy-8a-methyl-3,4,4a,5,6,7,8,8a-octahydro-1'*H*,2*H*-spiro[naphthalene-1,2'-quinazolin]-4'(3'*H*)-one (5a)

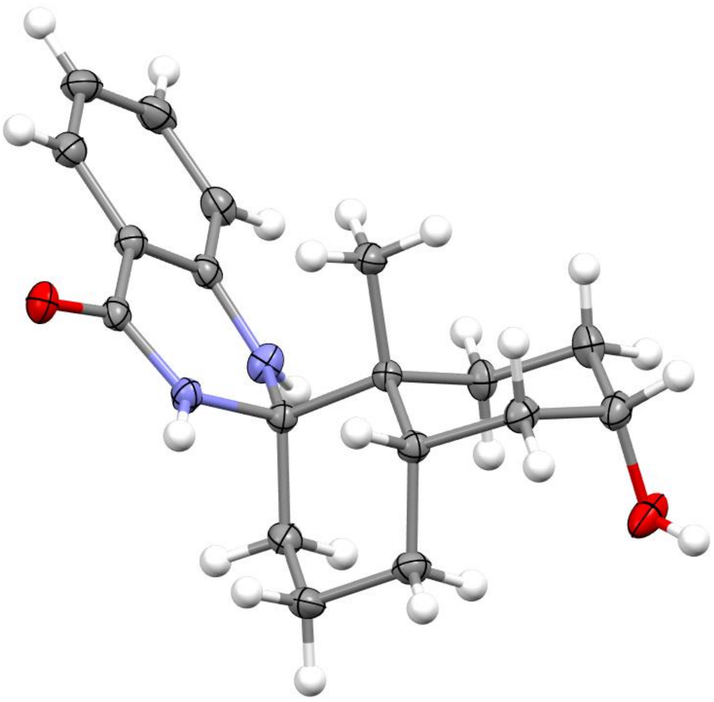

X-ray crystal structure of **5a** (CCDC 2347999)

The X-ray crystallographic experiment was carried out as described in the general directions. Using Olex2[23], the structure was solved with the SHELXD[24] structure solution program using Dual Space and refined with the SHELXL[25] refinement package using Least Squares minimisation. All non-hydrogen atoms were refined anisotropically. The crystallographic data are summarised in Table S7.

Note: Good data and refinement. There is one copy of the molecule in the asymmetric unit. The crystal structure is of racemic material but only enantiomer (*R*,*S*,*S*,*R*)-**5a** is represented in the structure. Comment to alert “PLAT420_ALERT_2_C D-H Bond Without Acceptor N004--H004”: There are no suitable acceptors around this NH group.

Table S7: Crystal data and structure refinement for LL_FSB_114_F34_59 (**5a**).

CCDC number	2347999
Identification code	LL_FSB_114_F34_59
Empirical formula	$C_{18}H_{24}N_2O_2$
Formula weight	300.39
Temperature / K	120.00(10)
Crystal system	orthorhombic
Space group	*Pccn*
a / Å	10.4971(2)
b / Å	20.5111(5)
c / Å	13.9749(4)
α / °	90
β / °	90
γ / °	90
Volume / Å^3	3008.89(13)
Z	8
ρ_{calc} / g·cm^{-3}	1.326
μ / mm^{-1}	0.688
F(000)	1296.0
Crystal size / mm^3	0.45 × 0.2 × 0.03

Radiation	Cu Kα (λ = 1.54184)
2Θ range for data collection / °	8.622 to 153.864
Index ranges	-13 ≤ h ≤ 13, -25 ≤ k ≤ 25, -16 ≤ l ≤ 17
Reflections collected	59724
Independent reflections	3146 [R_{int} = 0.0589, R_{sigma} = 0.0175]
Data / restraints / parameters	3146 / 0 / 205
Goodness-of-fit on F^2	1.048
Final *R* indexes [I>=2σ (I)]	R_1 = 0.0389, wR_2 = 0.1035
Final *R* indexes [all data]	R_1 = 0.0447, wR_2 = 0.1087
Largest diff. peak and hole / e Å^{-3}	0.24 and -0.26

(1*R,3a*R**,5*R**,7a*S**)-5-Hydroxy-5',7a-dimethyl-2,3,3a,4,5,6,7,7a-octahydrospiro[indene-1,2'-indolin]-3'-one (13)**

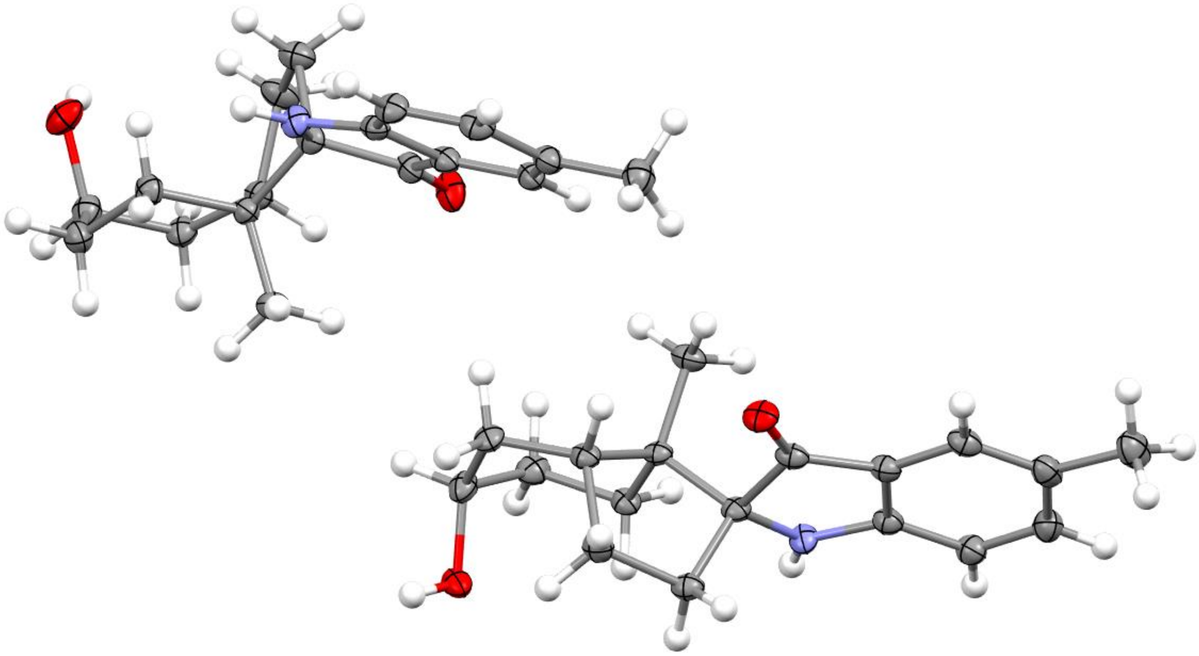

X-ray crystal structure of **13** (CCDC 2345800)

The X-ray crystallographic experiment was carried out as described in the general directions. Using Olex2[23], the structure was solved with the SHELXT[26] structure solution program using Intrinsic Phasing and refined with the SHELXL[25] refinement package using Least Squares minimisation. All non-hydrogen atoms were refined anisotropically. The crystallographic data are summarised in Table S8.

Note: Poor but acceptable data and refinement. The crystal is twinned with BASF 0.3291(10). There are two copies of the molecule in the asymmetric unit. The crystal structure is of racemic material but only enantiomer (*R*,*R*,*R*,*S*)-**13** is represented in the structure.

Table S8: Crystal data and structure refinement for LL_FSB_133_F51_71_twin1_hklf4 (**13**).

CCDC number	2345800
Identification code	LL_FSB_133_F51_71_twin1_hklf4
Empirical formula	$C_{18}H_{23}NO_2$
Formula weight	285.37
Temperature / K	120.00(10)
Crystal system	triclinic
Space group	*P-1*
a / Å	7.1510(5)
b / Å	13.3261(10)
c / Å	15.9841(11)
α / °	84.767(6)
β / °	82.534(6)
γ / °	79.158(6)
Volume / Å^3	1479.84(19)
Z	4
ρ_{calc} / g·cm^{-3}	1.281
μ / mm^{-1}	0.653
F(000)	616.0
Crystal size / mm^3	0.7 × 0.5 × 0.2
Radiation	Cu Kα (λ = 1.54184)
2*Θ* range for data collection / °	8.482 to 153.976
Index ranges	-9 ≤ h ≤ 9, -16 ≤ k ≤ 16, -20 ≤ l ≤ 20
Reflections collected	9856
Independent reflections	9856 [R_{sigma} = 0.0294]

Data / restraints / parameters	9856 / 0 / 386
Goodness-of-fit on F^2	1.040
Final R indexes [I>=2σ (I)]	R_1 = 0.0520, wR_2 = 0.1415
Final R indexes [all data]	R_1 = 0.0635, wR_2 = 0.1478
Largest diff. peak and hole / e Å^{-3}	0.40 and -0.43

(3*R,4a*R**,6a*R**,11b*S**)-8-Methoxy-11b-methyl-1,2,3,4,4a,5,6,11b-octahydro-6a*H*-benzo[*a*]carbazole-3,6a-diol (14b)**

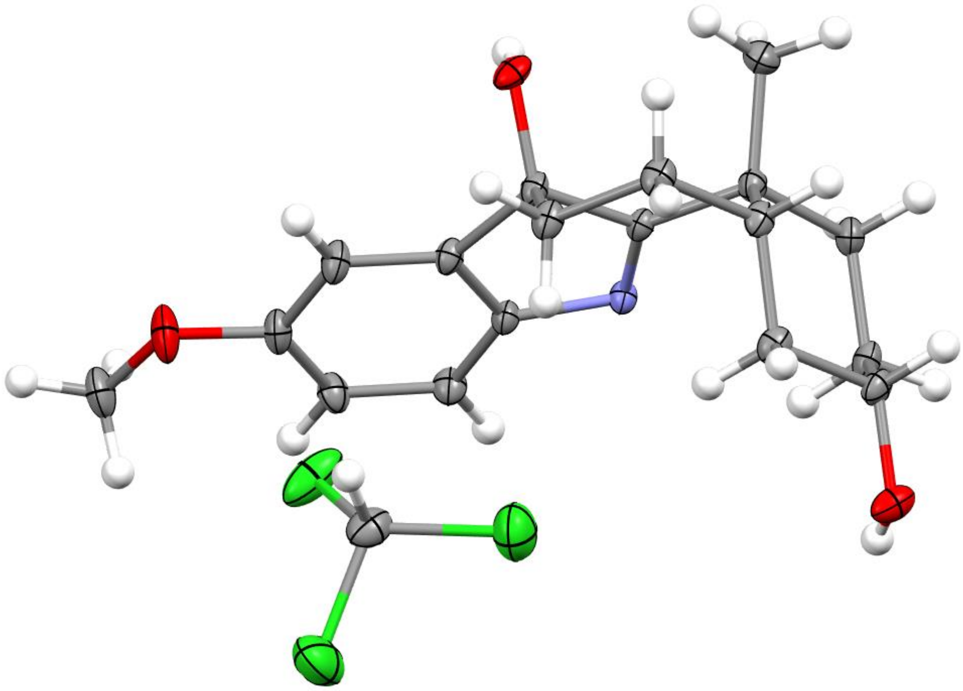

X-ray crystal structure of **14b**·$CHCl_3$ (CCDC 2345784)

The X-ray crystallographic experiment was carried out as described in the general directions. Using Olex2[23], the structure was solved with the SHELXT[26] structure solution program using Intrinsic Phasing and refined with the SHELXL[25] refinement package using Least Squares minimisation. All non-hydrogen atoms were refined anisotropically. The crystallographic data are summarised in Table S9.

Note: Good data and refinement. There is one copy of the molecule and a chloroform molecule in the asymmetric unit. The crystal structure is of racemic material but only enantiomer (*S*,*S*,*S*,*R*)-**14b** is represented in the structure.

Table S9: Crystal data and structure refinement for LL_FSB_081_F26_34 (**14b**).

CCDC number	2345784
Identification code	LL_FSB_081_F26_34
Empirical formula	$C_{19}H_{24}Cl_3NO_3$
Formula weight	420.74
Temperature / K	120.00(10)
Crystal system	monoclinic
Space group	$P2_1/c$
a / Å	11.19820(10)
b / Å	21.3184(2)
c / Å	8.36990(10)
α / °	90
β / °	98.6410(10)
γ / °	90
Volume / Å³	1975.45(4)
Z	4
ρ_{calc} / g·cm^{-3}	1.415
μ / mm^{-1}	4.359
F(000)	880.0
Crystal size / mm³	0.5 × 0.2 × 0.1
Radiation	Cu Kα (λ = 1.54184)
2*Θ* range for data collection / °	7.986 to 153.148
Index ranges	-14 ≤ h ≤ 14, -26 ≤ k ≤ 25, -10 ≤ l ≤ 10

Reflections collected	44253
Independent reflections	4140 [R_{int} = 0.0693, R_{sigma} = 0.0236]
Data / restraints / parameters	4140 / 0 / 242
Goodness-of-fit on F^2	1.078
Final R indexes [I>=2σ (I)]	R_1 = 0.0461, wR_2 = 0.1261
Final R indexes [all data]	R_1 = 0.0482, wR_2 = 0.1285
Largest diff. peak and hole / e Å^{-3}	1.09 and -0.73

(3*R,3a*S**,6*R**,12b*R**)-3a-Methyl-2,3,3a,4,5,6-hexahydro-1*H*-3,6-methanocyclopenta[3,4]oxepino[2,3-*b*]indole (15a)**

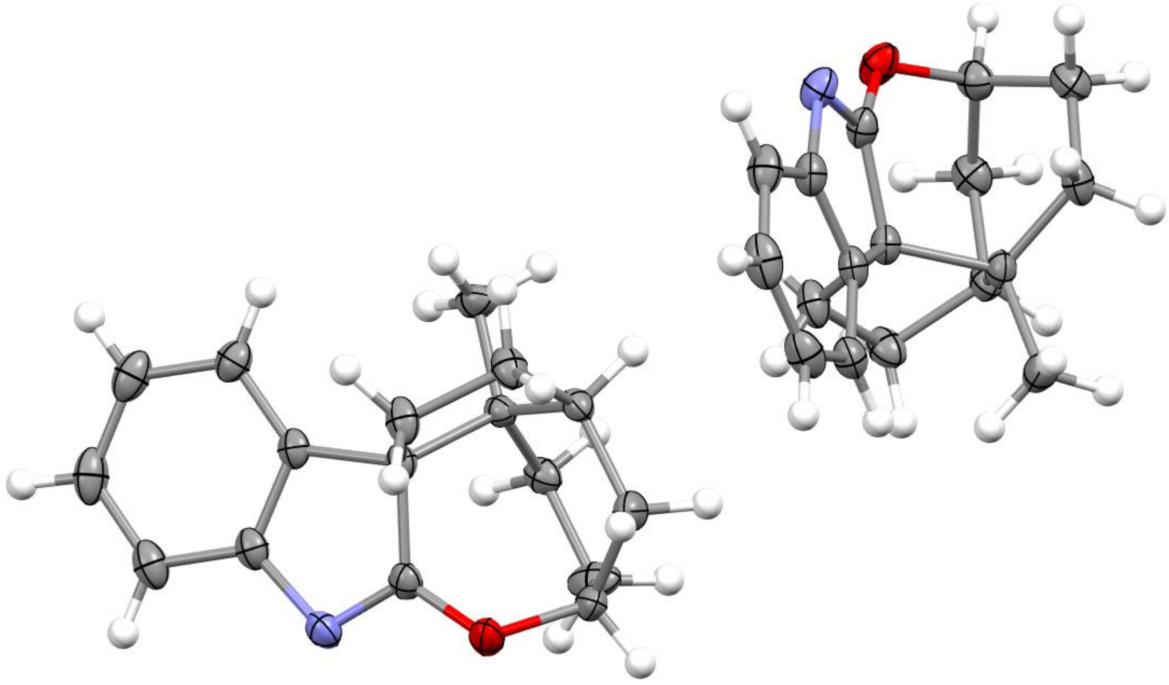

X-ray crystal structure of **15a** (CCDC 2345798)

The X-ray crystallographic experiment was carried out as described in the general directions. Using Olex2[23], the structure was solved with the SHELXT[26] structure solution program using Intrinsic Phasing and refined with the SHELXL[25] refinement package using Least Squares minimisation. All non-hydrogen atoms were refined anisotropically. The crystallographic data are summarised in Table S10.

Note: Acceptable data and refinement. The crystal is twinned with BASF 0.4841(10). There are two copies of the molecule in the asymmetric unit. The crystal structure is of racemic material but only enantiomer (*S*,*R*,*S*,*S*)-**15a** is represented in the structure.

Table S10: Crystal data and structure refinement for LL_FSB_073_twin1_hklf4 (**15a**).

CCDC number	2345798
Identification code	LL_FSB_073_twin1_hklf4
Empirical formula	$C_{17}H_{19}NO$
Formula weight	253.33
Temperature / K	120.00(10)
Crystal system	triclinic
Space group	*P-1*
a / Å	7.1011(2)
b / Å	12.3747(3)
c / Å	14.8259(4)
α / °	89.849(2)
β / °	84.179(2)
γ / °	86.986(2)
Volume / Å^3	1294.30(6)
Z	4
ρ_{calc} / g·cm^{-3}	1.300
μ / mm^{-1}	0.624
F(000)	544.0
Crystal size / mm^3	0.4 × 0.2 × 0.1
Radiation	Cu Kα (λ = 1.54184)
2*Θ* range for data collection / °	7.154 to 152.992
Index ranges	-8 ≤ h ≤ 8, -15 ≤ k ≤ 15, -18 ≤ l ≤ 18

Reflections collected	16796
Independent reflections	16796 [R_{sigma} = 0.0148]
Data / restraints / parameters	16796 / 0 / 346
Goodness-of-fit on F^2	1.038
Final R indexes [I>=2σ (I)]	R_1 = 0.0458, wR_2 = 0.1238
Final R indexes [all data]	R_1 = 0.0503, wR_2 = 0.1266
Largest diff. peak and hole / e $Å^{-3}$	0.41 and -0.22

References

(1) Pavia, D. L.; Lampman, G. M.; Kriz, G. S.; Vyvyan, J. R. *Introduction to Spectroscopy*; Cengage Learning, 2013.

(2) Pretsch, E.; Bühlmann, P.; Badertscher, M. *Structure Determination of Organic Compounds*; Springer, 2020.

(3) Laraia, L.; Friese, A.; Corkery, D. P.; Konstantinidis, G.; Erwin, N.; Hofer, W.; Karatas, H.; Klewer, L.; Brockmeyer, A.; Metz, M.; Schölermann, B.; Dwivedi, M.; Li, L.; Rios-Munoz, P.; Köhn, M.; Winter, R.; Vetter, I. R.; Ziegler, S.; Janning, P.; Wu, Y.-W.; Waldmann, H. The Cholesterol Transfer Protein GRAMD1A Regulates Autophagosome Biogenesis. *Nat. Chem. Biol.* **2019**, *15* (7), 710–720. https://doi.org/10.1038/s41589-019-0307-5.

(4) Tsujishita, Y.; Hurley, J. H. Structure and Lipid Transport Mechanism of a StAR-Related Domain. *Nat. Struct. Biol.* **2000**, *7* (5), 408–414. https://doi.org/10.1038/75192.

(5) Tan, L.; Tong, J.; Chun, C.; Im, Y. J. Structural Analysis of Human Sterol Transfer Protein STARD4. *Biochem. Biophys. Res. Commun.* **2019**, *520* (2), 466–472. https://doi.org/10.1016/j.bbrc.2019.10.054.

(6) Portoghese, P. S. Revision of Purity Criteria for Tested Compounds. *J. Med. Chem.* **2009**, *52* (1), 1. https://doi.org/10.1021/jm801525s.

(7) Davies, S. G.; Russell, A. J.; Sheppard, R. L.; Smith, A. D.; Thomson, J. E. Evaluating β-Amino Acids as Enantioselective Organocatalysts of the Hajos–Parrish–Eder–Sauer–Wiechert Reaction. *Org. Biomol. Chem.* **2007**, *5* (19), 3190–3200. https://doi.org/10.1039/B711171A.

(8) Bradshaw, B.; Etxebarria-Jardí, G.; Bonjoch, J.; Viózquez, S. F.; Guillena, G.; Nájera, C. Synthesis of (S)-8a-Methyl-3,4,8,8a-Tetrahydro-1,6-(2H,7H)-Naphthalenedione via N-Tosyl-(Sa)-Binam-L-Prolinamide Organocatalysis. *Org. Synth.* **2011**, *88*, 330–341. https://doi.org/10.15227/orgsyn.088.0330.

(9) Corey, E. J.; Ohno, M.; Vatakencherry, P. A.; Mitra, R. B. Total Synthesis of d,l-Longifolene. *J. Am. Chem. Soc.* **1961**, *83* (5), 1251–1253. https://doi.org/10.1021/ja01466a056.

(10) Karimov, R. R.; Tan, D. S.; Gin, D. Y. Synthesis of the Hexacyclic Triterpene Core of the Jujuboside Saponins via Tandem Wolff Rearrangement–Intramolecular Ketene Hetero-Diels–Alder Reaction. *Tetrahedron* **2018**, *74* (26), 3370–3383. https://doi.org/10.1016/j.tet.2018.04.051.

(11) Whitmarsh-Everiss, T.; Olsen, A. H.; Laraia, L. Identification of Inhibitors of Cholesterol Transport Proteins Through the Synthesis of a Diverse, Sterol-Inspired Compound Collection. *Angew. Chem. Int. Ed.* **2021**, *60* (51), 26755–26761. https://doi.org/10.1002/anie.202111639.

(12) Park, K.; Scott, W. J.; Wiemer, D. F. Effect of C-9 Substituents on the Regioselectivity of A-Ring Reactions in Derivatives of the Wieland-Miescher Ketone. *J. Org. Chem.* **1994**, *59* (21), 6313–6317. https://doi.org/10.1021/jo00100a037.

(13) Kametani, T.; Suzuki, K.; Nemoto, H. Studies on the Syntheses of Heterocyclic and Natural Compounds. 950. Asymmetric Total Synthesis of (+)-Chenodeoxycholic Acid. Stereoselectivity of Intramolecular Cycloaddition of Olefinic o-Quinodimethanes. *J. Org. Chem.* **1982**, *47* (12), 2331–2342. https://doi.org/10.1021/jo00133a020.

(14) Sammes, P. G.; Street, L. J.; Whitby, R. J. Synthesis of (±)-Cryptofauronol and Related Valerane Sesquiterpenes via Rearrangement of Bicyclo[5.3.0]Decane Precursors. *J. Chem. Soc. Perkin Trans. I* **1986**, 281–289. https://doi.org/10.1039/P19860000281.

(15) Ottolina, G.; de Gonzalo, G.; Carrea, G.; Danieli, B. Enzymatic Baeyer–Villiger Oxidation of Bicyclic Diketones. *Adv. Synth. Catal.* **2005**, *347* (7–8), 1035–1040. https://doi.org/10.1002/adsc.200505027.

(16) Jung, M. E.; Guzaev, M. Studies Toward the Enantiospecific Total Synthesis of Rhodexin A. *J. Org. Chem.* **2013**, *78* (15), 7518–7526. https://doi.org/10.1021/jo400909t.

(17) Wodtke, R.; Steinberg, J.; Köckerling, M.; Löser, R.; Mamat, C. NMR-Based Investigations of Acyl-Functionalized Piperazines Concerning Their Conformational Behavior in Solution. *RSC Adv.* **2018**, *8* (71), 40921–40933. https://doi.org/10.1039/C8RA09152H.

(18) Viózquez, S. F.; Guillena, G.; Nájera, C.; Bradshaw, B.; Etxebarria-Jardi, G.; Bonjoch, J. (Sa,S)-N-[2´-(4-Methylphenylsulfonamido)-1,1´-Binaphthyl-2-Yl]Pyrrolidine-2-Carboxamide: An Organocatalyst for the Direct Aldol Reaction. *Org. Synth.* **2011**, *88*, 317–329. https://doi.org/10.15227/orgsyn.088.0317.

(19) D'Elia, V.; Zwicknagl, H.; Reiser, O. Short α/β-Peptides as Catalysts for Intra- and Intermolecular Aldol Reactions. *J. Org. Chem.* **2008**, *73* (8), 3262–3265. https://doi.org/10.1021/jo800168h.

(20) Mori, K.; Katoh, T.; Suzuki, T.; Noji, T.; Yamanaka, M.; Akiyama, T. Chiral Phosphoric Acid Catalyzed Desymmetrization of Meso-1,3-Diones: Asymmetric Synthesis of Chiral Cyclohexenones. *Angew. Chem. Int. Ed.* **2009**, *48* (51), 9652–9654. https://doi.org/10.1002/anie.200905271.
(21) Kametani, T.; Suzuki, K.; Nemoto, H. Asymmetric Synthesis of (-) -3.Beta.-Hydroxy-17-Methoxy-D-Homo-18-nor-5.Alpha.-Androsta-13,15,17-Triene. *J. Org. Chem.* **1980**, *45* (11), 2204–2207. https://doi.org/10.1021/jo01299a033.
(22) Yin, S.; Sugimoto, K.; Nemoto, H.; Matsuya, Y. Synthetic Study Towards Construction of Potential Scaffold of Antitumor Agents Andrastins. *Heterocycles* **2017**, *95* (1), 187–199. https://doi.org/10.3987/COM-16-S(S)2.
(23) Dolomanov, O. V.; Bourhis, L. J.; Gildea, R. J.; Howard, J. a. K.; Puschmann, H. OLEX2: A Complete Structure Solution, Refinement and Analysis Program. *J. Appl. Crystallogr.* **2009**, *42* (2), 339–341. https://doi.org/10.1107/S0021889808042726.
(24) Sheldrick, G. M. A Short History of SHELX. *Acta Crystallogr., Sect. A: Found. Adv.* **2008**, *64* (1), 112–122. https://doi.org/10.1107/S0108767307043930.
(25) Sheldrick, G. M. Crystal Structure Refinement with SHELXL. *Acta Crystallogr., Sect. C: Struct. Chem.* **2015**, *71* (1), 3–8. https://doi.org/10.1107/S2053229614024218.
(26) Sheldrick, G. M. SHELXT – Integrated Space-Group and Crystal-Structure Determination. *Acta Crystallogr., Sect. A: Found. Adv.* **2015**, *71* (1), 3–8. https://doi.org/10.1107/S2053273314026370.
