# Supplementary material for: Identification of a Privileged Scaffold for Inhibition of Sterol Transport Proteins through the Synthesis and Ring Distortion of Diverse, Pseudo-Natural Products
Source: ACS Cent Sci. 2025 Jan 9;11(1):136–46. doi: 10.1021/acscentsci.4c01657 (PMC11758220; doi:10.1021/acscentsci.4c01657)
Supplement: Supplementary file 7 — oc4c01657_si_007.pdf [file oc4c01657_si_007.pdf]

oc-2024-01657d.R1

Name: Peer Review Information for "Identification of a privileged scaffold for inhibition of sterol transport proteins through the synthesis and ring distortion of diverse, pseudo-natural products"

First Round of Reviewer Comments

Reviewer: 1

Comments to the Author

The authors have identified a lead compound for the potent and selective binding of Aster-A (the GRAMD1A protein), a member of the sterol transport proteins (STPs). STPs are implicated in a range of diseases so that their selective targeting by a small molecule (drug) could be important as a tool/probe or therapeutic. Inhibition of Aster-A affects autophagosome biogenesis (the corresponding author's postdoctoral work) and the authors suggest this as a pathway for development of their lead series (see below). This work builds on prior efforts by Laraia group to use the sterol A/B ring as a recognition unit for STP binding with replacement of the sterol C/D rings by diverse heterocycles (see esp. Ref. 23). These heterocycles can be easily substituted to create libraries that are then searched for selectivity among STPs. Along the way, they find that an indole-fused decalin undergoes oxidative rearrangement and, surprisingly, provides the most selective series of ASTER-A ligand.

Overall, this could be important work, but its main messages are lost in the way the paper is framed. First, the authors' main finding is that 19a, which they name Asteroxin-1, shows high selectivity for Aster A. However, the authors provide little framework, context or background information to support why this Aster-A inhibition is important. Greater description of a hypothetical link between Aster-A, autophagosome biogenesis, hypothetical roles in cancer and prior art in chemotherapy sensitization would help establish why the work is significant. The synthetic chemistry is quite standard, so the authors should focus on this exciting and underexplored biology.

Second, the authors' series are derived from the substrates of these transport proteins (sterols)—a common strategy in the development of a probe, tool or drug. For example, as the authors certainly know, there are common protease, neuroamidase and nucleosidase inhibitors (to name the obvious ones) that mimic an enzyme substrate as a design feature. Sometimes, the substrate mimics undergo substantial change during the exploratory or optimization process. The authors don't describe their work this way, and I don't understand why. Instead, they note a variety of

“thought experiments” from academia that have little correspondence to this manuscript and even to one another. This is a lost opportunity to put the work in its proper context and makes it very hard for referees to evaluate its significance and novelty (much like the importance of Aster A). Instead, the authors should compare/contrast their work to the development of transport protein inhibitors, perhaps selective STP inhibitors, if there are enough to compare. As it is, the first two paragraphs of the introduction confuse the reader, rather than inform him/her. Just tell us what you discovered, how it was discovered and why it’s important.

The identification of “Asteroxin-1” for the selective inhibition of cholesterol transport by Aster-A is a nice start for biological discovery. However, the chemistry and biological studies are not frontier or advanced enough for publication in ACS Central Science. Maybe describing the work in the context of “pseudo-natural products” was an attempt by the authors enhance the novelty/significance of the chemistry, but it’s not convincing and seems post-hoc. The scholarship would be substantially improved by different framing and would be far more educational for student readers.

Reviewer: 2

Comments to the Author

This is a very interesting report on identification of several scaffolds, derived from natural products, which show activity and selectivity towards different sterol transport proteins. Authors systematically and convincingly present the logic of the design of new compounds and preliminary studies, which allow them to determine the biological activity of these new entities against certain targets. The supplementary materials are of excellent quality, giving very detailed information not only on the synthesis and spectral characteristic of the compounds, but also trying to explain (sometimes with support of appropriate experiments) the mechanisms of many interesting transformations. The only think that I would recommend to add, for clarity, is short description of the most active compounds, in terms of their activity and selectivity, maybe in the form of table (abstract or summary). It would make the results of the work more pronounced.

Reviewer: 3

Comments to the Author

The manuscript of Bro et al describes the identification of a privileged scaffold for inhibition of sterol transport proteins (STPs). This was achieved through the fusion of a primary sterol scaffold and natural product fragments followed by ring distortions. The authors synthesized a cholic acid-inspired library consisting of 69 compounds with 13 distinct scaffolds, which were designed by the

combination of the pseudo-natural product (PNP) and complexity-to diversity (CtD) strategies. The library was screened against a broad panel of the STPs using FP and DSF assays. A morpholine substituted spirooxepinoindole (19a, named (±)-Asteroxin-1) was identified as a very potent Aster-A inhibitor with an IC50 of 0.77 μM and a very promising selectivity profile. Screening of the enantiomers revealed (−)-Asteroxin-1 as the active enantiomer. (−)-Asteroxin-1 binding to the different Aster proteins was modelled to understand its selectivity.,

The work is very well designed and executed. The authors present in a clear manner their results, which have been recorded in detail.

In 2021, the authors presented a similar strategy to develop STPs inhibitors (ref 23, Identification of Inhibitors of Cholesterol Transport Proteins Through the Synthesis of a Diverse, Sterol-Inspired Compound Collection. Angew. Chem. Int. Ed. 2021, 60 (51), 26755–26761), however the present work presents its own novelty.

Given the importance of the discovery of inhibitors of STPs and the elegant approach followed for the identification of the privileged scaffold, I recommend publication of this manuscript in ACS Central Science after minor revision.

Specific comment

The authors have to compare the newly identified Aster-A inhibitor with any existing inhibitor and discuss the comparison.

Reviewer: 4

Comments to the Author

The present manuscript is a follow-up to a previous Angew. Chem. Int. Ed. paper from 2021 by the same authors. While both manuscripts share sterol-inspired libraries to identify STP inhibitors, the ACS Central Science paper introduces significant advancements: by integrating ring distortion methods and the authors achieve selective Aster-A inhibition with (−)-Asteroxin-1. Aster-A is involved in sterol transport and autophagy. The SAR analysis presented is comprehensive, detailing substituent effects on Aster-A selectivity and STP interaction profiles. However, further clarification on how specific substituent patterns influence selectivity across different STPs could be helpful. Differential scanning fluorimetry (DSF) and fluorescence polarization (FP) assays were used to characterize compound activity. The proposed binding mode of (−)-Asteroxin-1 with Aster-A, supported by molecular docking, is plausible, yet X-ray crystallography would be the icing on the cake. Further elaborating on biochemical consequences of Aster-A inhibition - perhaps with cellular assays to demonstrate functional outcomes - would enhance the manuscript's impact and demonstrate therapeutic relevance. The characterization of new compounds is thorough, and I did not find any mistakes. I recommend acceptance of this study that clearly expands on the ACIE communication.

Author's Response to Peer Review Comments:

Please find the response to the editorial and reviewer comments attached here.

Response to formatting needs and reviewer comments

Manuscript ID: oc-2024-01657d.

Formatting needs

SYNOPSIS LENGTH: The synopsis should be no more than 200 characters (including spaces).

The synopsis has been shortened to comply with the character limit.

SI PARAGRAPH: Please provide a brief description of the contents of the supplementary material at the end of the manuscript file before the Acknowledgments and References sections with the heading "Supporting Information." Please avoid long paragraphs or lists of Supplemental figure captions.

The section "Data availability" has been changed to "Supporting information". The section has been formatted to a list containing the supplementary files with a brief description of the contents. Supplementary discussions, figures, schemes, and tables are not mentioned.

Reviewer comments

We thank all three reviewers for their constructive feedback and helpful comments. We have now addressed all the comments below, and believe the manuscript is suitable for publication in its current form.

Reviewer 1:

We thank the reviewer for the careful evaluation of our manuscript and helpful comments. We have addressed the concerns below:

However, the authors provide little framework, context or background information to support why this Aster-A inhibition is important. Greater description of a hypothetical link between Aster-A, autophagosome biogenesis, hypothetical roles in cancer and prior art in chemotherapy sensitization would help establish why the work is significant.

We agree that a more detailed description about the importance of Aster-A inhibition with small molecules would be useful. Thus, we have included description of functional redundancy observed with STPs in the introduction. Additionally, we have further elaborated the role of Aster-A in autophagy and the therapeutic potential of Aster-A inhibition in cancer treatment in the results and discussion.

Second, the authors' series are derived from the substrates of these transport proteins (sterols) - a common strategy in the development of a probe, tool or drug. For example, as the authors certainly know, there are common protease, neuroamidase and nucleosidase inhibitors (to name the obvious ones) that mimic an enzyme substrate as a design feature. Sometimes, the substrate mimics undergo substantial change during the exploratory or optimization process. The authors don't describe their work this way, and I don't understand why. Instead, they note a variety of "thought experiments" from academia that have little correspondence to this manuscript and even to one another. This is a lost opportunity to put the work in its proper context and makes it very hard for referees to evaluate its significance and novelty (much like the importance of Aster A). Instead, the authors should compare/contrast their work to the development of transport protein inhibitors, perhaps selective STP inhibitors, if there are enough to compare. As it is, the first two paragraphs of the introduction confuse the reader, rather than inform him/her. Just tell us what you discovered, how it was discovered and why it's important.

We agree that using a known ligand for the target in question in the development of bioactive compounds is a widely used strategy and a common feature in many of the strategies for the synthesis of natural product-derived and -inspired compound libraries. We have included this

this alongside some examples of enzyme inhibitors mimicking the natural substrates at the end of the introduction. Although we seek inspiration from a fragment of cholic acid (the AB ring system), we would like to point out that we do not use the whole tetracyclic scaffold of cholic acid. Additionally, the spirooxepinoindole scaffold is no longer steroidal and shares very little resemblance to cholic acid, which does not bind Aster-A. We completely agree that adding more information about current selective STP inhibitors and how (−)-asteroxin-1 compares is very useful. We have added a brief description of current inhibitors of STPs in the introduction. Furthermore, we have compared (−)-asteroxin-1 to other known Aster-A inhibitors in terms of selectivity and potency in the results.

However, the chemistry and biological studies are not frontier or advanced enough for publication in ACS Central Science. Maybe describing the work in the context of "pseudo-natural products" was an attempt by the authors enhance the novelty/significance of the chemistry, but it's not convincing and seems post-hoc. The scholarship would be substantially improved by different framing and would be far more educational for student readers.

We would like to point of that the PNP concept is a well-established strategy (reviewed recently here: https://pubs.acs.org/doi/full/10.1021/jacs.1c11270) and has been used successfully in our group in other cases (https://doi.org/10.1002/anie.202111639, https://doi.org/10.1002/cbic.202200555, https://doi.org/10.1021/acs.jmedchem.3c01059). Importantly, the work presented here is not exclusively PNP-based. In fact, the integrative approach combining several library synthesis strategies proved to be crucial, since the CtD-like ring distortions led to the identification of the spirooxepinoindole. We have added a sentence to this effect in the conclusion.

Reviewer 2:

We thank the reviewer for the positive assessment of our manuscript, and particularly the appreciation of the supplementary materials. We have addressed the minor comment below.

The only think that I would recommend to add, for clarity, is short description of the most active compounds, in terms of their activity and selectivity, maybe in the form of table (abstract or summary). It would make the results of the work more pronounced.

We completely agree and to address this, we have included a summary of the SAR profiling in the form of a figure in the supporting information (Figure S11, reproduced below) to support the existing text. It describes how the different substitution patterns on the spirooxepinoindole influence activity and selectivity across the STP panel using some of the most active compounds as examples.

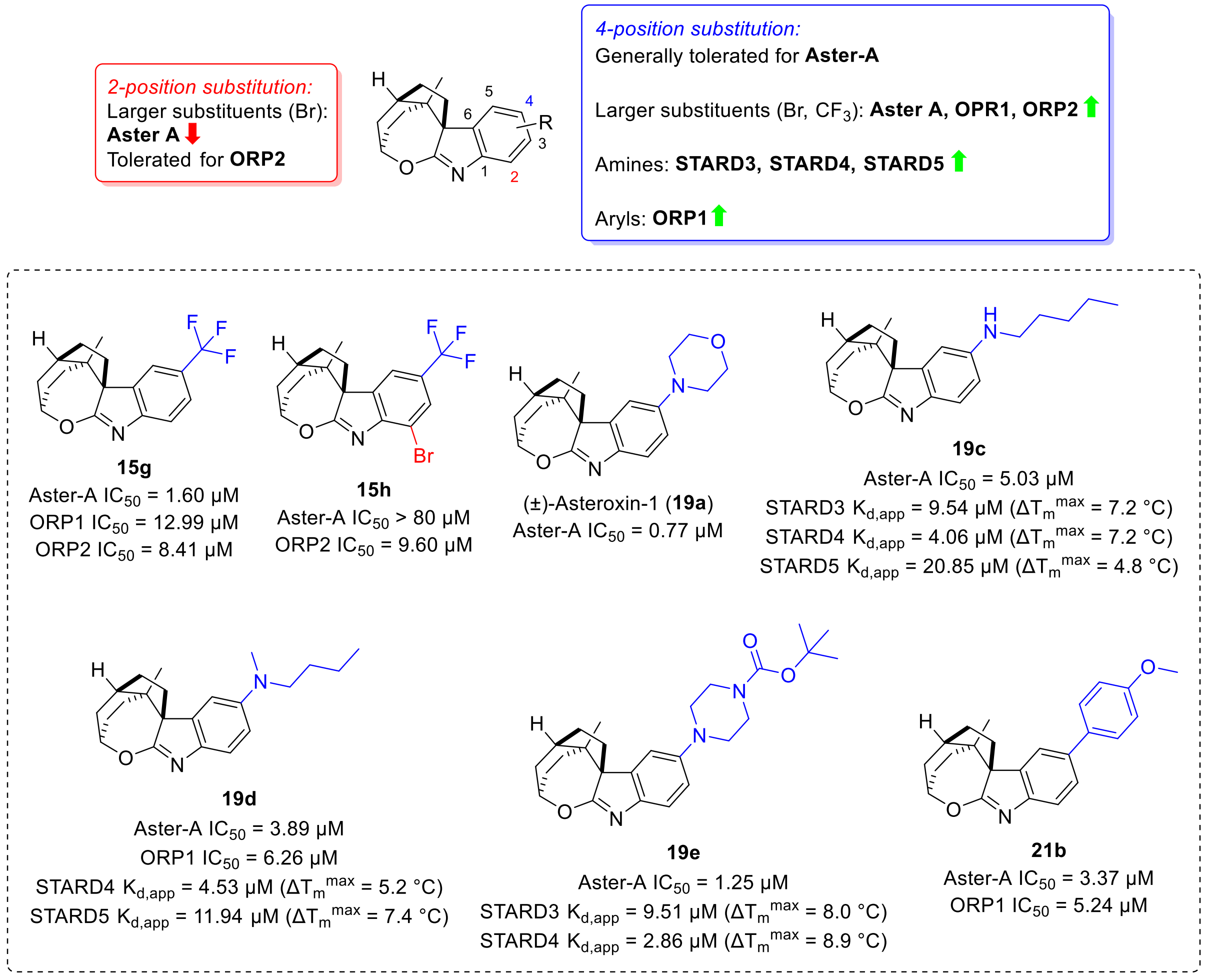

Reviewer 3:

We thank the reviewer for the positive assessment of our manuscript, and for appreciating the design strategy and presentation of the results. We have addressed the minor comment below.

The authors have to compare the newly identified Aster-A inhibitor with any existing inhibitor and discuss the comparison.

This is an excellent suggestion, which we have addressed by including the known Aster-A inhibitors autogramin-2 and U18666A and their activity towards the STP panel in Table 1. In the text, the superior selectivity of (−)-asteroxin-1 is highlighted by comparison to these known inhibitors which all have additional activities within the panel of STPs.

Reviewer 4:

We thank the reviewer for the positive assessment of our manuscript, and in particular the appreciation of SAR analysis. We have addressed the minor comments below.

However, further clarification on how specific substituent patterns influence selectivity across different STPs could be helpful.

We fully agree and to address this, we have included a summary of the SAR profiling in form of a figure in the supporting information (Figure S11, please see above in response to reviewer 2) to support the existing text. It describes how the different substitution patterns on the spirooxepinoindole influence activity and selectivity across the STP panel.

The proposed binding mode of (−)-Asteroxin-1 with Aster-A, supported by molecular docking, is plausible, yet X-ray crystallography would be the icing on the cake.

We completely agree that X-ray crystallography would be a valuable addition. Consequently, this has been attempted prior to submission of the manuscript with no success so far. This is still ongoing but is unlikely to be resolved within a reasonable timeframe.

Further elaborating on biochemical consequences of Aster-A inhibition - perhaps with cellular assays to demonstrate functional outcomes - would enhance the manuscript's impact and demonstrate therapeutic relevance.

To explore the potential application of the spirooxepinoindoles in a cellular setting we have performed target engagement experiments in Jurkat cells. Using an isothermal shift assay performed at 50 °C, we were able to determine the changes in thermal stability of Aster-A upon compound treatment (new Figure S14, reproduced below). All compounds tested stabilised Aster-A to some degree, with **19e** showing the most pronounced effects, after the control compound autogramin-2. This is in line with biochemical thermal shift assays, where **19e** stabilized Aster-A more than (−)-asteroxin-1, however it contrasts the FP experiments, where (−)-asteroxin-1 was the most potent analogue. While these early cellular results are promising, additional target engagement assays which do not depend on thermal stability will be required, as well as assays to quantitatively compare cell permeability. These, along with additional functional assays measuring the outcome of Aster-A inhibition, are currently being developed but require a substantial amount of extra work and time. As such, they will be the subject of a separate publication in the future.

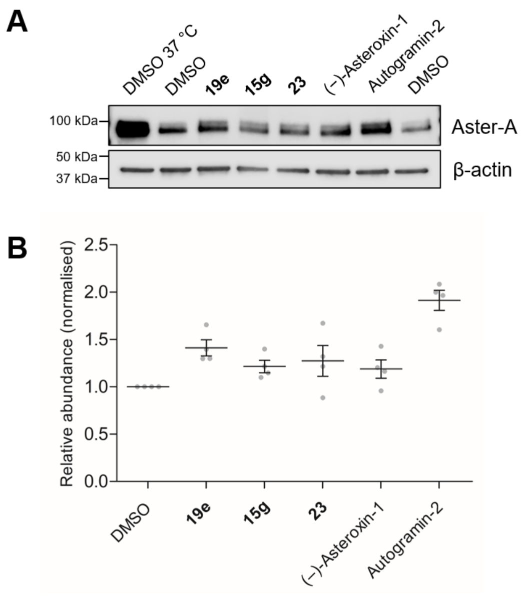

oc-2024-01657d.R2

Name: Peer Review Information for "Identification of a privileged scaffold for inhibition of sterol transport proteins through the synthesis and ring distortion of diverse, pseudo-natural products"

Second Round of Reviewer Comments

Reviewer: 3

Comments to the Author

The authors have successfully addressed reviewers' comments. I suggest publication in the current form.

Reviewer: 1

Comments to the Author

The authors have responded productively to the comments of the referees (In fact, the authors have gone above-and-beyond in their revision. The referees' comments were rather superficial and I apologize to the authors for the superficiality of the peer review). The isothermal shift assay data are very interesting and point to great science ahead. I encourage the authors to include Figure S14 in the manuscript itself, at the discretion of the editorial office. It transforms a standard SAR campaign into a promise for deeper questions and greater insight. I consider that a win, not a loss.

I am still not comfortable with the authors' framing of this paper as a pseudo-natural product (PNP) study. It's the sort of academic jargon that causes industrial medicinal chemists (or historians) to roll their eyes. What is conceptually different from early work on steroids (even truncated steroids), tropane alkaloids, beta-lactams or innumerable other NP series studied a century ago? I'll consider it artistic license to frame the paper this way, but my particular area of science also suffers from the invention of new terms to rebrand old ideas, at the cost of legitimate science. I only ask the authors to keep this trend in mind. It steers students and resources towards settled areas instead of pushing back the frontiers.

Reviewer: 2

Comments to the Author

n/a

Reviewer: 4

Comments to the Author

The authors have done a great job in revising the manuscript. I have no further objections. Congrats to the authors.

Author's Response to Peer Review Comments:

Please fine the response to the reviewer comments attached.

Response reviewer comments

Manuscript ID: oc-2024-01657d.R1.

Reviewer comments

We thank all four reviewers for their careful evaluation and positive assessment of our manuscript. Thank you to reviewers 2, 3, and 4 for their recommendation to publish our manuscript in *ACS Central Science*. We have now addressed the comments from reviewer 1 below, and believe the manuscript is suitable for publication in its current form.

Reviewer 1:

We thank the reviewer for the positive assessment of our manuscript, and the appreciation of the revisions and the cell biology carried out. We have addressed the minor comments below:

The authors have responded productively to the comments of the referees (In fact, the authors have gone above-and-beyond in their revision. The referees' comments were rather superficial and I apologize to the authors for the superficiality of the peer review). The isothermal shift assay data are very interesting and point to great science ahead. I encourage the authors to include Figure S14 in the manuscript itself, at the discretion of the editorial office. It transforms a standard SAR campaign into a promise for deeper questions and greater insight. I consider that a win, not a loss.

We completely agree that the isothermal shift assay data is very important and have therefore moved Figure S14 to the manuscript as suggested (now Figure 4).

I am still not comfortable with the authors' framing of this paper as a pseudo-natural product (PNP) study. It's the sort of academic jargon that causes industrial medicinal chemists (or historians) to roll their eyes. What is conceptually different from early work on steroids (even truncated steroids), tropane alkaloids, beta-lactams or innumerable other NP series studied a century ago? I'll consider it artistic license to frame the paper this way, but my particular area of science also suffers from the invention of new terms to rebrand old ideas, at the cost of legitimate science. I

only ask the authors to keep this trend in mind. It steers students and resources towards settled areas instead of pushing back the frontiers.

We understand that naming synthetic strategies can be divisive in the field; however, we would like to clarify how we personally use these strategies to guide and structure our thought process when designing compound collections. We believe that the need for well-defined strategies and the differentiation between them provides theoretical frameworks that simplify a project, allowing faster access to compounds of desired structures and properties. We would like to highlight that while the final spirooxepinoindole scaffold shares very little resemblance to a steroidal scaffold, the initial PNP-fusion between the AB-ring found in cholic acid and the indole fragment was crucial for the identification of the spirooxepinoindole scaffold, as it enabled the subsequent synthesis of four different scaffolds by varying the oxidation conditions.
